# High similarity of phylogenetic profiles of rate-limiting enzymes with inhibitory relation in Human, Mouse, Rat, budding Yeast and *E*. *coli*

**DOI:** 10.1186/1471-2164-12-S3-S10

**Published:** 2011-11-30

**Authors:** Min Zhao, Hong Qu

**Affiliations:** 1 Center for Bioinformatics, National Laboratory of Protein Engineering and Plant Genetic Engineering, College of Life Sciences, Peking University, Beijing, 100871, P.R. China

## Abstract

**Background:**

The phylogenetic profile is widely used to characterize functional linkage and conservation between proteins without amino acid sequence similarity. To survey the conservative regulatory properties of rate-limiting enzymes (RLEs) in metabolic inhibitory network across different species, we define the enzyme inhibiting pair as: where the first enzyme in a pair is the inhibitor provider and the second is the target of the inhibitor. Phylogenetic profiles of enzymes in the inhibiting pairs are further generated to measure the functional linkage of these enzymes during evolutionary history.

**Results:**

We find that the RLEs generate, on average, over half of all *in vivo* inhibitors in each surveyed model organism. And these inhibitors inhibit on average over 85% targets in metabolic inhibitory network and cover the majority of targets of cross-pathway inhibiting relations. Furthermore, we demonstrate that the phylogenetic profiles of the enzymes in inhibiting pairs in which at least one enzyme is rate-limiting often show higher similarities than those in common inhibiting enzyme pairs. In addition, RLEs, compared to common metabolic enzymes, often tend to produce ADP instead of AMP in conservative inhibitory networks.

**Conclusions:**

Combined with the conservative roles of RLEs in their efficiency in sensing metabolic signals and transmitting regulatory signals to the rest of the metabolic system, the RLEs may be important molecules in balancing energy homeostasis via maintaining the ratio of ATP to ADP in living cells. Furthermore, our results indicate that similarities of phylogenetic profiles of enzymes in the inhibiting enzyme pairs are not only correlated with enzyme topological importance, but also related with roles of the enzymes in metabolic inhibitory network.

## Background

The phylogenetic profile of a given protein is a string that encodes the presence or absence of all the homologs of the protein in each organism [1]. The similarity of phylogenetic profiles has been widely used to characterize functional linkage between proteins having no amino acid sequence similarity with each other [2-5]. The phylogenetic profiles of proteins with contextual information such as interactions, pathways, and cellular localizations have been widely studied. It is often supposed that proteins which tend to co-occur across species are more likely to co-evolve. The similarity of phylogenetic profiles is also correlated better with topological properties of metabolic enzymes such as degree and betweenness centrality, but independent of enzyme network importance [6].

The RLEs are a class of important enzymes playing roles of flux control in cell [7]. According to modern flux control theory, RLEs often catalyze the slowest step in a metabolic pathway with a high flux control coefficient. Thus they are the ideal molecules to link metabolic pathways to regulatory networks. Based on their importance on the flux control and regulation of metabolic pathway, we built the first evidence-based database by systematic collecting RLEs in Human, Mouse, Rat, budding Yeast and *E. coli*[8]. Our recent study reveals that RLEs in human liver often locate in the branch points and produce nearly half of *in vivo* metabolic enzyme inhibitors [9]. However, the extent to which RLEs interact with enzyme inhibitors in liver or other tissue is less important in investigating the phylogenetic conservation of the regulatory mechanisms. The crucial question is whether these conservative RLEs involving inhibitory networks also maintain a higher functional linkage as expected.

In this study, enzyme inhibiting pairs and inhibitory network were constructed using enzyme inhibitor data from BRENDA database [10,11]. We found that the RLEs provided over half of all *in vivo* inhibitors in five surveyed model organisms. These inhibitors inhibited over 85% targets *in vivo* in the metabolic inhibitory network. Additionally, we found that the phylogenetic profiles of RLEs in inhibiting pairs often showed higher similarities than those of common enzymes, which implied that the inhibitory relationship between two enzymes in an inhibiting pair was conservative across different species.

## Results

In total, 230 RLEs were collected from RLEdb [8]. Based on these 230 RLEs, we extracted species specific RLE datasets using the enzyme organism distribution information from KEGG LIGAND database [12,13]. In the five most common model organisms including Human, Mouse, Rat, budding Yeast and *E. coli*, there are 204, 201, 175, 139, 121 RLEs respectively (Additional file 1). According to the six pathway categories from KEGG (i.e., Carbohydrate, Lipid, Nucleotide, Amino acid, Cofactor and vitamin and Others molecular metabolism) as in [9], we investigated the functional distribution of all RLEs in each organism.

### RLEs produce over half enzymes inhibitors

**Figure 1:**
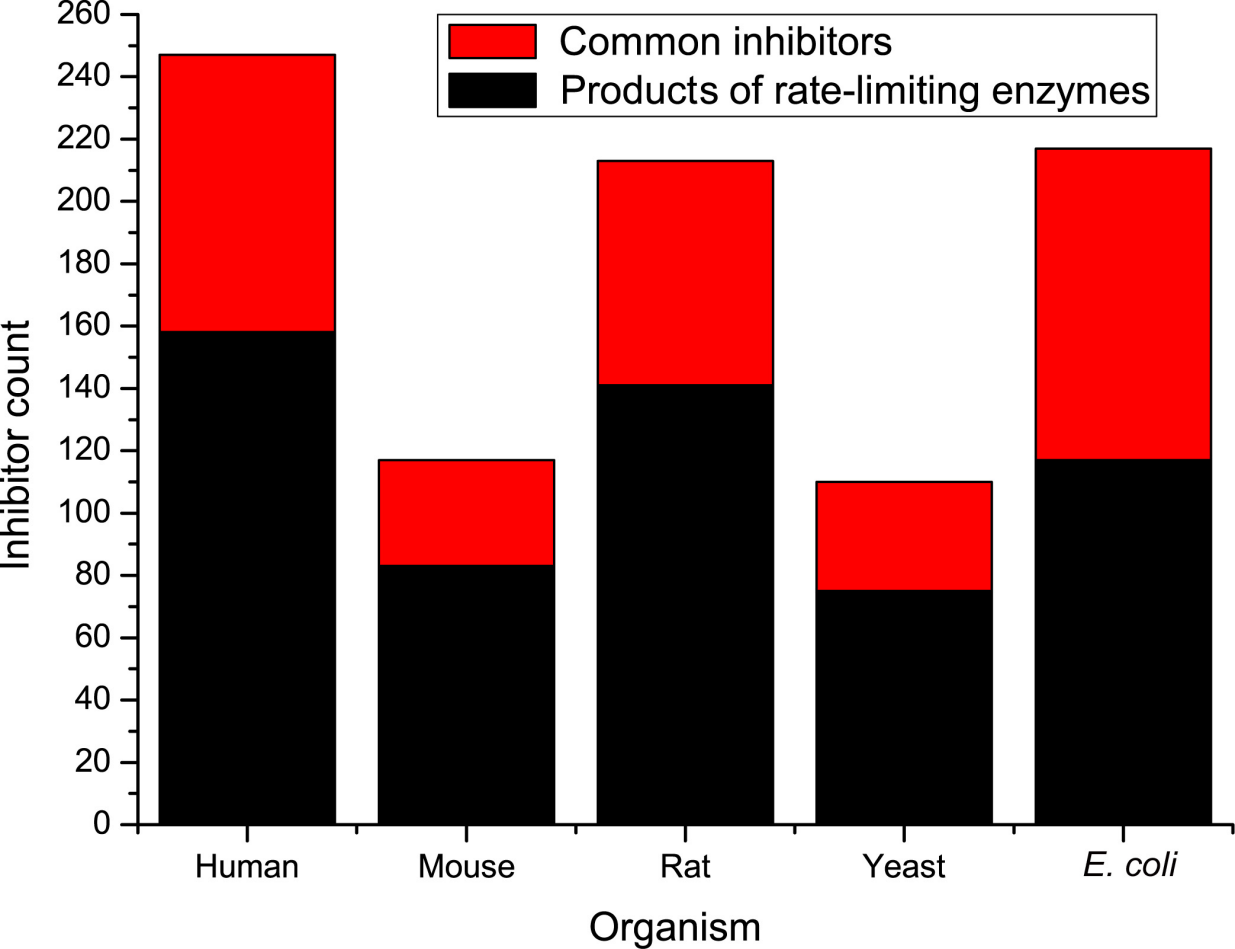
**Distribution of inhibitors in Human, Mouse, Rat, budding Yeast and *E*. *coli*.** Each bar in figure shows the inhibitor count in an organism. The black part represents the amount of inhibitors produced by RLEs. The red one represents the amount of other common *in vivo* inhibitors not produced by RLEs.

Our survey of *in vivo* inhibitors in Human, Mouse, Rat, budding Yeast and *E. coli* revealed that RLEs produced 64%, 71%, 66%, 68% and 54% *in vivo* enzyme inhibitors respectively. As shown in Figure 1, for instance, there are 247 *in vivo* inhibitors in Human, among which 158 are produced by 186 RLEs. We identified that the *in vivo* inhibitors were significantly enriched in the products of RLEs as compared in all the metabolic compounds in five organisms (hypergeometric tests, all p value <0.001). These results led us to ask following questions: Why such a high proportion of *in vivo* inhibitors were products of RLEs and how could metabolic network be influenced by such a high proportion of rate-limiting producing inhibitors. To address these questions, we compared the inhibiting efficiency of inhibitors produced by RLEs with that of all the inhibitors from each metabolic pathway.

### Eighty-Twenty rule style in enzyme inhibitory network (RLEs could inhibit most targets *in vivo* via natural products)

**Figure 2:**
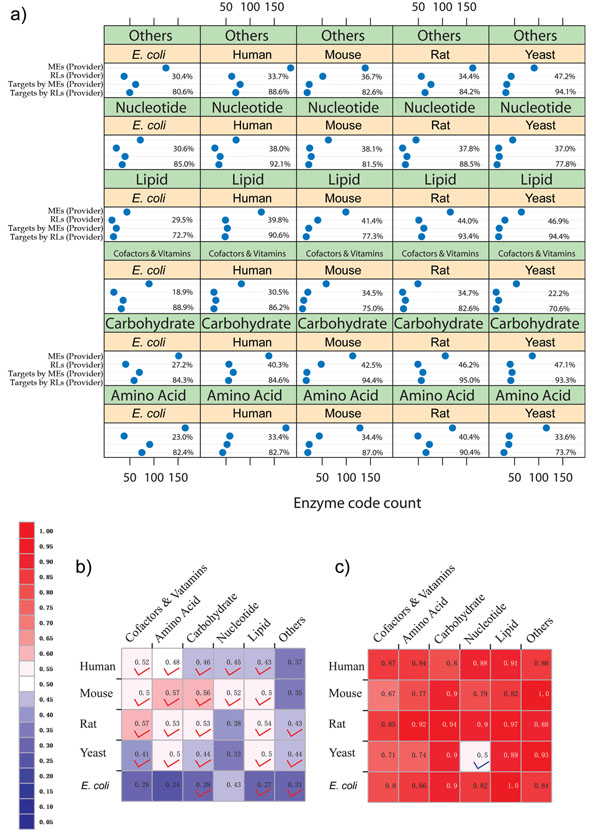
**Distribution of RLEs in inhibitory network.** (a) Dot plots of enzyme counts for each pathway from four following aspects for Human, Mouse, Rat, budding Yeast and *E*. *coli*. The statistic counts for certain pathways of an organism are grouped in each cell. In each cell, MEs (Provider) mean count of all metabolic enzymes which produce inhibitors, RLEs (Provider) mean count of RLEs which produce inhibitors, Targets by MEs (Provider) mean count of inhibited enzymes by all the metabolic enzymes, Targets by RLEs (Provider) mean count of inhibited enzymes by RLEs. The above percentage in each cell is the ratio between the first line and the second line meaning the fraction of the RLEs (Provider) to all metabolic enzymes (Provider); the bottom percentage is the ratio between the third line and the fourth line meaning the fraction of Targets by the RLEs (Provider) to those by all metabolic enzymes (Provider). (b) Colour grid for fraction of the inhibited RLEs to all inhibited metabolic enzymes for each pathway in five model species. The inhibited targets are enriched in RLEs are red-marked (hypergeometric tests, p value <0.05). (c) Colour grid for fraction of the inhibited RLEs by RLEs to inhibited RLEs by all the metabolic enzymes in each pathway from five model species. Hypergeometric tests reveal that all the inhibited RLEs are preferred inhibited by RLEs except one blue-marked (p value < 0.05).

Based on the number of how many enzymes were inhibited by products of enzymes, the efficiency of RLEs was calculated in metabolic inhibitory networks. Combining the metabolic enzyme inhibitor information in BRENDA database [10,11] and enzyme product information in KEGG LIGAND database [12,13], we constructed the inhibiting enzyme pairs dataset (see Methods). As illustrated in Figure 2a, the products of RLEs cover most of the targets ranging from 70.6% to 95.0% in each pathway category. Totally, the products of RLEs have effects on 85.15% of all targets from all pathway categories in five model organisms. However, only a small number of RLEs participate in inhibitory network as inhibitor provider ranging from18.9% to 47.2%. On average, only 35.8% inhibitor providers are RLEs. These two aspects, taken together, suggest that RLEs follow an Eighty-Twenty rule style in metabolic inhibitory network. More interestingly, the inhibiting regulation systems consisting of these *in vivo* inhibitors generated by RLEs might also be initiated in a rate-limiting mode.

### The efficiency of RLEs in sensing metabolic signals as targets of enzyme inhibitors

To describe how effectively enzyme inhibitor signals on the RLEs were spread to the metabolic system, all the fractions of RLEs as inhibitor targets to all targets from each pathway category in five model organisms were summarized in Figure 2b. The average fraction in human is over 0.45, which suggests that RLEs tend to be inhibited by enzyme inhibitors. As marked in Figure 2b, the inhibited targets are enriched in RLEs in five sixths pathway categories in four eukaryotic model organisms (hypergeometric tests, p value <0.05).

Besides both important roles as inhibitor providers and targets, further analysis (Figure 2c) shows that most targets of RLEs are inhibited by other RLEs for each pathway category in five model organisms. It implies that the overall targets of RLEs tend to be inhibited by themselves. All fractions of the inhibited RLEs by other RLEs to all inhibited RLEs by all metabolic enzymes in each pathway category in five model organisms were calculated. The average fraction in human is more than 0.86, which means that eighty-six percent of RLEs can be inhibited by other RLEs. Further hypergeometric tests reveal that all the inhibited RLEs are preferably inhibited by RLEs except one blue-marked as shown in Figure 2c (all p value < 0.05).

### The products of RLEs have effects on the majority of targets of cross pathway regulatory system

**Figure 3:**
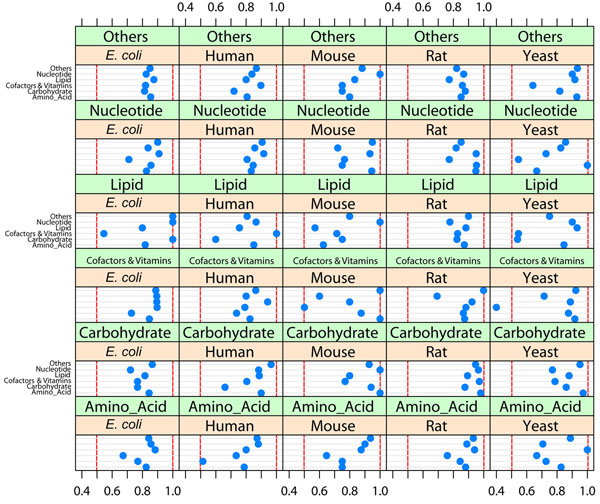
**Inhibitory relation between six pathway categories for Human, Mouse, Rat, Yeast, *E*. *coli*.** The regulatory relations for certain pathways of an organism are grouped in each cell, the blue dot in the first line means the ratio of inhibited enzymes by RLEs from Others metabolic pathway category to the inhibited enzymes by all metabolic enzyme in the same pathway; the dot in the second line means the ratio of inhibited enzymes by RLEs from Nucleotide metabolic pathway category to the inhibited enzymes by all metabolic enzyme in the same pathway; the dot in the third line means the ratio of inhibited enzymes by RLEs from Cofactors and Vitamins metabolic pathway category to the inhibited enzymes by all metabolic enzyme in the same pathway; the dot in the fourth line means the ratio of inhibited enzymes by RLEs from Lipid metabolic pathway category to the inhibited enzymes by all metabolic enzymes in the same pathway; the dot in the fifth line means the ratio of inhibited enzymes by RLEs from Carbohydrate metabolic pathway category to the inhibited enzymes by all metabolic enzyme in the same pathway; the dot in the sixth line means the ratio of inhibited enzymes by RLEs from Amino acid metabolic pathway category to the inhibited enzymes by all metabolic enzyme in the same pathway.

Cross pathway regulations are crucial to maintaining homeostasis [14,15]. Inhibiting the targets in cross pathways should be one of the quickest cross pathway regulation of enzyme inhibitors. High efficiencies of RLEs as inhibitor providers cross pathway inhibitory network were also found in five model organisms. Here the 44607 cross-pathway inhibiting pairs from five model organisms were isolated if the pathway annotations of inhibitor provider and target in a pair were different. Only eight efficiency fractions are less than 60%, and all the left 172 fractions are higher than 60% (Figure 3). The average fraction in total is 83.2%, which reveals that the RLEs, as inhibitor providers, have effect on over 83% of the cross pathway inhibitory relationship. For efficient metabolic system, it is important for the cellular metabolic network to balance flux precisely between different pathways. The high efficiency of RLEs in cross pathway inhibitory relation networks highlights their roles in maintaining metabolic homeostasis between different pathways via inhibitory regulation.

### The phylogenetic profiles of RLEs in inhibiting pairs often show higher similarities than those in common inhibiting enzyme pairs

**Figure 4:**
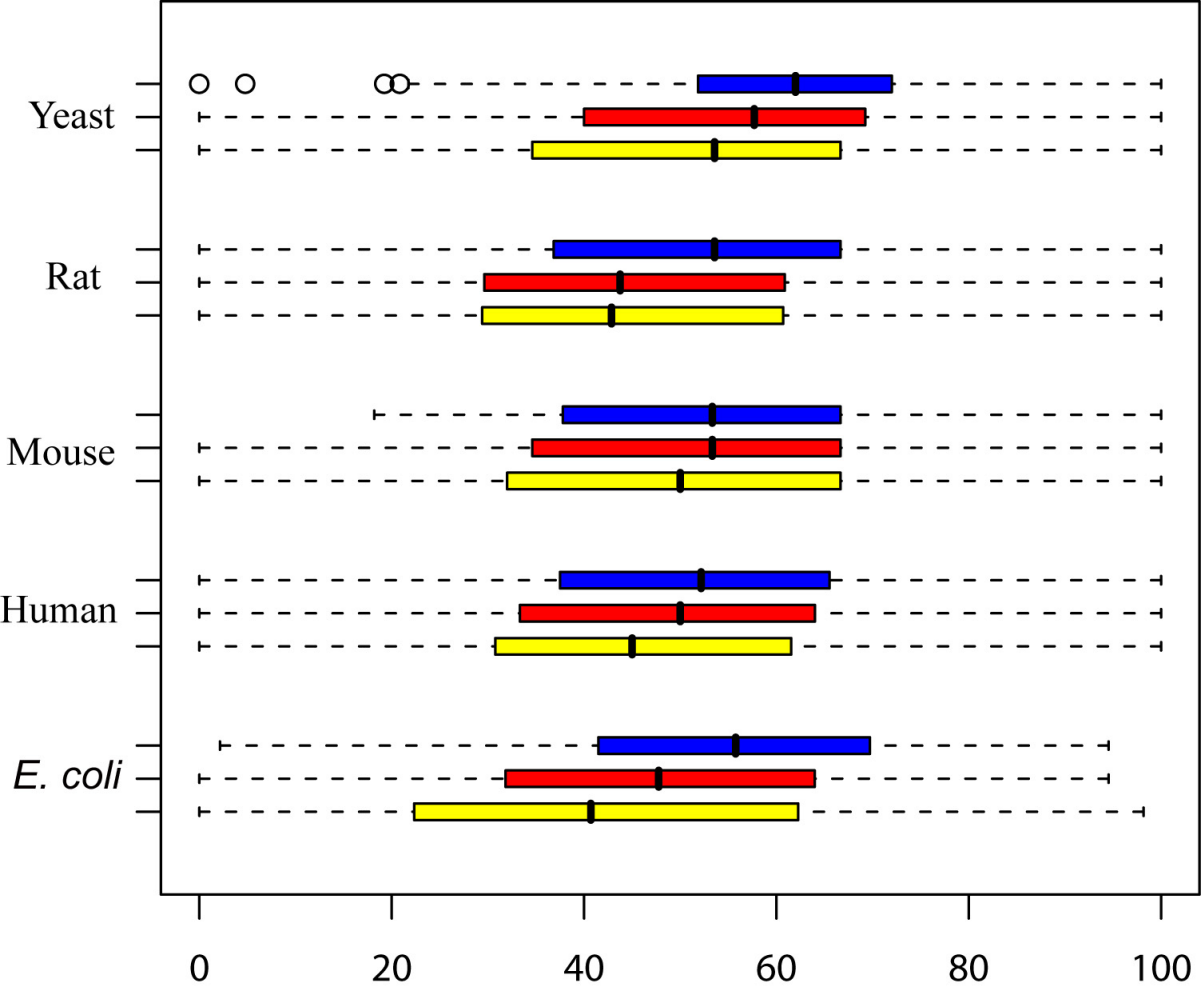
**The average phylogenetic Jaccard coefficients for the inhibiting enzyme pairs in Human, Mouse, Rat, budding Yeast and *E*. *coli*.** The higher score indicates higher functional correlation in an enzyme pair. The product of the first enzyme in the pair is the inhibitor for the second enzyme in the pair. For each organism, the yellow bar represents the average phylogenetic Jaccard coefficients from the common enzyme pairs whose inhibiting enzyme and inhibited enzyme are both from all the metabolic enzyme; the red one represents the average phylogenetic Jaccard coefficients from the RLE pairs whose inhibiting enzyme is from all the RLE; the blue one represents the average phylogenetic Jaccard coefficients from the pairs whose inhibiting enzyme and inhibited enzyme are both RLEs.

The patterns of enzyme phylogenetic profiles among species are a good indicator of their metabolic correlation during evolution [6]. To survey the functional links between enzymes in inhibiting pairs with inhibitory relation, phylogenetic profiles were first constructed for each enzyme involved in inhibitory networks. Then Jaccard coefficients were used to depict similarity score between a pair of enzymes with inhibitory relation (Figure 4). The inhibiting pairs whose inhibitor providers are RLEs have higher similarity scores than those of all inhibitory pairs (p value <0.05, unequal t test) except in Rat. Furthermore, the enzymes in inhibiting pairs whose two members are RLEs have higher functional links than those in all the inhibiting pairs where only inhibitor providers are RLEs (p value <0.05, unequal t test) except in Mouse. These results suggest that the inhibitory relations of enzymes in inhibiting pairs are not random, they have some functional correlation.

These global inhibitory relations conserved across several model organisms reflect potential regulatory modules during evolution of these organisms. Based on the number of their occurrence in Human, Mouse, Rat, budding Yeast and *E*. *coli*, 1070 conservative inhibiting enzyme pairs across at least three organisms were identified, of which 410 pairs contain RLEs as inhibitor provider. Our further analysis identified 317 and 234 conservative inhibiting pairs initiated by ADP and AMP respectively, among which 171 ADP and 32 AMP were products of RLEs (Additional file 2). Thus, comparing with the conservative inhibiting pairs initiated by ADP and AMP, RLEs are more likely to produce ADP instead of AMP in inhibitory network (hypergeometric tests, p value <0.05).

## Discussion

We conducted a systematic survey of regulatory roles of RLEs in metabolic inhibitory network in Human, Mouse, Rat, budding Yeast and *E. coli*. Our analysis emphasised their regulatory capacity and their possibility of being regulated in metabolic inhibitory regulation. As over half of *in vivo* inhibitors are produced by RLEs in five surveyed model organisms, RLEs as a whole can initiate inhibiting regulation and spread flux signals to other enzymes quickly. As inhibitor provider, RLEs cover over 85% *in vivo* inhibited targets in five model organisms. Viewed from inhibited targets, RLEs also possess their high possibility of being reached by inhibitors. Forty-five percent of inhibited targets are RLEs, which means an enzyme inhibitor has nearly a fifty percent chance to inhibit RLEs. Our recent study also reveals that the RLEs often locate near to branch points [9]. Therefore, the inhibitory signals received by RLEs are easy to be amplified to different branches of the rest metabolic network in rate-limiting mode.

The cellular metabolic network can be thought as a molecular economic system of supply-and-demand relations between all the participating molecules [16]. One of the most important cellular functions is to carry out the overall metabolic activities to adapt dynamically different environmental or *in vivo* metabolic conditions [17,18]. As enzyme inhibitor is a short-term regulatory style, the cross pathway inhibiting capacity is useful as a feedback to balance metabolic signals between different pathway categories quickly. Our cross-pathway inhibiting analyses confirm the regulatory capacity of RLEs as precise inhibitor providers to maintain the balance of metabolic flux from different pathways, which may form an ideal mechanism to support a quick self-regulatory system.

To investigate the pathway topological evolution, earlier studies have been conducted on relations between phylogenetic profiles of metabolic enzymes and their topological importance including degree, betweenness centrality and closeness centrality [6]. Our present study on phylogenetic profiles of enzymes in inhibiting pairs highlights the evolution of regulatory mechanisms in pathway. Our results suggest that the inhibiting pairs whose inhibitor providers are RLEs have higher similarity scores of phylogenetic profile than those of all common inhibiting pairs except in Rat. Furthermore the inhibiting pairs whose two members are RLEs have higher similarity scores of phylogenetic profile than all the inhibiting pairs where only inhibitor providers are RLEs except in Mouse. Despite the fact that inhibitor data in some organisms are not yet comprehensive, the higher similarities of phylogenetic profiles of RLEs imply their high functional linkages. Especially for conservative inhibiting pairs constituting two RLEs, their highest functional correlation implies that the RLEs possess modularity in metabolic inhibitory regulation network. In summary, our results indicate that the similarities of phylogenetic profiles of metabolic enzymes are not only correlated with topological importance, but also related with their roles in metabolic inhibitory network.

Our comparison between the conservative inhibiting pairs initiated by ADP and AMP indicates that RLEs are more likely to produce ADP instead of AMP in inhibitory network. Previous studies on regulatory role of small compounds in metabolic network revealed that chemical structures of compounds were related with their suitability for use in regulation [19]. And their results show that ATP, ADP and AMP were the top three regulator compounds. Our results confirmed the important regulatory role of ADP in RLE involved inhibitory network. As molecular unit of currency, ATP is the most important energy source for metabolic reactions. And ADP is the most common and quickest product of hydrolysis of ATP in the cell with release of a large amount of free energy. The ratio of ATP to ADP in living cells is the most important metabolic signal in maintaining energy homeostasis. As the RLEs are often related with conversion between ATP to ADP, they are ideal molecules to sense and maintain energy homeostasis.

## Conclusions

In conclusion, our systematic study reveals that over 60% *in vivo* enzyme inhibitors are products of RLEs in Human, Mouse, Rat and Yeast. And the conservative roles of RLEs in efficiency in sensing metabolic signals and transmitting regulatory signals to the rest of the metabolic network reveal that they are ideal regulatory molecules in balancing energy homeostasis via maintaining the ratio of ATP to ADP in living cells. Furthermore, our results indicate that the similarities of phylogenetic profiles of metabolic enzymes are not only correlated with their topological importance, but also related with their roles in enzyme inhibitory network.

## Methods

### Collection of RLEs

230 potential RLEs from Human, Mouse, Rat, budding Yeast (*Saccharomyces cerevisiae*) and *E*. *coli* were compiled from RLE database (RLEdb) [8]. Although some RLEs have species specific metabolic roles, most of RLEs are overlapped in the three mammalian organisms. To do comparative study for the global RLE roles in different organisms, we assumed that all the RLEs could play similar rate-limiting roles in the same pathway in different organisms. If there was a gene to encode a RLE in an organism, the RLE would be assigned to the organism. KEGG pathway annotations were added to each RLE according to KEGG-LIGAND database as described in [9].

### Collection of *in vivo* enzyme inhibitors from BRENDA database and construction of the inhibiting enzyme pairs

*In vivo* enzyme inhibitor data were extracted from the BRENDA database 7.1 [10,11]. And each inhibitor was assigned a KEGG compound identifier. Then the inhibiting enzyme pairs were constructed as described in [9]. We did not transfer the inhibitor annotation between different organisms as the previous study [12,13], so there were different amounts of inhibitors between different organisms. For example, the inhibitors in Mouse were far fewer than those in Human and Rat (Figure 1).

### Construction of enzyme phylogenetic profile

To analyze the functional linkage of two enzymes in an inhibiting pair, we assigned a profile to each enzyme involved in inhibiting network. To construct a reliable phylogenetic profile, 23 prokaryotes (Their genomes were completed before the end of 2000) and 59 eukaryotes (Their annotation data were from Refseq database in KEGG database) were selected. Data from these 82 well studied genomes were included to minimize the bias of in-complete enzyme annotation. Then for each given enzyme, a profile including a string with 82 entries were calculated. If there were one or more enzyme genes in a certain organism, the assigned value for the organism of the enzyme would be one, otherwise it would be assigned zero; finally the functional linkages of enzymes in inhibiting pairs were calculated by Jaccard coefficient.

### Evaluation of the similarity between two profiles using Jaccard coefficient

To assess the similarity between two profiles A and B, we used Jaccard coefficient to score the overlapping values between A and B. Each value of A and B could either be 0 or 1.

The Jaccard similarity coefficient J was calculated as:

J = *M*_11_ ÷ (*M*_01_ + *M*_10_ + *M*_11_ )

Let J be Jaccard coefficient. The total number of each combination of values for both A and B were specified as follows:

M_11_ means the count of values when A and B both were 1.

M_01_ means the count of values when the value of A was 0 and the value of B was 1.

M_10_ means the count of values when the value of A was 1 and the value of B was 0.

### Statistical significant tests

Throughout the paper, we calculated p values based on hypergeometric test and unequal t test in R package 2.6.2 [20].

## Competing interests

The authors declare that they have no competing interests.

## Authors' contributions

MZ conceived of the analysis, carried out analyses and helped write the manuscript. HQ conceived of the analysis and helped write the manuscript.

## Supplementary Material

Additional file 1 **RLEs from literature** The curated 230 RLEs are listed in Additional file 1. For each so-called RLE, we record the original PubMed abstract for reference. Click here for file

Additional file 2 **The conservative inhibitory relations initiated by ADP and AMP produced by RLEs in Human, Mouse, Rat, budding Yeast and *E*. *coli*** The conservative inhibitory relations initiated by ADP (171 pairs) and AMP (32 pairs), products of RLEs, are listed in Additional file 2. Click here for file
