# Supplementary material for: High similarity of phylogenetic profiles of rate-limiting enzymes with inhibitory relation in Human, Mouse, Rat, budding Yeast and E. coli
Source: BMC Genomics. 2011 Nov 30;12(Suppl 3):S10. doi: 10.1186/1471-2164-12-S3-S10 (PMC3333169; doi:10.1186/1471-2164-12-S3-S10)
Supplement: Additional file 1 — RLEs from literature The curated 230 RLEs are listed in Additional file 1. For each so-called RLE, we record the original PubMed abstract for reference. [file 1471-2164-12-S3-S10-S1.pdf]

Additional 1: RLEs from literature

The curated 230 RLEs are listed in Additional file 1. For each so-called RLE, we record the original PubMed abstract for reference.

Enzyme_name	Enzyme_code	Species	Reference/PubMed_id
alcohol dehydrogenase	1.1.1.1	E.coli	1096557
alcohol dehydrogenase	1.1.1.1	E.coli	11303599
alcohol dehydrogenase	1.1.1.1	E.coli	1148277
alcohol dehydrogenase	1.1.1.1	E.coli	12147722
alcohol dehydrogenase	1.1.1.1	E.coli	12489977
alcohol dehydrogenase	1.1.1.1	E.coli	16662798
alcohol dehydrogenase	1.1.1.1	E.coli	167557
alcohol dehydrogenase	1.1.1.1	E.coli	2932116
alcohol dehydrogenase	1.1.1.1	E.coli	3067025
alcohol dehydrogenase	1.1.1.1	E.coli	3893194
alcohol dehydrogenase	1.1.1.1	E.coli	4038269
alcohol dehydrogenase	1.1.1.1	E.coli	6340613
alcohol dehydrogenase	1.1.1.1	E.coli	6341787
alcohol dehydrogenase	1.1.1.1	E.coli	6356161
alcohol dehydrogenase	1.1.1.1	E.coli	6363888
alcohol dehydrogenase	1.1.1.1	E.coli	6370140
alcohol dehydrogenase	1.1.1.1	E.coli	8277258
alcohol dehydrogenase	1.1.1.1	E.coli	8692838
alcohol dehydrogenase	1.1.1.1	E.coli	8905240
alcohol dehydrogenase	1.1.1.1	E.coli	9526508
IMP dehydrogenase	1.1.1.205	E.coli	10194364
IMP dehydrogenase	1.1.1.205	E.coli	10390601
IMP dehydrogenase	1.1.1.205	E.coli	10390603
IMP dehydrogenase	1.1.1.205	E.coli	10391669
IMP dehydrogenase	1.1.1.205	E.coli	10417742
IMP dehydrogenase	1.1.1.205	E.coli	10930578
IMP dehydrogenase	1.1.1.205	E.coli	10953035
IMP dehydrogenase	1.1.1.205	E.coli	10953295
IMP dehydrogenase	1.1.1.205	E.coli	10973868
IMP dehydrogenase	1.1.1.205	E.coli	11003640
IMP dehydrogenase	1.1.1.205	E.coli	1106431
IMP dehydrogenase	1.1.1.205	E.coli	11076502
IMP dehydrogenase	1.1.1.205	E.coli	11145582
IMP dehydrogenase	1.1.1.205	E.coli	11223253
IMP dehydrogenase	1.1.1.205	E.coli	11233304
IMP dehydrogenase	1.1.1.205	E.coli	11288107
IMP dehydrogenase	1.1.1.205	E.coli	11454943
IMP dehydrogenase	1.1.1.205	E.coli	11522119

IMP dehydrogenase	1.1.1.205	E.coli	11566360
IMP dehydrogenase	1.1.1.205	E.coli	11712223
IMP dehydrogenase	1.1.1.205	E.coli	11724288
IMP dehydrogenase	1.1.1.205	E.coli	11875050
IMP dehydrogenase	1.1.1.205	E.coli	11966437
IMP dehydrogenase	1.1.1.205	E.coli	11966440
IMP dehydrogenase	1.1.1.205	E.coli	11966441
IMP dehydrogenase	1.1.1.205	E.coli	12014950
IMP dehydrogenase	1.1.1.205	E.coli	12183689
IMP dehydrogenase	1.1.1.205	E.coli	12213477
IMP dehydrogenase	1.1.1.205	E.coli	12235158
IMP dehydrogenase	1.1.1.205	E.coli	12403633
IMP dehydrogenase	1.1.1.205	E.coli	12559919
IMP dehydrogenase	1.1.1.205	E.coli	12609835
IMP dehydrogenase	1.1.1.205	E.coli	12746440
IMP dehydrogenase	1.1.1.205	E.coli	12773970
IMP dehydrogenase	1.1.1.205	E.coli	12944494
IMP dehydrogenase	1.1.1.205	E.coli	1353938
IMP dehydrogenase	1.1.1.205	E.coli	1356621
IMP dehydrogenase	1.1.1.205	E.coli	14703952
IMP dehydrogenase	1.1.1.205	E.coli	14757177
IMP dehydrogenase	1.1.1.205	E.coli	14766016
IMP dehydrogenase	1.1.1.205	E.coli	14973196
IMP dehydrogenase	1.1.1.205	E.coli	14981049
IMP dehydrogenase	1.1.1.205	E.coli	15043157
IMP dehydrogenase	1.1.1.205	E.coli	15083807
IMP dehydrogenase	1.1.1.205	E.coli	15292516
IMP dehydrogenase	1.1.1.205	E.coli	15355510
IMP dehydrogenase	1.1.1.205	E.coli	15829418
IMP dehydrogenase	1.1.1.205	E.coli	15869715
IMP dehydrogenase	1.1.1.205	E.coli	15882147
IMP dehydrogenase	1.1.1.205	E.coli	15940263
IMP dehydrogenase	1.1.1.205	E.coli	16128570
IMP dehydrogenase	1.1.1.205	E.coli	16243838
IMP dehydrogenase	1.1.1.205	E.coli	16248022
IMP dehydrogenase	1.1.1.205	E.coli	16333815
IMP dehydrogenase	1.1.1.205	E.coli	16647299
IMP dehydrogenase	1.1.1.205	E.coli	16725387
IMP dehydrogenase	1.1.1.205	E.coli	1677309
IMP dehydrogenase	1.1.1.205	E.coli	16936083
IMP dehydrogenase	1.1.1.205	E.coli	17100698
IMP dehydrogenase	1.1.1.205	E.coli	1717828
IMP dehydrogenase	1.1.1.205	E.coli	1723703

IMP dehydrogenase	1.1.1.205	E.coli	1975748
IMP dehydrogenase	1.1.1.205	E.coli	197916
IMP dehydrogenase	1.1.1.205	E.coli	2902093
IMP dehydrogenase	1.1.1.205	E.coli	3314714
IMP dehydrogenase	1.1.1.205	E.coli	4868171
IMP dehydrogenase	1.1.1.205	E.coli	6120758
IMP dehydrogenase	1.1.1.205	E.coli	7476879
IMP dehydrogenase	1.1.1.205	E.coli	7476895
IMP dehydrogenase	1.1.1.205	E.coli	7520100
IMP dehydrogenase	1.1.1.205	E.coli	7874783
IMP dehydrogenase	1.1.1.205	E.coli	7903533
IMP dehydrogenase	1.1.1.205	E.coli	7914720
IMP dehydrogenase	1.1.1.205	E.coli	8103312
IMP dehydrogenase	1.1.1.205	E.coli	8555204
IMP dehydrogenase	1.1.1.205	E.coli	8560580
IMP dehydrogenase	1.1.1.205	E.coli	8830834
IMP dehydrogenase	1.1.1.205	E.coli	8869741
IMP dehydrogenase	1.1.1.205	E.coli	8910338
IMP dehydrogenase	1.1.1.205	E.coli	9042309
IMP dehydrogenase	1.1.1.205	E.coli	9108641
IMP dehydrogenase	1.1.1.205	E.coli	9268334
IMP dehydrogenase	1.1.1.205	E.coli	9278455
IMP dehydrogenase	1.1.1.205	E.coli	9339960
IMP dehydrogenase	1.1.1.205	E.coli	9399601
IMP dehydrogenase	1.1.1.205	E.coli	9413163
IMP dehydrogenase	1.1.1.205	E.coli	9436988
IMP dehydrogenase	1.1.1.205	E.coli	9752721
IMP dehydrogenase	1.1.1.205	E.coli	9766533
IMP dehydrogenase	1.1.1.205	E.coli	9881055
UDP-glucose 6-dehydrogenase	1.1.1.22	E.coli	11044215
UDP-glucose 6-dehydrogenase	1.1.1.22	E.coli	15741737
UDP-glucose 6-dehydrogenase	1.1.1.22	E.coli	2778766
UDP-glucose 6-dehydrogenase	1.1.1.22	E.coli	8471533
3-hydroxyacyl-CoA dehydrogenase	1.1.1.35	E.coli	15358356
3-hydroxyacyl-CoA dehydrogenase	1.1.1.35	E.coli	1637289
3-hydroxyacyl-CoA dehydrogenase	1.1.1.35	E.coli	2388659
3-hydroxyacyl-CoA dehydrogenase	1.1.1.35	E.coli	6588129
malate dehydrogenase	1.1.1.37	E.coli	11583380
malate dehydrogenase	1.1.1.37	E.coli	11767008
malate dehydrogenase	1.1.1.37	E.coli	11855723
malate dehydrogenase	1.1.1.37	E.coli	131232
malate dehydrogenase	1.1.1.37	E.coli	16028114
malate dehydrogenase	1.1.1.37	E.coli	16212411

malate dehydrogenase	1.1.1.37	E.coli	16661455
malate dehydrogenase	1.1.1.37	E.coli	3995045
malate dehydrogenase	1.1.1.37	E.coli	4053567
malate dehydrogenase	1.1.1.37	E.coli	7138874
malate dehydrogenase	1.1.1.37	E.coli	8624506
malate dehydrogenase	1.1.1.37	E.coli	9348107
glucose-6-phosphate dehydrogenase	1.1.1.49	E.coli	10098886
glucose-6-phosphate dehydrogenase	1.1.1.49	E.coli	10099785
glucose-6-phosphate dehydrogenase	1.1.1.49	E.coli	10329961
glucose-6-phosphate dehydrogenase	1.1.1.49	E.coli	10825753
glucose-6-phosphate dehydrogenase	1.1.1.49	E.coli	10998184
glucose-6-phosphate dehydrogenase	1.1.1.49	E.coli	11023706
glucose-6-phosphate dehydrogenase	1.1.1.49	E.coli	11245448
glucose-6-phosphate dehydrogenase	1.1.1.49	E.coli	11463792
glucose-6-phosphate dehydrogenase	1.1.1.49	E.coli	11520909
glucose-6-phosphate dehydrogenase	1.1.1.49	E.coli	12027950
glucose-6-phosphate dehydrogenase	1.1.1.49	E.coli	12204336
glucose-6-phosphate dehydrogenase	1.1.1.49	E.coli	12393032
glucose-6-phosphate dehydrogenase	1.1.1.49	E.coli	12414804
glucose-6-phosphate dehydrogenase	1.1.1.49	E.coli	12453665
glucose-6-phosphate dehydrogenase	1.1.1.49	E.coli	12472120
glucose-6-phosphate dehydrogenase	1.1.1.49	E.coli	12502759
glucose-6-phosphate dehydrogenase	1.1.1.49	E.coli	131232
glucose-6-phosphate dehydrogenase	1.1.1.49	E.coli	1384463
glucose-6-phosphate dehydrogenase	1.1.1.49	E.coli	1417703
glucose-6-phosphate dehydrogenase	1.1.1.49	E.coli	147929
glucose-6-phosphate dehydrogenase	1.1.1.49	E.coli	15331344
glucose-6-phosphate dehydrogenase	1.1.1.49	E.coli	15345489
glucose-6-phosphate dehydrogenase	1.1.1.49	E.coli	15527069
glucose-6-phosphate dehydrogenase	1.1.1.49	E.coli	15550513
glucose-6-phosphate dehydrogenase	1.1.1.49	E.coli	15634201
glucose-6-phosphate dehydrogenase	1.1.1.49	E.coli	15739803
glucose-6-phosphate dehydrogenase	1.1.1.49	E.coli	15760711
glucose-6-phosphate dehydrogenase	1.1.1.49	E.coli	15858258
glucose-6-phosphate dehydrogenase	1.1.1.49	E.coli	15975496
glucose-6-phosphate dehydrogenase	1.1.1.49	E.coli	16039947
glucose-6-phosphate dehydrogenase	1.1.1.49	E.coli	16439706
glucose-6-phosphate dehydrogenase	1.1.1.49	E.coli	16849632
glucose-6-phosphate dehydrogenase	1.1.1.49	E.coli	17157446
glucose-6-phosphate dehydrogenase	1.1.1.49	E.coli	1830744
glucose-6-phosphate dehydrogenase	1.1.1.49	E.coli	1922658
glucose-6-phosphate dehydrogenase	1.1.1.49	E.coli	1978808
glucose-6-phosphate dehydrogenase	1.1.1.49	E.coli	2208076

glucose-6-phosphate dehydrogenase	1.1.1.49	E.coli	2296762
glucose-6-phosphate dehydrogenase	1.1.1.49	E.coli	2767006
glucose-6-phosphate dehydrogenase	1.1.1.49	E.coli	2808772
glucose-6-phosphate dehydrogenase	1.1.1.49	E.coli	2846196
glucose-6-phosphate dehydrogenase	1.1.1.49	E.coli	2984461
glucose-6-phosphate dehydrogenase	1.1.1.49	E.coli	3116361
glucose-6-phosphate dehydrogenase	1.1.1.49	E.coli	3161339
glucose-6-phosphate dehydrogenase	1.1.1.49	E.coli	3316204
glucose-6-phosphate dehydrogenase	1.1.1.49	E.coli	3337882
glucose-6-phosphate dehydrogenase	1.1.1.49	E.coli	3365274
glucose-6-phosphate dehydrogenase	1.1.1.49	E.coli	3532684
glucose-6-phosphate dehydrogenase	1.1.1.49	E.coli	3621197
glucose-6-phosphate dehydrogenase	1.1.1.49	E.coli	3717951
glucose-6-phosphate dehydrogenase	1.1.1.49	E.coli	3765490
glucose-6-phosphate dehydrogenase	1.1.1.49	E.coli	3949801
glucose-6-phosphate dehydrogenase	1.1.1.49	E.coli	4288679
glucose-6-phosphate dehydrogenase	1.1.1.49	E.coli	4382249
glucose-6-phosphate dehydrogenase	1.1.1.49	E.coli	4400642
glucose-6-phosphate dehydrogenase	1.1.1.49	E.coli	4941552
glucose-6-phosphate dehydrogenase	1.1.1.49	E.coli	6295653
glucose-6-phosphate dehydrogenase	1.1.1.49	E.coli	6341787
glucose-6-phosphate dehydrogenase	1.1.1.49	E.coli	6363888
glucose-6-phosphate dehydrogenase	1.1.1.49	E.coli	6420889
glucose-6-phosphate dehydrogenase	1.1.1.49	E.coli	645360
glucose-6-phosphate dehydrogenase	1.1.1.49	E.coli	6591771
glucose-6-phosphate dehydrogenase	1.1.1.49	E.coli	6696439
glucose-6-phosphate dehydrogenase	1.1.1.49	E.coli	7126822
glucose-6-phosphate dehydrogenase	1.1.1.49	E.coli	7578910
glucose-6-phosphate dehydrogenase	1.1.1.49	E.coli	7681896
glucose-6-phosphate dehydrogenase	1.1.1.49	E.coli	7768207
glucose-6-phosphate dehydrogenase	1.1.1.49	E.coli	7930940
glucose-6-phosphate dehydrogenase	1.1.1.49	E.coli	8316633
glucose-6-phosphate dehydrogenase	1.1.1.49	E.coli	864
glucose-6-phosphate dehydrogenase	1.1.1.49	E.coli	8760336
glucose-6-phosphate dehydrogenase	1.1.1.49	E.coli	8797095
glucose-6-phosphate dehydrogenase	1.1.1.49	E.coli	8857518
glucose-6-phosphate dehydrogenase	1.1.1.49	E.coli	8910528
glucose-6-phosphate dehydrogenase	1.1.1.49	E.coli	8954569
glucose-6-phosphate dehydrogenase	1.1.1.49	E.coli	9042391
glucose-6-phosphate dehydrogenase	1.1.1.49	E.coli	9553122
glucose-6-phosphate dehydrogenase	1.1.1.49	E.coli	9581796
glucose-6-phosphate dehydrogenase	1.1.1.49	E.coli	9915806
choline dehydrogenase	1.1.99.1	E.coli	8868068

xanthine dehydrogenase	1.17.1.4	E.coli	11086257
xanthine dehydrogenase	1.17.1.4	E.coli	11154741
xanthine dehydrogenase	1.17.1.4	E.coli	12502743
ribonucleoside-diphosphate reductase	1.17.4.1	E.coli	10441745
ribonucleoside-diphosphate reductase	1.17.4.1	E.coli	10593972
ribonucleoside-diphosphate reductase	1.17.4.1	E.coli	10769119
ribonucleoside-diphosphate reductase	1.17.4.1	E.coli	10805162
ribonucleoside-diphosphate reductase	1.17.4.1	E.coli	10953295
ribonucleoside-diphosphate reductase	1.17.4.1	E.coli	10989193
ribonucleoside-diphosphate reductase	1.17.4.1	E.coli	11489836
ribonucleoside-diphosphate reductase	1.17.4.1	E.coli	11551528
ribonucleoside-diphosphate reductase	1.17.4.1	E.coli	11904430
ribonucleoside-diphosphate reductase	1.17.4.1	E.coli	12147300
ribonucleoside-diphosphate reductase	1.17.4.1	E.coli	12359454
ribonucleoside-diphosphate reductase	1.17.4.1	E.coli	12655059
ribonucleoside-diphosphate reductase	1.17.4.1	E.coli	12690517
ribonucleoside-diphosphate reductase	1.17.4.1	E.coli	12732713
ribonucleoside-diphosphate reductase	1.17.4.1	E.coli	12749906
ribonucleoside-diphosphate reductase	1.17.4.1	E.coli	12967138
ribonucleoside-diphosphate reductase	1.17.4.1	E.coli	1299271
ribonucleoside-diphosphate reductase	1.17.4.1	E.coli	1385411
ribonucleoside-diphosphate reductase	1.17.4.1	E.coli	1412696
ribonucleoside-diphosphate reductase	1.17.4.1	E.coli	14963934
ribonucleoside-diphosphate reductase	1.17.4.1	E.coli	14966112
ribonucleoside-diphosphate reductase	1.17.4.1	E.coli	1496919
ribonucleoside-diphosphate reductase	1.17.4.1	E.coli	15094776
ribonucleoside-diphosphate reductase	1.17.4.1	E.coli	15133626
ribonucleoside-diphosphate reductase	1.17.4.1	E.coli	1516817
ribonucleoside-diphosphate reductase	1.17.4.1	E.coli	15300180
ribonucleoside-diphosphate reductase	1.17.4.1	E.coli	15571292
ribonucleoside-diphosphate reductase	1.17.4.1	E.coli	15656518
ribonucleoside-diphosphate reductase	1.17.4.1	E.coli	15673563
ribonucleoside-diphosphate reductase	1.17.4.1	E.coli	15730856
ribonucleoside-diphosphate reductase	1.17.4.1	E.coli	15769467
ribonucleoside-diphosphate reductase	1.17.4.1	E.coli	15803490
ribonucleoside-diphosphate reductase	1.17.4.1	E.coli	15805194
ribonucleoside-diphosphate reductase	1.17.4.1	E.coli	15888728
ribonucleoside-diphosphate reductase	1.17.4.1	E.coli	160558
ribonucleoside-diphosphate reductase	1.17.4.1	E.coli	16399800
ribonucleoside-diphosphate reductase	1.17.4.1	E.coli	1643157
ribonucleoside-diphosphate reductase	1.17.4.1	E.coli	16489218
ribonucleoside-diphosphate reductase	1.17.4.1	E.coli	16530987
ribonucleoside-diphosphate reductase	1.17.4.1	E.coli	16834759

ribonucleoside-diphosphate reductase	1.17.4.1	E.coli	16925573
ribonucleoside-diphosphate reductase	1.17.4.1	E.coli	17065057
ribonucleoside-diphosphate reductase	1.17.4.1	E.coli	1717630
ribonucleoside-diphosphate reductase	1.17.4.1	E.coli	1748682
ribonucleoside-diphosphate reductase	1.17.4.1	E.coli	1793565
ribonucleoside-diphosphate reductase	1.17.4.1	E.coli	2085432
ribonucleoside-diphosphate reductase	1.17.4.1	E.coli	2178608
ribonucleoside-diphosphate reductase	1.17.4.1	E.coli	2199320
ribonucleoside-diphosphate reductase	1.17.4.1	E.coli	2257322
ribonucleoside-diphosphate reductase	1.17.4.1	E.coli	2642388
ribonucleoside-diphosphate reductase	1.17.4.1	E.coli	2673261
ribonucleoside-diphosphate reductase	1.17.4.1	E.coli	2775821
ribonucleoside-diphosphate reductase	1.17.4.1	E.coli	2827767
ribonucleoside-diphosphate reductase	1.17.4.1	E.coli	2832057
ribonucleoside-diphosphate reductase	1.17.4.1	E.coli	3044371
ribonucleoside-diphosphate reductase	1.17.4.1	E.coli	3044582
ribonucleoside-diphosphate reductase	1.17.4.1	E.coli	3061459
ribonucleoside-diphosphate reductase	1.17.4.1	E.coli	3300645
ribonucleoside-diphosphate reductase	1.17.4.1	E.coli	3511848
ribonucleoside-diphosphate reductase	1.17.4.1	E.coli	3536076
ribonucleoside-diphosphate reductase	1.17.4.1	E.coli	3907637
ribonucleoside-diphosphate reductase	1.17.4.1	E.coli	3914643
ribonucleoside-diphosphate reductase	1.17.4.1	E.coli	3915189
ribonucleoside-diphosphate reductase	1.17.4.1	E.coli	3986794
ribonucleoside-diphosphate reductase	1.17.4.1	E.coli	6353195
ribonucleoside-diphosphate reductase	1.17.4.1	E.coli	6375753
ribonucleoside-diphosphate reductase	1.17.4.1	E.coli	6752137
ribonucleoside-diphosphate reductase	1.17.4.1	E.coli	6757589
ribonucleoside-diphosphate reductase	1.17.4.1	E.coli	767333
ribonucleoside-diphosphate reductase	1.17.4.1	E.coli	7727399
ribonucleoside-diphosphate reductase	1.17.4.1	E.coli	7768988
ribonucleoside-diphosphate reductase	1.17.4.1	E.coli	7838172
ribonucleoside-diphosphate reductase	1.17.4.1	E.coli	7881162
ribonucleoside-diphosphate reductase	1.17.4.1	E.coli	7882331
ribonucleoside-diphosphate reductase	1.17.4.1	E.coli	7893463
ribonucleoside-diphosphate reductase	1.17.4.1	E.coli	7937896
ribonucleoside-diphosphate reductase	1.17.4.1	E.coli	7984431
ribonucleoside-diphosphate reductase	1.17.4.1	E.coli	8241321
ribonucleoside-diphosphate reductase	1.17.4.1	E.coli	8265664
ribonucleoside-diphosphate reductase	1.17.4.1	E.coli	8343143
ribonucleoside-diphosphate reductase	1.17.4.1	E.coli	8463252
ribonucleoside-diphosphate reductase	1.17.4.1	E.coli	8521087
ribonucleoside-diphosphate reductase	1.17.4.1	E.coli	8620054

ribonucleoside-diphosphate reductase	1.17.4.1	E.coli	8662944
ribonucleoside-diphosphate reductase	1.17.4.1	E.coli	8674535
ribonucleoside-diphosphate reductase	1.17.4.1	E.coli	8813126
ribonucleoside-diphosphate reductase	1.17.4.1	E.coli	8878781
ribonucleoside-diphosphate reductase	1.17.4.1	E.coli	8920917
ribonucleoside-diphosphate reductase	1.17.4.1	E.coli	8943056
ribonucleoside-diphosphate reductase	1.17.4.1	E.coli	9192674
ribonucleoside-diphosphate reductase	1.17.4.1	E.coli	9315670
ribonucleoside-diphosphate reductase	1.17.4.1	E.coli	9347313
ribonucleoside-diphosphate reductase	1.17.4.1	E.coli	9354452
ribonucleoside-diphosphate reductase	1.17.4.1	E.coli	9371820
ribonucleoside-diphosphate reductase	1.17.4.1	E.coli	9393942
ribonucleoside-diphosphate reductase	1.17.4.1	E.coli	9415718
ribonucleoside-diphosphate reductase	1.17.4.1	E.coli	9439883
ribonucleoside-diphosphate reductase	1.17.4.1	E.coli	9558318
ribonucleoside-diphosphate reductase	1.17.4.1	E.coli	9570515
ribonucleoside-diphosphate reductase	1.17.4.1	E.coli	9598136
ribonucleoside-diphosphate reductase	1.17.4.1	E.coli	9605773
ribonucleoside-diphosphate reductase	1.17.4.1	E.coli	9634002
ribonucleoside-diphosphate reductase	1.17.4.1	E.coli	9696008
ribonucleoside-diphosphate reductase	1.17.4.1	E.coli	9718080
ribonucleoside-diphosphate reductase	1.17.4.1	E.coli	9852067
ribonucleoside-diphosphate reductase	1.17.4.1	E.coli	9990288
glyceraldehyde-3-phosphate dehydrogenase (phosphorylating)	1.2.1.12	E.coli	10966377
glyceraldehyde-3-phosphate dehydrogenase (phosphorylating)	1.2.1.12	E.coli	11018719
glyceraldehyde-3-phosphate dehydrogenase (phosphorylating)	1.2.1.12	E.coli	12123463
glyceraldehyde-3-phosphate dehydrogenase (phosphorylating)	1.2.1.12	E.coli	12634343
glyceraldehyde-3-phosphate dehydrogenase (phosphorylating)	1.2.1.12	E.coli	14502604
glyceraldehyde-3-phosphate dehydrogenase (phosphorylating)	1.2.1.12	E.coli	15299328
glyceraldehyde-3-phosphate dehydrogenase (phosphorylating)	1.2.1.12	E.coli	15631980
glyceraldehyde-3-phosphate dehydrogenase (phosphorylating)	1.2.1.12	E.coli	15680915
glyceraldehyde-3-phosphate dehydrogenase (phosphorylating)	1.2.1.12	E.coli	3530169
glyceraldehyde-3-phosphate dehydrogenase (phosphorylating)	1.2.1.12	E.coli	6095107

glyceraldehyde-3-phosphate dehydrogenase (phosphorylating)	1.2.1.12	E.coli	6440018
glyceraldehyde-3-phosphate dehydrogenase (phosphorylating)	1.2.1.12	E.coli	664114
glyceraldehyde-3-phosphate dehydrogenase (phosphorylating)	1.2.1.12	E.coli	7165719
glyceraldehyde-3-phosphate dehydrogenase (phosphorylating)	1.2.1.12	E.coli	7173737
glyceraldehyde-3-phosphate dehydrogenase (phosphorylating)	1.2.1.12	E.coli	8304415
glyceraldehyde-3-phosphate dehydrogenase (phosphorylating)	1.2.1.12	E.coli	8454610
glyceraldehyde-3-phosphate dehydrogenase (phosphorylating)	1.2.1.12	E.coli	8791005
glyceraldehyde-3-phosphate dehydrogenase (phosphorylating)	1.2.1.12	E.coli	8805794
glyceraldehyde-3-phosphate dehydrogenase (phosphorylating)	1.2.1.12	E.coli	8905295
glyceraldehyde-3-phosphate dehydrogenase (phosphorylating)	1.2.1.12	E.coli	9437188
glyceraldehyde-3-phosphate dehydrogenase (phosphorylating)	1.2.1.12	E.coli	9793073
glyceraldehyde-3-phosphate dehydrogenase (phosphorylating)	1.2.1.12	E.coli	9922941
N-acetyl-gamma-glutamyl-phosphate reductase	1.2.1.38	E.coli	14602584
glutamyl-tRNA reductase	1.2.1.70	E.coli	11309145
glutamyl-tRNA reductase	1.2.1.70	E.coli	15584960
glutamyl-tRNA reductase	1.2.1.70	E.coli	16122943
glutamyl-tRNA reductase	1.2.1.70	E.coli	16158240
glutamyl-tRNA reductase	1.2.1.70	E.coli	7942284
pyruvate dehydrogenase (acetyl-transferring)	1.2.4.1	E.coli	10720420
pyruvate dehydrogenase (acetyl-transferring)	1.2.4.1	E.coli	10759582
pyruvate dehydrogenase (acetyl-transferring)	1.2.4.1	E.coli	10826498
pyruvate dehydrogenase (acetyl-transferring)	1.2.4.1	E.coli	11223534
pyruvate dehydrogenase (acetyl-transferring)	1.2.4.1	E.coli	11427685
pyruvate dehydrogenase (acetyl-transferring)	1.2.4.1	E.coli	11557577
pyruvate dehydrogenase (acetyl-transferring)	1.2.4.1	E.coli	11866475
pyruvate dehydrogenase (acetyl-transferring)	1.2.4.1	E.coli	11900279
pyruvate dehydrogenase (acetyl-transferring)	1.2.4.1	E.coli	12196468
pyruvate dehydrogenase (acetyl-transferring)	1.2.4.1	E.coli	12557749
pyruvate dehydrogenase (acetyl-transferring)	1.2.4.1	E.coli	12663261
pyruvate dehydrogenase (acetyl-transferring)	1.2.4.1	E.coli	14607783

pyruvate dehydrogenase (acetyl-transferring)	1.2.4.1	E.coli	14641018
pyruvate dehydrogenase (acetyl-transferring)	1.2.4.1	E.coli	15191896
pyruvate dehydrogenase (acetyl-transferring)	1.2.4.1	E.coli	15256563
pyruvate dehydrogenase (acetyl-transferring)	1.2.4.1	E.coli	15921824
pyruvate dehydrogenase (acetyl-transferring)	1.2.4.1	E.coli	1600837
pyruvate dehydrogenase (acetyl-transferring)	1.2.4.1	E.coli	17065338
pyruvate dehydrogenase (acetyl-transferring)	1.2.4.1	E.coli	2902801
pyruvate dehydrogenase (acetyl-transferring)	1.2.4.1	E.coli	3127087
pyruvate dehydrogenase (acetyl-transferring)	1.2.4.1	E.coli	3918587
pyruvate dehydrogenase (acetyl-transferring)	1.2.4.1	E.coli	4030556
pyruvate dehydrogenase (acetyl-transferring)	1.2.4.1	E.coli	6149743
pyruvate dehydrogenase (acetyl-transferring)	1.2.4.1	E.coli	6234885
pyruvate dehydrogenase (acetyl-transferring)	1.2.4.1	E.coli	7127258
pyruvate dehydrogenase (acetyl-transferring)	1.2.4.1	E.coli	7848280
pyruvate dehydrogenase (acetyl-transferring)	1.2.4.1	E.coli	7864215
pyruvate dehydrogenase (acetyl-transferring)	1.2.4.1	E.coli	8557765
pyruvate dehydrogenase (acetyl-transferring)	1.2.4.1	E.coli	9089387
pyruvate dehydrogenase (acetyl-transferring)	1.2.4.1	E.coli	9381974
pyruvate dehydrogenase (acetyl-transferring)	1.2.4.1	E.coli	9426381
pyruvate dehydrogenase (acetyl-transferring)	1.2.4.1	E.coli	9438382
pyruvate dehydrogenase (acetyl-transferring)	1.2.4.1	E.coli	9497164
pyruvate dehydrogenase (acetyl-transferring)	1.2.4.1	E.coli	9725804
oxoglutarate dehydrogenase (succinyl-transferring)	1.2.4.2	E.coli	1352447
oxoglutarate dehydrogenase (succinyl-transferring)	1.2.4.2	E.coli	15356188
oxoglutarate dehydrogenase (succinyl-transferring)	1.2.4.2	E.coli	15466852
oxoglutarate dehydrogenase (succinyl-transferring)	1.2.4.2	E.coli	423807
oxoglutarate dehydrogenase (succinyl-transferring)	1.2.4.2	E.coli	6778477
oxoglutarate dehydrogenase (succinyl-transferring)	1.2.4.2	E.coli	7995856
enoyl-[acyl-carrier-protein] reductase (NADH)	1.3.1.9	E.coli	17095231
enoyl-[acyl-carrier-protein] reductase (NADH)	1.3.1.9	E.coli	8075395
dihydroorotate oxidase	1.3.3.1	E.coli	10348793
dihydroorotate oxidase	1.3.3.1	E.coli	10473079
dihydroorotate oxidase	1.3.3.1	E.coli	10848981
dihydroorotate oxidase	1.3.3.1	E.coli	10853015
dihydroorotate oxidase	1.3.3.1	E.coli	10878295

dihydroorotate oxidase	1.3.3.1	E.coli	10887632
dihydroorotate oxidase	1.3.3.1	E.coli	11053058
dihydroorotate oxidase	1.3.3.1	E.coli	11060767
dihydroorotate oxidase	1.3.3.1	E.coli	11098485
dihydroorotate oxidase	1.3.3.1	E.coli	11219978
dihydroorotate oxidase	1.3.3.1	E.coli	11267945
dihydroorotate oxidase	1.3.3.1	E.coli	11334264
dihydroorotate oxidase	1.3.3.1	E.coli	11376565
dihydroorotate oxidase	1.3.3.1	E.coli	11522581
dihydroorotate oxidase	1.3.3.1	E.coli	11555601
dihydroorotate oxidase	1.3.3.1	E.coli	11697836
dihydroorotate oxidase	1.3.3.1	E.coli	11796730
dihydroorotate oxidase	1.3.3.1	E.coli	11862480
dihydroorotate oxidase	1.3.3.1	E.coli	11865631
dihydroorotate oxidase	1.3.3.1	E.coli	11936689
dihydroorotate oxidase	1.3.3.1	E.coli	11962674
dihydroorotate oxidase	1.3.3.1	E.coli	12084458
dihydroorotate oxidase	1.3.3.1	E.coli	12187768
dihydroorotate oxidase	1.3.3.1	E.coli	12469154
dihydroorotate oxidase	1.3.3.1	E.coli	12527935
dihydroorotate oxidase	1.3.3.1	E.coli	12634225
dihydroorotate oxidase	1.3.3.1	E.coli	12820455
dihydroorotate oxidase	1.3.3.1	E.coli	12885330
dihydroorotate oxidase	1.3.3.1	E.coli	12967482
dihydroorotate oxidase	1.3.3.1	E.coli	14689231
dihydroorotate oxidase	1.3.3.1	E.coli	14744810
dihydroorotate oxidase	1.3.3.1	E.coli	15025949
dihydroorotate oxidase	1.3.3.1	E.coli	15045945
dihydroorotate oxidase	1.3.3.1	E.coli	15069534
dihydroorotate oxidase	1.3.3.1	E.coli	15134221
dihydroorotate oxidase	1.3.3.1	E.coli	15222105
dihydroorotate oxidase	1.3.3.1	E.coli	15450176
dihydroorotate oxidase	1.3.3.1	E.coli	15944938
dihydroorotate oxidase	1.3.3.1	E.coli	16163233
dihydroorotate oxidase	1.3.3.1	E.coli	1629785
dihydroorotate oxidase	1.3.3.1	E.coli	16556484
dihydroorotate oxidase	1.3.3.1	E.coli	17046731
dihydroorotate oxidase	1.3.3.1	E.coli	2060083
dihydroorotate oxidase	1.3.3.1	E.coli	3202908
dihydroorotate oxidase	1.3.3.1	E.coli	6761622
dihydroorotate oxidase	1.3.3.1	E.coli	7602356
dihydroorotate oxidase	1.3.3.1	E.coli	7825962
dihydroorotate oxidase	1.3.3.1	E.coli	8093030

dihydroorotate oxidase	1.3.3.1	E.coli	8097697
dihydroorotate oxidase	1.3.3.1	E.coli	8878781
dihydroorotate oxidase	1.3.3.1	E.coli	8895681
dihydroorotate oxidase	1.3.3.1	E.coli	8950202
dihydroorotate oxidase	1.3.3.1	E.coli	9056186
dihydroorotate oxidase	1.3.3.1	E.coli	9182832
dihydroorotate oxidase	1.3.3.1	E.coli	9280881
dihydroorotate oxidase	1.3.3.1	E.coli	9464498
dihydroorotate oxidase	1.3.3.1	E.coli	9597696
dihydroorotate oxidase	1.3.3.1	E.coli	9636062
dihydroorotate oxidase	1.3.3.1	E.coli	9819714
dihydroorotate oxidase	1.3.3.1	E.coli	9860876
dihydroorotate oxidase	1.3.3.1	E.coli	9914783
coproporphyrinogen oxidase	1.3.3.3	E.coli	10787385
coproporphyrinogen oxidase	1.3.3.3	E.coli	11368326
coproporphyrinogen oxidase	1.3.3.3	E.coli	16567402
glutamate dehydrogenase (NADP+)	1.4.1.4	E.coli	10323225
glutamate dehydrogenase (NADP+)	1.4.1.4	E.coli	1576153
glutamate dehydrogenase (NADP+)	1.4.1.4	E.coli	3139028
glutamate dehydrogenase (NADP+)	1.4.1.4	E.coli	6116360
glutamate dehydrogenase (NADP+)	1.4.1.4	E.coli	7470041
glutamate dehydrogenase (NADP+)	1.4.1.4	E.coli	7765894
glutamate dehydrogenase (NADP+)	1.4.1.4	E.coli	8122033
glutamate dehydrogenase (NADP+)	1.4.1.4	E.coli	9918511
pyridoxal 5'-phosphate synthase	1.4.3.5	E.coli	6822512
pyridoxal 5'-phosphate synthase	1.4.3.5	E.coli	9601034
1-pyrroline-5-carboxylate dehydrogenase	1.5.1.12	E.coli	10363370
1-pyrroline-5-carboxylate dehydrogenase	1.5.1.12	E.coli	10398729
1-pyrroline-5-carboxylate dehydrogenase	1.5.1.12	E.coli	10441499
1-pyrroline-5-carboxylate dehydrogenase	1.5.1.12	E.coli	10759508
1-pyrroline-5-carboxylate dehydrogenase	1.5.1.12	E.coli	10945345
1-pyrroline-5-carboxylate dehydrogenase	1.5.1.12	E.coli	11726714
1-pyrroline-5-carboxylate dehydrogenase	1.5.1.12	E.coli	12602867
1-pyrroline-5-carboxylate dehydrogenase	1.5.1.12	E.coli	14602584
1-pyrroline-5-carboxylate dehydrogenase	1.5.1.12	E.coli	15077666
1-pyrroline-5-carboxylate dehydrogenase	1.5.1.12	E.coli	2860838
1-pyrroline-5-carboxylate dehydrogenase	1.5.1.12	E.coli	9351242
methylenetetrahydrofolate reductase [NAD(P)H]	1.5.1.20	E.coli	10462593
methylenetetrahydrofolate reductase [NAD(P)H]	1.5.1.20	E.coli	10948708
methylenetetrahydrofolate reductase [NAD(P)H]	1.5.1.20	E.coli	11302003

methylenetetrahydrofolate reductase [NAD(P)H]	1.5.1.20	E.coli	11343335
methylenetetrahydrofolate reductase [NAD(P)H]	1.5.1.20	E.coli	11398138
methylenetetrahydrofolate reductase [NAD(P)H]	1.5.1.20	E.coli	11680544
methylenetetrahydrofolate reductase [NAD(P)H]	1.5.1.20	E.coli	11712321
methylenetetrahydrofolate reductase [NAD(P)H]	1.5.1.20	E.coli	11927833
methylenetetrahydrofolate reductase [NAD(P)H]	1.5.1.20	E.coli	12028998
methylenetetrahydrofolate reductase [NAD(P)H]	1.5.1.20	E.coli	12038037
methylenetetrahydrofolate reductase [NAD(P)H]	1.5.1.20	E.coli	12600862
methylenetetrahydrofolate reductase [NAD(P)H]	1.5.1.20	E.coli	12897091
methylenetetrahydrofolate reductase [NAD(P)H]	1.5.1.20	E.coli	12914571
methylenetetrahydrofolate reductase [NAD(P)H]	1.5.1.20	E.coli	14608052
methylenetetrahydrofolate reductase [NAD(P)H]	1.5.1.20	E.coli	14728017
methylenetetrahydrofolate reductase [NAD(P)H]	1.5.1.20	E.coli	14973104
methylenetetrahydrofolate reductase [NAD(P)H]	1.5.1.20	E.coli	15033905
methylenetetrahydrofolate reductase [NAD(P)H]	1.5.1.20	E.coli	15207432
methylenetetrahydrofolate reductase [NAD(P)H]	1.5.1.20	E.coli	15449187
methylenetetrahydrofolate reductase [NAD(P)H]	1.5.1.20	E.coli	15546509
methylenetetrahydrofolate reductase [NAD(P)H]	1.5.1.20	E.coli	15581487
methylenetetrahydrofolate reductase [NAD(P)H]	1.5.1.20	E.coli	15598763
methylenetetrahydrofolate reductase [NAD(P)H]	1.5.1.20	E.coli	15688606
methylenetetrahydrofolate reductase [NAD(P)H]	1.5.1.20	E.coli	15773669

methylenetetrahydrofolate reductase [NAD(P)H]	1.5.1.20	E.coli	15894672
methylenetetrahydrofolate reductase [NAD(P)H]	1.5.1.20	E.coli	15935452
methylenetetrahydrofolate reductase [NAD(P)H]	1.5.1.20	E.coli	16055944
methylenetetrahydrofolate reductase [NAD(P)H]	1.5.1.20	E.coli	16108833
methylenetetrahydrofolate reductase [NAD(P)H]	1.5.1.20	E.coli	16128738
methylenetetrahydrofolate reductase [NAD(P)H]	1.5.1.20	E.coli	16274753
methylenetetrahydrofolate reductase [NAD(P)H]	1.5.1.20	E.coli	16538645
methylenetetrahydrofolate reductase [NAD(P)H]	1.5.1.20	E.coli	16706930
methylenetetrahydrofolate reductase [NAD(P)H]	1.5.1.20	E.coli	17105984
methylenetetrahydrofolate reductase [NAD(P)H]	1.5.1.20	E.coli	3143307
methylenetetrahydrofolate reductase [NAD(P)H]	1.5.1.20	E.coli	6352699
methylenetetrahydrofolate reductase [NAD(P)H]	1.5.1.20	E.coli	6391540
methylenetetrahydrofolate reductase [NAD(P)H]	1.5.1.20	E.coli	9789068
6,7-dihydropteridine reductase	1.5.1.34	E.coli	2913929
6,7-dihydropteridine reductase	1.5.1.34	E.coli	6797415
proline dehydrogenase	1.5.99.8	E.coli	11788754
proline dehydrogenase	1.5.99.8	E.coli	12602867
dihydrolipoyl dehydrogenase	1.8.1.4	E.coli	10885793
dihydrolipoyl dehydrogenase	1.8.1.4	E.coli	11641455
dihydrolipoyl dehydrogenase	1.8.1.4	E.coli	12812918
dihydrolipoyl dehydrogenase	1.8.1.4	E.coli	15173434
dihydrolipoyl dehydrogenase	1.8.1.4	E.coli	15915669
dihydrolipoyl dehydrogenase	1.8.1.4	E.coli	16581023
dihydrolipoyl dehydrogenase	1.8.1.4	E.coli	16616211
dihydrolipoyl dehydrogenase	1.8.1.4	E.coli	16875466
dihydrolipoyl dehydrogenase	1.8.1.4	E.coli	2381301
dihydrolipoyl dehydrogenase	1.8.1.4	E.coli	2404025
dihydrolipoyl dehydrogenase	1.8.1.4	E.coli	3103467
dihydrolipoyl dehydrogenase	1.8.1.4	E.coli	3425711
dihydrolipoyl dehydrogenase	1.8.1.4	E.coli	7782942

glutathione-disulfide reductase	1.8.1.7	E.coli	11133045
glutathione-disulfide reductase	1.8.1.7	E.coli	12204336
glutathione-disulfide reductase	1.8.1.7	E.coli	12453665
glutathione-disulfide reductase	1.8.1.7	E.coli	1605642
glutathione-disulfide reductase	1.8.1.7	E.coli	2848577
glutathione-disulfide reductase	1.8.1.7	E.coli	3698652
glutathione-disulfide reductase	1.8.1.7	E.coli	3963383
glutathione-disulfide reductase	1.8.1.7	E.coli	8843715
glutathione-disulfide reductase	1.8.1.7	E.coli	9350472
thymidylate synthase	2.1.1.45	E.coli	11358693
thymidylate synthase	2.1.1.45	E.coli	12412165
thymidylate synthase	2.1.1.45	E.coli	12457437
thymidylate synthase	2.1.1.45	E.coli	12470718
thymidylate synthase	2.1.1.45	E.coli	12544347
thymidylate synthase	2.1.1.45	E.coli	14578129
thymidylate synthase	2.1.1.45	E.coli	14648018
thymidylate synthase	2.1.1.45	E.coli	14689231
thymidylate synthase	2.1.1.45	E.coli	15025949
thymidylate synthase	2.1.1.45	E.coli	15134221
thymidylate synthase	2.1.1.45	E.coli	15598787
thymidylate synthase	2.1.1.45	E.coli	16077970
thymidylate synthase	2.1.1.45	E.coli	16540728
thymidylate synthase	2.1.1.45	E.coli	16617381
thymidylate synthase	2.1.1.45	E.coli	3709927
thymidylate synthase	2.1.1.45	E.coli	7602356
thymidylate synthase	2.1.1.45	E.coli	8805515
phosphoribosylaminoimidazolecarboxamide formyltransferase	2.1.2.3	E.coli	6335666
aspartate carbamoyltransferase	2.1.3.2	E.coli	10336386
aspartate carbamoyltransferase	2.1.3.2	E.coli	10593256
aspartate carbamoyltransferase	2.1.3.2	E.coli	11397099
aspartate carbamoyltransferase	2.1.3.2	E.coli	15165857
aspartate carbamoyltransferase	2.1.3.2	E.coli	15529744
aspartate carbamoyltransferase	2.1.3.2	E.coli	16120448
aspartate carbamoyltransferase	2.1.3.2	E.coli	17008138
aspartate carbamoyltransferase	2.1.3.2	E.coli	3047117
aspartate carbamoyltransferase	2.1.3.2	E.coli	3542019
aspartate carbamoyltransferase	2.1.3.2	E.coli	3907993
aspartate carbamoyltransferase	2.1.3.2	E.coli	4092695
aspartate carbamoyltransferase	2.1.3.2	E.coli	6115855
aspartate carbamoyltransferase	2.1.3.2	E.coli	6298785
aspartate carbamoyltransferase	2.1.3.2	E.coli	7051000
aspartate carbamoyltransferase	2.1.3.2	E.coli	9611817

aspartate carbamoyltransferase	2.1.3.2	E.coli	9626698
aspartate carbamoyltransferase	2.1.3.2	E.coli	9659392
ornithine carbamoyltransferase	2.1.3.3	E.coli	1505922
ornithine carbamoyltransferase	2.1.3.3	E.coli	205703
ornithine carbamoyltransferase	2.1.3.3	E.coli	2667139
ornithine carbamoyltransferase	2.1.3.3	E.coli	2667140
ornithine carbamoyltransferase	2.1.3.3	E.coli	2667141
ornithine carbamoyltransferase	2.1.3.3	E.coli	8019156
ornithine carbamoyltransferase	2.1.3.3	E.coli	8168544
ornithine carbamoyltransferase	2.1.3.3	E.coli	9501170
ornithine carbamoyltransferase	2.1.3.3	E.coli	9540805
transketolase	2.2.1.1	E.coli	10975072
transketolase	2.2.1.1	E.coli	11072071
transketolase	2.2.1.1	E.coli	16354724
transketolase	2.2.1.1	E.coli	1939098
transketolase	2.2.1.1	E.coli	9924800
transaldolase	2.2.1.2	E.coli	11390181
transaldolase	2.2.1.2	E.coli	12359249
transaldolase	2.2.1.2	E.coli	15263091
transaldolase	2.2.1.2	E.coli	16092052
transaldolase	2.2.1.2	E.coli	17046540
transaldolase	2.2.1.2	E.coli	9565623
transaldolase	2.2.1.2	E.coli	9973403
1-deoxy-D-xylulose-5-phosphate synthase	2.2.1.7	E.coli	10886770
1-deoxy-D-xylulose-5-phosphate synthase	2.2.1.7	E.coli	11532167
1-deoxy-D-xylulose-5-phosphate synthase	2.2.1.7	E.coli	12957384
1-deoxy-D-xylulose-5-phosphate synthase	2.2.1.7	E.coli	15898075
1-deoxy-D-xylulose-5-phosphate synthase	2.2.1.7	E.coli	16941216
dihydrolipoyllysine-residue acetyltransferase	2.3.1.12	E.coli	3840997
glycerol-3-phosphate O-acyltransferase	2.3.1.15	E.coli	11003606
glycerol-3-phosphate O-acyltransferase	2.3.1.15	E.coli	11284717
glycerol-3-phosphate O-acyltransferase	2.3.1.15	E.coli	12464581
glycerol-3-phosphate O-acyltransferase	2.3.1.15	E.coli	12730219
glycerol-3-phosphate O-acyltransferase	2.3.1.15	E.coli	14724270
glycerol-3-phosphate O-acyltransferase	2.3.1.15	E.coli	15102885
glycerol-3-phosphate O-acyltransferase	2.3.1.15	E.coli	16234267
glycerol-3-phosphate O-acyltransferase	2.3.1.15	E.coli	16431156
glycerol-3-phosphate O-acyltransferase	2.3.1.15	E.coli	16507761
glycerol-3-phosphate O-acyltransferase	2.3.1.15	E.coli	2303421
glycerol-3-phosphate O-acyltransferase	2.3.1.15	E.coli	2559137
glycerol-3-phosphate O-acyltransferase	2.3.1.15	E.coli	2674663
glycerol-3-phosphate O-acyltransferase	2.3.1.15	E.coli	2737209
glycerol-3-phosphate O-acyltransferase	2.3.1.15	E.coli	2830105

glycerol-3-phosphate O-acyltransferase	2.3.1.15	E.coli	3103689
glycerol-3-phosphate O-acyltransferase	2.3.1.15	E.coli	3311005
glycerol-3-phosphate O-acyltransferase	2.3.1.15	E.coli	3334861
glycerol-3-phosphate O-acyltransferase	2.3.1.15	E.coli	6497849
glycerol-3-phosphate O-acyltransferase	2.3.1.15	E.coli	813635
glycerol-3-phosphate O-acyltransferase	2.3.1.15	E.coli	8387510
glycerol-3-phosphate O-acyltransferase	2.3.1.15	E.coli	9393688
acetyl-CoA C-acyltransferase	2.3.1.16	E.coli	3967008
acetyl-CoA C-acyltransferase	2.3.1.16	E.coli	7068598
acetyl-CoA C-acyltransferase	2.3.1.16	E.coli	7374368
serine O-acetyltransferase	2.3.1.30	E.coli	11106665
serine O-acetyltransferase	2.3.1.30	E.coli	11168407
serine O-acetyltransferase	2.3.1.30	E.coli	1885524
serine O-acetyltransferase	2.3.1.30	E.coli	7851429
serine O-acetyltransferase	2.3.1.30	E.coli	9161412
serine O-acetyltransferase	2.3.1.30	E.coli	9622597
diamine N-acetyltransferase	2.3.1.57	E.coli	10101027
diamine N-acetyltransferase	2.3.1.57	E.coli	10430062
diamine N-acetyltransferase	2.3.1.57	E.coli	11166157
diamine N-acetyltransferase	2.3.1.57	E.coli	11298808
diamine N-acetyltransferase	2.3.1.57	E.coli	11779193
diamine N-acetyltransferase	2.3.1.57	E.coli	12798351
diamine N-acetyltransferase	2.3.1.57	E.coli	12827295
diamine N-acetyltransferase	2.3.1.57	E.coli	1360468
diamine N-acetyltransferase	2.3.1.57	E.coli	1420355
diamine N-acetyltransferase	2.3.1.57	E.coli	15223770
diamine N-acetyltransferase	2.3.1.57	E.coli	15737201
diamine N-acetyltransferase	2.3.1.57	E.coli	1581359
diamine N-acetyltransferase	2.3.1.57	E.coli	1590311
diamine N-acetyltransferase	2.3.1.57	E.coli	16207710
diamine N-acetyltransferase	2.3.1.57	E.coli	16262603
diamine N-acetyltransferase	2.3.1.57	E.coli	16400014
diamine N-acetyltransferase	2.3.1.57	E.coli	16455797
diamine N-acetyltransferase	2.3.1.57	E.coli	2463788
diamine N-acetyltransferase	2.3.1.57	E.coli	2987201
diamine N-acetyltransferase	2.3.1.57	E.coli	3435554
diamine N-acetyltransferase	2.3.1.57	E.coli	3443136
diamine N-acetyltransferase	2.3.1.57	E.coli	3928404
diamine N-acetyltransferase	2.3.1.57	E.coli	4093447
diamine N-acetyltransferase	2.3.1.57	E.coli	6510524
diamine N-acetyltransferase	2.3.1.57	E.coli	6654645
diamine N-acetyltransferase	2.3.1.57	E.coli	7141002
diamine N-acetyltransferase	2.3.1.57	E.coli	7803498

diamine N-acetyltransferase	2.3.1.57	E.coli	8119700
diamine N-acetyltransferase	2.3.1.57	E.coli	8427870
diamine N-acetyltransferase	2.3.1.57	E.coli	8500690
diamine N-acetyltransferase	2.3.1.57	E.coli	8549747
diamine N-acetyltransferase	2.3.1.57	E.coli	8573111
diamine N-acetyltransferase	2.3.1.57	E.coli	8737675
diamine N-acetyltransferase	2.3.1.57	E.coli	8814137
diamine N-acetyltransferase	2.3.1.57	E.coli	8876622
diamine N-acetyltransferase	2.3.1.57	E.coli	8954982
diamine N-acetyltransferase	2.3.1.57	E.coli	9063811
diamine N-acetyltransferase	2.3.1.57	E.coli	9115288
diamine N-acetyltransferase	2.3.1.57	E.coli	9585063
diamine N-acetyltransferase	2.3.1.57	E.coli	9620361
diamine N-acetyltransferase	2.3.1.57	E.coli	9780334
acetyl-CoA C-acetyltransferase	2.3.1.9	E.coli	14693556
acetyl-CoA C-acetyltransferase	2.3.1.9	E.coli	15135409
acetyl-CoA C-acetyltransferase	2.3.1.9	E.coli	15466479
acetyl-CoA C-acetyltransferase	2.3.1.9	E.coli	2869784
gamma-glutamyltransferase	2.3.2.2	E.coli	11810401
gamma-glutamyltransferase	2.3.2.2	E.coli	12030366
gamma-glutamyltransferase	2.3.2.2	E.coli	12468440
gamma-glutamyltransferase	2.3.2.2	E.coli	12780970
gamma-glutamyltransferase	2.3.2.2	E.coli	15006645
gamma-glutamyltransferase	2.3.2.2	E.coli	16302185
gamma-glutamyltransferase	2.3.2.2	E.coli	2881890
gamma-glutamyltransferase	2.3.2.2	E.coli	2903803
gamma-glutamyltransferase	2.3.2.2	E.coli	7485380
gamma-glutamyltransferase	2.3.2.2	E.coli	8067452
gamma-glutamyltransferase	2.3.2.2	E.coli	8564390
gamma-glutamyltransferase	2.3.2.2	E.coli	8972486
gamma-glutamyltransferase	2.3.2.2	E.coli	9974125
citrate (Si)-synthase	2.3.3.1	E.coli	1004246
citrate (Si)-synthase	2.3.3.1	E.coli	11842094
citrate (Si)-synthase	2.3.3.1	E.coli	11872452
citrate (Si)-synthase	2.3.3.1	E.coli	15994367
citrate (Si)-synthase	2.3.3.1	E.coli	16269721
citrate (Si)-synthase	2.3.3.1	E.coli	3776117
citrate (Si)-synthase	2.3.3.1	E.coli	3916224
citrate (Si)-synthase	2.3.3.1	E.coli	6799496
citrate (Si)-synthase	2.3.3.1	E.coli	8526514
citrate (Si)-synthase	2.3.3.1	E.coli	9353808
citrate (Si)-synthase	2.3.3.1	E.coli	9554114
phosphorylase	2.4.1.1	E.coli	10548038

phosphorylase	2.4.1.1	E.coli	11391834
phosphorylase	2.4.1.1	E.coli	11391835
phosphorylase	2.4.1.1	E.coli	11391836
phosphorylase	2.4.1.1	E.coli	11391837
phosphorylase	2.4.1.1	E.coli	11391838
phosphorylase	2.4.1.1	E.coli	11391839
phosphorylase	2.4.1.1	E.coli	11391840
phosphorylase	2.4.1.1	E.coli	11391841
phosphorylase	2.4.1.1	E.coli	11391842
phosphorylase	2.4.1.1	E.coli	12769745
phosphorylase	2.4.1.1	E.coli	15299833
phosphorylase	2.4.1.1	E.coli	15721288
phosphorylase	2.4.1.1	E.coli	1691273
phosphorylase	2.4.1.1	E.coli	7664039
nicotinate phosphoribosyltransferase	2.4.2.11	E.coli	10825532
amidophosphoribosyltransferase	2.4.2.14	E.coli	10675983
amidophosphoribosyltransferase	2.4.2.14	E.coli	11158364
amidophosphoribosyltransferase	2.4.2.14	E.coli	12930749
amidophosphoribosyltransferase	2.4.2.14	E.coli	15266056
amidophosphoribosyltransferase	2.4.2.14	E.coli	17434429
amidophosphoribosyltransferase	2.4.2.14	E.coli	214373
amidophosphoribosyltransferase	2.4.2.14	E.coli	6327016
amidophosphoribosyltransferase	2.4.2.14	E.coli	701284
amidophosphoribosyltransferase	2.4.2.14	E.coli	7683680
amidophosphoribosyltransferase	2.4.2.14	E.coli	8150282
amidophosphoribosyltransferase	2.4.2.14	E.coli	8197456
amidophosphoribosyltransferase	2.4.2.14	E.coli	8197457
amidophosphoribosyltransferase	2.4.2.14	E.coli	8197458
amidophosphoribosyltransferase	2.4.2.14	E.coli	8380692
amidophosphoribosyltransferase	2.4.2.14	E.coli	8463258
amidophosphoribosyltransferase	2.4.2.14	E.coli	8809759
amidophosphoribosyltransferase	2.4.2.14	E.coli	8976092
amidophosphoribosyltransferase	2.4.2.14	E.coli	9615746
amidophosphoribosyltransferase	2.4.2.14	E.coli	9881055
thymidine phosphorylase	2.4.2.4	E.coli	10853015
thymidine phosphorylase	2.4.2.4	E.coli	11530879
thymidine phosphorylase	2.4.2.4	E.coli	11585972
thymidine phosphorylase	2.4.2.4	E.coli	12680231
thymidine phosphorylase	2.4.2.4	E.coli	15134221
thymidine phosphorylase	2.4.2.4	E.coli	15289834
thymidine phosphorylase	2.4.2.4	E.coli	15917420
thymidine phosphorylase	2.4.2.4	E.coli	16302736
adenine phosphoribosyltransferase	2.4.2.7	E.coli	2154328

adenine phosphoribosyltransferase	2.4.2.7	E.coli	6327016
geranyltranstransferase	2.5.1.10	E.coli	10484604
geranyltranstransferase	2.5.1.10	E.coli	11202437
geranyltranstransferase	2.5.1.10	E.coli	15459425
geranyltranstransferase	2.5.1.10	E.coli	15605175
geranyltranstransferase	2.5.1.10	E.coli	15713990
geranyltranstransferase	2.5.1.10	E.coli	15827605
geranyltranstransferase	2.5.1.10	E.coli	15827618
geranyltranstransferase	2.5.1.10	E.coli	16179378
geranyltranstransferase	2.5.1.10	E.coli	16932286
geranyltranstransferase	2.5.1.10	E.coli	1779710
geranyltranstransferase	2.5.1.10	E.coli	9061016
geranyltranstransferase	2.5.1.10	E.coli	9640665
spermidine synthase	2.5.1.16	E.coli	16515550
spermidine synthase	2.5.1.16	E.coli	2775206
glutathione transferase	2.5.1.18	E.coli	12484753
glutathione transferase	2.5.1.18	E.coli	15604283
glutathione transferase	2.5.1.18	E.coli	15761769
glutathione transferase	2.5.1.18	E.coli	16328982
glutathione transferase	2.5.1.18	E.coli	17176043
glutathione transferase	2.5.1.18	E.coli	17397868
cystathionine gamma-synthase	2.5.1.48	E.coli	10558994
cystathionine gamma-synthase	2.5.1.48	E.coli	11842149
cystathionine gamma-synthase	2.5.1.48	E.coli	12692344
cystathionine gamma-synthase	2.5.1.48	E.coli	14618405
cystathionine gamma-synthase	2.5.1.48	E.coli	14622248
cystathionine gamma-synthase	2.5.1.48	E.coli	15270685
cystathionine gamma-synthase	2.5.1.48	E.coli	15581575
methionine adenosyltransferase	2.5.1.6	E.coli	10415148
methionine adenosyltransferase	2.5.1.6	E.coli	1511738
methionine adenosyltransferase	2.5.1.6	E.coli	16413417
methionine adenosyltransferase	2.5.1.6	E.coli	2764959
methionine adenosyltransferase	2.5.1.6	E.coli	7980467
hydroxymethylbilane synthase	2.5.1.61	E.coli	10546563
hydroxymethylbilane synthase	2.5.1.61	E.coli	10787385
hydroxymethylbilane synthase	2.5.1.61	E.coli	11953837
hydroxymethylbilane synthase	2.5.1.61	E.coli	14559249
hydroxymethylbilane synthase	2.5.1.61	E.coli	1522882
hydroxymethylbilane synthase	2.5.1.61	E.coli	16886091
hydroxymethylbilane synthase	2.5.1.61	E.coli	4067519
hydroxymethylbilane synthase	2.5.1.61	E.coli	7326026
hydroxymethylbilane synthase	2.5.1.61	E.coli	7682572
hydroxymethylbilane synthase	2.5.1.61	E.coli	8023693

hydroxymethylbilane synthase	2.5.1.61	E.coli	9065797
hydroxymethylbilane synthase	2.5.1.61	E.coli	9460994
aspartate transaminase	2.6.1.1	E.coli	8580353
glutamine---fructose-6-phosphate transaminase (isomerizing)	2.6.1.16	E.coli	10329452
glutamine---fructose-6-phosphate transaminase (isomerizing)	2.6.1.16	E.coli	10865863
glutamine---fructose-6-phosphate transaminase (isomerizing)	2.6.1.16	E.coli	11270676
glutamine---fructose-6-phosphate transaminase (isomerizing)	2.6.1.16	E.coli	11895440
glutamine---fructose-6-phosphate transaminase (isomerizing)	2.6.1.16	E.coli	15158264
glutamine---fructose-6-phosphate transaminase (isomerizing)	2.6.1.16	E.coli	15308130
glutamine---fructose-6-phosphate transaminase (isomerizing)	2.6.1.16	E.coli	15613679
glutamine---fructose-6-phosphate transaminase (isomerizing)	2.6.1.16	E.coli	17941647
glutamine---fructose-6-phosphate transaminase (isomerizing)	2.6.1.16	E.coli	6184359
glutamine---fructose-6-phosphate transaminase (isomerizing)	2.6.1.16	E.coli	8394312
glutamine---fructose-6-phosphate transaminase (isomerizing)	2.6.1.16	E.coli	9421478
6-phosphofructokinase	2.7.1.11	E.coli	10323269
6-phosphofructokinase	2.7.1.11	E.coli	10444344
6-phosphofructokinase	2.7.1.11	E.coli	10742704
6-phosphofructokinase	2.7.1.11	E.coli	10909961
6-phosphofructokinase	2.7.1.11	E.coli	10931197
6-phosphofructokinase	2.7.1.11	E.coli	1100622
6-phosphofructokinase	2.7.1.11	E.coli	1100623
6-phosphofructokinase	2.7.1.11	E.coli	11014908
6-phosphofructokinase	2.7.1.11	E.coli	11045948
6-phosphofructokinase	2.7.1.11	E.coli	11058792
6-phosphofructokinase	2.7.1.11	E.coli	11391835
6-phosphofructokinase	2.7.1.11	E.coli	11391836
6-phosphofructokinase	2.7.1.11	E.coli	11391837
6-phosphofructokinase	2.7.1.11	E.coli	11560513
6-phosphofructokinase	2.7.1.11	E.coli	12023862
6-phosphofructokinase	2.7.1.11	E.coli	12051897
6-phosphofructokinase	2.7.1.11	E.coli	12125051
6-phosphofructokinase	2.7.1.11	E.coli	12453221

6-phosphofructokinase	2.7.1.11	E.coli	131232
6-phosphofructokinase	2.7.1.11	E.coli	14585511
6-phosphofructokinase	2.7.1.11	E.coli	147929
6-phosphofructokinase	2.7.1.11	E.coli	149128
6-phosphofructokinase	2.7.1.11	E.coli	15157773
6-phosphofructokinase	2.7.1.11	E.coli	15466668
6-phosphofructokinase	2.7.1.11	E.coli	15504384
6-phosphofructokinase	2.7.1.11	E.coli	156307
6-phosphofructokinase	2.7.1.11	E.coli	15991998
6-phosphofructokinase	2.7.1.11	E.coli	16088331
6-phosphofructokinase	2.7.1.11	E.coli	16103521
6-phosphofructokinase	2.7.1.11	E.coli	16115917
6-phosphofructokinase	2.7.1.11	E.coli	16346876
6-phosphofructokinase	2.7.1.11	E.coli	16377227
6-phosphofructokinase	2.7.1.11	E.coli	1658253
6-phosphofructokinase	2.7.1.11	E.coli	16593209
6-phosphofructokinase	2.7.1.11	E.coli	1825156
6-phosphofructokinase	2.7.1.11	E.coli	1828673
6-phosphofructokinase	2.7.1.11	E.coli	1830744
6-phosphofructokinase	2.7.1.11	E.coli	1833303
6-phosphofructokinase	2.7.1.11	E.coli	191426
6-phosphofructokinase	2.7.1.11	E.coli	2137204
6-phosphofructokinase	2.7.1.11	E.coli	2147292
6-phosphofructokinase	2.7.1.11	E.coli	2149746
6-phosphofructokinase	2.7.1.11	E.coli	2434517
6-phosphofructokinase	2.7.1.11	E.coli	2502581
6-phosphofructokinase	2.7.1.11	E.coli	2522395
6-phosphofructokinase	2.7.1.11	E.coli	2527305
6-phosphofructokinase	2.7.1.11	E.coli	2820531
6-phosphofructokinase	2.7.1.11	E.coli	28629
6-phosphofructokinase	2.7.1.11	E.coli	2933146
6-phosphofructokinase	2.7.1.11	E.coli	2935776
6-phosphofructokinase	2.7.1.11	E.coli	2938549
6-phosphofructokinase	2.7.1.11	E.coli	2956156
6-phosphofructokinase	2.7.1.11	E.coli	2963653
6-phosphofructokinase	2.7.1.11	E.coli	2972577
6-phosphofructokinase	2.7.1.11	E.coli	2981949
6-phosphofructokinase	2.7.1.11	E.coli	3364152
6-phosphofructokinase	2.7.1.11	E.coli	3407760
6-phosphofructokinase	2.7.1.11	E.coli	356174
6-phosphofructokinase	2.7.1.11	E.coli	3768440
6-phosphofructokinase	2.7.1.11	E.coli	3931461
6-phosphofructokinase	2.7.1.11	E.coli	4030556

6-phosphofructokinase	2.7.1.11	E.coli	4243437
6-phosphofructokinase	2.7.1.11	E.coli	4252961
6-phosphofructokinase	2.7.1.11	E.coli	6093562
6-phosphofructokinase	2.7.1.11	E.coli	6133774
6-phosphofructokinase	2.7.1.11	E.coli	6211175
6-phosphofructokinase	2.7.1.11	E.coli	6231923
6-phosphofructokinase	2.7.1.11	E.coli	6232272
6-phosphofructokinase	2.7.1.11	E.coli	6234885
6-phosphofructokinase	2.7.1.11	E.coli	6279392
6-phosphofructokinase	2.7.1.11	E.coli	6325266
6-phosphofructokinase	2.7.1.11	E.coli	6331422
6-phosphofructokinase	2.7.1.11	E.coli	6440018
6-phosphofructokinase	2.7.1.11	E.coli	6444231
6-phosphofructokinase	2.7.1.11	E.coli	6452426
6-phosphofructokinase	2.7.1.11	E.coli	6779470
6-phosphofructokinase	2.7.1.11	E.coli	7233512
6-phosphofructokinase	2.7.1.11	E.coli	7440254
6-phosphofructokinase	2.7.1.11	E.coli	7522206
6-phosphofructokinase	2.7.1.11	E.coli	7589825
6-phosphofructokinase	2.7.1.11	E.coli	7602786
6-phosphofructokinase	2.7.1.11	E.coli	7710770
6-phosphofructokinase	2.7.1.11	E.coli	7875554
6-phosphofructokinase	2.7.1.11	E.coli	8224738
6-phosphofructokinase	2.7.1.11	E.coli	8366430
6-phosphofructokinase	2.7.1.11	E.coli	8514849
6-phosphofructokinase	2.7.1.11	E.coli	8557664
6-phosphofructokinase	2.7.1.11	E.coli	8593533
6-phosphofructokinase	2.7.1.11	E.coli	8643924
6-phosphofructokinase	2.7.1.11	E.coli	8910548
6-phosphofructokinase	2.7.1.11	E.coli	8981075
6-phosphofructokinase	2.7.1.11	E.coli	9267516
6-phosphofructokinase	2.7.1.11	E.coli	9287040
6-phosphofructokinase	2.7.1.11	E.coli	9329694
6-phosphofructokinase	2.7.1.11	E.coli	9371084
6-phosphofructokinase	2.7.1.11	E.coli	9439886
6-phosphofructokinase	2.7.1.11	E.coli	9447322
6-phosphofructokinase	2.7.1.11	E.coli	9555897
6-phosphofructokinase	2.7.1.11	E.coli	9580251
6-phosphofructokinase	2.7.1.11	E.coli	9580875
6-phosphofructokinase	2.7.1.11	E.coli	9608547
6-phosphofructokinase	2.7.1.11	E.coli	9766212
6-phosphofructokinase	2.7.1.11	E.coli	9777012
6-phosphofructokinase	2.7.1.11	E.coli	9851886

6-phosphofructokinase	2.7.1.11	E.coli	9973548
xylulokinase	2.7.1.17	E.coli	10981687
xylulokinase	2.7.1.17	E.coli	15727825
glucokinase	2.7.1.2	E.coli	10456334
glucokinase	2.7.1.2	E.coli	10494657
glucokinase	2.7.1.2	E.coli	10905475
glucokinase	2.7.1.2	E.coli	11311143
glucokinase	2.7.1.2	E.coli	11947549
glucokinase	2.7.1.2	E.coli	11950391
glucokinase	2.7.1.2	E.coli	12941786
glucokinase	2.7.1.2	E.coli	14979565
glucokinase	2.7.1.2	E.coli	15009676
glucokinase	2.7.1.2	E.coli	15016359
glucokinase	2.7.1.2	E.coli	15134337
glucokinase	2.7.1.2	E.coli	15226592
glucokinase	2.7.1.2	E.coli	15277402
glucokinase	2.7.1.2	E.coli	1545870
glucokinase	2.7.1.2	E.coli	15707679
glucokinase	2.7.1.2	E.coli	15955369
glucokinase	2.7.1.2	E.coli	16186394
glucokinase	2.7.1.2	E.coli	16834571
glucokinase	2.7.1.2	E.coli	16899262
glucokinase	2.7.1.2	E.coli	16916947
glucokinase	2.7.1.2	E.coli	2210070
glucokinase	2.7.1.2	E.coli	2584235
glucokinase	2.7.1.2	E.coli	2682629
glucokinase	2.7.1.2	E.coli	6780351
glucokinase	2.7.1.2	E.coli	6836273
glucokinase	2.7.1.2	E.coli	7010073
glucokinase	2.7.1.2	E.coli	7553875
glucokinase	2.7.1.2	E.coli	7821741
glucokinase	2.7.1.2	E.coli	7983782
glucokinase	2.7.1.2	E.coli	8194664
glucokinase	2.7.1.2	E.coli	8344416
glucokinase	2.7.1.2	E.coli	8433729
glucokinase	2.7.1.2	E.coli	8446591
glucokinase	2.7.1.2	E.coli	8549869
glucokinase	2.7.1.2	E.coli	8550593
glucokinase	2.7.1.2	E.coli	8631975
glucokinase	2.7.1.2	E.coli	8690154
glucokinase	2.7.1.2	E.coli	8692940
glucokinase	2.7.1.2	E.coli	8751724
glucokinase	2.7.1.2	E.coli	9113996

glucokinase	2.7.1.2	E.coli	9460079
thymidine kinase	2.7.1.21	E.coli	12750029
thymidine kinase	2.7.1.21	E.coli	12750297
thymidine kinase	2.7.1.21	E.coli	1337357
thymidine kinase	2.7.1.21	E.coli	1455450
thymidine kinase	2.7.1.21	E.coli	14770427
thymidine kinase	2.7.1.21	E.coli	15196542
thymidine kinase	2.7.1.21	E.coli	1525336
thymidine kinase	2.7.1.21	E.coli	1531285
thymidine kinase	2.7.1.21	E.coli	15809747
thymidine kinase	2.7.1.21	E.coli	16336273
thymidine kinase	2.7.1.21	E.coli	16473525
thymidine kinase	2.7.1.21	E.coli	17065087
thymidine kinase	2.7.1.21	E.coli	2025497
thymidine kinase	2.7.1.21	E.coli	348202
thymidine kinase	2.7.1.21	E.coli	3558173
thymidine kinase	2.7.1.21	E.coli	6684556
thymidine kinase	2.7.1.21	E.coli	6840218
thymidine kinase	2.7.1.21	E.coli	8016290
thymidine kinase	2.7.1.21	E.coli	8140585
thymidine kinase	2.7.1.21	E.coli	8432201
thymidine kinase	2.7.1.21	E.coli	8878781
thymidine kinase	2.7.1.21	E.coli	8941385
thymidine kinase	2.7.1.21	E.coli	8955897
thymidine kinase	2.7.1.21	E.coli	9154996
thymidine kinase	2.7.1.21	E.coli	956273
thymidine kinase	2.7.1.21	E.coli	9661884
thymidine kinase	2.7.1.21	E.coli	9816259
riboflavin kinase	2.7.1.26	E.coli	6138398
glycerol kinase	2.7.1.30	E.coli	10.1002/anie.198810401
glycerol kinase	2.7.1.30	E.coli	11388799
glycerol kinase	2.7.1.30	E.coli	11811537
glycerol kinase	2.7.1.30	E.coli	1985967
glycerol kinase	2.7.1.30	E.coli	2547969
glycerol kinase	2.7.1.30	E.coli	4914079
glycerol kinase	2.7.1.30	E.coli	6292169
glycerol kinase	2.7.1.30	E.coli	6440018
glycerol kinase	2.7.1.30	E.coli	9162046
pantothenate kinase	2.7.1.33	E.coli	10625688
pantothenate kinase	2.7.1.33	E.coli	11809413
pantothenate kinase	2.7.1.33	E.coli	12697433
pantothenate kinase	2.7.1.33	E.coli	15176870
pantothenate kinase	2.7.1.33	E.coli	15843025

pantothenate kinase	2.7.1.33	E.coli	16701556
pantothenate kinase	2.7.1.33	E.coli	17323930
pantothenate kinase	2.7.1.33	E.coli	17581817
pantothenate kinase	2.7.1.33	E.coli	9890959
pyruvate kinase	2.7.1.40	E.coli	101523
pyruvate kinase	2.7.1.40	E.coli	11181519
pyruvate kinase	2.7.1.40	E.coli	1175605
pyruvate kinase	2.7.1.40	E.coli	1328007
pyruvate kinase	2.7.1.40	E.coli	1406667
pyruvate kinase	2.7.1.40	E.coli	15028426
pyruvate kinase	2.7.1.40	E.coli	15567985
pyruvate kinase	2.7.1.40	E.coli	16046853
pyruvate kinase	2.7.1.40	E.coli	16511150
pyruvate kinase	2.7.1.40	E.coli	16549526
pyruvate kinase	2.7.1.40	E.coli	1959479
pyruvate kinase	2.7.1.40	E.coli	2387024
pyruvate kinase	2.7.1.40	E.coli	2813362
pyruvate kinase	2.7.1.40	E.coli	2820531
pyruvate kinase	2.7.1.40	E.coli	2846196
pyruvate kinase	2.7.1.40	E.coli	291050
pyruvate kinase	2.7.1.40	E.coli	29278
pyruvate kinase	2.7.1.40	E.coli	2935776
pyruvate kinase	2.7.1.40	E.coli	2970638
pyruvate kinase	2.7.1.40	E.coli	3023262
pyruvate kinase	2.7.1.40	E.coli	3032541
pyruvate kinase	2.7.1.40	E.coli	3159473
pyruvate kinase	2.7.1.40	E.coli	3161219
pyruvate kinase	2.7.1.40	E.coli	3350145
pyruvate kinase	2.7.1.40	E.coli	4053567
pyruvate kinase	2.7.1.40	E.coli	6222515
pyruvate kinase	2.7.1.40	E.coli	6241274
pyruvate kinase	2.7.1.40	E.coli	6268138
pyruvate kinase	2.7.1.40	E.coli	6370232
pyruvate kinase	2.7.1.40	E.coli	6588273
pyruvate kinase	2.7.1.40	E.coli	6682991
pyruvate kinase	2.7.1.40	E.coli	6713301
pyruvate kinase	2.7.1.40	E.coli	7357032
pyruvate kinase	2.7.1.40	E.coli	7961441
pyruvate kinase	2.7.1.40	E.coli	8074527
pyruvate kinase	2.7.1.40	E.coli	8144600
pyruvate kinase	2.7.1.40	E.coli	8436141
pyruvate kinase	2.7.1.40	E.coli	8476115
pyruvate kinase	2.7.1.40	E.coli	8765986

pyruvate kinase	2.7.1.40	E.coli	9252361
uridine kinase	2.7.1.48	E.coli	15735337
uridine kinase	2.7.1.48	E.coli	195585
uridine kinase	2.7.1.48	E.coli	9923963
galactokinase	2.7.1.6	E.coli	12694189
galactokinase	2.7.1.6	E.coli	14596685
galactokinase	2.7.1.6	E.coli	14763977
galactokinase	2.7.1.6	E.coli	16452467
galactokinase	2.7.1.6	E.coli	6836273
N-acylmannosamine kinase	2.7.1.60	E.coli	15987957
glutamate 5-kinase	2.7.2.11	E.coli	12602867
glutamate 5-kinase	2.7.2.11	E.coli	15077666
glutamate 5-kinase	2.7.2.11	E.coli	17449694
aspartate kinase	2.7.2.4	E.coli	16232634
aspartate kinase	2.7.2.4	E.coli	16232840
aspartate kinase	2.7.2.4	E.coli	16658323
aspartate kinase	2.7.2.4	E.coli	16666968
aspartate kinase	2.7.2.4	E.coli	17309616
aspartate kinase	2.7.2.4	E.coli	17350037
aspartate kinase	2.7.2.4	E.coli	17611754
aspartate kinase	2.7.2.4	E.coli	3081884
aspartate kinase	2.7.2.4	E.coli	8299165
adenylate kinase	2.7.4.3	E.coli	126626
adenylate kinase	2.7.4.3	E.coli	14656997
adenylate kinase	2.7.4.3	E.coli	15941717
adenylate kinase	2.7.4.3	E.coli	16668787
adenylate kinase	2.7.4.3	E.coli	6440018
adenylate kinase	2.7.4.3	E.coli	7764491
dTMP kinase	2.7.4.9	E.coli	164949
ribose-phosphate diphosphokinase	2.7.6.1	E.coli	15878857
ribose-phosphate diphosphokinase	2.7.6.1	E.coli	217337
nicotinamide-nucleotide adenylyltransferase	2.7.7.1	E.coli	11248244
nicotinamide-nucleotide adenylyltransferase	2.7.7.1	E.coli	11966442
nicotinamide-nucleotide adenylyltransferase	2.7.7.1	E.coli	17360427
glucose-1-phosphate adenylyltransferase	2.7.7.27	E.coli	10217430
glucose-1-phosphate adenylyltransferase	2.7.7.27	E.coli	10606772
glucose-1-phosphate adenylyltransferase	2.7.7.27	E.coli	10625679
glucose-1-phosphate adenylyltransferase	2.7.7.27	E.coli	11237727
glucose-1-phosphate adenylyltransferase	2.7.7.27	E.coli	11387976
glucose-1-phosphate adenylyltransferase	2.7.7.27	E.coli	11524424
glucose-1-phosphate adenylyltransferase	2.7.7.27	E.coli	11525517
glucose-1-phosphate adenylyltransferase	2.7.7.27	E.coli	11830133
glucose-1-phosphate adenylyltransferase	2.7.7.27	E.coli	11830676

glucose-1-phosphate adenylyltransferase	2.7.7.27	E.coli	12111225
glucose-1-phosphate adenylyltransferase	2.7.7.27	E.coli	12231679
glucose-1-phosphate adenylyltransferase	2.7.7.27	E.coli	12569408
glucose-1-phosphate adenylyltransferase	2.7.7.27	E.coli	12794190
glucose-1-phosphate adenylyltransferase	2.7.7.27	E.coli	15122037
glucose-1-phosphate adenylyltransferase	2.7.7.27	E.coli	15631980
glucose-1-phosphate adenylyltransferase	2.7.7.27	E.coli	15863446
glucose-1-phosphate adenylyltransferase	2.7.7.27	E.coli	16228397
glucose-1-phosphate adenylyltransferase	2.7.7.27	E.coli	16299180
glucose-1-phosphate adenylyltransferase	2.7.7.27	E.coli	1663749
glucose-1-phosphate adenylyltransferase	2.7.7.27	E.coli	17406793
glucose-1-phosphate adenylyltransferase	2.7.7.27	E.coli	2162151
glucose-1-phosphate adenylyltransferase	2.7.7.27	E.coli	224050
glucose-1-phosphate adenylyltransferase	2.7.7.27	E.coli	2981798
glucose-1-phosphate adenylyltransferase	2.7.7.27	E.coli	381303
glucose-1-phosphate adenylyltransferase	2.7.7.27	E.coli	8676861
glucose-1-phosphate adenylyltransferase	2.7.7.27	E.coli	9244254
glucose-1-phosphate adenylyltransferase	2.7.7.27	E.coli	9628013
glucose-1-phosphate adenylyltransferase	2.7.7.27	E.coli	9841881
pantetheine-phosphate adenylyltransferase	2.7.7.3	E.coli	1746161
sulfate adenylyltransferase	2.7.7.4	E.coli	10759525
sulfate adenylyltransferase	2.7.7.4	E.coli	10956658
sulfate adenylyltransferase	2.7.7.4	E.coli	11157739
sulfate adenylyltransferase	2.7.7.4	E.coli	14613928
sulfate adenylyltransferase	2.7.7.4	E.coli	16008502
sulfate adenylyltransferase	2.7.7.4	E.coli	17095009
sulfate adenylyltransferase	2.7.7.4	E.coli	8206850
sulfate adenylyltransferase	2.7.7.4	E.coli	9880353
phosphatidate cytidylyltransferase	2.7.7.41	E.coli	11985865
phosphatidate cytidylyltransferase	2.7.7.41	E.coli	9345289
polyribonucleotide nucleotidyltransferase	2.7.7.8	E.coli	126862
UTP---glucose-1-phosphate uridylyltransferase	2.7.7.9	E.coli	11171080
UTP---glucose-1-phosphate uridylyltransferase	2.7.7.9	E.coli	1149741
UTP---glucose-1-phosphate uridylyltransferase	2.7.7.9	E.coli	12088504
UTP---glucose-1-phosphate uridylyltransferase	2.7.7.9	E.coli	15274139
biotin synthase	2.8.1.6	E.coli	16195795
alkaline phosphatase	3.1.3.1	E.coli	11029583
alkaline phosphatase	3.1.3.1	E.coli	11139445
alkaline phosphatase	3.1.3.1	E.coli	12412807

alkaline phosphatase	3.1.3.1	E.coli	1445337
alkaline phosphatase	3.1.3.1	E.coli	14560000
alkaline phosphatase	3.1.3.1	E.coli	3509742
alkaline phosphatase	3.1.3.1	E.coli	7032602
fructose-bisphosphatase	3.1.3.11	E.coli	10327613
fructose-bisphosphatase	3.1.3.11	E.coli	10393302
fructose-bisphosphatase	3.1.3.11	E.coli	10773464
fructose-bisphosphatase	3.1.3.11	E.coli	11536627
fructose-bisphosphatase	3.1.3.11	E.coli	11864619
fructose-bisphosphatase	3.1.3.11	E.coli	12190028
fructose-bisphosphatase	3.1.3.11	E.coli	12686616
fructose-bisphosphatase	3.1.3.11	E.coli	15225753
fructose-bisphosphatase	3.1.3.11	E.coli	15498578
fructose-bisphosphatase	3.1.3.11	E.coli	15631980
fructose-bisphosphatase	3.1.3.11	E.coli	16199065
fructose-bisphosphatase	3.1.3.11	E.coli	16580859
fructose-bisphosphatase	3.1.3.11	E.coli	16593209
fructose-bisphosphatase	3.1.3.11	E.coli	16657971
fructose-bisphosphatase	3.1.3.11	E.coli	16814784
fructose-bisphosphatase	3.1.3.11	E.coli	16857246
fructose-bisphosphatase	3.1.3.11	E.coli	2835013
fructose-bisphosphatase	3.1.3.11	E.coli	2983680
fructose-bisphosphatase	3.1.3.11	E.coli	3068502
fructose-bisphosphatase	3.1.3.11	E.coli	3291467
fructose-bisphosphatase	3.1.3.11	E.coli	4342496
fructose-bisphosphatase	3.1.3.11	E.coli	7532742
fructose-bisphosphatase	3.1.3.11	E.coli	7552262
fructose-bisphosphatase	3.1.3.11	E.coli	7558035
fructose-bisphosphatase	3.1.3.11	E.coli	7579072
fructose-bisphosphatase	3.1.3.11	E.coli	7592860
fructose-bisphosphatase	3.1.3.11	E.coli	8043646
acid phosphatase	3.1.3.2	E.coli	10471332
acid phosphatase	3.1.3.2	E.coli	10646119
acid phosphatase	3.1.3.2	E.coli	1364364
acid phosphatase	3.1.3.2	E.coli	2153810
acid phosphatase	3.1.3.2	E.coli	2545859
acid phosphatase	3.1.3.2	E.coli	2713876
acid phosphatase	3.1.3.2	E.coli	9370316
inositol-phosphate phosphatase	3.1.3.25	E.coli	12479670
inositol-phosphate phosphatase	3.1.3.25	E.coli	15809430
inositol-phosphate phosphatase	3.1.3.25	E.coli	9462881
phosphoserine phosphatase	3.1.3.3	E.coli	8858931
5'-nucleotidase	3.1.3.5	E.coli	10506947

5'-nucleotidase	3.1.3.5	E.coli	10617137
5'-nucleotidase	3.1.3.5	E.coli	10766785
5'-nucleotidase	3.1.3.5	E.coli	10869532
5'-nucleotidase	3.1.3.5	E.coli	11408527
5'-nucleotidase	3.1.3.5	E.coli	12030367
5'-nucleotidase	3.1.3.5	E.coli	12061138
5'-nucleotidase	3.1.3.5	E.coli	12204768
5'-nucleotidase	3.1.3.5	E.coli	12493585
5'-nucleotidase	3.1.3.5	E.coli	12571440
5'-nucleotidase	3.1.3.5	E.coli	12667292
5'-nucleotidase	3.1.3.5	E.coli	15748706
5'-nucleotidase	3.1.3.5	E.coli	15946667
5'-nucleotidase	3.1.3.5	E.coli	6326848
5'-nucleotidase	3.1.3.5	E.coli	7999131
5'-nucleotidase	3.1.3.5	E.coli	9009712
5'-nucleotidase	3.1.3.5	E.coli	9315889
5'-nucleotidase	3.1.3.5	E.coli	9806332
arylsulfatase	3.1.6.1	E.coli	6233132
alpha-glucosidase	3.2.1.20	E.coli	10619707
alpha-glucosidase	3.2.1.20	E.coli	10810293
alpha-glucosidase	3.2.1.20	E.coli	11134937
alpha-glucosidase	3.2.1.20	E.coli	11230125
alpha-glucosidase	3.2.1.20	E.coli	11404235
alpha-glucosidase	3.2.1.20	E.coli	16233203
alpha-glucosidase	3.2.1.20	E.coli	16233375
alpha-glucosidase	3.2.1.20	E.coli	7626594
alpha-glucosidase	3.2.1.20	E.coli	8625892
beta-glucosidase	3.2.1.21	E.coli	10824094
beta-glucosidase	3.2.1.21	E.coli	15966329
beta-glucosidase	3.2.1.21	E.coli	6773958
membrane alanyl aminopeptidase	3.4.11.2	E.coli	11751433
membrane alanyl aminopeptidase	3.4.11.2	E.coli	12075625
membrane alanyl aminopeptidase	3.4.11.2	E.coli	16019130
dihydroorotase	3.5.2.3	E.coli	3345746
dihydroorotase	3.5.2.3	E.coli	4092695
dihydroorotase	3.5.2.3	E.coli	42399
dihydroorotase	3.5.2.3	E.coli	6115855
GTP cyclohydrolase I	3.5.4.16	E.coli	12392559
GTP cyclohydrolase I	3.5.4.16	E.coli	12855421
GTP cyclohydrolase I	3.5.4.16	E.coli	1459137
GTP cyclohydrolase I	3.5.4.16	E.coli	15044686
GTP cyclohydrolase I	3.5.4.16	E.coli	16179591
GTP cyclohydrolase I	3.5.4.16	E.coli	16636057

GTP cyclohydrolase I	3.5.4.16	E.coli	737222
GTP cyclohydrolase I	3.5.4.16	E.coli	7521513
GTP cyclohydrolase I	3.5.4.16	E.coli	9182249
GTP cyclohydrolase I	3.5.4.16	E.coli	9444617
H+-transporting two-sector ATPase	3.6.3.14	E.coli	11744700
H+-transporting two-sector ATPase	3.6.3.14	E.coli	11893513
H+-transporting two-sector ATPase	3.6.3.14	E.coli	12587531
H+-transporting two-sector ATPase	3.6.3.14	E.coli	15712234
H+-transporting two-sector ATPase	3.6.3.14	E.coli	16510118
H+-transporting two-sector ATPase	3.6.3.14	E.coli	16730639
H+-transporting two-sector ATPase	3.6.3.14	E.coli	2532597
H+-transporting two-sector ATPase	3.6.3.14	E.coli	2889730
H+-transporting two-sector ATPase	3.6.3.14	E.coli	6456904
H+-transporting two-sector ATPase	3.6.3.14	E.coli	8516333
glutamate decarboxylase	4.1.1.15	E.coli	10331265
glutamate decarboxylase	4.1.1.15	E.coli	12196588
glutamate decarboxylase	4.1.1.15	E.coli	12746320
glutamate decarboxylase	4.1.1.15	E.coli	15210535
glutamate decarboxylase	4.1.1.15	E.coli	15581395
glutamate decarboxylase	4.1.1.15	E.coli	1697032
glutamate decarboxylase	4.1.1.15	E.coli	17044036
glutamate decarboxylase	4.1.1.15	E.coli	1976015
glutamate decarboxylase	4.1.1.15	E.coli	2180326
glutamate decarboxylase	4.1.1.15	E.coli	2735448
glutamate decarboxylase	4.1.1.15	E.coli	2857768
glutamate decarboxylase	4.1.1.15	E.coli	3896834
glutamate decarboxylase	4.1.1.15	E.coli	6975381
glutamate decarboxylase	4.1.1.15	E.coli	7702443
glutamate decarboxylase	4.1.1.15	E.coli	7885536
glutamate decarboxylase	4.1.1.15	E.coli	8302162
glutamate decarboxylase	4.1.1.15	E.coli	9011754
glutamate decarboxylase	4.1.1.15	E.coli	9053794
ornithine decarboxylase	4.1.1.17	E.coli	10069996
ornithine decarboxylase	4.1.1.17	E.coli	10216947
ornithine decarboxylase	4.1.1.17	E.coli	10319188
ornithine decarboxylase	4.1.1.17	E.coli	10320037
ornithine decarboxylase	4.1.1.17	E.coli	10321508
ornithine decarboxylase	4.1.1.17	E.coli	10430664
ornithine decarboxylase	4.1.1.17	E.coli	10453061
ornithine decarboxylase	4.1.1.17	E.coli	10456943
ornithine decarboxylase	4.1.1.17	E.coli	10473083
ornithine decarboxylase	4.1.1.17	E.coli	10485326
ornithine decarboxylase	4.1.1.17	E.coli	10544213

ornithine decarboxylase	4.1.1.17	E.coli	10550568
ornithine decarboxylase	4.1.1.17	E.coli	10564512
ornithine decarboxylase	4.1.1.17	E.coli	10589756
ornithine decarboxylase	4.1.1.17	E.coli	10593613
ornithine decarboxylase	4.1.1.17	E.coli	10607762
ornithine decarboxylase	4.1.1.17	E.coli	10629084
ornithine decarboxylase	4.1.1.17	E.coli	10712236
ornithine decarboxylase	4.1.1.17	E.coli	10713131
ornithine decarboxylase	4.1.1.17	E.coli	10760944
ornithine decarboxylase	4.1.1.17	E.coli	10772389
ornithine decarboxylase	4.1.1.17	E.coli	10816435
ornithine decarboxylase	4.1.1.17	E.coli	10817834
ornithine decarboxylase	4.1.1.17	E.coli	10882097
ornithine decarboxylase	4.1.1.17	E.coli	10931831
ornithine decarboxylase	4.1.1.17	E.coli	10940513
ornithine decarboxylase	4.1.1.17	E.coli	10965017
ornithine decarboxylase	4.1.1.17	E.coli	11003584
ornithine decarboxylase	4.1.1.17	E.coli	11085920
ornithine decarboxylase	4.1.1.17	E.coli	11095648
ornithine decarboxylase	4.1.1.17	E.coli	11137705
ornithine decarboxylase	4.1.1.17	E.coli	11180396
ornithine decarboxylase	4.1.1.17	E.coli	11235918
ornithine decarboxylase	4.1.1.17	E.coli	11355005
ornithine decarboxylase	4.1.1.17	E.coli	11376395
ornithine decarboxylase	4.1.1.17	E.coli	11408092
ornithine decarboxylase	4.1.1.17	E.coli	11408253
ornithine decarboxylase	4.1.1.17	E.coli	11408542
ornithine decarboxylase	4.1.1.17	E.coli	11413269
ornithine decarboxylase	4.1.1.17	E.coli	11540835
ornithine decarboxylase	4.1.1.17	E.coli	11558274
ornithine decarboxylase	4.1.1.17	E.coli	11736657
ornithine decarboxylase	4.1.1.17	E.coli	11782361
ornithine decarboxylase	4.1.1.17	E.coli	11852055
ornithine decarboxylase	4.1.1.17	E.coli	11883715
ornithine decarboxylase	4.1.1.17	E.coli	11922393
ornithine decarboxylase	4.1.1.17	E.coli	11923270
ornithine decarboxylase	4.1.1.17	E.coli	11964084
ornithine decarboxylase	4.1.1.17	E.coli	11997243
ornithine decarboxylase	4.1.1.17	E.coli	12054570
ornithine decarboxylase	4.1.1.17	E.coli	12105848
ornithine decarboxylase	4.1.1.17	E.coli	12148577
ornithine decarboxylase	4.1.1.17	E.coli	12355213
ornithine decarboxylase	4.1.1.17	E.coli	12452334

ornithine decarboxylase	4.1.1.17	E.coli	12497077
ornithine decarboxylase	4.1.1.17	E.coli	12527115
ornithine decarboxylase	4.1.1.17	E.coli	12663506
ornithine decarboxylase	4.1.1.17	E.coli	12716308
ornithine decarboxylase	4.1.1.17	E.coli	12716758
ornithine decarboxylase	4.1.1.17	E.coli	12766050
ornithine decarboxylase	4.1.1.17	E.coli	12816757
ornithine decarboxylase	4.1.1.17	E.coli	12856719
ornithine decarboxylase	4.1.1.17	E.coli	12882169
ornithine decarboxylase	4.1.1.17	E.coli	1289667
ornithine decarboxylase	4.1.1.17	E.coli	1324153
ornithine decarboxylase	4.1.1.17	E.coli	1360468
ornithine decarboxylase	4.1.1.17	E.coli	1397089
ornithine decarboxylase	4.1.1.17	E.coli	1407701
ornithine decarboxylase	4.1.1.17	E.coli	1409247
ornithine decarboxylase	4.1.1.17	E.coli	1417733
ornithine decarboxylase	4.1.1.17	E.coli	14769544
ornithine decarboxylase	4.1.1.17	E.coli	1495349
ornithine decarboxylase	4.1.1.17	E.coli	15002659
ornithine decarboxylase	4.1.1.17	E.coli	1511780
ornithine decarboxylase	4.1.1.17	E.coli	15120115
ornithine decarboxylase	4.1.1.17	E.coli	15180186
ornithine decarboxylase	4.1.1.17	E.coli	1521915
ornithine decarboxylase	4.1.1.17	E.coli	15223770
ornithine decarboxylase	4.1.1.17	E.coli	15228220
ornithine decarboxylase	4.1.1.17	E.coli	15233741
ornithine decarboxylase	4.1.1.17	E.coli	15247138
ornithine decarboxylase	4.1.1.17	E.coli	15296840
ornithine decarboxylase	4.1.1.17	E.coli	15306645
ornithine decarboxylase	4.1.1.17	E.coli	15355308
ornithine decarboxylase	4.1.1.17	E.coli	15514084
ornithine decarboxylase	4.1.1.17	E.coli	15538383
ornithine decarboxylase	4.1.1.17	E.coli	15539331
ornithine decarboxylase	4.1.1.17	E.coli	1563337
ornithine decarboxylase	4.1.1.17	E.coli	15697240
ornithine decarboxylase	4.1.1.17	E.coli	1569947
ornithine decarboxylase	4.1.1.17	E.coli	15716048
ornithine decarboxylase	4.1.1.17	E.coli	15843384
ornithine decarboxylase	4.1.1.17	E.coli	1584960
ornithine decarboxylase	4.1.1.17	E.coli	1590311
ornithine decarboxylase	4.1.1.17	E.coli	1601800
ornithine decarboxylase	4.1.1.17	E.coli	16091008
ornithine decarboxylase	4.1.1.17	E.coli	16168128

ornithine decarboxylase	4.1.1.17	E.coli	16170669
ornithine decarboxylase	4.1.1.17	E.coli	16181115
ornithine decarboxylase	4.1.1.17	E.coli	16223706
ornithine decarboxylase	4.1.1.17	E.coli	16230862
ornithine decarboxylase	4.1.1.17	E.coli	16290266
ornithine decarboxylase	4.1.1.17	E.coli	16342411
ornithine decarboxylase	4.1.1.17	E.coli	1641775
ornithine decarboxylase	4.1.1.17	E.coli	16445292
ornithine decarboxylase	4.1.1.17	E.coli	1655898
ornithine decarboxylase	4.1.1.17	E.coli	16568078
ornithine decarboxylase	4.1.1.17	E.coli	16630547
ornithine decarboxylase	4.1.1.17	E.coli	16662219
ornithine decarboxylase	4.1.1.17	E.coli	16666570
ornithine decarboxylase	4.1.1.17	E.coli	16678846
ornithine decarboxylase	4.1.1.17	E.coli	16729674
ornithine decarboxylase	4.1.1.17	E.coli	1697882
ornithine decarboxylase	4.1.1.17	E.coli	1703390
ornithine decarboxylase	4.1.1.17	E.coli	1733364
ornithine decarboxylase	4.1.1.17	E.coli	17407445
ornithine decarboxylase	4.1.1.17	E.coli	1745018
ornithine decarboxylase	4.1.1.17	E.coli	1782416
ornithine decarboxylase	4.1.1.17	E.coli	1814556
ornithine decarboxylase	4.1.1.17	E.coli	1814755
ornithine decarboxylase	4.1.1.17	E.coli	1831810
ornithine decarboxylase	4.1.1.17	E.coli	1846091
ornithine decarboxylase	4.1.1.17	E.coli	1878921
ornithine decarboxylase	4.1.1.17	E.coli	1884248
ornithine decarboxylase	4.1.1.17	E.coli	1892753
ornithine decarboxylase	4.1.1.17	E.coli	1900385
ornithine decarboxylase	4.1.1.17	E.coli	1932775
ornithine decarboxylase	4.1.1.17	E.coli	1940203
ornithine decarboxylase	4.1.1.17	E.coli	1962522
ornithine decarboxylase	4.1.1.17	E.coli	196870
ornithine decarboxylase	4.1.1.17	E.coli	1997184
ornithine decarboxylase	4.1.1.17	E.coli	2006469
ornithine decarboxylase	4.1.1.17	E.coli	2009332
ornithine decarboxylase	4.1.1.17	E.coli	2019760
ornithine decarboxylase	4.1.1.17	E.coli	203259
ornithine decarboxylase	4.1.1.17	E.coli	2051775
ornithine decarboxylase	4.1.1.17	E.coli	2088816
ornithine decarboxylase	4.1.1.17	E.coli	2118148
ornithine decarboxylase	4.1.1.17	E.coli	2148056
ornithine decarboxylase	4.1.1.17	E.coli	2160044

ornithine decarboxylase	4.1.1.17	E.coli	2197525
ornithine decarboxylase	4.1.1.17	E.coli	2210666
ornithine decarboxylase	4.1.1.17	E.coli	2243540
ornithine decarboxylase	4.1.1.17	E.coli	2293084
ornithine decarboxylase	4.1.1.17	E.coli	2296762
ornithine decarboxylase	4.1.1.17	E.coli	2298913
ornithine decarboxylase	4.1.1.17	E.coli	2409817
ornithine decarboxylase	4.1.1.17	E.coli	2469492
ornithine decarboxylase	4.1.1.17	E.coli	2472814
ornithine decarboxylase	4.1.1.17	E.coli	2493794
ornithine decarboxylase	4.1.1.17	E.coli	2494779
ornithine decarboxylase	4.1.1.17	E.coli	2497460
ornithine decarboxylase	4.1.1.17	E.coli	2497556
ornithine decarboxylase	4.1.1.17	E.coli	2505399
ornithine decarboxylase	4.1.1.17	E.coli	2505959
ornithine decarboxylase	4.1.1.17	E.coli	2507383
ornithine decarboxylase	4.1.1.17	E.coli	2507471
ornithine decarboxylase	4.1.1.17	E.coli	2525760
ornithine decarboxylase	4.1.1.17	E.coli	2553150
ornithine decarboxylase	4.1.1.17	E.coli	2610929
ornithine decarboxylase	4.1.1.17	E.coli	2651129
ornithine decarboxylase	4.1.1.17	E.coli	2699646
ornithine decarboxylase	4.1.1.17	E.coli	2754510
ornithine decarboxylase	4.1.1.17	E.coli	2775206
ornithine decarboxylase	4.1.1.17	E.coli	2829727
ornithine decarboxylase	4.1.1.17	E.coli	2840461
ornithine decarboxylase	4.1.1.17	E.coli	2915649
ornithine decarboxylase	4.1.1.17	E.coli	2916900
ornithine decarboxylase	4.1.1.17	E.coli	2977154
ornithine decarboxylase	4.1.1.17	E.coli	2979197
ornithine decarboxylase	4.1.1.17	E.coli	2986953
ornithine decarboxylase	4.1.1.17	E.coli	3004707
ornithine decarboxylase	4.1.1.17	E.coli	3009424
ornithine decarboxylase	4.1.1.17	E.coli	3022056
ornithine decarboxylase	4.1.1.17	E.coli	3036091
ornithine decarboxylase	4.1.1.17	E.coli	3037250
ornithine decarboxylase	4.1.1.17	E.coli	3040821
ornithine decarboxylase	4.1.1.17	E.coli	3082276
ornithine decarboxylase	4.1.1.17	E.coli	3084209
ornithine decarboxylase	4.1.1.17	E.coli	3084872
ornithine decarboxylase	4.1.1.17	E.coli	3086160
ornithine decarboxylase	4.1.1.17	E.coli	3092827
ornithine decarboxylase	4.1.1.17	E.coli	3093095

ornithine decarboxylase	4.1.1.17	E.coli	3096557
ornithine decarboxylase	4.1.1.17	E.coli	3100897
ornithine decarboxylase	4.1.1.17	E.coli	3102397
ornithine decarboxylase	4.1.1.17	E.coli	3105968
ornithine decarboxylase	4.1.1.17	E.coli	3106075
ornithine decarboxylase	4.1.1.17	E.coli	3108666
ornithine decarboxylase	4.1.1.17	E.coli	3109979
ornithine decarboxylase	4.1.1.17	E.coli	3109985
ornithine decarboxylase	4.1.1.17	E.coli	3111384
ornithine decarboxylase	4.1.1.17	E.coli	3113732
ornithine decarboxylase	4.1.1.17	E.coli	3117720
ornithine decarboxylase	4.1.1.17	E.coli	3121457
ornithine decarboxylase	4.1.1.17	E.coli	3122042
ornithine decarboxylase	4.1.1.17	E.coli	3129184
ornithine decarboxylase	4.1.1.17	E.coli	3130188
ornithine decarboxylase	4.1.1.17	E.coli	3139441
ornithine decarboxylase	4.1.1.17	E.coli	3141045
ornithine decarboxylase	4.1.1.17	E.coli	3143046
ornithine decarboxylase	4.1.1.17	E.coli	3180091
ornithine decarboxylase	4.1.1.17	E.coli	3279036
ornithine decarboxylase	4.1.1.17	E.coli	3328430
ornithine decarboxylase	4.1.1.17	E.coli	3356404
ornithine decarboxylase	4.1.1.17	E.coli	3403538
ornithine decarboxylase	4.1.1.17	E.coli	3443298
ornithine decarboxylase	4.1.1.17	E.coli	3538740
ornithine decarboxylase	4.1.1.17	E.coli	3548994
ornithine decarboxylase	4.1.1.17	E.coli	3661847
ornithine decarboxylase	4.1.1.17	E.coli	3672608
ornithine decarboxylase	4.1.1.17	E.coli	3688216
ornithine decarboxylase	4.1.1.17	E.coli	3729588
ornithine decarboxylase	4.1.1.17	E.coli	3743773
ornithine decarboxylase	4.1.1.17	E.coli	3753036
ornithine decarboxylase	4.1.1.17	E.coli	3775249
ornithine decarboxylase	4.1.1.17	E.coli	3794781
ornithine decarboxylase	4.1.1.17	E.coli	3857388
ornithine decarboxylase	4.1.1.17	E.coli	3901680
ornithine decarboxylase	4.1.1.17	E.coli	3905315
ornithine decarboxylase	4.1.1.17	E.coli	3921243
ornithine decarboxylase	4.1.1.17	E.coli	3926303
ornithine decarboxylase	4.1.1.17	E.coli	3930649
ornithine decarboxylase	4.1.1.17	E.coli	3931300
ornithine decarboxylase	4.1.1.17	E.coli	3934106
ornithine decarboxylase	4.1.1.17	E.coli	3999751

ornithine decarboxylase	4.1.1.17	E.coli	4029343
ornithine decarboxylase	4.1.1.17	E.coli	4053280
ornithine decarboxylase	4.1.1.17	E.coli	497279
ornithine decarboxylase	4.1.1.17	E.coli	6124275
ornithine decarboxylase	4.1.1.17	E.coli	6178351
ornithine decarboxylase	4.1.1.17	E.coli	6190690
ornithine decarboxylase	4.1.1.17	E.coli	6192925
ornithine decarboxylase	4.1.1.17	E.coli	6223191
ornithine decarboxylase	4.1.1.17	E.coli	6256169
ornithine decarboxylase	4.1.1.17	E.coli	6272311
ornithine decarboxylase	4.1.1.17	E.coli	6307502
ornithine decarboxylase	4.1.1.17	E.coli	6365078
ornithine decarboxylase	4.1.1.17	E.coli	6432312
ornithine decarboxylase	4.1.1.17	E.coli	6432848
ornithine decarboxylase	4.1.1.17	E.coli	6439208
ornithine decarboxylase	4.1.1.17	E.coli	6440787
ornithine decarboxylase	4.1.1.17	E.coli	6445842
ornithine decarboxylase	4.1.1.17	E.coli	6467454
ornithine decarboxylase	4.1.1.17	E.coli	6571411
ornithine decarboxylase	4.1.1.17	E.coli	6591862
ornithine decarboxylase	4.1.1.17	E.coli	6624798
ornithine decarboxylase	4.1.1.17	E.coli	6692409
ornithine decarboxylase	4.1.1.17	E.coli	6696980
ornithine decarboxylase	4.1.1.17	E.coli	6721578
ornithine decarboxylase	4.1.1.17	E.coli	6750139
ornithine decarboxylase	4.1.1.17	E.coli	6812570
ornithine decarboxylase	4.1.1.17	E.coli	6813460
ornithine decarboxylase	4.1.1.17	E.coli	6865777
ornithine decarboxylase	4.1.1.17	E.coli	6891933
ornithine decarboxylase	4.1.1.17	E.coli	7093948
ornithine decarboxylase	4.1.1.17	E.coli	7104206
ornithine decarboxylase	4.1.1.17	E.coli	7159401
ornithine decarboxylase	4.1.1.17	E.coli	7205597
ornithine decarboxylase	4.1.1.17	E.coli	7310281
ornithine decarboxylase	4.1.1.17	E.coli	7381752
ornithine decarboxylase	4.1.1.17	E.coli	7426404
ornithine decarboxylase	4.1.1.17	E.coli	7488168
ornithine decarboxylase	4.1.1.17	E.coli	7498733
ornithine decarboxylase	4.1.1.17	E.coli	7525612
ornithine decarboxylase	4.1.1.17	E.coli	7616440
ornithine decarboxylase	4.1.1.17	E.coli	7628376
ornithine decarboxylase	4.1.1.17	E.coli	7656288
ornithine decarboxylase	4.1.1.17	E.coli	7718766

ornithine decarboxylase	4.1.1.17	E.coli	7813017
ornithine decarboxylase	4.1.1.17	E.coli	7823874
ornithine decarboxylase	4.1.1.17	E.coli	7865470
ornithine decarboxylase	4.1.1.17	E.coli	7872745
ornithine decarboxylase	4.1.1.17	E.coli	7874572
ornithine decarboxylase	4.1.1.17	E.coli	7895420
ornithine decarboxylase	4.1.1.17	E.coli	7929646
ornithine decarboxylase	4.1.1.17	E.coli	7943199
ornithine decarboxylase	4.1.1.17	E.coli	7951165
ornithine decarboxylase	4.1.1.17	E.coli	7965748
ornithine decarboxylase	4.1.1.17	E.coli	7972938
ornithine decarboxylase	4.1.1.17	E.coli	7981636
ornithine decarboxylase	4.1.1.17	E.coli	8010156
ornithine decarboxylase	4.1.1.17	E.coli	8028020
ornithine decarboxylase	4.1.1.17	E.coli	8065308
ornithine decarboxylase	4.1.1.17	E.coli	8095973
ornithine decarboxylase	4.1.1.17	E.coli	8110472
ornithine decarboxylase	4.1.1.17	E.coli	8140036
ornithine decarboxylase	4.1.1.17	E.coli	8141779
ornithine decarboxylase	4.1.1.17	E.coli	8152342
ornithine decarboxylase	4.1.1.17	E.coli	8185631
ornithine decarboxylase	4.1.1.17	E.coli	8190721
ornithine decarboxylase	4.1.1.17	E.coli	8242794
ornithine decarboxylase	4.1.1.17	E.coli	8344985
ornithine decarboxylase	4.1.1.17	E.coli	8368314
ornithine decarboxylase	4.1.1.17	E.coli	8374143
ornithine decarboxylase	4.1.1.17	E.coli	8419528
ornithine decarboxylase	4.1.1.17	E.coli	8447420
ornithine decarboxylase	4.1.1.17	E.coli	8453677
ornithine decarboxylase	4.1.1.17	E.coli	8462726
ornithine decarboxylase	4.1.1.17	E.coli	8465553
ornithine decarboxylase	4.1.1.17	E.coli	8478959
ornithine decarboxylase	4.1.1.17	E.coli	8501729
ornithine decarboxylase	4.1.1.17	E.coli	8538189
ornithine decarboxylase	4.1.1.17	E.coli	8549635
ornithine decarboxylase	4.1.1.17	E.coli	8572176
ornithine decarboxylase	4.1.1.17	E.coli	8660289
ornithine decarboxylase	4.1.1.17	E.coli	8693031
ornithine decarboxylase	4.1.1.17	E.coli	8707896
ornithine decarboxylase	4.1.1.17	E.coli	8727257
ornithine decarboxylase	4.1.1.17	E.coli	8760120
ornithine decarboxylase	4.1.1.17	E.coli	8760129
ornithine decarboxylase	4.1.1.17	E.coli	8768305

ornithine decarboxylase	4.1.1.17	E.coli	8777294
ornithine decarboxylase	4.1.1.17	E.coli	8814137
ornithine decarboxylase	4.1.1.17	E.coli	8848835
ornithine decarboxylase	4.1.1.17	E.coli	8858522
ornithine decarboxylase	4.1.1.17	E.coli	8878500
ornithine decarboxylase	4.1.1.17	E.coli	8882155
ornithine decarboxylase	4.1.1.17	E.coli	8912847
ornithine decarboxylase	4.1.1.17	E.coli	8944705
ornithine decarboxylase	4.1.1.17	E.coli	9009157
ornithine decarboxylase	4.1.1.17	E.coli	9016399
ornithine decarboxylase	4.1.1.17	E.coli	9017896
ornithine decarboxylase	4.1.1.17	E.coli	9022291
ornithine decarboxylase	4.1.1.17	E.coli	9024941
ornithine decarboxylase	4.1.1.17	E.coli	9063811
ornithine decarboxylase	4.1.1.17	E.coli	9073141
ornithine decarboxylase	4.1.1.17	E.coli	9134011
ornithine decarboxylase	4.1.1.17	E.coli	9142900
ornithine decarboxylase	4.1.1.17	E.coli	9191978
ornithine decarboxylase	4.1.1.17	E.coli	9213218
ornithine decarboxylase	4.1.1.17	E.coli	9223345
ornithine decarboxylase	4.1.1.17	E.coli	9224728
ornithine decarboxylase	4.1.1.17	E.coli	9251105
ornithine decarboxylase	4.1.1.17	E.coli	9252524
ornithine decarboxylase	4.1.1.17	E.coli	9294258
ornithine decarboxylase	4.1.1.17	E.coli	9368191
ornithine decarboxylase	4.1.1.17	E.coli	9371082
ornithine decarboxylase	4.1.1.17	E.coli	9454972
ornithine decarboxylase	4.1.1.17	E.coli	9458728
ornithine decarboxylase	4.1.1.17	E.coli	9468098
ornithine decarboxylase	4.1.1.17	E.coli	9495243
ornithine decarboxylase	4.1.1.17	E.coli	9525811
ornithine decarboxylase	4.1.1.17	E.coli	9590135
ornithine decarboxylase	4.1.1.17	E.coli	9609384
ornithine decarboxylase	4.1.1.17	E.coli	9612269
ornithine decarboxylase	4.1.1.17	E.coli	9624108
ornithine decarboxylase	4.1.1.17	E.coli	9648877
ornithine decarboxylase	4.1.1.17	E.coli	9685330
ornithine decarboxylase	4.1.1.17	E.coli	9688665
ornithine decarboxylase	4.1.1.17	E.coli	9769382
ornithine decarboxylase	4.1.1.17	E.coli	9772292
ornithine decarboxylase	4.1.1.17	E.coli	9795249
ornithine decarboxylase	4.1.1.17	E.coli	9806166
ornithine decarboxylase	4.1.1.17	E.coli	9829706

ornithine decarboxylase	4.1.1.17	E.coli	9868187
ornithine decarboxylase	4.1.1.17	E.coli	9884080
ornithine decarboxylase	4.1.1.17	E.coli	9890191
arginine decarboxylase	4.1.1.19	E.coli	10420650
arginine decarboxylase	4.1.1.19	E.coli	11029703
arginine decarboxylase	4.1.1.19	E.coli	11540835
arginine decarboxylase	4.1.1.19	E.coli	11576438
arginine decarboxylase	4.1.1.19	E.coli	11903975
arginine decarboxylase	4.1.1.19	E.coli	12060267
arginine decarboxylase	4.1.1.19	E.coli	15032880
arginine decarboxylase	4.1.1.19	E.coli	15092366
arginine decarboxylase	4.1.1.19	E.coli	15120115
arginine decarboxylase	4.1.1.19	E.coli	15527979
arginine decarboxylase	4.1.1.19	E.coli	15733873
arginine decarboxylase	4.1.1.19	E.coli	16662219
arginine decarboxylase	4.1.1.19	E.coli	16663805
arginine decarboxylase	4.1.1.19	E.coli	16769152
arginine decarboxylase	4.1.1.19	E.coli	3931079
arginine decarboxylase	4.1.1.19	E.coli	9230111
arginine decarboxylase	4.1.1.19	E.coli	9624108
phosphoenolpyruvate carboxykinase (ATP)	4.1.1.49	E.coli	10230645
phosphoenolpyruvate carboxykinase (ATP)	4.1.1.49	E.coli	10409689
phosphoenolpyruvate carboxykinase (ATP)	4.1.1.49	E.coli	10416947
phosphoenolpyruvate carboxykinase (ATP)	4.1.1.49	E.coli	10508111
phosphoenolpyruvate carboxykinase (ATP)	4.1.1.49	E.coli	10805165
phosphoenolpyruvate carboxykinase (ATP)	4.1.1.49	E.coli	10909974
phosphoenolpyruvate carboxykinase (ATP)	4.1.1.49	E.coli	11038056
phosphoenolpyruvate carboxykinase (ATP)	4.1.1.49	E.coli	11553511
phosphoenolpyruvate carboxykinase (ATP)	4.1.1.49	E.coli	11557984
phosphoenolpyruvate carboxykinase (ATP)	4.1.1.49	E.coli	11677594
phosphoenolpyruvate carboxykinase (ATP)	4.1.1.49	E.coli	11679417
phosphoenolpyruvate carboxykinase (ATP)	4.1.1.49	E.coli	11700062
phosphoenolpyruvate carboxykinase (ATP)	4.1.1.49	E.coli	11728630
phosphoenolpyruvate carboxykinase (ATP)	4.1.1.49	E.coli	11741859
phosphoenolpyruvate carboxykinase (ATP)	4.1.1.49	E.coli	12020659
phosphoenolpyruvate carboxykinase (ATP)	4.1.1.49	E.coli	12089355
phosphoenolpyruvate carboxykinase (ATP)	4.1.1.49	E.coli	12455594
phosphoenolpyruvate carboxykinase (ATP)	4.1.1.49	E.coli	12485530
phosphoenolpyruvate carboxykinase (ATP)	4.1.1.49	E.coli	12644461
phosphoenolpyruvate carboxykinase (ATP)	4.1.1.49	E.coli	12646246
phosphoenolpyruvate carboxykinase (ATP)	4.1.1.49	E.coli	12757760
phosphoenolpyruvate carboxykinase (ATP)	4.1.1.49	E.coli	12865425
phosphoenolpyruvate carboxykinase (ATP)	4.1.1.49	E.coli	1315012

phosphoenolpyruvate carboxykinase (ATP)	4.1.1.49	E.coli	1398916
phosphoenolpyruvate carboxykinase (ATP)	4.1.1.49	E.coli	14739071
phosphoenolpyruvate carboxykinase (ATP)	4.1.1.49	E.coli	14739078
phosphoenolpyruvate carboxykinase (ATP)	4.1.1.49	E.coli	14739255
phosphoenolpyruvate carboxykinase (ATP)	4.1.1.49	E.coli	1510582
phosphoenolpyruvate carboxykinase (ATP)	4.1.1.49	E.coli	15265774
phosphoenolpyruvate carboxykinase (ATP)	4.1.1.49	E.coli	15733733
phosphoenolpyruvate carboxykinase (ATP)	4.1.1.49	E.coli	15907483
phosphoenolpyruvate carboxykinase (ATP)	4.1.1.49	E.coli	15983413
phosphoenolpyruvate carboxykinase (ATP)	4.1.1.49	E.coli	16236252
phosphoenolpyruvate carboxykinase (ATP)	4.1.1.49	E.coli	16249187
phosphoenolpyruvate carboxykinase (ATP)	4.1.1.49	E.coli	16324924
phosphoenolpyruvate carboxykinase (ATP)	4.1.1.49	E.coli	16330542
phosphoenolpyruvate carboxykinase (ATP)	4.1.1.49	E.coli	16458327
phosphoenolpyruvate carboxykinase (ATP)	4.1.1.49	E.coli	16503364
phosphoenolpyruvate carboxykinase (ATP)	4.1.1.49	E.coli	1653277
phosphoenolpyruvate carboxykinase (ATP)	4.1.1.49	E.coli	16819824
phosphoenolpyruvate carboxykinase (ATP)	4.1.1.49	E.coli	16941667
phosphoenolpyruvate carboxykinase (ATP)	4.1.1.49	E.coli	17097062
phosphoenolpyruvate carboxykinase (ATP)	4.1.1.49	E.coli	1720862
phosphoenolpyruvate carboxykinase (ATP)	4.1.1.49	E.coli	1733721
phosphoenolpyruvate carboxykinase (ATP)	4.1.1.49	E.coli	176867
phosphoenolpyruvate carboxykinase (ATP)	4.1.1.49	E.coli	1820332
phosphoenolpyruvate carboxykinase (ATP)	4.1.1.49	E.coli	1848696
phosphoenolpyruvate carboxykinase (ATP)	4.1.1.49	E.coli	2265217
phosphoenolpyruvate carboxykinase (ATP)	4.1.1.49	E.coli	25836
phosphoenolpyruvate carboxykinase (ATP)	4.1.1.49	E.coli	2919162
phosphoenolpyruvate carboxykinase (ATP)	4.1.1.49	E.coli	3023262
phosphoenolpyruvate carboxykinase (ATP)	4.1.1.49	E.coli	3059968
phosphoenolpyruvate carboxykinase (ATP)	4.1.1.49	E.coli	3068502
phosphoenolpyruvate carboxykinase (ATP)	4.1.1.49	E.coli	3203688
phosphoenolpyruvate carboxykinase (ATP)	4.1.1.49	E.coli	329666
phosphoenolpyruvate carboxykinase (ATP)	4.1.1.49	E.coli	3542066
phosphoenolpyruvate carboxykinase (ATP)	4.1.1.49	E.coli	3947691
phosphoenolpyruvate carboxykinase (ATP)	4.1.1.49	E.coli	3957187
phosphoenolpyruvate carboxykinase (ATP)	4.1.1.49	E.coli	422559
phosphoenolpyruvate carboxykinase (ATP)	4.1.1.49	E.coli	582464
phosphoenolpyruvate carboxykinase (ATP)	4.1.1.49	E.coli	6440018
phosphoenolpyruvate carboxykinase (ATP)	4.1.1.49	E.coli	6583707
phosphoenolpyruvate carboxykinase (ATP)	4.1.1.49	E.coli	6917781
phosphoenolpyruvate carboxykinase (ATP)	4.1.1.49	E.coli	7587654
phosphoenolpyruvate carboxykinase (ATP)	4.1.1.49	E.coli	7854322
phosphoenolpyruvate carboxykinase (ATP)	4.1.1.49	E.coli	7980440

phosphoenolpyruvate carboxykinase (ATP)	4.1.1.49	E.coli	8074220
phosphoenolpyruvate carboxykinase (ATP)	4.1.1.49	E.coli	8375031
phosphoenolpyruvate carboxykinase (ATP)	4.1.1.49	E.coli	8384841
phosphoenolpyruvate carboxykinase (ATP)	4.1.1.49	E.coli	8432541
phosphoenolpyruvate carboxykinase (ATP)	4.1.1.49	E.coli	8449898
phosphoenolpyruvate carboxykinase (ATP)	4.1.1.49	E.coli	8557765
phosphoenolpyruvate carboxykinase (ATP)	4.1.1.49	E.coli	8567635
phosphoenolpyruvate carboxykinase (ATP)	4.1.1.49	E.coli	8636258
phosphoenolpyruvate carboxykinase (ATP)	4.1.1.49	E.coli	9214454
phosphoenolpyruvate carboxykinase (ATP)	4.1.1.49	E.coli	9242918
phosphoenolpyruvate carboxykinase (ATP)	4.1.1.49	E.coli	9452431
phosphoenolpyruvate carboxykinase (ATP)	4.1.1.49	E.coli	9473304
phosphoenolpyruvate carboxykinase (ATP)	4.1.1.49	E.coli	9530152
phosphoenolpyruvate carboxykinase (ATP)	4.1.1.49	E.coli	9762796
phosphoenolpyruvate carboxykinase (ATP)	4.1.1.49	E.coli	9854182
adenosylmethionine decarboxylase	4.1.1.50	E.coli	10216947
adenosylmethionine decarboxylase	4.1.1.50	E.coli	10378277
adenosylmethionine decarboxylase	4.1.1.50	E.coli	10413038
adenosylmethionine decarboxylase	4.1.1.50	E.coli	10467042
adenosylmethionine decarboxylase	4.1.1.50	E.coli	10713131
adenosylmethionine decarboxylase	4.1.1.50	E.coli	10949915
adenosylmethionine decarboxylase	4.1.1.50	E.coli	11076965
adenosylmethionine decarboxylase	4.1.1.50	E.coli	11085920
adenosylmethionine decarboxylase	4.1.1.50	E.coli	11348531
adenosylmethionine decarboxylase	4.1.1.50	E.coli	11390378
adenosylmethionine decarboxylase	4.1.1.50	E.coli	11526206
adenosylmethionine decarboxylase	4.1.1.50	E.coli	11583148
adenosylmethionine decarboxylase	4.1.1.50	E.coli	11923270
adenosylmethionine decarboxylase	4.1.1.50	E.coli	12674502
adenosylmethionine decarboxylase	4.1.1.50	E.coli	14618239
adenosylmethionine decarboxylase	4.1.1.50	E.coli	15150268
adenosylmethionine decarboxylase	4.1.1.50	E.coli	15821146
adenosylmethionine decarboxylase	4.1.1.50	E.coli	16372273
adenosylmethionine decarboxylase	4.1.1.50	E.coli	16423999
adenosylmethionine decarboxylase	4.1.1.50	E.coli	16515461
adenosylmethionine decarboxylase	4.1.1.50	E.coli	16642382
adenosylmethionine decarboxylase	4.1.1.50	E.coli	16941339
adenosylmethionine decarboxylase	4.1.1.50	E.coli	2013278
adenosylmethionine decarboxylase	4.1.1.50	E.coli	2209170
adenosylmethionine decarboxylase	4.1.1.50	E.coli	2775206
adenosylmethionine decarboxylase	4.1.1.50	E.coli	3250232
adenosylmethionine decarboxylase	4.1.1.50	E.coli	4062886
adenosylmethionine decarboxylase	4.1.1.50	E.coli	7789170

adenosylmethionine decarboxylase	4.1.1.50	E.coli	7945201
adenosylmethionine decarboxylase	4.1.1.50	E.coli	8142949
adenosylmethionine decarboxylase	4.1.1.50	E.coli	8178573
adenosylmethionine decarboxylase	4.1.1.50	E.coli	8198469
adenosylmethionine decarboxylase	4.1.1.50	E.coli	8353934
adenosylmethionine decarboxylase	4.1.1.50	E.coli	8760129
adenosylmethionine decarboxylase	4.1.1.50	E.coli	8814137
adenosylmethionine decarboxylase	4.1.1.50	E.coli	8973561
adenosylmethionine decarboxylase	4.1.1.50	E.coli	9435790
adenosylmethionine decarboxylase	4.1.1.50	E.coli	9841864
adenosylmethionine decarboxylase	4.1.1.50	E.coli	9879883
fructose-bisphosphate aldolase	4.1.2.13	E.coli	10498814
fructose-bisphosphate aldolase	4.1.2.13	E.coli	12020659
fructose-bisphosphate aldolase	4.1.2.13	E.coli	12876349
fructose-bisphosphate aldolase	4.1.2.13	E.coli	15142555
fructose-bisphosphate aldolase	4.1.2.13	E.coli	15869466
fructose-bisphosphate aldolase	4.1.2.13	E.coli	16502329
fructose-bisphosphate aldolase	4.1.2.13	E.coli	1658253
fructose-bisphosphate aldolase	4.1.2.13	E.coli	1894606
fructose-bisphosphate aldolase	4.1.2.13	E.coli	6440018
fructose-bisphosphate aldolase	4.1.2.13	E.coli	678439
fructose-bisphosphate aldolase	4.1.2.13	E.coli	7388140
fructose-bisphosphate aldolase	4.1.2.13	E.coli	7786768
fructose-bisphosphate aldolase	4.1.2.13	E.coli	8015399
fructose-bisphosphate aldolase	4.1.2.13	E.coli	8913875
fructose-bisphosphate aldolase	4.1.2.13	E.coli	9163906
fructose-bisphosphate aldolase	4.1.2.13	E.coli	9473304
isocitrate lyase	4.1.3.1	E.coli	10439413
isocitrate lyase	4.1.3.1	E.coli	10805817
isocitrate lyase	4.1.3.1	E.coli	11038056
isocitrate lyase	4.1.3.1	E.coli	11526312
isocitrate lyase	4.1.3.1	E.coli	11888209
isocitrate lyase	4.1.3.1	E.coli	14558475
isocitrate lyase	4.1.3.1	E.coli	15299923
isocitrate lyase	4.1.3.1	E.coli	16603391
isocitrate lyase	4.1.3.1	E.coli	16856937
isocitrate lyase	4.1.3.1	E.coli	2209599
isocitrate lyase	4.1.3.1	E.coli	2361956
isocitrate lyase	4.1.3.1	E.coli	238950
isocitrate lyase	4.1.3.1	E.coli	6389540
isocitrate lyase	4.1.3.1	E.coli	9738442
anthranilate synthase	4.1.3.27	E.coli	10978548
anthranilate synthase	4.1.3.27	E.coli	15563620

anthranilate synthase	4.1.3.27	E.coli	15645305
anthranilate synthase	4.1.3.27	E.coli	15980261
anthranilate synthase	4.1.3.27	E.coli	16040654
anthranilate synthase	4.1.3.27	E.coli	3311153
anthranilate synthase	4.1.3.27	E.coli	378978
anthranilate synthase	4.1.3.27	E.coli	9299222
carbonate dehydratase	4.2.1.1	E.coli	16051345
carbonate dehydratase	4.2.1.1	E.coli	16310354
carbonate dehydratase	4.2.1.1	E.coli	1908243
carbonate dehydratase	4.2.1.1	E.coli	2117006
carbonate dehydratase	4.2.1.1	E.coli	3128444
carbonate dehydratase	4.2.1.1	E.coli	6408083
carbonate dehydratase	4.2.1.1	E.coli	6772280
carbonate dehydratase	4.2.1.1	E.coli	6776264
carbonate dehydratase	4.2.1.1	E.coli	6791257
carbonate dehydratase	4.2.1.1	E.coli	7899732
carbonate dehydratase	4.2.1.1	E.coli	9186493
carbonate dehydratase	4.2.1.1	E.coli	9486145
carbonate dehydratase	4.2.1.1	E.coli	9882455
phosphopyruvate hydratase	4.2.1.11	E.coli	15459207
phosphopyruvate hydratase	4.2.1.11	E.coli	17437631
phosphopyruvate hydratase	4.2.1.11	E.coli	8651685
tryptophan synthase	4.2.1.20	E.coli	10433729
tryptophan synthase	4.2.1.20	E.coli	10769125
tryptophan synthase	4.2.1.20	E.coli	11756454
tryptophan synthase	4.2.1.20	E.coli	15542062
tryptophan synthase	4.2.1.20	E.coli	2183877
tryptophan synthase	4.2.1.20	E.coli	2185841
tryptophan synthase	4.2.1.20	E.coli	3297161
tryptophan synthase	4.2.1.20	E.coli	8193152
porphobilinogen synthase	4.2.1.24	E.coli	10634305
porphobilinogen synthase	4.2.1.24	E.coli	10787385
porphobilinogen synthase	4.2.1.24	E.coli	15141099
porphobilinogen synthase	4.2.1.24	E.coli	15259603
porphobilinogen synthase	4.2.1.24	E.coli	1526942
porphobilinogen synthase	4.2.1.24	E.coli	16819823
porphobilinogen synthase	4.2.1.24	E.coli	1959865
porphobilinogen synthase	4.2.1.24	E.coli	2050126
porphobilinogen synthase	4.2.1.24	E.coli	2317819
porphobilinogen synthase	4.2.1.24	E.coli	3009001
porphobilinogen synthase	4.2.1.24	E.coli	3755290
porphobilinogen synthase	4.2.1.24	E.coli	3840094
porphobilinogen synthase	4.2.1.24	E.coli	3966797

porphobilinogen synthase	4.2.1.24	E.coli	4265023
porphobilinogen synthase	4.2.1.24	E.coli	6547609
porphobilinogen synthase	4.2.1.24	E.coli	6688350
porphobilinogen synthase	4.2.1.24	E.coli	6873612
porphobilinogen synthase	4.2.1.24	E.coli	7436670
aconitate hydratase	4.2.1.3	E.coli	11295257
aconitate hydratase	4.2.1.3	E.coli	11329290
aconitate hydratase	4.2.1.3	E.coli	15149735
aconitate hydratase	4.2.1.3	E.coli	15543948
aconitate hydratase	4.2.1.3	E.coli	16094633
aconitate hydratase	4.2.1.3	E.coli	16201454
aconitate hydratase	4.2.1.3	E.coli	7589784
aconitate hydratase	4.2.1.3	E.coli	8115279
prephenate dehydratase	4.2.1.51	E.coli	11326337
prephenate dehydratase	4.2.1.51	E.coli	14749915
prephenate dehydratase	4.2.1.51	E.coli	14749916
prephenate dehydratase	4.2.1.51	E.coli	14749917
prephenate dehydratase	4.2.1.51	E.coli	14749918
prephenate dehydratase	4.2.1.51	E.coli	15753077
prephenate dehydratase	4.2.1.51	E.coli	6150022
6-pyruvoyltetrahydropterin synthase	4.2.3.12	E.coli	11022034
6-pyruvoyltetrahydropterin synthase	4.2.3.12	E.coli	11744995
6-pyruvoyltetrahydropterin synthase	4.2.3.12	E.coli	11778454
6-pyruvoyltetrahydropterin synthase	4.2.3.12	E.coli	7545485
6-pyruvoyltetrahydropterin synthase	4.2.3.12	E.coli	8307017
6-pyruvoyltetrahydropterin synthase	4.2.3.12	E.coli	9788822
UDP-N-acetylglucosamine 2-epimerase	5.1.3.14	E.coli	10334995
UDP-N-acetylglucosamine 2-epimerase	5.1.3.14	E.coli	11326336
UDP-N-acetylglucosamine 2-epimerase	5.1.3.14	E.coli	11956597
UDP-N-acetylglucosamine 2-epimerase	5.1.3.14	E.coli	12927803
UDP-N-acetylglucosamine 2-epimerase	5.1.3.14	E.coli	15135418
UDP-N-acetylglucosamine 2-epimerase	5.1.3.14	E.coli	17565386
UDP-N-acetylglucosamine 2-epimerase	5.1.3.14	E.coli	3780977
UDP-N-acetylglucosamine 2-epimerase	5.1.3.14	E.coli	8439453
UDP-N-acetylglucosamine 2-epimerase	5.1.3.14	E.coli	9305888
UDP-glucose 4-epimerase	5.1.3.2	E.coli	1149741
phosphoglycerate mutase	5.4.2.1	E.coli	15181008
phosphoglycerate mutase	5.4.2.1	E.coli	17204863
phosphoglycerate mutase	5.4.2.1	E.coli	2830218
phosphoglucomutase	5.4.2.2	E.coli	11102370
phosphoglucomutase	5.4.2.2	E.coli	1149741
phosphoglucomutase	5.4.2.2	E.coli	12026175
phosphoglucomutase	5.4.2.2	E.coli	12791685

phosphoglucomutase	5.4.2.2	E.coli	15378030
phosphoglucomutase	5.4.2.2	E.coli	15996095
phosphoglucomutase	5.4.2.2	E.coli	16046289
isochorismate synthase	5.4.4.2	E.coli	12624097
isochorismate synthase	5.4.4.2	E.coli	12872482
isochorismate synthase	5.4.4.2	E.coli	16085832
isochorismate synthase	5.4.4.2	E.coli	1913340
isochorismate synthase	5.4.4.2	E.coli	2154945
acetate---CoA ligase	6.2.1.1	E.coli	15236963
acetate---CoA ligase	6.2.1.1	E.coli	15899897
acetate---CoA ligase	6.2.1.1	E.coli	2902801
acetate---CoA ligase	6.2.1.1	E.coli	4149947
acetate---CoA ligase	6.2.1.1	E.coli	8218953
long-chain-fatty-acid---CoA ligase	6.2.1.3	E.coli	11375393
long-chain-fatty-acid---CoA ligase	6.2.1.3	E.coli	1769731
succinate---CoA ligase (ADP-forming)	6.2.1.5	E.coli	16101500
succinate---CoA ligase (ADP-forming)	6.2.1.5	E.coli	1986797
succinate---CoA ligase (ADP-forming)	6.2.1.5	E.coli	3108130
succinate---CoA ligase (ADP-forming)	6.2.1.5	E.coli	3746465
succinate---CoA ligase (ADP-forming)	6.2.1.5	E.coli	6109001
succinate---CoA ligase (ADP-forming)	6.2.1.5	E.coli	7017725
succinate---CoA ligase (ADP-forming)	6.2.1.5	E.coli	7062046
succinate---CoA ligase (ADP-forming)	6.2.1.5	E.coli	7783627
glutamate---ammonia ligase	6.3.1.2	E.coli	10092169
glutamate---ammonia ligase	6.3.1.2	E.coli	10383611
glutamate---ammonia ligase	6.3.1.2	E.coli	10440891
glutamate---ammonia ligase	6.3.1.2	E.coli	10664131
glutamate---ammonia ligase	6.3.1.2	E.coli	11069692
glutamate---ammonia ligase	6.3.1.2	E.coli	11413247
glutamate---ammonia ligase	6.3.1.2	E.coli	11939529
glutamate---ammonia ligase	6.3.1.2	E.coli	12232192
glutamate---ammonia ligase	6.3.1.2	E.coli	12517141
glutamate---ammonia ligase	6.3.1.2	E.coli	12552916
glutamate---ammonia ligase	6.3.1.2	E.coli	14714472
glutamate---ammonia ligase	6.3.1.2	E.coli	14723991
glutamate---ammonia ligase	6.3.1.2	E.coli	15130478
glutamate---ammonia ligase	6.3.1.2	E.coli	15489445
glutamate---ammonia ligase	6.3.1.2	E.coli	15581577
glutamate---ammonia ligase	6.3.1.2	E.coli	1612427
glutamate---ammonia ligase	6.3.1.2	E.coli	16687472
glutamate---ammonia ligase	6.3.1.2	E.coli	16829528
glutamate---ammonia ligase	6.3.1.2	E.coli	16946267
glutamate---ammonia ligase	6.3.1.2	E.coli	2434618

glutamate---ammonia ligase	6.3.1.2	E.coli	26663
glutamate---ammonia ligase	6.3.1.2	E.coli	6118373
glutamate---ammonia ligase	6.3.1.2	E.coli	9624228
glutamate---cysteine ligase	6.3.2.2	E.coli	10215022
glutamate---cysteine ligase	6.3.2.2	E.coli	10218647
glutamate---cysteine ligase	6.3.2.2	E.coli	10349842
glutamate---cysteine ligase	6.3.2.2	E.coli	10385608
glutamate---cysteine ligase	6.3.2.2	E.coli	10385658
glutamate---cysteine ligase	6.3.2.2	E.coli	10399958
glutamate---cysteine ligase	6.3.2.2	E.coli	10439045
glutamate---cysteine ligase	6.3.2.2	E.coli	10441483
glutamate---cysteine ligase	6.3.2.2	E.coli	10486302
glutamate---cysteine ligase	6.3.2.2	E.coli	10515588
glutamate---cysteine ligase	6.3.2.2	E.coli	10518117
glutamate---cysteine ligase	6.3.2.2	E.coli	10544055
glutamate---cysteine ligase	6.3.2.2	E.coli	10544272
glutamate---cysteine ligase	6.3.2.2	E.coli	10590319
glutamate---cysteine ligase	6.3.2.2	E.coli	10593589
glutamate---cysteine ligase	6.3.2.2	E.coli	10594104
glutamate---cysteine ligase	6.3.2.2	E.coli	10600876
glutamate---cysteine ligase	6.3.2.2	E.coli	10623879
glutamate---cysteine ligase	6.3.2.2	E.coli	10644053
glutamate---cysteine ligase	6.3.2.2	E.coli	10674357
glutamate---cysteine ligase	6.3.2.2	E.coli	10677377
glutamate---cysteine ligase	6.3.2.2	E.coli	10702364
glutamate---cysteine ligase	6.3.2.2	E.coli	10719238
glutamate---cysteine ligase	6.3.2.2	E.coli	10733945
glutamate---cysteine ligase	6.3.2.2	E.coli	10748080
glutamate---cysteine ligase	6.3.2.2	E.coli	10773686
glutamate---cysteine ligase	6.3.2.2	E.coli	10777712
glutamate---cysteine ligase	6.3.2.2	E.coli	10802223
glutamate---cysteine ligase	6.3.2.2	E.coli	10928075
glutamate---cysteine ligase	6.3.2.2	E.coli	10960449
glutamate---cysteine ligase	6.3.2.2	E.coli	11007940
glutamate---cysteine ligase	6.3.2.2	E.coli	11025451
glutamate---cysteine ligase	6.3.2.2	E.coli	11028671
glutamate---cysteine ligase	6.3.2.2	E.coli	11032771
glutamate---cysteine ligase	6.3.2.2	E.coli	11097862
glutamate---cysteine ligase	6.3.2.2	E.coli	11133045
glutamate---cysteine ligase	6.3.2.2	E.coli	11157875
glutamate---cysteine ligase	6.3.2.2	E.coli	11163433
glutamate---cysteine ligase	6.3.2.2	E.coli	11181934
glutamate---cysteine ligase	6.3.2.2	E.coli	11233143

glutamate---cysteine ligase	6.3.2.2	E.coli	11306445
glutamate---cysteine ligase	6.3.2.2	E.coli	11339815
glutamate---cysteine ligase	6.3.2.2	E.coli	11352989
glutamate---cysteine ligase	6.3.2.2	E.coli	11353135
glutamate---cysteine ligase	6.3.2.2	E.coli	11500053
glutamate---cysteine ligase	6.3.2.2	E.coli	11560771
glutamate---cysteine ligase	6.3.2.2	E.coli	11565956
glutamate---cysteine ligase	6.3.2.2	E.coli	11687904
glutamate---cysteine ligase	6.3.2.2	E.coli	11705692
glutamate---cysteine ligase	6.3.2.2	E.coli	11780957
glutamate---cysteine ligase	6.3.2.2	E.coli	11781188
glutamate---cysteine ligase	6.3.2.2	E.coli	11790356
glutamate---cysteine ligase	6.3.2.2	E.coli	11812649
glutamate---cysteine ligase	6.3.2.2	E.coli	11820781
glutamate---cysteine ligase	6.3.2.2	E.coli	11841806
glutamate---cysteine ligase	6.3.2.2	E.coli	11849402
glutamate---cysteine ligase	6.3.2.2	E.coli	11876501
glutamate---cysteine ligase	6.3.2.2	E.coli	11970852
glutamate---cysteine ligase	6.3.2.2	E.coli	12079521
glutamate---cysteine ligase	6.3.2.2	E.coli	12111865
glutamate---cysteine ligase	6.3.2.2	E.coli	12147223
glutamate---cysteine ligase	6.3.2.2	E.coli	12196927
glutamate---cysteine ligase	6.3.2.2	E.coli	12200125
glutamate---cysteine ligase	6.3.2.2	E.coli	12204877
glutamate---cysteine ligase	6.3.2.2	E.coli	12433058
glutamate---cysteine ligase	6.3.2.2	E.coli	12448821
glutamate---cysteine ligase	6.3.2.2	E.coli	12452384
glutamate---cysteine ligase	6.3.2.2	E.coli	12535742
glutamate---cysteine ligase	6.3.2.2	E.coli	12594957
glutamate---cysteine ligase	6.3.2.2	E.coli	12601050
glutamate---cysteine ligase	6.3.2.2	E.coli	12607907
glutamate---cysteine ligase	6.3.2.2	E.coli	12628495
glutamate---cysteine ligase	6.3.2.2	E.coli	12637989
glutamate---cysteine ligase	6.3.2.2	E.coli	12814619
glutamate---cysteine ligase	6.3.2.2	E.coli	12882455
glutamate---cysteine ligase	6.3.2.2	E.coli	12913252
glutamate---cysteine ligase	6.3.2.2	E.coli	1350904
glutamate---cysteine ligase	6.3.2.2	E.coli	1351382
glutamate---cysteine ligase	6.3.2.2	E.coli	1353765
glutamate---cysteine ligase	6.3.2.2	E.coli	1355406
glutamate---cysteine ligase	6.3.2.2	E.coli	1362226
glutamate---cysteine ligase	6.3.2.2	E.coli	13679058
glutamate---cysteine ligase	6.3.2.2	E.coli	14500406

glutamate---cysteine ligase	6.3.2.2	E.coli	14514673
glutamate---cysteine ligase	6.3.2.2	E.coli	14744626
glutamate---cysteine ligase	6.3.2.2	E.coli	14962359
glutamate---cysteine ligase	6.3.2.2	E.coli	15020643
glutamate---cysteine ligase	6.3.2.2	E.coli	15050748
glutamate---cysteine ligase	6.3.2.2	E.coli	15257546
glutamate---cysteine ligase	6.3.2.2	E.coli	15314090
glutamate---cysteine ligase	6.3.2.2	E.coli	15374419
glutamate---cysteine ligase	6.3.2.2	E.coli	15451066
glutamate---cysteine ligase	6.3.2.2	E.coli	15477603
glutamate---cysteine ligase	6.3.2.2	E.coli	15509664
glutamate---cysteine ligase	6.3.2.2	E.coli	15878398
glutamate---cysteine ligase	6.3.2.2	E.coli	15946948
glutamate---cysteine ligase	6.3.2.2	E.coli	16032782
glutamate---cysteine ligase	6.3.2.2	E.coli	16081425
glutamate---cysteine ligase	6.3.2.2	E.coli	16162662
glutamate---cysteine ligase	6.3.2.2	E.coli	16183645
glutamate---cysteine ligase	6.3.2.2	E.coli	16549430
glutamate---cysteine ligase	6.3.2.2	E.coli	16566126
glutamate---cysteine ligase	6.3.2.2	E.coli	1678010
glutamate---cysteine ligase	6.3.2.2	E.coli	16781460
glutamate---cysteine ligase	6.3.2.2	E.coli	16949561
glutamate---cysteine ligase	6.3.2.2	E.coli	16960387
glutamate---cysteine ligase	6.3.2.2	E.coli	17144898
glutamate---cysteine ligase	6.3.2.2	E.coli	1784629
glutamate---cysteine ligase	6.3.2.2	E.coli	1970723
glutamate---cysteine ligase	6.3.2.2	E.coli	1997009
glutamate---cysteine ligase	6.3.2.2	E.coli	2572174
glutamate---cysteine ligase	6.3.2.2	E.coli	2574245
glutamate---cysteine ligase	6.3.2.2	E.coli	2879531
glutamate---cysteine ligase	6.3.2.2	E.coli	2897858
glutamate---cysteine ligase	6.3.2.2	E.coli	2901982
glutamate---cysteine ligase	6.3.2.2	E.coli	3621155
glutamate---cysteine ligase	6.3.2.2	E.coli	6897891
glutamate---cysteine ligase	6.3.2.2	E.coli	7351635
glutamate---cysteine ligase	6.3.2.2	E.coli	7503776
glutamate---cysteine ligase	6.3.2.2	E.coli	7568279
glutamate---cysteine ligase	6.3.2.2	E.coli	7570642
glutamate---cysteine ligase	6.3.2.2	E.coli	7585502
glutamate---cysteine ligase	6.3.2.2	E.coli	7622006
glutamate---cysteine ligase	6.3.2.2	E.coli	7651354
glutamate---cysteine ligase	6.3.2.2	E.coli	7768207
glutamate---cysteine ligase	6.3.2.2	E.coli	7901332

glutamate---cysteine ligase	6.3.2.2	E.coli	7908245
glutamate---cysteine ligase	6.3.2.2	E.coli	7910419
glutamate---cysteine ligase	6.3.2.2	E.coli	7929374
glutamate---cysteine ligase	6.3.2.2	E.coli	7955076
glutamate---cysteine ligase	6.3.2.2	E.coli	7969079
glutamate---cysteine ligase	6.3.2.2	E.coli	8001239
glutamate---cysteine ligase	6.3.2.2	E.coli	8065332
glutamate---cysteine ligase	6.3.2.2	E.coli	8101766
glutamate---cysteine ligase	6.3.2.2	E.coli	8106072
glutamate---cysteine ligase	6.3.2.2	E.coli	8120650
glutamate---cysteine ligase	6.3.2.2	E.coli	8538700
glutamate---cysteine ligase	6.3.2.2	E.coli	8582653
glutamate---cysteine ligase	6.3.2.2	E.coli	8648118
glutamate---cysteine ligase	6.3.2.2	E.coli	8661240
glutamate---cysteine ligase	6.3.2.2	E.coli	8751598
glutamate---cysteine ligase	6.3.2.2	E.coli	8781554
glutamate---cysteine ligase	6.3.2.2	E.coli	8792848
glutamate---cysteine ligase	6.3.2.2	E.coli	8806884
glutamate---cysteine ligase	6.3.2.2	E.coli	8843715
glutamate---cysteine ligase	6.3.2.2	E.coli	8917676
glutamate---cysteine ligase	6.3.2.2	E.coli	8930687
glutamate---cysteine ligase	6.3.2.2	E.coli	8947504
glutamate---cysteine ligase	6.3.2.2	E.coli	8973794
glutamate---cysteine ligase	6.3.2.2	E.coli	8981036
glutamate---cysteine ligase	6.3.2.2	E.coli	8995480
glutamate---cysteine ligase	6.3.2.2	E.coli	9029270
glutamate---cysteine ligase	6.3.2.2	E.coli	9054446
glutamate---cysteine ligase	6.3.2.2	E.coli	9063478
glutamate---cysteine ligase	6.3.2.2	E.coli	9093011
glutamate---cysteine ligase	6.3.2.2	E.coli	9119067
glutamate---cysteine ligase	6.3.2.2	E.coli	9157984
glutamate---cysteine ligase	6.3.2.2	E.coli	9163779
glutamate---cysteine ligase	6.3.2.2	E.coli	9185621
glutamate---cysteine ligase	6.3.2.2	E.coli	9214623
glutamate---cysteine ligase	6.3.2.2	E.coli	9259355
glutamate---cysteine ligase	6.3.2.2	E.coli	9268987
glutamate---cysteine ligase	6.3.2.2	E.coli	9288403
glutamate---cysteine ligase	6.3.2.2	E.coli	9311606
glutamate---cysteine ligase	6.3.2.2	E.coli	9374111
glutamate---cysteine ligase	6.3.2.2	E.coli	9389600
glutamate---cysteine ligase	6.3.2.2	E.coli	9393741
glutamate---cysteine ligase	6.3.2.2	E.coli	9425930
glutamate---cysteine ligase	6.3.2.2	E.coli	9582278

glutamate---cysteine ligase	6.3.2.2	E.coli	9614065
glutamate---cysteine ligase	6.3.2.2	E.coli	9626582
glutamate---cysteine ligase	6.3.2.2	E.coli	9647756
glutamate---cysteine ligase	6.3.2.2	E.coli	9679558
glutamate---cysteine ligase	6.3.2.2	E.coli	9703946
glutamate---cysteine ligase	6.3.2.2	E.coli	9729439
glutamate---cysteine ligase	6.3.2.2	E.coli	9750167
glutamate---cysteine ligase	6.3.2.2	E.coli	9756861
glutamate---cysteine ligase	6.3.2.2	E.coli	9762423
glutamate---cysteine ligase	6.3.2.2	E.coli	9875552
glutamate---cysteine ligase	6.3.2.2	E.coli	9895302
glutathione synthase	6.3.2.3	E.coli	10964706
glutathione synthase	6.3.2.3	E.coli	11708780
glutathione synthase	6.3.2.3	E.coli	9880348
phosphoribosylaminoimidazolesuccinocarboxamide synthase	6.3.2.6	E.coli	701284
CTP synthase	6.3.4.2	E.coli	12678497
CTP synthase	6.3.4.2	E.coli	16820675
CTP synthase	6.3.4.2	E.coli	17463002
CTP synthase	6.3.4.2	E.coli	17681942
CTP synthase	6.3.4.2	E.coli	2787169
argininosuccinate synthase	6.3.4.5	E.coli	10709858
argininosuccinate synthase	6.3.4.5	E.coli	1122920
argininosuccinate synthase	6.3.4.5	E.coli	11556547
argininosuccinate synthase	6.3.4.5	E.coli	12618329
argininosuccinate synthase	6.3.4.5	E.coli	12672181
argininosuccinate synthase	6.3.4.5	E.coli	1372742
argininosuccinate synthase	6.3.4.5	E.coli	14571701
argininosuccinate synthase	6.3.4.5	E.coli	15588718
argininosuccinate synthase	6.3.4.5	E.coli	16085056
argininosuccinate synthase	6.3.4.5	E.coli	16787144
argininosuccinate synthase	6.3.4.5	E.coli	558104
argininosuccinate synthase	6.3.4.5	E.coli	845694
argininosuccinate synthase	6.3.4.5	E.coli	8616812
argininosuccinate synthase	6.3.4.5	E.coli	8798625
argininosuccinate synthase	6.3.4.5	E.coli	9096605
argininosuccinate synthase	6.3.4.5	E.coli	9176259
argininosuccinate synthase	6.3.4.5	E.coli	9211993
argininosuccinate synthase	6.3.4.5	E.coli	9252090
carbamoyl-phosphate synthase (glutamine-hydrolysing)	6.3.5.5	E.coli	10659854
carbamoyl-phosphate synthase (glutamine-hydrolysing)	6.3.5.5	E.coli	10736367

carbamoyl-phosphate synthase (glutamine-hydrolysing)	6.3.5.5	E.coli	11441057
carbamoyl-phosphate synthase (glutamine-hydrolysing)	6.3.5.5	E.coli	11872754
carbamoyl-phosphate synthase (glutamine-hydrolysing)	6.3.5.5	E.coli	11956684
carbamoyl-phosphate synthase (glutamine-hydrolysing)	6.3.5.5	E.coli	12678497
carbamoyl-phosphate synthase (glutamine-hydrolysing)	6.3.5.5	E.coli	15326225
carbamoyl-phosphate synthase (glutamine-hydrolysing)	6.3.5.5	E.coli	15453495
carbamoyl-phosphate synthase (glutamine-hydrolysing)	6.3.5.5	E.coli	4018077
carbamoyl-phosphate synthase (glutamine-hydrolysing)	6.3.5.5	E.coli	6115855
carbamoyl-phosphate synthase (glutamine-hydrolysing)	6.3.5.5	E.coli	6408083
carbamoyl-phosphate synthase (glutamine-hydrolysing)	6.3.5.5	E.coli	7053379
carbamoyl-phosphate synthase (glutamine-hydrolysing)	6.3.5.5	E.coli	7209543
carbamoyl-phosphate synthase (glutamine-hydrolysing)	6.3.5.5	E.coli	7608487
carbamoyl-phosphate synthase (glutamine-hydrolysing)	6.3.5.5	E.coli	7916269
acetyl-CoA carboxylase	6.4.1.2	E.coli	10098661
acetyl-CoA carboxylase	6.4.1.2	E.coli	10215591
acetyl-CoA carboxylase	6.4.1.2	E.coli	10757783
acetyl-CoA carboxylase	6.4.1.2	E.coli	10945143
acetyl-CoA carboxylase	6.4.1.2	E.coli	11078738
acetyl-CoA carboxylase	6.4.1.2	E.coli	11205884
acetyl-CoA carboxylase	6.4.1.2	E.coli	11504381
acetyl-CoA carboxylase	6.4.1.2	E.coli	11515553
acetyl-CoA carboxylase	6.4.1.2	E.coli	11546765
acetyl-CoA carboxylase	6.4.1.2	E.coli	12440972
acetyl-CoA carboxylase	6.4.1.2	E.coli	14627750
acetyl-CoA carboxylase	6.4.1.2	E.coli	15333468
acetyl-CoA carboxylase	6.4.1.2	E.coli	15607423
acetyl-CoA carboxylase	6.4.1.2	E.coli	15607568
acetyl-CoA carboxylase	6.4.1.2	E.coli	16222055
acetyl-CoA carboxylase	6.4.1.2	E.coli	16707454
acetyl-CoA carboxylase	6.4.1.2	E.coli	16968879

acetyl-CoA carboxylase	6.4.1.2	E.coli	17266990
acetyl-CoA carboxylase	6.4.1.2	E.coli	17653193
acetyl-CoA carboxylase	6.4.1.2	E.coli	1978829
acetyl-CoA carboxylase	6.4.1.2	E.coli	2570725
acetyl-CoA carboxylase	6.4.1.2	E.coli	2861941
acetyl-CoA carboxylase	6.4.1.2	E.coli	2894828
acetyl-CoA carboxylase	6.4.1.2	E.coli	7436865
acetyl-CoA carboxylase	6.4.1.2	E.coli	7903266
acetyl-CoA carboxylase	6.4.1.2	E.coli	7915138
acetyl-CoA carboxylase	6.4.1.2	E.coli	8814137
acetyl-CoA carboxylase	6.4.1.2	E.coli	9028876
acetyl-CoA carboxylase	6.4.1.2	E.coli	9082912
acetyl-CoA carboxylase	6.4.1.2	E.coli	9109840
alcohol dehydrogenase	1.1.1.1	Human	1096557
alcohol dehydrogenase	1.1.1.1	Human	11303599
alcohol dehydrogenase	1.1.1.1	Human	1148277
alcohol dehydrogenase	1.1.1.1	Human	12147722
alcohol dehydrogenase	1.1.1.1	Human	12489977
alcohol dehydrogenase	1.1.1.1	Human	16662798
alcohol dehydrogenase	1.1.1.1	Human	167557
alcohol dehydrogenase	1.1.1.1	Human	2932116
alcohol dehydrogenase	1.1.1.1	Human	3067025
alcohol dehydrogenase	1.1.1.1	Human	3893194
alcohol dehydrogenase	1.1.1.1	Human	4038269
alcohol dehydrogenase	1.1.1.1	Human	6340613
alcohol dehydrogenase	1.1.1.1	Human	6341787
alcohol dehydrogenase	1.1.1.1	Human	6356161
alcohol dehydrogenase	1.1.1.1	Human	6363888
alcohol dehydrogenase	1.1.1.1	Human	6370140
alcohol dehydrogenase	1.1.1.1	Human	8277258
alcohol dehydrogenase	1.1.1.1	Human	8692838
alcohol dehydrogenase	1.1.1.1	Human	8905240
alcohol dehydrogenase	1.1.1.1	Human	9526508
retinol dehydrogenase	1.1.1.105	Human	11377978
3beta-hydroxy-Delta5-steroid dehydrogenase	1.1.1.145	Human	12441193
3beta-hydroxy-Delta5-steroid dehydrogenase	1.1.1.145	Human	12782399
3beta-hydroxy-Delta5-steroid dehydrogenase	1.1.1.145	Human	2019257
3beta-hydroxy-Delta5-steroid dehydrogenase	1.1.1.145	Human	239964

3beta-hydroxy-Delta5-steroid dehydrogenase	1.1.1.145	Human	7736258
3beta-hydroxy-Delta5-steroid dehydrogenase	1.1.1.145	Human	8574339
IMP dehydrogenase	1.1.1.205	Human	10194364
IMP dehydrogenase	1.1.1.205	Human	10390601
IMP dehydrogenase	1.1.1.205	Human	10390603
IMP dehydrogenase	1.1.1.205	Human	10391669
IMP dehydrogenase	1.1.1.205	Human	10417742
IMP dehydrogenase	1.1.1.205	Human	10930578
IMP dehydrogenase	1.1.1.205	Human	10953035
IMP dehydrogenase	1.1.1.205	Human	10953295
IMP dehydrogenase	1.1.1.205	Human	10973868
IMP dehydrogenase	1.1.1.205	Human	11003640
IMP dehydrogenase	1.1.1.205	Human	1106431
IMP dehydrogenase	1.1.1.205	Human	11076502
IMP dehydrogenase	1.1.1.205	Human	11145582
IMP dehydrogenase	1.1.1.205	Human	11223253
IMP dehydrogenase	1.1.1.205	Human	11233304
IMP dehydrogenase	1.1.1.205	Human	11288107
IMP dehydrogenase	1.1.1.205	Human	11454943
IMP dehydrogenase	1.1.1.205	Human	11522119
IMP dehydrogenase	1.1.1.205	Human	11566360
IMP dehydrogenase	1.1.1.205	Human	11712223
IMP dehydrogenase	1.1.1.205	Human	11724288
IMP dehydrogenase	1.1.1.205	Human	11875050
IMP dehydrogenase	1.1.1.205	Human	11966437
IMP dehydrogenase	1.1.1.205	Human	11966440
IMP dehydrogenase	1.1.1.205	Human	11966441
IMP dehydrogenase	1.1.1.205	Human	12014950
IMP dehydrogenase	1.1.1.205	Human	12183689
IMP dehydrogenase	1.1.1.205	Human	12213477
IMP dehydrogenase	1.1.1.205	Human	12235158
IMP dehydrogenase	1.1.1.205	Human	12403633
IMP dehydrogenase	1.1.1.205	Human	12559919
IMP dehydrogenase	1.1.1.205	Human	12609835
IMP dehydrogenase	1.1.1.205	Human	12746440
IMP dehydrogenase	1.1.1.205	Human	12773970
IMP dehydrogenase	1.1.1.205	Human	12944494
IMP dehydrogenase	1.1.1.205	Human	1353938
IMP dehydrogenase	1.1.1.205	Human	1356621
IMP dehydrogenase	1.1.1.205	Human	14703952
IMP dehydrogenase	1.1.1.205	Human	14757177

IMP dehydrogenase	1.1.1.205	Human	14766016
IMP dehydrogenase	1.1.1.205	Human	14973196
IMP dehydrogenase	1.1.1.205	Human	14981049
IMP dehydrogenase	1.1.1.205	Human	15043157
IMP dehydrogenase	1.1.1.205	Human	15083807
IMP dehydrogenase	1.1.1.205	Human	15292516
IMP dehydrogenase	1.1.1.205	Human	15355510
IMP dehydrogenase	1.1.1.205	Human	15829418
IMP dehydrogenase	1.1.1.205	Human	15869715
IMP dehydrogenase	1.1.1.205	Human	15882147
IMP dehydrogenase	1.1.1.205	Human	15940263
IMP dehydrogenase	1.1.1.205	Human	16128570
IMP dehydrogenase	1.1.1.205	Human	16243838
IMP dehydrogenase	1.1.1.205	Human	16248022
IMP dehydrogenase	1.1.1.205	Human	16333815
IMP dehydrogenase	1.1.1.205	Human	16647299
IMP dehydrogenase	1.1.1.205	Human	16725387
IMP dehydrogenase	1.1.1.205	Human	1677309
IMP dehydrogenase	1.1.1.205	Human	16936083
IMP dehydrogenase	1.1.1.205	Human	17100698
IMP dehydrogenase	1.1.1.205	Human	1717828
IMP dehydrogenase	1.1.1.205	Human	1723703
IMP dehydrogenase	1.1.1.205	Human	1975748
IMP dehydrogenase	1.1.1.205	Human	197916
IMP dehydrogenase	1.1.1.205	Human	2902093
IMP dehydrogenase	1.1.1.205	Human	3314714
IMP dehydrogenase	1.1.1.205	Human	4868171
IMP dehydrogenase	1.1.1.205	Human	6120758
IMP dehydrogenase	1.1.1.205	Human	7476879
IMP dehydrogenase	1.1.1.205	Human	7476895
IMP dehydrogenase	1.1.1.205	Human	7520100
IMP dehydrogenase	1.1.1.205	Human	7874783
IMP dehydrogenase	1.1.1.205	Human	7903533
IMP dehydrogenase	1.1.1.205	Human	7914720
IMP dehydrogenase	1.1.1.205	Human	8103312
IMP dehydrogenase	1.1.1.205	Human	8555204
IMP dehydrogenase	1.1.1.205	Human	8560580
IMP dehydrogenase	1.1.1.205	Human	8830834
IMP dehydrogenase	1.1.1.205	Human	8869741
IMP dehydrogenase	1.1.1.205	Human	8910338
IMP dehydrogenase	1.1.1.205	Human	9042309
IMP dehydrogenase	1.1.1.205	Human	9108641
IMP dehydrogenase	1.1.1.205	Human	9268334

IMP dehydrogenase	1.1.1.205	Human	9278455
IMP dehydrogenase	1.1.1.205	Human	9339960
IMP dehydrogenase	1.1.1.205	Human	9399601
IMP dehydrogenase	1.1.1.205	Human	9413163
IMP dehydrogenase	1.1.1.205	Human	9436988
IMP dehydrogenase	1.1.1.205	Human	9752721
IMP dehydrogenase	1.1.1.205	Human	9766533
IMP dehydrogenase	1.1.1.205	Human	9881055
aldehyde reductase	1.1.1.21	Human	10424772
aldehyde reductase	1.1.1.21	Human	10656235
aldehyde reductase	1.1.1.21	Human	11095596
aldehyde reductase	1.1.1.21	Human	11370705
aldehyde reductase	1.1.1.21	Human	11422753
aldehyde reductase	1.1.1.21	Human	11440832
aldehyde reductase	1.1.1.21	Human	11798960
aldehyde reductase	1.1.1.21	Human	12135102
aldehyde reductase	1.1.1.21	Human	12363257
aldehyde reductase	1.1.1.21	Human	12394272
aldehyde reductase	1.1.1.21	Human	12871133
aldehyde reductase	1.1.1.21	Human	12871136
aldehyde reductase	1.1.1.21	Human	12881532
aldehyde reductase	1.1.1.21	Human	1393828
aldehyde reductase	1.1.1.21	Human	14577653
aldehyde reductase	1.1.1.21	Human	1499867
aldehyde reductase	1.1.1.21	Human	15210146
aldehyde reductase	1.1.1.21	Human	15569136
aldehyde reductase	1.1.1.21	Human	15584919
aldehyde reductase	1.1.1.21	Human	15734861
aldehyde reductase	1.1.1.21	Human	15736047
aldehyde reductase	1.1.1.21	Human	16026266
aldehyde reductase	1.1.1.21	Human	16037296
aldehyde reductase	1.1.1.21	Human	16048249
aldehyde reductase	1.1.1.21	Human	16114079
aldehyde reductase	1.1.1.21	Human	16452468
aldehyde reductase	1.1.1.21	Human	16806328
aldehyde reductase	1.1.1.21	Human	16870454
aldehyde reductase	1.1.1.21	Human	16900332
aldehyde reductase	1.1.1.21	Human	16911628
aldehyde reductase	1.1.1.21	Human	2120282
aldehyde reductase	1.1.1.21	Human	3025043
aldehyde reductase	1.1.1.21	Human	7641310
aldehyde reductase	1.1.1.21	Human	8457142
aldehyde reductase	1.1.1.21	Human	9215310

aldehyde reductase	1.1.1.21	Human	9454604
aldehyde reductase	1.1.1.21	Human	9481088
aldehyde reductase	1.1.1.21	Human	9709964
UDP-glucose 6-dehydrogenase	1.1.1.22	Human	11044215
UDP-glucose 6-dehydrogenase	1.1.1.22	Human	15741737
UDP-glucose 6-dehydrogenase	1.1.1.22	Human	2778766
UDP-glucose 6-dehydrogenase	1.1.1.22	Human	8471533
hydroxymethylglutaryl-CoA reductase (NADPH)	1.1.1.34	Human	10084306
hydroxymethylglutaryl-CoA reductase (NADPH)	1.1.1.34	Human	10204089
hydroxymethylglutaryl-CoA reductase (NADPH)	1.1.1.34	Human	10377386
hydroxymethylglutaryl-CoA reductase (NADPH)	1.1.1.34	Human	10892724
hydroxymethylglutaryl-CoA reductase (NADPH)	1.1.1.34	Human	10964918
hydroxymethylglutaryl-CoA reductase (NADPH)	1.1.1.34	Human	11043510
hydroxymethylglutaryl-CoA reductase (NADPH)	1.1.1.34	Human	11516100
hydroxymethylglutaryl-CoA reductase (NADPH)	1.1.1.34	Human	11881568
hydroxymethylglutaryl-CoA reductase (NADPH)	1.1.1.34	Human	12405293
hydroxymethylglutaryl-CoA reductase (NADPH)	1.1.1.34	Human	12467639
hydroxymethylglutaryl-CoA reductase (NADPH)	1.1.1.34	Human	12736772
hydroxymethylglutaryl-CoA reductase (NADPH)	1.1.1.34	Human	14523992
hydroxymethylglutaryl-CoA reductase (NADPH)	1.1.1.34	Human	14720509
hydroxymethylglutaryl-CoA reductase (NADPH)	1.1.1.34	Human	15034683
hydroxymethylglutaryl-CoA reductase (NADPH)	1.1.1.34	Human	15248477
hydroxymethylglutaryl-CoA reductase (NADPH)	1.1.1.34	Human	15605175
hydroxymethylglutaryl-CoA reductase (NADPH)	1.1.1.34	Human	16101500
hydroxymethylglutaryl-CoA reductase (NADPH)	1.1.1.34	Human	16168377

hydroxymethylglutaryl-CoA reductase (NADPH)	1.1.1.34	Human	1629633
hydroxymethylglutaryl-CoA reductase (NADPH)	1.1.1.34	Human	1652430
hydroxymethylglutaryl-CoA reductase (NADPH)	1.1.1.34	Human	16611135
hydroxymethylglutaryl-CoA reductase (NADPH)	1.1.1.34	Human	187533
hydroxymethylglutaryl-CoA reductase (NADPH)	1.1.1.34	Human	1954650
hydroxymethylglutaryl-CoA reductase (NADPH)	1.1.1.34	Human	216867
hydroxymethylglutaryl-CoA reductase (NADPH)	1.1.1.34	Human	2719596
hydroxymethylglutaryl-CoA reductase (NADPH)	1.1.1.34	Human	278983
hydroxymethylglutaryl-CoA reductase (NADPH)	1.1.1.34	Human	2921640
hydroxymethylglutaryl-CoA reductase (NADPH)	1.1.1.34	Human	2995161
hydroxymethylglutaryl-CoA reductase (NADPH)	1.1.1.34	Human	3055919
hydroxymethylglutaryl-CoA reductase (NADPH)	1.1.1.34	Human	3131638
hydroxymethylglutaryl-CoA reductase (NADPH)	1.1.1.34	Human	3308873
hydroxymethylglutaryl-CoA reductase (NADPH)	1.1.1.34	Human	3314447
hydroxymethylglutaryl-CoA reductase (NADPH)	1.1.1.34	Human	3689494
hydroxymethylglutaryl-CoA reductase (NADPH)	1.1.1.34	Human	3968683
hydroxymethylglutaryl-CoA reductase (NADPH)	1.1.1.34	Human	4075700
hydroxymethylglutaryl-CoA reductase (NADPH)	1.1.1.34	Human	6088070
hydroxymethylglutaryl-CoA reductase (NADPH)	1.1.1.34	Human	6256737
hydroxymethylglutaryl-CoA reductase (NADPH)	1.1.1.34	Human	6274615
hydroxymethylglutaryl-CoA reductase (NADPH)	1.1.1.34	Human	6286363

hydroxymethylglutaryl-CoA reductase (NADPH)	1.1.1.34	Human	6347025
hydroxymethylglutaryl-CoA reductase (NADPH)	1.1.1.34	Human	6396116
hydroxymethylglutaryl-CoA reductase (NADPH)	1.1.1.34	Human	6594693
hydroxymethylglutaryl-CoA reductase (NADPH)	1.1.1.34	Human	666819
hydroxymethylglutaryl-CoA reductase (NADPH)	1.1.1.34	Human	6685129
hydroxymethylglutaryl-CoA reductase (NADPH)	1.1.1.34	Human	6698994
hydroxymethylglutaryl-CoA reductase (NADPH)	1.1.1.34	Human	8054400
hydroxymethylglutaryl-CoA reductase (NADPH)	1.1.1.34	Human	8182149
hydroxymethylglutaryl-CoA reductase (NADPH)	1.1.1.34	Human	8393338
hydroxymethylglutaryl-CoA reductase (NADPH)	1.1.1.34	Human	8504036
hydroxymethylglutaryl-CoA reductase (NADPH)	1.1.1.34	Human	8772195
hydroxymethylglutaryl-CoA reductase (NADPH)	1.1.1.34	Human	9151797
hydroxymethylglutaryl-CoA reductase (NADPH)	1.1.1.34	Human	9185766
hydroxymethylglutaryl-CoA reductase (NADPH)	1.1.1.34	Human	9372476
hydroxymethylglutaryl-CoA reductase (NADPH)	1.1.1.34	Human	9558731
hydroxymethylglutaryl-CoA reductase (NADPH)	1.1.1.34	Human	9802623
3-hydroxyacyl-CoA dehydrogenase	1.1.1.35	Human	15358356
3-hydroxyacyl-CoA dehydrogenase	1.1.1.35	Human	1637289
3-hydroxyacyl-CoA dehydrogenase	1.1.1.35	Human	2388659
3-hydroxyacyl-CoA dehydrogenase	1.1.1.35	Human	6588129
malate dehydrogenase	1.1.1.37	Human	11583380
malate dehydrogenase	1.1.1.37	Human	11767008
malate dehydrogenase	1.1.1.37	Human	11855723
malate dehydrogenase	1.1.1.37	Human	131232
malate dehydrogenase	1.1.1.37	Human	16028114
malate dehydrogenase	1.1.1.37	Human	16212411
malate dehydrogenase	1.1.1.37	Human	16661455

malate dehydrogenase	1.1.1.37	Human	3995045
malate dehydrogenase	1.1.1.37	Human	4053567
malate dehydrogenase	1.1.1.37	Human	7138874
malate dehydrogenase	1.1.1.37	Human	8624506
malate dehydrogenase	1.1.1.37	Human	9348107
isocitrate dehydrogenase (NAD+)	1.1.1.41	Human	10461937
isocitrate dehydrogenase (NAD+)	1.1.1.41	Human	12033940
isocitrate dehydrogenase (NAD+)	1.1.1.41	Human	12619682
isocitrate dehydrogenase (NAD+)	1.1.1.41	Human	14555658
isocitrate dehydrogenase (NAD+)	1.1.1.41	Human	15173171
isocitrate dehydrogenase (NAD+)	1.1.1.41	Human	15314217
isocitrate dehydrogenase (NAD+)	1.1.1.41	Human	16415587
isocitrate dehydrogenase (NAD+)	1.1.1.41	Human	6389540
isocitrate dehydrogenase (NAD+)	1.1.1.41	Human	7710326
isocitrate dehydrogenase (NAD+)	1.1.1.41	Human	8626605
isocitrate dehydrogenase (NAD+)	1.1.1.41	Human	9733544
isocitrate dehydrogenase (NAD+)	1.1.1.41	Human	9881153
glucose-6-phosphate dehydrogenase	1.1.1.49	Human	10098886
glucose-6-phosphate dehydrogenase	1.1.1.49	Human	10099785
glucose-6-phosphate dehydrogenase	1.1.1.49	Human	10329961
glucose-6-phosphate dehydrogenase	1.1.1.49	Human	10825753
glucose-6-phosphate dehydrogenase	1.1.1.49	Human	10998184
glucose-6-phosphate dehydrogenase	1.1.1.49	Human	11023706
glucose-6-phosphate dehydrogenase	1.1.1.49	Human	11245448
glucose-6-phosphate dehydrogenase	1.1.1.49	Human	11463792
glucose-6-phosphate dehydrogenase	1.1.1.49	Human	11520909
glucose-6-phosphate dehydrogenase	1.1.1.49	Human	12027950
glucose-6-phosphate dehydrogenase	1.1.1.49	Human	12204336
glucose-6-phosphate dehydrogenase	1.1.1.49	Human	12393032
glucose-6-phosphate dehydrogenase	1.1.1.49	Human	12414804
glucose-6-phosphate dehydrogenase	1.1.1.49	Human	12453665
glucose-6-phosphate dehydrogenase	1.1.1.49	Human	12472120
glucose-6-phosphate dehydrogenase	1.1.1.49	Human	12502759
glucose-6-phosphate dehydrogenase	1.1.1.49	Human	131232
glucose-6-phosphate dehydrogenase	1.1.1.49	Human	1384463
glucose-6-phosphate dehydrogenase	1.1.1.49	Human	1417703
glucose-6-phosphate dehydrogenase	1.1.1.49	Human	147929
glucose-6-phosphate dehydrogenase	1.1.1.49	Human	15331344
glucose-6-phosphate dehydrogenase	1.1.1.49	Human	15345489
glucose-6-phosphate dehydrogenase	1.1.1.49	Human	15527069
glucose-6-phosphate dehydrogenase	1.1.1.49	Human	15550513
glucose-6-phosphate dehydrogenase	1.1.1.49	Human	15634201
glucose-6-phosphate dehydrogenase	1.1.1.49	Human	15739803

glucose-6-phosphate dehydrogenase	1.1.1.49	Human	15760711
glucose-6-phosphate dehydrogenase	1.1.1.49	Human	15858258
glucose-6-phosphate dehydrogenase	1.1.1.49	Human	15975496
glucose-6-phosphate dehydrogenase	1.1.1.49	Human	16039947
glucose-6-phosphate dehydrogenase	1.1.1.49	Human	16439706
glucose-6-phosphate dehydrogenase	1.1.1.49	Human	16849632
glucose-6-phosphate dehydrogenase	1.1.1.49	Human	17157446
glucose-6-phosphate dehydrogenase	1.1.1.49	Human	1830744
glucose-6-phosphate dehydrogenase	1.1.1.49	Human	1922658
glucose-6-phosphate dehydrogenase	1.1.1.49	Human	1978808
glucose-6-phosphate dehydrogenase	1.1.1.49	Human	2208076
glucose-6-phosphate dehydrogenase	1.1.1.49	Human	2296762
glucose-6-phosphate dehydrogenase	1.1.1.49	Human	2767006
glucose-6-phosphate dehydrogenase	1.1.1.49	Human	2808772
glucose-6-phosphate dehydrogenase	1.1.1.49	Human	2846196
glucose-6-phosphate dehydrogenase	1.1.1.49	Human	2984461
glucose-6-phosphate dehydrogenase	1.1.1.49	Human	3116361
glucose-6-phosphate dehydrogenase	1.1.1.49	Human	3161339
glucose-6-phosphate dehydrogenase	1.1.1.49	Human	3316204
glucose-6-phosphate dehydrogenase	1.1.1.49	Human	3337882
glucose-6-phosphate dehydrogenase	1.1.1.49	Human	3365274
glucose-6-phosphate dehydrogenase	1.1.1.49	Human	3532684
glucose-6-phosphate dehydrogenase	1.1.1.49	Human	3621197
glucose-6-phosphate dehydrogenase	1.1.1.49	Human	3717951
glucose-6-phosphate dehydrogenase	1.1.1.49	Human	3765490
glucose-6-phosphate dehydrogenase	1.1.1.49	Human	3949801
glucose-6-phosphate dehydrogenase	1.1.1.49	Human	4288679
glucose-6-phosphate dehydrogenase	1.1.1.49	Human	4382249
glucose-6-phosphate dehydrogenase	1.1.1.49	Human	4400642
glucose-6-phosphate dehydrogenase	1.1.1.49	Human	4941552
glucose-6-phosphate dehydrogenase	1.1.1.49	Human	6295653
glucose-6-phosphate dehydrogenase	1.1.1.49	Human	6341787
glucose-6-phosphate dehydrogenase	1.1.1.49	Human	6363888
glucose-6-phosphate dehydrogenase	1.1.1.49	Human	6420889
glucose-6-phosphate dehydrogenase	1.1.1.49	Human	645360
glucose-6-phosphate dehydrogenase	1.1.1.49	Human	6591771
glucose-6-phosphate dehydrogenase	1.1.1.49	Human	6696439
glucose-6-phosphate dehydrogenase	1.1.1.49	Human	7126822
glucose-6-phosphate dehydrogenase	1.1.1.49	Human	7578910
glucose-6-phosphate dehydrogenase	1.1.1.49	Human	7681896
glucose-6-phosphate dehydrogenase	1.1.1.49	Human	7768207
glucose-6-phosphate dehydrogenase	1.1.1.49	Human	7930940
glucose-6-phosphate dehydrogenase	1.1.1.49	Human	8316633

glucose-6-phosphate dehydrogenase	1.1.1.49	Human	864
glucose-6-phosphate dehydrogenase	1.1.1.49	Human	8760336
glucose-6-phosphate dehydrogenase	1.1.1.49	Human	8797095
glucose-6-phosphate dehydrogenase	1.1.1.49	Human	8857518
glucose-6-phosphate dehydrogenase	1.1.1.49	Human	8910528
glucose-6-phosphate dehydrogenase	1.1.1.49	Human	8954569
glucose-6-phosphate dehydrogenase	1.1.1.49	Human	9042391
glucose-6-phosphate dehydrogenase	1.1.1.49	Human	9553122
glucose-6-phosphate dehydrogenase	1.1.1.49	Human	9581796
glucose-6-phosphate dehydrogenase	1.1.1.49	Human	9915806
choline dehydrogenase	1.1.99.1	Human	8868068
ubiquinol---cytochrome-c reductase	1.10.2.2	Human	16005845
ubiquinol---cytochrome-c reductase	1.10.2.2	Human	17223530
tryptophan 2,3-dioxygenase	1.13.11.11	Human	10966936
tryptophan 2,3-dioxygenase	1.13.11.11	Human	17761498
tryptophan 2,3-dioxygenase	1.13.11.11	Human	2215078
tryptophan 2,3-dioxygenase	1.13.11.11	Human	4293961
tryptophan 2,3-dioxygenase	1.13.11.11	Human	8806758
tryptophan 2,3-dioxygenase	1.13.11.11	Human	9291104
4-hydroxyphenylpyruvate dioxygenase	1.13.11.27	Human	10098661
4-hydroxyphenylpyruvate dioxygenase	1.13.11.27	Human	12014960
4-hydroxyphenylpyruvate dioxygenase	1.13.11.27	Human	12127941
4-hydroxyphenylpyruvate dioxygenase	1.13.11.27	Human	240411
4-hydroxyphenylpyruvate dioxygenase	1.13.11.27	Human	9701587
arachidonate 5-lipoxygenase	1.13.11.34	Human	10337029
arachidonate 5-lipoxygenase	1.13.11.34	Human	10378985
arachidonate 5-lipoxygenase	1.13.11.34	Human	10978197
arachidonate 5-lipoxygenase	1.13.11.34	Human	10984486
arachidonate 5-lipoxygenase	1.13.11.34	Human	11369634
arachidonate 5-lipoxygenase	1.13.11.34	Human	11394934
arachidonate 5-lipoxygenase	1.13.11.34	Human	11488449
arachidonate 5-lipoxygenase	1.13.11.34	Human	11542380
arachidonate 5-lipoxygenase	1.13.11.34	Human	11706027
arachidonate 5-lipoxygenase	1.13.11.34	Human	11730938
arachidonate 5-lipoxygenase	1.13.11.34	Human	11844797
arachidonate 5-lipoxygenase	1.13.11.34	Human	12142344
arachidonate 5-lipoxygenase	1.13.11.34	Human	12205041
arachidonate 5-lipoxygenase	1.13.11.34	Human	1311589
arachidonate 5-lipoxygenase	1.13.11.34	Human	1357659
arachidonate 5-lipoxygenase	1.13.11.34	Human	15120715
arachidonate 5-lipoxygenase	1.13.11.34	Human	15265316
arachidonate 5-lipoxygenase	1.13.11.34	Human	15640495
arachidonate 5-lipoxygenase	1.13.11.34	Human	15852496

arachidonate 5-lipoxygenase	1.13.11.34	Human	15947420
arachidonate 5-lipoxygenase	1.13.11.34	Human	16165096
arachidonate 5-lipoxygenase	1.13.11.34	Human	16216483
arachidonate 5-lipoxygenase	1.13.11.34	Human	16223251
arachidonate 5-lipoxygenase	1.13.11.34	Human	16566819
arachidonate 5-lipoxygenase	1.13.11.34	Human	16787324
arachidonate 5-lipoxygenase	1.13.11.34	Human	16821792
arachidonate 5-lipoxygenase	1.13.11.34	Human	16868922
arachidonate 5-lipoxygenase	1.13.11.34	Human	17376394
arachidonate 5-lipoxygenase	1.13.11.34	Human	1903222
arachidonate 5-lipoxygenase	1.13.11.34	Human	1910248
arachidonate 5-lipoxygenase	1.13.11.34	Human	2649885
arachidonate 5-lipoxygenase	1.13.11.34	Human	3417684
arachidonate 5-lipoxygenase	1.13.11.34	Human	6135325
arachidonate 5-lipoxygenase	1.13.11.34	Human	6312489
arachidonate 5-lipoxygenase	1.13.11.34	Human	7768385
arachidonate 5-lipoxygenase	1.13.11.34	Human	7780156
arachidonate 5-lipoxygenase	1.13.11.34	Human	8140120
arachidonate 5-lipoxygenase	1.13.11.34	Human	8245474
arachidonate 5-lipoxygenase	1.13.11.34	Human	8283055
arachidonate 5-lipoxygenase	1.13.11.34	Human	8364900
arachidonate 5-lipoxygenase	1.13.11.34	Human	8567845
arachidonate 5-lipoxygenase	1.13.11.34	Human	9049593
arachidonate 5-lipoxygenase	1.13.11.34	Human	9133649
arachidonate 5-lipoxygenase	1.13.11.34	Human	9411661
arachidonate 5-lipoxygenase	1.13.11.34	Human	9491904
arachidonate 5-lipoxygenase	1.13.11.34	Human	9873115
indoleamine 2,3-dioxygenase	1.13.11.52	Human	10721098
indoleamine 2,3-dioxygenase	1.13.11.52	Human	10731095
indoleamine 2,3-dioxygenase	1.13.11.52	Human	10833386
indoleamine 2,3-dioxygenase	1.13.11.52	Human	10926204
indoleamine 2,3-dioxygenase	1.13.11.52	Human	10939283
indoleamine 2,3-dioxygenase	1.13.11.52	Human	10939284
indoleamine 2,3-dioxygenase	1.13.11.52	Human	10957719
indoleamine 2,3-dioxygenase	1.13.11.52	Human	11180976
indoleamine 2,3-dioxygenase	1.13.11.52	Human	11230514
indoleamine 2,3-dioxygenase	1.13.11.52	Human	11440641
indoleamine 2,3-dioxygenase	1.13.11.52	Human	11477543
indoleamine 2,3-dioxygenase	1.13.11.52	Human	11507170
indoleamine 2,3-dioxygenase	1.13.11.52	Human	12414962
indoleamine 2,3-dioxygenase	1.13.11.52	Human	12832720
indoleamine 2,3-dioxygenase	1.13.11.52	Human	12848846
indoleamine 2,3-dioxygenase	1.13.11.52	Human	15206741

indoleamine 2,3-dioxygenase	1.13.11.52	Human	15254594
indoleamine 2,3-dioxygenase	1.13.11.52	Human	15358362
indoleamine 2,3-dioxygenase	1.13.11.52	Human	15542091
indoleamine 2,3-dioxygenase	1.13.11.52	Human	15853924
indoleamine 2,3-dioxygenase	1.13.11.52	Human	15961516
indoleamine 2,3-dioxygenase	1.13.11.52	Human	16075385
indoleamine 2,3-dioxygenase	1.13.11.52	Human	16083346
indoleamine 2,3-dioxygenase	1.13.11.52	Human	16176799
indoleamine 2,3-dioxygenase	1.13.11.52	Human	16624246
indoleamine 2,3-dioxygenase	1.13.11.52	Human	17055065
indoleamine 2,3-dioxygenase	1.13.11.52	Human	8423409
indoleamine 2,3-dioxygenase	1.13.11.52	Human	8702590
indoleamine 2,3-dioxygenase	1.13.11.52	Human	9466588
cholestanetriol 26-monooxygenase	1.14.13.15	Human	11108738
cholestanetriol 26-monooxygenase	1.14.13.15	Human	11166758
cholestanetriol 26-monooxygenase	1.14.13.15	Human	11406622
cholestanetriol 26-monooxygenase	1.14.13.15	Human	15936349
cholestanetriol 26-monooxygenase	1.14.13.15	Human	2019602
cholestanetriol 26-monooxygenase	1.14.13.15	Human	7557873
cholestanetriol 26-monooxygenase	1.14.13.15	Human	8001744
cholesterol 7alpha-monooxygenase	1.14.13.17	Human	10334992
cholesterol 7alpha-monooxygenase	1.14.13.17	Human	10405006
cholesterol 7alpha-monooxygenase	1.14.13.17	Human	10431389
cholesterol 7alpha-monooxygenase	1.14.13.17	Human	10588945
cholesterol 7alpha-monooxygenase	1.14.13.17	Human	10599986
cholesterol 7alpha-monooxygenase	1.14.13.17	Human	10656290
cholesterol 7alpha-monooxygenase	1.14.13.17	Human	10731667
cholesterol 7alpha-monooxygenase	1.14.13.17	Human	10744771
cholesterol 7alpha-monooxygenase	1.14.13.17	Human	10858029
cholesterol 7alpha-monooxygenase	1.14.13.17	Human	10900260
cholesterol 7alpha-monooxygenase	1.14.13.17	Human	10936612
cholesterol 7alpha-monooxygenase	1.14.13.17	Human	11030331
cholesterol 7alpha-monooxygenase	1.14.13.17	Human	11075809
cholesterol 7alpha-monooxygenase	1.14.13.17	Human	11108738
cholesterol 7alpha-monooxygenase	1.14.13.17	Human	11136553
cholesterol 7alpha-monooxygenase	1.14.13.17	Human	11254888
cholesterol 7alpha-monooxygenase	1.14.13.17	Human	11264982
cholesterol 7alpha-monooxygenase	1.14.13.17	Human	11402042
cholesterol 7alpha-monooxygenase	1.14.13.17	Human	11406622
cholesterol 7alpha-monooxygenase	1.14.13.17	Human	11427207
cholesterol 7alpha-monooxygenase	1.14.13.17	Human	11438503
cholesterol 7alpha-monooxygenase	1.14.13.17	Human	11557507
cholesterol 7alpha-monooxygenase	1.14.13.17	Human	11604260

cholesterol 7alpha-monooxygenase	1.14.13.17	Human	11787596
cholesterol 7alpha-monooxygenase	1.14.13.17	Human	11967026
cholesterol 7alpha-monooxygenase	1.14.13.17	Human	12042433
cholesterol 7alpha-monooxygenase	1.14.13.17	Human	12049994
cholesterol 7alpha-monooxygenase	1.14.13.17	Human	12393855
cholesterol 7alpha-monooxygenase	1.14.13.17	Human	1245792
cholesterol 7alpha-monooxygenase	1.14.13.17	Human	12554795
cholesterol 7alpha-monooxygenase	1.14.13.17	Human	12562858
cholesterol 7alpha-monooxygenase	1.14.13.17	Human	12675851
cholesterol 7alpha-monooxygenase	1.14.13.17	Human	12787409
cholesterol 7alpha-monooxygenase	1.14.13.17	Human	12805410
cholesterol 7alpha-monooxygenase	1.14.13.17	Human	12865425
cholesterol 7alpha-monooxygenase	1.14.13.17	Human	12917427
cholesterol 7alpha-monooxygenase	1.14.13.17	Human	14660582
cholesterol 7alpha-monooxygenase	1.14.13.17	Human	14698038
cholesterol 7alpha-monooxygenase	1.14.13.17	Human	14748721
cholesterol 7alpha-monooxygenase	1.14.13.17	Human	14762172
cholesterol 7alpha-monooxygenase	1.14.13.17	Human	14960319
cholesterol 7alpha-monooxygenase	1.14.13.17	Human	15145977
cholesterol 7alpha-monooxygenase	1.14.13.17	Human	15241483
cholesterol 7alpha-monooxygenase	1.14.13.17	Human	15333704
cholesterol 7alpha-monooxygenase	1.14.13.17	Human	15375335
cholesterol 7alpha-monooxygenase	1.14.13.17	Human	15458444
cholesterol 7alpha-monooxygenase	1.14.13.17	Human	15574426
cholesterol 7alpha-monooxygenase	1.14.13.17	Human	15583480
cholesterol 7alpha-monooxygenase	1.14.13.17	Human	15649292
cholesterol 7alpha-monooxygenase	1.14.13.17	Human	15707388
cholesterol 7alpha-monooxygenase	1.14.13.17	Human	15750181
cholesterol 7alpha-monooxygenase	1.14.13.17	Human	15752749
cholesterol 7alpha-monooxygenase	1.14.13.17	Human	15795435
cholesterol 7alpha-monooxygenase	1.14.13.17	Human	15936349
cholesterol 7alpha-monooxygenase	1.14.13.17	Human	16075052
cholesterol 7alpha-monooxygenase	1.14.13.17	Human	16213224
cholesterol 7alpha-monooxygenase	1.14.13.17	Human	16271991
cholesterol 7alpha-monooxygenase	1.14.13.17	Human	16464476
cholesterol 7alpha-monooxygenase	1.14.13.17	Human	17054913
cholesterol 7alpha-monooxygenase	1.14.13.17	Human	223590
cholesterol 7alpha-monooxygenase	1.14.13.17	Human	2713876
cholesterol 7alpha-monooxygenase	1.14.13.17	Human	3768033
cholesterol 7alpha-monooxygenase	1.14.13.17	Human	3981265
cholesterol 7alpha-monooxygenase	1.14.13.17	Human	8663429
cholesterol 7alpha-monooxygenase	1.14.13.17	Human	8759369
cholesterol 7alpha-monooxygenase	1.14.13.17	Human	8858751

cholesterol 7alpha-monooxygenase	1.14.13.17	Human	8895609
cholesterol 7alpha-monooxygenase	1.14.13.17	Human	8943286
cholesterol 7alpha-monooxygenase	1.14.13.17	Human	9013589
cholesterol 7alpha-monooxygenase	1.14.13.17	Human	9151797
cholesterol 7alpha-monooxygenase	1.14.13.17	Human	9185766
cholesterol 7alpha-monooxygenase	1.14.13.17	Human	9462665
cholesterol 7alpha-monooxygenase	1.14.13.17	Human	9550534
cholesterol 7alpha-monooxygenase	1.14.13.17	Human	9558731
cholesterol 7alpha-monooxygenase	1.14.13.17	Human	9625597
cholesterol 7alpha-monooxygenase	1.14.13.17	Human	9630215
cholesterol 7alpha-monooxygenase	1.14.13.17	Human	9649606
cholesterol 7alpha-monooxygenase	1.14.13.17	Human	9721193
cholesterol 7alpha-monooxygenase	1.14.13.17	Human	9799805
cholesterol 7alpha-monooxygenase	1.14.13.17	Human	9881645
nitric-oxide synthase	1.14.13.39	Human	10630682
nitric-oxide synthase	1.14.13.39	Human	10821631
nitric-oxide synthase	1.14.13.39	Human	10868974
nitric-oxide synthase	1.14.13.39	Human	11095648
nitric-oxide synthase	1.14.13.39	Human	11292821
nitric-oxide synthase	1.14.13.39	Human	11328947
nitric-oxide synthase	1.14.13.39	Human	11468403
nitric-oxide synthase	1.14.13.39	Human	11556547
nitric-oxide synthase	1.14.13.39	Human	11852055
nitric-oxide synthase	1.14.13.39	Human	12176955
nitric-oxide synthase	1.14.13.39	Human	12297263
nitric-oxide synthase	1.14.13.39	Human	12354096
nitric-oxide synthase	1.14.13.39	Human	12402580
nitric-oxide synthase	1.14.13.39	Human	12503100
nitric-oxide synthase	1.14.13.39	Human	12566086
nitric-oxide synthase	1.14.13.39	Human	12591128
nitric-oxide synthase	1.14.13.39	Human	12810358
nitric-oxide synthase	1.14.13.39	Human	12830073
nitric-oxide synthase	1.14.13.39	Human	12855421
nitric-oxide synthase	1.14.13.39	Human	12869534
nitric-oxide synthase	1.14.13.39	Human	14662726
nitric-oxide synthase	1.14.13.39	Human	14668561
nitric-oxide synthase	1.14.13.39	Human	14769821
nitric-oxide synthase	1.14.13.39	Human	15099358
nitric-oxide synthase	1.14.13.39	Human	15167268
nitric-oxide synthase	1.14.13.39	Human	15223360
nitric-oxide synthase	1.14.13.39	Human	15448133
nitric-oxide synthase	1.14.13.39	Human	15588718
nitric-oxide synthase	1.14.13.39	Human	15698596

nitric-oxide synthase	1.14.13.39	Human	16179540
nitric-oxide synthase	1.14.13.39	Human	16249336
nitric-oxide synthase	1.14.13.39	Human	16297560
nitric-oxide synthase	1.14.13.39	Human	16315601
nitric-oxide synthase	1.14.13.39	Human	16464859
nitric-oxide synthase	1.14.13.39	Human	16641207
nitric-oxide synthase	1.14.13.39	Human	17003331
nitric-oxide synthase	1.14.13.39	Human	17293453
nitric-oxide synthase	1.14.13.39	Human	7689840
nitric-oxide synthase	1.14.13.39	Human	8694803
nitric-oxide synthase	1.14.13.39	Human	8791097
nitric-oxide synthase	1.14.13.39	Human	8945918
nitric-oxide synthase	1.14.13.39	Human	8958563
nitric-oxide synthase	1.14.13.39	Human	9247967
nitric-oxide synthase	1.14.13.39	Human	9336394
nitric-oxide synthase	1.14.13.39	Human	9409304
nitric-oxide synthase	1.14.13.39	Human	9525991
nitric-oxide synthase	1.14.13.39	Human	9654136
nitric-oxide synthase	1.14.13.39	Human	9665318
nitric-oxide synthase	1.14.13.39	Human	9822514
nitric-oxide synthase	1.14.13.39	Human	9856817
kynurenine 3-monooxygenase	1.14.13.9	Human	42292
kynurenine 3-monooxygenase	1.14.13.9	Human	7131096
kynurenine 3-monooxygenase	1.14.13.9	Human	7131097
unspecific monooxygenase	1.14.14.1	Human	10462973
unspecific monooxygenase	1.14.14.1	Human	10473018
unspecific monooxygenase	1.14.14.1	Human	10474272
unspecific monooxygenase	1.14.14.1	Human	10690899
unspecific monooxygenase	1.14.14.1	Human	10713305
unspecific monooxygenase	1.14.14.1	Human	10746939
unspecific monooxygenase	1.14.14.1	Human	10947336
unspecific monooxygenase	1.14.14.1	Human	11116206
unspecific monooxygenase	1.14.14.1	Human	11259506
unspecific monooxygenase	1.14.14.1	Human	11312650
unspecific monooxygenase	1.14.14.1	Human	11403896
unspecific monooxygenase	1.14.14.1	Human	11502834
unspecific monooxygenase	1.14.14.1	Human	11922772
unspecific monooxygenase	1.14.14.1	Human	11961225
unspecific monooxygenase	1.14.14.1	Human	12053085
unspecific monooxygenase	1.14.14.1	Human	12439219
unspecific monooxygenase	1.14.14.1	Human	12606587
unspecific monooxygenase	1.14.14.1	Human	12706301
unspecific monooxygenase	1.14.14.1	Human	12715371

unspecific monooxygenase	1.14.14.1	Human	12736278
unspecific monooxygenase	1.14.14.1	Human	12810639
unspecific monooxygenase	1.14.14.1	Human	12845227
unspecific monooxygenase	1.14.14.1	Human	12957661
unspecific monooxygenase	1.14.14.1	Human	12960093
unspecific monooxygenase	1.14.14.1	Human	1339246
unspecific monooxygenase	1.14.14.1	Human	14501171
unspecific monooxygenase	1.14.14.1	Human	14580722
unspecific monooxygenase	1.14.14.1	Human	14623534
unspecific monooxygenase	1.14.14.1	Human	14965567
unspecific monooxygenase	1.14.14.1	Human	15226009
unspecific monooxygenase	1.14.14.1	Human	15255840
unspecific monooxygenase	1.14.14.1	Human	15319488
unspecific monooxygenase	1.14.14.1	Human	15322103
unspecific monooxygenase	1.14.14.1	Human	15582747
unspecific monooxygenase	1.14.14.1	Human	15591029
unspecific monooxygenase	1.14.14.1	Human	15623590
unspecific monooxygenase	1.14.14.1	Human	15876405
unspecific monooxygenase	1.14.14.1	Human	15885269
unspecific monooxygenase	1.14.14.1	Human	15930283
unspecific monooxygenase	1.14.14.1	Human	16006326
unspecific monooxygenase	1.14.14.1	Human	16077170
unspecific monooxygenase	1.14.14.1	Human	16109788
unspecific monooxygenase	1.14.14.1	Human	16109840
unspecific monooxygenase	1.14.14.1	Human	16170371
unspecific monooxygenase	1.14.14.1	Human	16260617
unspecific monooxygenase	1.14.14.1	Human	16285913
unspecific monooxygenase	1.14.14.1	Human	16303757
unspecific monooxygenase	1.14.14.1	Human	16322267
unspecific monooxygenase	1.14.14.1	Human	16426763
unspecific monooxygenase	1.14.14.1	Human	16473000
unspecific monooxygenase	1.14.14.1	Human	16541462
unspecific monooxygenase	1.14.14.1	Human	16611627
unspecific monooxygenase	1.14.14.1	Human	16733710
unspecific monooxygenase	1.14.14.1	Human	16763069
unspecific monooxygenase	1.14.14.1	Human	16877675
unspecific monooxygenase	1.14.14.1	Human	1694074
unspecific monooxygenase	1.14.14.1	Human	17005180
unspecific monooxygenase	1.14.14.1	Human	17079138
unspecific monooxygenase	1.14.14.1	Human	17573783
unspecific monooxygenase	1.14.14.1	Human	1835644
unspecific monooxygenase	1.14.14.1	Human	2149503
unspecific monooxygenase	1.14.14.1	Human	3419162

unspecific monooxygenase	1.14.14.1	Human	3626552
unspecific monooxygenase	1.14.14.1	Human	6233132
unspecific monooxygenase	1.14.14.1	Human	6580512
unspecific monooxygenase	1.14.14.1	Human	6645505
unspecific monooxygenase	1.14.14.1	Human	7083186
unspecific monooxygenase	1.14.14.1	Human	7581491
unspecific monooxygenase	1.14.14.1	Human	7626472
unspecific monooxygenase	1.14.14.1	Human	7694590
unspecific monooxygenase	1.14.14.1	Human	7949200
unspecific monooxygenase	1.14.14.1	Human	7968362
unspecific monooxygenase	1.14.14.1	Human	8017854
unspecific monooxygenase	1.14.14.1	Human	8073072
unspecific monooxygenase	1.14.14.1	Human	8126145
unspecific monooxygenase	1.14.14.1	Human	8698750
unspecific monooxygenase	1.14.14.1	Human	8824461
unspecific monooxygenase	1.14.14.1	Human	8989259
unspecific monooxygenase	1.14.14.1	Human	9027406
unspecific monooxygenase	1.14.14.1	Human	9038782
unspecific monooxygenase	1.14.14.1	Human	9187104
unspecific monooxygenase	1.14.14.1	Human	9365209
unspecific monooxygenase	1.14.14.1	Human	9415804
unspecific monooxygenase	1.14.14.1	Human	9442349
unspecific monooxygenase	1.14.14.1	Human	9456245
unspecific monooxygenase	1.14.14.1	Human	9511180
unspecific monooxygenase	1.14.14.1	Human	9523724
unspecific monooxygenase	1.14.14.1	Human	9541188
unspecific monooxygenase	1.14.14.1	Human	9698080
unspecific monooxygenase	1.14.14.1	Human	9703907
unspecific monooxygenase	1.14.14.1	Human	9788750
unspecific monooxygenase	1.14.14.1	Human	9797023
unspecific monooxygenase	1.14.14.1	Human	9804911
cholesterol monooxygenase (side-chain-cleaving)	1.14.15.6	Human	10418987
cholesterol monooxygenase (side-chain-cleaving)	1.14.15.6	Human	10700725
cholesterol monooxygenase (side-chain-cleaving)	1.14.15.6	Human	10729197
cholesterol monooxygenase (side-chain-cleaving)	1.14.15.6	Human	10964798
cholesterol monooxygenase (side-chain-cleaving)	1.14.15.6	Human	11191081
cholesterol monooxygenase (side-chain-cleaving)	1.14.15.6	Human	11297612

cholesterol monooxygenase (side-chain-cleaving)	1.14.15.6	Human	12137805
cholesterol monooxygenase (side-chain-cleaving)	1.14.15.6	Human	12596229
cholesterol monooxygenase (side-chain-cleaving)	1.14.15.6	Human	1282463
cholesterol monooxygenase (side-chain-cleaving)	1.14.15.6	Human	1327721
cholesterol monooxygenase (side-chain-cleaving)	1.14.15.6	Human	1337141
cholesterol monooxygenase (side-chain-cleaving)	1.14.15.6	Human	15026180
cholesterol monooxygenase (side-chain-cleaving)	1.14.15.6	Human	15159300
cholesterol monooxygenase (side-chain-cleaving)	1.14.15.6	Human	15205373
cholesterol monooxygenase (side-chain-cleaving)	1.14.15.6	Human	15231695
cholesterol monooxygenase (side-chain-cleaving)	1.14.15.6	Human	15344917
cholesterol monooxygenase (side-chain-cleaving)	1.14.15.6	Human	15635147
cholesterol monooxygenase (side-chain-cleaving)	1.14.15.6	Human	15666823
cholesterol monooxygenase (side-chain-cleaving)	1.14.15.6	Human	15804366
cholesterol monooxygenase (side-chain-cleaving)	1.14.15.6	Human	16139613
cholesterol monooxygenase (side-chain-cleaving)	1.14.15.6	Human	16410306
cholesterol monooxygenase (side-chain-cleaving)	1.14.15.6	Human	16541462
cholesterol monooxygenase (side-chain-cleaving)	1.14.15.6	Human	16551645
cholesterol monooxygenase (side-chain-cleaving)	1.14.15.6	Human	16632873
cholesterol monooxygenase (side-chain-cleaving)	1.14.15.6	Human	16780839
cholesterol monooxygenase (side-chain-cleaving)	1.14.15.6	Human	16999944
cholesterol monooxygenase (side-chain-cleaving)	1.14.15.6	Human	1700277

cholesterol monooxygenase (side-chain-cleaving)	1.14.15.6	Human	1874173
cholesterol monooxygenase (side-chain-cleaving)	1.14.15.6	Human	2226329
cholesterol monooxygenase (side-chain-cleaving)	1.14.15.6	Human	2555382
cholesterol monooxygenase (side-chain-cleaving)	1.14.15.6	Human	3027455
cholesterol monooxygenase (side-chain-cleaving)	1.14.15.6	Human	3356301
cholesterol monooxygenase (side-chain-cleaving)	1.14.15.6	Human	3502608
cholesterol monooxygenase (side-chain-cleaving)	1.14.15.6	Human	6249251
cholesterol monooxygenase (side-chain-cleaving)	1.14.15.6	Human	6256693
cholesterol monooxygenase (side-chain-cleaving)	1.14.15.6	Human	6546877
cholesterol monooxygenase (side-chain-cleaving)	1.14.15.6	Human	7594421
cholesterol monooxygenase (side-chain-cleaving)	1.14.15.6	Human	7664658
cholesterol monooxygenase (side-chain-cleaving)	1.14.15.6	Human	7669257
cholesterol monooxygenase (side-chain-cleaving)	1.14.15.6	Human	7826890
cholesterol monooxygenase (side-chain-cleaving)	1.14.15.6	Human	7882898
cholesterol monooxygenase (side-chain-cleaving)	1.14.15.6	Human	7956928
cholesterol monooxygenase (side-chain-cleaving)	1.14.15.6	Human	8026494
cholesterol monooxygenase (side-chain-cleaving)	1.14.15.6	Human	8152434
cholesterol monooxygenase (side-chain-cleaving)	1.14.15.6	Human	8278356
cholesterol monooxygenase (side-chain-cleaving)	1.14.15.6	Human	8425475
cholesterol monooxygenase (side-chain-cleaving)	1.14.15.6	Human	8432024
cholesterol monooxygenase (side-chain-cleaving)	1.14.15.6	Human	8547188

cholesterol monooxygenase (side-chain-cleaving)	1.14.15.6	Human	8584034
cholesterol monooxygenase (side-chain-cleaving)	1.14.15.6	Human	9013761
cholesterol monooxygenase (side-chain-cleaving)	1.14.15.6	Human	9326645
cholesterol monooxygenase (side-chain-cleaving)	1.14.15.6	Human	9511181
cholesterol monooxygenase (side-chain-cleaving)	1.14.15.6	Human	9622308
cholesterol monooxygenase (side-chain-cleaving)	1.14.15.6	Human	9635133
cholesterol monooxygenase (side-chain-cleaving)	1.14.15.6	Human	9888540
cholesterol monooxygenase (side-chain-cleaving)	1.14.15.6	Human	9922097
phenylalanine 4-monooxygenase	1.14.16.1	Human	10444341
phenylalanine 4-monooxygenase	1.14.16.1	Human	10800950
phenylalanine 4-monooxygenase	1.14.16.1	Human	10900078
phenylalanine 4-monooxygenase	1.14.16.1	Human	10984661
phenylalanine 4-monooxygenase	1.14.16.1	Human	15493924
phenylalanine 4-monooxygenase	1.14.16.1	Human	16139311
phenylalanine 4-monooxygenase	1.14.16.1	Human	16402341
phenylalanine 4-monooxygenase	1.14.16.1	Human	16429477
phenylalanine 4-monooxygenase	1.14.16.1	Human	3768311
phenylalanine 4-monooxygenase	1.14.16.1	Human	7635153
phenylalanine 4-monooxygenase	1.14.16.1	Human	7769265
phenylalanine 4-monooxygenase	1.14.16.1	Human	8214611
phenylalanine 4-monooxygenase	1.14.16.1	Human	8246172
phenylalanine 4-monooxygenase	1.14.16.1	Human	8502995
phenylalanine 4-monooxygenase	1.14.16.1	Human	8927234
phenylalanine 4-monooxygenase	1.14.16.1	Human	9168638
tyrosine 3-monooxygenase	1.14.16.2	Human	10320089
tyrosine 3-monooxygenase	1.14.16.2	Human	10476677
tyrosine 3-monooxygenase	1.14.16.2	Human	10725922
tyrosine 3-monooxygenase	1.14.16.2	Human	10970027
tyrosine 3-monooxygenase	1.14.16.2	Human	11424954
tyrosine 3-monooxygenase	1.14.16.2	Human	11517172
tyrosine 3-monooxygenase	1.14.16.2	Human	11948424
tyrosine 3-monooxygenase	1.14.16.2	Human	12124430
tyrosine 3-monooxygenase	1.14.16.2	Human	12640623
tyrosine 3-monooxygenase	1.14.16.2	Human	12717737
tyrosine 3-monooxygenase	1.14.16.2	Human	12891655

tyrosine 3-monooxygenase	1.14.16.2	Human	1383560
tyrosine 3-monooxygenase	1.14.16.2	Human	14675149
tyrosine 3-monooxygenase	1.14.16.2	Human	15447670
tyrosine 3-monooxygenase	1.14.16.2	Human	15649149
tyrosine 3-monooxygenase	1.14.16.2	Human	15683467
tyrosine 3-monooxygenase	1.14.16.2	Human	15897221
tyrosine 3-monooxygenase	1.14.16.2	Human	15935064
tyrosine 3-monooxygenase	1.14.16.2	Human	16049992
tyrosine 3-monooxygenase	1.14.16.2	Human	16052322
tyrosine 3-monooxygenase	1.14.16.2	Human	16080996
tyrosine 3-monooxygenase	1.14.16.2	Human	16187166
tyrosine 3-monooxygenase	1.14.16.2	Human	16199893
tyrosine 3-monooxygenase	1.14.16.2	Human	16650497
tyrosine 3-monooxygenase	1.14.16.2	Human	1847187
tyrosine 3-monooxygenase	1.14.16.2	Human	1972039
tyrosine 3-monooxygenase	1.14.16.2	Human	2573869
tyrosine 3-monooxygenase	1.14.16.2	Human	2883182
tyrosine 3-monooxygenase	1.14.16.2	Human	2902543
tyrosine 3-monooxygenase	1.14.16.2	Human	3267347
tyrosine 3-monooxygenase	1.14.16.2	Human	6150485
tyrosine 3-monooxygenase	1.14.16.2	Human	7494448
tyrosine 3-monooxygenase	1.14.16.2	Human	7576974
tyrosine 3-monooxygenase	1.14.16.2	Human	7719703
tyrosine 3-monooxygenase	1.14.16.2	Human	8723206
tyrosine 3-monooxygenase	1.14.16.2	Human	8984738
tyrosine 3-monooxygenase	1.14.16.2	Human	9247090
tyrosine 3-monooxygenase	1.14.16.2	Human	9350044
tyrosine 3-monooxygenase	1.14.16.2	Human	9719456
tyrosine 3-monooxygenase	1.14.16.2	Human	9822156
tyrosine 3-monooxygenase	1.14.16.2	Human	9914719
tryptophan 5-monooxygenase	1.14.16.4	Human	10327914
tryptophan 5-monooxygenase	1.14.16.4	Human	10483053
tryptophan 5-monooxygenase	1.14.16.4	Human	10514446
tryptophan 5-monooxygenase	1.14.16.4	Human	10581400
tryptophan 5-monooxygenase	1.14.16.4	Human	1059145
tryptophan 5-monooxygenase	1.14.16.4	Human	10636468
tryptophan 5-monooxygenase	1.14.16.4	Human	10676966
tryptophan 5-monooxygenase	1.14.16.4	Human	10715363
tryptophan 5-monooxygenase	1.14.16.4	Human	10721034
tryptophan 5-monooxygenase	1.14.16.4	Human	10800950
tryptophan 5-monooxygenase	1.14.16.4	Human	10899755
tryptophan 5-monooxygenase	1.14.16.4	Human	10900076
tryptophan 5-monooxygenase	1.14.16.4	Human	10900078

tryptophan 5-monooxygenase	1.14.16.4	Human	10909123
tryptophan 5-monooxygenase	1.14.16.4	Human	10950846
tryptophan 5-monooxygenase	1.14.16.4	Human	10953292
tryptophan 5-monooxygenase	1.14.16.4	Human	10993738
tryptophan 5-monooxygenase	1.14.16.4	Human	11085312
tryptophan 5-monooxygenase	1.14.16.4	Human	11113315
tryptophan 5-monooxygenase	1.14.16.4	Human	11121198
tryptophan 5-monooxygenase	1.14.16.4	Human	11326294
tryptophan 5-monooxygenase	1.14.16.4	Human	11386854
tryptophan 5-monooxygenase	1.14.16.4	Human	11426508
tryptophan 5-monooxygenase	1.14.16.4	Human	11747434
tryptophan 5-monooxygenase	1.14.16.4	Human	12015221
tryptophan 5-monooxygenase	1.14.16.4	Human	12116193
tryptophan 5-monooxygenase	1.14.16.4	Human	12399958
tryptophan 5-monooxygenase	1.14.16.4	Human	12507391
tryptophan 5-monooxygenase	1.14.16.4	Human	12818363
tryptophan 5-monooxygenase	1.14.16.4	Human	12915291
tryptophan 5-monooxygenase	1.14.16.4	Human	14563478
tryptophan 5-monooxygenase	1.14.16.4	Human	14960297
tryptophan 5-monooxygenase	1.14.16.4	Human	15124006
tryptophan 5-monooxygenase	1.14.16.4	Human	15163437
tryptophan 5-monooxygenase	1.14.16.4	Human	15281067
tryptophan 5-monooxygenase	1.14.16.4	Human	15476687
tryptophan 5-monooxygenase	1.14.16.4	Human	15663479
tryptophan 5-monooxygenase	1.14.16.4	Human	15677682
tryptophan 5-monooxygenase	1.14.16.4	Human	15804496
tryptophan 5-monooxygenase	1.14.16.4	Human	15940290
tryptophan 5-monooxygenase	1.14.16.4	Human	15968084
tryptophan 5-monooxygenase	1.14.16.4	Human	16023677
tryptophan 5-monooxygenase	1.14.16.4	Human	16109589
tryptophan 5-monooxygenase	1.14.16.4	Human	16165107
tryptophan 5-monooxygenase	1.14.16.4	Human	16192985
tryptophan 5-monooxygenase	1.14.16.4	Human	16198203
tryptophan 5-monooxygenase	1.14.16.4	Human	16203956
tryptophan 5-monooxygenase	1.14.16.4	Human	16314762
tryptophan 5-monooxygenase	1.14.16.4	Human	16381305
tryptophan 5-monooxygenase	1.14.16.4	Human	16405505
tryptophan 5-monooxygenase	1.14.16.4	Human	16407550
tryptophan 5-monooxygenase	1.14.16.4	Human	16436194
tryptophan 5-monooxygenase	1.14.16.4	Human	16467214
tryptophan 5-monooxygenase	1.14.16.4	Human	16495936
tryptophan 5-monooxygenase	1.14.16.4	Human	16498236
tryptophan 5-monooxygenase	1.14.16.4	Human	16581041

tryptophan 5-monooxygenase	1.14.16.4	Human	16615082
tryptophan 5-monooxygenase	1.14.16.4	Human	16806098
tryptophan 5-monooxygenase	1.14.16.4	Human	16864580
tryptophan 5-monooxygenase	1.14.16.4	Human	16924415
tryptophan 5-monooxygenase	1.14.16.4	Human	16979275
tryptophan 5-monooxygenase	1.14.16.4	Human	17015812
tryptophan 5-monooxygenase	1.14.16.4	Human	1707735
tryptophan 5-monooxygenase	1.14.16.4	Human	1997008
tryptophan 5-monooxygenase	1.14.16.4	Human	2466525
tryptophan 5-monooxygenase	1.14.16.4	Human	2568404
tryptophan 5-monooxygenase	1.14.16.4	Human	2737466
tryptophan 5-monooxygenase	1.14.16.4	Human	3379411
tryptophan 5-monooxygenase	1.14.16.4	Human	3742199
tryptophan 5-monooxygenase	1.14.16.4	Human	3996672
tryptophan 5-monooxygenase	1.14.16.4	Human	7552299
tryptophan 5-monooxygenase	1.14.16.4	Human	761167
tryptophan 5-monooxygenase	1.14.16.4	Human	8015380
tryptophan 5-monooxygenase	1.14.16.4	Human	8255926
tryptophan 5-monooxygenase	1.14.16.4	Human	8527368
tryptophan 5-monooxygenase	1.14.16.4	Human	8726569
tryptophan 5-monooxygenase	1.14.16.4	Human	8752114
tryptophan 5-monooxygenase	1.14.16.4	Human	9084419
tryptophan 5-monooxygenase	1.14.16.4	Human	9109552
tryptophan 5-monooxygenase	1.14.16.4	Human	9244768
tryptophan 5-monooxygenase	1.14.16.4	Human	9295371
tryptophan 5-monooxygenase	1.14.16.4	Human	9326303
tryptophan 5-monooxygenase	1.14.16.4	Human	9392522
tryptophan 5-monooxygenase	1.14.16.4	Human	9603613
tryptophan 5-monooxygenase	1.14.16.4	Human	9630672
tryptophan 5-monooxygenase	1.14.16.4	Human	9672049
tryptophan 5-monooxygenase	1.14.16.4	Human	9770640
tryptophan 5-monooxygenase	1.14.16.4	Human	9886051
monophenol monooxygenase	1.14.18.1	Human	10102625
monophenol monooxygenase	1.14.18.1	Human	10495428
monophenol monooxygenase	1.14.18.1	Human	10671066
monophenol monooxygenase	1.14.18.1	Human	10691963
monophenol monooxygenase	1.14.18.1	Human	10839460
monophenol monooxygenase	1.14.18.1	Human	10870514
monophenol monooxygenase	1.14.18.1	Human	10956040
monophenol monooxygenase	1.14.18.1	Human	10960773
monophenol monooxygenase	1.14.18.1	Human	10966567
monophenol monooxygenase	1.14.18.1	Human	10998146
monophenol monooxygenase	1.14.18.1	Human	11076759

monophenol monooxygenase	1.14.18.1	Human	11139343
monophenol monooxygenase	1.14.18.1	Human	11167218
monophenol monooxygenase	1.14.18.1	Human	11171088
monophenol monooxygenase	1.14.18.1	Human	11178974
monophenol monooxygenase	1.14.18.1	Human	11180971
monophenol monooxygenase	1.14.18.1	Human	11236829
monophenol monooxygenase	1.14.18.1	Human	11277411
monophenol monooxygenase	1.14.18.1	Human	11493672
monophenol monooxygenase	1.14.18.1	Human	11574907
monophenol monooxygenase	1.14.18.1	Human	11601654
monophenol monooxygenase	1.14.18.1	Human	11602344
monophenol monooxygenase	1.14.18.1	Human	11673476
monophenol monooxygenase	1.14.18.1	Human	11701975
monophenol monooxygenase	1.14.18.1	Human	11799132
monophenol monooxygenase	1.14.18.1	Human	11821691
monophenol monooxygenase	1.14.18.1	Human	11834083
monophenol monooxygenase	1.14.18.1	Human	11851885
monophenol monooxygenase	1.14.18.1	Human	11858948
monophenol monooxygenase	1.14.18.1	Human	12069489
monophenol monooxygenase	1.14.18.1	Human	12201672
monophenol monooxygenase	1.14.18.1	Human	12565907
monophenol monooxygenase	1.14.18.1	Human	12595535
monophenol monooxygenase	1.14.18.1	Human	12950726
monophenol monooxygenase	1.14.18.1	Human	14511124
monophenol monooxygenase	1.14.18.1	Human	14597395
monophenol monooxygenase	1.14.18.1	Human	14615486
monophenol monooxygenase	1.14.18.1	Human	14622170
monophenol monooxygenase	1.14.18.1	Human	14699163
monophenol monooxygenase	1.14.18.1	Human	14726002
monophenol monooxygenase	1.14.18.1	Human	15016305
monophenol monooxygenase	1.14.18.1	Human	15067002
monophenol monooxygenase	1.14.18.1	Human	15089040
monophenol monooxygenase	1.14.18.1	Human	15147727
monophenol monooxygenase	1.14.18.1	Human	15154289
monophenol monooxygenase	1.14.18.1	Human	15250942
monophenol monooxygenase	1.14.18.1	Human	15252133
monophenol monooxygenase	1.14.18.1	Human	15381243
monophenol monooxygenase	1.14.18.1	Human	15520878
monophenol monooxygenase	1.14.18.1	Human	15572362
monophenol monooxygenase	1.14.18.1	Human	15684492
monophenol monooxygenase	1.14.18.1	Human	15748887
monophenol monooxygenase	1.14.18.1	Human	15760340
monophenol monooxygenase	1.14.18.1	Human	15760341

monophenol monooxygenase	1.14.18.1	Human	15895362
monophenol monooxygenase	1.14.18.1	Human	16146766
monophenol monooxygenase	1.14.18.1	Human	16280010
monophenol monooxygenase	1.14.18.1	Human	16293777
monophenol monooxygenase	1.14.18.1	Human	16327152
monophenol monooxygenase	1.14.18.1	Human	16335789
monophenol monooxygenase	1.14.18.1	Human	16456529
monophenol monooxygenase	1.14.18.1	Human	16477373
monophenol monooxygenase	1.14.18.1	Human	16524430
monophenol monooxygenase	1.14.18.1	Human	16579986
monophenol monooxygenase	1.14.18.1	Human	16691509
monophenol monooxygenase	1.14.18.1	Human	16704452
monophenol monooxygenase	1.14.18.1	Human	1671405
monophenol monooxygenase	1.14.18.1	Human	16757562
monophenol monooxygenase	1.14.18.1	Human	16847266
monophenol monooxygenase	1.14.18.1	Human	17083330
monophenol monooxygenase	1.14.18.1	Human	17083484
monophenol monooxygenase	1.14.18.1	Human	1720016
monophenol monooxygenase	1.14.18.1	Human	1739407
monophenol monooxygenase	1.14.18.1	Human	1902569
monophenol monooxygenase	1.14.18.1	Human	1903356
monophenol monooxygenase	1.14.18.1	Human	2118105
monophenol monooxygenase	1.14.18.1	Human	2497447
monophenol monooxygenase	1.14.18.1	Human	2497448
monophenol monooxygenase	1.14.18.1	Human	2498458
monophenol monooxygenase	1.14.18.1	Human	2543086
monophenol monooxygenase	1.14.18.1	Human	2720797
monophenol monooxygenase	1.14.18.1	Human	2983883
monophenol monooxygenase	1.14.18.1	Human	2992767
monophenol monooxygenase	1.14.18.1	Human	3121644
monophenol monooxygenase	1.14.18.1	Human	3127037
monophenol monooxygenase	1.14.18.1	Human	3143738
monophenol monooxygenase	1.14.18.1	Human	3148919
monophenol monooxygenase	1.14.18.1	Human	3927896
monophenol monooxygenase	1.14.18.1	Human	6826662
monophenol monooxygenase	1.14.18.1	Human	7646526
monophenol monooxygenase	1.14.18.1	Human	7685020
monophenol monooxygenase	1.14.18.1	Human	7775602
monophenol monooxygenase	1.14.18.1	Human	7792254
monophenol monooxygenase	1.14.18.1	Human	7811291
monophenol monooxygenase	1.14.18.1	Human	7821799
monophenol monooxygenase	1.14.18.1	Human	7969144
monophenol monooxygenase	1.14.18.1	Human	8039502

monophenol monooxygenase	1.14.18.1	Human	8124097
monophenol monooxygenase	1.14.18.1	Human	8136316
monophenol monooxygenase	1.14.18.1	Human	8138743
monophenol monooxygenase	1.14.18.1	Human	8151127
monophenol monooxygenase	1.14.18.1	Human	8197131
monophenol monooxygenase	1.14.18.1	Human	8270948
monophenol monooxygenase	1.14.18.1	Human	8292889
monophenol monooxygenase	1.14.18.1	Human	8332936
monophenol monooxygenase	1.14.18.1	Human	8433000
monophenol monooxygenase	1.14.18.1	Human	8462726
monophenol monooxygenase	1.14.18.1	Human	8496620
monophenol monooxygenase	1.14.18.1	Human	8577719
monophenol monooxygenase	1.14.18.1	Human	8577860
monophenol monooxygenase	1.14.18.1	Human	8806453
monophenol monooxygenase	1.14.18.1	Human	8861753
monophenol monooxygenase	1.14.18.1	Human	9029814
monophenol monooxygenase	1.14.18.1	Human	9056640
monophenol monooxygenase	1.14.18.1	Human	9088570
monophenol monooxygenase	1.14.18.1	Human	9177196
monophenol monooxygenase	1.14.18.1	Human	9240530
monophenol monooxygenase	1.14.18.1	Human	9417870
monophenol monooxygenase	1.14.18.1	Human	9447965
monophenol monooxygenase	1.14.18.1	Human	9475178
monophenol monooxygenase	1.14.18.1	Human	9500554
monophenol monooxygenase	1.14.18.1	Human	9500997
monophenol monooxygenase	1.14.18.1	Human	9545341
monophenol monooxygenase	1.14.18.1	Human	9593634
monophenol monooxygenase	1.14.18.1	Human	9719456
monophenol monooxygenase	1.14.18.1	Human	9736634
monophenol monooxygenase	1.14.18.1	Human	9788743
monophenol monooxygenase	1.14.18.1	Human	9822646
monophenol monooxygenase	1.14.18.1	Human	9830058
monophenol monooxygenase	1.14.18.1	Human	9853616
monophenol monooxygenase	1.14.18.1	Human	9880801
stearoyl-CoA 9-desaturase	1.14.19.1	Human	10484602
stearoyl-CoA 9-desaturase	1.14.19.1	Human	10581155
stearoyl-CoA 9-desaturase	1.14.19.1	Human	11352637
stearoyl-CoA 9-desaturase	1.14.19.1	Human	11396956
stearoyl-CoA 9-desaturase	1.14.19.1	Human	11415448
stearoyl-CoA 9-desaturase	1.14.19.1	Human	11533264
stearoyl-CoA 9-desaturase	1.14.19.1	Human	11677241
stearoyl-CoA 9-desaturase	1.14.19.1	Human	12364560
stearoyl-CoA 9-desaturase	1.14.19.1	Human	12815040

stearoyl-CoA 9-desaturase	1.14.19.1	Human	14610276
stearoyl-CoA 9-desaturase	1.14.19.1	Human	14654089
stearoyl-CoA 9-desaturase	1.14.19.1	Human	14734557
stearoyl-CoA 9-desaturase	1.14.19.1	Human	15096593
stearoyl-CoA 9-desaturase	1.14.19.1	Human	15180999
stearoyl-CoA 9-desaturase	1.14.19.1	Human	15556291
stearoyl-CoA 9-desaturase	1.14.19.1	Human	15726820
stearoyl-CoA 9-desaturase	1.14.19.1	Human	15761499
stearoyl-CoA 9-desaturase	1.14.19.1	Human	15829484
stearoyl-CoA 9-desaturase	1.14.19.1	Human	15836467
stearoyl-CoA 9-desaturase	1.14.19.1	Human	16245372
stearoyl-CoA 9-desaturase	1.14.19.1	Human	16767221
stearoyl-CoA 9-desaturase	1.14.19.1	Human	16804073
stearoyl-CoA 9-desaturase	1.14.19.1	Human	9272444
prostaglandin-endoperoxide synthase	1.14.99.1	Human	10024686
prostaglandin-endoperoxide synthase	1.14.99.1	Human	10091284
prostaglandin-endoperoxide synthase	1.14.99.1	Human	10226539
prostaglandin-endoperoxide synthase	1.14.99.1	Human	10228831
prostaglandin-endoperoxide synthase	1.14.99.1	Human	10365086
prostaglandin-endoperoxide synthase	1.14.99.1	Human	10395693
prostaglandin-endoperoxide synthase	1.14.99.1	Human	10399316
prostaglandin-endoperoxide synthase	1.14.99.1	Human	10425287
prostaglandin-endoperoxide synthase	1.14.99.1	Human	10426495
prostaglandin-endoperoxide synthase	1.14.99.1	Human	10560661
prostaglandin-endoperoxide synthase	1.14.99.1	Human	10652449
prostaglandin-endoperoxide synthase	1.14.99.1	Human	10667579
prostaglandin-endoperoxide synthase	1.14.99.1	Human	10754263
prostaglandin-endoperoxide synthase	1.14.99.1	Human	10807888
prostaglandin-endoperoxide synthase	1.14.99.1	Human	10859338
prostaglandin-endoperoxide synthase	1.14.99.1	Human	10935540
prostaglandin-endoperoxide synthase	1.14.99.1	Human	10991924
prostaglandin-endoperoxide synthase	1.14.99.1	Human	10999846
prostaglandin-endoperoxide synthase	1.14.99.1	Human	11153163
prostaglandin-endoperoxide synthase	1.14.99.1	Human	11192946
prostaglandin-endoperoxide synthase	1.14.99.1	Human	11220737
prostaglandin-endoperoxide synthase	1.14.99.1	Human	11239825
prostaglandin-endoperoxide synthase	1.14.99.1	Human	11303759
prostaglandin-endoperoxide synthase	1.14.99.1	Human	11338298
prostaglandin-endoperoxide synthase	1.14.99.1	Human	11338376
prostaglandin-endoperoxide synthase	1.14.99.1	Human	11407522
prostaglandin-endoperoxide synthase	1.14.99.1	Human	11427038
prostaglandin-endoperoxide synthase	1.14.99.1	Human	11451994
prostaglandin-endoperoxide synthase	1.14.99.1	Human	11455568

prostaglandin-endoperoxide synthase	1.14.99.1	Human	11501838
prostaglandin-endoperoxide synthase	1.14.99.1	Human	11506967
prostaglandin-endoperoxide synthase	1.14.99.1	Human	11519793
prostaglandin-endoperoxide synthase	1.14.99.1	Human	11520057
prostaglandin-endoperoxide synthase	1.14.99.1	Human	11588192
prostaglandin-endoperoxide synthase	1.14.99.1	Human	11673276
prostaglandin-endoperoxide synthase	1.14.99.1	Human	11746474
prostaglandin-endoperoxide synthase	1.14.99.1	Human	11758826
prostaglandin-endoperoxide synthase	1.14.99.1	Human	11781662
prostaglandin-endoperoxide synthase	1.14.99.1	Human	11857443
prostaglandin-endoperoxide synthase	1.14.99.1	Human	11870370
prostaglandin-endoperoxide synthase	1.14.99.1	Human	11945149
prostaglandin-endoperoxide synthase	1.14.99.1	Human	11966528
prostaglandin-endoperoxide synthase	1.14.99.1	Human	11991685
prostaglandin-endoperoxide synthase	1.14.99.1	Human	12021045
prostaglandin-endoperoxide synthase	1.14.99.1	Human	12034746
prostaglandin-endoperoxide synthase	1.14.99.1	Human	12047931
prostaglandin-endoperoxide synthase	1.14.99.1	Human	12211105
prostaglandin-endoperoxide synthase	1.14.99.1	Human	12242329
prostaglandin-endoperoxide synthase	1.14.99.1	Human	12349897
prostaglandin-endoperoxide synthase	1.14.99.1	Human	12352459
prostaglandin-endoperoxide synthase	1.14.99.1	Human	12377741
prostaglandin-endoperoxide synthase	1.14.99.1	Human	12397176
prostaglandin-endoperoxide synthase	1.14.99.1	Human	12414874
prostaglandin-endoperoxide synthase	1.14.99.1	Human	12432912
prostaglandin-endoperoxide synthase	1.14.99.1	Human	12467525
prostaglandin-endoperoxide synthase	1.14.99.1	Human	12468643
prostaglandin-endoperoxide synthase	1.14.99.1	Human	12491803
prostaglandin-endoperoxide synthase	1.14.99.1	Human	12502903
prostaglandin-endoperoxide synthase	1.14.99.1	Human	12576462
prostaglandin-endoperoxide synthase	1.14.99.1	Human	12604670
prostaglandin-endoperoxide synthase	1.14.99.1	Human	12614262
prostaglandin-endoperoxide synthase	1.14.99.1	Human	12669881
prostaglandin-endoperoxide synthase	1.14.99.1	Human	12683423
prostaglandin-endoperoxide synthase	1.14.99.1	Human	12735864
prostaglandin-endoperoxide synthase	1.14.99.1	Human	12845600
prostaglandin-endoperoxide synthase	1.14.99.1	Human	12846004
prostaglandin-endoperoxide synthase	1.14.99.1	Human	12885872
prostaglandin-endoperoxide synthase	1.14.99.1	Human	12969240
prostaglandin-endoperoxide synthase	1.14.99.1	Human	12970891
prostaglandin-endoperoxide synthase	1.14.99.1	Human	1305681
prostaglandin-endoperoxide synthase	1.14.99.1	Human	1316915
prostaglandin-endoperoxide synthase	1.14.99.1	Human	1400321

prostaglandin-endoperoxide synthase	1.14.99.1	Human	1410519
prostaglandin-endoperoxide synthase	1.14.99.1	Human	14518562
prostaglandin-endoperoxide synthase	1.14.99.1	Human	14566678
prostaglandin-endoperoxide synthase	1.14.99.1	Human	14634339
prostaglandin-endoperoxide synthase	1.14.99.1	Human	14704742
prostaglandin-endoperoxide synthase	1.14.99.1	Human	14707443
prostaglandin-endoperoxide synthase	1.14.99.1	Human	14743889
prostaglandin-endoperoxide synthase	1.14.99.1	Human	1480760
prostaglandin-endoperoxide synthase	1.14.99.1	Human	14965326
prostaglandin-endoperoxide synthase	1.14.99.1	Human	15037210
prostaglandin-endoperoxide synthase	1.14.99.1	Human	15106811
prostaglandin-endoperoxide synthase	1.14.99.1	Human	15109739
prostaglandin-endoperoxide synthase	1.14.99.1	Human	15148333
prostaglandin-endoperoxide synthase	1.14.99.1	Human	15205395
prostaglandin-endoperoxide synthase	1.14.99.1	Human	15299086
prostaglandin-endoperoxide synthase	1.14.99.1	Human	15351039
prostaglandin-endoperoxide synthase	1.14.99.1	Human	15486039
prostaglandin-endoperoxide synthase	1.14.99.1	Human	15496409
prostaglandin-endoperoxide synthase	1.14.99.1	Human	15507510
prostaglandin-endoperoxide synthase	1.14.99.1	Human	15596243
prostaglandin-endoperoxide synthase	1.14.99.1	Human	15604817
prostaglandin-endoperoxide synthase	1.14.99.1	Human	15639337
prostaglandin-endoperoxide synthase	1.14.99.1	Human	15653788
prostaglandin-endoperoxide synthase	1.14.99.1	Human	15668527
prostaglandin-endoperoxide synthase	1.14.99.1	Human	15753380
prostaglandin-endoperoxide synthase	1.14.99.1	Human	15809059
prostaglandin-endoperoxide synthase	1.14.99.1	Human	15821352
prostaglandin-endoperoxide synthase	1.14.99.1	Human	15885672
prostaglandin-endoperoxide synthase	1.14.99.1	Human	15889237
prostaglandin-endoperoxide synthase	1.14.99.1	Human	15900018
prostaglandin-endoperoxide synthase	1.14.99.1	Human	15992053
prostaglandin-endoperoxide synthase	1.14.99.1	Human	15993594
prostaglandin-endoperoxide synthase	1.14.99.1	Human	16046792
prostaglandin-endoperoxide synthase	1.14.99.1	Human	16137646
prostaglandin-endoperoxide synthase	1.14.99.1	Human	16169091
prostaglandin-endoperoxide synthase	1.14.99.1	Human	16169124
prostaglandin-endoperoxide synthase	1.14.99.1	Human	16253229
prostaglandin-endoperoxide synthase	1.14.99.1	Human	16284883
prostaglandin-endoperoxide synthase	1.14.99.1	Human	16445867
prostaglandin-endoperoxide synthase	1.14.99.1	Human	16458279
prostaglandin-endoperoxide synthase	1.14.99.1	Human	16493584
prostaglandin-endoperoxide synthase	1.14.99.1	Human	16516846
prostaglandin-endoperoxide synthase	1.14.99.1	Human	16529558

prostaglandin-endoperoxide synthase	1.14.99.1	Human	16709833
prostaglandin-endoperoxide synthase	1.14.99.1	Human	16716827
prostaglandin-endoperoxide synthase	1.14.99.1	Human	16740978
prostaglandin-endoperoxide synthase	1.14.99.1	Human	16753269
prostaglandin-endoperoxide synthase	1.14.99.1	Human	16827136
prostaglandin-endoperoxide synthase	1.14.99.1	Human	16840740
prostaglandin-endoperoxide synthase	1.14.99.1	Human	16842132
prostaglandin-endoperoxide synthase	1.14.99.1	Human	16842185
prostaglandin-endoperoxide synthase	1.14.99.1	Human	16847764
prostaglandin-endoperoxide synthase	1.14.99.1	Human	16870271
prostaglandin-endoperoxide synthase	1.14.99.1	Human	16871410
prostaglandin-endoperoxide synthase	1.14.99.1	Human	16872525
prostaglandin-endoperoxide synthase	1.14.99.1	Human	16885386
prostaglandin-endoperoxide synthase	1.14.99.1	Human	1694171
prostaglandin-endoperoxide synthase	1.14.99.1	Human	16966442
prostaglandin-endoperoxide synthase	1.14.99.1	Human	1706264
prostaglandin-endoperoxide synthase	1.14.99.1	Human	17066444
prostaglandin-endoperoxide synthase	1.14.99.1	Human	17082565
prostaglandin-endoperoxide synthase	1.14.99.1	Human	17140386
prostaglandin-endoperoxide synthase	1.14.99.1	Human	1721064
prostaglandin-endoperoxide synthase	1.14.99.1	Human	1730539
prostaglandin-endoperoxide synthase	1.14.99.1	Human	1731622
prostaglandin-endoperoxide synthase	1.14.99.1	Human	1744122
prostaglandin-endoperoxide synthase	1.14.99.1	Human	1836009
prostaglandin-endoperoxide synthase	1.14.99.1	Human	1903222
prostaglandin-endoperoxide synthase	1.14.99.1	Human	2125559
prostaglandin-endoperoxide synthase	1.14.99.1	Human	2150736
prostaglandin-endoperoxide synthase	1.14.99.1	Human	2497108
prostaglandin-endoperoxide synthase	1.14.99.1	Human	2519899
prostaglandin-endoperoxide synthase	1.14.99.1	Human	26389
prostaglandin-endoperoxide synthase	1.14.99.1	Human	2770427
prostaglandin-endoperoxide synthase	1.14.99.1	Human	3040660
prostaglandin-endoperoxide synthase	1.14.99.1	Human	3510842
prostaglandin-endoperoxide synthase	1.14.99.1	Human	6197028
prostaglandin-endoperoxide synthase	1.14.99.1	Human	6252692
prostaglandin-endoperoxide synthase	1.14.99.1	Human	6312489
prostaglandin-endoperoxide synthase	1.14.99.1	Human	6324240
prostaglandin-endoperoxide synthase	1.14.99.1	Human	663276
prostaglandin-endoperoxide synthase	1.14.99.1	Human	7229430
prostaglandin-endoperoxide synthase	1.14.99.1	Human	7635765
prostaglandin-endoperoxide synthase	1.14.99.1	Human	7706373
prostaglandin-endoperoxide synthase	1.14.99.1	Human	7775455
prostaglandin-endoperoxide synthase	1.14.99.1	Human	7784470

prostaglandin-endoperoxide synthase	1.14.99.1	Human	7920026
prostaglandin-endoperoxide synthase	1.14.99.1	Human	7926468
prostaglandin-endoperoxide synthase	1.14.99.1	Human	7980528
prostaglandin-endoperoxide synthase	1.14.99.1	Human	8132578
prostaglandin-endoperoxide synthase	1.14.99.1	Human	8163473
prostaglandin-endoperoxide synthase	1.14.99.1	Human	8188473
prostaglandin-endoperoxide synthase	1.14.99.1	Human	8261019
prostaglandin-endoperoxide synthase	1.14.99.1	Human	8280164
prostaglandin-endoperoxide synthase	1.14.99.1	Human	8365485
prostaglandin-endoperoxide synthase	1.14.99.1	Human	8521479
prostaglandin-endoperoxide synthase	1.14.99.1	Human	8616915
prostaglandin-endoperoxide synthase	1.14.99.1	Human	8641206
prostaglandin-endoperoxide synthase	1.14.99.1	Human	8653697
prostaglandin-endoperoxide synthase	1.14.99.1	Human	8752493
prostaglandin-endoperoxide synthase	1.14.99.1	Human	8918053
prostaglandin-endoperoxide synthase	1.14.99.1	Human	9013627
prostaglandin-endoperoxide synthase	1.14.99.1	Human	9057838
prostaglandin-endoperoxide synthase	1.14.99.1	Human	9096394
prostaglandin-endoperoxide synthase	1.14.99.1	Human	9102213
prostaglandin-endoperoxide synthase	1.14.99.1	Human	9126611
prostaglandin-endoperoxide synthase	1.14.99.1	Human	9144511
prostaglandin-endoperoxide synthase	1.14.99.1	Human	9177102
prostaglandin-endoperoxide synthase	1.14.99.1	Human	9177247
prostaglandin-endoperoxide synthase	1.14.99.1	Human	9261162
prostaglandin-endoperoxide synthase	1.14.99.1	Human	9360765
prostaglandin-endoperoxide synthase	1.14.99.1	Human	9373878
prostaglandin-endoperoxide synthase	1.14.99.1	Human	9383435
prostaglandin-endoperoxide synthase	1.14.99.1	Human	9402302
prostaglandin-endoperoxide synthase	1.14.99.1	Human	9458802
prostaglandin-endoperoxide synthase	1.14.99.1	Human	9483916
prostaglandin-endoperoxide synthase	1.14.99.1	Human	9492015
prostaglandin-endoperoxide synthase	1.14.99.1	Human	9513902
prostaglandin-endoperoxide synthase	1.14.99.1	Human	9517759
prostaglandin-endoperoxide synthase	1.14.99.1	Human	9521170
prostaglandin-endoperoxide synthase	1.14.99.1	Human	9526843
prostaglandin-endoperoxide synthase	1.14.99.1	Human	9562240
prostaglandin-endoperoxide synthase	1.14.99.1	Human	9568703
prostaglandin-endoperoxide synthase	1.14.99.1	Human	9585093
prostaglandin-endoperoxide synthase	1.14.99.1	Human	9649571
prostaglandin-endoperoxide synthase	1.14.99.1	Human	97337
prostaglandin-endoperoxide synthase	1.14.99.1	Human	9740394
prostaglandin-endoperoxide synthase	1.14.99.1	Human	9758208
prostaglandin-endoperoxide synthase	1.14.99.1	Human	9764845

prostaglandin-endoperoxide synthase	1.14.99.1	Human	9790545
prostaglandin-endoperoxide synthase	1.14.99.1	Human	9811310
prostaglandin-endoperoxide synthase	1.14.99.1	Human	9823297
prostaglandin-endoperoxide synthase	1.14.99.1	Human	9870924
prostaglandin-endoperoxide synthase	1.14.99.1	Human	9879525
heme oxygenase	1.14.99.3	Human	10349844
heme oxygenase	1.14.99.3	Human	10353745
heme oxygenase	1.14.99.3	Human	10630670
heme oxygenase	1.14.99.3	Human	10634305
heme oxygenase	1.14.99.3	Human	10644516
heme oxygenase	1.14.99.3	Human	10692503
heme oxygenase	1.14.99.3	Human	10727990
heme oxygenase	1.14.99.3	Human	10793064
heme oxygenase	1.14.99.3	Human	10814519
heme oxygenase	1.14.99.3	Human	10961657
heme oxygenase	1.14.99.3	Human	11007950
heme oxygenase	1.14.99.3	Human	11135063
heme oxygenase	1.14.99.3	Human	11258550
heme oxygenase	1.14.99.3	Human	11498995
heme oxygenase	1.14.99.3	Human	11591199
heme oxygenase	1.14.99.3	Human	11592943
heme oxygenase	1.14.99.3	Human	11698254
heme oxygenase	1.14.99.3	Human	11705458
heme oxygenase	1.14.99.3	Human	11758836
heme oxygenase	1.14.99.3	Human	11801258
heme oxygenase	1.14.99.3	Human	11803410
heme oxygenase	1.14.99.3	Human	11868390
heme oxygenase	1.14.99.3	Human	11978880
heme oxygenase	1.14.99.3	Human	12042070
heme oxygenase	1.14.99.3	Human	12057765
heme oxygenase	1.14.99.3	Human	12117910
heme oxygenase	1.14.99.3	Human	12136229
heme oxygenase	1.14.99.3	Human	12230871
heme oxygenase	1.14.99.3	Human	12236785
heme oxygenase	1.14.99.3	Human	12372789
heme oxygenase	1.14.99.3	Human	12372951
heme oxygenase	1.14.99.3	Human	12397597
heme oxygenase	1.14.99.3	Human	12489116
heme oxygenase	1.14.99.3	Human	12508080
heme oxygenase	1.14.99.3	Human	12581208
heme oxygenase	1.14.99.3	Human	12646399
heme oxygenase	1.14.99.3	Human	12668974
heme oxygenase	1.14.99.3	Human	12709591

heme oxygenase	1.14.99.3	Human	12736395
heme oxygenase	1.14.99.3	Human	12757849
heme oxygenase	1.14.99.3	Human	12783778
heme oxygenase	1.14.99.3	Human	12874251
heme oxygenase	1.14.99.3	Human	12892830
heme oxygenase	1.14.99.3	Human	12963497
heme oxygenase	1.14.99.3	Human	14523007
heme oxygenase	1.14.99.3	Human	14649057
heme oxygenase	1.14.99.3	Human	14683741
heme oxygenase	1.14.99.3	Human	14761930
heme oxygenase	1.14.99.3	Human	14766239
heme oxygenase	1.14.99.3	Human	14985830
heme oxygenase	1.14.99.3	Human	15018736
heme oxygenase	1.14.99.3	Human	15276480
heme oxygenase	1.14.99.3	Human	15298625
heme oxygenase	1.14.99.3	Human	15345139
heme oxygenase	1.14.99.3	Human	15345147
heme oxygenase	1.14.99.3	Human	15345149
heme oxygenase	1.14.99.3	Human	15499042
heme oxygenase	1.14.99.3	Human	15499991
heme oxygenase	1.14.99.3	Human	15565657
heme oxygenase	1.14.99.3	Human	15588929
heme oxygenase	1.14.99.3	Human	15599843
heme oxygenase	1.14.99.3	Human	15649645
heme oxygenase	1.14.99.3	Human	1572205
heme oxygenase	1.14.99.3	Human	15833736
heme oxygenase	1.14.99.3	Human	15869055
heme oxygenase	1.14.99.3	Human	15896346
heme oxygenase	1.14.99.3	Human	15897578
heme oxygenase	1.14.99.3	Human	15933765
heme oxygenase	1.14.99.3	Human	16043027
heme oxygenase	1.14.99.3	Human	16048902
heme oxygenase	1.14.99.3	Human	16123320
heme oxygenase	1.14.99.3	Human	16181105
heme oxygenase	1.14.99.3	Human	16181109
heme oxygenase	1.14.99.3	Human	16214041
heme oxygenase	1.14.99.3	Human	16276181
heme oxygenase	1.14.99.3	Human	16309569
heme oxygenase	1.14.99.3	Human	16319139
heme oxygenase	1.14.99.3	Human	16374439
heme oxygenase	1.14.99.3	Human	16476737
heme oxygenase	1.14.99.3	Human	16598857
heme oxygenase	1.14.99.3	Human	16678019

heme oxygenase	1.14.99.3	Human	16775600
heme oxygenase	1.14.99.3	Human	16828975
heme oxygenase	1.14.99.3	Human	16858012
heme oxygenase	1.14.99.3	Human	16948473
heme oxygenase	1.14.99.3	Human	16964402
heme oxygenase	1.14.99.3	Human	16966352
heme oxygenase	1.14.99.3	Human	17002867
heme oxygenase	1.14.99.3	Human	1700666
heme oxygenase	1.14.99.3	Human	17042977
heme oxygenase	1.14.99.3	Human	1737989
heme oxygenase	1.14.99.3	Human	1884769
heme oxygenase	1.14.99.3	Human	2759552
heme oxygenase	1.14.99.3	Human	3107908
heme oxygenase	1.14.99.3	Human	3113993
heme oxygenase	1.14.99.3	Human	3177434
heme oxygenase	1.14.99.3	Human	3965329
heme oxygenase	1.14.99.3	Human	6548384
heme oxygenase	1.14.99.3	Human	7525927
heme oxygenase	1.14.99.3	Human	7576696
heme oxygenase	1.14.99.3	Human	7626076
heme oxygenase	1.14.99.3	Human	7768976
heme oxygenase	1.14.99.3	Human	7793979
heme oxygenase	1.14.99.3	Human	7889361
heme oxygenase	1.14.99.3	Human	8016102
heme oxygenase	1.14.99.3	Human	8216309
heme oxygenase	1.14.99.3	Human	8548408
heme oxygenase	1.14.99.3	Human	8564848
heme oxygenase	1.14.99.3	Human	8590277
heme oxygenase	1.14.99.3	Human	8694803
heme oxygenase	1.14.99.3	Human	8774698
heme oxygenase	1.14.99.3	Human	8816811
heme oxygenase	1.14.99.3	Human	8827779
heme oxygenase	1.14.99.3	Human	8852585
heme oxygenase	1.14.99.3	Human	8872603
heme oxygenase	1.14.99.3	Human	8897916
heme oxygenase	1.14.99.3	Human	8913885
heme oxygenase	1.14.99.3	Human	9125512
heme oxygenase	1.14.99.3	Human	9225984
heme oxygenase	1.14.99.3	Human	9236721
heme oxygenase	1.14.99.3	Human	9259978
heme oxygenase	1.14.99.3	Human	9276739
heme oxygenase	1.14.99.3	Human	9495815
heme oxygenase	1.14.99.3	Human	9530200

heme oxygenase	1.14.99.3	Human	9608682
heme oxygenase	1.14.99.3	Human	9927151
squalene monooxygenase	1.14.99.7	Human	10679280
squalene monooxygenase	1.14.99.7	Human	10733917
squalene monooxygenase	1.14.99.7	Human	11199136
squalene monooxygenase	1.14.99.7	Human	11520216
squalene monooxygenase	1.14.99.7	Human	12226513
squalene monooxygenase	1.14.99.7	Human	16466954
squalene monooxygenase	1.14.99.7	Human	6087072
squalene monooxygenase	1.14.99.7	Human	6547247
squalene monooxygenase	1.14.99.7	Human	8771716
squalene monooxygenase	1.14.99.7	Human	9017503
steroid 17alpha-monooxygenase	1.14.99.9	Human	10066888
steroid 17alpha-monooxygenase	1.14.99.9	Human	10352919
steroid 17alpha-monooxygenase	1.14.99.9	Human	10574247
steroid 17alpha-monooxygenase	1.14.99.9	Human	11446160
steroid 17alpha-monooxygenase	1.14.99.9	Human	11817165
steroid 17alpha-monooxygenase	1.14.99.9	Human	12444467
steroid 17alpha-monooxygenase	1.14.99.9	Human	15001547
steroid 17alpha-monooxygenase	1.14.99.9	Human	16022662
steroid 17alpha-monooxygenase	1.14.99.9	Human	1605399
steroid 17alpha-monooxygenase	1.14.99.9	Human	1607640
steroid 17alpha-monooxygenase	1.14.99.9	Human	1958544
steroid 17alpha-monooxygenase	1.14.99.9	Human	2019257
steroid 17alpha-monooxygenase	1.14.99.9	Human	3502608
steroid 17alpha-monooxygenase	1.14.99.9	Human	6593324 cytochrome P450
steroid 17alpha-monooxygenase	1.14.99.9	Human	9115645
steroid 17alpha-monooxygenase	1.14.99.9	Human	9408743
steroid 17alpha-monooxygenase	1.14.99.9	Human	9892022
xanthine dehydrogenase	1.17.1.4	Human	11086257
xanthine dehydrogenase	1.17.1.4	Human	11154741
xanthine dehydrogenase	1.17.1.4	Human	12502743
xanthine oxidase	1.17.3.2	Human	11086257
xanthine oxidase	1.17.3.2	Human	11278616
xanthine oxidase	1.17.3.2	Human	12423257
xanthine oxidase	1.17.3.2	Human	12618887
xanthine oxidase	1.17.3.2	Human	12780970
xanthine oxidase	1.17.3.2	Human	1353938
xanthine oxidase	1.17.3.2	Human	16429783
xanthine oxidase	1.17.3.2	Human	187329
xanthine oxidase	1.17.3.2	Human	1889832
xanthine oxidase	1.17.3.2	Human	3245099

xanthine oxidase	1.17.3.2	Human	8274165
ribonucleoside-diphosphate reductase	1.17.4.1	Human	10441745
ribonucleoside-diphosphate reductase	1.17.4.1	Human	10593972
ribonucleoside-diphosphate reductase	1.17.4.1	Human	10769119
ribonucleoside-diphosphate reductase	1.17.4.1	Human	10805162
ribonucleoside-diphosphate reductase	1.17.4.1	Human	10953295
ribonucleoside-diphosphate reductase	1.17.4.1	Human	10989193
ribonucleoside-diphosphate reductase	1.17.4.1	Human	11489836
ribonucleoside-diphosphate reductase	1.17.4.1	Human	11551528
ribonucleoside-diphosphate reductase	1.17.4.1	Human	11904430
ribonucleoside-diphosphate reductase	1.17.4.1	Human	12147300
ribonucleoside-diphosphate reductase	1.17.4.1	Human	12359454
ribonucleoside-diphosphate reductase	1.17.4.1	Human	12655059
ribonucleoside-diphosphate reductase	1.17.4.1	Human	12690517
ribonucleoside-diphosphate reductase	1.17.4.1	Human	12732713
ribonucleoside-diphosphate reductase	1.17.4.1	Human	12749906
ribonucleoside-diphosphate reductase	1.17.4.1	Human	12967138
ribonucleoside-diphosphate reductase	1.17.4.1	Human	1299271
ribonucleoside-diphosphate reductase	1.17.4.1	Human	1385411
ribonucleoside-diphosphate reductase	1.17.4.1	Human	1412696
ribonucleoside-diphosphate reductase	1.17.4.1	Human	14963934
ribonucleoside-diphosphate reductase	1.17.4.1	Human	14966112
ribonucleoside-diphosphate reductase	1.17.4.1	Human	1496919
ribonucleoside-diphosphate reductase	1.17.4.1	Human	15094776
ribonucleoside-diphosphate reductase	1.17.4.1	Human	15133626
ribonucleoside-diphosphate reductase	1.17.4.1	Human	1516817
ribonucleoside-diphosphate reductase	1.17.4.1	Human	15300180
ribonucleoside-diphosphate reductase	1.17.4.1	Human	15571292
ribonucleoside-diphosphate reductase	1.17.4.1	Human	15656518
ribonucleoside-diphosphate reductase	1.17.4.1	Human	15673563
ribonucleoside-diphosphate reductase	1.17.4.1	Human	15730856
ribonucleoside-diphosphate reductase	1.17.4.1	Human	15769467
ribonucleoside-diphosphate reductase	1.17.4.1	Human	15803490
ribonucleoside-diphosphate reductase	1.17.4.1	Human	15805194
ribonucleoside-diphosphate reductase	1.17.4.1	Human	15888728
ribonucleoside-diphosphate reductase	1.17.4.1	Human	160558
ribonucleoside-diphosphate reductase	1.17.4.1	Human	16399800
ribonucleoside-diphosphate reductase	1.17.4.1	Human	1643157
ribonucleoside-diphosphate reductase	1.17.4.1	Human	16489218
ribonucleoside-diphosphate reductase	1.17.4.1	Human	16530987
ribonucleoside-diphosphate reductase	1.17.4.1	Human	16834759
ribonucleoside-diphosphate reductase	1.17.4.1	Human	16925573
ribonucleoside-diphosphate reductase	1.17.4.1	Human	17065057

ribonucleoside-diphosphate reductase	1.17.4.1	Human	1717630
ribonucleoside-diphosphate reductase	1.17.4.1	Human	1748682
ribonucleoside-diphosphate reductase	1.17.4.1	Human	1793565
ribonucleoside-diphosphate reductase	1.17.4.1	Human	2085432
ribonucleoside-diphosphate reductase	1.17.4.1	Human	2178608
ribonucleoside-diphosphate reductase	1.17.4.1	Human	2199320
ribonucleoside-diphosphate reductase	1.17.4.1	Human	2257322
ribonucleoside-diphosphate reductase	1.17.4.1	Human	2642388
ribonucleoside-diphosphate reductase	1.17.4.1	Human	2673261
ribonucleoside-diphosphate reductase	1.17.4.1	Human	2775821
ribonucleoside-diphosphate reductase	1.17.4.1	Human	2827767
ribonucleoside-diphosphate reductase	1.17.4.1	Human	2832057
ribonucleoside-diphosphate reductase	1.17.4.1	Human	3044371
ribonucleoside-diphosphate reductase	1.17.4.1	Human	3044582
ribonucleoside-diphosphate reductase	1.17.4.1	Human	3061459
ribonucleoside-diphosphate reductase	1.17.4.1	Human	3300645
ribonucleoside-diphosphate reductase	1.17.4.1	Human	3511848
ribonucleoside-diphosphate reductase	1.17.4.1	Human	3536076
ribonucleoside-diphosphate reductase	1.17.4.1	Human	3907637
ribonucleoside-diphosphate reductase	1.17.4.1	Human	3914643
ribonucleoside-diphosphate reductase	1.17.4.1	Human	3915189
ribonucleoside-diphosphate reductase	1.17.4.1	Human	3986794
ribonucleoside-diphosphate reductase	1.17.4.1	Human	6353195
ribonucleoside-diphosphate reductase	1.17.4.1	Human	6375753
ribonucleoside-diphosphate reductase	1.17.4.1	Human	6752137
ribonucleoside-diphosphate reductase	1.17.4.1	Human	6757589
ribonucleoside-diphosphate reductase	1.17.4.1	Human	767333
ribonucleoside-diphosphate reductase	1.17.4.1	Human	7727399
ribonucleoside-diphosphate reductase	1.17.4.1	Human	7768988
ribonucleoside-diphosphate reductase	1.17.4.1	Human	7838172
ribonucleoside-diphosphate reductase	1.17.4.1	Human	7881162
ribonucleoside-diphosphate reductase	1.17.4.1	Human	7882331
ribonucleoside-diphosphate reductase	1.17.4.1	Human	7893463
ribonucleoside-diphosphate reductase	1.17.4.1	Human	7937896
ribonucleoside-diphosphate reductase	1.17.4.1	Human	7984431
ribonucleoside-diphosphate reductase	1.17.4.1	Human	8241321
ribonucleoside-diphosphate reductase	1.17.4.1	Human	8265664
ribonucleoside-diphosphate reductase	1.17.4.1	Human	8343143
ribonucleoside-diphosphate reductase	1.17.4.1	Human	8463252
ribonucleoside-diphosphate reductase	1.17.4.1	Human	8521087
ribonucleoside-diphosphate reductase	1.17.4.1	Human	8620054
ribonucleoside-diphosphate reductase	1.17.4.1	Human	8662944
ribonucleoside-diphosphate reductase	1.17.4.1	Human	8674535

ribonucleoside-diphosphate reductase	1.17.4.1	Human	8813126
ribonucleoside-diphosphate reductase	1.17.4.1	Human	8878781
ribonucleoside-diphosphate reductase	1.17.4.1	Human	8920917
ribonucleoside-diphosphate reductase	1.17.4.1	Human	8943056
ribonucleoside-diphosphate reductase	1.17.4.1	Human	9192674
ribonucleoside-diphosphate reductase	1.17.4.1	Human	9315670
ribonucleoside-diphosphate reductase	1.17.4.1	Human	9347313
ribonucleoside-diphosphate reductase	1.17.4.1	Human	9354452
ribonucleoside-diphosphate reductase	1.17.4.1	Human	9371820
ribonucleoside-diphosphate reductase	1.17.4.1	Human	9393942
ribonucleoside-diphosphate reductase	1.17.4.1	Human	9415718
ribonucleoside-diphosphate reductase	1.17.4.1	Human	9439883
ribonucleoside-diphosphate reductase	1.17.4.1	Human	9558318
ribonucleoside-diphosphate reductase	1.17.4.1	Human	9570515
ribonucleoside-diphosphate reductase	1.17.4.1	Human	9598136
ribonucleoside-diphosphate reductase	1.17.4.1	Human	9605773
ribonucleoside-diphosphate reductase	1.17.4.1	Human	9634002
ribonucleoside-diphosphate reductase	1.17.4.1	Human	9696008
ribonucleoside-diphosphate reductase	1.17.4.1	Human	9718080
ribonucleoside-diphosphate reductase	1.17.4.1	Human	9852067
ribonucleoside-diphosphate reductase	1.17.4.1	Human	9990288
glyceraldehyde-3-phosphate dehydrogenase (phosphorylating)	1.2.1.12	Human	10966377
glyceraldehyde-3-phosphate dehydrogenase (phosphorylating)	1.2.1.12	Human	11018719
glyceraldehyde-3-phosphate dehydrogenase (phosphorylating)	1.2.1.12	Human	12123463
glyceraldehyde-3-phosphate dehydrogenase (phosphorylating)	1.2.1.12	Human	12634343
glyceraldehyde-3-phosphate dehydrogenase (phosphorylating)	1.2.1.12	Human	14502604
glyceraldehyde-3-phosphate dehydrogenase (phosphorylating)	1.2.1.12	Human	15299328
glyceraldehyde-3-phosphate dehydrogenase (phosphorylating)	1.2.1.12	Human	15631980
glyceraldehyde-3-phosphate dehydrogenase (phosphorylating)	1.2.1.12	Human	15680915
glyceraldehyde-3-phosphate dehydrogenase (phosphorylating)	1.2.1.12	Human	3530169
glyceraldehyde-3-phosphate dehydrogenase (phosphorylating)	1.2.1.12	Human	6095107
glyceraldehyde-3-phosphate dehydrogenase (phosphorylating)	1.2.1.12	Human	6440018

glyceraldehyde-3-phosphate dehydrogenase (phosphorylating)	1.2.1.12	Human	664114
glyceraldehyde-3-phosphate dehydrogenase (phosphorylating)	1.2.1.12	Human	7165719
glyceraldehyde-3-phosphate dehydrogenase (phosphorylating)	1.2.1.12	Human	7173737
glyceraldehyde-3-phosphate dehydrogenase (phosphorylating)	1.2.1.12	Human	8304415
glyceraldehyde-3-phosphate dehydrogenase (phosphorylating)	1.2.1.12	Human	8454610
glyceraldehyde-3-phosphate dehydrogenase (phosphorylating)	1.2.1.12	Human	8791005
glyceraldehyde-3-phosphate dehydrogenase (phosphorylating)	1.2.1.12	Human	8805794
glyceraldehyde-3-phosphate dehydrogenase (phosphorylating)	1.2.1.12	Human	8905295
glyceraldehyde-3-phosphate dehydrogenase (phosphorylating)	1.2.1.12	Human	9437188
glyceraldehyde-3-phosphate dehydrogenase (phosphorylating)	1.2.1.12	Human	9793073
glyceraldehyde-3-phosphate dehydrogenase (phosphorylating)	1.2.1.12	Human	9922941
aldehyde dehydrogenase (NAD+)	1.2.1.3	Human	10913633
aldehyde dehydrogenase (NAD+)	1.2.1.3	Human	11306027
aldehyde dehydrogenase (NAD+)	1.2.1.3	Human	12223435
aldehyde dehydrogenase (NAD+)	1.2.1.3	Human	14597338
aldehyde dehydrogenase (NAD+)	1.2.1.3	Human	1472111
aldehyde dehydrogenase (NAD+)	1.2.1.3	Human	16126235
aldehyde dehydrogenase (NAD+)	1.2.1.3	Human	16499490
aldehyde dehydrogenase (NAD+)	1.2.1.3	Human	16878979
aldehyde dehydrogenase (NAD+)	1.2.1.3	Human	17607160
aldehyde dehydrogenase (NAD+)	1.2.1.3	Human	2332351
aldehyde dehydrogenase (NAD+)	1.2.1.3	Human	3593277
aldehyde dehydrogenase (NAD+)	1.2.1.3	Human	6603890
aldehyde dehydrogenase (NAD+)	1.2.1.3	Human	7910607
aldehyde dehydrogenase (NAD+)	1.2.1.3	Human	8452208
aldehyde dehydrogenase (NAD+)	1.2.1.3	Human	8823154
aldehyde dehydrogenase (NAD+)	1.2.1.3	Human	8850269
aldehyde dehydrogenase (NAD+)	1.2.1.3	Human	9013560
aldehyde dehydrogenase (NAD+)	1.2.1.3	Human	9228057
aldehyde dehydrogenase (NAD+)	1.2.1.3	Human	945270
retinal dehydrogenase	1.2.1.36	Human	11600826
retinal dehydrogenase	1.2.1.36	Human	12547725

retinal dehydrogenase	1.2.1.36	Human	16763553
retinal dehydrogenase	1.2.1.36	Human	17098734
retinal dehydrogenase	1.2.1.36	Human	2910311
pyruvate dehydrogenase (acetyl-transferring)	1.2.4.1	Human	10720420
pyruvate dehydrogenase (acetyl-transferring)	1.2.4.1	Human	10759582
pyruvate dehydrogenase (acetyl-transferring)	1.2.4.1	Human	10826498
pyruvate dehydrogenase (acetyl-transferring)	1.2.4.1	Human	11223534
pyruvate dehydrogenase (acetyl-transferring)	1.2.4.1	Human	11427685
pyruvate dehydrogenase (acetyl-transferring)	1.2.4.1	Human	11557577
pyruvate dehydrogenase (acetyl-transferring)	1.2.4.1	Human	11866475
pyruvate dehydrogenase (acetyl-transferring)	1.2.4.1	Human	11900279
pyruvate dehydrogenase (acetyl-transferring)	1.2.4.1	Human	12196468
pyruvate dehydrogenase (acetyl-transferring)	1.2.4.1	Human	12557749
pyruvate dehydrogenase (acetyl-transferring)	1.2.4.1	Human	12663261
pyruvate dehydrogenase (acetyl-transferring)	1.2.4.1	Human	14607783
pyruvate dehydrogenase (acetyl-transferring)	1.2.4.1	Human	14641018
pyruvate dehydrogenase (acetyl-transferring)	1.2.4.1	Human	15191896
pyruvate dehydrogenase (acetyl-transferring)	1.2.4.1	Human	15256563
pyruvate dehydrogenase (acetyl-transferring)	1.2.4.1	Human	15921824
pyruvate dehydrogenase (acetyl-transferring)	1.2.4.1	Human	1600837
pyruvate dehydrogenase (acetyl-transferring)	1.2.4.1	Human	17065338
pyruvate dehydrogenase (acetyl-transferring)	1.2.4.1	Human	2902801
pyruvate dehydrogenase (acetyl-transferring)	1.2.4.1	Human	3127087
pyruvate dehydrogenase (acetyl-transferring)	1.2.4.1	Human	3918587
pyruvate dehydrogenase (acetyl-transferring)	1.2.4.1	Human	4030556
pyruvate dehydrogenase (acetyl-transferring)	1.2.4.1	Human	6149743
pyruvate dehydrogenase (acetyl-transferring)	1.2.4.1	Human	6234885
pyruvate dehydrogenase (acetyl-transferring)	1.2.4.1	Human	7127258
pyruvate dehydrogenase (acetyl-transferring)	1.2.4.1	Human	7848280
pyruvate dehydrogenase (acetyl-transferring)	1.2.4.1	Human	7864215
pyruvate dehydrogenase (acetyl-transferring)	1.2.4.1	Human	8557765
pyruvate dehydrogenase (acetyl-transferring)	1.2.4.1	Human	9089387
pyruvate dehydrogenase (acetyl-transferring)	1.2.4.1	Human	9381974
pyruvate dehydrogenase (acetyl-transferring)	1.2.4.1	Human	9426381
pyruvate dehydrogenase (acetyl-transferring)	1.2.4.1	Human	9438382
pyruvate dehydrogenase (acetyl-transferring)	1.2.4.1	Human	9497164
pyruvate dehydrogenase (acetyl-transferring)	1.2.4.1	Human	9725804
oxoglutarate dehydrogenase (succinyl-transferring)	1.2.4.2	Human	1352447
oxoglutarate dehydrogenase (succinyl-transferring)	1.2.4.2	Human	15356188
oxoglutarate dehydrogenase (succinyl-transferring)	1.2.4.2	Human	15466852

oxoglutarate dehydrogenase (succinyl-transferring)	1.2.4.2	Human	423807
oxoglutarate dehydrogenase (succinyl-transferring)	1.2.4.2	Human	6778477
oxoglutarate dehydrogenase (succinyl-transferring)	1.2.4.2	Human	7995856
3-methyl-2-oxobutanoate dehydrogenase	1.2.4.4	Human	10562606
3-methyl-2-oxobutanoate dehydrogenase	1.2.4.4	Human	11385053
3-methyl-2-oxobutanoate dehydrogenase	1.2.4.4	Human	17656140
3-methyl-2-oxobutanoate dehydrogenase	1.2.4.4	Human	3343251
3-methyl-2-oxobutanoate dehydrogenase	1.2.4.4	Human	4054446
3-methyl-2-oxobutanoate dehydrogenase	1.2.4.4	Human	6510417
3-methyl-2-oxobutanoate dehydrogenase	1.2.4.4	Human	9460082
enoyl-[acyl-carrier-protein] reductase (NADPH, B-specific)	1.3.1.10	Human	17095231
enoyl-[acyl-carrier-protein] reductase (NADPH, B-specific)	1.3.1.10	Human	8075395
dihydropyrimidine dehydrogenase (NADP+)	1.3.1.2	Human	10348793
dihydropyrimidine dehydrogenase (NADP+)	1.3.1.2	Human	10473079
dihydropyrimidine dehydrogenase (NADP+)	1.3.1.2	Human	10848981
dihydropyrimidine dehydrogenase (NADP+)	1.3.1.2	Human	10853015
dihydropyrimidine dehydrogenase (NADP+)	1.3.1.2	Human	10887632
dihydropyrimidine dehydrogenase (NADP+)	1.3.1.2	Human	10897217
dihydropyrimidine dehydrogenase (NADP+)	1.3.1.2	Human	11060767
dihydropyrimidine dehydrogenase (NADP+)	1.3.1.2	Human	11098485
dihydropyrimidine dehydrogenase (NADP+)	1.3.1.2	Human	11219978
dihydropyrimidine dehydrogenase (NADP+)	1.3.1.2	Human	11267945
dihydropyrimidine dehydrogenase (NADP+)	1.3.1.2	Human	11334264
dihydropyrimidine dehydrogenase (NADP+)	1.3.1.2	Human	11376565
dihydropyrimidine dehydrogenase (NADP+)	1.3.1.2	Human	11445849
dihydropyrimidine dehydrogenase (NADP+)	1.3.1.2	Human	11555593
dihydropyrimidine dehydrogenase (NADP+)	1.3.1.2	Human	11555601
dihydropyrimidine dehydrogenase (NADP+)	1.3.1.2	Human	11697836
dihydropyrimidine dehydrogenase (NADP+)	1.3.1.2	Human	11862480
dihydropyrimidine dehydrogenase (NADP+)	1.3.1.2	Human	11865631
dihydropyrimidine dehydrogenase (NADP+)	1.3.1.2	Human	11936689
dihydropyrimidine dehydrogenase (NADP+)	1.3.1.2	Human	11962674
dihydropyrimidine dehydrogenase (NADP+)	1.3.1.2	Human	12084458
dihydropyrimidine dehydrogenase (NADP+)	1.3.1.2	Human	12187768
dihydropyrimidine dehydrogenase (NADP+)	1.3.1.2	Human	12469154
dihydropyrimidine dehydrogenase (NADP+)	1.3.1.2	Human	12527935
dihydropyrimidine dehydrogenase (NADP+)	1.3.1.2	Human	12616366
dihydropyrimidine dehydrogenase (NADP+)	1.3.1.2	Human	12820455

dihydropyrimidine dehydrogenase (NADP+)	1.3.1.2	Human	12885330
dihydropyrimidine dehydrogenase (NADP+)	1.3.1.2	Human	12967482
dihydropyrimidine dehydrogenase (NADP+)	1.3.1.2	Human	14689231
dihydropyrimidine dehydrogenase (NADP+)	1.3.1.2	Human	14744810
dihydropyrimidine dehydrogenase (NADP+)	1.3.1.2	Human	15025949
dihydropyrimidine dehydrogenase (NADP+)	1.3.1.2	Human	15045945
dihydropyrimidine dehydrogenase (NADP+)	1.3.1.2	Human	15069534
dihydropyrimidine dehydrogenase (NADP+)	1.3.1.2	Human	15134221
dihydropyrimidine dehydrogenase (NADP+)	1.3.1.2	Human	15222105
dihydropyrimidine dehydrogenase (NADP+)	1.3.1.2	Human	1581906
dihydropyrimidine dehydrogenase (NADP+)	1.3.1.2	Human	15944938
dihydropyrimidine dehydrogenase (NADP+)	1.3.1.2	Human	16163233
dihydropyrimidine dehydrogenase (NADP+)	1.3.1.2	Human	1629785
dihydropyrimidine dehydrogenase (NADP+)	1.3.1.2	Human	16556484
dihydropyrimidine dehydrogenase (NADP+)	1.3.1.2	Human	16761622
dihydropyrimidine dehydrogenase (NADP+)	1.3.1.2	Human	16786143
dihydropyrimidine dehydrogenase (NADP+)	1.3.1.2	Human	16912518
dihydropyrimidine dehydrogenase (NADP+)	1.3.1.2	Human	17046731
dihydropyrimidine dehydrogenase (NADP+)	1.3.1.2	Human	2060083
dihydropyrimidine dehydrogenase (NADP+)	1.3.1.2	Human	3202908
dihydropyrimidine dehydrogenase (NADP+)	1.3.1.2	Human	7602356
dihydropyrimidine dehydrogenase (NADP+)	1.3.1.2	Human	7825962
dihydropyrimidine dehydrogenase (NADP+)	1.3.1.2	Human	8093030
dihydropyrimidine dehydrogenase (NADP+)	1.3.1.2	Human	8097697
dihydropyrimidine dehydrogenase (NADP+)	1.3.1.2	Human	8504424
dihydropyrimidine dehydrogenase (NADP+)	1.3.1.2	Human	8878781
dihydropyrimidine dehydrogenase (NADP+)	1.3.1.2	Human	8895681
dihydropyrimidine dehydrogenase (NADP+)	1.3.1.2	Human	8950202
dihydropyrimidine dehydrogenase (NADP+)	1.3.1.2	Human	9029059
dihydropyrimidine dehydrogenase (NADP+)	1.3.1.2	Human	9056186
dihydropyrimidine dehydrogenase (NADP+)	1.3.1.2	Human	9152608
dihydropyrimidine dehydrogenase (NADP+)	1.3.1.2	Human	9182832
dihydropyrimidine dehydrogenase (NADP+)	1.3.1.2	Human	9264323
dihydropyrimidine dehydrogenase (NADP+)	1.3.1.2	Human	9280881
dihydropyrimidine dehydrogenase (NADP+)	1.3.1.2	Human	9414600
dihydropyrimidine dehydrogenase (NADP+)	1.3.1.2	Human	9464498
dihydropyrimidine dehydrogenase (NADP+)	1.3.1.2	Human	9597696
dihydropyrimidine dehydrogenase (NADP+)	1.3.1.2	Human	9808711
dihydropyrimidine dehydrogenase (NADP+)	1.3.1.2	Human	9819714
dihydropyrimidine dehydrogenase (NADP+)	1.3.1.2	Human	9914783
dihydroorotate oxidase	1.3.3.1	Human	10348793
dihydroorotate oxidase	1.3.3.1	Human	10473079
dihydroorotate oxidase	1.3.3.1	Human	10848981

dihydroorotate oxidase	1.3.3.1	Human	10853015
dihydroorotate oxidase	1.3.3.1	Human	10878295
dihydroorotate oxidase	1.3.3.1	Human	10887632
dihydroorotate oxidase	1.3.3.1	Human	11053058
dihydroorotate oxidase	1.3.3.1	Human	11060767
dihydroorotate oxidase	1.3.3.1	Human	11098485
dihydroorotate oxidase	1.3.3.1	Human	11219978
dihydroorotate oxidase	1.3.3.1	Human	11267945
dihydroorotate oxidase	1.3.3.1	Human	11334264
dihydroorotate oxidase	1.3.3.1	Human	11376565
dihydroorotate oxidase	1.3.3.1	Human	11522581
dihydroorotate oxidase	1.3.3.1	Human	11555601
dihydroorotate oxidase	1.3.3.1	Human	11697836
dihydroorotate oxidase	1.3.3.1	Human	11796730
dihydroorotate oxidase	1.3.3.1	Human	11862480
dihydroorotate oxidase	1.3.3.1	Human	11865631
dihydroorotate oxidase	1.3.3.1	Human	11936689
dihydroorotate oxidase	1.3.3.1	Human	11962674
dihydroorotate oxidase	1.3.3.1	Human	12084458
dihydroorotate oxidase	1.3.3.1	Human	12187768
dihydroorotate oxidase	1.3.3.1	Human	12469154
dihydroorotate oxidase	1.3.3.1	Human	12527935
dihydroorotate oxidase	1.3.3.1	Human	12634225
dihydroorotate oxidase	1.3.3.1	Human	12820455
dihydroorotate oxidase	1.3.3.1	Human	12885330
dihydroorotate oxidase	1.3.3.1	Human	12967482
dihydroorotate oxidase	1.3.3.1	Human	14689231
dihydroorotate oxidase	1.3.3.1	Human	14744810
dihydroorotate oxidase	1.3.3.1	Human	15025949
dihydroorotate oxidase	1.3.3.1	Human	15045945
dihydroorotate oxidase	1.3.3.1	Human	15069534
dihydroorotate oxidase	1.3.3.1	Human	15134221
dihydroorotate oxidase	1.3.3.1	Human	15222105
dihydroorotate oxidase	1.3.3.1	Human	15450176
dihydroorotate oxidase	1.3.3.1	Human	15944938
dihydroorotate oxidase	1.3.3.1	Human	16163233
dihydroorotate oxidase	1.3.3.1	Human	1629785
dihydroorotate oxidase	1.3.3.1	Human	16556484
dihydroorotate oxidase	1.3.3.1	Human	17046731
dihydroorotate oxidase	1.3.3.1	Human	2060083
dihydroorotate oxidase	1.3.3.1	Human	3202908
dihydroorotate oxidase	1.3.3.1	Human	6761622
dihydroorotate oxidase	1.3.3.1	Human	7602356

dihydroorotate oxidase	1.3.3.1	Human	7825962
dihydroorotate oxidase	1.3.3.1	Human	8093030
dihydroorotate oxidase	1.3.3.1	Human	8097697
dihydroorotate oxidase	1.3.3.1	Human	8878781
dihydroorotate oxidase	1.3.3.1	Human	8895681
dihydroorotate oxidase	1.3.3.1	Human	8950202
dihydroorotate oxidase	1.3.3.1	Human	9056186
dihydroorotate oxidase	1.3.3.1	Human	9182832
dihydroorotate oxidase	1.3.3.1	Human	9280881
dihydroorotate oxidase	1.3.3.1	Human	9464498
dihydroorotate oxidase	1.3.3.1	Human	9597696
dihydroorotate oxidase	1.3.3.1	Human	9636062
dihydroorotate oxidase	1.3.3.1	Human	9819714
dihydroorotate oxidase	1.3.3.1	Human	9860876
dihydroorotate oxidase	1.3.3.1	Human	9914783
coproporphyrinogen oxidase	1.3.3.3	Human	10787385
coproporphyrinogen oxidase	1.3.3.3	Human	11368326
coproporphyrinogen oxidase	1.3.3.3	Human	16567402
acyl-CoA oxidase	1.3.3.6	Human	10318668
acyl-CoA oxidase	1.3.3.6	Human	11156684
acyl-CoA oxidase	1.3.3.6	Human	11330046
acyl-CoA oxidase	1.3.3.6	Human	12538078
acyl-CoA oxidase	1.3.3.6	Human	12758125
acyl-CoA oxidase	1.3.3.6	Human	14500732
acyl-CoA oxidase	1.3.3.6	Human	15805059
acyl-CoA oxidase	1.3.3.6	Human	1989516
acyl-CoA oxidase	1.3.3.6	Human	2049482
acyl-CoA oxidase	1.3.3.6	Human	2811611
acyl-CoA oxidase	1.3.3.6	Human	3367697
acyl-CoA oxidase	1.3.3.6	Human	3732222
acyl-CoA oxidase	1.3.3.6	Human	518563
acyl-CoA oxidase	1.3.3.6	Human	6240978
acyl-CoA oxidase	1.3.3.6	Human	6540549
acyl-CoA oxidase	1.3.3.6	Human	6541949
acyl-CoA oxidase	1.3.3.6	Human	7860752
acyl-CoA oxidase	1.3.3.6	Human	7867664
acyl-CoA oxidase	1.3.3.6	Human	8662598
acyl-CoA oxidase	1.3.3.6	Human	8784738
acyl-CoA oxidase	1.3.3.6	Human	8798738
acyl-CoA oxidase	1.3.3.6	Human	8895731
long-chain-acyl-CoA dehydrogenase	1.3.99.13	Human	12716879
long-chain-acyl-CoA dehydrogenase	1.3.99.13	Human	15535801
long-chain-acyl-CoA dehydrogenase	1.3.99.13	Human	15850553

long-chain-acyl-CoA dehydrogenase	1.3.99.13	Human	1637289
long-chain-acyl-CoA dehydrogenase	1.3.99.13	Human	1744086
long-chain-acyl-CoA dehydrogenase	1.3.99.13	Human	2914148
long-chain-acyl-CoA dehydrogenase	1.3.99.13	Human	3967008
long-chain-acyl-CoA dehydrogenase	1.3.99.13	Human	7551821
long-chain-acyl-CoA dehydrogenase	1.3.99.13	Human	8034667
long-chain-acyl-CoA dehydrogenase	1.3.99.13	Human	8798738
long-chain-acyl-CoA dehydrogenase	1.3.99.13	Human	8941110
long-chain-acyl-CoA dehydrogenase	1.3.99.13	Human	9177236
long-chain-acyl-CoA dehydrogenase	1.3.99.13	Human	9973285
butyryl-CoA dehydrogenase	1.3.99.2	Human	12892042
butyryl-CoA dehydrogenase	1.3.99.2	Human	15358373
butyryl-CoA dehydrogenase	1.3.99.2	Human	15850406
butyryl-CoA dehydrogenase	1.3.99.2	Human	16297616
butyryl-CoA dehydrogenase	1.3.99.2	Human	8941110
butyryl-CoA dehydrogenase	1.3.99.2	Human	9177236
butyryl-CoA dehydrogenase	1.3.99.2	Human	9271417
acyl-CoA dehydrogenase	1.3.99.3	Human	10832093
acyl-CoA dehydrogenase	1.3.99.3	Human	12359260
acyl-CoA dehydrogenase	1.3.99.3	Human	15358373
acyl-CoA dehydrogenase	1.3.99.3	Human	15850406
acyl-CoA dehydrogenase	1.3.99.3	Human	9177236
acyl-CoA dehydrogenase	1.3.99.3	Human	9484241
3-oxo-5alpha-steroid 4-dehydrogenase	1.3.99.5	Human	11408363
3-oxo-5alpha-steroid 4-dehydrogenase	1.3.99.5	Human	12746845
3-oxo-5alpha-steroid 4-dehydrogenase	1.3.99.5	Human	16174723
3-oxo-5alpha-steroid 4-dehydrogenase	1.3.99.5	Human	6523531
3-oxo-5alpha-steroid 4-dehydrogenase	1.3.99.5	Human	7588289
3-oxo-5alpha-steroid 4-dehydrogenase	1.3.99.5	Human	9078395
glutamate dehydrogenase [NAD(P)+]	1.4.1.3	Human	17507377
glutamate dehydrogenase [NAD(P)+]	1.4.1.3	Human	3139028
glutamate dehydrogenase [NAD(P)+]	1.4.1.3	Human	3449598
glutamate dehydrogenase [NAD(P)+]	1.4.1.3	Human	7470041
glutamate dehydrogenase [NAD(P)+]	1.4.1.3	Human	8122033
pyridoxal 5'-phosphate synthase	1.4.3.5	Human	6822512
pyridoxal 5'-phosphate synthase	1.4.3.5	Human	9601034
1-pyrroline-5-carboxylate dehydrogenase	1.5.1.12	Human	10363370
1-pyrroline-5-carboxylate dehydrogenase	1.5.1.12	Human	10398729
1-pyrroline-5-carboxylate dehydrogenase	1.5.1.12	Human	10441499
1-pyrroline-5-carboxylate dehydrogenase	1.5.1.12	Human	10759508
1-pyrroline-5-carboxylate dehydrogenase	1.5.1.12	Human	10945345
1-pyrroline-5-carboxylate dehydrogenase	1.5.1.12	Human	11726714
1-pyrroline-5-carboxylate dehydrogenase	1.5.1.12	Human	12602867

1-pyrroline-5-carboxylate dehydrogenase	1.5.1.12	Human	14602584
1-pyrroline-5-carboxylate dehydrogenase	1.5.1.12	Human	15077666
1-pyrroline-5-carboxylate dehydrogenase	1.5.1.12	Human	2860838
1-pyrroline-5-carboxylate dehydrogenase	1.5.1.12	Human	9351242
methylenetetrahydrofolate dehydrogenase (NAD+)	1.5.1.15	Human	718836
methylenetetrahydrofolate reductase [NAD(P)H]	1.5.1.20	Human	10462593
methylenetetrahydrofolate reductase [NAD(P)H]	1.5.1.20	Human	10948708
methylenetetrahydrofolate reductase [NAD(P)H]	1.5.1.20	Human	11302003
methylenetetrahydrofolate reductase [NAD(P)H]	1.5.1.20	Human	11343335
methylenetetrahydrofolate reductase [NAD(P)H]	1.5.1.20	Human	11398138
methylenetetrahydrofolate reductase [NAD(P)H]	1.5.1.20	Human	11680544
methylenetetrahydrofolate reductase [NAD(P)H]	1.5.1.20	Human	11712321
methylenetetrahydrofolate reductase [NAD(P)H]	1.5.1.20	Human	11927833
methylenetetrahydrofolate reductase [NAD(P)H]	1.5.1.20	Human	12028998
methylenetetrahydrofolate reductase [NAD(P)H]	1.5.1.20	Human	12038037
methylenetetrahydrofolate reductase [NAD(P)H]	1.5.1.20	Human	12600862
methylenetetrahydrofolate reductase [NAD(P)H]	1.5.1.20	Human	12897091
methylenetetrahydrofolate reductase [NAD(P)H]	1.5.1.20	Human	12914571
methylenetetrahydrofolate reductase [NAD(P)H]	1.5.1.20	Human	14608052
methylenetetrahydrofolate reductase [NAD(P)H]	1.5.1.20	Human	14728017
methylenetetrahydrofolate reductase [NAD(P)H]	1.5.1.20	Human	14973104
methylenetetrahydrofolate reductase [NAD(P)H]	1.5.1.20	Human	15033905
methylenetetrahydrofolate reductase [NAD(P)H]	1.5.1.20	Human	15207432

methylenetetrahydrofolate reductase [NAD(P)H]	1.5.1.20	Human	15449187
methylenetetrahydrofolate reductase [NAD(P)H]	1.5.1.20	Human	15546509
methylenetetrahydrofolate reductase [NAD(P)H]	1.5.1.20	Human	15581487
methylenetetrahydrofolate reductase [NAD(P)H]	1.5.1.20	Human	15598763
methylenetetrahydrofolate reductase [NAD(P)H]	1.5.1.20	Human	15688606
methylenetetrahydrofolate reductase [NAD(P)H]	1.5.1.20	Human	15773669
methylenetetrahydrofolate reductase [NAD(P)H]	1.5.1.20	Human	15894672
methylenetetrahydrofolate reductase [NAD(P)H]	1.5.1.20	Human	15935452
methylenetetrahydrofolate reductase [NAD(P)H]	1.5.1.20	Human	16055944
methylenetetrahydrofolate reductase [NAD(P)H]	1.5.1.20	Human	16108833
methylenetetrahydrofolate reductase [NAD(P)H]	1.5.1.20	Human	16128738
methylenetetrahydrofolate reductase [NAD(P)H]	1.5.1.20	Human	16274753
methylenetetrahydrofolate reductase [NAD(P)H]	1.5.1.20	Human	16538645
methylenetetrahydrofolate reductase [NAD(P)H]	1.5.1.20	Human	16706930
methylenetetrahydrofolate reductase [NAD(P)H]	1.5.1.20	Human	17105984
methylenetetrahydrofolate reductase [NAD(P)H]	1.5.1.20	Human	3143307
methylenetetrahydrofolate reductase [NAD(P)H]	1.5.1.20	Human	6352699
methylenetetrahydrofolate reductase [NAD(P)H]	1.5.1.20	Human	6391540
methylenetetrahydrofolate reductase [NAD(P)H]	1.5.1.20	Human	9789068
6,7-dihydropteridine reductase	1.5.1.34	Human	2913929
6,7-dihydropteridine reductase	1.5.1.34	Human	6797415
formyltetrahydrofolate dehydrogenase	1.5.1.6	Human	9359603
proline dehydrogenase	1.5.99.8	Human	11788754
proline dehydrogenase	1.5.99.8	Human	12602867

dihydrolipoyl dehydrogenase	1.8.1.4	Human	10885793
dihydrolipoyl dehydrogenase	1.8.1.4	Human	11641455
dihydrolipoyl dehydrogenase	1.8.1.4	Human	12812918
dihydrolipoyl dehydrogenase	1.8.1.4	Human	15173434
dihydrolipoyl dehydrogenase	1.8.1.4	Human	15915669
dihydrolipoyl dehydrogenase	1.8.1.4	Human	16581023
dihydrolipoyl dehydrogenase	1.8.1.4	Human	16616211
dihydrolipoyl dehydrogenase	1.8.1.4	Human	16875466
dihydrolipoyl dehydrogenase	1.8.1.4	Human	2381301
dihydrolipoyl dehydrogenase	1.8.1.4	Human	2404025
dihydrolipoyl dehydrogenase	1.8.1.4	Human	3103467
dihydrolipoyl dehydrogenase	1.8.1.4	Human	3425711
dihydrolipoyl dehydrogenase	1.8.1.4	Human	7782942
glutathione-disulfide reductase	1.8.1.7	Human	11133045
glutathione-disulfide reductase	1.8.1.7	Human	12204336
glutathione-disulfide reductase	1.8.1.7	Human	12453665
glutathione-disulfide reductase	1.8.1.7	Human	1605642
glutathione-disulfide reductase	1.8.1.7	Human	2848577
glutathione-disulfide reductase	1.8.1.7	Human	3698652
glutathione-disulfide reductase	1.8.1.7	Human	3963383
glutathione-disulfide reductase	1.8.1.7	Human	8843715
glutathione-disulfide reductase	1.8.1.7	Human	9350472
cytochrome-c oxidase	1.9.3.1	Human	10490029
cytochrome-c oxidase	1.9.3.1	Human	11988227
cytochrome-c oxidase	1.9.3.1	Human	12059041
cytochrome-c oxidase	1.9.3.1	Human	12145150
cytochrome-c oxidase	1.9.3.1	Human	12874793
cytochrome-c oxidase	1.9.3.1	Human	1315683
cytochrome-c oxidase	1.9.3.1	Human	1450614
cytochrome-c oxidase	1.9.3.1	Human	15504366
cytochrome-c oxidase	1.9.3.1	Human	15708625
cytochrome-c oxidase	1.9.3.1	Human	16704969
cytochrome-c oxidase	1.9.3.1	Human	167928
cytochrome-c oxidase	1.9.3.1	Human	174553
cytochrome-c oxidase	1.9.3.1	Human	194851
cytochrome-c oxidase	1.9.3.1	Human	206437
cytochrome-c oxidase	1.9.3.1	Human	2822680
cytochrome-c oxidase	1.9.3.1	Human	2849368
cytochrome-c oxidase	1.9.3.1	Human	2854388
cytochrome-c oxidase	1.9.3.1	Human	3000820
cytochrome-c oxidase	1.9.3.1	Human	3002436
cytochrome-c oxidase	1.9.3.1	Human	356174
cytochrome-c oxidase	1.9.3.1	Human	3569141

cytochrome-c oxidase	1.9.3.1	Human	3924042
cytochrome-c oxidase	1.9.3.1	Human	6091751
cytochrome-c oxidase	1.9.3.1	Human	6254961
cytochrome-c oxidase	1.9.3.1	Human	6268138
cytochrome-c oxidase	1.9.3.1	Human	6286610
cytochrome-c oxidase	1.9.3.1	Human	6320180
cytochrome-c oxidase	1.9.3.1	Human	7356983
cytochrome-c oxidase	1.9.3.1	Human	8013452
cytochrome-c oxidase	1.9.3.1	Human	8386021
phosphatidylethanolamine N-methyltransferase	2.1.1.17	Human	8207329
phosphatidylethanolamine N-methyltransferase	2.1.1.17	Human	9380436
thymidylate synthase	2.1.1.45	Human	11358693
thymidylate synthase	2.1.1.45	Human	12412165
thymidylate synthase	2.1.1.45	Human	12457437
thymidylate synthase	2.1.1.45	Human	12470718
thymidylate synthase	2.1.1.45	Human	12544347
thymidylate synthase	2.1.1.45	Human	14578129
thymidylate synthase	2.1.1.45	Human	14648018
thymidylate synthase	2.1.1.45	Human	14689231
thymidylate synthase	2.1.1.45	Human	15025949
thymidylate synthase	2.1.1.45	Human	15134221
thymidylate synthase	2.1.1.45	Human	15598787
thymidylate synthase	2.1.1.45	Human	16077970
thymidylate synthase	2.1.1.45	Human	16540728
thymidylate synthase	2.1.1.45	Human	16617381
thymidylate synthase	2.1.1.45	Human	3709927
thymidylate synthase	2.1.1.45	Human	7602356
thymidylate synthase	2.1.1.45	Human	8805515
phosphoribosylaminoimidazolecarboxamide formyltransferase	2.1.2.3	Human	6335666
aspartate carbamoyltransferase	2.1.3.2	Human	10336386
aspartate carbamoyltransferase	2.1.3.2	Human	10593256
aspartate carbamoyltransferase	2.1.3.2	Human	11397099
aspartate carbamoyltransferase	2.1.3.2	Human	15165857
aspartate carbamoyltransferase	2.1.3.2	Human	15529744
aspartate carbamoyltransferase	2.1.3.2	Human	16120448
aspartate carbamoyltransferase	2.1.3.2	Human	17008138
aspartate carbamoyltransferase	2.1.3.2	Human	3047117
aspartate carbamoyltransferase	2.1.3.2	Human	3542019
aspartate carbamoyltransferase	2.1.3.2	Human	3907993
aspartate carbamoyltransferase	2.1.3.2	Human	4092695

aspartate carbamoyltransferase	2.1.3.2	Human	6115855
aspartate carbamoyltransferase	2.1.3.2	Human	6298785
aspartate carbamoyltransferase	2.1.3.2	Human	7051000
aspartate carbamoyltransferase	2.1.3.2	Human	9611817
aspartate carbamoyltransferase	2.1.3.2	Human	9626698
aspartate carbamoyltransferase	2.1.3.2	Human	9659392
ornithine carbamoyltransferase	2.1.3.3	Human	1505922
ornithine carbamoyltransferase	2.1.3.3	Human	205703
ornithine carbamoyltransferase	2.1.3.3	Human	2667139
ornithine carbamoyltransferase	2.1.3.3	Human	2667140
ornithine carbamoyltransferase	2.1.3.3	Human	2667141
ornithine carbamoyltransferase	2.1.3.3	Human	8019156
ornithine carbamoyltransferase	2.1.3.3	Human	8168544
ornithine carbamoyltransferase	2.1.3.3	Human	9501170
ornithine carbamoyltransferase	2.1.3.3	Human	9540805
glycine amidinotransferase	2.1.4.1	Human	7419715
transketolase	2.2.1.1	Human	10975072
transketolase	2.2.1.1	Human	11072071
transketolase	2.2.1.1	Human	16354724
transketolase	2.2.1.1	Human	1939098
transketolase	2.2.1.1	Human	9924800
transaldolase	2.2.1.2	Human	11390181
transaldolase	2.2.1.2	Human	12359249
transaldolase	2.2.1.2	Human	15263091
transaldolase	2.2.1.2	Human	16092052
transaldolase	2.2.1.2	Human	17046540
transaldolase	2.2.1.2	Human	9565623
transaldolase	2.2.1.2	Human	9973403
dihydrolipoyllysine-residue acetyltransferase	2.3.1.12	Human	3840997
glycerol-3-phosphate O-acyltransferase	2.3.1.15	Human	11003606
glycerol-3-phosphate O-acyltransferase	2.3.1.15	Human	11284717
glycerol-3-phosphate O-acyltransferase	2.3.1.15	Human	12464581
glycerol-3-phosphate O-acyltransferase	2.3.1.15	Human	12730219
glycerol-3-phosphate O-acyltransferase	2.3.1.15	Human	14724270
glycerol-3-phosphate O-acyltransferase	2.3.1.15	Human	15102885
glycerol-3-phosphate O-acyltransferase	2.3.1.15	Human	16234267
glycerol-3-phosphate O-acyltransferase	2.3.1.15	Human	16431156
glycerol-3-phosphate O-acyltransferase	2.3.1.15	Human	16507761
glycerol-3-phosphate O-acyltransferase	2.3.1.15	Human	2303421
glycerol-3-phosphate O-acyltransferase	2.3.1.15	Human	2559137
glycerol-3-phosphate O-acyltransferase	2.3.1.15	Human	2674663
glycerol-3-phosphate O-acyltransferase	2.3.1.15	Human	2737209
glycerol-3-phosphate O-acyltransferase	2.3.1.15	Human	2830105

glycerol-3-phosphate O-acyltransferase	2.3.1.15	Human	3103689
glycerol-3-phosphate O-acyltransferase	2.3.1.15	Human	3311005
glycerol-3-phosphate O-acyltransferase	2.3.1.15	Human	3334861
glycerol-3-phosphate O-acyltransferase	2.3.1.15	Human	6497849
glycerol-3-phosphate O-acyltransferase	2.3.1.15	Human	813635
glycerol-3-phosphate O-acyltransferase	2.3.1.15	Human	8387510
glycerol-3-phosphate O-acyltransferase	2.3.1.15	Human	9393688
acetyl-CoA C-acyltransferase	2.3.1.16	Human	3967008
acetyl-CoA C-acyltransferase	2.3.1.16	Human	7068598
acetyl-CoA C-acyltransferase	2.3.1.16	Human	7374368
carnitine O-palmitoyltransferase	2.3.1.21	Human	10417344
carnitine O-palmitoyltransferase	2.3.1.21	Human	10709666
carnitine O-palmitoyltransferase	2.3.1.21	Human	10956641
carnitine O-palmitoyltransferase	2.3.1.21	Human	11356163
carnitine O-palmitoyltransferase	2.3.1.21	Human	11371554
carnitine O-palmitoyltransferase	2.3.1.21	Human	11463952
carnitine O-palmitoyltransferase	2.3.1.21	Human	12015320
carnitine O-palmitoyltransferase	2.3.1.21	Human	12359092
carnitine O-palmitoyltransferase	2.3.1.21	Human	12574149
carnitine O-palmitoyltransferase	2.3.1.21	Human	12619873
carnitine O-palmitoyltransferase	2.3.1.21	Human	12761301
carnitine O-palmitoyltransferase	2.3.1.21	Human	14751860
carnitine O-palmitoyltransferase	2.3.1.21	Human	15044358
carnitine O-palmitoyltransferase	2.3.1.21	Human	15247243
carnitine O-palmitoyltransferase	2.3.1.21	Human	15539300
carnitine O-palmitoyltransferase	2.3.1.21	Human	15590999
carnitine O-palmitoyltransferase	2.3.1.21	Human	15623825
carnitine O-palmitoyltransferase	2.3.1.21	Human	1563551
carnitine O-palmitoyltransferase	2.3.1.21	Human	15878185
carnitine O-palmitoyltransferase	2.3.1.21	Human	15919095
carnitine O-palmitoyltransferase	2.3.1.21	Human	16177188
carnitine O-palmitoyltransferase	2.3.1.21	Human	16225603
carnitine O-palmitoyltransferase	2.3.1.21	Human	16509570
carnitine O-palmitoyltransferase	2.3.1.21	Human	16528409
carnitine O-palmitoyltransferase	2.3.1.21	Human	16545538
carnitine O-palmitoyltransferase	2.3.1.21	Human	16584169
carnitine O-palmitoyltransferase	2.3.1.21	Human	16763001
carnitine O-palmitoyltransferase	2.3.1.21	Human	17021367
carnitine O-palmitoyltransferase	2.3.1.21	Human	17089095
carnitine O-palmitoyltransferase	2.3.1.21	Human	1979695
carnitine O-palmitoyltransferase	2.3.1.21	Human	2166437
carnitine O-palmitoyltransferase	2.3.1.21	Human	2914148
carnitine O-palmitoyltransferase	2.3.1.21	Human	3090894

carnitine O-palmitoyltransferase	2.3.1.21	Human	3709811
carnitine O-palmitoyltransferase	2.3.1.21	Human	3800962
carnitine O-palmitoyltransferase	2.3.1.21	Human	3834060
carnitine O-palmitoyltransferase	2.3.1.21	Human	6105152
carnitine O-palmitoyltransferase	2.3.1.21	Human	670195
carnitine O-palmitoyltransferase	2.3.1.21	Human	7334004
carnitine O-palmitoyltransferase	2.3.1.21	Human	7734439
carnitine O-palmitoyltransferase	2.3.1.21	Human	8141397
carnitine O-palmitoyltransferase	2.3.1.21	Human	8335590
carnitine O-palmitoyltransferase	2.3.1.21	Human	8479178
carnitine O-palmitoyltransferase	2.3.1.21	Human	8589632
carnitine O-palmitoyltransferase	2.3.1.21	Human	8857518
carnitine O-palmitoyltransferase	2.3.1.21	Human	9136891
carnitine O-palmitoyltransferase	2.3.1.21	Human	9447322
carnitine O-palmitoyltransferase	2.3.1.21	Human	9545636
carnitine O-palmitoyltransferase	2.3.1.21	Human	9792707
carnitine O-palmitoyltransferase	2.3.1.21	Human	9989283
sterol O-acyltransferase	2.3.1.26	Human	10593897
sterol O-acyltransferase	2.3.1.26	Human	10656290
sterol O-acyltransferase	2.3.1.26	Human	11888294
sterol O-acyltransferase	2.3.1.26	Human	12787409
sterol O-acyltransferase	2.3.1.26	Human	1450216
sterol O-acyltransferase	2.3.1.26	Human	2885178
sterol O-acyltransferase	2.3.1.26	Human	3593752
sterol O-acyltransferase	2.3.1.26	Human	3719008
sterol O-acyltransferase	2.3.1.26	Human	3766727
sterol O-acyltransferase	2.3.1.26	Human	3812206
sterol O-acyltransferase	2.3.1.26	Human	3926761
sterol O-acyltransferase	2.3.1.26	Human	4056052
sterol O-acyltransferase	2.3.1.26	Human	6347025
sterol O-acyltransferase	2.3.1.26	Human	8258956
sterol O-acyltransferase	2.3.1.26	Human	8466946
sterol O-acyltransferase	2.3.1.26	Human	8820097
sterol O-acyltransferase	2.3.1.26	Human	9857049
5-aminolevulinate synthase	2.3.1.37	Human	10634305
5-aminolevulinate synthase	2.3.1.37	Human	10727444
5-aminolevulinate synthase	2.3.1.37	Human	10729988
5-aminolevulinate synthase	2.3.1.37	Human	10787385
5-aminolevulinate synthase	2.3.1.37	Human	10825473
5-aminolevulinate synthase	2.3.1.37	Human	11202048
5-aminolevulinate synthase	2.3.1.37	Human	11202050
5-aminolevulinate synthase	2.3.1.37	Human	11368326
5-aminolevulinate synthase	2.3.1.37	Human	12121995

5-aminolevulinate synthase	2.3.1.37	Human	12393745
5-aminolevulinate synthase	2.3.1.37	Human	12469218
5-aminolevulinate synthase	2.3.1.37	Human	12627002
5-aminolevulinate synthase	2.3.1.37	Human	126586
5-aminolevulinate synthase	2.3.1.37	Human	12881517
5-aminolevulinate synthase	2.3.1.37	Human	14643887
5-aminolevulinate synthase	2.3.1.37	Human	1511083
5-aminolevulinate synthase	2.3.1.37	Human	15178759
5-aminolevulinate synthase	2.3.1.37	Human	15259603
5-aminolevulinate synthase	2.3.1.37	Human	1526942
5-aminolevulinate synthase	2.3.1.37	Human	15547665
5-aminolevulinate synthase	2.3.1.37	Human	15797241
5-aminolevulinate synthase	2.3.1.37	Human	15972158
5-aminolevulinate synthase	2.3.1.37	Human	16122419
5-aminolevulinate synthase	2.3.1.37	Human	16181105
5-aminolevulinate synthase	2.3.1.37	Human	16567402
5-aminolevulinate synthase	2.3.1.37	Human	16846079
5-aminolevulinate synthase	2.3.1.37	Human	1688552
5-aminolevulinate synthase	2.3.1.37	Human	16892088
5-aminolevulinate synthase	2.3.1.37	Human	16904069
5-aminolevulinate synthase	2.3.1.37	Human	1954232
5-aminolevulinate synthase	2.3.1.37	Human	1959865
5-aminolevulinate synthase	2.3.1.37	Human	2050126
5-aminolevulinate synthase	2.3.1.37	Human	2241158
5-aminolevulinate synthase	2.3.1.37	Human	2317819
5-aminolevulinate synthase	2.3.1.37	Human	266710
5-aminolevulinate synthase	2.3.1.37	Human	3009001
5-aminolevulinate synthase	2.3.1.37	Human	3094379
5-aminolevulinate synthase	2.3.1.37	Human	3359971
5-aminolevulinate synthase	2.3.1.37	Human	3755290
5-aminolevulinate synthase	2.3.1.37	Human	3840094
5-aminolevulinate synthase	2.3.1.37	Human	3966797
5-aminolevulinate synthase	2.3.1.37	Human	6092369
5-aminolevulinate synthase	2.3.1.37	Human	6213232
5-aminolevulinate synthase	2.3.1.37	Human	6547609
5-aminolevulinate synthase	2.3.1.37	Human	6824732
5-aminolevulinate synthase	2.3.1.37	Human	6873612
5-aminolevulinate synthase	2.3.1.37	Human	6954546
5-aminolevulinate synthase	2.3.1.37	Human	7093306
5-aminolevulinate synthase	2.3.1.37	Human	7592563
5-aminolevulinate synthase	2.3.1.37	Human	8076930
5-aminolevulinate synthase	2.3.1.37	Human	818637
5-aminolevulinate synthase	2.3.1.37	Human	8385933

5-aminolevulinate synthase	2.3.1.37	Human	8413301
5-aminolevulinate synthase	2.3.1.37	Human	9169013
5-aminolevulinate synthase	2.3.1.37	Human	9173682
5-aminolevulinate synthase	2.3.1.37	Human	9806796
5-aminolevulinate synthase	2.3.1.37	Human	9879810
arylamine N-acetyltransferase	2.3.1.5	Human	10100739
arylamine N-acetyltransferase	2.3.1.5	Human	10467435
arylamine N-acetyltransferase	2.3.1.5	Human	10471401
arylamine N-acetyltransferase	2.3.1.5	Human	10721063
arylamine N-acetyltransferase	2.3.1.5	Human	10843785
arylamine N-acetyltransferase	2.3.1.5	Human	10931207
arylamine N-acetyltransferase	2.3.1.5	Human	11122368
arylamine N-acetyltransferase	2.3.1.5	Human	12465141
arylamine N-acetyltransferase	2.3.1.5	Human	12734775
arylamine N-acetyltransferase	2.3.1.5	Human	12736370
arylamine N-acetyltransferase	2.3.1.5	Human	12736803
arylamine N-acetyltransferase	2.3.1.5	Human	14550904
arylamine N-acetyltransferase	2.3.1.5	Human	14578935
arylamine N-acetyltransferase	2.3.1.5	Human	15219412
arylamine N-acetyltransferase	2.3.1.5	Human	15228600
arylamine N-acetyltransferase	2.3.1.5	Human	15782814
arylamine N-acetyltransferase	2.3.1.5	Human	15978059
arylamine N-acetyltransferase	2.3.1.5	Human	15978063
arylamine N-acetyltransferase	2.3.1.5	Human	16095816
arylamine N-acetyltransferase	2.3.1.5	Human	1715400
arylamine N-acetyltransferase	2.3.1.5	Human	1806289
arylamine N-acetyltransferase	2.3.1.5	Human	1829510
arylamine N-acetyltransferase	2.3.1.5	Human	1974286
arylamine N-acetyltransferase	2.3.1.5	Human	1976757
arylamine N-acetyltransferase	2.3.1.5	Human	1977416
arylamine N-acetyltransferase	2.3.1.5	Human	2054611
arylamine N-acetyltransferase	2.3.1.5	Human	2133350
arylamine N-acetyltransferase	2.3.1.5	Human	2167223
arylamine N-acetyltransferase	2.3.1.5	Human	3179707
arylamine N-acetyltransferase	2.3.1.5	Human	3574290
arylamine N-acetyltransferase	2.3.1.5	Human	3702592
arylamine N-acetyltransferase	2.3.1.5	Human	7552254
arylamine N-acetyltransferase	2.3.1.5	Human	7686576
arylamine N-acetyltransferase	2.3.1.5	Human	7741268
arylamine N-acetyltransferase	2.3.1.5	Human	8098713
arylamine N-acetyltransferase	2.3.1.5	Human	8106958
arylamine N-acetyltransferase	2.3.1.5	Human	8176952
arylamine N-acetyltransferase	2.3.1.5	Human	8420625

arylamine N-acetyltransferase	2.3.1.5	Human	8524412
arylamine N-acetyltransferase	2.3.1.5	Human	8550820
arylamine N-acetyltransferase	2.3.1.5	Human	8599175
arylamine N-acetyltransferase	2.3.1.5	Human	8888105
arylamine N-acetyltransferase	2.3.1.5	Human	8930697
arylamine N-acetyltransferase	2.3.1.5	Human	8943074
arylamine N-acetyltransferase	2.3.1.5	Human	9181516
arylamine N-acetyltransferase	2.3.1.5	Human	9238850
arylamine N-acetyltransferase	2.3.1.5	Human	9437760
arylamine N-acetyltransferase	2.3.1.5	Human	9566052
arylamine N-acetyltransferase	2.3.1.5	Human	9597751
arylamine N-acetyltransferase	2.3.1.5	Human	9669546
arylamine N-acetyltransferase	2.3.1.5	Human	9811631
arylamine N-acetyltransferase	2.3.1.5	Human	9949305
serine C-palmitoyltransferase	2.3.1.50	Human	10722759
serine C-palmitoyltransferase	2.3.1.50	Human	10736421
serine C-palmitoyltransferase	2.3.1.50	Human	10764732
serine C-palmitoyltransferase	2.3.1.50	Human	10818445
serine C-palmitoyltransferase	2.3.1.50	Human	10862608
serine C-palmitoyltransferase	2.3.1.50	Human	10971324
serine C-palmitoyltransferase	2.3.1.50	Human	11279212
serine C-palmitoyltransferase	2.3.1.50	Human	11903061
serine C-palmitoyltransferase	2.3.1.50	Human	12445191
serine C-palmitoyltransferase	2.3.1.50	Human	12531548
serine C-palmitoyltransferase	2.3.1.50	Human	12570999
serine C-palmitoyltransferase	2.3.1.50	Human	12612207
serine C-palmitoyltransferase	2.3.1.50	Human	12686119
serine C-palmitoyltransferase	2.3.1.50	Human	12704216
serine C-palmitoyltransferase	2.3.1.50	Human	12736045
serine C-palmitoyltransferase	2.3.1.50	Human	12782147
serine C-palmitoyltransferase	2.3.1.50	Human	14563682
serine C-palmitoyltransferase	2.3.1.50	Human	14744154
serine C-palmitoyltransferase	2.3.1.50	Human	15066023
serine C-palmitoyltransferase	2.3.1.50	Human	15180163
serine C-palmitoyltransferase	2.3.1.50	Human	15297021
serine C-palmitoyltransferase	2.3.1.50	Human	15545514
serine C-palmitoyltransferase	2.3.1.50	Human	15560753
serine C-palmitoyltransferase	2.3.1.50	Human	15562249
serine C-palmitoyltransferase	2.3.1.50	Human	16117797
serine C-palmitoyltransferase	2.3.1.50	Human	16157870
serine C-palmitoyltransferase	2.3.1.50	Human	16210380
serine C-palmitoyltransferase	2.3.1.50	Human	16216550
serine C-palmitoyltransferase	2.3.1.50	Human	17090526

serine C-palmitoyltransferase	2.3.1.50	Human	9186561
serine C-palmitoyltransferase	2.3.1.50	Human	9363775
serine C-palmitoyltransferase	2.3.1.50	Human	9405408
serine C-palmitoyltransferase	2.3.1.50	Human	9714132
serine C-palmitoyltransferase	2.3.1.50	Human	9788249
diamine N-acetyltransferase	2.3.1.57	Human	10101027
diamine N-acetyltransferase	2.3.1.57	Human	10430062
diamine N-acetyltransferase	2.3.1.57	Human	11166157
diamine N-acetyltransferase	2.3.1.57	Human	11298808
diamine N-acetyltransferase	2.3.1.57	Human	11779193
diamine N-acetyltransferase	2.3.1.57	Human	12798351
diamine N-acetyltransferase	2.3.1.57	Human	12827295
diamine N-acetyltransferase	2.3.1.57	Human	1360468
diamine N-acetyltransferase	2.3.1.57	Human	1420355
diamine N-acetyltransferase	2.3.1.57	Human	15223770
diamine N-acetyltransferase	2.3.1.57	Human	15737201
diamine N-acetyltransferase	2.3.1.57	Human	1581359
diamine N-acetyltransferase	2.3.1.57	Human	1590311
diamine N-acetyltransferase	2.3.1.57	Human	16207710
diamine N-acetyltransferase	2.3.1.57	Human	16262603
diamine N-acetyltransferase	2.3.1.57	Human	16400014
diamine N-acetyltransferase	2.3.1.57	Human	16455797
diamine N-acetyltransferase	2.3.1.57	Human	2463788
diamine N-acetyltransferase	2.3.1.57	Human	2987201
diamine N-acetyltransferase	2.3.1.57	Human	3435554
diamine N-acetyltransferase	2.3.1.57	Human	3443136
diamine N-acetyltransferase	2.3.1.57	Human	3928404
diamine N-acetyltransferase	2.3.1.57	Human	4093447
diamine N-acetyltransferase	2.3.1.57	Human	6510524
diamine N-acetyltransferase	2.3.1.57	Human	6654645
diamine N-acetyltransferase	2.3.1.57	Human	7141002
diamine N-acetyltransferase	2.3.1.57	Human	7803498
diamine N-acetyltransferase	2.3.1.57	Human	8119700
diamine N-acetyltransferase	2.3.1.57	Human	8427870
diamine N-acetyltransferase	2.3.1.57	Human	8500690
diamine N-acetyltransferase	2.3.1.57	Human	8549747
diamine N-acetyltransferase	2.3.1.57	Human	8573111
diamine N-acetyltransferase	2.3.1.57	Human	8737675
diamine N-acetyltransferase	2.3.1.57	Human	8814137
diamine N-acetyltransferase	2.3.1.57	Human	8876622
diamine N-acetyltransferase	2.3.1.57	Human	8954982
diamine N-acetyltransferase	2.3.1.57	Human	9063811
diamine N-acetyltransferase	2.3.1.57	Human	9115288

diamine N-acetyltransferase	2.3.1.57	Human	9585063
diamine N-acetyltransferase	2.3.1.57	Human	9620361
diamine N-acetyltransferase	2.3.1.57	Human	9780334
choline O-acetyltransferase	2.3.1.6	Human	16480703
choline O-acetyltransferase	2.3.1.6	Human	2023918
choline O-acetyltransferase	2.3.1.6	Human	2572615
choline O-acetyltransferase	2.3.1.6	Human	566752
choline O-acetyltransferase	2.3.1.6	Human	7682855
fatty-acid synthase	2.3.1.85	Human	10385596
fatty-acid synthase	2.3.1.85	Human	11032949
fatty-acid synthase	2.3.1.85	Human	12131249
fatty-acid synthase	2.3.1.85	Human	12820377
fatty-acid synthase	2.3.1.85	Human	14767544
fatty-acid synthase	2.3.1.85	Human	15302084
fatty-acid synthase	2.3.1.85	Human	15491158
fatty-acid synthase	2.3.1.85	Human	15577743
fatty-acid synthase	2.3.1.85	Human	16007182
fatty-acid synthase	2.3.1.85	Human	16054091
fatty-acid synthase	2.3.1.85	Human	16374067
fatty-acid synthase	2.3.1.85	Human	16582625
fatty-acid synthase	2.3.1.85	Human	16729974
fatty-acid synthase	2.3.1.85	Human	7948007
fatty-acid synthase	2.3.1.85	Human	9510066
fatty-acid synthase	2.3.1.85	Human	9593836
aralkylamine N-acetyltransferase	2.3.1.87	Human	10329462
aralkylamine N-acetyltransferase	2.3.1.87	Human	10451021
aralkylamine N-acetyltransferase	2.3.1.87	Human	10537047
aralkylamine N-acetyltransferase	2.3.1.87	Human	10537048
aralkylamine N-acetyltransferase	2.3.1.87	Human	10537049
aralkylamine N-acetyltransferase	2.3.1.87	Human	11325593
aralkylamine N-acetyltransferase	2.3.1.87	Human	11432976
aralkylamine N-acetyltransferase	2.3.1.87	Human	11506377
aralkylamine N-acetyltransferase	2.3.1.87	Human	12052171
aralkylamine N-acetyltransferase	2.3.1.87	Human	12059970
aralkylamine N-acetyltransferase	2.3.1.87	Human	14759496
aralkylamine N-acetyltransferase	2.3.1.87	Human	15026119
aralkylamine N-acetyltransferase	2.3.1.87	Human	15081830
aralkylamine N-acetyltransferase	2.3.1.87	Human	15519681
aralkylamine N-acetyltransferase	2.3.1.87	Human	15773915
aralkylamine N-acetyltransferase	2.3.1.87	Human	15950762
aralkylamine N-acetyltransferase	2.3.1.87	Human	16024134
aralkylamine N-acetyltransferase	2.3.1.87	Human	16207298
aralkylamine N-acetyltransferase	2.3.1.87	Human	16556767

aralkylamine N-acetyltransferase	2.3.1.87	Human	16604054
aralkylamine N-acetyltransferase	2.3.1.87	Human	16687310
aralkylamine N-acetyltransferase	2.3.1.87	Human	16842539
aralkylamine N-acetyltransferase	2.3.1.87	Human	16842546
aralkylamine N-acetyltransferase	2.3.1.87	Human	16869299
aralkylamine N-acetyltransferase	2.3.1.87	Human	16962714
aralkylamine N-acetyltransferase	2.3.1.87	Human	1705890
aralkylamine N-acetyltransferase	2.3.1.87	Human	503196
aralkylamine N-acetyltransferase	2.3.1.87	Human	7498465
aralkylamine N-acetyltransferase	2.3.1.87	Human	7566441
aralkylamine N-acetyltransferase	2.3.1.87	Human	8674865
aralkylamine N-acetyltransferase	2.3.1.87	Human	9605498
aralkylamine N-acetyltransferase	2.3.1.87	Human	9703021
aralkylamine N-acetyltransferase	2.3.1.87	Human	9708862
acetyl-CoA C-acetyltransferase	2.3.1.9	Human	14693556
acetyl-CoA C-acetyltransferase	2.3.1.9	Human	15135409
acetyl-CoA C-acetyltransferase	2.3.1.9	Human	15466479
acetyl-CoA C-acetyltransferase	2.3.1.9	Human	2869784
gamma-glutamyltransferase	2.3.2.2	Human	11810401
gamma-glutamyltransferase	2.3.2.2	Human	12030366
gamma-glutamyltransferase	2.3.2.2	Human	12468440
gamma-glutamyltransferase	2.3.2.2	Human	12780970
gamma-glutamyltransferase	2.3.2.2	Human	15006645
gamma-glutamyltransferase	2.3.2.2	Human	16302185
gamma-glutamyltransferase	2.3.2.2	Human	2881890
gamma-glutamyltransferase	2.3.2.2	Human	2903803
gamma-glutamyltransferase	2.3.2.2	Human	7485380
gamma-glutamyltransferase	2.3.2.2	Human	8067452
gamma-glutamyltransferase	2.3.2.2	Human	8564390
gamma-glutamyltransferase	2.3.2.2	Human	8972486
gamma-glutamyltransferase	2.3.2.2	Human	9974125
citrate (Si)-synthase	2.3.3.1	Human	1004246
citrate (Si)-synthase	2.3.3.1	Human	11842094
citrate (Si)-synthase	2.3.3.1	Human	11872452
citrate (Si)-synthase	2.3.3.1	Human	15994367
citrate (Si)-synthase	2.3.3.1	Human	16269721
citrate (Si)-synthase	2.3.3.1	Human	3776117
citrate (Si)-synthase	2.3.3.1	Human	3916224
citrate (Si)-synthase	2.3.3.1	Human	6799496
citrate (Si)-synthase	2.3.3.1	Human	8526514
citrate (Si)-synthase	2.3.3.1	Human	9353808
citrate (Si)-synthase	2.3.3.1	Human	9554114
hydroxymethylglutaryl-CoA synthase	2.3.3.10	Human	11160362

hydroxymethylglutaryl-CoA synthase	2.3.3.10	Human	11485325
hydroxymethylglutaryl-CoA synthase	2.3.3.10	Human	16101500
hydroxymethylglutaryl-CoA synthase	2.3.3.10	Human	16864776
hydroxymethylglutaryl-CoA synthase	2.3.3.10	Human	16962226
hydroxymethylglutaryl-CoA synthase	2.3.3.10	Human	475
hydroxymethylglutaryl-CoA synthase	2.3.3.10	Human	7907092
ATP citrate synthase	2.3.3.8	Human	10759520
ATP citrate synthase	2.3.3.8	Human	10801800
ATP citrate synthase	2.3.3.8	Human	12135479
ATP citrate synthase	2.3.3.8	Human	12957888
ATP citrate synthase	2.3.3.8	Human	131232
ATP citrate synthase	2.3.3.8	Human	16226706
ATP citrate synthase	2.3.3.8	Human	16269773
ATP citrate synthase	2.3.3.8	Human	16461683
ATP citrate synthase	2.3.3.8	Human	16988757
ATP citrate synthase	2.3.3.8	Human	17404227
ATP citrate synthase	2.3.3.8	Human	2295639
ATP citrate synthase	2.3.3.8	Human	8832570
ATP citrate synthase	2.3.3.8	Human	9082912
phosphorylase	2.4.1.1	Human	10548038
phosphorylase	2.4.1.1	Human	11391834
phosphorylase	2.4.1.1	Human	11391835
phosphorylase	2.4.1.1	Human	11391836
phosphorylase	2.4.1.1	Human	11391837
phosphorylase	2.4.1.1	Human	11391838
phosphorylase	2.4.1.1	Human	11391839
phosphorylase	2.4.1.1	Human	11391840
phosphorylase	2.4.1.1	Human	11391841
phosphorylase	2.4.1.1	Human	11391842
phosphorylase	2.4.1.1	Human	12769745
phosphorylase	2.4.1.1	Human	15299833
phosphorylase	2.4.1.1	Human	15721288
phosphorylase	2.4.1.1	Human	1691273
phosphorylase	2.4.1.1	Human	7664039
glycogen(starch) synthase	2.4.1.11	Human	10067873
glycogen(starch) synthase	2.4.1.11	Human	10222257
glycogen(starch) synthase	2.4.1.11	Human	10684630
glycogen(starch) synthase	2.4.1.11	Human	11181947
glycogen(starch) synthase	2.4.1.11	Human	11467410
glycogen(starch) synthase	2.4.1.11	Human	11534633
glycogen(starch) synthase	2.4.1.11	Human	11834204
glycogen(starch) synthase	2.4.1.11	Human	11900279
glycogen(starch) synthase	2.4.1.11	Human	12617691

glycogen(starch) synthase	2.4.1.11	Human	14570701
glycogen(starch) synthase	2.4.1.11	Human	15840572
glycogen(starch) synthase	2.4.1.11	Human	15932409
glycogen(starch) synthase	2.4.1.11	Human	16101290
glycogen(starch) synthase	2.4.1.11	Human	1756915
glycogen(starch) synthase	2.4.1.11	Human	17569761
glycogen(starch) synthase	2.4.1.11	Human	17698598
glycogen(starch) synthase	2.4.1.11	Human	1959479
glycogen(starch) synthase	2.4.1.11	Human	208368
glycogen(starch) synthase	2.4.1.11	Human	2115296
glycogen(starch) synthase	2.4.1.11	Human	2154910
glycogen(starch) synthase	2.4.1.11	Human	219866
glycogen(starch) synthase	2.4.1.11	Human	227915
glycogen(starch) synthase	2.4.1.11	Human	2405698
glycogen(starch) synthase	2.4.1.11	Human	2822414
glycogen(starch) synthase	2.4.1.11	Human	3032541
glycogen(starch) synthase	2.4.1.11	Human	3092743
glycogen(starch) synthase	2.4.1.11	Human	3143265
glycogen(starch) synthase	2.4.1.11	Human	3930321
glycogen(starch) synthase	2.4.1.11	Human	6409592
glycogen(starch) synthase	2.4.1.11	Human	6412593
glycogen(starch) synthase	2.4.1.11	Human	7010073
glycogen(starch) synthase	2.4.1.11	Human	7672505
glycogen(starch) synthase	2.4.1.11	Human	7983805
glycogen(starch) synthase	2.4.1.11	Human	8226927
glycogen(starch) synthase	2.4.1.11	Human	8416266
glycogen(starch) synthase	2.4.1.11	Human	8514767
glycogen(starch) synthase	2.4.1.11	Human	8514849
glycogen(starch) synthase	2.4.1.11	Human	8541012
glycogen(starch) synthase	2.4.1.11	Human	8569754
glycogen(starch) synthase	2.4.1.11	Human	8591890
glycogen(starch) synthase	2.4.1.11	Human	8593937
glycogen(starch) synthase	2.4.1.11	Human	8612539
glycogen(starch) synthase	2.4.1.11	Human	8645005
glycogen(starch) synthase	2.4.1.11	Human	8721777
glycogen(starch) synthase	2.4.1.11	Human	8769349
glycogen(starch) synthase	2.4.1.11	Human	9126490
glycogen(starch) synthase	2.4.1.11	Human	9162607
glycogen(starch) synthase	2.4.1.11	Human	9267990
glycogen(starch) synthase	2.4.1.11	Human	9389424
glycogen(starch) synthase	2.4.1.11	Human	9450985
glycogen(starch) synthase	2.4.1.11	Human	9609122
glycogen(starch) synthase	2.4.1.11	Human	9712712

alpha-1,6-mannosyl-glycoprotein	2.4.1.143	Human	11250723
alpha-1,6-mannosyl-glycoprotein	2.4.1.143	Human	11552947
alpha-1,6-mannosyl-glycoprotein	2.4.1.143	Human	7841796
alpha-1,6-mannosyl-glycoprotein	2.4.1.143	Human	9028721
glucuronosyltransferase	2.4.1.17	Human	10353933
glucuronosyltransferase	2.4.1.17	Human	10427418
glucuronosyltransferase	2.4.1.17	Human	15710570
glucuronosyltransferase	2.4.1.17	Human	16623861
glucuronosyltransferase	2.4.1.17	Human	4715994
glucuronosyltransferase	2.4.1.17	Human	7900959
lactosylceramide 1,3-N-acetyl-beta-D-glucosaminyltransferase	2.4.1.206	Human	2564417
lactose synthase	2.4.1.22	Human	16664466
lactose synthase	2.4.1.22	Human	6766957
lactose synthase	2.4.1.22	Human	7539442
lactose synthase	2.4.1.22	Human	9387870
amidophosphoribosyltransferase	2.4.2.14	Human	10675983
amidophosphoribosyltransferase	2.4.2.14	Human	11158364
amidophosphoribosyltransferase	2.4.2.14	Human	12930749
amidophosphoribosyltransferase	2.4.2.14	Human	15266056
amidophosphoribosyltransferase	2.4.2.14	Human	17434429
amidophosphoribosyltransferase	2.4.2.14	Human	214373
amidophosphoribosyltransferase	2.4.2.14	Human	6327016
amidophosphoribosyltransferase	2.4.2.14	Human	701284
amidophosphoribosyltransferase	2.4.2.14	Human	7683680
amidophosphoribosyltransferase	2.4.2.14	Human	8150282
amidophosphoribosyltransferase	2.4.2.14	Human	8197456
amidophosphoribosyltransferase	2.4.2.14	Human	8197457
amidophosphoribosyltransferase	2.4.2.14	Human	8197458
amidophosphoribosyltransferase	2.4.2.14	Human	8380692
amidophosphoribosyltransferase	2.4.2.14	Human	8463258
amidophosphoribosyltransferase	2.4.2.14	Human	8809759
amidophosphoribosyltransferase	2.4.2.14	Human	8976092
amidophosphoribosyltransferase	2.4.2.14	Human	9615746
amidophosphoribosyltransferase	2.4.2.14	Human	9881055
thymidine phosphorylase	2.4.2.4	Human	10853015
thymidine phosphorylase	2.4.2.4	Human	11530879
thymidine phosphorylase	2.4.2.4	Human	11585972
thymidine phosphorylase	2.4.2.4	Human	12680231
thymidine phosphorylase	2.4.2.4	Human	15134221
thymidine phosphorylase	2.4.2.4	Human	15289834
thymidine phosphorylase	2.4.2.4	Human	15917420
thymidine phosphorylase	2.4.2.4	Human	16302736

adenine phosphoribosyltransferase	2.4.2.7	Human	2154328
adenine phosphoribosyltransferase	2.4.2.7	Human	6327016
dimethylallyltranstransferase	2.5.1.1	Human	11442630
dimethylallyltranstransferase	2.5.1.1	Human	14512521
dimethylallyltranstransferase	2.5.1.1	Human	15597200
dimethylallyltranstransferase	2.5.1.1	Human	7697819
dimethylallyltranstransferase	2.5.1.1	Human	7843406
dimethylallyltranstransferase	2.5.1.1	Human	8631820
geranyltranstransferase	2.5.1.10	Human	10484604
geranyltranstransferase	2.5.1.10	Human	11202437
geranyltranstransferase	2.5.1.10	Human	15459425
geranyltranstransferase	2.5.1.10	Human	15605175
geranyltranstransferase	2.5.1.10	Human	15713990
geranyltranstransferase	2.5.1.10	Human	15827605
geranyltranstransferase	2.5.1.10	Human	15827618
geranyltranstransferase	2.5.1.10	Human	16179378
geranyltranstransferase	2.5.1.10	Human	16932286
geranyltranstransferase	2.5.1.10	Human	1779710
geranyltranstransferase	2.5.1.10	Human	9061016
geranyltranstransferase	2.5.1.10	Human	9640665
spermidine synthase	2.5.1.16	Human	16515550
spermidine synthase	2.5.1.16	Human	2775206
glutathione transferase	2.5.1.18	Human	12484753
glutathione transferase	2.5.1.18	Human	15604283
glutathione transferase	2.5.1.18	Human	15761769
glutathione transferase	2.5.1.18	Human	16328982
glutathione transferase	2.5.1.18	Human	17176043
glutathione transferase	2.5.1.18	Human	17397868
squalene synthase	2.5.1.21	Human	10649449
squalene synthase	2.5.1.21	Human	10677224
squalene synthase	2.5.1.21	Human	12114564
squalene synthase	2.5.1.21	Human	15356323
squalene synthase	2.5.1.21	Human	1601846
squalene synthase	2.5.1.21	Human	17016471
squalene synthase	2.5.1.21	Human	17531951
squalene synthase	2.5.1.21	Human	2068081
squalene synthase	2.5.1.21	Human	7766395
squalene synthase	2.5.1.21	Human	7843406
squalene synthase	2.5.1.21	Human	9070296
methionine adenosyltransferase	2.5.1.6	Human	10415148
methionine adenosyltransferase	2.5.1.6	Human	1511738
methionine adenosyltransferase	2.5.1.6	Human	16413417
methionine adenosyltransferase	2.5.1.6	Human	2764959

methionine adenosyltransferase	2.5.1.6	Human	7980467
hydroxymethylbilane synthase	2.5.1.61	Human	10546563
hydroxymethylbilane synthase	2.5.1.61	Human	10787385
hydroxymethylbilane synthase	2.5.1.61	Human	11953837
hydroxymethylbilane synthase	2.5.1.61	Human	14559249
hydroxymethylbilane synthase	2.5.1.61	Human	1522882
hydroxymethylbilane synthase	2.5.1.61	Human	16886091
hydroxymethylbilane synthase	2.5.1.61	Human	4067519
hydroxymethylbilane synthase	2.5.1.61	Human	7326026
hydroxymethylbilane synthase	2.5.1.61	Human	7682572
hydroxymethylbilane synthase	2.5.1.61	Human	8023693
hydroxymethylbilane synthase	2.5.1.61	Human	9065797
hydroxymethylbilane synthase	2.5.1.61	Human	9460994
aspartate transaminase	2.6.1.1	Human	8580353
ornithine aminotransferase	2.6.1.13	Human	11691635
ornithine aminotransferase	2.6.1.13	Human	12462748
ornithine aminotransferase	2.6.1.13	Human	7883744
glutamine---fructose-6-phosphate transaminase (isomerizing)	2.6.1.16	Human	10329452
glutamine---fructose-6-phosphate transaminase (isomerizing)	2.6.1.16	Human	10865863
glutamine---fructose-6-phosphate transaminase (isomerizing)	2.6.1.16	Human	11270676
glutamine---fructose-6-phosphate transaminase (isomerizing)	2.6.1.16	Human	11895440
glutamine---fructose-6-phosphate transaminase (isomerizing)	2.6.1.16	Human	15158264
glutamine---fructose-6-phosphate transaminase (isomerizing)	2.6.1.16	Human	15308130
glutamine---fructose-6-phosphate transaminase (isomerizing)	2.6.1.16	Human	15613679
glutamine---fructose-6-phosphate transaminase (isomerizing)	2.6.1.16	Human	17941647
glutamine---fructose-6-phosphate transaminase (isomerizing)	2.6.1.16	Human	6184359
glutamine---fructose-6-phosphate transaminase (isomerizing)	2.6.1.16	Human	8394312
glutamine---fructose-6-phosphate transaminase (isomerizing)	2.6.1.16	Human	9421478
tyrosine transaminase	2.6.1.5	Human	1348057
tyrosine transaminase	2.6.1.5	Human	1526942
tyrosine transaminase	2.6.1.5	Human	240411
tyrosine transaminase	2.6.1.5	Human	2870018

tyrosine transaminase	2.6.1.5	Human	6123525
tyrosine transaminase	2.6.1.5	Human	9089286
tyrosine transaminase	2.6.1.5	Human	9228277
hexokinase	2.7.1.1	Human	11319725
hexokinase	2.7.1.1	Human	11391834
hexokinase	2.7.1.1	Human	11828256
hexokinase	2.7.1.1	Human	12660493
hexokinase	2.7.1.1	Human	1331693
hexokinase	2.7.1.1	Human	14672622
hexokinase	2.7.1.1	Human	15607940
hexokinase	2.7.1.1	Human	6341787
hexokinase	2.7.1.1	Human	6363888
hexokinase	2.7.1.1	Human	6440018
hexokinase	2.7.1.1	Human	6993859
hexokinase	2.7.1.1	Human	8027295
hexokinase	2.7.1.1	Human	9523722
6-phosphofructokinase	2.7.1.11	Human	10323269
6-phosphofructokinase	2.7.1.11	Human	10444344
6-phosphofructokinase	2.7.1.11	Human	10742704
6-phosphofructokinase	2.7.1.11	Human	10909961
6-phosphofructokinase	2.7.1.11	Human	10931197
6-phosphofructokinase	2.7.1.11	Human	1100622
6-phosphofructokinase	2.7.1.11	Human	1100623
6-phosphofructokinase	2.7.1.11	Human	11014908
6-phosphofructokinase	2.7.1.11	Human	11045948
6-phosphofructokinase	2.7.1.11	Human	11058792
6-phosphofructokinase	2.7.1.11	Human	11391835
6-phosphofructokinase	2.7.1.11	Human	11391836
6-phosphofructokinase	2.7.1.11	Human	11391837
6-phosphofructokinase	2.7.1.11	Human	11560513
6-phosphofructokinase	2.7.1.11	Human	12023862
6-phosphofructokinase	2.7.1.11	Human	12051897
6-phosphofructokinase	2.7.1.11	Human	12125051
6-phosphofructokinase	2.7.1.11	Human	12453221
6-phosphofructokinase	2.7.1.11	Human	131232
6-phosphofructokinase	2.7.1.11	Human	14585511
6-phosphofructokinase	2.7.1.11	Human	147929
6-phosphofructokinase	2.7.1.11	Human	149128
6-phosphofructokinase	2.7.1.11	Human	15157773
6-phosphofructokinase	2.7.1.11	Human	15466668
6-phosphofructokinase	2.7.1.11	Human	15504384
6-phosphofructokinase	2.7.1.11	Human	156307
6-phosphofructokinase	2.7.1.11	Human	15991998

6-phosphofructokinase	2.7.1.11	Human	16088331
6-phosphofructokinase	2.7.1.11	Human	16103521
6-phosphofructokinase	2.7.1.11	Human	16115917
6-phosphofructokinase	2.7.1.11	Human	16346876
6-phosphofructokinase	2.7.1.11	Human	16377227
6-phosphofructokinase	2.7.1.11	Human	1658253
6-phosphofructokinase	2.7.1.11	Human	16593209
6-phosphofructokinase	2.7.1.11	Human	1825156
6-phosphofructokinase	2.7.1.11	Human	1828673
6-phosphofructokinase	2.7.1.11	Human	1830744
6-phosphofructokinase	2.7.1.11	Human	1833303
6-phosphofructokinase	2.7.1.11	Human	191426
6-phosphofructokinase	2.7.1.11	Human	2137204
6-phosphofructokinase	2.7.1.11	Human	2147292
6-phosphofructokinase	2.7.1.11	Human	2149746
6-phosphofructokinase	2.7.1.11	Human	2434517
6-phosphofructokinase	2.7.1.11	Human	2502581
6-phosphofructokinase	2.7.1.11	Human	2522395
6-phosphofructokinase	2.7.1.11	Human	2527305
6-phosphofructokinase	2.7.1.11	Human	2820531
6-phosphofructokinase	2.7.1.11	Human	28629
6-phosphofructokinase	2.7.1.11	Human	2933146
6-phosphofructokinase	2.7.1.11	Human	2935776
6-phosphofructokinase	2.7.1.11	Human	2938549
6-phosphofructokinase	2.7.1.11	Human	2956156
6-phosphofructokinase	2.7.1.11	Human	2963653
6-phosphofructokinase	2.7.1.11	Human	2972577
6-phosphofructokinase	2.7.1.11	Human	2981949
6-phosphofructokinase	2.7.1.11	Human	3364152
6-phosphofructokinase	2.7.1.11	Human	3407760
6-phosphofructokinase	2.7.1.11	Human	356174
6-phosphofructokinase	2.7.1.11	Human	3768440
6-phosphofructokinase	2.7.1.11	Human	3931461
6-phosphofructokinase	2.7.1.11	Human	4030556
6-phosphofructokinase	2.7.1.11	Human	4243437
6-phosphofructokinase	2.7.1.11	Human	4252961
6-phosphofructokinase	2.7.1.11	Human	6093562
6-phosphofructokinase	2.7.1.11	Human	6133774
6-phosphofructokinase	2.7.1.11	Human	6211175
6-phosphofructokinase	2.7.1.11	Human	6231923
6-phosphofructokinase	2.7.1.11	Human	6232272
6-phosphofructokinase	2.7.1.11	Human	6234885
6-phosphofructokinase	2.7.1.11	Human	6279392

6-phosphofructokinase	2.7.1.11	Human	6325266
6-phosphofructokinase	2.7.1.11	Human	6331422
6-phosphofructokinase	2.7.1.11	Human	6440018
6-phosphofructokinase	2.7.1.11	Human	6444231
6-phosphofructokinase	2.7.1.11	Human	6452426
6-phosphofructokinase	2.7.1.11	Human	6779470
6-phosphofructokinase	2.7.1.11	Human	7233512
6-phosphofructokinase	2.7.1.11	Human	7440254
6-phosphofructokinase	2.7.1.11	Human	7522206
6-phosphofructokinase	2.7.1.11	Human	7589825
6-phosphofructokinase	2.7.1.11	Human	7602786
6-phosphofructokinase	2.7.1.11	Human	7710770
6-phosphofructokinase	2.7.1.11	Human	7875554
6-phosphofructokinase	2.7.1.11	Human	8224738
6-phosphofructokinase	2.7.1.11	Human	8366430
6-phosphofructokinase	2.7.1.11	Human	8514849
6-phosphofructokinase	2.7.1.11	Human	8557664
6-phosphofructokinase	2.7.1.11	Human	8593533
6-phosphofructokinase	2.7.1.11	Human	8643924
6-phosphofructokinase	2.7.1.11	Human	8910548
6-phosphofructokinase	2.7.1.11	Human	8981075
6-phosphofructokinase	2.7.1.11	Human	9267516
6-phosphofructokinase	2.7.1.11	Human	9287040
6-phosphofructokinase	2.7.1.11	Human	9329694
6-phosphofructokinase	2.7.1.11	Human	9371084
6-phosphofructokinase	2.7.1.11	Human	9439886
6-phosphofructokinase	2.7.1.11	Human	9447322
6-phosphofructokinase	2.7.1.11	Human	9555897
6-phosphofructokinase	2.7.1.11	Human	9580251
6-phosphofructokinase	2.7.1.11	Human	9580875
6-phosphofructokinase	2.7.1.11	Human	9608547
6-phosphofructokinase	2.7.1.11	Human	9766212
6-phosphofructokinase	2.7.1.11	Human	9777012
6-phosphofructokinase	2.7.1.11	Human	9851886
6-phosphofructokinase	2.7.1.11	Human	9973548
inositol-trisphosphate 3-kinase	2.7.1.127	Human	15837423
inositol-trisphosphate 3-kinase	2.7.1.127	Human	9211876
inositol-tetrakisphosphate 1-kinase	2.7.1.134	Human	11533064
inositol-tetrakisphosphate 1-kinase	2.7.1.134	Human	15837423
phosphatidylinositol 3-kinase	2.7.1.137	Human	10869418
phosphatidylinositol 3-kinase	2.7.1.137	Human	10874027
phosphatidylinositol 3-kinase	2.7.1.137	Human	10998146
phosphatidylinositol 3-kinase	2.7.1.137	Human	11259761

phosphatidylinositol 3-kinase	2.7.1.137	Human	11744698
phosphatidylinositol 3-kinase	2.7.1.137	Human	7669049
phosphatidylinositol 3-kinase	2.7.1.137	Human	9292730
phosphatidylinositol 3-kinase	2.7.1.137	Human	9312149
phosphatidylinositol 3-kinase	2.7.1.137	Human	9478990
phosphatidylinositol 3-kinase	2.7.1.137	Human	9826526
phosphatidylinositol 3-kinase	2.7.1.137	Human	9826674
glucokinase	2.7.1.2	Human	10456334
glucokinase	2.7.1.2	Human	10494657
glucokinase	2.7.1.2	Human	10905475
glucokinase	2.7.1.2	Human	11311143
glucokinase	2.7.1.2	Human	11947549
glucokinase	2.7.1.2	Human	11950391
glucokinase	2.7.1.2	Human	12941786
glucokinase	2.7.1.2	Human	14979565
glucokinase	2.7.1.2	Human	15009676
glucokinase	2.7.1.2	Human	15016359
glucokinase	2.7.1.2	Human	15134337
glucokinase	2.7.1.2	Human	15226592
glucokinase	2.7.1.2	Human	15277402
glucokinase	2.7.1.2	Human	1545870
glucokinase	2.7.1.2	Human	15707679
glucokinase	2.7.1.2	Human	15955369
glucokinase	2.7.1.2	Human	16186394
glucokinase	2.7.1.2	Human	16834571
glucokinase	2.7.1.2	Human	16899262
glucokinase	2.7.1.2	Human	16916947
glucokinase	2.7.1.2	Human	2210070
glucokinase	2.7.1.2	Human	2584235
glucokinase	2.7.1.2	Human	2682629
glucokinase	2.7.1.2	Human	6780351
glucokinase	2.7.1.2	Human	6836273
glucokinase	2.7.1.2	Human	7010073
glucokinase	2.7.1.2	Human	7553875
glucokinase	2.7.1.2	Human	7821741
glucokinase	2.7.1.2	Human	7983782
glucokinase	2.7.1.2	Human	8194664
glucokinase	2.7.1.2	Human	8344416
glucokinase	2.7.1.2	Human	8433729
glucokinase	2.7.1.2	Human	8446591
glucokinase	2.7.1.2	Human	8549869
glucokinase	2.7.1.2	Human	8550593
glucokinase	2.7.1.2	Human	8631975

glucokinase	2.7.1.2	Human	8690154
glucokinase	2.7.1.2	Human	8692940
glucokinase	2.7.1.2	Human	8751724
glucokinase	2.7.1.2	Human	9113996
glucokinase	2.7.1.2	Human	9460079
adenosine kinase	2.7.1.20	Human	10794412
adenosine kinase	2.7.1.20	Human	12228764
adenosine kinase	2.7.1.20	Human	15632276
adenosine kinase	2.7.1.20	Human	2154328
adenosine kinase	2.7.1.20	Human	8184939
thymidine kinase	2.7.1.21	Human	12750029
thymidine kinase	2.7.1.21	Human	12750297
thymidine kinase	2.7.1.21	Human	1337357
thymidine kinase	2.7.1.21	Human	1455450
thymidine kinase	2.7.1.21	Human	14770427
thymidine kinase	2.7.1.21	Human	15196542
thymidine kinase	2.7.1.21	Human	1525336
thymidine kinase	2.7.1.21	Human	1531285
thymidine kinase	2.7.1.21	Human	15809747
thymidine kinase	2.7.1.21	Human	16336273
thymidine kinase	2.7.1.21	Human	16473525
thymidine kinase	2.7.1.21	Human	17065087
thymidine kinase	2.7.1.21	Human	2025497
thymidine kinase	2.7.1.21	Human	348202
thymidine kinase	2.7.1.21	Human	3558173
thymidine kinase	2.7.1.21	Human	6684556
thymidine kinase	2.7.1.21	Human	6840218
thymidine kinase	2.7.1.21	Human	8016290
thymidine kinase	2.7.1.21	Human	8140585
thymidine kinase	2.7.1.21	Human	8432201
thymidine kinase	2.7.1.21	Human	8878781
thymidine kinase	2.7.1.21	Human	8941385
thymidine kinase	2.7.1.21	Human	8955897
thymidine kinase	2.7.1.21	Human	9154996
thymidine kinase	2.7.1.21	Human	956273
thymidine kinase	2.7.1.21	Human	9661884
thymidine kinase	2.7.1.21	Human	9816259
riboflavin kinase	2.7.1.26	Human	6138398
glycerol kinase	2.7.1.30	Human	10.1002/anie.198810401
glycerol kinase	2.7.1.30	Human	11388799
glycerol kinase	2.7.1.30	Human	11811537
glycerol kinase	2.7.1.30	Human	1985967
glycerol kinase	2.7.1.30	Human	2547969

glycerol kinase	2.7.1.30	Human	4914079
glycerol kinase	2.7.1.30	Human	6292169
glycerol kinase	2.7.1.30	Human	6440018
glycerol kinase	2.7.1.30	Human	9162046
choline kinase	2.7.1.32	Human	1336121
choline kinase	2.7.1.32	Human	16490392
choline kinase	2.7.1.32	Human	2153442
choline kinase	2.7.1.32	Human	217369
choline kinase	2.7.1.32	Human	3365445
choline kinase	2.7.1.32	Human	3447597
choline kinase	2.7.1.32	Human	5495730
choline kinase	2.7.1.32	Human	6503617
choline kinase	2.7.1.32	Human	8182083
choline kinase	2.7.1.32	Human	828054
choline kinase	2.7.1.32	Human	8414498
pantothenate kinase	2.7.1.33	Human	10625688
pantothenate kinase	2.7.1.33	Human	11809413
pantothenate kinase	2.7.1.33	Human	12697433
pantothenate kinase	2.7.1.33	Human	15176870
pantothenate kinase	2.7.1.33	Human	15843025
pantothenate kinase	2.7.1.33	Human	16701556
pantothenate kinase	2.7.1.33	Human	17323930
pantothenate kinase	2.7.1.33	Human	17581817
pantothenate kinase	2.7.1.33	Human	9890959
pyruvate kinase	2.7.1.40	Human	101523
pyruvate kinase	2.7.1.40	Human	11181519
pyruvate kinase	2.7.1.40	Human	1175605
pyruvate kinase	2.7.1.40	Human	1328007
pyruvate kinase	2.7.1.40	Human	1406667
pyruvate kinase	2.7.1.40	Human	15028426
pyruvate kinase	2.7.1.40	Human	15567985
pyruvate kinase	2.7.1.40	Human	16046853
pyruvate kinase	2.7.1.40	Human	16511150
pyruvate kinase	2.7.1.40	Human	16549526
pyruvate kinase	2.7.1.40	Human	1959479
pyruvate kinase	2.7.1.40	Human	2387024
pyruvate kinase	2.7.1.40	Human	2813362
pyruvate kinase	2.7.1.40	Human	2820531
pyruvate kinase	2.7.1.40	Human	2846196
pyruvate kinase	2.7.1.40	Human	291050
pyruvate kinase	2.7.1.40	Human	29278
pyruvate kinase	2.7.1.40	Human	2935776
pyruvate kinase	2.7.1.40	Human	2970638

pyruvate kinase	2.7.1.40	Human	3023262
pyruvate kinase	2.7.1.40	Human	3032541
pyruvate kinase	2.7.1.40	Human	3159473
pyruvate kinase	2.7.1.40	Human	3161219
pyruvate kinase	2.7.1.40	Human	3350145
pyruvate kinase	2.7.1.40	Human	4053567
pyruvate kinase	2.7.1.40	Human	6222515
pyruvate kinase	2.7.1.40	Human	6241274
pyruvate kinase	2.7.1.40	Human	6268138
pyruvate kinase	2.7.1.40	Human	6370232
pyruvate kinase	2.7.1.40	Human	6588273
pyruvate kinase	2.7.1.40	Human	6682991
pyruvate kinase	2.7.1.40	Human	6713301
pyruvate kinase	2.7.1.40	Human	7357032
pyruvate kinase	2.7.1.40	Human	7961441
pyruvate kinase	2.7.1.40	Human	8074527
pyruvate kinase	2.7.1.40	Human	8144600
pyruvate kinase	2.7.1.40	Human	8436141
pyruvate kinase	2.7.1.40	Human	8476115
pyruvate kinase	2.7.1.40	Human	8765986
pyruvate kinase	2.7.1.40	Human	9252361
uridine kinase	2.7.1.48	Human	15735337
uridine kinase	2.7.1.48	Human	195585
uridine kinase	2.7.1.48	Human	9923963
galactokinase	2.7.1.6	Human	12694189
galactokinase	2.7.1.6	Human	14596685
galactokinase	2.7.1.6	Human	14763977
galactokinase	2.7.1.6	Human	16452467
galactokinase	2.7.1.6	Human	6836273
N-acylmannosamine kinase	2.7.1.60	Human	15987957
1-phosphatidylinositol 4-kinase	2.7.1.67	Human	12594831
1-phosphatidylinositol 4-kinase	2.7.1.67	Human	12620118
1-phosphatidylinositol 4-kinase	2.7.1.67	Human	16912074
1-phosphatidylinositol 4-kinase	2.7.1.67	Human	17003043
1-phosphatidylinositol 4-kinase	2.7.1.67	Human	7961848
1-phosphatidylinositol 4-kinase	2.7.1.67	Human	8152413
1-phosphatidylinositol 4-kinase	2.7.1.67	Human	8190262
1-phosphatidylinositol 4-kinase	2.7.1.67	Human	9654085
1-phosphatidylinositol 4-kinase	2.7.1.67	Human	9854149
1-phosphatidylinositol 4-kinase	2.7.1.67	Human	9891985
1-phosphatidylinositol-4-phosphate 5-kinase	2.7.1.68	Human	11098053
1-phosphatidylinositol-4-phosphate 5-kinase	2.7.1.68	Human	12620118
1-phosphatidylinositol-4-phosphate 5-kinase	2.7.1.68	Human	15277528

1-phosphatidylinositol-4-phosphate 5-kinase	2.7.1.68	Human	15738269
1-phosphatidylinositol-4-phosphate 5-kinase	2.7.1.68	Human	17635937
1-phosphatidylinositol-4-phosphate 5-kinase	2.7.1.68	Human	2849321
1-phosphatidylinositol-4-phosphate 5-kinase	2.7.1.68	Human	8190262
1-phosphatidylinositol-4-phosphate 5-kinase	2.7.1.68	Human	9292730
deoxycytidine kinase	2.7.1.74	Human	10499616
deoxycytidine kinase	2.7.1.74	Human	10848830
deoxycytidine kinase	2.7.1.74	Human	11888330
deoxycytidine kinase	2.7.1.74	Human	12054682
deoxycytidine kinase	2.7.1.74	Human	15561147
deoxycytidine kinase	2.7.1.74	Human	15803490
deoxycytidine kinase	2.7.1.74	Human	16180016
deoxycytidine kinase	2.7.1.74	Human	16421443
deoxycytidine kinase	2.7.1.74	Human	16463058
deoxycytidine kinase	2.7.1.74	Human	17065079
deoxycytidine kinase	2.7.1.74	Human	2436757
deoxycytidine kinase	2.7.1.74	Human	3335008
deoxycytidine kinase	2.7.1.74	Human	7805176
deoxycytidine kinase	2.7.1.74	Human	8616717
deoxycytidine kinase	2.7.1.74	Human	8718419
ethanolamine kinase	2.7.1.82	Human	11044454
ethanolamine kinase	2.7.1.82	Human	1480155
ethanolamine kinase	2.7.1.82	Human	475777
ethanolamine kinase	2.7.1.82	Human	7142139
sphinganine kinase	2.7.1.91	Human	10567432
sphinganine kinase	2.7.1.91	Human	14568343
sphinganine kinase	2.7.1.91	Human	15451787
sphinganine kinase	2.7.1.91	Human	16831409
glutamate 5-kinase	2.7.2.11	Human	12602867
glutamate 5-kinase	2.7.2.11	Human	15077666
glutamate 5-kinase	2.7.2.11	Human	17449694
adenylate kinase	2.7.4.3	Human	126626
adenylate kinase	2.7.4.3	Human	14656997
adenylate kinase	2.7.4.3	Human	15941717
adenylate kinase	2.7.4.3	Human	16668787
adenylate kinase	2.7.4.3	Human	6440018
adenylate kinase	2.7.4.3	Human	7764491
dTMP kinase	2.7.4.9	Human	164949
ribose-phosphate diphosphokinase	2.7.6.1	Human	15878857
ribose-phosphate diphosphokinase	2.7.6.1	Human	217337
nicotinamide-nucleotide adenylyltransferase	2.7.7.1	Human	11248244
nicotinamide-nucleotide adenylyltransferase	2.7.7.1	Human	11966442
nicotinamide-nucleotide adenylyltransferase	2.7.7.1	Human	17360427

choline-phosphate cytidylyltransferase	2.7.7.15	Human	10101264
choline-phosphate cytidylyltransferase	2.7.7.15	Human	10208837
choline-phosphate cytidylyltransferase	2.7.7.15	Human	10473578
choline-phosphate cytidylyltransferase	2.7.7.15	Human	10615073
choline-phosphate cytidylyltransferase	2.7.7.15	Human	10908674
choline-phosphate cytidylyltransferase	2.7.7.15	Human	10946015
choline-phosphate cytidylyltransferase	2.7.7.15	Human	11029581
choline-phosphate cytidylyltransferase	2.7.7.15	Human	11097182
choline-phosphate cytidylyltransferase	2.7.7.15	Human	11279002
choline-phosphate cytidylyltransferase	2.7.7.15	Human	11404252
choline-phosphate cytidylyltransferase	2.7.7.15	Human	11404253
choline-phosphate cytidylyltransferase	2.7.7.15	Human	11521967
choline-phosphate cytidylyltransferase	2.7.7.15	Human	11829742
choline-phosphate cytidylyltransferase	2.7.7.15	Human	12034570
choline-phosphate cytidylyltransferase	2.7.7.15	Human	12052891
choline-phosphate cytidylyltransferase	2.7.7.15	Human	12062780
choline-phosphate cytidylyltransferase	2.7.7.15	Human	12221122
choline-phosphate cytidylyltransferase	2.7.7.15	Human	12271462
choline-phosphate cytidylyltransferase	2.7.7.15	Human	12370080
choline-phosphate cytidylyltransferase	2.7.7.15	Human	12620118
choline-phosphate cytidylyltransferase	2.7.7.15	Human	12659631
choline-phosphate cytidylyltransferase	2.7.7.15	Human	12842190
choline-phosphate cytidylyltransferase	2.7.7.15	Human	12928431
choline-phosphate cytidylyltransferase	2.7.7.15	Human	14536058
choline-phosphate cytidylyltransferase	2.7.7.15	Human	15079868
choline-phosphate cytidylyltransferase	2.7.7.15	Human	15139854
choline-phosphate cytidylyltransferase	2.7.7.15	Human	15210848
choline-phosphate cytidylyltransferase	2.7.7.15	Human	15522825
choline-phosphate cytidylyltransferase	2.7.7.15	Human	15574675
choline-phosphate cytidylyltransferase	2.7.7.15	Human	15635091
choline-phosphate cytidylyltransferase	2.7.7.15	Human	15788406
choline-phosphate cytidylyltransferase	2.7.7.15	Human	15982005
choline-phosphate cytidylyltransferase	2.7.7.15	Human	16097951
choline-phosphate cytidylyltransferase	2.7.7.15	Human	16153613
choline-phosphate cytidylyltransferase	2.7.7.15	Human	16236026
choline-phosphate cytidylyltransferase	2.7.7.15	Human	16511521
choline-phosphate cytidylyltransferase	2.7.7.15	Human	16580250
choline-phosphate cytidylyltransferase	2.7.7.15	Human	16580875
choline-phosphate cytidylyltransferase	2.7.7.15	Human	2160812
choline-phosphate cytidylyltransferase	2.7.7.15	Human	2268410
choline-phosphate cytidylyltransferase	2.7.7.15	Human	2665794
choline-phosphate cytidylyltransferase	2.7.7.15	Human	2833508
choline-phosphate cytidylyltransferase	2.7.7.15	Human	2838058

choline-phosphate cytidylyltransferase	2.7.7.15	Human	3004590
choline-phosphate cytidylyltransferase	2.7.7.15	Human	3365445
choline-phosphate cytidylyltransferase	2.7.7.15	Human	3367156
choline-phosphate cytidylyltransferase	2.7.7.15	Human	3447597
choline-phosphate cytidylyltransferase	2.7.7.15	Human	603639
choline-phosphate cytidylyltransferase	2.7.7.15	Human	6134645
choline-phosphate cytidylyltransferase	2.7.7.15	Human	6243289
choline-phosphate cytidylyltransferase	2.7.7.15	Human	6477961
choline-phosphate cytidylyltransferase	2.7.7.15	Human	7126613
choline-phosphate cytidylyltransferase	2.7.7.15	Human	7487944
choline-phosphate cytidylyltransferase	2.7.7.15	Human	7588775
choline-phosphate cytidylyltransferase	2.7.7.15	Human	7637558
choline-phosphate cytidylyltransferase	2.7.7.15	Human	7768909
choline-phosphate cytidylyltransferase	2.7.7.15	Human	7782919
choline-phosphate cytidylyltransferase	2.7.7.15	Human	7836412
choline-phosphate cytidylyltransferase	2.7.7.15	Human	8006517
choline-phosphate cytidylyltransferase	2.7.7.15	Human	8182083
choline-phosphate cytidylyltransferase	2.7.7.15	Human	8239319
choline-phosphate cytidylyltransferase	2.7.7.15	Human	8255685
choline-phosphate cytidylyltransferase	2.7.7.15	Human	8387510
choline-phosphate cytidylyltransferase	2.7.7.15	Human	8504126
choline-phosphate cytidylyltransferase	2.7.7.15	Human	8597584
choline-phosphate cytidylyltransferase	2.7.7.15	Human	8626633
choline-phosphate cytidylyltransferase	2.7.7.15	Human	8663247
choline-phosphate cytidylyltransferase	2.7.7.15	Human	8756587
choline-phosphate cytidylyltransferase	2.7.7.15	Human	8761490
choline-phosphate cytidylyltransferase	2.7.7.15	Human	8810902
choline-phosphate cytidylyltransferase	2.7.7.15	Human	9009253
choline-phosphate cytidylyltransferase	2.7.7.15	Human	9046356
choline-phosphate cytidylyltransferase	2.7.7.15	Human	9148929
choline-phosphate cytidylyltransferase	2.7.7.15	Human	9335949
choline-phosphate cytidylyltransferase	2.7.7.15	Human	9370319
choline-phosphate cytidylyltransferase	2.7.7.15	Human	9421188
choline-phosphate cytidylyltransferase	2.7.7.15	Human	9714757
pantetheine-phosphate adenylyltransferase	2.7.7.3	Human	1746161
sulfate adenylyltransferase	2.7.7.4	Human	10759525
sulfate adenylyltransferase	2.7.7.4	Human	10956658
sulfate adenylyltransferase	2.7.7.4	Human	11157739
sulfate adenylyltransferase	2.7.7.4	Human	14613928
sulfate adenylyltransferase	2.7.7.4	Human	16008502
sulfate adenylyltransferase	2.7.7.4	Human	17095009
sulfate adenylyltransferase	2.7.7.4	Human	8206850
sulfate adenylyltransferase	2.7.7.4	Human	9880353

phosphatidate cytidylyltransferase	2.7.7.41	Human	11985865
phosphatidate cytidylyltransferase	2.7.7.41	Human	9345289
N-acylneuraminate cytidylyltransferase	2.7.7.43	Human	10320348
N-acylneuraminate cytidylyltransferase	2.7.7.43	Human	3024643
N-acylneuraminate cytidylyltransferase	2.7.7.43	Human	7830552
polyribonucleotide nucleotidyltransferase	2.7.7.8	Human	126862
UTP---glucose-1-phosphate uridylyltransferase	2.7.7.9	Human	11171080
UTP---glucose-1-phosphate uridylyltransferase	2.7.7.9	Human	1149741
UTP---glucose-1-phosphate uridylyltransferase	2.7.7.9	Human	12088504
UTP---glucose-1-phosphate uridylyltransferase	2.7.7.9	Human	15274139
alcohol sulfotransferase	2.8.2.2	Human	7900959
[heparan sulfate]-glucosamine 3-sulfotransferase 1	2.8.2.23	Human	12671048
sterol esterase	3.1.1.13	Human	10569995
sterol esterase	3.1.1.13	Human	15809341
sterol esterase	3.1.1.13	Human	8258956
triacylglycerol lipase	3.1.1.3	Human	11217140
triacylglycerol lipase	3.1.1.3	Human	12689525
triacylglycerol lipase	3.1.1.3	Human	3678753
lipoprotein lipase	3.1.1.34	Human	10077655
lipoprotein lipase	3.1.1.34	Human	10226565
lipoprotein lipase	3.1.1.34	Human	10364085
lipoprotein lipase	3.1.1.34	Human	10388470
lipoprotein lipase	3.1.1.34	Human	10515359
lipoprotein lipase	3.1.1.34	Human	10650951
lipoprotein lipase	3.1.1.34	Human	10704617
lipoprotein lipase	3.1.1.34	Human	10965219
lipoprotein lipase	3.1.1.34	Human	11158876
lipoprotein lipase	3.1.1.34	Human	11334409
lipoprotein lipase	3.1.1.34	Human	11432868
lipoprotein lipase	3.1.1.34	Human	11591230
lipoprotein lipase	3.1.1.34	Human	12079052
lipoprotein lipase	3.1.1.34	Human	12352010
lipoprotein lipase	3.1.1.34	Human	12847564
lipoprotein lipase	3.1.1.34	Human	12862202
lipoprotein lipase	3.1.1.34	Human	12934668
lipoprotein lipase	3.1.1.34	Human	1401083
lipoprotein lipase	3.1.1.34	Human	14580165
lipoprotein lipase	3.1.1.34	Human	14656997

lipoprotein lipase	3.1.1.34	Human	14660566
lipoprotein lipase	3.1.1.34	Human	15178298
lipoprotein lipase	3.1.1.34	Human	15262189
lipoprotein lipase	3.1.1.34	Human	15320848
lipoprotein lipase	3.1.1.34	Human	15562391
lipoprotein lipase	3.1.1.34	Human	15697220
lipoprotein lipase	3.1.1.34	Human	15801017
lipoprotein lipase	3.1.1.34	Human	16195388
lipoprotein lipase	3.1.1.34	Human	16416313
lipoprotein lipase	3.1.1.34	Human	16531751
lipoprotein lipase	3.1.1.34	Human	16767221
lipoprotein lipase	3.1.1.34	Human	1737833
lipoprotein lipase	3.1.1.34	Human	1999438
lipoprotein lipase	3.1.1.34	Human	2117022
lipoprotein lipase	3.1.1.34	Human	2165281
lipoprotein lipase	3.1.1.34	Human	2765496
lipoprotein lipase	3.1.1.34	Human	3304415
lipoprotein lipase	3.1.1.34	Human	3519325
lipoprotein lipase	3.1.1.34	Human	3817303
lipoprotein lipase	3.1.1.34	Human	3920639
lipoprotein lipase	3.1.1.34	Human	3950770
lipoprotein lipase	3.1.1.34	Human	4077022
lipoprotein lipase	3.1.1.34	Human	6482738
lipoprotein lipase	3.1.1.34	Human	7033153
lipoprotein lipase	3.1.1.34	Human	7126037
lipoprotein lipase	3.1.1.34	Human	7229033
lipoprotein lipase	3.1.1.34	Human	7276825
lipoprotein lipase	3.1.1.34	Human	7592875
lipoprotein lipase	3.1.1.34	Human	7630312
lipoprotein lipase	3.1.1.34	Human	7939219
lipoprotein lipase	3.1.1.34	Human	7956906
lipoprotein lipase	3.1.1.34	Human	8371063
lipoprotein lipase	3.1.1.34	Human	8374222
lipoprotein lipase	3.1.1.34	Human	8422428
lipoprotein lipase	3.1.1.34	Human	8480620
lipoprotein lipase	3.1.1.34	Human	8729382
lipoprotein lipase	3.1.1.34	Human	8882874
lipoprotein lipase	3.1.1.34	Human	8919277
lipoprotein lipase	3.1.1.34	Human	9188470
lipoprotein lipase	3.1.1.34	Human	9193431
lipoprotein lipase	3.1.1.34	Human	9264396
lipoprotein lipase	3.1.1.34	Human	9294198
lipoprotein lipase	3.1.1.34	Human	9298816

lipoprotein lipase	3.1.1.34	Human	9351402
lipoprotein lipase	3.1.1.34	Human	9358077
lipoprotein lipase	3.1.1.34	Human	9382958
lipoprotein lipase	3.1.1.34	Human	9495276
lipoprotein lipase	3.1.1.34	Human	9727057
lipoprotein lipase	3.1.1.34	Human	9888641
lipoprotein lipase	3.1.1.34	Human	9888650
lipoprotein lipase	3.1.1.34	Human	9924194
lipoprotein lipase	3.1.1.34	Human	9973300
phospholipase A2	3.1.1.4	Human	10435206
phospholipase A2	3.1.1.4	Human	10482042
phospholipase A2	3.1.1.4	Human	10614936
phospholipase A2	3.1.1.4	Human	106389
phospholipase A2	3.1.1.4	Human	10749741
phospholipase A2	3.1.1.4	Human	10793641
phospholipase A2	3.1.1.4	Human	10919502
phospholipase A2	3.1.1.4	Human	10970711
phospholipase A2	3.1.1.4	Human	11080682
phospholipase A2	3.1.1.4	Human	11085935
phospholipase A2	3.1.1.4	Human	11099485
phospholipase A2	3.1.1.4	Human	11115401
phospholipase A2	3.1.1.4	Human	11328947
phospholipase A2	3.1.1.4	Human	11374398
phospholipase A2	3.1.1.4	Human	11964173
phospholipase A2	3.1.1.4	Human	12076714
phospholipase A2	3.1.1.4	Human	12143044
phospholipase A2	3.1.1.4	Human	12189011
phospholipase A2	3.1.1.4	Human	124658
phospholipase A2	3.1.1.4	Human	12724134
phospholipase A2	3.1.1.4	Human	12855693
phospholipase A2	3.1.1.4	Human	1322564
phospholipase A2	3.1.1.4	Human	1400321
phospholipase A2	3.1.1.4	Human	1410519
phospholipase A2	3.1.1.4	Human	14561756
phospholipase A2	3.1.1.4	Human	15041029
phospholipase A2	3.1.1.4	Human	1510970
phospholipase A2	3.1.1.4	Human	15211007
phospholipase A2	3.1.1.4	Human	15214789
phospholipase A2	3.1.1.4	Human	15283760
phospholipase A2	3.1.1.4	Human	15377291
phospholipase A2	3.1.1.4	Human	15472477
phospholipase A2	3.1.1.4	Human	15521009
phospholipase A2	3.1.1.4	Human	15743759

phospholipase A2	3.1.1.4	Human	15900018
phospholipase A2	3.1.1.4	Human	16179540
phospholipase A2	3.1.1.4	Human	16221889
phospholipase A2	3.1.1.4	Human	16318667
phospholipase A2	3.1.1.4	Human	16443193
phospholipase A2	3.1.1.4	Human	16716827
phospholipase A2	3.1.1.4	Human	16754327
phospholipase A2	3.1.1.4	Human	16794537
phospholipase A2	3.1.1.4	Human	16968951
phospholipase A2	3.1.1.4	Human	17008548
phospholipase A2	3.1.1.4	Human	1762050
phospholipase A2	3.1.1.4	Human	1836009
phospholipase A2	3.1.1.4	Human	1976627
phospholipase A2	3.1.1.4	Human	2075195
phospholipase A2	3.1.1.4	Human	2217203
phospholipase A2	3.1.1.4	Human	2218714
phospholipase A2	3.1.1.4	Human	2223919
phospholipase A2	3.1.1.4	Human	2250570
phospholipase A2	3.1.1.4	Human	2354835
phospholipase A2	3.1.1.4	Human	2454329
phospholipase A2	3.1.1.4	Human	2646218
phospholipase A2	3.1.1.4	Human	2742867
phospholipase A2	3.1.1.4	Human	2841909
phospholipase A2	3.1.1.4	Human	2848583
phospholipase A2	3.1.1.4	Human	3085592
phospholipase A2	3.1.1.4	Human	3141409
phospholipase A2	3.1.1.4	Human	3164726
phospholipase A2	3.1.1.4	Human	3240001
phospholipase A2	3.1.1.4	Human	3343241
phospholipase A2	3.1.1.4	Human	3745162
phospholipase A2	3.1.1.4	Human	386140
phospholipase A2	3.1.1.4	Human	6135325
phospholipase A2	3.1.1.4	Human	6527548
phospholipase A2	3.1.1.4	Human	6955805
phospholipase A2	3.1.1.4	Human	7539396
phospholipase A2	3.1.1.4	Human	7649158
phospholipase A2	3.1.1.4	Human	7681828
phospholipase A2	3.1.1.4	Human	7835820
phospholipase A2	3.1.1.4	Human	7938094
phospholipase A2	3.1.1.4	Human	7945230
phospholipase A2	3.1.1.4	Human	7998975
phospholipase A2	3.1.1.4	Human	8067978
phospholipase A2	3.1.1.4	Human	8148385

phospholipase A2	3.1.1.4	Human	8278618
phospholipase A2	3.1.1.4	Human	8280164
phospholipase A2	3.1.1.4	Human	8292024
phospholipase A2	3.1.1.4	Human	8307447
phospholipase A2	3.1.1.4	Human	8307472
phospholipase A2	3.1.1.4	Human	8347632
phospholipase A2	3.1.1.4	Human	8536274
phospholipase A2	3.1.1.4	Human	8570769
phospholipase A2	3.1.1.4	Human	8648901
phospholipase A2	3.1.1.4	Human	8739397
phospholipase A2	3.1.1.4	Human	8773214
phospholipase A2	3.1.1.4	Human	8808111
phospholipase A2	3.1.1.4	Human	8832056
phospholipase A2	3.1.1.4	Human	8863185
phospholipase A2	3.1.1.4	Human	8865467
phospholipase A2	3.1.1.4	Human	8873778
phospholipase A2	3.1.1.4	Human	8888134
phospholipase A2	3.1.1.4	Human	9172747
phospholipase A2	3.1.1.4	Human	9173912
phospholipase A2	3.1.1.4	Human	9187306
phospholipase A2	3.1.1.4	Human	9219895
phospholipase A2	3.1.1.4	Human	9221748
phospholipase A2	3.1.1.4	Human	9275050
phospholipase A2	3.1.1.4	Human	9314599
phospholipase A2	3.1.1.4	Human	9322315
phospholipase A2	3.1.1.4	Human	9398170
phospholipase A2	3.1.1.4	Human	9410883
phospholipase A2	3.1.1.4	Human	9425915
phospholipase A2	3.1.1.4	Human	9448723
phospholipase A2	3.1.1.4	Human	9463402
phospholipase A2	3.1.1.4	Human	9513902
phospholipase A2	3.1.1.4	Human	9538252
phospholipase A2	3.1.1.4	Human	9562240
phospholipase A2	3.1.1.4	Human	9585093
phospholipase A2	3.1.1.4	Human	9764845
phospholipase A2	3.1.1.4	Human	9879666
acetylcholinesterase	3.1.1.7	Human	10766776
acetylcholinesterase	3.1.1.7	Human	12415870
acetylcholinesterase	3.1.1.7	Human	15936353
acetylcholinesterase	3.1.1.7	Human	17562604
acetylcholinesterase	3.1.1.7	Human	1820094
acetylcholinesterase	3.1.1.7	Human	2587621
acetylcholinesterase	3.1.1.7	Human	4795368

acetylcholinesterase	3.1.1.7	Human	668
acetylcholinesterase	3.1.1.7	Human	9109840
acetylcholinesterase	3.1.1.7	Human	9548556
alkaline phosphatase	3.1.3.1	Human	11029583
alkaline phosphatase	3.1.3.1	Human	11139445
alkaline phosphatase	3.1.3.1	Human	12412807
alkaline phosphatase	3.1.3.1	Human	1445337
alkaline phosphatase	3.1.3.1	Human	14560000
alkaline phosphatase	3.1.3.1	Human	3509742
alkaline phosphatase	3.1.3.1	Human	7032602
fructose-bisphosphatase	3.1.3.11	Human	10327613
fructose-bisphosphatase	3.1.3.11	Human	10393302
fructose-bisphosphatase	3.1.3.11	Human	10773464
fructose-bisphosphatase	3.1.3.11	Human	11536627
fructose-bisphosphatase	3.1.3.11	Human	11864619
fructose-bisphosphatase	3.1.3.11	Human	12190028
fructose-bisphosphatase	3.1.3.11	Human	12686616
fructose-bisphosphatase	3.1.3.11	Human	15225753
fructose-bisphosphatase	3.1.3.11	Human	15498578
fructose-bisphosphatase	3.1.3.11	Human	15631980
fructose-bisphosphatase	3.1.3.11	Human	16199065
fructose-bisphosphatase	3.1.3.11	Human	16580859
fructose-bisphosphatase	3.1.3.11	Human	16593209
fructose-bisphosphatase	3.1.3.11	Human	16657971
fructose-bisphosphatase	3.1.3.11	Human	16814784
fructose-bisphosphatase	3.1.3.11	Human	16857246
fructose-bisphosphatase	3.1.3.11	Human	2835013
fructose-bisphosphatase	3.1.3.11	Human	2983680
fructose-bisphosphatase	3.1.3.11	Human	3068502
fructose-bisphosphatase	3.1.3.11	Human	3291467
fructose-bisphosphatase	3.1.3.11	Human	4342496
fructose-bisphosphatase	3.1.3.11	Human	7532742
fructose-bisphosphatase	3.1.3.11	Human	7552262
fructose-bisphosphatase	3.1.3.11	Human	7558035
fructose-bisphosphatase	3.1.3.11	Human	7579072
fructose-bisphosphatase	3.1.3.11	Human	7592860
fructose-bisphosphatase	3.1.3.11	Human	8043646
acid phosphatase	3.1.3.2	Human	10471332
acid phosphatase	3.1.3.2	Human	10646119
acid phosphatase	3.1.3.2	Human	1364364
acid phosphatase	3.1.3.2	Human	2153810
acid phosphatase	3.1.3.2	Human	2545859
acid phosphatase	3.1.3.2	Human	2713876

acid phosphatase	3.1.3.2	Human	9370316
inositol-phosphate phosphatase	3.1.3.25	Human	12479670
inositol-phosphate phosphatase	3.1.3.25	Human	15809430
inositol-phosphate phosphatase	3.1.3.25	Human	9462881
phosphoserine phosphatase	3.1.3.3	Human	8858931
phosphatidate phosphatase	3.1.3.4	Human	10695929
phosphatidate phosphatase	3.1.3.4	Human	11678440
phosphatidate phosphatase	3.1.3.4	Human	12359092
phosphatidate phosphatase	3.1.3.4	Human	15527069
phosphatidate phosphatase	3.1.3.4	Human	15539300
phosphatidate phosphatase	3.1.3.4	Human	15975496
phosphatidate phosphatase	3.1.3.4	Human	16684527
phosphatidate phosphatase	3.1.3.4	Human	16968695
phosphatidate phosphatase	3.1.3.4	Human	2153810
phosphatidate phosphatase	3.1.3.4	Human	2174075
phosphatidate phosphatase	3.1.3.4	Human	2674663
phosphatidate phosphatase	3.1.3.4	Human	2713876
phosphatidate phosphatase	3.1.3.4	Human	6277612
phosphatidate phosphatase	3.1.3.4	Human	716971
phosphatidate phosphatase	3.1.3.4	Human	7273126
phosphatidate phosphatase	3.1.3.4	Human	7305894
phosphatidate phosphatase	3.1.3.4	Human	7407220
phosphatidate phosphatase	3.1.3.4	Human	7748273
phosphatidate phosphatase	3.1.3.4	Human	8406356
phosphatidate phosphatase	3.1.3.4	Human	8784738
5'-nucleotidase	3.1.3.5	Human	10506947
5'-nucleotidase	3.1.3.5	Human	10617137
5'-nucleotidase	3.1.3.5	Human	10766785
5'-nucleotidase	3.1.3.5	Human	10869532
5'-nucleotidase	3.1.3.5	Human	11408527
5'-nucleotidase	3.1.3.5	Human	12030367
5'-nucleotidase	3.1.3.5	Human	12061138
5'-nucleotidase	3.1.3.5	Human	12204768
5'-nucleotidase	3.1.3.5	Human	12493585
5'-nucleotidase	3.1.3.5	Human	12571440
5'-nucleotidase	3.1.3.5	Human	12667292
5'-nucleotidase	3.1.3.5	Human	15748706
5'-nucleotidase	3.1.3.5	Human	15946667
5'-nucleotidase	3.1.3.5	Human	6326848
5'-nucleotidase	3.1.3.5	Human	7999131
5'-nucleotidase	3.1.3.5	Human	9009712
5'-nucleotidase	3.1.3.5	Human	9315889
5'-nucleotidase	3.1.3.5	Human	9806332

glucose-6-phosphatase	3.1.3.9	Human	10625614
glucose-6-phosphatase	3.1.3.9	Human	12189168
glucose-6-phosphatase	3.1.3.9	Human	12373573
glucose-6-phosphatase	3.1.3.9	Human	12507516
glucose-6-phosphatase	3.1.3.9	Human	1323600
glucose-6-phosphatase	3.1.3.9	Human	15702236
glucose-6-phosphatase	3.1.3.9	Human	16012821
glucose-6-phosphatase	3.1.3.9	Human	16330542
glucose-6-phosphatase	3.1.3.9	Human	17075770
glucose-6-phosphatase	3.1.3.9	Human	3038860
glucose-6-phosphatase	3.1.3.9	Human	3629725
glucose-6-phosphatase	3.1.3.9	Human	6295653
glucose-6-phosphatase	3.1.3.9	Human	6317150
glucose-6-phosphatase	3.1.3.9	Human	6591771
glucose-6-phosphatase	3.1.3.9	Human	8182131
glucose-6-phosphatase	3.1.3.9	Human	8211187
glucose-6-phosphatase	3.1.3.9	Human	8407995
glucose-6-phosphatase	3.1.3.9	Human	8640227
glucose-6-phosphatase	3.1.3.9	Human	9369482
glucose-6-phosphatase	3.1.3.9	Human	9497333
glucose-6-phosphatase	3.1.3.9	Human	9506766
glucose-6-phosphatase	3.1.3.9	Human	9839806
arylsulfatase	3.1.6.1	Human	6233132
cerebroside-sulfatase	3.1.6.8	Human	6233132
alpha-glucosidase	3.2.1.20	Human	10619707
alpha-glucosidase	3.2.1.20	Human	10810293
alpha-glucosidase	3.2.1.20	Human	11134937
alpha-glucosidase	3.2.1.20	Human	11230125
alpha-glucosidase	3.2.1.20	Human	11404235
alpha-glucosidase	3.2.1.20	Human	16233203
alpha-glucosidase	3.2.1.20	Human	16233375
alpha-glucosidase	3.2.1.20	Human	7626594
alpha-glucosidase	3.2.1.20	Human	8625892
beta-glucosidase	3.2.1.21	Human	10824094
beta-glucosidase	3.2.1.21	Human	15966329
beta-glucosidase	3.2.1.21	Human	6773958
alpha-mannosidase	3.2.1.24	Human	11406577
alpha-mannosidase	3.2.1.24	Human	16460512
alpha-mannosidase	3.2.1.24	Human	16806128
alpha-mannosidase	3.2.1.24	Human	16899540
alpha-mannosidase	3.2.1.24	Human	2466460
alpha-mannosidase	3.2.1.24	Human	2843530
alpha-mannosidase	3.2.1.24	Human	4092864

alpha-mannosidase	3.2.1.24	Human	9022667
alpha-mannosidase	3.2.1.24	Human	9230311
leukotriene-A4 hydrolase	3.3.2.6	Human	10691697
leukotriene-A4 hydrolase	3.3.2.6	Human	11805219
leukotriene-A4 hydrolase	3.3.2.6	Human	12139459
leukotriene-A4 hydrolase	3.3.2.6	Human	12865451
leukotriene-A4 hydrolase	3.3.2.6	Human	1311589
leukotriene-A4 hydrolase	3.3.2.6	Human	6329309
leukotriene-A4 hydrolase	3.3.2.6	Human	7732829
leukotriene-A4 hydrolase	3.3.2.6	Human	9395533
leukotriene-A4 hydrolase	3.3.2.6	Human	9413890
membrane alanyl aminopeptidase	3.4.11.2	Human	11751433
membrane alanyl aminopeptidase	3.4.11.2	Human	12075625
membrane alanyl aminopeptidase	3.4.11.2	Human	16019130
dihydroorotase	3.5.2.3	Human	3345746
dihydroorotase	3.5.2.3	Human	4092695
dihydroorotase	3.5.2.3	Human	42399
dihydroorotase	3.5.2.3	Human	6115855
GTP cyclohydrolase I	3.5.4.16	Human	12392559
GTP cyclohydrolase I	3.5.4.16	Human	12855421
GTP cyclohydrolase I	3.5.4.16	Human	1459137
GTP cyclohydrolase I	3.5.4.16	Human	15044686
GTP cyclohydrolase I	3.5.4.16	Human	16179591
GTP cyclohydrolase I	3.5.4.16	Human	16636057
GTP cyclohydrolase I	3.5.4.16	Human	737222
GTP cyclohydrolase I	3.5.4.16	Human	7521513
GTP cyclohydrolase I	3.5.4.16	Human	9182249
GTP cyclohydrolase I	3.5.4.16	Human	9444617
adenosinetriphosphatase	3.6.1.3	Human	126449
adenosinetriphosphatase	3.6.1.3	Human	160792
adenosinetriphosphatase	3.6.1.3	Human	1727783
adenosinetriphosphatase	3.6.1.3	Human	3772811
adenosinetriphosphatase	3.6.1.3	Human	8667177
H+-transporting two-sector ATPase	3.6.3.14	Human	11744700
H+-transporting two-sector ATPase	3.6.3.14	Human	11893513
H+-transporting two-sector ATPase	3.6.3.14	Human	12587531
H+-transporting two-sector ATPase	3.6.3.14	Human	15712234
H+-transporting two-sector ATPase	3.6.3.14	Human	16510118
H+-transporting two-sector ATPase	3.6.3.14	Human	16730639
H+-transporting two-sector ATPase	3.6.3.14	Human	2532597
H+-transporting two-sector ATPase	3.6.3.14	Human	2889730
H+-transporting two-sector ATPase	3.6.3.14	Human	6456904
H+-transporting two-sector ATPase	3.6.3.14	Human	8516333

fumarylacetoacetase	3.7.1.2	Human	9734339
glutamate decarboxylase	4.1.1.15	Human	10331265
glutamate decarboxylase	4.1.1.15	Human	12196588
glutamate decarboxylase	4.1.1.15	Human	12746320
glutamate decarboxylase	4.1.1.15	Human	15210535
glutamate decarboxylase	4.1.1.15	Human	15581395
glutamate decarboxylase	4.1.1.15	Human	1697032
glutamate decarboxylase	4.1.1.15	Human	17044036
glutamate decarboxylase	4.1.1.15	Human	1976015
glutamate decarboxylase	4.1.1.15	Human	2180326
glutamate decarboxylase	4.1.1.15	Human	2735448
glutamate decarboxylase	4.1.1.15	Human	2857768
glutamate decarboxylase	4.1.1.15	Human	3896834
glutamate decarboxylase	4.1.1.15	Human	6975381
glutamate decarboxylase	4.1.1.15	Human	7702443
glutamate decarboxylase	4.1.1.15	Human	7885536
glutamate decarboxylase	4.1.1.15	Human	8302162
glutamate decarboxylase	4.1.1.15	Human	9011754
glutamate decarboxylase	4.1.1.15	Human	9053794
ornithine decarboxylase	4.1.1.17	Human	10069996
ornithine decarboxylase	4.1.1.17	Human	10216947
ornithine decarboxylase	4.1.1.17	Human	10319188
ornithine decarboxylase	4.1.1.17	Human	10320037
ornithine decarboxylase	4.1.1.17	Human	10321508
ornithine decarboxylase	4.1.1.17	Human	10430664
ornithine decarboxylase	4.1.1.17	Human	10453061
ornithine decarboxylase	4.1.1.17	Human	10456943
ornithine decarboxylase	4.1.1.17	Human	10473083
ornithine decarboxylase	4.1.1.17	Human	10485326
ornithine decarboxylase	4.1.1.17	Human	10544213
ornithine decarboxylase	4.1.1.17	Human	10550568
ornithine decarboxylase	4.1.1.17	Human	10564512
ornithine decarboxylase	4.1.1.17	Human	10589756
ornithine decarboxylase	4.1.1.17	Human	10593613
ornithine decarboxylase	4.1.1.17	Human	10607762
ornithine decarboxylase	4.1.1.17	Human	10629084
ornithine decarboxylase	4.1.1.17	Human	10712236
ornithine decarboxylase	4.1.1.17	Human	10713131
ornithine decarboxylase	4.1.1.17	Human	10760944
ornithine decarboxylase	4.1.1.17	Human	10772389
ornithine decarboxylase	4.1.1.17	Human	10816435
ornithine decarboxylase	4.1.1.17	Human	10817834
ornithine decarboxylase	4.1.1.17	Human	10882097

ornithine decarboxylase	4.1.1.17	Human	10931831
ornithine decarboxylase	4.1.1.17	Human	10940513
ornithine decarboxylase	4.1.1.17	Human	10965017
ornithine decarboxylase	4.1.1.17	Human	11003584
ornithine decarboxylase	4.1.1.17	Human	11085920
ornithine decarboxylase	4.1.1.17	Human	11095648
ornithine decarboxylase	4.1.1.17	Human	11137705
ornithine decarboxylase	4.1.1.17	Human	11180396
ornithine decarboxylase	4.1.1.17	Human	11235918
ornithine decarboxylase	4.1.1.17	Human	11355005
ornithine decarboxylase	4.1.1.17	Human	11376395
ornithine decarboxylase	4.1.1.17	Human	11408092
ornithine decarboxylase	4.1.1.17	Human	11408253
ornithine decarboxylase	4.1.1.17	Human	11408542
ornithine decarboxylase	4.1.1.17	Human	11413269
ornithine decarboxylase	4.1.1.17	Human	11540835
ornithine decarboxylase	4.1.1.17	Human	11558274
ornithine decarboxylase	4.1.1.17	Human	11736657
ornithine decarboxylase	4.1.1.17	Human	11782361
ornithine decarboxylase	4.1.1.17	Human	11852055
ornithine decarboxylase	4.1.1.17	Human	11883715
ornithine decarboxylase	4.1.1.17	Human	11922393
ornithine decarboxylase	4.1.1.17	Human	11923270
ornithine decarboxylase	4.1.1.17	Human	11964084
ornithine decarboxylase	4.1.1.17	Human	11997243
ornithine decarboxylase	4.1.1.17	Human	12054570
ornithine decarboxylase	4.1.1.17	Human	12105848
ornithine decarboxylase	4.1.1.17	Human	12148577
ornithine decarboxylase	4.1.1.17	Human	12355213
ornithine decarboxylase	4.1.1.17	Human	12452334
ornithine decarboxylase	4.1.1.17	Human	12497077
ornithine decarboxylase	4.1.1.17	Human	12527115
ornithine decarboxylase	4.1.1.17	Human	12663506
ornithine decarboxylase	4.1.1.17	Human	12716308
ornithine decarboxylase	4.1.1.17	Human	12716758
ornithine decarboxylase	4.1.1.17	Human	12766050
ornithine decarboxylase	4.1.1.17	Human	12816757
ornithine decarboxylase	4.1.1.17	Human	12856719
ornithine decarboxylase	4.1.1.17	Human	12882169
ornithine decarboxylase	4.1.1.17	Human	1289667
ornithine decarboxylase	4.1.1.17	Human	1324153
ornithine decarboxylase	4.1.1.17	Human	1360468
ornithine decarboxylase	4.1.1.17	Human	1397089

ornithine decarboxylase	4.1.1.17	Human	1407701
ornithine decarboxylase	4.1.1.17	Human	1409247
ornithine decarboxylase	4.1.1.17	Human	1417733
ornithine decarboxylase	4.1.1.17	Human	14769544
ornithine decarboxylase	4.1.1.17	Human	1495349
ornithine decarboxylase	4.1.1.17	Human	15002659
ornithine decarboxylase	4.1.1.17	Human	1511780
ornithine decarboxylase	4.1.1.17	Human	15120115
ornithine decarboxylase	4.1.1.17	Human	15180186
ornithine decarboxylase	4.1.1.17	Human	1521915
ornithine decarboxylase	4.1.1.17	Human	15223770
ornithine decarboxylase	4.1.1.17	Human	15228220
ornithine decarboxylase	4.1.1.17	Human	15233741
ornithine decarboxylase	4.1.1.17	Human	15247138
ornithine decarboxylase	4.1.1.17	Human	15296840
ornithine decarboxylase	4.1.1.17	Human	15306645
ornithine decarboxylase	4.1.1.17	Human	15355308
ornithine decarboxylase	4.1.1.17	Human	15514084
ornithine decarboxylase	4.1.1.17	Human	15538383
ornithine decarboxylase	4.1.1.17	Human	15539331
ornithine decarboxylase	4.1.1.17	Human	1563337
ornithine decarboxylase	4.1.1.17	Human	15697240
ornithine decarboxylase	4.1.1.17	Human	1569947
ornithine decarboxylase	4.1.1.17	Human	15716048
ornithine decarboxylase	4.1.1.17	Human	15843384
ornithine decarboxylase	4.1.1.17	Human	1584960
ornithine decarboxylase	4.1.1.17	Human	1590311
ornithine decarboxylase	4.1.1.17	Human	1601800
ornithine decarboxylase	4.1.1.17	Human	16091008
ornithine decarboxylase	4.1.1.17	Human	16168128
ornithine decarboxylase	4.1.1.17	Human	16170669
ornithine decarboxylase	4.1.1.17	Human	16181115
ornithine decarboxylase	4.1.1.17	Human	16223706
ornithine decarboxylase	4.1.1.17	Human	16230862
ornithine decarboxylase	4.1.1.17	Human	16290266
ornithine decarboxylase	4.1.1.17	Human	16342411
ornithine decarboxylase	4.1.1.17	Human	1641775
ornithine decarboxylase	4.1.1.17	Human	16445292
ornithine decarboxylase	4.1.1.17	Human	1655898
ornithine decarboxylase	4.1.1.17	Human	16568078
ornithine decarboxylase	4.1.1.17	Human	16630547
ornithine decarboxylase	4.1.1.17	Human	16662219
ornithine decarboxylase	4.1.1.17	Human	16666570

ornithine decarboxylase	4.1.1.17	Human	16678846
ornithine decarboxylase	4.1.1.17	Human	16729674
ornithine decarboxylase	4.1.1.17	Human	1697882
ornithine decarboxylase	4.1.1.17	Human	1703390
ornithine decarboxylase	4.1.1.17	Human	1733364
ornithine decarboxylase	4.1.1.17	Human	17407445
ornithine decarboxylase	4.1.1.17	Human	1745018
ornithine decarboxylase	4.1.1.17	Human	1782416
ornithine decarboxylase	4.1.1.17	Human	1814556
ornithine decarboxylase	4.1.1.17	Human	1814755
ornithine decarboxylase	4.1.1.17	Human	1831810
ornithine decarboxylase	4.1.1.17	Human	1846091
ornithine decarboxylase	4.1.1.17	Human	1878921
ornithine decarboxylase	4.1.1.17	Human	1884248
ornithine decarboxylase	4.1.1.17	Human	1892753
ornithine decarboxylase	4.1.1.17	Human	1900385
ornithine decarboxylase	4.1.1.17	Human	1932775
ornithine decarboxylase	4.1.1.17	Human	1940203
ornithine decarboxylase	4.1.1.17	Human	1962522
ornithine decarboxylase	4.1.1.17	Human	196870
ornithine decarboxylase	4.1.1.17	Human	1997184
ornithine decarboxylase	4.1.1.17	Human	2006469
ornithine decarboxylase	4.1.1.17	Human	2009332
ornithine decarboxylase	4.1.1.17	Human	2019760
ornithine decarboxylase	4.1.1.17	Human	203259
ornithine decarboxylase	4.1.1.17	Human	2051775
ornithine decarboxylase	4.1.1.17	Human	2088816
ornithine decarboxylase	4.1.1.17	Human	2118148
ornithine decarboxylase	4.1.1.17	Human	2148056
ornithine decarboxylase	4.1.1.17	Human	2160044
ornithine decarboxylase	4.1.1.17	Human	2197525
ornithine decarboxylase	4.1.1.17	Human	2210666
ornithine decarboxylase	4.1.1.17	Human	2243540
ornithine decarboxylase	4.1.1.17	Human	2293084
ornithine decarboxylase	4.1.1.17	Human	2296762
ornithine decarboxylase	4.1.1.17	Human	2298913
ornithine decarboxylase	4.1.1.17	Human	2409817
ornithine decarboxylase	4.1.1.17	Human	2469492
ornithine decarboxylase	4.1.1.17	Human	2472814
ornithine decarboxylase	4.1.1.17	Human	2493794
ornithine decarboxylase	4.1.1.17	Human	2494779
ornithine decarboxylase	4.1.1.17	Human	2497460
ornithine decarboxylase	4.1.1.17	Human	2497556

ornithine decarboxylase	4.1.1.17	Human	2505399
ornithine decarboxylase	4.1.1.17	Human	2505959
ornithine decarboxylase	4.1.1.17	Human	2507383
ornithine decarboxylase	4.1.1.17	Human	2507471
ornithine decarboxylase	4.1.1.17	Human	2525760
ornithine decarboxylase	4.1.1.17	Human	2553150
ornithine decarboxylase	4.1.1.17	Human	2610929
ornithine decarboxylase	4.1.1.17	Human	2651129
ornithine decarboxylase	4.1.1.17	Human	2699646
ornithine decarboxylase	4.1.1.17	Human	2754510
ornithine decarboxylase	4.1.1.17	Human	2775206
ornithine decarboxylase	4.1.1.17	Human	2829727
ornithine decarboxylase	4.1.1.17	Human	2840461
ornithine decarboxylase	4.1.1.17	Human	2915649
ornithine decarboxylase	4.1.1.17	Human	2916900
ornithine decarboxylase	4.1.1.17	Human	2977154
ornithine decarboxylase	4.1.1.17	Human	2979197
ornithine decarboxylase	4.1.1.17	Human	2986953
ornithine decarboxylase	4.1.1.17	Human	3004707
ornithine decarboxylase	4.1.1.17	Human	3009424
ornithine decarboxylase	4.1.1.17	Human	3022056
ornithine decarboxylase	4.1.1.17	Human	3036091
ornithine decarboxylase	4.1.1.17	Human	3037250
ornithine decarboxylase	4.1.1.17	Human	3040821
ornithine decarboxylase	4.1.1.17	Human	3082276
ornithine decarboxylase	4.1.1.17	Human	3084209
ornithine decarboxylase	4.1.1.17	Human	3084872
ornithine decarboxylase	4.1.1.17	Human	3086160
ornithine decarboxylase	4.1.1.17	Human	3092827
ornithine decarboxylase	4.1.1.17	Human	3093095
ornithine decarboxylase	4.1.1.17	Human	3096557
ornithine decarboxylase	4.1.1.17	Human	3100897
ornithine decarboxylase	4.1.1.17	Human	3102397
ornithine decarboxylase	4.1.1.17	Human	3105968
ornithine decarboxylase	4.1.1.17	Human	3106075
ornithine decarboxylase	4.1.1.17	Human	3108666
ornithine decarboxylase	4.1.1.17	Human	3109979
ornithine decarboxylase	4.1.1.17	Human	3109985
ornithine decarboxylase	4.1.1.17	Human	3111384
ornithine decarboxylase	4.1.1.17	Human	3113732
ornithine decarboxylase	4.1.1.17	Human	3117720
ornithine decarboxylase	4.1.1.17	Human	3121457
ornithine decarboxylase	4.1.1.17	Human	3122042

ornithine decarboxylase	4.1.1.17	Human	3129184
ornithine decarboxylase	4.1.1.17	Human	3130188
ornithine decarboxylase	4.1.1.17	Human	3139441
ornithine decarboxylase	4.1.1.17	Human	3141045
ornithine decarboxylase	4.1.1.17	Human	3143046
ornithine decarboxylase	4.1.1.17	Human	3180091
ornithine decarboxylase	4.1.1.17	Human	3279036
ornithine decarboxylase	4.1.1.17	Human	3328430
ornithine decarboxylase	4.1.1.17	Human	3356404
ornithine decarboxylase	4.1.1.17	Human	3403538
ornithine decarboxylase	4.1.1.17	Human	3443298
ornithine decarboxylase	4.1.1.17	Human	3538740
ornithine decarboxylase	4.1.1.17	Human	3548994
ornithine decarboxylase	4.1.1.17	Human	3661847
ornithine decarboxylase	4.1.1.17	Human	3672608
ornithine decarboxylase	4.1.1.17	Human	3688216
ornithine decarboxylase	4.1.1.17	Human	3729588
ornithine decarboxylase	4.1.1.17	Human	3743773
ornithine decarboxylase	4.1.1.17	Human	3753036
ornithine decarboxylase	4.1.1.17	Human	3775249
ornithine decarboxylase	4.1.1.17	Human	3794781
ornithine decarboxylase	4.1.1.17	Human	3857388
ornithine decarboxylase	4.1.1.17	Human	3901680
ornithine decarboxylase	4.1.1.17	Human	3905315
ornithine decarboxylase	4.1.1.17	Human	3921243
ornithine decarboxylase	4.1.1.17	Human	3926303
ornithine decarboxylase	4.1.1.17	Human	3930649
ornithine decarboxylase	4.1.1.17	Human	3931300
ornithine decarboxylase	4.1.1.17	Human	3934106
ornithine decarboxylase	4.1.1.17	Human	3999751
ornithine decarboxylase	4.1.1.17	Human	4029343
ornithine decarboxylase	4.1.1.17	Human	4053280
ornithine decarboxylase	4.1.1.17	Human	497279
ornithine decarboxylase	4.1.1.17	Human	6124275
ornithine decarboxylase	4.1.1.17	Human	6178351
ornithine decarboxylase	4.1.1.17	Human	6190690
ornithine decarboxylase	4.1.1.17	Human	6192925
ornithine decarboxylase	4.1.1.17	Human	6223191
ornithine decarboxylase	4.1.1.17	Human	6256169
ornithine decarboxylase	4.1.1.17	Human	6272311
ornithine decarboxylase	4.1.1.17	Human	6307502
ornithine decarboxylase	4.1.1.17	Human	6365078
ornithine decarboxylase	4.1.1.17	Human	6432312

ornithine decarboxylase	4.1.1.17	Human	6432848
ornithine decarboxylase	4.1.1.17	Human	6439208
ornithine decarboxylase	4.1.1.17	Human	6440787
ornithine decarboxylase	4.1.1.17	Human	6445842
ornithine decarboxylase	4.1.1.17	Human	6467454
ornithine decarboxylase	4.1.1.17	Human	6571411
ornithine decarboxylase	4.1.1.17	Human	6591862
ornithine decarboxylase	4.1.1.17	Human	6624798
ornithine decarboxylase	4.1.1.17	Human	6692409
ornithine decarboxylase	4.1.1.17	Human	6696980
ornithine decarboxylase	4.1.1.17	Human	6721578
ornithine decarboxylase	4.1.1.17	Human	6750139
ornithine decarboxylase	4.1.1.17	Human	6812570
ornithine decarboxylase	4.1.1.17	Human	6813460
ornithine decarboxylase	4.1.1.17	Human	6865777
ornithine decarboxylase	4.1.1.17	Human	6891933
ornithine decarboxylase	4.1.1.17	Human	7093948
ornithine decarboxylase	4.1.1.17	Human	7104206
ornithine decarboxylase	4.1.1.17	Human	7159401
ornithine decarboxylase	4.1.1.17	Human	7205597
ornithine decarboxylase	4.1.1.17	Human	7310281
ornithine decarboxylase	4.1.1.17	Human	7381752
ornithine decarboxylase	4.1.1.17	Human	7426404
ornithine decarboxylase	4.1.1.17	Human	7488168
ornithine decarboxylase	4.1.1.17	Human	7498733
ornithine decarboxylase	4.1.1.17	Human	7525612
ornithine decarboxylase	4.1.1.17	Human	7616440
ornithine decarboxylase	4.1.1.17	Human	7628376
ornithine decarboxylase	4.1.1.17	Human	7656288
ornithine decarboxylase	4.1.1.17	Human	7718766
ornithine decarboxylase	4.1.1.17	Human	7813017
ornithine decarboxylase	4.1.1.17	Human	7823874
ornithine decarboxylase	4.1.1.17	Human	7865470
ornithine decarboxylase	4.1.1.17	Human	7872745
ornithine decarboxylase	4.1.1.17	Human	7874572
ornithine decarboxylase	4.1.1.17	Human	7895420
ornithine decarboxylase	4.1.1.17	Human	7929646
ornithine decarboxylase	4.1.1.17	Human	7943199
ornithine decarboxylase	4.1.1.17	Human	7951165
ornithine decarboxylase	4.1.1.17	Human	7965748
ornithine decarboxylase	4.1.1.17	Human	7972938
ornithine decarboxylase	4.1.1.17	Human	7981636
ornithine decarboxylase	4.1.1.17	Human	8010156

ornithine decarboxylase	4.1.1.17	Human	8028020
ornithine decarboxylase	4.1.1.17	Human	8065308
ornithine decarboxylase	4.1.1.17	Human	8095973
ornithine decarboxylase	4.1.1.17	Human	8110472
ornithine decarboxylase	4.1.1.17	Human	8140036
ornithine decarboxylase	4.1.1.17	Human	8141779
ornithine decarboxylase	4.1.1.17	Human	8152342
ornithine decarboxylase	4.1.1.17	Human	8185631
ornithine decarboxylase	4.1.1.17	Human	8190721
ornithine decarboxylase	4.1.1.17	Human	8242794
ornithine decarboxylase	4.1.1.17	Human	8344985
ornithine decarboxylase	4.1.1.17	Human	8368314
ornithine decarboxylase	4.1.1.17	Human	8374143
ornithine decarboxylase	4.1.1.17	Human	8419528
ornithine decarboxylase	4.1.1.17	Human	8447420
ornithine decarboxylase	4.1.1.17	Human	8453677
ornithine decarboxylase	4.1.1.17	Human	8462726
ornithine decarboxylase	4.1.1.17	Human	8465553
ornithine decarboxylase	4.1.1.17	Human	8478959
ornithine decarboxylase	4.1.1.17	Human	8501729
ornithine decarboxylase	4.1.1.17	Human	8538189
ornithine decarboxylase	4.1.1.17	Human	8549635
ornithine decarboxylase	4.1.1.17	Human	8572176
ornithine decarboxylase	4.1.1.17	Human	8660289
ornithine decarboxylase	4.1.1.17	Human	8693031
ornithine decarboxylase	4.1.1.17	Human	8707896
ornithine decarboxylase	4.1.1.17	Human	8727257
ornithine decarboxylase	4.1.1.17	Human	8760120
ornithine decarboxylase	4.1.1.17	Human	8760129
ornithine decarboxylase	4.1.1.17	Human	8768305
ornithine decarboxylase	4.1.1.17	Human	8777294
ornithine decarboxylase	4.1.1.17	Human	8814137
ornithine decarboxylase	4.1.1.17	Human	8848835
ornithine decarboxylase	4.1.1.17	Human	8858522
ornithine decarboxylase	4.1.1.17	Human	8878500
ornithine decarboxylase	4.1.1.17	Human	8882155
ornithine decarboxylase	4.1.1.17	Human	8912847
ornithine decarboxylase	4.1.1.17	Human	8944705
ornithine decarboxylase	4.1.1.17	Human	9009157
ornithine decarboxylase	4.1.1.17	Human	9016399
ornithine decarboxylase	4.1.1.17	Human	9017896
ornithine decarboxylase	4.1.1.17	Human	9022291
ornithine decarboxylase	4.1.1.17	Human	9024941

ornithine decarboxylase	4.1.1.17	Human	9063811
ornithine decarboxylase	4.1.1.17	Human	9073141
ornithine decarboxylase	4.1.1.17	Human	9134011
ornithine decarboxylase	4.1.1.17	Human	9142900
ornithine decarboxylase	4.1.1.17	Human	9191978
ornithine decarboxylase	4.1.1.17	Human	9213218
ornithine decarboxylase	4.1.1.17	Human	9223345
ornithine decarboxylase	4.1.1.17	Human	9224728
ornithine decarboxylase	4.1.1.17	Human	9251105
ornithine decarboxylase	4.1.1.17	Human	9252524
ornithine decarboxylase	4.1.1.17	Human	9294258
ornithine decarboxylase	4.1.1.17	Human	9368191
ornithine decarboxylase	4.1.1.17	Human	9371082
ornithine decarboxylase	4.1.1.17	Human	9454972
ornithine decarboxylase	4.1.1.17	Human	9458728
ornithine decarboxylase	4.1.1.17	Human	9468098
ornithine decarboxylase	4.1.1.17	Human	9495243
ornithine decarboxylase	4.1.1.17	Human	9525811
ornithine decarboxylase	4.1.1.17	Human	9590135
ornithine decarboxylase	4.1.1.17	Human	9609384
ornithine decarboxylase	4.1.1.17	Human	9612269
ornithine decarboxylase	4.1.1.17	Human	9624108
ornithine decarboxylase	4.1.1.17	Human	9648877
ornithine decarboxylase	4.1.1.17	Human	9685330
ornithine decarboxylase	4.1.1.17	Human	9688665
ornithine decarboxylase	4.1.1.17	Human	9769382
ornithine decarboxylase	4.1.1.17	Human	9772292
ornithine decarboxylase	4.1.1.17	Human	9795249
ornithine decarboxylase	4.1.1.17	Human	9806166
ornithine decarboxylase	4.1.1.17	Human	9829706
ornithine decarboxylase	4.1.1.17	Human	9868187
ornithine decarboxylase	4.1.1.17	Human	9884080
ornithine decarboxylase	4.1.1.17	Human	9890191
arginine decarboxylase	4.1.1.19	Human	10420650
arginine decarboxylase	4.1.1.19	Human	11029703
arginine decarboxylase	4.1.1.19	Human	11540835
arginine decarboxylase	4.1.1.19	Human	11576438
arginine decarboxylase	4.1.1.19	Human	11903975
arginine decarboxylase	4.1.1.19	Human	12060267
arginine decarboxylase	4.1.1.19	Human	15032880
arginine decarboxylase	4.1.1.19	Human	15092366
arginine decarboxylase	4.1.1.19	Human	15120115
arginine decarboxylase	4.1.1.19	Human	15527979

arginine decarboxylase	4.1.1.19	Human	15733873
arginine decarboxylase	4.1.1.19	Human	16662219
arginine decarboxylase	4.1.1.19	Human	16663805
arginine decarboxylase	4.1.1.19	Human	16769152
arginine decarboxylase	4.1.1.19	Human	3931079
arginine decarboxylase	4.1.1.19	Human	9230111
arginine decarboxylase	4.1.1.19	Human	9624108
histidine decarboxylase	4.1.1.22	Human	10652359
histidine decarboxylase	4.1.1.22	Human	10843737
histidine decarboxylase	4.1.1.22	Human	11566179
histidine decarboxylase	4.1.1.22	Human	11827977
histidine decarboxylase	4.1.1.22	Human	12875237
histidine decarboxylase	4.1.1.22	Human	12960041
histidine decarboxylase	4.1.1.22	Human	15114681
histidine decarboxylase	4.1.1.22	Human	16203768
histidine decarboxylase	4.1.1.22	Human	16564918
histidine decarboxylase	4.1.1.22	Human	2889701
histidine decarboxylase	4.1.1.22	Human	3587507
histidine decarboxylase	4.1.1.22	Human	7742310
histidine decarboxylase	4.1.1.22	Human	9041686
aromatic-L-amino-acid decarboxylase	4.1.1.28	Human	10080715
aromatic-L-amino-acid decarboxylase	4.1.1.28	Human	10608659
aromatic-L-amino-acid decarboxylase	4.1.1.28	Human	12235825
aromatic-L-amino-acid decarboxylase	4.1.1.28	Human	15684695
aromatic-L-amino-acid decarboxylase	4.1.1.28	Human	15900211
aromatic-L-amino-acid decarboxylase	4.1.1.28	Human	15927700
aromatic-L-amino-acid decarboxylase	4.1.1.28	Human	16164646
aromatic-L-amino-acid decarboxylase	4.1.1.28	Human	17017570
aromatic-L-amino-acid decarboxylase	4.1.1.28	Human	1729407
aromatic-L-amino-acid decarboxylase	4.1.1.28	Human	6968497
aromatic-L-amino-acid decarboxylase	4.1.1.28	Human	7651438
aromatic-L-amino-acid decarboxylase	4.1.1.28	Human	9625353
sulfinoalanine decarboxylase	4.1.1.29	Human	10993179
phosphoenolpyruvate carboxykinase (GTP)	4.1.1.32	Human	11053047
phosphoenolpyruvate carboxykinase (GTP)	4.1.1.32	Human	11700062
phosphoenolpyruvate carboxykinase (GTP)	4.1.1.32	Human	11728630
phosphoenolpyruvate carboxykinase (GTP)	4.1.1.32	Human	11741859
phosphoenolpyruvate carboxykinase (GTP)	4.1.1.32	Human	11872659
phosphoenolpyruvate carboxykinase (GTP)	4.1.1.32	Human	11959679
phosphoenolpyruvate carboxykinase (GTP)	4.1.1.32	Human	12485530
phosphoenolpyruvate carboxykinase (GTP)	4.1.1.32	Human	12925798
phosphoenolpyruvate carboxykinase (GTP)	4.1.1.32	Human	14739078
phosphoenolpyruvate carboxykinase (GTP)	4.1.1.32	Human	15733733

phosphoenolpyruvate carboxykinase (GTP)	4.1.1.32	Human	15983413
phosphoenolpyruvate carboxykinase (GTP)	4.1.1.32	Human	16132948
phosphoenolpyruvate carboxykinase (GTP)	4.1.1.32	Human	16324924
phosphoenolpyruvate carboxykinase (GTP)	4.1.1.32	Human	16330542
phosphoenolpyruvate carboxykinase (GTP)	4.1.1.32	Human	16375695
phosphoenolpyruvate carboxykinase (GTP)	4.1.1.32	Human	16458327
phosphoenolpyruvate carboxykinase (GTP)	4.1.1.32	Human	1701430
phosphoenolpyruvate carboxykinase (GTP)	4.1.1.32	Human	17097062
phosphoenolpyruvate carboxykinase (GTP)	4.1.1.32	Human	1720862
phosphoenolpyruvate carboxykinase (GTP)	4.1.1.32	Human	17446233
phosphoenolpyruvate carboxykinase (GTP)	4.1.1.32	Human	2919162
phosphoenolpyruvate carboxykinase (GTP)	4.1.1.32	Human	3023262
phosphoenolpyruvate carboxykinase (GTP)	4.1.1.32	Human	3068502
phosphoenolpyruvate carboxykinase (GTP)	4.1.1.32	Human	4053567
phosphoenolpyruvate carboxykinase (GTP)	4.1.1.32	Human	6440018
phosphoenolpyruvate carboxykinase (GTP)	4.1.1.32	Human	7854322
phosphoenolpyruvate carboxykinase (GTP)	4.1.1.32	Human	8902188
phosphoenolpyruvate carboxykinase (GTP)	4.1.1.32	Human	9214454
phosphoenolpyruvate carboxykinase (GTP)	4.1.1.32	Human	9242918
phosphoenolpyruvate carboxykinase (GTP)	4.1.1.32	Human	9762796
adenosylmethionine decarboxylase	4.1.1.50	Human	10216947
adenosylmethionine decarboxylase	4.1.1.50	Human	10378277
adenosylmethionine decarboxylase	4.1.1.50	Human	10413038
adenosylmethionine decarboxylase	4.1.1.50	Human	10467042
adenosylmethionine decarboxylase	4.1.1.50	Human	10713131
adenosylmethionine decarboxylase	4.1.1.50	Human	10949915
adenosylmethionine decarboxylase	4.1.1.50	Human	11076965
adenosylmethionine decarboxylase	4.1.1.50	Human	11085920
adenosylmethionine decarboxylase	4.1.1.50	Human	11348531
adenosylmethionine decarboxylase	4.1.1.50	Human	11390378
adenosylmethionine decarboxylase	4.1.1.50	Human	11526206
adenosylmethionine decarboxylase	4.1.1.50	Human	11583148
adenosylmethionine decarboxylase	4.1.1.50	Human	11923270
adenosylmethionine decarboxylase	4.1.1.50	Human	12674502
adenosylmethionine decarboxylase	4.1.1.50	Human	14618239
adenosylmethionine decarboxylase	4.1.1.50	Human	15150268
adenosylmethionine decarboxylase	4.1.1.50	Human	15821146
adenosylmethionine decarboxylase	4.1.1.50	Human	16372273
adenosylmethionine decarboxylase	4.1.1.50	Human	16423999
adenosylmethionine decarboxylase	4.1.1.50	Human	16515461
adenosylmethionine decarboxylase	4.1.1.50	Human	16642382
adenosylmethionine decarboxylase	4.1.1.50	Human	16941339
adenosylmethionine decarboxylase	4.1.1.50	Human	2013278

adenosylmethionine decarboxylase	4.1.1.50	Human	2209170
adenosylmethionine decarboxylase	4.1.1.50	Human	2775206
adenosylmethionine decarboxylase	4.1.1.50	Human	3250232
adenosylmethionine decarboxylase	4.1.1.50	Human	4062886
adenosylmethionine decarboxylase	4.1.1.50	Human	7789170
adenosylmethionine decarboxylase	4.1.1.50	Human	7945201
adenosylmethionine decarboxylase	4.1.1.50	Human	8142949
adenosylmethionine decarboxylase	4.1.1.50	Human	8178573
adenosylmethionine decarboxylase	4.1.1.50	Human	8198469
adenosylmethionine decarboxylase	4.1.1.50	Human	8353934
adenosylmethionine decarboxylase	4.1.1.50	Human	8760129
adenosylmethionine decarboxylase	4.1.1.50	Human	8814137
adenosylmethionine decarboxylase	4.1.1.50	Human	8973561
adenosylmethionine decarboxylase	4.1.1.50	Human	9435790
adenosylmethionine decarboxylase	4.1.1.50	Human	9841864
adenosylmethionine decarboxylase	4.1.1.50	Human	9879883
fructose-bisphosphate aldolase	4.1.2.13	Human	10498814
fructose-bisphosphate aldolase	4.1.2.13	Human	12020659
fructose-bisphosphate aldolase	4.1.2.13	Human	12876349
fructose-bisphosphate aldolase	4.1.2.13	Human	15142555
fructose-bisphosphate aldolase	4.1.2.13	Human	15869466
fructose-bisphosphate aldolase	4.1.2.13	Human	16502329
fructose-bisphosphate aldolase	4.1.2.13	Human	1658253
fructose-bisphosphate aldolase	4.1.2.13	Human	1894606
fructose-bisphosphate aldolase	4.1.2.13	Human	6440018
fructose-bisphosphate aldolase	4.1.2.13	Human	678439
fructose-bisphosphate aldolase	4.1.2.13	Human	7388140
fructose-bisphosphate aldolase	4.1.2.13	Human	7786768
fructose-bisphosphate aldolase	4.1.2.13	Human	8015399
fructose-bisphosphate aldolase	4.1.2.13	Human	8913875
fructose-bisphosphate aldolase	4.1.2.13	Human	9163906
fructose-bisphosphate aldolase	4.1.2.13	Human	9473304
carbonate dehydratase	4.2.1.1	Human	16051345
carbonate dehydratase	4.2.1.1	Human	16310354
carbonate dehydratase	4.2.1.1	Human	1908243
carbonate dehydratase	4.2.1.1	Human	2117006
carbonate dehydratase	4.2.1.1	Human	3128444
carbonate dehydratase	4.2.1.1	Human	6408083
carbonate dehydratase	4.2.1.1	Human	6772280
carbonate dehydratase	4.2.1.1	Human	6776264
carbonate dehydratase	4.2.1.1	Human	6791257
carbonate dehydratase	4.2.1.1	Human	7899732
carbonate dehydratase	4.2.1.1	Human	9186493

carbonate dehydratase	4.2.1.1	Human	9486145
carbonate dehydratase	4.2.1.1	Human	9882455
phosphopyruvate hydratase	4.2.1.11	Human	15459207
phosphopyruvate hydratase	4.2.1.11	Human	17437631
phosphopyruvate hydratase	4.2.1.11	Human	8651685
cystathionine beta-synthase	4.2.1.22	Human	11051561
cystathionine beta-synthase	4.2.1.22	Human	11106665
cystathionine beta-synthase	4.2.1.22	Human	12198128
cystathionine beta-synthase	4.2.1.22	Human	15581575
cystathionine beta-synthase	4.2.1.22	Human	15642325
cystathionine beta-synthase	4.2.1.22	Human	16096271
cystathionine beta-synthase	4.2.1.22	Human	16769053
cystathionine beta-synthase	4.2.1.22	Human	17629356
porphobilinogen synthase	4.2.1.24	Human	10634305
porphobilinogen synthase	4.2.1.24	Human	10787385
porphobilinogen synthase	4.2.1.24	Human	15141099
porphobilinogen synthase	4.2.1.24	Human	15259603
porphobilinogen synthase	4.2.1.24	Human	1526942
porphobilinogen synthase	4.2.1.24	Human	16819823
porphobilinogen synthase	4.2.1.24	Human	1959865
porphobilinogen synthase	4.2.1.24	Human	2050126
porphobilinogen synthase	4.2.1.24	Human	2317819
porphobilinogen synthase	4.2.1.24	Human	3009001
porphobilinogen synthase	4.2.1.24	Human	3755290
porphobilinogen synthase	4.2.1.24	Human	3840094
porphobilinogen synthase	4.2.1.24	Human	3966797
porphobilinogen synthase	4.2.1.24	Human	4265023
porphobilinogen synthase	4.2.1.24	Human	6547609
porphobilinogen synthase	4.2.1.24	Human	6688350
porphobilinogen synthase	4.2.1.24	Human	6873612
porphobilinogen synthase	4.2.1.24	Human	7436670
aconitate hydratase	4.2.1.3	Human	11295257
aconitate hydratase	4.2.1.3	Human	11329290
aconitate hydratase	4.2.1.3	Human	15149735
aconitate hydratase	4.2.1.3	Human	15543948
aconitate hydratase	4.2.1.3	Human	16094633
aconitate hydratase	4.2.1.3	Human	16201454
aconitate hydratase	4.2.1.3	Human	7589784
aconitate hydratase	4.2.1.3	Human	8115279
6-pyruvoyltetrahydropterin synthase	4.2.3.12	Human	11022034
6-pyruvoyltetrahydropterin synthase	4.2.3.12	Human	11744995
6-pyruvoyltetrahydropterin synthase	4.2.3.12	Human	11778454
6-pyruvoyltetrahydropterin synthase	4.2.3.12	Human	7545485

6-pyruvoyltetrahydropterin synthase	4.2.3.12	Human	8307017
6-pyruvoyltetrahydropterin synthase	4.2.3.12	Human	9788822
cystathionine gamma-lyase	4.4.1.1	Human	10960449
cystathionine gamma-lyase	4.4.1.1	Human	15038791
cystathionine gamma-lyase	4.4.1.1	Human	15347670
cystathionine gamma-lyase	4.4.1.1	Human	16786305
cystathionine gamma-lyase	4.4.1.1	Human	16946488
cystathionine gamma-lyase	4.4.1.1	Human	17095121
cystathionine gamma-lyase	4.4.1.1	Human	17525332
cystathionine gamma-lyase	4.4.1.1	Human	8973544
leukotriene-C4 synthase	4.4.1.20	Human	10222453
leukotriene-C4 synthase	4.4.1.20	Human	11964666
leukotriene-C4 synthase	4.4.1.20	Human	15730873
leukotriene-C4 synthase	4.4.1.20	Human	17397868
leukotriene-C4 synthase	4.4.1.20	Human	9431445
UDP-N-acetylglucosamine 2-epimerase	5.1.3.14	Human	10334995
UDP-N-acetylglucosamine 2-epimerase	5.1.3.14	Human	11326336
UDP-N-acetylglucosamine 2-epimerase	5.1.3.14	Human	11956597
UDP-N-acetylglucosamine 2-epimerase	5.1.3.14	Human	12927803
UDP-N-acetylglucosamine 2-epimerase	5.1.3.14	Human	15135418
UDP-N-acetylglucosamine 2-epimerase	5.1.3.14	Human	17565386
UDP-N-acetylglucosamine 2-epimerase	5.1.3.14	Human	3780977
UDP-N-acetylglucosamine 2-epimerase	5.1.3.14	Human	8439453
UDP-N-acetylglucosamine 2-epimerase	5.1.3.14	Human	9305888
UDP-glucose 4-epimerase	5.1.3.2	Human	1149741
maleylacetoacetate isomerase	5.2.1.2	Human	11327815
maleylacetoacetate isomerase	5.2.1.2	Human	9734339
steroid Delta-isomerase	5.3.3.1	Human	239964
phosphoglycerate mutase	5.4.2.1	Human	15181008
phosphoglycerate mutase	5.4.2.1	Human	17204863
phosphoglycerate mutase	5.4.2.1	Human	2830218
phosphoglucomutase	5.4.2.2	Human	11102370
phosphoglucomutase	5.4.2.2	Human	1149741
phosphoglucomutase	5.4.2.2	Human	12026175
phosphoglucomutase	5.4.2.2	Human	12791685
phosphoglucomutase	5.4.2.2	Human	15378030
phosphoglucomutase	5.4.2.2	Human	15996095
phosphoglucomutase	5.4.2.2	Human	16046289
lanosterol synthase	5.4.99.7	Human	11995928
lanosterol synthase	5.4.99.7	Human	15763540
lanosterol synthase	5.4.99.7	Human	17088293
inositol-3-phosphate synthase	5.5.1.4	Human	10713523
inositol-3-phosphate synthase	5.5.1.4	Human	11779862

inositol-3-phosphate synthase	5.5.1.4	Human	12005437
inositol-3-phosphate synthase	5.5.1.4	Human	12941308
inositol-3-phosphate synthase	5.5.1.4	Human	14550537
inositol-3-phosphate synthase	5.5.1.4	Human	14684747
inositol-3-phosphate synthase	5.5.1.4	Human	14730448
inositol-3-phosphate synthase	5.5.1.4	Human	1587797
inositol-3-phosphate synthase	5.5.1.4	Human	15965017
inositol-3-phosphate synthase	5.5.1.4	Human	16453101
inositol-3-phosphate synthase	5.5.1.4	Human	6687929
inositol-3-phosphate synthase	5.5.1.4	Human	9106505
acetate---CoA ligase	6.2.1.1	Human	15236963
acetate---CoA ligase	6.2.1.1	Human	15899897
acetate---CoA ligase	6.2.1.1	Human	2902801
acetate---CoA ligase	6.2.1.1	Human	4149947
acetate---CoA ligase	6.2.1.1	Human	8218953
long-chain-fatty-acid---CoA ligase	6.2.1.3	Human	11375393
long-chain-fatty-acid---CoA ligase	6.2.1.3	Human	1769731
succinate---CoA ligase (ADP-forming)	6.2.1.5	Human	16101500
succinate---CoA ligase (ADP-forming)	6.2.1.5	Human	1986797
succinate---CoA ligase (ADP-forming)	6.2.1.5	Human	3108130
succinate---CoA ligase (ADP-forming)	6.2.1.5	Human	3746465
succinate---CoA ligase (ADP-forming)	6.2.1.5	Human	6109001
succinate---CoA ligase (ADP-forming)	6.2.1.5	Human	7017725
succinate---CoA ligase (ADP-forming)	6.2.1.5	Human	7062046
succinate---CoA ligase (ADP-forming)	6.2.1.5	Human	7783627
glutamate---ammonia ligase	6.3.1.2	Human	10092169
glutamate---ammonia ligase	6.3.1.2	Human	10383611
glutamate---ammonia ligase	6.3.1.2	Human	10440891
glutamate---ammonia ligase	6.3.1.2	Human	10664131
glutamate---ammonia ligase	6.3.1.2	Human	11069692
glutamate---ammonia ligase	6.3.1.2	Human	11413247
glutamate---ammonia ligase	6.3.1.2	Human	11939529
glutamate---ammonia ligase	6.3.1.2	Human	12232192
glutamate---ammonia ligase	6.3.1.2	Human	12517141
glutamate---ammonia ligase	6.3.1.2	Human	12552916
glutamate---ammonia ligase	6.3.1.2	Human	14714472
glutamate---ammonia ligase	6.3.1.2	Human	14723991
glutamate---ammonia ligase	6.3.1.2	Human	15130478
glutamate---ammonia ligase	6.3.1.2	Human	15489445
glutamate---ammonia ligase	6.3.1.2	Human	15581577
glutamate---ammonia ligase	6.3.1.2	Human	1612427
glutamate---ammonia ligase	6.3.1.2	Human	16687472
glutamate---ammonia ligase	6.3.1.2	Human	16829528

glutamate---ammonia ligase	6.3.1.2	Human	16946267
glutamate---ammonia ligase	6.3.1.2	Human	2434618
glutamate---ammonia ligase	6.3.1.2	Human	26663
glutamate---ammonia ligase	6.3.1.2	Human	6118373
glutamate---ammonia ligase	6.3.1.2	Human	9624228
glutamate---cysteine ligase	6.3.2.2	Human	10215022
glutamate---cysteine ligase	6.3.2.2	Human	10218647
glutamate---cysteine ligase	6.3.2.2	Human	10349842
glutamate---cysteine ligase	6.3.2.2	Human	10385608
glutamate---cysteine ligase	6.3.2.2	Human	10385658
glutamate---cysteine ligase	6.3.2.2	Human	10399958
glutamate---cysteine ligase	6.3.2.2	Human	10439045
glutamate---cysteine ligase	6.3.2.2	Human	10441483
glutamate---cysteine ligase	6.3.2.2	Human	10486302
glutamate---cysteine ligase	6.3.2.2	Human	10515588
glutamate---cysteine ligase	6.3.2.2	Human	10518117
glutamate---cysteine ligase	6.3.2.2	Human	10544055
glutamate---cysteine ligase	6.3.2.2	Human	10544272
glutamate---cysteine ligase	6.3.2.2	Human	10590319
glutamate---cysteine ligase	6.3.2.2	Human	10593589
glutamate---cysteine ligase	6.3.2.2	Human	10594104
glutamate---cysteine ligase	6.3.2.2	Human	10600876
glutamate---cysteine ligase	6.3.2.2	Human	10623879
glutamate---cysteine ligase	6.3.2.2	Human	10644053
glutamate---cysteine ligase	6.3.2.2	Human	10674357
glutamate---cysteine ligase	6.3.2.2	Human	10677377
glutamate---cysteine ligase	6.3.2.2	Human	10702364
glutamate---cysteine ligase	6.3.2.2	Human	10719238
glutamate---cysteine ligase	6.3.2.2	Human	10733945
glutamate---cysteine ligase	6.3.2.2	Human	10748080
glutamate---cysteine ligase	6.3.2.2	Human	10773686
glutamate---cysteine ligase	6.3.2.2	Human	10777712
glutamate---cysteine ligase	6.3.2.2	Human	10802223
glutamate---cysteine ligase	6.3.2.2	Human	10928075
glutamate---cysteine ligase	6.3.2.2	Human	10960449
glutamate---cysteine ligase	6.3.2.2	Human	11007940
glutamate---cysteine ligase	6.3.2.2	Human	11025451
glutamate---cysteine ligase	6.3.2.2	Human	11028671
glutamate---cysteine ligase	6.3.2.2	Human	11032771
glutamate---cysteine ligase	6.3.2.2	Human	11097862
glutamate---cysteine ligase	6.3.2.2	Human	11133045
glutamate---cysteine ligase	6.3.2.2	Human	11157875
glutamate---cysteine ligase	6.3.2.2	Human	11163433

glutamate---cysteine ligase	6.3.2.2	Human	11181934
glutamate---cysteine ligase	6.3.2.2	Human	11233143
glutamate---cysteine ligase	6.3.2.2	Human	11306445
glutamate---cysteine ligase	6.3.2.2	Human	11339815
glutamate---cysteine ligase	6.3.2.2	Human	11352989
glutamate---cysteine ligase	6.3.2.2	Human	11353135
glutamate---cysteine ligase	6.3.2.2	Human	11500053
glutamate---cysteine ligase	6.3.2.2	Human	11560771
glutamate---cysteine ligase	6.3.2.2	Human	11565956
glutamate---cysteine ligase	6.3.2.2	Human	11687904
glutamate---cysteine ligase	6.3.2.2	Human	11705692
glutamate---cysteine ligase	6.3.2.2	Human	11780957
glutamate---cysteine ligase	6.3.2.2	Human	11781188
glutamate---cysteine ligase	6.3.2.2	Human	11790356
glutamate---cysteine ligase	6.3.2.2	Human	11812649
glutamate---cysteine ligase	6.3.2.2	Human	11820781
glutamate---cysteine ligase	6.3.2.2	Human	11841806
glutamate---cysteine ligase	6.3.2.2	Human	11849402
glutamate---cysteine ligase	6.3.2.2	Human	11876501
glutamate---cysteine ligase	6.3.2.2	Human	11970852
glutamate---cysteine ligase	6.3.2.2	Human	12079521
glutamate---cysteine ligase	6.3.2.2	Human	12111865
glutamate---cysteine ligase	6.3.2.2	Human	12147223
glutamate---cysteine ligase	6.3.2.2	Human	12196927
glutamate---cysteine ligase	6.3.2.2	Human	12200125
glutamate---cysteine ligase	6.3.2.2	Human	12204877
glutamate---cysteine ligase	6.3.2.2	Human	12433058
glutamate---cysteine ligase	6.3.2.2	Human	12448821
glutamate---cysteine ligase	6.3.2.2	Human	12452384
glutamate---cysteine ligase	6.3.2.2	Human	12535742
glutamate---cysteine ligase	6.3.2.2	Human	12594957
glutamate---cysteine ligase	6.3.2.2	Human	12601050
glutamate---cysteine ligase	6.3.2.2	Human	12607907
glutamate---cysteine ligase	6.3.2.2	Human	12628495
glutamate---cysteine ligase	6.3.2.2	Human	12637989
glutamate---cysteine ligase	6.3.2.2	Human	12814619
glutamate---cysteine ligase	6.3.2.2	Human	12882455
glutamate---cysteine ligase	6.3.2.2	Human	12913252
glutamate---cysteine ligase	6.3.2.2	Human	1350904
glutamate---cysteine ligase	6.3.2.2	Human	1351382
glutamate---cysteine ligase	6.3.2.2	Human	1353765
glutamate---cysteine ligase	6.3.2.2	Human	1355406
glutamate---cysteine ligase	6.3.2.2	Human	1362226

glutamate---cysteine ligase	6.3.2.2	Human	13679058
glutamate---cysteine ligase	6.3.2.2	Human	14500406
glutamate---cysteine ligase	6.3.2.2	Human	14514673
glutamate---cysteine ligase	6.3.2.2	Human	14744626
glutamate---cysteine ligase	6.3.2.2	Human	14962359
glutamate---cysteine ligase	6.3.2.2	Human	15020643
glutamate---cysteine ligase	6.3.2.2	Human	15050748
glutamate---cysteine ligase	6.3.2.2	Human	15257546
glutamate---cysteine ligase	6.3.2.2	Human	15314090
glutamate---cysteine ligase	6.3.2.2	Human	15374419
glutamate---cysteine ligase	6.3.2.2	Human	15451066
glutamate---cysteine ligase	6.3.2.2	Human	15477603
glutamate---cysteine ligase	6.3.2.2	Human	15509664
glutamate---cysteine ligase	6.3.2.2	Human	15878398
glutamate---cysteine ligase	6.3.2.2	Human	15946948
glutamate---cysteine ligase	6.3.2.2	Human	16032782
glutamate---cysteine ligase	6.3.2.2	Human	16081425
glutamate---cysteine ligase	6.3.2.2	Human	16162662
glutamate---cysteine ligase	6.3.2.2	Human	16183645
glutamate---cysteine ligase	6.3.2.2	Human	16549430
glutamate---cysteine ligase	6.3.2.2	Human	16566126
glutamate---cysteine ligase	6.3.2.2	Human	1678010
glutamate---cysteine ligase	6.3.2.2	Human	16781460
glutamate---cysteine ligase	6.3.2.2	Human	16949561
glutamate---cysteine ligase	6.3.2.2	Human	16960387
glutamate---cysteine ligase	6.3.2.2	Human	17144898
glutamate---cysteine ligase	6.3.2.2	Human	1784629
glutamate---cysteine ligase	6.3.2.2	Human	1970723
glutamate---cysteine ligase	6.3.2.2	Human	1997009
glutamate---cysteine ligase	6.3.2.2	Human	2572174
glutamate---cysteine ligase	6.3.2.2	Human	2574245
glutamate---cysteine ligase	6.3.2.2	Human	2879531
glutamate---cysteine ligase	6.3.2.2	Human	2897858
glutamate---cysteine ligase	6.3.2.2	Human	2901982
glutamate---cysteine ligase	6.3.2.2	Human	3621155
glutamate---cysteine ligase	6.3.2.2	Human	6897891
glutamate---cysteine ligase	6.3.2.2	Human	7351635
glutamate---cysteine ligase	6.3.2.2	Human	7503776
glutamate---cysteine ligase	6.3.2.2	Human	7568279
glutamate---cysteine ligase	6.3.2.2	Human	7570642
glutamate---cysteine ligase	6.3.2.2	Human	7585502
glutamate---cysteine ligase	6.3.2.2	Human	7622006
glutamate---cysteine ligase	6.3.2.2	Human	7651354

glutamate---cysteine ligase	6.3.2.2	Human	7768207
glutamate---cysteine ligase	6.3.2.2	Human	7901332
glutamate---cysteine ligase	6.3.2.2	Human	7908245
glutamate---cysteine ligase	6.3.2.2	Human	7910419
glutamate---cysteine ligase	6.3.2.2	Human	7929374
glutamate---cysteine ligase	6.3.2.2	Human	7955076
glutamate---cysteine ligase	6.3.2.2	Human	7969079
glutamate---cysteine ligase	6.3.2.2	Human	8001239
glutamate---cysteine ligase	6.3.2.2	Human	8065332
glutamate---cysteine ligase	6.3.2.2	Human	8101766
glutamate---cysteine ligase	6.3.2.2	Human	8106072
glutamate---cysteine ligase	6.3.2.2	Human	8120650
glutamate---cysteine ligase	6.3.2.2	Human	8538700
glutamate---cysteine ligase	6.3.2.2	Human	8582653
glutamate---cysteine ligase	6.3.2.2	Human	8648118
glutamate---cysteine ligase	6.3.2.2	Human	8661240
glutamate---cysteine ligase	6.3.2.2	Human	8751598
glutamate---cysteine ligase	6.3.2.2	Human	8781554
glutamate---cysteine ligase	6.3.2.2	Human	8792848
glutamate---cysteine ligase	6.3.2.2	Human	8806884
glutamate---cysteine ligase	6.3.2.2	Human	8843715
glutamate---cysteine ligase	6.3.2.2	Human	8917676
glutamate---cysteine ligase	6.3.2.2	Human	8930687
glutamate---cysteine ligase	6.3.2.2	Human	8947504
glutamate---cysteine ligase	6.3.2.2	Human	8973794
glutamate---cysteine ligase	6.3.2.2	Human	8981036
glutamate---cysteine ligase	6.3.2.2	Human	8995480
glutamate---cysteine ligase	6.3.2.2	Human	9029270
glutamate---cysteine ligase	6.3.2.2	Human	9054446
glutamate---cysteine ligase	6.3.2.2	Human	9063478
glutamate---cysteine ligase	6.3.2.2	Human	9093011
glutamate---cysteine ligase	6.3.2.2	Human	9119067
glutamate---cysteine ligase	6.3.2.2	Human	9157984
glutamate---cysteine ligase	6.3.2.2	Human	9163779
glutamate---cysteine ligase	6.3.2.2	Human	9185621
glutamate---cysteine ligase	6.3.2.2	Human	9214623
glutamate---cysteine ligase	6.3.2.2	Human	9259355
glutamate---cysteine ligase	6.3.2.2	Human	9268987
glutamate---cysteine ligase	6.3.2.2	Human	9288403
glutamate---cysteine ligase	6.3.2.2	Human	9311606
glutamate---cysteine ligase	6.3.2.2	Human	9374111
glutamate---cysteine ligase	6.3.2.2	Human	9389600
glutamate---cysteine ligase	6.3.2.2	Human	9393741

glutamate---cysteine ligase	6.3.2.2	Human	9425930
glutamate---cysteine ligase	6.3.2.2	Human	9582278
glutamate---cysteine ligase	6.3.2.2	Human	9614065
glutamate---cysteine ligase	6.3.2.2	Human	9626582
glutamate---cysteine ligase	6.3.2.2	Human	9647756
glutamate---cysteine ligase	6.3.2.2	Human	9679558
glutamate---cysteine ligase	6.3.2.2	Human	9703946
glutamate---cysteine ligase	6.3.2.2	Human	9729439
glutamate---cysteine ligase	6.3.2.2	Human	9750167
glutamate---cysteine ligase	6.3.2.2	Human	9756861
glutamate---cysteine ligase	6.3.2.2	Human	9762423
glutamate---cysteine ligase	6.3.2.2	Human	9875552
glutamate---cysteine ligase	6.3.2.2	Human	9895302
glutathione synthase	6.3.2.3	Human	10964706
glutathione synthase	6.3.2.3	Human	11708780
glutathione synthase	6.3.2.3	Human	9880348
phosphoribosylaminoimidazolesuccinocarboxamide synthase	6.3.2.6	Human	701284
carbamoyl-phosphate synthase (ammonia)	6.3.4.16	Human	10047492
carbamoyl-phosphate synthase (ammonia)	6.3.4.16	Human	11872754
carbamoyl-phosphate synthase (ammonia)	6.3.4.16	Human	189805
carbamoyl-phosphate synthase (ammonia)	6.3.4.16	Human	7459875
carbamoyl-phosphate synthase (ammonia)	6.3.4.16	Human	7587391
carbamoyl-phosphate synthase (ammonia)	6.3.4.16	Human	8752005
CTP synthase	6.3.4.2	Human	12678497
CTP synthase	6.3.4.2	Human	16820675
CTP synthase	6.3.4.2	Human	17463002
CTP synthase	6.3.4.2	Human	17681942
CTP synthase	6.3.4.2	Human	2787169
argininosuccinate synthase	6.3.4.5	Human	10709858
argininosuccinate synthase	6.3.4.5	Human	1122920
argininosuccinate synthase	6.3.4.5	Human	11556547
argininosuccinate synthase	6.3.4.5	Human	12618329
argininosuccinate synthase	6.3.4.5	Human	12672181
argininosuccinate synthase	6.3.4.5	Human	1372742
argininosuccinate synthase	6.3.4.5	Human	14571701
argininosuccinate synthase	6.3.4.5	Human	15588718
argininosuccinate synthase	6.3.4.5	Human	16085056
argininosuccinate synthase	6.3.4.5	Human	16787144
argininosuccinate synthase	6.3.4.5	Human	558104
argininosuccinate synthase	6.3.4.5	Human	845694
argininosuccinate synthase	6.3.4.5	Human	8616812
argininosuccinate synthase	6.3.4.5	Human	8798625

argininosuccinate synthase	6.3.4.5	Human	9096605
argininosuccinate synthase	6.3.4.5	Human	9176259
argininosuccinate synthase	6.3.4.5	Human	9211993
argininosuccinate synthase	6.3.4.5	Human	9252090
carbamoyl-phosphate synthase (glutamine-hydrolysing)	6.3.5.5	Human	10659854
carbamoyl-phosphate synthase (glutamine-hydrolysing)	6.3.5.5	Human	10736367
carbamoyl-phosphate synthase (glutamine-hydrolysing)	6.3.5.5	Human	11441057
carbamoyl-phosphate synthase (glutamine-hydrolysing)	6.3.5.5	Human	11872754
carbamoyl-phosphate synthase (glutamine-hydrolysing)	6.3.5.5	Human	11956684
carbamoyl-phosphate synthase (glutamine-hydrolysing)	6.3.5.5	Human	12678497
carbamoyl-phosphate synthase (glutamine-hydrolysing)	6.3.5.5	Human	15326225
carbamoyl-phosphate synthase (glutamine-hydrolysing)	6.3.5.5	Human	15453495
carbamoyl-phosphate synthase (glutamine-hydrolysing)	6.3.5.5	Human	4018077
carbamoyl-phosphate synthase (glutamine-hydrolysing)	6.3.5.5	Human	6115855
carbamoyl-phosphate synthase (glutamine-hydrolysing)	6.3.5.5	Human	6408083
carbamoyl-phosphate synthase (glutamine-hydrolysing)	6.3.5.5	Human	7053379
carbamoyl-phosphate synthase (glutamine-hydrolysing)	6.3.5.5	Human	7209543
carbamoyl-phosphate synthase (glutamine-hydrolysing)	6.3.5.5	Human	7608487
carbamoyl-phosphate synthase (glutamine-hydrolysing)	6.3.5.5	Human	7916269
pyruvate carboxylase	6.4.1.1	Human	10323732
pyruvate carboxylase	6.4.1.1	Human	16325442
pyruvate carboxylase	6.4.1.1	Human	3182810
pyruvate carboxylase	6.4.1.1	Human	6721853
acetyl-CoA carboxylase	6.4.1.2	Human	10098661
acetyl-CoA carboxylase	6.4.1.2	Human	10215591
acetyl-CoA carboxylase	6.4.1.2	Human	10757783
acetyl-CoA carboxylase	6.4.1.2	Human	10945143
acetyl-CoA carboxylase	6.4.1.2	Human	11078738

acetyl-CoA carboxylase	6.4.1.2	Human	11205884
acetyl-CoA carboxylase	6.4.1.2	Human	11504381
acetyl-CoA carboxylase	6.4.1.2	Human	11515553
acetyl-CoA carboxylase	6.4.1.2	Human	11546765
acetyl-CoA carboxylase	6.4.1.2	Human	12440972
acetyl-CoA carboxylase	6.4.1.2	Human	14627750
acetyl-CoA carboxylase	6.4.1.2	Human	15333468
acetyl-CoA carboxylase	6.4.1.2	Human	15607423
acetyl-CoA carboxylase	6.4.1.2	Human	15607568
acetyl-CoA carboxylase	6.4.1.2	Human	16222055
acetyl-CoA carboxylase	6.4.1.2	Human	16707454
acetyl-CoA carboxylase	6.4.1.2	Human	16968879
acetyl-CoA carboxylase	6.4.1.2	Human	17266990
acetyl-CoA carboxylase	6.4.1.2	Human	17653193
acetyl-CoA carboxylase	6.4.1.2	Human	1978829
acetyl-CoA carboxylase	6.4.1.2	Human	2570725
acetyl-CoA carboxylase	6.4.1.2	Human	2861941
acetyl-CoA carboxylase	6.4.1.2	Human	2894828
acetyl-CoA carboxylase	6.4.1.2	Human	7436865
acetyl-CoA carboxylase	6.4.1.2	Human	7903266
acetyl-CoA carboxylase	6.4.1.2	Human	7915138
acetyl-CoA carboxylase	6.4.1.2	Human	8814137
acetyl-CoA carboxylase	6.4.1.2	Human	9028876
acetyl-CoA carboxylase	6.4.1.2	Human	9082912
acetyl-CoA carboxylase	6.4.1.2	Human	9109840
alcohol dehydrogenase	1.1.1.1	Mouse	1096557
alcohol dehydrogenase	1.1.1.1	Mouse	11303599
alcohol dehydrogenase	1.1.1.1	Mouse	1148277
alcohol dehydrogenase	1.1.1.1	Mouse	12147722
alcohol dehydrogenase	1.1.1.1	Mouse	12489977
alcohol dehydrogenase	1.1.1.1	Mouse	16662798
alcohol dehydrogenase	1.1.1.1	Mouse	167557
alcohol dehydrogenase	1.1.1.1	Mouse	2932116
alcohol dehydrogenase	1.1.1.1	Mouse	3067025
alcohol dehydrogenase	1.1.1.1	Mouse	3893194
alcohol dehydrogenase	1.1.1.1	Mouse	4038269
alcohol dehydrogenase	1.1.1.1	Mouse	6340613
alcohol dehydrogenase	1.1.1.1	Mouse	6341787
alcohol dehydrogenase	1.1.1.1	Mouse	6356161
alcohol dehydrogenase	1.1.1.1	Mouse	6363888
alcohol dehydrogenase	1.1.1.1	Mouse	6370140
alcohol dehydrogenase	1.1.1.1	Mouse	8277258
alcohol dehydrogenase	1.1.1.1	Mouse	8692838

alcohol dehydrogenase	1.1.1.1	Mouse	8905240
alcohol dehydrogenase	1.1.1.1	Mouse	9526508
retinol dehydrogenase	1.1.1.105	Mouse	11377978
3beta-hydroxy-Delta5-steroid dehydrogenase	1.1.1.145	Mouse	12441193
3beta-hydroxy-Delta5-steroid dehydrogenase	1.1.1.145	Mouse	12782399
3beta-hydroxy-Delta5-steroid dehydrogenase	1.1.1.145	Mouse	2019257
3beta-hydroxy-Delta5-steroid dehydrogenase	1.1.1.145	Mouse	239964
3beta-hydroxy-Delta5-steroid dehydrogenase	1.1.1.145	Mouse	7736258
3beta-hydroxy-Delta5-steroid dehydrogenase	1.1.1.145	Mouse	8574339
IMP dehydrogenase	1.1.1.205	Mouse	10194364
IMP dehydrogenase	1.1.1.205	Mouse	10390601
IMP dehydrogenase	1.1.1.205	Mouse	10390603
IMP dehydrogenase	1.1.1.205	Mouse	10391669
IMP dehydrogenase	1.1.1.205	Mouse	10417742
IMP dehydrogenase	1.1.1.205	Mouse	10930578
IMP dehydrogenase	1.1.1.205	Mouse	10953035
IMP dehydrogenase	1.1.1.205	Mouse	10953295
IMP dehydrogenase	1.1.1.205	Mouse	10973868
IMP dehydrogenase	1.1.1.205	Mouse	11003640
IMP dehydrogenase	1.1.1.205	Mouse	1106431
IMP dehydrogenase	1.1.1.205	Mouse	11076502
IMP dehydrogenase	1.1.1.205	Mouse	11145582
IMP dehydrogenase	1.1.1.205	Mouse	11223253
IMP dehydrogenase	1.1.1.205	Mouse	11233304
IMP dehydrogenase	1.1.1.205	Mouse	11288107
IMP dehydrogenase	1.1.1.205	Mouse	11454943
IMP dehydrogenase	1.1.1.205	Mouse	11522119
IMP dehydrogenase	1.1.1.205	Mouse	11566360
IMP dehydrogenase	1.1.1.205	Mouse	11712223
IMP dehydrogenase	1.1.1.205	Mouse	11724288
IMP dehydrogenase	1.1.1.205	Mouse	11875050
IMP dehydrogenase	1.1.1.205	Mouse	11966437
IMP dehydrogenase	1.1.1.205	Mouse	11966440
IMP dehydrogenase	1.1.1.205	Mouse	11966441
IMP dehydrogenase	1.1.1.205	Mouse	12014950
IMP dehydrogenase	1.1.1.205	Mouse	12183689
IMP dehydrogenase	1.1.1.205	Mouse	12213477

IMP dehydrogenase	1.1.1.205	Mouse	12235158
IMP dehydrogenase	1.1.1.205	Mouse	12403633
IMP dehydrogenase	1.1.1.205	Mouse	12559919
IMP dehydrogenase	1.1.1.205	Mouse	12609835
IMP dehydrogenase	1.1.1.205	Mouse	12746440
IMP dehydrogenase	1.1.1.205	Mouse	12773970
IMP dehydrogenase	1.1.1.205	Mouse	12944494
IMP dehydrogenase	1.1.1.205	Mouse	1353938
IMP dehydrogenase	1.1.1.205	Mouse	1356621
IMP dehydrogenase	1.1.1.205	Mouse	14703952
IMP dehydrogenase	1.1.1.205	Mouse	14757177
IMP dehydrogenase	1.1.1.205	Mouse	14766016
IMP dehydrogenase	1.1.1.205	Mouse	14973196
IMP dehydrogenase	1.1.1.205	Mouse	14981049
IMP dehydrogenase	1.1.1.205	Mouse	15043157
IMP dehydrogenase	1.1.1.205	Mouse	15083807
IMP dehydrogenase	1.1.1.205	Mouse	15292516
IMP dehydrogenase	1.1.1.205	Mouse	15355510
IMP dehydrogenase	1.1.1.205	Mouse	15829418
IMP dehydrogenase	1.1.1.205	Mouse	15869715
IMP dehydrogenase	1.1.1.205	Mouse	15882147
IMP dehydrogenase	1.1.1.205	Mouse	15940263
IMP dehydrogenase	1.1.1.205	Mouse	16128570
IMP dehydrogenase	1.1.1.205	Mouse	16243838
IMP dehydrogenase	1.1.1.205	Mouse	16248022
IMP dehydrogenase	1.1.1.205	Mouse	16333815
IMP dehydrogenase	1.1.1.205	Mouse	16647299
IMP dehydrogenase	1.1.1.205	Mouse	16725387
IMP dehydrogenase	1.1.1.205	Mouse	1677309
IMP dehydrogenase	1.1.1.205	Mouse	16936083
IMP dehydrogenase	1.1.1.205	Mouse	17100698
IMP dehydrogenase	1.1.1.205	Mouse	1717828
IMP dehydrogenase	1.1.1.205	Mouse	1723703
IMP dehydrogenase	1.1.1.205	Mouse	1975748
IMP dehydrogenase	1.1.1.205	Mouse	197916
IMP dehydrogenase	1.1.1.205	Mouse	2902093
IMP dehydrogenase	1.1.1.205	Mouse	3314714
IMP dehydrogenase	1.1.1.205	Mouse	4868171
IMP dehydrogenase	1.1.1.205	Mouse	6120758
IMP dehydrogenase	1.1.1.205	Mouse	7476879
IMP dehydrogenase	1.1.1.205	Mouse	7476895
IMP dehydrogenase	1.1.1.205	Mouse	7520100
IMP dehydrogenase	1.1.1.205	Mouse	7874783

IMP dehydrogenase	1.1.1.205	Mouse	7903533
IMP dehydrogenase	1.1.1.205	Mouse	7914720
IMP dehydrogenase	1.1.1.205	Mouse	8103312
IMP dehydrogenase	1.1.1.205	Mouse	8555204
IMP dehydrogenase	1.1.1.205	Mouse	8560580
IMP dehydrogenase	1.1.1.205	Mouse	8830834
IMP dehydrogenase	1.1.1.205	Mouse	8869741
IMP dehydrogenase	1.1.1.205	Mouse	8910338
IMP dehydrogenase	1.1.1.205	Mouse	9042309
IMP dehydrogenase	1.1.1.205	Mouse	9108641
IMP dehydrogenase	1.1.1.205	Mouse	9268334
IMP dehydrogenase	1.1.1.205	Mouse	9278455
IMP dehydrogenase	1.1.1.205	Mouse	9339960
IMP dehydrogenase	1.1.1.205	Mouse	9399601
IMP dehydrogenase	1.1.1.205	Mouse	9413163
IMP dehydrogenase	1.1.1.205	Mouse	9436988
IMP dehydrogenase	1.1.1.205	Mouse	9752721
IMP dehydrogenase	1.1.1.205	Mouse	9766533
IMP dehydrogenase	1.1.1.205	Mouse	9881055
aldehyde reductase	1.1.1.21	Mouse	10424772
aldehyde reductase	1.1.1.21	Mouse	10656235
aldehyde reductase	1.1.1.21	Mouse	11095596
aldehyde reductase	1.1.1.21	Mouse	11370705
aldehyde reductase	1.1.1.21	Mouse	11422753
aldehyde reductase	1.1.1.21	Mouse	11440832
aldehyde reductase	1.1.1.21	Mouse	11798960
aldehyde reductase	1.1.1.21	Mouse	12135102
aldehyde reductase	1.1.1.21	Mouse	12363257
aldehyde reductase	1.1.1.21	Mouse	12394272
aldehyde reductase	1.1.1.21	Mouse	12871133
aldehyde reductase	1.1.1.21	Mouse	12871136
aldehyde reductase	1.1.1.21	Mouse	12881532
aldehyde reductase	1.1.1.21	Mouse	1393828
aldehyde reductase	1.1.1.21	Mouse	14577653
aldehyde reductase	1.1.1.21	Mouse	1499867
aldehyde reductase	1.1.1.21	Mouse	15210146
aldehyde reductase	1.1.1.21	Mouse	15569136
aldehyde reductase	1.1.1.21	Mouse	15584919
aldehyde reductase	1.1.1.21	Mouse	15734861
aldehyde reductase	1.1.1.21	Mouse	15736047
aldehyde reductase	1.1.1.21	Mouse	16026266
aldehyde reductase	1.1.1.21	Mouse	16037296
aldehyde reductase	1.1.1.21	Mouse	16048249

aldehyde reductase	1.1.1.21	Mouse	16114079
aldehyde reductase	1.1.1.21	Mouse	16452468
aldehyde reductase	1.1.1.21	Mouse	16806328
aldehyde reductase	1.1.1.21	Mouse	16870454
aldehyde reductase	1.1.1.21	Mouse	16900332
aldehyde reductase	1.1.1.21	Mouse	16911628
aldehyde reductase	1.1.1.21	Mouse	2120282
aldehyde reductase	1.1.1.21	Mouse	3025043
aldehyde reductase	1.1.1.21	Mouse	7641310
aldehyde reductase	1.1.1.21	Mouse	8457142
aldehyde reductase	1.1.1.21	Mouse	9215310
aldehyde reductase	1.1.1.21	Mouse	9454604
aldehyde reductase	1.1.1.21	Mouse	9481088
aldehyde reductase	1.1.1.21	Mouse	9709964
UDP-glucose 6-dehydrogenase	1.1.1.22	Mouse	11044215
UDP-glucose 6-dehydrogenase	1.1.1.22	Mouse	15741737
UDP-glucose 6-dehydrogenase	1.1.1.22	Mouse	2778766
UDP-glucose 6-dehydrogenase	1.1.1.22	Mouse	8471533
hydroxymethylglutaryl-CoA reductase (NADPH)	1.1.1.34	Mouse	10084306
hydroxymethylglutaryl-CoA reductase (NADPH)	1.1.1.34	Mouse	10204089
hydroxymethylglutaryl-CoA reductase (NADPH)	1.1.1.34	Mouse	10377386
hydroxymethylglutaryl-CoA reductase (NADPH)	1.1.1.34	Mouse	10892724
hydroxymethylglutaryl-CoA reductase (NADPH)	1.1.1.34	Mouse	10964918
hydroxymethylglutaryl-CoA reductase (NADPH)	1.1.1.34	Mouse	11043510
hydroxymethylglutaryl-CoA reductase (NADPH)	1.1.1.34	Mouse	11516100
hydroxymethylglutaryl-CoA reductase (NADPH)	1.1.1.34	Mouse	11881568
hydroxymethylglutaryl-CoA reductase (NADPH)	1.1.1.34	Mouse	12405293
hydroxymethylglutaryl-CoA reductase (NADPH)	1.1.1.34	Mouse	12467639
hydroxymethylglutaryl-CoA reductase (NADPH)	1.1.1.34	Mouse	12736772
hydroxymethylglutaryl-CoA reductase (NADPH)	1.1.1.34	Mouse	14523992

hydroxymethylglutaryl-CoA reductase (NADPH)	1.1.1.34	Mouse	14720509
hydroxymethylglutaryl-CoA reductase (NADPH)	1.1.1.34	Mouse	15034683
hydroxymethylglutaryl-CoA reductase (NADPH)	1.1.1.34	Mouse	15248477
hydroxymethylglutaryl-CoA reductase (NADPH)	1.1.1.34	Mouse	15605175
hydroxymethylglutaryl-CoA reductase (NADPH)	1.1.1.34	Mouse	16101500
hydroxymethylglutaryl-CoA reductase (NADPH)	1.1.1.34	Mouse	16168377
hydroxymethylglutaryl-CoA reductase (NADPH)	1.1.1.34	Mouse	1629633
hydroxymethylglutaryl-CoA reductase (NADPH)	1.1.1.34	Mouse	1652430
hydroxymethylglutaryl-CoA reductase (NADPH)	1.1.1.34	Mouse	16611135
hydroxymethylglutaryl-CoA reductase (NADPH)	1.1.1.34	Mouse	187533
hydroxymethylglutaryl-CoA reductase (NADPH)	1.1.1.34	Mouse	1954650
hydroxymethylglutaryl-CoA reductase (NADPH)	1.1.1.34	Mouse	216867
hydroxymethylglutaryl-CoA reductase (NADPH)	1.1.1.34	Mouse	2719596
hydroxymethylglutaryl-CoA reductase (NADPH)	1.1.1.34	Mouse	278983
hydroxymethylglutaryl-CoA reductase (NADPH)	1.1.1.34	Mouse	2921640
hydroxymethylglutaryl-CoA reductase (NADPH)	1.1.1.34	Mouse	2995161
hydroxymethylglutaryl-CoA reductase (NADPH)	1.1.1.34	Mouse	3055919
hydroxymethylglutaryl-CoA reductase (NADPH)	1.1.1.34	Mouse	3131638
hydroxymethylglutaryl-CoA reductase (NADPH)	1.1.1.34	Mouse	3308873
hydroxymethylglutaryl-CoA reductase (NADPH)	1.1.1.34	Mouse	3314447
hydroxymethylglutaryl-CoA reductase (NADPH)	1.1.1.34	Mouse	3689494

hydroxymethylglutaryl-CoA reductase (NADPH)	1.1.1.34	Mouse	3968683
hydroxymethylglutaryl-CoA reductase (NADPH)	1.1.1.34	Mouse	4075700
hydroxymethylglutaryl-CoA reductase (NADPH)	1.1.1.34	Mouse	6088070
hydroxymethylglutaryl-CoA reductase (NADPH)	1.1.1.34	Mouse	6256737
hydroxymethylglutaryl-CoA reductase (NADPH)	1.1.1.34	Mouse	6274615
hydroxymethylglutaryl-CoA reductase (NADPH)	1.1.1.34	Mouse	6286363
hydroxymethylglutaryl-CoA reductase (NADPH)	1.1.1.34	Mouse	6347025
hydroxymethylglutaryl-CoA reductase (NADPH)	1.1.1.34	Mouse	6396116
hydroxymethylglutaryl-CoA reductase (NADPH)	1.1.1.34	Mouse	6594693
hydroxymethylglutaryl-CoA reductase (NADPH)	1.1.1.34	Mouse	666819
hydroxymethylglutaryl-CoA reductase (NADPH)	1.1.1.34	Mouse	6685129
hydroxymethylglutaryl-CoA reductase (NADPH)	1.1.1.34	Mouse	6698994
hydroxymethylglutaryl-CoA reductase (NADPH)	1.1.1.34	Mouse	8054400
hydroxymethylglutaryl-CoA reductase (NADPH)	1.1.1.34	Mouse	8182149
hydroxymethylglutaryl-CoA reductase (NADPH)	1.1.1.34	Mouse	8393338
hydroxymethylglutaryl-CoA reductase (NADPH)	1.1.1.34	Mouse	8504036
hydroxymethylglutaryl-CoA reductase (NADPH)	1.1.1.34	Mouse	8772195
hydroxymethylglutaryl-CoA reductase (NADPH)	1.1.1.34	Mouse	9151797
hydroxymethylglutaryl-CoA reductase (NADPH)	1.1.1.34	Mouse	9185766
hydroxymethylglutaryl-CoA reductase (NADPH)	1.1.1.34	Mouse	9372476
hydroxymethylglutaryl-CoA reductase (NADPH)	1.1.1.34	Mouse	9558731

hydroxymethylglutaryl-CoA reductase (NADPH)	1.1.1.34	Mouse	9802623
3-hydroxyacyl-CoA dehydrogenase	1.1.1.35	Mouse	15358356
3-hydroxyacyl-CoA dehydrogenase	1.1.1.35	Mouse	1637289
3-hydroxyacyl-CoA dehydrogenase	1.1.1.35	Mouse	2388659
3-hydroxyacyl-CoA dehydrogenase	1.1.1.35	Mouse	6588129
malate dehydrogenase	1.1.1.37	Mouse	11583380
malate dehydrogenase	1.1.1.37	Mouse	11767008
malate dehydrogenase	1.1.1.37	Mouse	11855723
malate dehydrogenase	1.1.1.37	Mouse	131232
malate dehydrogenase	1.1.1.37	Mouse	16028114
malate dehydrogenase	1.1.1.37	Mouse	16212411
malate dehydrogenase	1.1.1.37	Mouse	16661455
malate dehydrogenase	1.1.1.37	Mouse	3995045
malate dehydrogenase	1.1.1.37	Mouse	4053567
malate dehydrogenase	1.1.1.37	Mouse	7138874
malate dehydrogenase	1.1.1.37	Mouse	8624506
malate dehydrogenase	1.1.1.37	Mouse	9348107
isocitrate dehydrogenase (NAD+)	1.1.1.41	Mouse	10461937
isocitrate dehydrogenase (NAD+)	1.1.1.41	Mouse	12033940
isocitrate dehydrogenase (NAD+)	1.1.1.41	Mouse	12619682
isocitrate dehydrogenase (NAD+)	1.1.1.41	Mouse	14555658
isocitrate dehydrogenase (NAD+)	1.1.1.41	Mouse	15173171
isocitrate dehydrogenase (NAD+)	1.1.1.41	Mouse	15314217
isocitrate dehydrogenase (NAD+)	1.1.1.41	Mouse	16415587
isocitrate dehydrogenase (NAD+)	1.1.1.41	Mouse	6389540
isocitrate dehydrogenase (NAD+)	1.1.1.41	Mouse	7710326
isocitrate dehydrogenase (NAD+)	1.1.1.41	Mouse	8626605
isocitrate dehydrogenase (NAD+)	1.1.1.41	Mouse	9733544
isocitrate dehydrogenase (NAD+)	1.1.1.41	Mouse	9881153
glucose-6-phosphate dehydrogenase	1.1.1.49	Mouse	10098886
glucose-6-phosphate dehydrogenase	1.1.1.49	Mouse	10099785
glucose-6-phosphate dehydrogenase	1.1.1.49	Mouse	10329961
glucose-6-phosphate dehydrogenase	1.1.1.49	Mouse	10825753
glucose-6-phosphate dehydrogenase	1.1.1.49	Mouse	10998184
glucose-6-phosphate dehydrogenase	1.1.1.49	Mouse	11023706
glucose-6-phosphate dehydrogenase	1.1.1.49	Mouse	11245448
glucose-6-phosphate dehydrogenase	1.1.1.49	Mouse	11463792
glucose-6-phosphate dehydrogenase	1.1.1.49	Mouse	11520909
glucose-6-phosphate dehydrogenase	1.1.1.49	Mouse	12027950
glucose-6-phosphate dehydrogenase	1.1.1.49	Mouse	12204336
glucose-6-phosphate dehydrogenase	1.1.1.49	Mouse	12393032
glucose-6-phosphate dehydrogenase	1.1.1.49	Mouse	12414804

glucose-6-phosphate dehydrogenase	1.1.1.49	Mouse	12453665
glucose-6-phosphate dehydrogenase	1.1.1.49	Mouse	12472120
glucose-6-phosphate dehydrogenase	1.1.1.49	Mouse	12502759
glucose-6-phosphate dehydrogenase	1.1.1.49	Mouse	131232
glucose-6-phosphate dehydrogenase	1.1.1.49	Mouse	1384463
glucose-6-phosphate dehydrogenase	1.1.1.49	Mouse	1417703
glucose-6-phosphate dehydrogenase	1.1.1.49	Mouse	147929
glucose-6-phosphate dehydrogenase	1.1.1.49	Mouse	15331344
glucose-6-phosphate dehydrogenase	1.1.1.49	Mouse	15345489
glucose-6-phosphate dehydrogenase	1.1.1.49	Mouse	15527069
glucose-6-phosphate dehydrogenase	1.1.1.49	Mouse	15550513
glucose-6-phosphate dehydrogenase	1.1.1.49	Mouse	15634201
glucose-6-phosphate dehydrogenase	1.1.1.49	Mouse	15739803
glucose-6-phosphate dehydrogenase	1.1.1.49	Mouse	15760711
glucose-6-phosphate dehydrogenase	1.1.1.49	Mouse	15858258
glucose-6-phosphate dehydrogenase	1.1.1.49	Mouse	15975496
glucose-6-phosphate dehydrogenase	1.1.1.49	Mouse	16039947
glucose-6-phosphate dehydrogenase	1.1.1.49	Mouse	16439706
glucose-6-phosphate dehydrogenase	1.1.1.49	Mouse	16849632
glucose-6-phosphate dehydrogenase	1.1.1.49	Mouse	17157446
glucose-6-phosphate dehydrogenase	1.1.1.49	Mouse	1830744
glucose-6-phosphate dehydrogenase	1.1.1.49	Mouse	1922658
glucose-6-phosphate dehydrogenase	1.1.1.49	Mouse	1978808
glucose-6-phosphate dehydrogenase	1.1.1.49	Mouse	2208076
glucose-6-phosphate dehydrogenase	1.1.1.49	Mouse	2296762
glucose-6-phosphate dehydrogenase	1.1.1.49	Mouse	2767006
glucose-6-phosphate dehydrogenase	1.1.1.49	Mouse	2808772
glucose-6-phosphate dehydrogenase	1.1.1.49	Mouse	2846196
glucose-6-phosphate dehydrogenase	1.1.1.49	Mouse	2984461
glucose-6-phosphate dehydrogenase	1.1.1.49	Mouse	3116361
glucose-6-phosphate dehydrogenase	1.1.1.49	Mouse	3161339
glucose-6-phosphate dehydrogenase	1.1.1.49	Mouse	3316204
glucose-6-phosphate dehydrogenase	1.1.1.49	Mouse	3337882
glucose-6-phosphate dehydrogenase	1.1.1.49	Mouse	3365274
glucose-6-phosphate dehydrogenase	1.1.1.49	Mouse	3532684
glucose-6-phosphate dehydrogenase	1.1.1.49	Mouse	3621197
glucose-6-phosphate dehydrogenase	1.1.1.49	Mouse	3717951
glucose-6-phosphate dehydrogenase	1.1.1.49	Mouse	3765490
glucose-6-phosphate dehydrogenase	1.1.1.49	Mouse	3949801
glucose-6-phosphate dehydrogenase	1.1.1.49	Mouse	4288679
glucose-6-phosphate dehydrogenase	1.1.1.49	Mouse	4382249
glucose-6-phosphate dehydrogenase	1.1.1.49	Mouse	4400642
glucose-6-phosphate dehydrogenase	1.1.1.49	Mouse	4941552

glucose-6-phosphate dehydrogenase	1.1.1.49	Mouse	6295653
glucose-6-phosphate dehydrogenase	1.1.1.49	Mouse	6341787
glucose-6-phosphate dehydrogenase	1.1.1.49	Mouse	6363888
glucose-6-phosphate dehydrogenase	1.1.1.49	Mouse	6420889
glucose-6-phosphate dehydrogenase	1.1.1.49	Mouse	645360
glucose-6-phosphate dehydrogenase	1.1.1.49	Mouse	6591771
glucose-6-phosphate dehydrogenase	1.1.1.49	Mouse	6696439
glucose-6-phosphate dehydrogenase	1.1.1.49	Mouse	7126822
glucose-6-phosphate dehydrogenase	1.1.1.49	Mouse	7578910
glucose-6-phosphate dehydrogenase	1.1.1.49	Mouse	7681896
glucose-6-phosphate dehydrogenase	1.1.1.49	Mouse	7768207
glucose-6-phosphate dehydrogenase	1.1.1.49	Mouse	7930940
glucose-6-phosphate dehydrogenase	1.1.1.49	Mouse	8316633
glucose-6-phosphate dehydrogenase	1.1.1.49	Mouse	864
glucose-6-phosphate dehydrogenase	1.1.1.49	Mouse	8760336
glucose-6-phosphate dehydrogenase	1.1.1.49	Mouse	8797095
glucose-6-phosphate dehydrogenase	1.1.1.49	Mouse	8857518
glucose-6-phosphate dehydrogenase	1.1.1.49	Mouse	8910528
glucose-6-phosphate dehydrogenase	1.1.1.49	Mouse	8954569
glucose-6-phosphate dehydrogenase	1.1.1.49	Mouse	9042391
glucose-6-phosphate dehydrogenase	1.1.1.49	Mouse	9553122
glucose-6-phosphate dehydrogenase	1.1.1.49	Mouse	9581796
glucose-6-phosphate dehydrogenase	1.1.1.49	Mouse	9915806
L-gulonolactone oxidase	1.1.3.8	Mouse	15674730
L-gulonolactone oxidase	1.1.3.8	Mouse	16177205
L-gulonolactone oxidase	1.1.3.8	Mouse	16632110
L-gulonolactone oxidase	1.1.3.8	Mouse	1889832
L-gulonolactone oxidase	1.1.3.8	Mouse	1962571
L-gulonolactone oxidase	1.1.3.8	Mouse	369458
choline dehydrogenase	1.1.99.1	Mouse	8868068
ubiquinol---cytochrome-c reductase	1.10.2.2	Mouse	16005845
ubiquinol---cytochrome-c reductase	1.10.2.2	Mouse	17223530
tryptophan 2,3-dioxygenase	1.13.11.11	Mouse	10966936
tryptophan 2,3-dioxygenase	1.13.11.11	Mouse	17761498
tryptophan 2,3-dioxygenase	1.13.11.11	Mouse	2215078
tryptophan 2,3-dioxygenase	1.13.11.11	Mouse	4293961
tryptophan 2,3-dioxygenase	1.13.11.11	Mouse	8806758
tryptophan 2,3-dioxygenase	1.13.11.11	Mouse	9291104
4-hydroxyphenylpyruvate dioxygenase	1.13.11.27	Mouse	10098661
4-hydroxyphenylpyruvate dioxygenase	1.13.11.27	Mouse	12014960
4-hydroxyphenylpyruvate dioxygenase	1.13.11.27	Mouse	12127941
4-hydroxyphenylpyruvate dioxygenase	1.13.11.27	Mouse	240411
4-hydroxyphenylpyruvate dioxygenase	1.13.11.27	Mouse	9701587

indoleamine 2,3-dioxygenase	1.13.11.52	Mouse	10721098
indoleamine 2,3-dioxygenase	1.13.11.52	Mouse	10731095
indoleamine 2,3-dioxygenase	1.13.11.52	Mouse	10833386
indoleamine 2,3-dioxygenase	1.13.11.52	Mouse	10926204
indoleamine 2,3-dioxygenase	1.13.11.52	Mouse	10939283
indoleamine 2,3-dioxygenase	1.13.11.52	Mouse	10939284
indoleamine 2,3-dioxygenase	1.13.11.52	Mouse	10957719
indoleamine 2,3-dioxygenase	1.13.11.52	Mouse	11180976
indoleamine 2,3-dioxygenase	1.13.11.52	Mouse	11230514
indoleamine 2,3-dioxygenase	1.13.11.52	Mouse	11440641
indoleamine 2,3-dioxygenase	1.13.11.52	Mouse	11477543
indoleamine 2,3-dioxygenase	1.13.11.52	Mouse	11507170
indoleamine 2,3-dioxygenase	1.13.11.52	Mouse	12414962
indoleamine 2,3-dioxygenase	1.13.11.52	Mouse	12832720
indoleamine 2,3-dioxygenase	1.13.11.52	Mouse	12848846
indoleamine 2,3-dioxygenase	1.13.11.52	Mouse	15206741
indoleamine 2,3-dioxygenase	1.13.11.52	Mouse	15254594
indoleamine 2,3-dioxygenase	1.13.11.52	Mouse	15358362
indoleamine 2,3-dioxygenase	1.13.11.52	Mouse	15542091
indoleamine 2,3-dioxygenase	1.13.11.52	Mouse	15853924
indoleamine 2,3-dioxygenase	1.13.11.52	Mouse	15961516
indoleamine 2,3-dioxygenase	1.13.11.52	Mouse	16075385
indoleamine 2,3-dioxygenase	1.13.11.52	Mouse	16083346
indoleamine 2,3-dioxygenase	1.13.11.52	Mouse	16176799
indoleamine 2,3-dioxygenase	1.13.11.52	Mouse	16624246
indoleamine 2,3-dioxygenase	1.13.11.52	Mouse	17055065
indoleamine 2,3-dioxygenase	1.13.11.52	Mouse	8423409
indoleamine 2,3-dioxygenase	1.13.11.52	Mouse	8702590
indoleamine 2,3-dioxygenase	1.13.11.52	Mouse	9466588
cholestanetriol 26-monooxygenase	1.14.13.15	Mouse	11108738
cholestanetriol 26-monooxygenase	1.14.13.15	Mouse	11166758
cholestanetriol 26-monooxygenase	1.14.13.15	Mouse	11406622
cholestanetriol 26-monooxygenase	1.14.13.15	Mouse	15936349
cholestanetriol 26-monooxygenase	1.14.13.15	Mouse	2019602
cholestanetriol 26-monooxygenase	1.14.13.15	Mouse	7557873
cholestanetriol 26-monooxygenase	1.14.13.15	Mouse	8001744
cholesterol 7alpha-monooxygenase	1.14.13.17	Mouse	10334992
cholesterol 7alpha-monooxygenase	1.14.13.17	Mouse	10405006
cholesterol 7alpha-monooxygenase	1.14.13.17	Mouse	10431389
cholesterol 7alpha-monooxygenase	1.14.13.17	Mouse	10588945
cholesterol 7alpha-monooxygenase	1.14.13.17	Mouse	10599986
cholesterol 7alpha-monooxygenase	1.14.13.17	Mouse	10656290
cholesterol 7alpha-monooxygenase	1.14.13.17	Mouse	10731667

cholesterol 7alpha-monooxygenase	1.14.13.17	Mouse	10744771
cholesterol 7alpha-monooxygenase	1.14.13.17	Mouse	10858029
cholesterol 7alpha-monooxygenase	1.14.13.17	Mouse	10900260
cholesterol 7alpha-monooxygenase	1.14.13.17	Mouse	10936612
cholesterol 7alpha-monooxygenase	1.14.13.17	Mouse	11030331
cholesterol 7alpha-monooxygenase	1.14.13.17	Mouse	11075809
cholesterol 7alpha-monooxygenase	1.14.13.17	Mouse	11108738
cholesterol 7alpha-monooxygenase	1.14.13.17	Mouse	11136553
cholesterol 7alpha-monooxygenase	1.14.13.17	Mouse	11254888
cholesterol 7alpha-monooxygenase	1.14.13.17	Mouse	11264982
cholesterol 7alpha-monooxygenase	1.14.13.17	Mouse	11402042
cholesterol 7alpha-monooxygenase	1.14.13.17	Mouse	11406622
cholesterol 7alpha-monooxygenase	1.14.13.17	Mouse	11427207
cholesterol 7alpha-monooxygenase	1.14.13.17	Mouse	11438503
cholesterol 7alpha-monooxygenase	1.14.13.17	Mouse	11557507
cholesterol 7alpha-monooxygenase	1.14.13.17	Mouse	11604260
cholesterol 7alpha-monooxygenase	1.14.13.17	Mouse	11787596
cholesterol 7alpha-monooxygenase	1.14.13.17	Mouse	11967026
cholesterol 7alpha-monooxygenase	1.14.13.17	Mouse	12042433
cholesterol 7alpha-monooxygenase	1.14.13.17	Mouse	12049994
cholesterol 7alpha-monooxygenase	1.14.13.17	Mouse	12393855
cholesterol 7alpha-monooxygenase	1.14.13.17	Mouse	1245792
cholesterol 7alpha-monooxygenase	1.14.13.17	Mouse	12554795
cholesterol 7alpha-monooxygenase	1.14.13.17	Mouse	12562858
cholesterol 7alpha-monooxygenase	1.14.13.17	Mouse	12675851
cholesterol 7alpha-monooxygenase	1.14.13.17	Mouse	12787409
cholesterol 7alpha-monooxygenase	1.14.13.17	Mouse	12805410
cholesterol 7alpha-monooxygenase	1.14.13.17	Mouse	12865425
cholesterol 7alpha-monooxygenase	1.14.13.17	Mouse	12917427
cholesterol 7alpha-monooxygenase	1.14.13.17	Mouse	14660582
cholesterol 7alpha-monooxygenase	1.14.13.17	Mouse	14698038
cholesterol 7alpha-monooxygenase	1.14.13.17	Mouse	14748721
cholesterol 7alpha-monooxygenase	1.14.13.17	Mouse	14762172
cholesterol 7alpha-monooxygenase	1.14.13.17	Mouse	14960319
cholesterol 7alpha-monooxygenase	1.14.13.17	Mouse	15145977
cholesterol 7alpha-monooxygenase	1.14.13.17	Mouse	15241483
cholesterol 7alpha-monooxygenase	1.14.13.17	Mouse	15333704
cholesterol 7alpha-monooxygenase	1.14.13.17	Mouse	15375335
cholesterol 7alpha-monooxygenase	1.14.13.17	Mouse	15458444
cholesterol 7alpha-monooxygenase	1.14.13.17	Mouse	15574426
cholesterol 7alpha-monooxygenase	1.14.13.17	Mouse	15583480
cholesterol 7alpha-monooxygenase	1.14.13.17	Mouse	15649292
cholesterol 7alpha-monooxygenase	1.14.13.17	Mouse	15707388

cholesterol 7alpha-monooxygenase	1.14.13.17	Mouse	15750181
cholesterol 7alpha-monooxygenase	1.14.13.17	Mouse	15752749
cholesterol 7alpha-monooxygenase	1.14.13.17	Mouse	15795435
cholesterol 7alpha-monooxygenase	1.14.13.17	Mouse	15936349
cholesterol 7alpha-monooxygenase	1.14.13.17	Mouse	16075052
cholesterol 7alpha-monooxygenase	1.14.13.17	Mouse	16213224
cholesterol 7alpha-monooxygenase	1.14.13.17	Mouse	16271991
cholesterol 7alpha-monooxygenase	1.14.13.17	Mouse	16464476
cholesterol 7alpha-monooxygenase	1.14.13.17	Mouse	17054913
cholesterol 7alpha-monooxygenase	1.14.13.17	Mouse	223590
cholesterol 7alpha-monooxygenase	1.14.13.17	Mouse	2713876
cholesterol 7alpha-monooxygenase	1.14.13.17	Mouse	3768033
cholesterol 7alpha-monooxygenase	1.14.13.17	Mouse	3981265
cholesterol 7alpha-monooxygenase	1.14.13.17	Mouse	8663429
cholesterol 7alpha-monooxygenase	1.14.13.17	Mouse	8759369
cholesterol 7alpha-monooxygenase	1.14.13.17	Mouse	8858751
cholesterol 7alpha-monooxygenase	1.14.13.17	Mouse	8895609
cholesterol 7alpha-monooxygenase	1.14.13.17	Mouse	8943286
cholesterol 7alpha-monooxygenase	1.14.13.17	Mouse	9013589
cholesterol 7alpha-monooxygenase	1.14.13.17	Mouse	9151797
cholesterol 7alpha-monooxygenase	1.14.13.17	Mouse	9185766
cholesterol 7alpha-monooxygenase	1.14.13.17	Mouse	9462665
cholesterol 7alpha-monooxygenase	1.14.13.17	Mouse	9550534
cholesterol 7alpha-monooxygenase	1.14.13.17	Mouse	9558731
cholesterol 7alpha-monooxygenase	1.14.13.17	Mouse	9625597
cholesterol 7alpha-monooxygenase	1.14.13.17	Mouse	9630215
cholesterol 7alpha-monooxygenase	1.14.13.17	Mouse	9649606
cholesterol 7alpha-monooxygenase	1.14.13.17	Mouse	9721193
cholesterol 7alpha-monooxygenase	1.14.13.17	Mouse	9799805
cholesterol 7alpha-monooxygenase	1.14.13.17	Mouse	9881645
nitric-oxide synthase	1.14.13.39	Mouse	10630682
nitric-oxide synthase	1.14.13.39	Mouse	10821631
nitric-oxide synthase	1.14.13.39	Mouse	10868974
nitric-oxide synthase	1.14.13.39	Mouse	11095648
nitric-oxide synthase	1.14.13.39	Mouse	11292821
nitric-oxide synthase	1.14.13.39	Mouse	11328947
nitric-oxide synthase	1.14.13.39	Mouse	11468403
nitric-oxide synthase	1.14.13.39	Mouse	11556547
nitric-oxide synthase	1.14.13.39	Mouse	11852055
nitric-oxide synthase	1.14.13.39	Mouse	12176955
nitric-oxide synthase	1.14.13.39	Mouse	12297263
nitric-oxide synthase	1.14.13.39	Mouse	12354096
nitric-oxide synthase	1.14.13.39	Mouse	12402580

nitric-oxide synthase	1.14.13.39	Mouse	12503100
nitric-oxide synthase	1.14.13.39	Mouse	12566086
nitric-oxide synthase	1.14.13.39	Mouse	12591128
nitric-oxide synthase	1.14.13.39	Mouse	12810358
nitric-oxide synthase	1.14.13.39	Mouse	12830073
nitric-oxide synthase	1.14.13.39	Mouse	12855421
nitric-oxide synthase	1.14.13.39	Mouse	12869534
nitric-oxide synthase	1.14.13.39	Mouse	14662726
nitric-oxide synthase	1.14.13.39	Mouse	14668561
nitric-oxide synthase	1.14.13.39	Mouse	14769821
nitric-oxide synthase	1.14.13.39	Mouse	15099358
nitric-oxide synthase	1.14.13.39	Mouse	15167268
nitric-oxide synthase	1.14.13.39	Mouse	15223360
nitric-oxide synthase	1.14.13.39	Mouse	15448133
nitric-oxide synthase	1.14.13.39	Mouse	15588718
nitric-oxide synthase	1.14.13.39	Mouse	15698596
nitric-oxide synthase	1.14.13.39	Mouse	16179540
nitric-oxide synthase	1.14.13.39	Mouse	16249336
nitric-oxide synthase	1.14.13.39	Mouse	16297560
nitric-oxide synthase	1.14.13.39	Mouse	16315601
nitric-oxide synthase	1.14.13.39	Mouse	16464859
nitric-oxide synthase	1.14.13.39	Mouse	16641207
nitric-oxide synthase	1.14.13.39	Mouse	17003331
nitric-oxide synthase	1.14.13.39	Mouse	17293453
nitric-oxide synthase	1.14.13.39	Mouse	7689840
nitric-oxide synthase	1.14.13.39	Mouse	8694803
nitric-oxide synthase	1.14.13.39	Mouse	8791097
nitric-oxide synthase	1.14.13.39	Mouse	8945918
nitric-oxide synthase	1.14.13.39	Mouse	8958563
nitric-oxide synthase	1.14.13.39	Mouse	9247967
nitric-oxide synthase	1.14.13.39	Mouse	9336394
nitric-oxide synthase	1.14.13.39	Mouse	9409304
nitric-oxide synthase	1.14.13.39	Mouse	9525991
nitric-oxide synthase	1.14.13.39	Mouse	9654136
nitric-oxide synthase	1.14.13.39	Mouse	9665318
nitric-oxide synthase	1.14.13.39	Mouse	9822514
nitric-oxide synthase	1.14.13.39	Mouse	9856817
kynurenine 3-monooxygenase	1.14.13.9	Mouse	42292
kynurenine 3-monooxygenase	1.14.13.9	Mouse	7131096
kynurenine 3-monooxygenase	1.14.13.9	Mouse	7131097
unspecific monooxygenase	1.14.14.1	Mouse	10462973
unspecific monooxygenase	1.14.14.1	Mouse	10473018
unspecific monooxygenase	1.14.14.1	Mouse	10474272

unspecific monooxygenase	1.14.14.1	Mouse	10690899
unspecific monooxygenase	1.14.14.1	Mouse	10713305
unspecific monooxygenase	1.14.14.1	Mouse	10746939
unspecific monooxygenase	1.14.14.1	Mouse	10947336
unspecific monooxygenase	1.14.14.1	Mouse	11116206
unspecific monooxygenase	1.14.14.1	Mouse	11259506
unspecific monooxygenase	1.14.14.1	Mouse	11312650
unspecific monooxygenase	1.14.14.1	Mouse	11403896
unspecific monooxygenase	1.14.14.1	Mouse	11502834
unspecific monooxygenase	1.14.14.1	Mouse	11922772
unspecific monooxygenase	1.14.14.1	Mouse	11961225
unspecific monooxygenase	1.14.14.1	Mouse	12053085
unspecific monooxygenase	1.14.14.1	Mouse	12439219
unspecific monooxygenase	1.14.14.1	Mouse	12606587
unspecific monooxygenase	1.14.14.1	Mouse	12706301
unspecific monooxygenase	1.14.14.1	Mouse	12715371
unspecific monooxygenase	1.14.14.1	Mouse	12736278
unspecific monooxygenase	1.14.14.1	Mouse	12810639
unspecific monooxygenase	1.14.14.1	Mouse	12845227
unspecific monooxygenase	1.14.14.1	Mouse	12957661
unspecific monooxygenase	1.14.14.1	Mouse	12960093
unspecific monooxygenase	1.14.14.1	Mouse	1339246
unspecific monooxygenase	1.14.14.1	Mouse	14501171
unspecific monooxygenase	1.14.14.1	Mouse	14580722
unspecific monooxygenase	1.14.14.1	Mouse	14623534
unspecific monooxygenase	1.14.14.1	Mouse	14965567
unspecific monooxygenase	1.14.14.1	Mouse	15226009
unspecific monooxygenase	1.14.14.1	Mouse	15255840
unspecific monooxygenase	1.14.14.1	Mouse	15319488
unspecific monooxygenase	1.14.14.1	Mouse	15322103
unspecific monooxygenase	1.14.14.1	Mouse	15582747
unspecific monooxygenase	1.14.14.1	Mouse	15591029
unspecific monooxygenase	1.14.14.1	Mouse	15623590
unspecific monooxygenase	1.14.14.1	Mouse	15876405
unspecific monooxygenase	1.14.14.1	Mouse	15885269
unspecific monooxygenase	1.14.14.1	Mouse	15930283
unspecific monooxygenase	1.14.14.1	Mouse	16006326
unspecific monooxygenase	1.14.14.1	Mouse	16077170
unspecific monooxygenase	1.14.14.1	Mouse	16109788
unspecific monooxygenase	1.14.14.1	Mouse	16109840
unspecific monooxygenase	1.14.14.1	Mouse	16170371
unspecific monooxygenase	1.14.14.1	Mouse	16260617
unspecific monooxygenase	1.14.14.1	Mouse	16285913

unspecific monooxygenase	1.14.14.1	Mouse	16303757
unspecific monooxygenase	1.14.14.1	Mouse	16322267
unspecific monooxygenase	1.14.14.1	Mouse	16426763
unspecific monooxygenase	1.14.14.1	Mouse	16473000
unspecific monooxygenase	1.14.14.1	Mouse	16541462
unspecific monooxygenase	1.14.14.1	Mouse	16611627
unspecific monooxygenase	1.14.14.1	Mouse	16733710
unspecific monooxygenase	1.14.14.1	Mouse	16763069
unspecific monooxygenase	1.14.14.1	Mouse	16877675
unspecific monooxygenase	1.14.14.1	Mouse	1694074
unspecific monooxygenase	1.14.14.1	Mouse	17005180
unspecific monooxygenase	1.14.14.1	Mouse	17079138
unspecific monooxygenase	1.14.14.1	Mouse	17573783
unspecific monooxygenase	1.14.14.1	Mouse	1835644
unspecific monooxygenase	1.14.14.1	Mouse	2149503
unspecific monooxygenase	1.14.14.1	Mouse	3419162
unspecific monooxygenase	1.14.14.1	Mouse	3626552
unspecific monooxygenase	1.14.14.1	Mouse	6233132
unspecific monooxygenase	1.14.14.1	Mouse	6580512
unspecific monooxygenase	1.14.14.1	Mouse	6645505
unspecific monooxygenase	1.14.14.1	Mouse	7083186
unspecific monooxygenase	1.14.14.1	Mouse	7581491
unspecific monooxygenase	1.14.14.1	Mouse	7626472
unspecific monooxygenase	1.14.14.1	Mouse	7694590
unspecific monooxygenase	1.14.14.1	Mouse	7949200
unspecific monooxygenase	1.14.14.1	Mouse	7968362
unspecific monooxygenase	1.14.14.1	Mouse	8017854
unspecific monooxygenase	1.14.14.1	Mouse	8073072
unspecific monooxygenase	1.14.14.1	Mouse	8126145
unspecific monooxygenase	1.14.14.1	Mouse	8698750
unspecific monooxygenase	1.14.14.1	Mouse	8824461
unspecific monooxygenase	1.14.14.1	Mouse	8989259
unspecific monooxygenase	1.14.14.1	Mouse	9027406
unspecific monooxygenase	1.14.14.1	Mouse	9038782
unspecific monooxygenase	1.14.14.1	Mouse	9187104
unspecific monooxygenase	1.14.14.1	Mouse	9365209
unspecific monooxygenase	1.14.14.1	Mouse	9415804
unspecific monooxygenase	1.14.14.1	Mouse	9442349
unspecific monooxygenase	1.14.14.1	Mouse	9456245
unspecific monooxygenase	1.14.14.1	Mouse	9511180
unspecific monooxygenase	1.14.14.1	Mouse	9523724
unspecific monooxygenase	1.14.14.1	Mouse	9541188
unspecific monooxygenase	1.14.14.1	Mouse	9698080

unspecific monooxygenase	1.14.14.1	Mouse	9703907
unspecific monooxygenase	1.14.14.1	Mouse	9788750
unspecific monooxygenase	1.14.14.1	Mouse	9797023
unspecific monooxygenase	1.14.14.1	Mouse	9804911
cholesterol monooxygenase (side-chain-cleaving)	1.14.15.6	Mouse	10418987
cholesterol monooxygenase (side-chain-cleaving)	1.14.15.6	Mouse	10700725
cholesterol monooxygenase (side-chain-cleaving)	1.14.15.6	Mouse	10729197
cholesterol monooxygenase (side-chain-cleaving)	1.14.15.6	Mouse	10964798
cholesterol monooxygenase (side-chain-cleaving)	1.14.15.6	Mouse	11191081
cholesterol monooxygenase (side-chain-cleaving)	1.14.15.6	Mouse	11297612
cholesterol monooxygenase (side-chain-cleaving)	1.14.15.6	Mouse	12137805
cholesterol monooxygenase (side-chain-cleaving)	1.14.15.6	Mouse	12596229
cholesterol monooxygenase (side-chain-cleaving)	1.14.15.6	Mouse	1282463
cholesterol monooxygenase (side-chain-cleaving)	1.14.15.6	Mouse	1327721
cholesterol monooxygenase (side-chain-cleaving)	1.14.15.6	Mouse	1337141
cholesterol monooxygenase (side-chain-cleaving)	1.14.15.6	Mouse	15026180
cholesterol monooxygenase (side-chain-cleaving)	1.14.15.6	Mouse	15159300
cholesterol monooxygenase (side-chain-cleaving)	1.14.15.6	Mouse	15205373
cholesterol monooxygenase (side-chain-cleaving)	1.14.15.6	Mouse	15231695
cholesterol monooxygenase (side-chain-cleaving)	1.14.15.6	Mouse	15344917
cholesterol monooxygenase (side-chain-cleaving)	1.14.15.6	Mouse	15635147
cholesterol monooxygenase (side-chain-cleaving)	1.14.15.6	Mouse	15666823
cholesterol monooxygenase (side-chain-cleaving)	1.14.15.6	Mouse	15804366

cholesterol monooxygenase (side-chain-cleaving)	1.14.15.6	Mouse	16139613
cholesterol monooxygenase (side-chain-cleaving)	1.14.15.6	Mouse	16410306
cholesterol monooxygenase (side-chain-cleaving)	1.14.15.6	Mouse	16541462
cholesterol monooxygenase (side-chain-cleaving)	1.14.15.6	Mouse	16551645
cholesterol monooxygenase (side-chain-cleaving)	1.14.15.6	Mouse	16632873
cholesterol monooxygenase (side-chain-cleaving)	1.14.15.6	Mouse	16780839
cholesterol monooxygenase (side-chain-cleaving)	1.14.15.6	Mouse	16999944
cholesterol monooxygenase (side-chain-cleaving)	1.14.15.6	Mouse	1700277
cholesterol monooxygenase (side-chain-cleaving)	1.14.15.6	Mouse	1874173
cholesterol monooxygenase (side-chain-cleaving)	1.14.15.6	Mouse	2226329
cholesterol monooxygenase (side-chain-cleaving)	1.14.15.6	Mouse	2555382
cholesterol monooxygenase (side-chain-cleaving)	1.14.15.6	Mouse	3027455
cholesterol monooxygenase (side-chain-cleaving)	1.14.15.6	Mouse	3356301
cholesterol monooxygenase (side-chain-cleaving)	1.14.15.6	Mouse	3502608
cholesterol monooxygenase (side-chain-cleaving)	1.14.15.6	Mouse	6249251
cholesterol monooxygenase (side-chain-cleaving)	1.14.15.6	Mouse	6256693
cholesterol monooxygenase (side-chain-cleaving)	1.14.15.6	Mouse	6546877
cholesterol monooxygenase (side-chain-cleaving)	1.14.15.6	Mouse	7594421
cholesterol monooxygenase (side-chain-cleaving)	1.14.15.6	Mouse	7664658
cholesterol monooxygenase (side-chain-cleaving)	1.14.15.6	Mouse	7669257
cholesterol monooxygenase (side-chain-cleaving)	1.14.15.6	Mouse	7826890

cholesterol monooxygenase (side-chain-cleaving)	1.14.15.6	Mouse	7882898
cholesterol monooxygenase (side-chain-cleaving)	1.14.15.6	Mouse	7956928
cholesterol monooxygenase (side-chain-cleaving)	1.14.15.6	Mouse	8026494
cholesterol monooxygenase (side-chain-cleaving)	1.14.15.6	Mouse	8152434
cholesterol monooxygenase (side-chain-cleaving)	1.14.15.6	Mouse	8278356
cholesterol monooxygenase (side-chain-cleaving)	1.14.15.6	Mouse	8425475
cholesterol monooxygenase (side-chain-cleaving)	1.14.15.6	Mouse	8432024
cholesterol monooxygenase (side-chain-cleaving)	1.14.15.6	Mouse	8547188
cholesterol monooxygenase (side-chain-cleaving)	1.14.15.6	Mouse	8584034
cholesterol monooxygenase (side-chain-cleaving)	1.14.15.6	Mouse	9013761
cholesterol monooxygenase (side-chain-cleaving)	1.14.15.6	Mouse	9326645
cholesterol monooxygenase (side-chain-cleaving)	1.14.15.6	Mouse	9511181
cholesterol monooxygenase (side-chain-cleaving)	1.14.15.6	Mouse	9622308
cholesterol monooxygenase (side-chain-cleaving)	1.14.15.6	Mouse	9635133
cholesterol monooxygenase (side-chain-cleaving)	1.14.15.6	Mouse	9888540
cholesterol monooxygenase (side-chain-cleaving)	1.14.15.6	Mouse	9922097
phenylalanine 4-monooxygenase	1.14.16.1	Mouse	10444341
phenylalanine 4-monooxygenase	1.14.16.1	Mouse	10800950
phenylalanine 4-monooxygenase	1.14.16.1	Mouse	10900078
phenylalanine 4-monooxygenase	1.14.16.1	Mouse	10984661
phenylalanine 4-monooxygenase	1.14.16.1	Mouse	15493924
phenylalanine 4-monooxygenase	1.14.16.1	Mouse	16139311
phenylalanine 4-monooxygenase	1.14.16.1	Mouse	16402341
phenylalanine 4-monooxygenase	1.14.16.1	Mouse	16429477
phenylalanine 4-monooxygenase	1.14.16.1	Mouse	3768311
phenylalanine 4-monooxygenase	1.14.16.1	Mouse	7635153
phenylalanine 4-monooxygenase	1.14.16.1	Mouse	7769265

phenylalanine 4-monooxygenase	1.14.16.1	Mouse	8214611
phenylalanine 4-monooxygenase	1.14.16.1	Mouse	8246172
phenylalanine 4-monooxygenase	1.14.16.1	Mouse	8502995
phenylalanine 4-monooxygenase	1.14.16.1	Mouse	8927234
phenylalanine 4-monooxygenase	1.14.16.1	Mouse	9168638
tyrosine 3-monooxygenase	1.14.16.2	Mouse	10320089
tyrosine 3-monooxygenase	1.14.16.2	Mouse	10476677
tyrosine 3-monooxygenase	1.14.16.2	Mouse	10725922
tyrosine 3-monooxygenase	1.14.16.2	Mouse	10970027
tyrosine 3-monooxygenase	1.14.16.2	Mouse	11424954
tyrosine 3-monooxygenase	1.14.16.2	Mouse	11517172
tyrosine 3-monooxygenase	1.14.16.2	Mouse	11948424
tyrosine 3-monooxygenase	1.14.16.2	Mouse	12124430
tyrosine 3-monooxygenase	1.14.16.2	Mouse	12640623
tyrosine 3-monooxygenase	1.14.16.2	Mouse	12717737
tyrosine 3-monooxygenase	1.14.16.2	Mouse	12891655
tyrosine 3-monooxygenase	1.14.16.2	Mouse	1383560
tyrosine 3-monooxygenase	1.14.16.2	Mouse	14675149
tyrosine 3-monooxygenase	1.14.16.2	Mouse	15447670
tyrosine 3-monooxygenase	1.14.16.2	Mouse	15649149
tyrosine 3-monooxygenase	1.14.16.2	Mouse	15683467
tyrosine 3-monooxygenase	1.14.16.2	Mouse	15897221
tyrosine 3-monooxygenase	1.14.16.2	Mouse	15935064
tyrosine 3-monooxygenase	1.14.16.2	Mouse	16049992
tyrosine 3-monooxygenase	1.14.16.2	Mouse	16052322
tyrosine 3-monooxygenase	1.14.16.2	Mouse	16080996
tyrosine 3-monooxygenase	1.14.16.2	Mouse	16187166
tyrosine 3-monooxygenase	1.14.16.2	Mouse	16199893
tyrosine 3-monooxygenase	1.14.16.2	Mouse	16650497
tyrosine 3-monooxygenase	1.14.16.2	Mouse	1847187
tyrosine 3-monooxygenase	1.14.16.2	Mouse	1972039
tyrosine 3-monooxygenase	1.14.16.2	Mouse	2573869
tyrosine 3-monooxygenase	1.14.16.2	Mouse	2883182
tyrosine 3-monooxygenase	1.14.16.2	Mouse	2902543
tyrosine 3-monooxygenase	1.14.16.2	Mouse	3267347
tyrosine 3-monooxygenase	1.14.16.2	Mouse	6150485
tyrosine 3-monooxygenase	1.14.16.2	Mouse	7494448
tyrosine 3-monooxygenase	1.14.16.2	Mouse	7576974
tyrosine 3-monooxygenase	1.14.16.2	Mouse	7719703
tyrosine 3-monooxygenase	1.14.16.2	Mouse	8723206
tyrosine 3-monooxygenase	1.14.16.2	Mouse	8984738
tyrosine 3-monooxygenase	1.14.16.2	Mouse	9247090
tyrosine 3-monooxygenase	1.14.16.2	Mouse	9350044

tyrosine 3-monooxygenase	1.14.16.2	Mouse	9719456
tyrosine 3-monooxygenase	1.14.16.2	Mouse	9822156
tyrosine 3-monooxygenase	1.14.16.2	Mouse	9914719
tryptophan 5-monooxygenase	1.14.16.4	Mouse	10327914
tryptophan 5-monooxygenase	1.14.16.4	Mouse	10483053
tryptophan 5-monooxygenase	1.14.16.4	Mouse	10514446
tryptophan 5-monooxygenase	1.14.16.4	Mouse	10581400
tryptophan 5-monooxygenase	1.14.16.4	Mouse	1059145
tryptophan 5-monooxygenase	1.14.16.4	Mouse	10636468
tryptophan 5-monooxygenase	1.14.16.4	Mouse	10676966
tryptophan 5-monooxygenase	1.14.16.4	Mouse	10715363
tryptophan 5-monooxygenase	1.14.16.4	Mouse	10721034
tryptophan 5-monooxygenase	1.14.16.4	Mouse	10800950
tryptophan 5-monooxygenase	1.14.16.4	Mouse	10899755
tryptophan 5-monooxygenase	1.14.16.4	Mouse	10900076
tryptophan 5-monooxygenase	1.14.16.4	Mouse	10900078
tryptophan 5-monooxygenase	1.14.16.4	Mouse	10909123
tryptophan 5-monooxygenase	1.14.16.4	Mouse	10950846
tryptophan 5-monooxygenase	1.14.16.4	Mouse	10953292
tryptophan 5-monooxygenase	1.14.16.4	Mouse	10993738
tryptophan 5-monooxygenase	1.14.16.4	Mouse	11085312
tryptophan 5-monooxygenase	1.14.16.4	Mouse	11113315
tryptophan 5-monooxygenase	1.14.16.4	Mouse	11121198
tryptophan 5-monooxygenase	1.14.16.4	Mouse	11326294
tryptophan 5-monooxygenase	1.14.16.4	Mouse	11386854
tryptophan 5-monooxygenase	1.14.16.4	Mouse	11426508
tryptophan 5-monooxygenase	1.14.16.4	Mouse	11747434
tryptophan 5-monooxygenase	1.14.16.4	Mouse	12015221
tryptophan 5-monooxygenase	1.14.16.4	Mouse	12116193
tryptophan 5-monooxygenase	1.14.16.4	Mouse	12399958
tryptophan 5-monooxygenase	1.14.16.4	Mouse	12507391
tryptophan 5-monooxygenase	1.14.16.4	Mouse	12818363
tryptophan 5-monooxygenase	1.14.16.4	Mouse	12915291
tryptophan 5-monooxygenase	1.14.16.4	Mouse	14563478
tryptophan 5-monooxygenase	1.14.16.4	Mouse	14960297
tryptophan 5-monooxygenase	1.14.16.4	Mouse	15124006
tryptophan 5-monooxygenase	1.14.16.4	Mouse	15163437
tryptophan 5-monooxygenase	1.14.16.4	Mouse	15281067
tryptophan 5-monooxygenase	1.14.16.4	Mouse	15476687
tryptophan 5-monooxygenase	1.14.16.4	Mouse	15663479
tryptophan 5-monooxygenase	1.14.16.4	Mouse	15677682
tryptophan 5-monooxygenase	1.14.16.4	Mouse	15804496
tryptophan 5-monooxygenase	1.14.16.4	Mouse	15940290

tryptophan 5-monooxygenase	1.14.16.4	Mouse	15968084
tryptophan 5-monooxygenase	1.14.16.4	Mouse	16023677
tryptophan 5-monooxygenase	1.14.16.4	Mouse	16109589
tryptophan 5-monooxygenase	1.14.16.4	Mouse	16165107
tryptophan 5-monooxygenase	1.14.16.4	Mouse	16192985
tryptophan 5-monooxygenase	1.14.16.4	Mouse	16198203
tryptophan 5-monooxygenase	1.14.16.4	Mouse	16203956
tryptophan 5-monooxygenase	1.14.16.4	Mouse	16314762
tryptophan 5-monooxygenase	1.14.16.4	Mouse	16381305
tryptophan 5-monooxygenase	1.14.16.4	Mouse	16405505
tryptophan 5-monooxygenase	1.14.16.4	Mouse	16407550
tryptophan 5-monooxygenase	1.14.16.4	Mouse	16436194
tryptophan 5-monooxygenase	1.14.16.4	Mouse	16467214
tryptophan 5-monooxygenase	1.14.16.4	Mouse	16495936
tryptophan 5-monooxygenase	1.14.16.4	Mouse	16498236
tryptophan 5-monooxygenase	1.14.16.4	Mouse	16581041
tryptophan 5-monooxygenase	1.14.16.4	Mouse	16615082
tryptophan 5-monooxygenase	1.14.16.4	Mouse	16806098
tryptophan 5-monooxygenase	1.14.16.4	Mouse	16864580
tryptophan 5-monooxygenase	1.14.16.4	Mouse	16924415
tryptophan 5-monooxygenase	1.14.16.4	Mouse	16979275
tryptophan 5-monooxygenase	1.14.16.4	Mouse	17015812
tryptophan 5-monooxygenase	1.14.16.4	Mouse	1707735
tryptophan 5-monooxygenase	1.14.16.4	Mouse	1997008
tryptophan 5-monooxygenase	1.14.16.4	Mouse	2466525
tryptophan 5-monooxygenase	1.14.16.4	Mouse	2568404
tryptophan 5-monooxygenase	1.14.16.4	Mouse	2737466
tryptophan 5-monooxygenase	1.14.16.4	Mouse	3379411
tryptophan 5-monooxygenase	1.14.16.4	Mouse	3742199
tryptophan 5-monooxygenase	1.14.16.4	Mouse	3996672
tryptophan 5-monooxygenase	1.14.16.4	Mouse	7552299
tryptophan 5-monooxygenase	1.14.16.4	Mouse	761167
tryptophan 5-monooxygenase	1.14.16.4	Mouse	8015380
tryptophan 5-monooxygenase	1.14.16.4	Mouse	8255926
tryptophan 5-monooxygenase	1.14.16.4	Mouse	8527368
tryptophan 5-monooxygenase	1.14.16.4	Mouse	8726569
tryptophan 5-monooxygenase	1.14.16.4	Mouse	8752114
tryptophan 5-monooxygenase	1.14.16.4	Mouse	9084419
tryptophan 5-monooxygenase	1.14.16.4	Mouse	9109552
tryptophan 5-monooxygenase	1.14.16.4	Mouse	9244768
tryptophan 5-monooxygenase	1.14.16.4	Mouse	9295371
tryptophan 5-monooxygenase	1.14.16.4	Mouse	9326303
tryptophan 5-monooxygenase	1.14.16.4	Mouse	9392522

tryptophan 5-monooxygenase	1.14.16.4	Mouse	9603613
tryptophan 5-monooxygenase	1.14.16.4	Mouse	9630672
tryptophan 5-monooxygenase	1.14.16.4	Mouse	9672049
tryptophan 5-monooxygenase	1.14.16.4	Mouse	9770640
tryptophan 5-monooxygenase	1.14.16.4	Mouse	9886051
monophenol monooxygenase	1.14.18.1	Mouse	10102625
monophenol monooxygenase	1.14.18.1	Mouse	10495428
monophenol monooxygenase	1.14.18.1	Mouse	10671066
monophenol monooxygenase	1.14.18.1	Mouse	10691963
monophenol monooxygenase	1.14.18.1	Mouse	10839460
monophenol monooxygenase	1.14.18.1	Mouse	10870514
monophenol monooxygenase	1.14.18.1	Mouse	10956040
monophenol monooxygenase	1.14.18.1	Mouse	10960773
monophenol monooxygenase	1.14.18.1	Mouse	10966567
monophenol monooxygenase	1.14.18.1	Mouse	10998146
monophenol monooxygenase	1.14.18.1	Mouse	11076759
monophenol monooxygenase	1.14.18.1	Mouse	11139343
monophenol monooxygenase	1.14.18.1	Mouse	11167218
monophenol monooxygenase	1.14.18.1	Mouse	11171088
monophenol monooxygenase	1.14.18.1	Mouse	11178974
monophenol monooxygenase	1.14.18.1	Mouse	11180971
monophenol monooxygenase	1.14.18.1	Mouse	11236829
monophenol monooxygenase	1.14.18.1	Mouse	11277411
monophenol monooxygenase	1.14.18.1	Mouse	11493672
monophenol monooxygenase	1.14.18.1	Mouse	11574907
monophenol monooxygenase	1.14.18.1	Mouse	11601654
monophenol monooxygenase	1.14.18.1	Mouse	11602344
monophenol monooxygenase	1.14.18.1	Mouse	11673476
monophenol monooxygenase	1.14.18.1	Mouse	11701975
monophenol monooxygenase	1.14.18.1	Mouse	11799132
monophenol monooxygenase	1.14.18.1	Mouse	11821691
monophenol monooxygenase	1.14.18.1	Mouse	11834083
monophenol monooxygenase	1.14.18.1	Mouse	11851885
monophenol monooxygenase	1.14.18.1	Mouse	11858948
monophenol monooxygenase	1.14.18.1	Mouse	12069489
monophenol monooxygenase	1.14.18.1	Mouse	12201672
monophenol monooxygenase	1.14.18.1	Mouse	12565907
monophenol monooxygenase	1.14.18.1	Mouse	12595535
monophenol monooxygenase	1.14.18.1	Mouse	12950726
monophenol monooxygenase	1.14.18.1	Mouse	14511124
monophenol monooxygenase	1.14.18.1	Mouse	14597395
monophenol monooxygenase	1.14.18.1	Mouse	14615486
monophenol monooxygenase	1.14.18.1	Mouse	14622170

monophenol monooxygenase	1.14.18.1	Mouse	14699163
monophenol monooxygenase	1.14.18.1	Mouse	14726002
monophenol monooxygenase	1.14.18.1	Mouse	15016305
monophenol monooxygenase	1.14.18.1	Mouse	15067002
monophenol monooxygenase	1.14.18.1	Mouse	15089040
monophenol monooxygenase	1.14.18.1	Mouse	15147727
monophenol monooxygenase	1.14.18.1	Mouse	15154289
monophenol monooxygenase	1.14.18.1	Mouse	15250942
monophenol monooxygenase	1.14.18.1	Mouse	15252133
monophenol monooxygenase	1.14.18.1	Mouse	15381243
monophenol monooxygenase	1.14.18.1	Mouse	15520878
monophenol monooxygenase	1.14.18.1	Mouse	15572362
monophenol monooxygenase	1.14.18.1	Mouse	15684492
monophenol monooxygenase	1.14.18.1	Mouse	15748887
monophenol monooxygenase	1.14.18.1	Mouse	15760340
monophenol monooxygenase	1.14.18.1	Mouse	15760341
monophenol monooxygenase	1.14.18.1	Mouse	15895362
monophenol monooxygenase	1.14.18.1	Mouse	16146766
monophenol monooxygenase	1.14.18.1	Mouse	16280010
monophenol monooxygenase	1.14.18.1	Mouse	16293777
monophenol monooxygenase	1.14.18.1	Mouse	16327152
monophenol monooxygenase	1.14.18.1	Mouse	16335789
monophenol monooxygenase	1.14.18.1	Mouse	16456529
monophenol monooxygenase	1.14.18.1	Mouse	16477373
monophenol monooxygenase	1.14.18.1	Mouse	16524430
monophenol monooxygenase	1.14.18.1	Mouse	16579986
monophenol monooxygenase	1.14.18.1	Mouse	16691509
monophenol monooxygenase	1.14.18.1	Mouse	16704452
monophenol monooxygenase	1.14.18.1	Mouse	1671405
monophenol monooxygenase	1.14.18.1	Mouse	16757562
monophenol monooxygenase	1.14.18.1	Mouse	16847266
monophenol monooxygenase	1.14.18.1	Mouse	17083330
monophenol monooxygenase	1.14.18.1	Mouse	17083484
monophenol monooxygenase	1.14.18.1	Mouse	1720016
monophenol monooxygenase	1.14.18.1	Mouse	1739407
monophenol monooxygenase	1.14.18.1	Mouse	1902569
monophenol monooxygenase	1.14.18.1	Mouse	1903356
monophenol monooxygenase	1.14.18.1	Mouse	2118105
monophenol monooxygenase	1.14.18.1	Mouse	2497447
monophenol monooxygenase	1.14.18.1	Mouse	2497448
monophenol monooxygenase	1.14.18.1	Mouse	2498458
monophenol monooxygenase	1.14.18.1	Mouse	2543086
monophenol monooxygenase	1.14.18.1	Mouse	2720797

monophenol monooxygenase	1.14.18.1	Mouse	2983883
monophenol monooxygenase	1.14.18.1	Mouse	2992767
monophenol monooxygenase	1.14.18.1	Mouse	3121644
monophenol monooxygenase	1.14.18.1	Mouse	3127037
monophenol monooxygenase	1.14.18.1	Mouse	3143738
monophenol monooxygenase	1.14.18.1	Mouse	3148919
monophenol monooxygenase	1.14.18.1	Mouse	3927896
monophenol monooxygenase	1.14.18.1	Mouse	6826662
monophenol monooxygenase	1.14.18.1	Mouse	7646526
monophenol monooxygenase	1.14.18.1	Mouse	7685020
monophenol monooxygenase	1.14.18.1	Mouse	7775602
monophenol monooxygenase	1.14.18.1	Mouse	7792254
monophenol monooxygenase	1.14.18.1	Mouse	7811291
monophenol monooxygenase	1.14.18.1	Mouse	7821799
monophenol monooxygenase	1.14.18.1	Mouse	7969144
monophenol monooxygenase	1.14.18.1	Mouse	8039502
monophenol monooxygenase	1.14.18.1	Mouse	8124097
monophenol monooxygenase	1.14.18.1	Mouse	8136316
monophenol monooxygenase	1.14.18.1	Mouse	8138743
monophenol monooxygenase	1.14.18.1	Mouse	8151127
monophenol monooxygenase	1.14.18.1	Mouse	8197131
monophenol monooxygenase	1.14.18.1	Mouse	8270948
monophenol monooxygenase	1.14.18.1	Mouse	8292889
monophenol monooxygenase	1.14.18.1	Mouse	8332936
monophenol monooxygenase	1.14.18.1	Mouse	8433000
monophenol monooxygenase	1.14.18.1	Mouse	8462726
monophenol monooxygenase	1.14.18.1	Mouse	8496620
monophenol monooxygenase	1.14.18.1	Mouse	8577719
monophenol monooxygenase	1.14.18.1	Mouse	8577860
monophenol monooxygenase	1.14.18.1	Mouse	8806453
monophenol monooxygenase	1.14.18.1	Mouse	8861753
monophenol monooxygenase	1.14.18.1	Mouse	9029814
monophenol monooxygenase	1.14.18.1	Mouse	9056640
monophenol monooxygenase	1.14.18.1	Mouse	9088570
monophenol monooxygenase	1.14.18.1	Mouse	9177196
monophenol monooxygenase	1.14.18.1	Mouse	9240530
monophenol monooxygenase	1.14.18.1	Mouse	9417870
monophenol monooxygenase	1.14.18.1	Mouse	9447965
monophenol monooxygenase	1.14.18.1	Mouse	9475178
monophenol monooxygenase	1.14.18.1	Mouse	9500554
monophenol monooxygenase	1.14.18.1	Mouse	9500997
monophenol monooxygenase	1.14.18.1	Mouse	9545341
monophenol monooxygenase	1.14.18.1	Mouse	9593634

monophenol monooxygenase	1.14.18.1	Mouse	9719456
monophenol monooxygenase	1.14.18.1	Mouse	9736634
monophenol monooxygenase	1.14.18.1	Mouse	9788743
monophenol monooxygenase	1.14.18.1	Mouse	9822646
monophenol monooxygenase	1.14.18.1	Mouse	9830058
monophenol monooxygenase	1.14.18.1	Mouse	9853616
monophenol monooxygenase	1.14.18.1	Mouse	9880801
CMP-N-acetylneuraminate monooxygenase	1.14.18.2	Mouse	10320348
CMP-N-acetylneuraminate monooxygenase	1.14.18.2	Mouse	11786991
CMP-N-acetylneuraminate monooxygenase	1.14.18.2	Mouse	8132639
CMP-N-acetylneuraminate monooxygenase	1.14.18.2	Mouse	8195125
stearoyl-CoA 9-desaturase	1.14.19.1	Mouse	10484602
stearoyl-CoA 9-desaturase	1.14.19.1	Mouse	10581155
stearoyl-CoA 9-desaturase	1.14.19.1	Mouse	11352637
stearoyl-CoA 9-desaturase	1.14.19.1	Mouse	11396956
stearoyl-CoA 9-desaturase	1.14.19.1	Mouse	11415448
stearoyl-CoA 9-desaturase	1.14.19.1	Mouse	11533264
stearoyl-CoA 9-desaturase	1.14.19.1	Mouse	11677241
stearoyl-CoA 9-desaturase	1.14.19.1	Mouse	12364560
stearoyl-CoA 9-desaturase	1.14.19.1	Mouse	12815040
stearoyl-CoA 9-desaturase	1.14.19.1	Mouse	14610276
stearoyl-CoA 9-desaturase	1.14.19.1	Mouse	14654089
stearoyl-CoA 9-desaturase	1.14.19.1	Mouse	14734557
stearoyl-CoA 9-desaturase	1.14.19.1	Mouse	15096593
stearoyl-CoA 9-desaturase	1.14.19.1	Mouse	15180999
stearoyl-CoA 9-desaturase	1.14.19.1	Mouse	15556291
stearoyl-CoA 9-desaturase	1.14.19.1	Mouse	15726820
stearoyl-CoA 9-desaturase	1.14.19.1	Mouse	15761499
stearoyl-CoA 9-desaturase	1.14.19.1	Mouse	15829484
stearoyl-CoA 9-desaturase	1.14.19.1	Mouse	15836467
stearoyl-CoA 9-desaturase	1.14.19.1	Mouse	16245372
stearoyl-CoA 9-desaturase	1.14.19.1	Mouse	16767221
stearoyl-CoA 9-desaturase	1.14.19.1	Mouse	16804073
stearoyl-CoA 9-desaturase	1.14.19.1	Mouse	9272444
prostaglandin-endoperoxide synthase	1.14.99.1	Mouse	10024686
prostaglandin-endoperoxide synthase	1.14.99.1	Mouse	10091284
prostaglandin-endoperoxide synthase	1.14.99.1	Mouse	10226539
prostaglandin-endoperoxide synthase	1.14.99.1	Mouse	10228831
prostaglandin-endoperoxide synthase	1.14.99.1	Mouse	10365086
prostaglandin-endoperoxide synthase	1.14.99.1	Mouse	10395693
prostaglandin-endoperoxide synthase	1.14.99.1	Mouse	10399316
prostaglandin-endoperoxide synthase	1.14.99.1	Mouse	10425287
prostaglandin-endoperoxide synthase	1.14.99.1	Mouse	10426495

prostaglandin-endoperoxide synthase	1.14.99.1	Mouse	10560661
prostaglandin-endoperoxide synthase	1.14.99.1	Mouse	10652449
prostaglandin-endoperoxide synthase	1.14.99.1	Mouse	10667579
prostaglandin-endoperoxide synthase	1.14.99.1	Mouse	10754263
prostaglandin-endoperoxide synthase	1.14.99.1	Mouse	10807888
prostaglandin-endoperoxide synthase	1.14.99.1	Mouse	10859338
prostaglandin-endoperoxide synthase	1.14.99.1	Mouse	10935540
prostaglandin-endoperoxide synthase	1.14.99.1	Mouse	10991924
prostaglandin-endoperoxide synthase	1.14.99.1	Mouse	10999846
prostaglandin-endoperoxide synthase	1.14.99.1	Mouse	11153163
prostaglandin-endoperoxide synthase	1.14.99.1	Mouse	11192946
prostaglandin-endoperoxide synthase	1.14.99.1	Mouse	11220737
prostaglandin-endoperoxide synthase	1.14.99.1	Mouse	11239825
prostaglandin-endoperoxide synthase	1.14.99.1	Mouse	11303759
prostaglandin-endoperoxide synthase	1.14.99.1	Mouse	11338298
prostaglandin-endoperoxide synthase	1.14.99.1	Mouse	11338376
prostaglandin-endoperoxide synthase	1.14.99.1	Mouse	11407522
prostaglandin-endoperoxide synthase	1.14.99.1	Mouse	11427038
prostaglandin-endoperoxide synthase	1.14.99.1	Mouse	11451994
prostaglandin-endoperoxide synthase	1.14.99.1	Mouse	11455568
prostaglandin-endoperoxide synthase	1.14.99.1	Mouse	11501838
prostaglandin-endoperoxide synthase	1.14.99.1	Mouse	11506967
prostaglandin-endoperoxide synthase	1.14.99.1	Mouse	11519793
prostaglandin-endoperoxide synthase	1.14.99.1	Mouse	11520057
prostaglandin-endoperoxide synthase	1.14.99.1	Mouse	11588192
prostaglandin-endoperoxide synthase	1.14.99.1	Mouse	11673276
prostaglandin-endoperoxide synthase	1.14.99.1	Mouse	11746474
prostaglandin-endoperoxide synthase	1.14.99.1	Mouse	11758826
prostaglandin-endoperoxide synthase	1.14.99.1	Mouse	11781662
prostaglandin-endoperoxide synthase	1.14.99.1	Mouse	11857443
prostaglandin-endoperoxide synthase	1.14.99.1	Mouse	11870370
prostaglandin-endoperoxide synthase	1.14.99.1	Mouse	11945149
prostaglandin-endoperoxide synthase	1.14.99.1	Mouse	11966528
prostaglandin-endoperoxide synthase	1.14.99.1	Mouse	11991685
prostaglandin-endoperoxide synthase	1.14.99.1	Mouse	12021045
prostaglandin-endoperoxide synthase	1.14.99.1	Mouse	12034746
prostaglandin-endoperoxide synthase	1.14.99.1	Mouse	12047931
prostaglandin-endoperoxide synthase	1.14.99.1	Mouse	12211105
prostaglandin-endoperoxide synthase	1.14.99.1	Mouse	12242329
prostaglandin-endoperoxide synthase	1.14.99.1	Mouse	12349897
prostaglandin-endoperoxide synthase	1.14.99.1	Mouse	12352459
prostaglandin-endoperoxide synthase	1.14.99.1	Mouse	12377741
prostaglandin-endoperoxide synthase	1.14.99.1	Mouse	12397176

prostaglandin-endoperoxide synthase	1.14.99.1	Mouse	12414874
prostaglandin-endoperoxide synthase	1.14.99.1	Mouse	12432912
prostaglandin-endoperoxide synthase	1.14.99.1	Mouse	12467525
prostaglandin-endoperoxide synthase	1.14.99.1	Mouse	12468643
prostaglandin-endoperoxide synthase	1.14.99.1	Mouse	12491803
prostaglandin-endoperoxide synthase	1.14.99.1	Mouse	12502903
prostaglandin-endoperoxide synthase	1.14.99.1	Mouse	12576462
prostaglandin-endoperoxide synthase	1.14.99.1	Mouse	12604670
prostaglandin-endoperoxide synthase	1.14.99.1	Mouse	12614262
prostaglandin-endoperoxide synthase	1.14.99.1	Mouse	12669881
prostaglandin-endoperoxide synthase	1.14.99.1	Mouse	12683423
prostaglandin-endoperoxide synthase	1.14.99.1	Mouse	12735864
prostaglandin-endoperoxide synthase	1.14.99.1	Mouse	12845600
prostaglandin-endoperoxide synthase	1.14.99.1	Mouse	12846004
prostaglandin-endoperoxide synthase	1.14.99.1	Mouse	12885872
prostaglandin-endoperoxide synthase	1.14.99.1	Mouse	12969240
prostaglandin-endoperoxide synthase	1.14.99.1	Mouse	12970891
prostaglandin-endoperoxide synthase	1.14.99.1	Mouse	1305681
prostaglandin-endoperoxide synthase	1.14.99.1	Mouse	1316915
prostaglandin-endoperoxide synthase	1.14.99.1	Mouse	1400321
prostaglandin-endoperoxide synthase	1.14.99.1	Mouse	1410519
prostaglandin-endoperoxide synthase	1.14.99.1	Mouse	14518562
prostaglandin-endoperoxide synthase	1.14.99.1	Mouse	14566678
prostaglandin-endoperoxide synthase	1.14.99.1	Mouse	14634339
prostaglandin-endoperoxide synthase	1.14.99.1	Mouse	14704742
prostaglandin-endoperoxide synthase	1.14.99.1	Mouse	14707443
prostaglandin-endoperoxide synthase	1.14.99.1	Mouse	14743889
prostaglandin-endoperoxide synthase	1.14.99.1	Mouse	1480760
prostaglandin-endoperoxide synthase	1.14.99.1	Mouse	14965326
prostaglandin-endoperoxide synthase	1.14.99.1	Mouse	15037210
prostaglandin-endoperoxide synthase	1.14.99.1	Mouse	15106811
prostaglandin-endoperoxide synthase	1.14.99.1	Mouse	15109739
prostaglandin-endoperoxide synthase	1.14.99.1	Mouse	15148333
prostaglandin-endoperoxide synthase	1.14.99.1	Mouse	15205395
prostaglandin-endoperoxide synthase	1.14.99.1	Mouse	15299086
prostaglandin-endoperoxide synthase	1.14.99.1	Mouse	15351039
prostaglandin-endoperoxide synthase	1.14.99.1	Mouse	15486039
prostaglandin-endoperoxide synthase	1.14.99.1	Mouse	15496409
prostaglandin-endoperoxide synthase	1.14.99.1	Mouse	15507510
prostaglandin-endoperoxide synthase	1.14.99.1	Mouse	15596243
prostaglandin-endoperoxide synthase	1.14.99.1	Mouse	15604817
prostaglandin-endoperoxide synthase	1.14.99.1	Mouse	15639337
prostaglandin-endoperoxide synthase	1.14.99.1	Mouse	15653788

prostaglandin-endoperoxide synthase	1.14.99.1	Mouse	15668527
prostaglandin-endoperoxide synthase	1.14.99.1	Mouse	15753380
prostaglandin-endoperoxide synthase	1.14.99.1	Mouse	15809059
prostaglandin-endoperoxide synthase	1.14.99.1	Mouse	15821352
prostaglandin-endoperoxide synthase	1.14.99.1	Mouse	15885672
prostaglandin-endoperoxide synthase	1.14.99.1	Mouse	15889237
prostaglandin-endoperoxide synthase	1.14.99.1	Mouse	15900018
prostaglandin-endoperoxide synthase	1.14.99.1	Mouse	15992053
prostaglandin-endoperoxide synthase	1.14.99.1	Mouse	15993594
prostaglandin-endoperoxide synthase	1.14.99.1	Mouse	16046792
prostaglandin-endoperoxide synthase	1.14.99.1	Mouse	16137646
prostaglandin-endoperoxide synthase	1.14.99.1	Mouse	16169091
prostaglandin-endoperoxide synthase	1.14.99.1	Mouse	16169124
prostaglandin-endoperoxide synthase	1.14.99.1	Mouse	16253229
prostaglandin-endoperoxide synthase	1.14.99.1	Mouse	16284883
prostaglandin-endoperoxide synthase	1.14.99.1	Mouse	16445867
prostaglandin-endoperoxide synthase	1.14.99.1	Mouse	16458279
prostaglandin-endoperoxide synthase	1.14.99.1	Mouse	16493584
prostaglandin-endoperoxide synthase	1.14.99.1	Mouse	16516846
prostaglandin-endoperoxide synthase	1.14.99.1	Mouse	16529558
prostaglandin-endoperoxide synthase	1.14.99.1	Mouse	16709833
prostaglandin-endoperoxide synthase	1.14.99.1	Mouse	16716827
prostaglandin-endoperoxide synthase	1.14.99.1	Mouse	16740978
prostaglandin-endoperoxide synthase	1.14.99.1	Mouse	16753269
prostaglandin-endoperoxide synthase	1.14.99.1	Mouse	16827136
prostaglandin-endoperoxide synthase	1.14.99.1	Mouse	16840740
prostaglandin-endoperoxide synthase	1.14.99.1	Mouse	16842132
prostaglandin-endoperoxide synthase	1.14.99.1	Mouse	16842185
prostaglandin-endoperoxide synthase	1.14.99.1	Mouse	16847764
prostaglandin-endoperoxide synthase	1.14.99.1	Mouse	16870271
prostaglandin-endoperoxide synthase	1.14.99.1	Mouse	16871410
prostaglandin-endoperoxide synthase	1.14.99.1	Mouse	16872525
prostaglandin-endoperoxide synthase	1.14.99.1	Mouse	16885386
prostaglandin-endoperoxide synthase	1.14.99.1	Mouse	1694171
prostaglandin-endoperoxide synthase	1.14.99.1	Mouse	16966442
prostaglandin-endoperoxide synthase	1.14.99.1	Mouse	1706264
prostaglandin-endoperoxide synthase	1.14.99.1	Mouse	17066444
prostaglandin-endoperoxide synthase	1.14.99.1	Mouse	17082565
prostaglandin-endoperoxide synthase	1.14.99.1	Mouse	17140386
prostaglandin-endoperoxide synthase	1.14.99.1	Mouse	1721064
prostaglandin-endoperoxide synthase	1.14.99.1	Mouse	1730539
prostaglandin-endoperoxide synthase	1.14.99.1	Mouse	1731622
prostaglandin-endoperoxide synthase	1.14.99.1	Mouse	1744122

prostaglandin-endoperoxide synthase	1.14.99.1	Mouse	1836009
prostaglandin-endoperoxide synthase	1.14.99.1	Mouse	1903222
prostaglandin-endoperoxide synthase	1.14.99.1	Mouse	2125559
prostaglandin-endoperoxide synthase	1.14.99.1	Mouse	2150736
prostaglandin-endoperoxide synthase	1.14.99.1	Mouse	2497108
prostaglandin-endoperoxide synthase	1.14.99.1	Mouse	2519899
prostaglandin-endoperoxide synthase	1.14.99.1	Mouse	26389
prostaglandin-endoperoxide synthase	1.14.99.1	Mouse	2770427
prostaglandin-endoperoxide synthase	1.14.99.1	Mouse	3040660
prostaglandin-endoperoxide synthase	1.14.99.1	Mouse	3510842
prostaglandin-endoperoxide synthase	1.14.99.1	Mouse	6197028
prostaglandin-endoperoxide synthase	1.14.99.1	Mouse	6252692
prostaglandin-endoperoxide synthase	1.14.99.1	Mouse	6312489
prostaglandin-endoperoxide synthase	1.14.99.1	Mouse	6324240
prostaglandin-endoperoxide synthase	1.14.99.1	Mouse	663276
prostaglandin-endoperoxide synthase	1.14.99.1	Mouse	7229430
prostaglandin-endoperoxide synthase	1.14.99.1	Mouse	7635765
prostaglandin-endoperoxide synthase	1.14.99.1	Mouse	7706373
prostaglandin-endoperoxide synthase	1.14.99.1	Mouse	7775455
prostaglandin-endoperoxide synthase	1.14.99.1	Mouse	7784470
prostaglandin-endoperoxide synthase	1.14.99.1	Mouse	7920026
prostaglandin-endoperoxide synthase	1.14.99.1	Mouse	7926468
prostaglandin-endoperoxide synthase	1.14.99.1	Mouse	7980528
prostaglandin-endoperoxide synthase	1.14.99.1	Mouse	8132578
prostaglandin-endoperoxide synthase	1.14.99.1	Mouse	8163473
prostaglandin-endoperoxide synthase	1.14.99.1	Mouse	8188473
prostaglandin-endoperoxide synthase	1.14.99.1	Mouse	8261019
prostaglandin-endoperoxide synthase	1.14.99.1	Mouse	8280164
prostaglandin-endoperoxide synthase	1.14.99.1	Mouse	8365485
prostaglandin-endoperoxide synthase	1.14.99.1	Mouse	8521479
prostaglandin-endoperoxide synthase	1.14.99.1	Mouse	8616915
prostaglandin-endoperoxide synthase	1.14.99.1	Mouse	8641206
prostaglandin-endoperoxide synthase	1.14.99.1	Mouse	8653697
prostaglandin-endoperoxide synthase	1.14.99.1	Mouse	8752493
prostaglandin-endoperoxide synthase	1.14.99.1	Mouse	8918053
prostaglandin-endoperoxide synthase	1.14.99.1	Mouse	9013627
prostaglandin-endoperoxide synthase	1.14.99.1	Mouse	9057838
prostaglandin-endoperoxide synthase	1.14.99.1	Mouse	9096394
prostaglandin-endoperoxide synthase	1.14.99.1	Mouse	9102213
prostaglandin-endoperoxide synthase	1.14.99.1	Mouse	9126611
prostaglandin-endoperoxide synthase	1.14.99.1	Mouse	9144511
prostaglandin-endoperoxide synthase	1.14.99.1	Mouse	9177102
prostaglandin-endoperoxide synthase	1.14.99.1	Mouse	9177247

prostaglandin-endoperoxide synthase	1.14.99.1	Mouse	9261162
prostaglandin-endoperoxide synthase	1.14.99.1	Mouse	9360765
prostaglandin-endoperoxide synthase	1.14.99.1	Mouse	9373878
prostaglandin-endoperoxide synthase	1.14.99.1	Mouse	9383435
prostaglandin-endoperoxide synthase	1.14.99.1	Mouse	9402302
prostaglandin-endoperoxide synthase	1.14.99.1	Mouse	9458802
prostaglandin-endoperoxide synthase	1.14.99.1	Mouse	9483916
prostaglandin-endoperoxide synthase	1.14.99.1	Mouse	9492015
prostaglandin-endoperoxide synthase	1.14.99.1	Mouse	9513902
prostaglandin-endoperoxide synthase	1.14.99.1	Mouse	9517759
prostaglandin-endoperoxide synthase	1.14.99.1	Mouse	9521170
prostaglandin-endoperoxide synthase	1.14.99.1	Mouse	9526843
prostaglandin-endoperoxide synthase	1.14.99.1	Mouse	9562240
prostaglandin-endoperoxide synthase	1.14.99.1	Mouse	9568703
prostaglandin-endoperoxide synthase	1.14.99.1	Mouse	9585093
prostaglandin-endoperoxide synthase	1.14.99.1	Mouse	9649571
prostaglandin-endoperoxide synthase	1.14.99.1	Mouse	97337
prostaglandin-endoperoxide synthase	1.14.99.1	Mouse	9740394
prostaglandin-endoperoxide synthase	1.14.99.1	Mouse	9758208
prostaglandin-endoperoxide synthase	1.14.99.1	Mouse	9764845
prostaglandin-endoperoxide synthase	1.14.99.1	Mouse	9790545
prostaglandin-endoperoxide synthase	1.14.99.1	Mouse	9811310
prostaglandin-endoperoxide synthase	1.14.99.1	Mouse	9823297
prostaglandin-endoperoxide synthase	1.14.99.1	Mouse	9870924
prostaglandin-endoperoxide synthase	1.14.99.1	Mouse	9879525
heme oxygenase	1.14.99.3	Mouse	10349844
heme oxygenase	1.14.99.3	Mouse	10353745
heme oxygenase	1.14.99.3	Mouse	10630670
heme oxygenase	1.14.99.3	Mouse	10634305
heme oxygenase	1.14.99.3	Mouse	10644516
heme oxygenase	1.14.99.3	Mouse	10692503
heme oxygenase	1.14.99.3	Mouse	10727990
heme oxygenase	1.14.99.3	Mouse	10793064
heme oxygenase	1.14.99.3	Mouse	10814519
heme oxygenase	1.14.99.3	Mouse	10961657
heme oxygenase	1.14.99.3	Mouse	11007950
heme oxygenase	1.14.99.3	Mouse	11135063
heme oxygenase	1.14.99.3	Mouse	11258550
heme oxygenase	1.14.99.3	Mouse	11498995
heme oxygenase	1.14.99.3	Mouse	11591199
heme oxygenase	1.14.99.3	Mouse	11592943
heme oxygenase	1.14.99.3	Mouse	11698254
heme oxygenase	1.14.99.3	Mouse	11705458

heme oxygenase	1.14.99.3	Mouse	11758836
heme oxygenase	1.14.99.3	Mouse	11801258
heme oxygenase	1.14.99.3	Mouse	11803410
heme oxygenase	1.14.99.3	Mouse	11868390
heme oxygenase	1.14.99.3	Mouse	11978880
heme oxygenase	1.14.99.3	Mouse	12042070
heme oxygenase	1.14.99.3	Mouse	12057765
heme oxygenase	1.14.99.3	Mouse	12117910
heme oxygenase	1.14.99.3	Mouse	12136229
heme oxygenase	1.14.99.3	Mouse	12230871
heme oxygenase	1.14.99.3	Mouse	12236785
heme oxygenase	1.14.99.3	Mouse	12372789
heme oxygenase	1.14.99.3	Mouse	12372951
heme oxygenase	1.14.99.3	Mouse	12397597
heme oxygenase	1.14.99.3	Mouse	12489116
heme oxygenase	1.14.99.3	Mouse	12508080
heme oxygenase	1.14.99.3	Mouse	12581208
heme oxygenase	1.14.99.3	Mouse	12646399
heme oxygenase	1.14.99.3	Mouse	12668974
heme oxygenase	1.14.99.3	Mouse	12709591
heme oxygenase	1.14.99.3	Mouse	12736395
heme oxygenase	1.14.99.3	Mouse	12757849
heme oxygenase	1.14.99.3	Mouse	12783778
heme oxygenase	1.14.99.3	Mouse	12874251
heme oxygenase	1.14.99.3	Mouse	12892830
heme oxygenase	1.14.99.3	Mouse	12963497
heme oxygenase	1.14.99.3	Mouse	14523007
heme oxygenase	1.14.99.3	Mouse	14649057
heme oxygenase	1.14.99.3	Mouse	14683741
heme oxygenase	1.14.99.3	Mouse	14761930
heme oxygenase	1.14.99.3	Mouse	14766239
heme oxygenase	1.14.99.3	Mouse	14985830
heme oxygenase	1.14.99.3	Mouse	15018736
heme oxygenase	1.14.99.3	Mouse	15276480
heme oxygenase	1.14.99.3	Mouse	15298625
heme oxygenase	1.14.99.3	Mouse	15345139
heme oxygenase	1.14.99.3	Mouse	15345147
heme oxygenase	1.14.99.3	Mouse	15345149
heme oxygenase	1.14.99.3	Mouse	15499042
heme oxygenase	1.14.99.3	Mouse	15499991
heme oxygenase	1.14.99.3	Mouse	15565657
heme oxygenase	1.14.99.3	Mouse	15588929
heme oxygenase	1.14.99.3	Mouse	15599843

heme oxygenase	1.14.99.3	Mouse	15649645
heme oxygenase	1.14.99.3	Mouse	1572205
heme oxygenase	1.14.99.3	Mouse	15833736
heme oxygenase	1.14.99.3	Mouse	15869055
heme oxygenase	1.14.99.3	Mouse	15896346
heme oxygenase	1.14.99.3	Mouse	15897578
heme oxygenase	1.14.99.3	Mouse	15933765
heme oxygenase	1.14.99.3	Mouse	16043027
heme oxygenase	1.14.99.3	Mouse	16048902
heme oxygenase	1.14.99.3	Mouse	16123320
heme oxygenase	1.14.99.3	Mouse	16181105
heme oxygenase	1.14.99.3	Mouse	16181109
heme oxygenase	1.14.99.3	Mouse	16214041
heme oxygenase	1.14.99.3	Mouse	16276181
heme oxygenase	1.14.99.3	Mouse	16309569
heme oxygenase	1.14.99.3	Mouse	16319139
heme oxygenase	1.14.99.3	Mouse	16374439
heme oxygenase	1.14.99.3	Mouse	16476737
heme oxygenase	1.14.99.3	Mouse	16598857
heme oxygenase	1.14.99.3	Mouse	16678019
heme oxygenase	1.14.99.3	Mouse	16775600
heme oxygenase	1.14.99.3	Mouse	16828975
heme oxygenase	1.14.99.3	Mouse	16858012
heme oxygenase	1.14.99.3	Mouse	16948473
heme oxygenase	1.14.99.3	Mouse	16964402
heme oxygenase	1.14.99.3	Mouse	16966352
heme oxygenase	1.14.99.3	Mouse	17002867
heme oxygenase	1.14.99.3	Mouse	1700666
heme oxygenase	1.14.99.3	Mouse	17042977
heme oxygenase	1.14.99.3	Mouse	1737989
heme oxygenase	1.14.99.3	Mouse	1884769
heme oxygenase	1.14.99.3	Mouse	2759552
heme oxygenase	1.14.99.3	Mouse	3107908
heme oxygenase	1.14.99.3	Mouse	3113993
heme oxygenase	1.14.99.3	Mouse	3177434
heme oxygenase	1.14.99.3	Mouse	3965329
heme oxygenase	1.14.99.3	Mouse	6548384
heme oxygenase	1.14.99.3	Mouse	7525927
heme oxygenase	1.14.99.3	Mouse	7576696
heme oxygenase	1.14.99.3	Mouse	7626076
heme oxygenase	1.14.99.3	Mouse	7768976
heme oxygenase	1.14.99.3	Mouse	7793979
heme oxygenase	1.14.99.3	Mouse	7889361

heme oxygenase	1.14.99.3	Mouse	8016102
heme oxygenase	1.14.99.3	Mouse	8216309
heme oxygenase	1.14.99.3	Mouse	8548408
heme oxygenase	1.14.99.3	Mouse	8564848
heme oxygenase	1.14.99.3	Mouse	8590277
heme oxygenase	1.14.99.3	Mouse	8694803
heme oxygenase	1.14.99.3	Mouse	8774698
heme oxygenase	1.14.99.3	Mouse	8816811
heme oxygenase	1.14.99.3	Mouse	8827779
heme oxygenase	1.14.99.3	Mouse	8852585
heme oxygenase	1.14.99.3	Mouse	8872603
heme oxygenase	1.14.99.3	Mouse	8897916
heme oxygenase	1.14.99.3	Mouse	8913885
heme oxygenase	1.14.99.3	Mouse	9125512
heme oxygenase	1.14.99.3	Mouse	9225984
heme oxygenase	1.14.99.3	Mouse	9236721
heme oxygenase	1.14.99.3	Mouse	9259978
heme oxygenase	1.14.99.3	Mouse	9276739
heme oxygenase	1.14.99.3	Mouse	9495815
heme oxygenase	1.14.99.3	Mouse	9530200
heme oxygenase	1.14.99.3	Mouse	9608682
heme oxygenase	1.14.99.3	Mouse	9927151
squalene monooxygenase	1.14.99.7	Mouse	10679280
squalene monooxygenase	1.14.99.7	Mouse	10733917
squalene monooxygenase	1.14.99.7	Mouse	11199136
squalene monooxygenase	1.14.99.7	Mouse	11520216
squalene monooxygenase	1.14.99.7	Mouse	12226513
squalene monooxygenase	1.14.99.7	Mouse	16466954
squalene monooxygenase	1.14.99.7	Mouse	6087072
squalene monooxygenase	1.14.99.7	Mouse	6547247
squalene monooxygenase	1.14.99.7	Mouse	8771716
squalene monooxygenase	1.14.99.7	Mouse	9017503
steroid 17alpha-monooxygenase	1.14.99.9	Mouse	10066888
steroid 17alpha-monooxygenase	1.14.99.9	Mouse	10352919
steroid 17alpha-monooxygenase	1.14.99.9	Mouse	10574247
steroid 17alpha-monooxygenase	1.14.99.9	Mouse	11446160
steroid 17alpha-monooxygenase	1.14.99.9	Mouse	11817165
steroid 17alpha-monooxygenase	1.14.99.9	Mouse	12444467
steroid 17alpha-monooxygenase	1.14.99.9	Mouse	15001547
steroid 17alpha-monooxygenase	1.14.99.9	Mouse	16022662
steroid 17alpha-monooxygenase	1.14.99.9	Mouse	1605399
steroid 17alpha-monooxygenase	1.14.99.9	Mouse	1607640
steroid 17alpha-monooxygenase	1.14.99.9	Mouse	1958544

steroid 17alpha-monooxygenase	1.14.99.9	Mouse	2019257
steroid 17alpha-monooxygenase	1.14.99.9	Mouse	3502608
steroid 17alpha-monooxygenase	1.14.99.9	Mouse	6593324
steroid 17alpha-monooxygenase	1.14.99.9	Mouse	9115645
steroid 17alpha-monooxygenase	1.14.99.9	Mouse	9408743
steroid 17alpha-monooxygenase	1.14.99.9	Mouse	9892022
xanthine dehydrogenase	1.17.1.4	Mouse	11086257
xanthine dehydrogenase	1.17.1.4	Mouse	11154741
xanthine dehydrogenase	1.17.1.4	Mouse	12502743
xanthine oxidase	1.17.3.2	Mouse	11086257
xanthine oxidase	1.17.3.2	Mouse	11278616
xanthine oxidase	1.17.3.2	Mouse	12423257
xanthine oxidase	1.17.3.2	Mouse	12618887
xanthine oxidase	1.17.3.2	Mouse	12780970
xanthine oxidase	1.17.3.2	Mouse	1353938
xanthine oxidase	1.17.3.2	Mouse	16429783
xanthine oxidase	1.17.3.2	Mouse	187329
xanthine oxidase	1.17.3.2	Mouse	1889832
xanthine oxidase	1.17.3.2	Mouse	3245099
xanthine oxidase	1.17.3.2	Mouse	8274165
ribonucleoside-diphosphate reductase	1.17.4.1	Mouse	10441745
ribonucleoside-diphosphate reductase	1.17.4.1	Mouse	10593972
ribonucleoside-diphosphate reductase	1.17.4.1	Mouse	10769119
ribonucleoside-diphosphate reductase	1.17.4.1	Mouse	10805162
ribonucleoside-diphosphate reductase	1.17.4.1	Mouse	10953295
ribonucleoside-diphosphate reductase	1.17.4.1	Mouse	10989193
ribonucleoside-diphosphate reductase	1.17.4.1	Mouse	11489836
ribonucleoside-diphosphate reductase	1.17.4.1	Mouse	11551528
ribonucleoside-diphosphate reductase	1.17.4.1	Mouse	11904430
ribonucleoside-diphosphate reductase	1.17.4.1	Mouse	12147300
ribonucleoside-diphosphate reductase	1.17.4.1	Mouse	12359454
ribonucleoside-diphosphate reductase	1.17.4.1	Mouse	12655059
ribonucleoside-diphosphate reductase	1.17.4.1	Mouse	12690517
ribonucleoside-diphosphate reductase	1.17.4.1	Mouse	12732713
ribonucleoside-diphosphate reductase	1.17.4.1	Mouse	12749906
ribonucleoside-diphosphate reductase	1.17.4.1	Mouse	12967138
ribonucleoside-diphosphate reductase	1.17.4.1	Mouse	1299271
ribonucleoside-diphosphate reductase	1.17.4.1	Mouse	1385411
ribonucleoside-diphosphate reductase	1.17.4.1	Mouse	1412696
ribonucleoside-diphosphate reductase	1.17.4.1	Mouse	14963934
ribonucleoside-diphosphate reductase	1.17.4.1	Mouse	14966112
ribonucleoside-diphosphate reductase	1.17.4.1	Mouse	1496919
ribonucleoside-diphosphate reductase	1.17.4.1	Mouse	15094776

ribonucleoside-diphosphate reductase	1.17.4.1	Mouse	15133626
ribonucleoside-diphosphate reductase	1.17.4.1	Mouse	1516817
ribonucleoside-diphosphate reductase	1.17.4.1	Mouse	15300180
ribonucleoside-diphosphate reductase	1.17.4.1	Mouse	15571292
ribonucleoside-diphosphate reductase	1.17.4.1	Mouse	15656518
ribonucleoside-diphosphate reductase	1.17.4.1	Mouse	15673563
ribonucleoside-diphosphate reductase	1.17.4.1	Mouse	15730856
ribonucleoside-diphosphate reductase	1.17.4.1	Mouse	15769467
ribonucleoside-diphosphate reductase	1.17.4.1	Mouse	15803490
ribonucleoside-diphosphate reductase	1.17.4.1	Mouse	15805194
ribonucleoside-diphosphate reductase	1.17.4.1	Mouse	15888728
ribonucleoside-diphosphate reductase	1.17.4.1	Mouse	160558
ribonucleoside-diphosphate reductase	1.17.4.1	Mouse	16399800
ribonucleoside-diphosphate reductase	1.17.4.1	Mouse	1643157
ribonucleoside-diphosphate reductase	1.17.4.1	Mouse	16489218
ribonucleoside-diphosphate reductase	1.17.4.1	Mouse	16530987
ribonucleoside-diphosphate reductase	1.17.4.1	Mouse	16834759
ribonucleoside-diphosphate reductase	1.17.4.1	Mouse	16925573
ribonucleoside-diphosphate reductase	1.17.4.1	Mouse	17065057
ribonucleoside-diphosphate reductase	1.17.4.1	Mouse	1717630
ribonucleoside-diphosphate reductase	1.17.4.1	Mouse	1748682
ribonucleoside-diphosphate reductase	1.17.4.1	Mouse	1793565
ribonucleoside-diphosphate reductase	1.17.4.1	Mouse	2085432
ribonucleoside-diphosphate reductase	1.17.4.1	Mouse	2178608
ribonucleoside-diphosphate reductase	1.17.4.1	Mouse	2199320
ribonucleoside-diphosphate reductase	1.17.4.1	Mouse	2257322
ribonucleoside-diphosphate reductase	1.17.4.1	Mouse	2642388
ribonucleoside-diphosphate reductase	1.17.4.1	Mouse	2673261
ribonucleoside-diphosphate reductase	1.17.4.1	Mouse	2775821
ribonucleoside-diphosphate reductase	1.17.4.1	Mouse	2827767
ribonucleoside-diphosphate reductase	1.17.4.1	Mouse	2832057
ribonucleoside-diphosphate reductase	1.17.4.1	Mouse	3044371
ribonucleoside-diphosphate reductase	1.17.4.1	Mouse	3044582
ribonucleoside-diphosphate reductase	1.17.4.1	Mouse	3061459
ribonucleoside-diphosphate reductase	1.17.4.1	Mouse	3300645
ribonucleoside-diphosphate reductase	1.17.4.1	Mouse	3511848
ribonucleoside-diphosphate reductase	1.17.4.1	Mouse	3536076
ribonucleoside-diphosphate reductase	1.17.4.1	Mouse	3907637
ribonucleoside-diphosphate reductase	1.17.4.1	Mouse	3914643
ribonucleoside-diphosphate reductase	1.17.4.1	Mouse	3915189
ribonucleoside-diphosphate reductase	1.17.4.1	Mouse	3986794
ribonucleoside-diphosphate reductase	1.17.4.1	Mouse	6353195
ribonucleoside-diphosphate reductase	1.17.4.1	Mouse	6375753

ribonucleoside-diphosphate reductase	1.17.4.1	Mouse	6752137
ribonucleoside-diphosphate reductase	1.17.4.1	Mouse	6757589
ribonucleoside-diphosphate reductase	1.17.4.1	Mouse	767333
ribonucleoside-diphosphate reductase	1.17.4.1	Mouse	7727399
ribonucleoside-diphosphate reductase	1.17.4.1	Mouse	7768988
ribonucleoside-diphosphate reductase	1.17.4.1	Mouse	7838172
ribonucleoside-diphosphate reductase	1.17.4.1	Mouse	7881162
ribonucleoside-diphosphate reductase	1.17.4.1	Mouse	7882331
ribonucleoside-diphosphate reductase	1.17.4.1	Mouse	7893463
ribonucleoside-diphosphate reductase	1.17.4.1	Mouse	7937896
ribonucleoside-diphosphate reductase	1.17.4.1	Mouse	7984431
ribonucleoside-diphosphate reductase	1.17.4.1	Mouse	8241321
ribonucleoside-diphosphate reductase	1.17.4.1	Mouse	8265664
ribonucleoside-diphosphate reductase	1.17.4.1	Mouse	8343143
ribonucleoside-diphosphate reductase	1.17.4.1	Mouse	8463252
ribonucleoside-diphosphate reductase	1.17.4.1	Mouse	8521087
ribonucleoside-diphosphate reductase	1.17.4.1	Mouse	8620054
ribonucleoside-diphosphate reductase	1.17.4.1	Mouse	8662944
ribonucleoside-diphosphate reductase	1.17.4.1	Mouse	8674535
ribonucleoside-diphosphate reductase	1.17.4.1	Mouse	8813126
ribonucleoside-diphosphate reductase	1.17.4.1	Mouse	8878781
ribonucleoside-diphosphate reductase	1.17.4.1	Mouse	8920917
ribonucleoside-diphosphate reductase	1.17.4.1	Mouse	8943056
ribonucleoside-diphosphate reductase	1.17.4.1	Mouse	9192674
ribonucleoside-diphosphate reductase	1.17.4.1	Mouse	9315670
ribonucleoside-diphosphate reductase	1.17.4.1	Mouse	9347313
ribonucleoside-diphosphate reductase	1.17.4.1	Mouse	9354452
ribonucleoside-diphosphate reductase	1.17.4.1	Mouse	9371820
ribonucleoside-diphosphate reductase	1.17.4.1	Mouse	9393942
ribonucleoside-diphosphate reductase	1.17.4.1	Mouse	9415718
ribonucleoside-diphosphate reductase	1.17.4.1	Mouse	9439883
ribonucleoside-diphosphate reductase	1.17.4.1	Mouse	9558318
ribonucleoside-diphosphate reductase	1.17.4.1	Mouse	9570515
ribonucleoside-diphosphate reductase	1.17.4.1	Mouse	9598136
ribonucleoside-diphosphate reductase	1.17.4.1	Mouse	9605773
ribonucleoside-diphosphate reductase	1.17.4.1	Mouse	9634002
ribonucleoside-diphosphate reductase	1.17.4.1	Mouse	9696008
ribonucleoside-diphosphate reductase	1.17.4.1	Mouse	9718080
ribonucleoside-diphosphate reductase	1.17.4.1	Mouse	9852067
ribonucleoside-diphosphate reductase	1.17.4.1	Mouse	9990288
glyceraldehyde-3-phosphate dehydrogenase (phosphorylating)	1.2.1.12	Mouse	10966377

glyceraldehyde-3-phosphate dehydrogenase (phosphorylating)	1.2.1.12	Mouse	11018719
glyceraldehyde-3-phosphate dehydrogenase (phosphorylating)	1.2.1.12	Mouse	12123463
glyceraldehyde-3-phosphate dehydrogenase (phosphorylating)	1.2.1.12	Mouse	12634343
glyceraldehyde-3-phosphate dehydrogenase (phosphorylating)	1.2.1.12	Mouse	14502604
glyceraldehyde-3-phosphate dehydrogenase (phosphorylating)	1.2.1.12	Mouse	15299328
glyceraldehyde-3-phosphate dehydrogenase (phosphorylating)	1.2.1.12	Mouse	15631980
glyceraldehyde-3-phosphate dehydrogenase (phosphorylating)	1.2.1.12	Mouse	15680915
glyceraldehyde-3-phosphate dehydrogenase (phosphorylating)	1.2.1.12	Mouse	3530169
glyceraldehyde-3-phosphate dehydrogenase (phosphorylating)	1.2.1.12	Mouse	6095107
glyceraldehyde-3-phosphate dehydrogenase (phosphorylating)	1.2.1.12	Mouse	6440018
glyceraldehyde-3-phosphate dehydrogenase (phosphorylating)	1.2.1.12	Mouse	664114
glyceraldehyde-3-phosphate dehydrogenase (phosphorylating)	1.2.1.12	Mouse	7165719
glyceraldehyde-3-phosphate dehydrogenase (phosphorylating)	1.2.1.12	Mouse	7173737
glyceraldehyde-3-phosphate dehydrogenase (phosphorylating)	1.2.1.12	Mouse	8304415
glyceraldehyde-3-phosphate dehydrogenase (phosphorylating)	1.2.1.12	Mouse	8454610
glyceraldehyde-3-phosphate dehydrogenase (phosphorylating)	1.2.1.12	Mouse	8791005
glyceraldehyde-3-phosphate dehydrogenase (phosphorylating)	1.2.1.12	Mouse	8805794
glyceraldehyde-3-phosphate dehydrogenase (phosphorylating)	1.2.1.12	Mouse	8905295
glyceraldehyde-3-phosphate dehydrogenase (phosphorylating)	1.2.1.12	Mouse	9437188
glyceraldehyde-3-phosphate dehydrogenase (phosphorylating)	1.2.1.12	Mouse	9793073
glyceraldehyde-3-phosphate dehydrogenase (phosphorylating)	1.2.1.12	Mouse	9922941
aldehyde dehydrogenase (NAD+)	1.2.1.3	Mouse	10913633

aldehyde dehydrogenase (NAD+)	1.2.1.3	Mouse	11306027
aldehyde dehydrogenase (NAD+)	1.2.1.3	Mouse	12223435
aldehyde dehydrogenase (NAD+)	1.2.1.3	Mouse	14597338
aldehyde dehydrogenase (NAD+)	1.2.1.3	Mouse	1472111
aldehyde dehydrogenase (NAD+)	1.2.1.3	Mouse	16126235
aldehyde dehydrogenase (NAD+)	1.2.1.3	Mouse	16499490
aldehyde dehydrogenase (NAD+)	1.2.1.3	Mouse	16878979
aldehyde dehydrogenase (NAD+)	1.2.1.3	Mouse	17607160
aldehyde dehydrogenase (NAD+)	1.2.1.3	Mouse	2332351
aldehyde dehydrogenase (NAD+)	1.2.1.3	Mouse	3593277
aldehyde dehydrogenase (NAD+)	1.2.1.3	Mouse	6603890
aldehyde dehydrogenase (NAD+)	1.2.1.3	Mouse	7910607
aldehyde dehydrogenase (NAD+)	1.2.1.3	Mouse	8452208
aldehyde dehydrogenase (NAD+)	1.2.1.3	Mouse	8823154
aldehyde dehydrogenase (NAD+)	1.2.1.3	Mouse	8850269
aldehyde dehydrogenase (NAD+)	1.2.1.3	Mouse	9013560
aldehyde dehydrogenase (NAD+)	1.2.1.3	Mouse	9228057
aldehyde dehydrogenase (NAD+)	1.2.1.3	Mouse	945270
retinal dehydrogenase	1.2.1.36	Mouse	11600826
retinal dehydrogenase	1.2.1.36	Mouse	12547725
retinal dehydrogenase	1.2.1.36	Mouse	16763553
retinal dehydrogenase	1.2.1.36	Mouse	17098734
retinal dehydrogenase	1.2.1.36	Mouse	2910311
pyruvate dehydrogenase (acetyl-transferring)	1.2.4.1	Mouse	10720420
pyruvate dehydrogenase (acetyl-transferring)	1.2.4.1	Mouse	10759582
pyruvate dehydrogenase (acetyl-transferring)	1.2.4.1	Mouse	10826498
pyruvate dehydrogenase (acetyl-transferring)	1.2.4.1	Mouse	11223534
pyruvate dehydrogenase (acetyl-transferring)	1.2.4.1	Mouse	11427685
pyruvate dehydrogenase (acetyl-transferring)	1.2.4.1	Mouse	11557577
pyruvate dehydrogenase (acetyl-transferring)	1.2.4.1	Mouse	11866475
pyruvate dehydrogenase (acetyl-transferring)	1.2.4.1	Mouse	11900279
pyruvate dehydrogenase (acetyl-transferring)	1.2.4.1	Mouse	12196468
pyruvate dehydrogenase (acetyl-transferring)	1.2.4.1	Mouse	12557749
pyruvate dehydrogenase (acetyl-transferring)	1.2.4.1	Mouse	12663261
pyruvate dehydrogenase (acetyl-transferring)	1.2.4.1	Mouse	14607783
pyruvate dehydrogenase (acetyl-transferring)	1.2.4.1	Mouse	14641018
pyruvate dehydrogenase (acetyl-transferring)	1.2.4.1	Mouse	15191896
pyruvate dehydrogenase (acetyl-transferring)	1.2.4.1	Mouse	15256563
pyruvate dehydrogenase (acetyl-transferring)	1.2.4.1	Mouse	15921824
pyruvate dehydrogenase (acetyl-transferring)	1.2.4.1	Mouse	1600837
pyruvate dehydrogenase (acetyl-transferring)	1.2.4.1	Mouse	17065338
pyruvate dehydrogenase (acetyl-transferring)	1.2.4.1	Mouse	2902801
pyruvate dehydrogenase (acetyl-transferring)	1.2.4.1	Mouse	3127087

pyruvate dehydrogenase (acetyl-transferring)	1.2.4.1	Mouse	3918587
pyruvate dehydrogenase (acetyl-transferring)	1.2.4.1	Mouse	4030556
pyruvate dehydrogenase (acetyl-transferring)	1.2.4.1	Mouse	6149743
pyruvate dehydrogenase (acetyl-transferring)	1.2.4.1	Mouse	6234885
pyruvate dehydrogenase (acetyl-transferring)	1.2.4.1	Mouse	7127258
pyruvate dehydrogenase (acetyl-transferring)	1.2.4.1	Mouse	7848280
pyruvate dehydrogenase (acetyl-transferring)	1.2.4.1	Mouse	7864215
pyruvate dehydrogenase (acetyl-transferring)	1.2.4.1	Mouse	8557765
pyruvate dehydrogenase (acetyl-transferring)	1.2.4.1	Mouse	9089387
pyruvate dehydrogenase (acetyl-transferring)	1.2.4.1	Mouse	9381974
pyruvate dehydrogenase (acetyl-transferring)	1.2.4.1	Mouse	9426381
pyruvate dehydrogenase (acetyl-transferring)	1.2.4.1	Mouse	9438382
pyruvate dehydrogenase (acetyl-transferring)	1.2.4.1	Mouse	9497164
pyruvate dehydrogenase (acetyl-transferring)	1.2.4.1	Mouse	9725804
oxoglutarate dehydrogenase (succinyl-transferring)	1.2.4.2	Mouse	1352447
oxoglutarate dehydrogenase (succinyl-transferring)	1.2.4.2	Mouse	15356188
oxoglutarate dehydrogenase (succinyl-transferring)	1.2.4.2	Mouse	15466852
oxoglutarate dehydrogenase (succinyl-transferring)	1.2.4.2	Mouse	423807
oxoglutarate dehydrogenase (succinyl-transferring)	1.2.4.2	Mouse	6778477
oxoglutarate dehydrogenase (succinyl-transferring)	1.2.4.2	Mouse	7995856
3-methyl-2-oxobutanoate dehydrogenase	1.2.4.4	Mouse	10562606
3-methyl-2-oxobutanoate dehydrogenase	1.2.4.4	Mouse	11385053
3-methyl-2-oxobutanoate dehydrogenase	1.2.4.4	Mouse	17656140
3-methyl-2-oxobutanoate dehydrogenase	1.2.4.4	Mouse	3343251
3-methyl-2-oxobutanoate dehydrogenase	1.2.4.4	Mouse	4054446
3-methyl-2-oxobutanoate dehydrogenase	1.2.4.4	Mouse	6510417
3-methyl-2-oxobutanoate dehydrogenase	1.2.4.4	Mouse	9460082
enoyl-[acyl-carrier-protein] reductase (NADPH, B-specific)	1.3.1.10	Mouse	17095231
enoyl-[acyl-carrier-protein] reductase (NADPH, B-specific)	1.3.1.10	Mouse	8075395
dihydropyrimidine dehydrogenase (NADP+)	1.3.1.2	Mouse	10348793
dihydropyrimidine dehydrogenase (NADP+)	1.3.1.2	Mouse	10473079
dihydropyrimidine dehydrogenase (NADP+)	1.3.1.2	Mouse	10848981
dihydropyrimidine dehydrogenase (NADP+)	1.3.1.2	Mouse	10853015
dihydropyrimidine dehydrogenase (NADP+)	1.3.1.2	Mouse	10887632
dihydropyrimidine dehydrogenase (NADP+)	1.3.1.2	Mouse	10897217

dihydropyrimidine dehydrogenase (NADP+)	1.3.1.2	Mouse	11060767
dihydropyrimidine dehydrogenase (NADP+)	1.3.1.2	Mouse	11098485
dihydropyrimidine dehydrogenase (NADP+)	1.3.1.2	Mouse	11219978
dihydropyrimidine dehydrogenase (NADP+)	1.3.1.2	Mouse	11267945
dihydropyrimidine dehydrogenase (NADP+)	1.3.1.2	Mouse	11334264
dihydropyrimidine dehydrogenase (NADP+)	1.3.1.2	Mouse	11376565
dihydropyrimidine dehydrogenase (NADP+)	1.3.1.2	Mouse	11445849
dihydropyrimidine dehydrogenase (NADP+)	1.3.1.2	Mouse	11555593
dihydropyrimidine dehydrogenase (NADP+)	1.3.1.2	Mouse	11555601
dihydropyrimidine dehydrogenase (NADP+)	1.3.1.2	Mouse	11697836
dihydropyrimidine dehydrogenase (NADP+)	1.3.1.2	Mouse	11862480
dihydropyrimidine dehydrogenase (NADP+)	1.3.1.2	Mouse	11865631
dihydropyrimidine dehydrogenase (NADP+)	1.3.1.2	Mouse	11936689
dihydropyrimidine dehydrogenase (NADP+)	1.3.1.2	Mouse	11962674
dihydropyrimidine dehydrogenase (NADP+)	1.3.1.2	Mouse	12084458
dihydropyrimidine dehydrogenase (NADP+)	1.3.1.2	Mouse	12187768
dihydropyrimidine dehydrogenase (NADP+)	1.3.1.2	Mouse	12469154
dihydropyrimidine dehydrogenase (NADP+)	1.3.1.2	Mouse	12527935
dihydropyrimidine dehydrogenase (NADP+)	1.3.1.2	Mouse	12616366
dihydropyrimidine dehydrogenase (NADP+)	1.3.1.2	Mouse	12820455
dihydropyrimidine dehydrogenase (NADP+)	1.3.1.2	Mouse	12885330
dihydropyrimidine dehydrogenase (NADP+)	1.3.1.2	Mouse	12967482
dihydropyrimidine dehydrogenase (NADP+)	1.3.1.2	Mouse	14689231
dihydropyrimidine dehydrogenase (NADP+)	1.3.1.2	Mouse	14744810
dihydropyrimidine dehydrogenase (NADP+)	1.3.1.2	Mouse	15025949
dihydropyrimidine dehydrogenase (NADP+)	1.3.1.2	Mouse	15045945
dihydropyrimidine dehydrogenase (NADP+)	1.3.1.2	Mouse	15069534
dihydropyrimidine dehydrogenase (NADP+)	1.3.1.2	Mouse	15134221
dihydropyrimidine dehydrogenase (NADP+)	1.3.1.2	Mouse	15222105
dihydropyrimidine dehydrogenase (NADP+)	1.3.1.2	Mouse	1581906
dihydropyrimidine dehydrogenase (NADP+)	1.3.1.2	Mouse	15944938
dihydropyrimidine dehydrogenase (NADP+)	1.3.1.2	Mouse	16163233
dihydropyrimidine dehydrogenase (NADP+)	1.3.1.2	Mouse	1629785
dihydropyrimidine dehydrogenase (NADP+)	1.3.1.2	Mouse	16556484
dihydropyrimidine dehydrogenase (NADP+)	1.3.1.2	Mouse	16761622
dihydropyrimidine dehydrogenase (NADP+)	1.3.1.2	Mouse	16786143
dihydropyrimidine dehydrogenase (NADP+)	1.3.1.2	Mouse	16912518
dihydropyrimidine dehydrogenase (NADP+)	1.3.1.2	Mouse	17046731
dihydropyrimidine dehydrogenase (NADP+)	1.3.1.2	Mouse	2060083
dihydropyrimidine dehydrogenase (NADP+)	1.3.1.2	Mouse	3202908
dihydropyrimidine dehydrogenase (NADP+)	1.3.1.2	Mouse	7602356
dihydropyrimidine dehydrogenase (NADP+)	1.3.1.2	Mouse	7825962
dihydropyrimidine dehydrogenase (NADP+)	1.3.1.2	Mouse	8093030

dihydropyrimidine dehydrogenase (NADP+)	1.3.1.2	Mouse	8097697
dihydropyrimidine dehydrogenase (NADP+)	1.3.1.2	Mouse	8504424
dihydropyrimidine dehydrogenase (NADP+)	1.3.1.2	Mouse	8878781
dihydropyrimidine dehydrogenase (NADP+)	1.3.1.2	Mouse	8895681
dihydropyrimidine dehydrogenase (NADP+)	1.3.1.2	Mouse	8950202
dihydropyrimidine dehydrogenase (NADP+)	1.3.1.2	Mouse	9029059
dihydropyrimidine dehydrogenase (NADP+)	1.3.1.2	Mouse	9056186
dihydropyrimidine dehydrogenase (NADP+)	1.3.1.2	Mouse	9152608
dihydropyrimidine dehydrogenase (NADP+)	1.3.1.2	Mouse	9182832
dihydropyrimidine dehydrogenase (NADP+)	1.3.1.2	Mouse	9264323
dihydropyrimidine dehydrogenase (NADP+)	1.3.1.2	Mouse	9280881
dihydropyrimidine dehydrogenase (NADP+)	1.3.1.2	Mouse	9414600
dihydropyrimidine dehydrogenase (NADP+)	1.3.1.2	Mouse	9464498
dihydropyrimidine dehydrogenase (NADP+)	1.3.1.2	Mouse	9597696
dihydropyrimidine dehydrogenase (NADP+)	1.3.1.2	Mouse	9808711
dihydropyrimidine dehydrogenase (NADP+)	1.3.1.2	Mouse	9819714
dihydropyrimidine dehydrogenase (NADP+)	1.3.1.2	Mouse	9914783
dihydroorotate oxidase	1.3.3.1	Mouse	10348793
dihydroorotate oxidase	1.3.3.1	Mouse	10473079
dihydroorotate oxidase	1.3.3.1	Mouse	10848981
dihydroorotate oxidase	1.3.3.1	Mouse	10853015
dihydroorotate oxidase	1.3.3.1	Mouse	10878295
dihydroorotate oxidase	1.3.3.1	Mouse	10887632
dihydroorotate oxidase	1.3.3.1	Mouse	11053058
dihydroorotate oxidase	1.3.3.1	Mouse	11060767
dihydroorotate oxidase	1.3.3.1	Mouse	11098485
dihydroorotate oxidase	1.3.3.1	Mouse	11219978
dihydroorotate oxidase	1.3.3.1	Mouse	11267945
dihydroorotate oxidase	1.3.3.1	Mouse	11334264
dihydroorotate oxidase	1.3.3.1	Mouse	11376565
dihydroorotate oxidase	1.3.3.1	Mouse	11522581
dihydroorotate oxidase	1.3.3.1	Mouse	11555601
dihydroorotate oxidase	1.3.3.1	Mouse	11697836
dihydroorotate oxidase	1.3.3.1	Mouse	11796730
dihydroorotate oxidase	1.3.3.1	Mouse	11862480
dihydroorotate oxidase	1.3.3.1	Mouse	11865631
dihydroorotate oxidase	1.3.3.1	Mouse	11936689
dihydroorotate oxidase	1.3.3.1	Mouse	11962674
dihydroorotate oxidase	1.3.3.1	Mouse	12084458
dihydroorotate oxidase	1.3.3.1	Mouse	12187768
dihydroorotate oxidase	1.3.3.1	Mouse	12469154
dihydroorotate oxidase	1.3.3.1	Mouse	12527935
dihydroorotate oxidase	1.3.3.1	Mouse	12634225

dihydroorotate oxidase	1.3.3.1	Mouse	12820455
dihydroorotate oxidase	1.3.3.1	Mouse	12885330
dihydroorotate oxidase	1.3.3.1	Mouse	12967482
dihydroorotate oxidase	1.3.3.1	Mouse	14689231
dihydroorotate oxidase	1.3.3.1	Mouse	14744810
dihydroorotate oxidase	1.3.3.1	Mouse	15025949
dihydroorotate oxidase	1.3.3.1	Mouse	15045945
dihydroorotate oxidase	1.3.3.1	Mouse	15069534
dihydroorotate oxidase	1.3.3.1	Mouse	15134221
dihydroorotate oxidase	1.3.3.1	Mouse	15222105
dihydroorotate oxidase	1.3.3.1	Mouse	15450176
dihydroorotate oxidase	1.3.3.1	Mouse	15944938
dihydroorotate oxidase	1.3.3.1	Mouse	16163233
dihydroorotate oxidase	1.3.3.1	Mouse	1629785
dihydroorotate oxidase	1.3.3.1	Mouse	16556484
dihydroorotate oxidase	1.3.3.1	Mouse	17046731
dihydroorotate oxidase	1.3.3.1	Mouse	2060083
dihydroorotate oxidase	1.3.3.1	Mouse	3202908
dihydroorotate oxidase	1.3.3.1	Mouse	6761622
dihydroorotate oxidase	1.3.3.1	Mouse	7602356
dihydroorotate oxidase	1.3.3.1	Mouse	7825962
dihydroorotate oxidase	1.3.3.1	Mouse	8093030
dihydroorotate oxidase	1.3.3.1	Mouse	8097697
dihydroorotate oxidase	1.3.3.1	Mouse	8878781
dihydroorotate oxidase	1.3.3.1	Mouse	8895681
dihydroorotate oxidase	1.3.3.1	Mouse	8950202
dihydroorotate oxidase	1.3.3.1	Mouse	9056186
dihydroorotate oxidase	1.3.3.1	Mouse	9182832
dihydroorotate oxidase	1.3.3.1	Mouse	9280881
dihydroorotate oxidase	1.3.3.1	Mouse	9464498
dihydroorotate oxidase	1.3.3.1	Mouse	9597696
dihydroorotate oxidase	1.3.3.1	Mouse	9636062
dihydroorotate oxidase	1.3.3.1	Mouse	9819714
dihydroorotate oxidase	1.3.3.1	Mouse	9860876
dihydroorotate oxidase	1.3.3.1	Mouse	9914783
coproporphyrinogen oxidase	1.3.3.3	Mouse	10787385
coproporphyrinogen oxidase	1.3.3.3	Mouse	11368326
coproporphyrinogen oxidase	1.3.3.3	Mouse	16567402
acyl-CoA oxidase	1.3.3.6	Mouse	10318668
acyl-CoA oxidase	1.3.3.6	Mouse	11156684
acyl-CoA oxidase	1.3.3.6	Mouse	11330046
acyl-CoA oxidase	1.3.3.6	Mouse	12538078
acyl-CoA oxidase	1.3.3.6	Mouse	12758125

acyl-CoA oxidase	1.3.3.6	Mouse	14500732
acyl-CoA oxidase	1.3.3.6	Mouse	15805059
acyl-CoA oxidase	1.3.3.6	Mouse	1989516
acyl-CoA oxidase	1.3.3.6	Mouse	2049482
acyl-CoA oxidase	1.3.3.6	Mouse	2811611
acyl-CoA oxidase	1.3.3.6	Mouse	3367697
acyl-CoA oxidase	1.3.3.6	Mouse	3732222
acyl-CoA oxidase	1.3.3.6	Mouse	518563
acyl-CoA oxidase	1.3.3.6	Mouse	6240978
acyl-CoA oxidase	1.3.3.6	Mouse	6540549
acyl-CoA oxidase	1.3.3.6	Mouse	6541949
acyl-CoA oxidase	1.3.3.6	Mouse	7860752
acyl-CoA oxidase	1.3.3.6	Mouse	7867664
acyl-CoA oxidase	1.3.3.6	Mouse	8662598
acyl-CoA oxidase	1.3.3.6	Mouse	8784738
acyl-CoA oxidase	1.3.3.6	Mouse	8798738
acyl-CoA oxidase	1.3.3.6	Mouse	8895731
long-chain-acyl-CoA dehydrogenase	1.3.99.13	Mouse	12716879
long-chain-acyl-CoA dehydrogenase	1.3.99.13	Mouse	15535801
long-chain-acyl-CoA dehydrogenase	1.3.99.13	Mouse	15850553
long-chain-acyl-CoA dehydrogenase	1.3.99.13	Mouse	1637289
long-chain-acyl-CoA dehydrogenase	1.3.99.13	Mouse	1744086
long-chain-acyl-CoA dehydrogenase	1.3.99.13	Mouse	2914148
long-chain-acyl-CoA dehydrogenase	1.3.99.13	Mouse	3967008
long-chain-acyl-CoA dehydrogenase	1.3.99.13	Mouse	7551821
long-chain-acyl-CoA dehydrogenase	1.3.99.13	Mouse	8034667
long-chain-acyl-CoA dehydrogenase	1.3.99.13	Mouse	8798738
long-chain-acyl-CoA dehydrogenase	1.3.99.13	Mouse	8941110
long-chain-acyl-CoA dehydrogenase	1.3.99.13	Mouse	9177236
long-chain-acyl-CoA dehydrogenase	1.3.99.13	Mouse	9973285
butyryl-CoA dehydrogenase	1.3.99.2	Mouse	12892042
butyryl-CoA dehydrogenase	1.3.99.2	Mouse	15358373
butyryl-CoA dehydrogenase	1.3.99.2	Mouse	15850406
butyryl-CoA dehydrogenase	1.3.99.2	Mouse	16297616
butyryl-CoA dehydrogenase	1.3.99.2	Mouse	8941110
butyryl-CoA dehydrogenase	1.3.99.2	Mouse	9177236
butyryl-CoA dehydrogenase	1.3.99.2	Mouse	9271417
acyl-CoA dehydrogenase	1.3.99.3	Mouse	10832093
acyl-CoA dehydrogenase	1.3.99.3	Mouse	12359260
acyl-CoA dehydrogenase	1.3.99.3	Mouse	15358373
acyl-CoA dehydrogenase	1.3.99.3	Mouse	15850406
acyl-CoA dehydrogenase	1.3.99.3	Mouse	9177236
acyl-CoA dehydrogenase	1.3.99.3	Mouse	9484241

3-oxo-5alpha-steroid 4-dehydrogenase	1.3.99.5	Mouse	11408363
3-oxo-5alpha-steroid 4-dehydrogenase	1.3.99.5	Mouse	12746845
3-oxo-5alpha-steroid 4-dehydrogenase	1.3.99.5	Mouse	16174723
3-oxo-5alpha-steroid 4-dehydrogenase	1.3.99.5	Mouse	6523531
3-oxo-5alpha-steroid 4-dehydrogenase	1.3.99.5	Mouse	7588289
3-oxo-5alpha-steroid 4-dehydrogenase	1.3.99.5	Mouse	9078395
glutamate dehydrogenase [NAD(P)+]	1.4.1.3	Mouse	17507377
glutamate dehydrogenase [NAD(P)+]	1.4.1.3	Mouse	3139028
glutamate dehydrogenase [NAD(P)+]	1.4.1.3	Mouse	3449598
glutamate dehydrogenase [NAD(P)+]	1.4.1.3	Mouse	7470041
glutamate dehydrogenase [NAD(P)+]	1.4.1.3	Mouse	8122033
pyridoxal 5'-phosphate synthase	1.4.3.5	Mouse	6822512
pyridoxal 5'-phosphate synthase	1.4.3.5	Mouse	9601034
1-pyrroline-5-carboxylate dehydrogenase	1.5.1.12	Mouse	10363370
1-pyrroline-5-carboxylate dehydrogenase	1.5.1.12	Mouse	10398729
1-pyrroline-5-carboxylate dehydrogenase	1.5.1.12	Mouse	10441499
1-pyrroline-5-carboxylate dehydrogenase	1.5.1.12	Mouse	10759508
1-pyrroline-5-carboxylate dehydrogenase	1.5.1.12	Mouse	10945345
1-pyrroline-5-carboxylate dehydrogenase	1.5.1.12	Mouse	11726714
1-pyrroline-5-carboxylate dehydrogenase	1.5.1.12	Mouse	12602867
1-pyrroline-5-carboxylate dehydrogenase	1.5.1.12	Mouse	14602584
1-pyrroline-5-carboxylate dehydrogenase	1.5.1.12	Mouse	15077666
1-pyrroline-5-carboxylate dehydrogenase	1.5.1.12	Mouse	2860838
1-pyrroline-5-carboxylate dehydrogenase	1.5.1.12	Mouse	9351242
methylenetetrahydrofolate dehydrogenase (NAD+)	1.5.1.15	Mouse	718836
methylenetetrahydrofolate reductase [NAD(P)H]	1.5.1.20	Mouse	10462593
methylenetetrahydrofolate reductase [NAD(P)H]	1.5.1.20	Mouse	10948708
methylenetetrahydrofolate reductase [NAD(P)H]	1.5.1.20	Mouse	11302003
methylenetetrahydrofolate reductase [NAD(P)H]	1.5.1.20	Mouse	11343335
methylenetetrahydrofolate reductase [NAD(P)H]	1.5.1.20	Mouse	11398138
methylenetetrahydrofolate reductase [NAD(P)H]	1.5.1.20	Mouse	11680544
methylenetetrahydrofolate reductase [NAD(P)H]	1.5.1.20	Mouse	11712321
methylenetetrahydrofolate reductase [NAD(P)H]	1.5.1.20	Mouse	11927833

methylenetetrahydrofolate reductase [NAD(P)H]	1.5.1.20	Mouse	12028998
methylenetetrahydrofolate reductase [NAD(P)H]	1.5.1.20	Mouse	12038037
methylenetetrahydrofolate reductase [NAD(P)H]	1.5.1.20	Mouse	12600862
methylenetetrahydrofolate reductase [NAD(P)H]	1.5.1.20	Mouse	12897091
methylenetetrahydrofolate reductase [NAD(P)H]	1.5.1.20	Mouse	12914571
methylenetetrahydrofolate reductase [NAD(P)H]	1.5.1.20	Mouse	14608052
methylenetetrahydrofolate reductase [NAD(P)H]	1.5.1.20	Mouse	14728017
methylenetetrahydrofolate reductase [NAD(P)H]	1.5.1.20	Mouse	14973104
methylenetetrahydrofolate reductase [NAD(P)H]	1.5.1.20	Mouse	15033905
methylenetetrahydrofolate reductase [NAD(P)H]	1.5.1.20	Mouse	15207432
methylenetetrahydrofolate reductase [NAD(P)H]	1.5.1.20	Mouse	15449187
methylenetetrahydrofolate reductase [NAD(P)H]	1.5.1.20	Mouse	15546509
methylenetetrahydrofolate reductase [NAD(P)H]	1.5.1.20	Mouse	15581487
methylenetetrahydrofolate reductase [NAD(P)H]	1.5.1.20	Mouse	15598763
methylenetetrahydrofolate reductase [NAD(P)H]	1.5.1.20	Mouse	15688606
methylenetetrahydrofolate reductase [NAD(P)H]	1.5.1.20	Mouse	15773669
methylenetetrahydrofolate reductase [NAD(P)H]	1.5.1.20	Mouse	15894672
methylenetetrahydrofolate reductase [NAD(P)H]	1.5.1.20	Mouse	15935452
methylenetetrahydrofolate reductase [NAD(P)H]	1.5.1.20	Mouse	16055944
methylenetetrahydrofolate reductase [NAD(P)H]	1.5.1.20	Mouse	16108833
methylenetetrahydrofolate reductase [NAD(P)H]	1.5.1.20	Mouse	16128738

methylenetetrahydrofolate reductase [NAD(P)H]	1.5.1.20	Mouse	16274753
methylenetetrahydrofolate reductase [NAD(P)H]	1.5.1.20	Mouse	16538645
methylenetetrahydrofolate reductase [NAD(P)H]	1.5.1.20	Mouse	16706930
methylenetetrahydrofolate reductase [NAD(P)H]	1.5.1.20	Mouse	17105984
methylenetetrahydrofolate reductase [NAD(P)H]	1.5.1.20	Mouse	3143307
methylenetetrahydrofolate reductase [NAD(P)H]	1.5.1.20	Mouse	6352699
methylenetetrahydrofolate reductase [NAD(P)H]	1.5.1.20	Mouse	6391540
methylenetetrahydrofolate reductase [NAD(P)H]	1.5.1.20	Mouse	9789068
6,7-dihydropteridine reductase	1.5.1.34	Mouse	2913929
6,7-dihydropteridine reductase	1.5.1.34	Mouse	6797415
formyltetrahydrofolate dehydrogenase	1.5.1.6	Mouse	9359603
proline dehydrogenase	1.5.99.8	Mouse	11788754
proline dehydrogenase	1.5.99.8	Mouse	12602867
dihydrolipoyl dehydrogenase	1.8.1.4	Mouse	10885793
dihydrolipoyl dehydrogenase	1.8.1.4	Mouse	11641455
dihydrolipoyl dehydrogenase	1.8.1.4	Mouse	12812918
dihydrolipoyl dehydrogenase	1.8.1.4	Mouse	15173434
dihydrolipoyl dehydrogenase	1.8.1.4	Mouse	15915669
dihydrolipoyl dehydrogenase	1.8.1.4	Mouse	16581023
dihydrolipoyl dehydrogenase	1.8.1.4	Mouse	16616211
dihydrolipoyl dehydrogenase	1.8.1.4	Mouse	16875466
dihydrolipoyl dehydrogenase	1.8.1.4	Mouse	2381301
dihydrolipoyl dehydrogenase	1.8.1.4	Mouse	2404025
dihydrolipoyl dehydrogenase	1.8.1.4	Mouse	3103467
dihydrolipoyl dehydrogenase	1.8.1.4	Mouse	3425711
dihydrolipoyl dehydrogenase	1.8.1.4	Mouse	7782942
glutathione-disulfide reductase	1.8.1.7	Mouse	11133045
glutathione-disulfide reductase	1.8.1.7	Mouse	12204336
glutathione-disulfide reductase	1.8.1.7	Mouse	12453665
glutathione-disulfide reductase	1.8.1.7	Mouse	1605642
glutathione-disulfide reductase	1.8.1.7	Mouse	2848577
glutathione-disulfide reductase	1.8.1.7	Mouse	3698652
glutathione-disulfide reductase	1.8.1.7	Mouse	3963383
glutathione-disulfide reductase	1.8.1.7	Mouse	8843715
glutathione-disulfide reductase	1.8.1.7	Mouse	9350472

cytochrome-c oxidase	1.9.3.1	Mouse	10490029
cytochrome-c oxidase	1.9.3.1	Mouse	11988227
cytochrome-c oxidase	1.9.3.1	Mouse	12059041
cytochrome-c oxidase	1.9.3.1	Mouse	12145150
cytochrome-c oxidase	1.9.3.1	Mouse	12874793
cytochrome-c oxidase	1.9.3.1	Mouse	1315683
cytochrome-c oxidase	1.9.3.1	Mouse	1450614
cytochrome-c oxidase	1.9.3.1	Mouse	15504366
cytochrome-c oxidase	1.9.3.1	Mouse	15708625
cytochrome-c oxidase	1.9.3.1	Mouse	16704969
cytochrome-c oxidase	1.9.3.1	Mouse	167928
cytochrome-c oxidase	1.9.3.1	Mouse	174553
cytochrome-c oxidase	1.9.3.1	Mouse	194851
cytochrome-c oxidase	1.9.3.1	Mouse	206437
cytochrome-c oxidase	1.9.3.1	Mouse	2822680
cytochrome-c oxidase	1.9.3.1	Mouse	2849368
cytochrome-c oxidase	1.9.3.1	Mouse	2854388
cytochrome-c oxidase	1.9.3.1	Mouse	3000820
cytochrome-c oxidase	1.9.3.1	Mouse	3002436
cytochrome-c oxidase	1.9.3.1	Mouse	356174
cytochrome-c oxidase	1.9.3.1	Mouse	3569141
cytochrome-c oxidase	1.9.3.1	Mouse	3924042
cytochrome-c oxidase	1.9.3.1	Mouse	6091751
cytochrome-c oxidase	1.9.3.1	Mouse	6254961
cytochrome-c oxidase	1.9.3.1	Mouse	6268138
cytochrome-c oxidase	1.9.3.1	Mouse	6286610
cytochrome-c oxidase	1.9.3.1	Mouse	6320180
cytochrome-c oxidase	1.9.3.1	Mouse	7356983
cytochrome-c oxidase	1.9.3.1	Mouse	8013452
cytochrome-c oxidase	1.9.3.1	Mouse	8386021
phosphatidylethanolamine N-methyltransferase	2.1.1.17	Mouse	8207329
phosphatidylethanolamine N-methyltransferase	2.1.1.17	Mouse	9380436
thymidylate synthase	2.1.1.45	Mouse	11358693
thymidylate synthase	2.1.1.45	Mouse	12412165
thymidylate synthase	2.1.1.45	Mouse	12457437
thymidylate synthase	2.1.1.45	Mouse	12470718
thymidylate synthase	2.1.1.45	Mouse	12544347
thymidylate synthase	2.1.1.45	Mouse	14578129
thymidylate synthase	2.1.1.45	Mouse	14648018
thymidylate synthase	2.1.1.45	Mouse	14689231
thymidylate synthase	2.1.1.45	Mouse	15025949

thymidylate synthase	2.1.1.45	Mouse	15134221
thymidylate synthase	2.1.1.45	Mouse	15598787
thymidylate synthase	2.1.1.45	Mouse	16077970
thymidylate synthase	2.1.1.45	Mouse	16540728
thymidylate synthase	2.1.1.45	Mouse	16617381
thymidylate synthase	2.1.1.45	Mouse	3709927
thymidylate synthase	2.1.1.45	Mouse	7602356
thymidylate synthase	2.1.1.45	Mouse	8805515
phosphoribosylaminoimidazolecarboxamide formyltransferase	2.1.2.3	Mouse	6335666
aspartate carbamoyltransferase	2.1.3.2	Mouse	10336386
aspartate carbamoyltransferase	2.1.3.2	Mouse	10593256
aspartate carbamoyltransferase	2.1.3.2	Mouse	11397099
aspartate carbamoyltransferase	2.1.3.2	Mouse	15165857
aspartate carbamoyltransferase	2.1.3.2	Mouse	15529744
aspartate carbamoyltransferase	2.1.3.2	Mouse	16120448
aspartate carbamoyltransferase	2.1.3.2	Mouse	17008138
aspartate carbamoyltransferase	2.1.3.2	Mouse	3047117
aspartate carbamoyltransferase	2.1.3.2	Mouse	3542019
aspartate carbamoyltransferase	2.1.3.2	Mouse	3907993
aspartate carbamoyltransferase	2.1.3.2	Mouse	4092695
aspartate carbamoyltransferase	2.1.3.2	Mouse	6115855
aspartate carbamoyltransferase	2.1.3.2	Mouse	6298785
aspartate carbamoyltransferase	2.1.3.2	Mouse	7051000
aspartate carbamoyltransferase	2.1.3.2	Mouse	9611817
aspartate carbamoyltransferase	2.1.3.2	Mouse	9626698
aspartate carbamoyltransferase	2.1.3.2	Mouse	9659392
ornithine carbamoyltransferase	2.1.3.3	Mouse	1505922
ornithine carbamoyltransferase	2.1.3.3	Mouse	205703
ornithine carbamoyltransferase	2.1.3.3	Mouse	2667139
ornithine carbamoyltransferase	2.1.3.3	Mouse	2667140
ornithine carbamoyltransferase	2.1.3.3	Mouse	2667141
ornithine carbamoyltransferase	2.1.3.3	Mouse	8019156
ornithine carbamoyltransferase	2.1.3.3	Mouse	8168544
ornithine carbamoyltransferase	2.1.3.3	Mouse	9501170
ornithine carbamoyltransferase	2.1.3.3	Mouse	9540805
glycine amidinotransferase	2.1.4.1	Mouse	7419715
transketolase	2.2.1.1	Mouse	10975072
transketolase	2.2.1.1	Mouse	11072071
transketolase	2.2.1.1	Mouse	16354724
transketolase	2.2.1.1	Mouse	1939098
transketolase	2.2.1.1	Mouse	9924800
transaldolase	2.2.1.2	Mouse	11390181

transaldolase	2.2.1.2	Mouse	12359249
transaldolase	2.2.1.2	Mouse	15263091
transaldolase	2.2.1.2	Mouse	16092052
transaldolase	2.2.1.2	Mouse	17046540
transaldolase	2.2.1.2	Mouse	9565623
transaldolase	2.2.1.2	Mouse	9973403
dihydrolipoyllysine-residue acetyltransferase	2.3.1.12	Mouse	3840997
glycerol-3-phosphate O-acyltransferase	2.3.1.15	Mouse	11003606
glycerol-3-phosphate O-acyltransferase	2.3.1.15	Mouse	11284717
glycerol-3-phosphate O-acyltransferase	2.3.1.15	Mouse	12464581
glycerol-3-phosphate O-acyltransferase	2.3.1.15	Mouse	12730219
glycerol-3-phosphate O-acyltransferase	2.3.1.15	Mouse	14724270
glycerol-3-phosphate O-acyltransferase	2.3.1.15	Mouse	15102885
glycerol-3-phosphate O-acyltransferase	2.3.1.15	Mouse	16234267
glycerol-3-phosphate O-acyltransferase	2.3.1.15	Mouse	16431156
glycerol-3-phosphate O-acyltransferase	2.3.1.15	Mouse	16507761
glycerol-3-phosphate O-acyltransferase	2.3.1.15	Mouse	2303421
glycerol-3-phosphate O-acyltransferase	2.3.1.15	Mouse	2559137
glycerol-3-phosphate O-acyltransferase	2.3.1.15	Mouse	2674663
glycerol-3-phosphate O-acyltransferase	2.3.1.15	Mouse	2737209
glycerol-3-phosphate O-acyltransferase	2.3.1.15	Mouse	2830105
glycerol-3-phosphate O-acyltransferase	2.3.1.15	Mouse	3103689
glycerol-3-phosphate O-acyltransferase	2.3.1.15	Mouse	3311005
glycerol-3-phosphate O-acyltransferase	2.3.1.15	Mouse	3334861
glycerol-3-phosphate O-acyltransferase	2.3.1.15	Mouse	6497849
glycerol-3-phosphate O-acyltransferase	2.3.1.15	Mouse	813635
glycerol-3-phosphate O-acyltransferase	2.3.1.15	Mouse	8387510
glycerol-3-phosphate O-acyltransferase	2.3.1.15	Mouse	9393688
acetyl-CoA C-acyltransferase	2.3.1.16	Mouse	3967008
acetyl-CoA C-acyltransferase	2.3.1.16	Mouse	7068598
acetyl-CoA C-acyltransferase	2.3.1.16	Mouse	7374368
carnitine O-palmitoyltransferase	2.3.1.21	Mouse	10417344
carnitine O-palmitoyltransferase	2.3.1.21	Mouse	10709666
carnitine O-palmitoyltransferase	2.3.1.21	Mouse	10956641
carnitine O-palmitoyltransferase	2.3.1.21	Mouse	11356163
carnitine O-palmitoyltransferase	2.3.1.21	Mouse	11371554
carnitine O-palmitoyltransferase	2.3.1.21	Mouse	11463952
carnitine O-palmitoyltransferase	2.3.1.21	Mouse	12015320
carnitine O-palmitoyltransferase	2.3.1.21	Mouse	12359092
carnitine O-palmitoyltransferase	2.3.1.21	Mouse	12574149
carnitine O-palmitoyltransferase	2.3.1.21	Mouse	12619873
carnitine O-palmitoyltransferase	2.3.1.21	Mouse	12761301
carnitine O-palmitoyltransferase	2.3.1.21	Mouse	14751860

carnitine O-palmitoyltransferase	2.3.1.21	Mouse	15044358
carnitine O-palmitoyltransferase	2.3.1.21	Mouse	15247243
carnitine O-palmitoyltransferase	2.3.1.21	Mouse	15539300
carnitine O-palmitoyltransferase	2.3.1.21	Mouse	15590999
carnitine O-palmitoyltransferase	2.3.1.21	Mouse	15623825
carnitine O-palmitoyltransferase	2.3.1.21	Mouse	1563551
carnitine O-palmitoyltransferase	2.3.1.21	Mouse	15878185
carnitine O-palmitoyltransferase	2.3.1.21	Mouse	15919095
carnitine O-palmitoyltransferase	2.3.1.21	Mouse	16177188
carnitine O-palmitoyltransferase	2.3.1.21	Mouse	16225603
carnitine O-palmitoyltransferase	2.3.1.21	Mouse	16509570
carnitine O-palmitoyltransferase	2.3.1.21	Mouse	16528409
carnitine O-palmitoyltransferase	2.3.1.21	Mouse	16545538
carnitine O-palmitoyltransferase	2.3.1.21	Mouse	16584169
carnitine O-palmitoyltransferase	2.3.1.21	Mouse	16763001
carnitine O-palmitoyltransferase	2.3.1.21	Mouse	17021367
carnitine O-palmitoyltransferase	2.3.1.21	Mouse	17089095
carnitine O-palmitoyltransferase	2.3.1.21	Mouse	1979695
carnitine O-palmitoyltransferase	2.3.1.21	Mouse	2166437
carnitine O-palmitoyltransferase	2.3.1.21	Mouse	2914148
carnitine O-palmitoyltransferase	2.3.1.21	Mouse	3090894
carnitine O-palmitoyltransferase	2.3.1.21	Mouse	3709811
carnitine O-palmitoyltransferase	2.3.1.21	Mouse	3800962
carnitine O-palmitoyltransferase	2.3.1.21	Mouse	3834060
carnitine O-palmitoyltransferase	2.3.1.21	Mouse	6105152
carnitine O-palmitoyltransferase	2.3.1.21	Mouse	670195
carnitine O-palmitoyltransferase	2.3.1.21	Mouse	7334004
carnitine O-palmitoyltransferase	2.3.1.21	Mouse	7734439
carnitine O-palmitoyltransferase	2.3.1.21	Mouse	8141397
carnitine O-palmitoyltransferase	2.3.1.21	Mouse	8335590
carnitine O-palmitoyltransferase	2.3.1.21	Mouse	8479178
carnitine O-palmitoyltransferase	2.3.1.21	Mouse	8589632
carnitine O-palmitoyltransferase	2.3.1.21	Mouse	8857518
carnitine O-palmitoyltransferase	2.3.1.21	Mouse	9136891
carnitine O-palmitoyltransferase	2.3.1.21	Mouse	9447322
carnitine O-palmitoyltransferase	2.3.1.21	Mouse	9545636
carnitine O-palmitoyltransferase	2.3.1.21	Mouse	9792707
carnitine O-palmitoyltransferase	2.3.1.21	Mouse	9989283
sterol O-acyltransferase	2.3.1.26	Mouse	10593897
sterol O-acyltransferase	2.3.1.26	Mouse	10656290
sterol O-acyltransferase	2.3.1.26	Mouse	11888294
sterol O-acyltransferase	2.3.1.26	Mouse	12787409
sterol O-acyltransferase	2.3.1.26	Mouse	1450216

sterol O-acyltransferase	2.3.1.26	Mouse	2885178
sterol O-acyltransferase	2.3.1.26	Mouse	3593752
sterol O-acyltransferase	2.3.1.26	Mouse	3719008
sterol O-acyltransferase	2.3.1.26	Mouse	3766727
sterol O-acyltransferase	2.3.1.26	Mouse	3812206
sterol O-acyltransferase	2.3.1.26	Mouse	3926761
sterol O-acyltransferase	2.3.1.26	Mouse	4056052
sterol O-acyltransferase	2.3.1.26	Mouse	6347025
sterol O-acyltransferase	2.3.1.26	Mouse	8258956
sterol O-acyltransferase	2.3.1.26	Mouse	8466946
sterol O-acyltransferase	2.3.1.26	Mouse	8820097
sterol O-acyltransferase	2.3.1.26	Mouse	9857049
5-aminolevulinate synthase	2.3.1.37	Mouse	10634305
5-aminolevulinate synthase	2.3.1.37	Mouse	10727444
5-aminolevulinate synthase	2.3.1.37	Mouse	10729988
5-aminolevulinate synthase	2.3.1.37	Mouse	10787385
5-aminolevulinate synthase	2.3.1.37	Mouse	10825473
5-aminolevulinate synthase	2.3.1.37	Mouse	11202048
5-aminolevulinate synthase	2.3.1.37	Mouse	11202050
5-aminolevulinate synthase	2.3.1.37	Mouse	11368326
5-aminolevulinate synthase	2.3.1.37	Mouse	12121995
5-aminolevulinate synthase	2.3.1.37	Mouse	12393745
5-aminolevulinate synthase	2.3.1.37	Mouse	12469218
5-aminolevulinate synthase	2.3.1.37	Mouse	12627002
5-aminolevulinate synthase	2.3.1.37	Mouse	126586
5-aminolevulinate synthase	2.3.1.37	Mouse	12881517
5-aminolevulinate synthase	2.3.1.37	Mouse	14643887
5-aminolevulinate synthase	2.3.1.37	Mouse	1511083
5-aminolevulinate synthase	2.3.1.37	Mouse	15178759
5-aminolevulinate synthase	2.3.1.37	Mouse	15259603
5-aminolevulinate synthase	2.3.1.37	Mouse	1526942
5-aminolevulinate synthase	2.3.1.37	Mouse	15547665
5-aminolevulinate synthase	2.3.1.37	Mouse	15797241
5-aminolevulinate synthase	2.3.1.37	Mouse	15972158
5-aminolevulinate synthase	2.3.1.37	Mouse	16122419
5-aminolevulinate synthase	2.3.1.37	Mouse	16181105
5-aminolevulinate synthase	2.3.1.37	Mouse	16567402
5-aminolevulinate synthase	2.3.1.37	Mouse	16846079
5-aminolevulinate synthase	2.3.1.37	Mouse	1688552
5-aminolevulinate synthase	2.3.1.37	Mouse	16892088
5-aminolevulinate synthase	2.3.1.37	Mouse	16904069
5-aminolevulinate synthase	2.3.1.37	Mouse	1954232
5-aminolevulinate synthase	2.3.1.37	Mouse	1959865

5-aminolevulinate synthase	2.3.1.37	Mouse	2050126
5-aminolevulinate synthase	2.3.1.37	Mouse	2241158
5-aminolevulinate synthase	2.3.1.37	Mouse	2317819
5-aminolevulinate synthase	2.3.1.37	Mouse	266710
5-aminolevulinate synthase	2.3.1.37	Mouse	3009001
5-aminolevulinate synthase	2.3.1.37	Mouse	3094379
5-aminolevulinate synthase	2.3.1.37	Mouse	3359971
5-aminolevulinate synthase	2.3.1.37	Mouse	3755290
5-aminolevulinate synthase	2.3.1.37	Mouse	3840094
5-aminolevulinate synthase	2.3.1.37	Mouse	3966797
5-aminolevulinate synthase	2.3.1.37	Mouse	6092369
5-aminolevulinate synthase	2.3.1.37	Mouse	6213232
5-aminolevulinate synthase	2.3.1.37	Mouse	6547609
5-aminolevulinate synthase	2.3.1.37	Mouse	6824732
5-aminolevulinate synthase	2.3.1.37	Mouse	6873612
5-aminolevulinate synthase	2.3.1.37	Mouse	6954546
5-aminolevulinate synthase	2.3.1.37	Mouse	7093306
5-aminolevulinate synthase	2.3.1.37	Mouse	7592563
5-aminolevulinate synthase	2.3.1.37	Mouse	8076930
5-aminolevulinate synthase	2.3.1.37	Mouse	818637
5-aminolevulinate synthase	2.3.1.37	Mouse	8385933
5-aminolevulinate synthase	2.3.1.37	Mouse	8413301
5-aminolevulinate synthase	2.3.1.37	Mouse	9169013
5-aminolevulinate synthase	2.3.1.37	Mouse	9173682
5-aminolevulinate synthase	2.3.1.37	Mouse	9806796
5-aminolevulinate synthase	2.3.1.37	Mouse	9879810
arylamine N-acetyltransferase	2.3.1.5	Mouse	10100739
arylamine N-acetyltransferase	2.3.1.5	Mouse	10467435
arylamine N-acetyltransferase	2.3.1.5	Mouse	10471401
arylamine N-acetyltransferase	2.3.1.5	Mouse	10721063
arylamine N-acetyltransferase	2.3.1.5	Mouse	10843785
arylamine N-acetyltransferase	2.3.1.5	Mouse	10931207
arylamine N-acetyltransferase	2.3.1.5	Mouse	11122368
arylamine N-acetyltransferase	2.3.1.5	Mouse	12465141
arylamine N-acetyltransferase	2.3.1.5	Mouse	12734775
arylamine N-acetyltransferase	2.3.1.5	Mouse	12736370
arylamine N-acetyltransferase	2.3.1.5	Mouse	12736803
arylamine N-acetyltransferase	2.3.1.5	Mouse	14550904
arylamine N-acetyltransferase	2.3.1.5	Mouse	14578935
arylamine N-acetyltransferase	2.3.1.5	Mouse	15219412
arylamine N-acetyltransferase	2.3.1.5	Mouse	15228600
arylamine N-acetyltransferase	2.3.1.5	Mouse	15782814
arylamine N-acetyltransferase	2.3.1.5	Mouse	15978059

arylamine N-acetyltransferase	2.3.1.5	Mouse	15978063
arylamine N-acetyltransferase	2.3.1.5	Mouse	16095816
arylamine N-acetyltransferase	2.3.1.5	Mouse	1715400
arylamine N-acetyltransferase	2.3.1.5	Mouse	1806289
arylamine N-acetyltransferase	2.3.1.5	Mouse	1829510
arylamine N-acetyltransferase	2.3.1.5	Mouse	1974286
arylamine N-acetyltransferase	2.3.1.5	Mouse	1976757
arylamine N-acetyltransferase	2.3.1.5	Mouse	1977416
arylamine N-acetyltransferase	2.3.1.5	Mouse	2054611
arylamine N-acetyltransferase	2.3.1.5	Mouse	2133350
arylamine N-acetyltransferase	2.3.1.5	Mouse	2167223
arylamine N-acetyltransferase	2.3.1.5	Mouse	3179707
arylamine N-acetyltransferase	2.3.1.5	Mouse	3574290
arylamine N-acetyltransferase	2.3.1.5	Mouse	3702592
arylamine N-acetyltransferase	2.3.1.5	Mouse	7552254
arylamine N-acetyltransferase	2.3.1.5	Mouse	7686576
arylamine N-acetyltransferase	2.3.1.5	Mouse	7741268
arylamine N-acetyltransferase	2.3.1.5	Mouse	8098713
arylamine N-acetyltransferase	2.3.1.5	Mouse	8106958
arylamine N-acetyltransferase	2.3.1.5	Mouse	8176952
arylamine N-acetyltransferase	2.3.1.5	Mouse	8420625
arylamine N-acetyltransferase	2.3.1.5	Mouse	8524412
arylamine N-acetyltransferase	2.3.1.5	Mouse	8550820
arylamine N-acetyltransferase	2.3.1.5	Mouse	8599175
arylamine N-acetyltransferase	2.3.1.5	Mouse	8888105
arylamine N-acetyltransferase	2.3.1.5	Mouse	8930697
arylamine N-acetyltransferase	2.3.1.5	Mouse	8943074
arylamine N-acetyltransferase	2.3.1.5	Mouse	9181516
arylamine N-acetyltransferase	2.3.1.5	Mouse	9238850
arylamine N-acetyltransferase	2.3.1.5	Mouse	9437760
arylamine N-acetyltransferase	2.3.1.5	Mouse	9566052
arylamine N-acetyltransferase	2.3.1.5	Mouse	9597751
arylamine N-acetyltransferase	2.3.1.5	Mouse	9669546
arylamine N-acetyltransferase	2.3.1.5	Mouse	9811631
arylamine N-acetyltransferase	2.3.1.5	Mouse	9949305
serine C-palmitoyltransferase	2.3.1.50	Mouse	10722759
serine C-palmitoyltransferase	2.3.1.50	Mouse	10736421
serine C-palmitoyltransferase	2.3.1.50	Mouse	10764732
serine C-palmitoyltransferase	2.3.1.50	Mouse	10818445
serine C-palmitoyltransferase	2.3.1.50	Mouse	10862608
serine C-palmitoyltransferase	2.3.1.50	Mouse	10971324
serine C-palmitoyltransferase	2.3.1.50	Mouse	11279212
serine C-palmitoyltransferase	2.3.1.50	Mouse	11903061

serine C-palmitoyltransferase	2.3.1.50	Mouse	12445191
serine C-palmitoyltransferase	2.3.1.50	Mouse	12531548
serine C-palmitoyltransferase	2.3.1.50	Mouse	12570999
serine C-palmitoyltransferase	2.3.1.50	Mouse	12612207
serine C-palmitoyltransferase	2.3.1.50	Mouse	12686119
serine C-palmitoyltransferase	2.3.1.50	Mouse	12704216
serine C-palmitoyltransferase	2.3.1.50	Mouse	12736045
serine C-palmitoyltransferase	2.3.1.50	Mouse	12782147
serine C-palmitoyltransferase	2.3.1.50	Mouse	14563682
serine C-palmitoyltransferase	2.3.1.50	Mouse	14744154
serine C-palmitoyltransferase	2.3.1.50	Mouse	15066023
serine C-palmitoyltransferase	2.3.1.50	Mouse	15180163
serine C-palmitoyltransferase	2.3.1.50	Mouse	15297021
serine C-palmitoyltransferase	2.3.1.50	Mouse	15545514
serine C-palmitoyltransferase	2.3.1.50	Mouse	15560753
serine C-palmitoyltransferase	2.3.1.50	Mouse	15562249
serine C-palmitoyltransferase	2.3.1.50	Mouse	16117797
serine C-palmitoyltransferase	2.3.1.50	Mouse	16157870
serine C-palmitoyltransferase	2.3.1.50	Mouse	16210380
serine C-palmitoyltransferase	2.3.1.50	Mouse	16216550
serine C-palmitoyltransferase	2.3.1.50	Mouse	17090526
serine C-palmitoyltransferase	2.3.1.50	Mouse	9186561
serine C-palmitoyltransferase	2.3.1.50	Mouse	9363775
serine C-palmitoyltransferase	2.3.1.50	Mouse	9405408
serine C-palmitoyltransferase	2.3.1.50	Mouse	9714132
serine C-palmitoyltransferase	2.3.1.50	Mouse	9788249
diamine N-acetyltransferase	2.3.1.57	Mouse	10101027
diamine N-acetyltransferase	2.3.1.57	Mouse	10430062
diamine N-acetyltransferase	2.3.1.57	Mouse	11166157
diamine N-acetyltransferase	2.3.1.57	Mouse	11298808
diamine N-acetyltransferase	2.3.1.57	Mouse	11779193
diamine N-acetyltransferase	2.3.1.57	Mouse	12798351
diamine N-acetyltransferase	2.3.1.57	Mouse	12827295
diamine N-acetyltransferase	2.3.1.57	Mouse	1360468
diamine N-acetyltransferase	2.3.1.57	Mouse	1420355
diamine N-acetyltransferase	2.3.1.57	Mouse	15223770
diamine N-acetyltransferase	2.3.1.57	Mouse	15737201
diamine N-acetyltransferase	2.3.1.57	Mouse	1581359
diamine N-acetyltransferase	2.3.1.57	Mouse	1590311
diamine N-acetyltransferase	2.3.1.57	Mouse	16207710
diamine N-acetyltransferase	2.3.1.57	Mouse	16262603
diamine N-acetyltransferase	2.3.1.57	Mouse	16400014
diamine N-acetyltransferase	2.3.1.57	Mouse	16455797

diamine N-acetyltransferase	2.3.1.57	Mouse	2463788
diamine N-acetyltransferase	2.3.1.57	Mouse	2987201
diamine N-acetyltransferase	2.3.1.57	Mouse	3435554
diamine N-acetyltransferase	2.3.1.57	Mouse	3443136
diamine N-acetyltransferase	2.3.1.57	Mouse	3928404
diamine N-acetyltransferase	2.3.1.57	Mouse	4093447
diamine N-acetyltransferase	2.3.1.57	Mouse	6510524
diamine N-acetyltransferase	2.3.1.57	Mouse	6654645
diamine N-acetyltransferase	2.3.1.57	Mouse	7141002
diamine N-acetyltransferase	2.3.1.57	Mouse	7803498
diamine N-acetyltransferase	2.3.1.57	Mouse	8119700
diamine N-acetyltransferase	2.3.1.57	Mouse	8427870
diamine N-acetyltransferase	2.3.1.57	Mouse	8500690
diamine N-acetyltransferase	2.3.1.57	Mouse	8549747
diamine N-acetyltransferase	2.3.1.57	Mouse	8573111
diamine N-acetyltransferase	2.3.1.57	Mouse	8737675
diamine N-acetyltransferase	2.3.1.57	Mouse	8814137
diamine N-acetyltransferase	2.3.1.57	Mouse	8876622
diamine N-acetyltransferase	2.3.1.57	Mouse	8954982
diamine N-acetyltransferase	2.3.1.57	Mouse	9063811
diamine N-acetyltransferase	2.3.1.57	Mouse	9115288
diamine N-acetyltransferase	2.3.1.57	Mouse	9585063
diamine N-acetyltransferase	2.3.1.57	Mouse	9620361
diamine N-acetyltransferase	2.3.1.57	Mouse	9780334
choline O-acetyltransferase	2.3.1.6	Mouse	16480703
choline O-acetyltransferase	2.3.1.6	Mouse	2023918
choline O-acetyltransferase	2.3.1.6	Mouse	2572615
choline O-acetyltransferase	2.3.1.6	Mouse	566752
choline O-acetyltransferase	2.3.1.6	Mouse	7682855
fatty-acid synthase	2.3.1.85	Mouse	10385596
fatty-acid synthase	2.3.1.85	Mouse	11032949
fatty-acid synthase	2.3.1.85	Mouse	12131249
fatty-acid synthase	2.3.1.85	Mouse	12820377
fatty-acid synthase	2.3.1.85	Mouse	14767544
fatty-acid synthase	2.3.1.85	Mouse	15302084
fatty-acid synthase	2.3.1.85	Mouse	15491158
fatty-acid synthase	2.3.1.85	Mouse	15577743
fatty-acid synthase	2.3.1.85	Mouse	16007182
fatty-acid synthase	2.3.1.85	Mouse	16054091
fatty-acid synthase	2.3.1.85	Mouse	16374067
fatty-acid synthase	2.3.1.85	Mouse	16582625
fatty-acid synthase	2.3.1.85	Mouse	16729974
fatty-acid synthase	2.3.1.85	Mouse	7948007

fatty-acid synthase	2.3.1.85	Mouse	9510066
fatty-acid synthase	2.3.1.85	Mouse	9593836
aralkylamine N-acetyltransferase	2.3.1.87	Mouse	10329462
aralkylamine N-acetyltransferase	2.3.1.87	Mouse	10451021
aralkylamine N-acetyltransferase	2.3.1.87	Mouse	10537047
aralkylamine N-acetyltransferase	2.3.1.87	Mouse	10537048
aralkylamine N-acetyltransferase	2.3.1.87	Mouse	10537049
aralkylamine N-acetyltransferase	2.3.1.87	Mouse	11325593
aralkylamine N-acetyltransferase	2.3.1.87	Mouse	11432976
aralkylamine N-acetyltransferase	2.3.1.87	Mouse	11506377
aralkylamine N-acetyltransferase	2.3.1.87	Mouse	12052171
aralkylamine N-acetyltransferase	2.3.1.87	Mouse	12059970
aralkylamine N-acetyltransferase	2.3.1.87	Mouse	14759496
aralkylamine N-acetyltransferase	2.3.1.87	Mouse	15026119
aralkylamine N-acetyltransferase	2.3.1.87	Mouse	15081830
aralkylamine N-acetyltransferase	2.3.1.87	Mouse	15519681
aralkylamine N-acetyltransferase	2.3.1.87	Mouse	15773915
aralkylamine N-acetyltransferase	2.3.1.87	Mouse	15950762
aralkylamine N-acetyltransferase	2.3.1.87	Mouse	16024134
aralkylamine N-acetyltransferase	2.3.1.87	Mouse	16207298
aralkylamine N-acetyltransferase	2.3.1.87	Mouse	16556767
aralkylamine N-acetyltransferase	2.3.1.87	Mouse	16604054
aralkylamine N-acetyltransferase	2.3.1.87	Mouse	16687310
aralkylamine N-acetyltransferase	2.3.1.87	Mouse	16842539
aralkylamine N-acetyltransferase	2.3.1.87	Mouse	16842546
aralkylamine N-acetyltransferase	2.3.1.87	Mouse	16869299
aralkylamine N-acetyltransferase	2.3.1.87	Mouse	16962714
aralkylamine N-acetyltransferase	2.3.1.87	Mouse	1705890
aralkylamine N-acetyltransferase	2.3.1.87	Mouse	503196
aralkylamine N-acetyltransferase	2.3.1.87	Mouse	7498465
aralkylamine N-acetyltransferase	2.3.1.87	Mouse	7566441
aralkylamine N-acetyltransferase	2.3.1.87	Mouse	8674865
aralkylamine N-acetyltransferase	2.3.1.87	Mouse	9605498
aralkylamine N-acetyltransferase	2.3.1.87	Mouse	9703021
aralkylamine N-acetyltransferase	2.3.1.87	Mouse	9708862
acetyl-CoA C-acetyltransferase	2.3.1.9	Mouse	14693556
acetyl-CoA C-acetyltransferase	2.3.1.9	Mouse	15135409
acetyl-CoA C-acetyltransferase	2.3.1.9	Mouse	15466479
acetyl-CoA C-acetyltransferase	2.3.1.9	Mouse	2869784
gamma-glutamyltransferase	2.3.2.2	Mouse	11810401
gamma-glutamyltransferase	2.3.2.2	Mouse	12030366
gamma-glutamyltransferase	2.3.2.2	Mouse	12468440
gamma-glutamyltransferase	2.3.2.2	Mouse	12780970

gamma-glutamyltransferase	2.3.2.2	Mouse	15006645
gamma-glutamyltransferase	2.3.2.2	Mouse	16302185
gamma-glutamyltransferase	2.3.2.2	Mouse	2881890
gamma-glutamyltransferase	2.3.2.2	Mouse	2903803
gamma-glutamyltransferase	2.3.2.2	Mouse	7485380
gamma-glutamyltransferase	2.3.2.2	Mouse	8067452
gamma-glutamyltransferase	2.3.2.2	Mouse	8564390
gamma-glutamyltransferase	2.3.2.2	Mouse	8972486
gamma-glutamyltransferase	2.3.2.2	Mouse	9974125
citrate (Si)-synthase	2.3.3.1	Mouse	1004246
citrate (Si)-synthase	2.3.3.1	Mouse	11842094
citrate (Si)-synthase	2.3.3.1	Mouse	11872452
citrate (Si)-synthase	2.3.3.1	Mouse	15994367
citrate (Si)-synthase	2.3.3.1	Mouse	16269721
citrate (Si)-synthase	2.3.3.1	Mouse	3776117
citrate (Si)-synthase	2.3.3.1	Mouse	3916224
citrate (Si)-synthase	2.3.3.1	Mouse	6799496
citrate (Si)-synthase	2.3.3.1	Mouse	8526514
citrate (Si)-synthase	2.3.3.1	Mouse	9353808
citrate (Si)-synthase	2.3.3.1	Mouse	9554114
hydroxymethylglutaryl-CoA synthase	2.3.3.10	Mouse	11160362
hydroxymethylglutaryl-CoA synthase	2.3.3.10	Mouse	11485325
hydroxymethylglutaryl-CoA synthase	2.3.3.10	Mouse	16101500
hydroxymethylglutaryl-CoA synthase	2.3.3.10	Mouse	16864776
hydroxymethylglutaryl-CoA synthase	2.3.3.10	Mouse	16962226
hydroxymethylglutaryl-CoA synthase	2.3.3.10	Mouse	475
hydroxymethylglutaryl-CoA synthase	2.3.3.10	Mouse	7907092
ATP citrate synthase	2.3.3.8	Mouse	10759520
ATP citrate synthase	2.3.3.8	Mouse	10801800
ATP citrate synthase	2.3.3.8	Mouse	12135479
ATP citrate synthase	2.3.3.8	Mouse	12957888
ATP citrate synthase	2.3.3.8	Mouse	131232
ATP citrate synthase	2.3.3.8	Mouse	16226706
ATP citrate synthase	2.3.3.8	Mouse	16269773
ATP citrate synthase	2.3.3.8	Mouse	16461683
ATP citrate synthase	2.3.3.8	Mouse	16988757
ATP citrate synthase	2.3.3.8	Mouse	17404227
ATP citrate synthase	2.3.3.8	Mouse	2295639
ATP citrate synthase	2.3.3.8	Mouse	8832570
ATP citrate synthase	2.3.3.8	Mouse	9082912
phosphorylase	2.4.1.1	Mouse	10548038
phosphorylase	2.4.1.1	Mouse	11391834
phosphorylase	2.4.1.1	Mouse	11391835

phosphorylase	2.4.1.1	Mouse	11391836
phosphorylase	2.4.1.1	Mouse	11391837
phosphorylase	2.4.1.1	Mouse	11391838
phosphorylase	2.4.1.1	Mouse	11391839
phosphorylase	2.4.1.1	Mouse	11391840
phosphorylase	2.4.1.1	Mouse	11391841
phosphorylase	2.4.1.1	Mouse	11391842
phosphorylase	2.4.1.1	Mouse	12769745
phosphorylase	2.4.1.1	Mouse	15299833
phosphorylase	2.4.1.1	Mouse	15721288
phosphorylase	2.4.1.1	Mouse	1691273
phosphorylase	2.4.1.1	Mouse	7664039
glycogen(starch) synthase	2.4.1.11	Mouse	10067873
glycogen(starch) synthase	2.4.1.11	Mouse	10222257
glycogen(starch) synthase	2.4.1.11	Mouse	10684630
glycogen(starch) synthase	2.4.1.11	Mouse	11181947
glycogen(starch) synthase	2.4.1.11	Mouse	11467410
glycogen(starch) synthase	2.4.1.11	Mouse	11534633
glycogen(starch) synthase	2.4.1.11	Mouse	11834204
glycogen(starch) synthase	2.4.1.11	Mouse	11900279
glycogen(starch) synthase	2.4.1.11	Mouse	12617691
glycogen(starch) synthase	2.4.1.11	Mouse	14570701
glycogen(starch) synthase	2.4.1.11	Mouse	15840572
glycogen(starch) synthase	2.4.1.11	Mouse	15932409
glycogen(starch) synthase	2.4.1.11	Mouse	16101290
glycogen(starch) synthase	2.4.1.11	Mouse	1756915
glycogen(starch) synthase	2.4.1.11	Mouse	17569761
glycogen(starch) synthase	2.4.1.11	Mouse	17698598
glycogen(starch) synthase	2.4.1.11	Mouse	1959479
glycogen(starch) synthase	2.4.1.11	Mouse	208368
glycogen(starch) synthase	2.4.1.11	Mouse	2115296
glycogen(starch) synthase	2.4.1.11	Mouse	2154910
glycogen(starch) synthase	2.4.1.11	Mouse	219866
glycogen(starch) synthase	2.4.1.11	Mouse	227915
glycogen(starch) synthase	2.4.1.11	Mouse	2405698
glycogen(starch) synthase	2.4.1.11	Mouse	2822414
glycogen(starch) synthase	2.4.1.11	Mouse	3032541
glycogen(starch) synthase	2.4.1.11	Mouse	3092743
glycogen(starch) synthase	2.4.1.11	Mouse	3143265
glycogen(starch) synthase	2.4.1.11	Mouse	3930321
glycogen(starch) synthase	2.4.1.11	Mouse	6409592
glycogen(starch) synthase	2.4.1.11	Mouse	6412593
glycogen(starch) synthase	2.4.1.11	Mouse	7010073

glycogen(starch) synthase	2.4.1.11	Mouse	7672505
glycogen(starch) synthase	2.4.1.11	Mouse	7983805
glycogen(starch) synthase	2.4.1.11	Mouse	8226927
glycogen(starch) synthase	2.4.1.11	Mouse	8416266
glycogen(starch) synthase	2.4.1.11	Mouse	8514767
glycogen(starch) synthase	2.4.1.11	Mouse	8514849
glycogen(starch) synthase	2.4.1.11	Mouse	8541012
glycogen(starch) synthase	2.4.1.11	Mouse	8569754
glycogen(starch) synthase	2.4.1.11	Mouse	8591890
glycogen(starch) synthase	2.4.1.11	Mouse	8593937
glycogen(starch) synthase	2.4.1.11	Mouse	8612539
glycogen(starch) synthase	2.4.1.11	Mouse	8645005
glycogen(starch) synthase	2.4.1.11	Mouse	8721777
glycogen(starch) synthase	2.4.1.11	Mouse	8769349
glycogen(starch) synthase	2.4.1.11	Mouse	9126490
glycogen(starch) synthase	2.4.1.11	Mouse	9162607
glycogen(starch) synthase	2.4.1.11	Mouse	9267990
glycogen(starch) synthase	2.4.1.11	Mouse	9389424
glycogen(starch) synthase	2.4.1.11	Mouse	9450985
glycogen(starch) synthase	2.4.1.11	Mouse	9609122
glycogen(starch) synthase	2.4.1.11	Mouse	9712712
alpha-1,6-mannosyl-glycoprotein	2.4.1.143	Mouse	11250723
alpha-1,6-mannosyl-glycoprotein	2.4.1.143	Mouse	11552947
alpha-1,6-mannosyl-glycoprotein	2.4.1.143	Mouse	7841796
alpha-1,6-mannosyl-glycoprotein	2.4.1.143	Mouse	9028721
glucuronosyltransferase	2.4.1.17	Mouse	10353933
glucuronosyltransferase	2.4.1.17	Mouse	10427418
glucuronosyltransferase	2.4.1.17	Mouse	15710570
glucuronosyltransferase	2.4.1.17	Mouse	16623861
glucuronosyltransferase	2.4.1.17	Mouse	4715994
glucuronosyltransferase	2.4.1.17	Mouse	7900959
lactosylceramide 1,3-N-acetyl-beta-D-glucosaminyltransferase	2.4.1.206	Mouse	2564417
lactose synthase	2.4.1.22	Mouse	16664466
lactose synthase	2.4.1.22	Mouse	6766957
lactose synthase	2.4.1.22	Mouse	7539442
lactose synthase	2.4.1.22	Mouse	9387870
amidophosphoribosyltransferase	2.4.2.14	Mouse	10675983
amidophosphoribosyltransferase	2.4.2.14	Mouse	11158364
amidophosphoribosyltransferase	2.4.2.14	Mouse	12930749
amidophosphoribosyltransferase	2.4.2.14	Mouse	15266056
amidophosphoribosyltransferase	2.4.2.14	Mouse	17434429
amidophosphoribosyltransferase	2.4.2.14	Mouse	214373

amidophosphoribosyltransferase	2.4.2.14	Mouse	6327016
amidophosphoribosyltransferase	2.4.2.14	Mouse	701284
amidophosphoribosyltransferase	2.4.2.14	Mouse	7683680
amidophosphoribosyltransferase	2.4.2.14	Mouse	8150282
amidophosphoribosyltransferase	2.4.2.14	Mouse	8197456
amidophosphoribosyltransferase	2.4.2.14	Mouse	8197457
amidophosphoribosyltransferase	2.4.2.14	Mouse	8197458
amidophosphoribosyltransferase	2.4.2.14	Mouse	8380692
amidophosphoribosyltransferase	2.4.2.14	Mouse	8463258
amidophosphoribosyltransferase	2.4.2.14	Mouse	8809759
amidophosphoribosyltransferase	2.4.2.14	Mouse	8976092
amidophosphoribosyltransferase	2.4.2.14	Mouse	9615746
amidophosphoribosyltransferase	2.4.2.14	Mouse	9881055
thymidine phosphorylase	2.4.2.4	Mouse	10853015
thymidine phosphorylase	2.4.2.4	Mouse	11530879
thymidine phosphorylase	2.4.2.4	Mouse	11585972
thymidine phosphorylase	2.4.2.4	Mouse	12680231
thymidine phosphorylase	2.4.2.4	Mouse	15134221
thymidine phosphorylase	2.4.2.4	Mouse	15289834
thymidine phosphorylase	2.4.2.4	Mouse	15917420
thymidine phosphorylase	2.4.2.4	Mouse	16302736
adenine phosphoribosyltransferase	2.4.2.7	Mouse	2154328
adenine phosphoribosyltransferase	2.4.2.7	Mouse	6327016
dimethylallyltranstransferase	2.5.1.1	Mouse	11442630
dimethylallyltranstransferase	2.5.1.1	Mouse	14512521
dimethylallyltranstransferase	2.5.1.1	Mouse	15597200
dimethylallyltranstransferase	2.5.1.1	Mouse	7697819
dimethylallyltranstransferase	2.5.1.1	Mouse	7843406
dimethylallyltranstransferase	2.5.1.1	Mouse	8631820
geranyltranstransferase	2.5.1.10	Mouse	10484604
geranyltranstransferase	2.5.1.10	Mouse	11202437
geranyltranstransferase	2.5.1.10	Mouse	15459425
geranyltranstransferase	2.5.1.10	Mouse	15605175
geranyltranstransferase	2.5.1.10	Mouse	15713990
geranyltranstransferase	2.5.1.10	Mouse	15827605
geranyltranstransferase	2.5.1.10	Mouse	15827618
geranyltranstransferase	2.5.1.10	Mouse	16179378
geranyltranstransferase	2.5.1.10	Mouse	16932286
geranyltranstransferase	2.5.1.10	Mouse	1779710
geranyltranstransferase	2.5.1.10	Mouse	9061016
geranyltranstransferase	2.5.1.10	Mouse	9640665
spermidine synthase	2.5.1.16	Mouse	16515550
spermidine synthase	2.5.1.16	Mouse	2775206

glutathione transferase	2.5.1.18	Mouse	12484753
glutathione transferase	2.5.1.18	Mouse	15604283
glutathione transferase	2.5.1.18	Mouse	15761769
glutathione transferase	2.5.1.18	Mouse	16328982
glutathione transferase	2.5.1.18	Mouse	17176043
glutathione transferase	2.5.1.18	Mouse	17397868
squalene synthase	2.5.1.21	Mouse	10649449
squalene synthase	2.5.1.21	Mouse	10677224
squalene synthase	2.5.1.21	Mouse	12114564
squalene synthase	2.5.1.21	Mouse	15356323
squalene synthase	2.5.1.21	Mouse	1601846
squalene synthase	2.5.1.21	Mouse	17016471
squalene synthase	2.5.1.21	Mouse	17531951
squalene synthase	2.5.1.21	Mouse	2068081
squalene synthase	2.5.1.21	Mouse	7766395
squalene synthase	2.5.1.21	Mouse	7843406
squalene synthase	2.5.1.21	Mouse	9070296
methionine adenosyltransferase	2.5.1.6	Mouse	10415148
methionine adenosyltransferase	2.5.1.6	Mouse	1511738
methionine adenosyltransferase	2.5.1.6	Mouse	16413417
methionine adenosyltransferase	2.5.1.6	Mouse	2764959
methionine adenosyltransferase	2.5.1.6	Mouse	7980467
hydroxymethylbilane synthase	2.5.1.61	Mouse	10546563
hydroxymethylbilane synthase	2.5.1.61	Mouse	10787385
hydroxymethylbilane synthase	2.5.1.61	Mouse	11953837
hydroxymethylbilane synthase	2.5.1.61	Mouse	14559249
hydroxymethylbilane synthase	2.5.1.61	Mouse	1522882
hydroxymethylbilane synthase	2.5.1.61	Mouse	16886091
hydroxymethylbilane synthase	2.5.1.61	Mouse	4067519
hydroxymethylbilane synthase	2.5.1.61	Mouse	7326026
hydroxymethylbilane synthase	2.5.1.61	Mouse	7682572
hydroxymethylbilane synthase	2.5.1.61	Mouse	8023693
hydroxymethylbilane synthase	2.5.1.61	Mouse	9065797
hydroxymethylbilane synthase	2.5.1.61	Mouse	9460994
aspartate transaminase	2.6.1.1	Mouse	8580353
ornithine aminotransferase	2.6.1.13	Mouse	11691635
ornithine aminotransferase	2.6.1.13	Mouse	12462748
ornithine aminotransferase	2.6.1.13	Mouse	7883744
glutamine---fructose-6-phosphate transaminase (isomerizing)	2.6.1.16	Mouse	10329452
glutamine---fructose-6-phosphate transaminase (isomerizing)	2.6.1.16	Mouse	10865863

glutamine---fructose-6-phosphate transaminase (isomerizing)	2.6.1.16	Mouse	11270676
glutamine---fructose-6-phosphate transaminase (isomerizing)	2.6.1.16	Mouse	11895440
glutamine---fructose-6-phosphate transaminase (isomerizing)	2.6.1.16	Mouse	15158264
glutamine---fructose-6-phosphate transaminase (isomerizing)	2.6.1.16	Mouse	15308130
glutamine---fructose-6-phosphate transaminase (isomerizing)	2.6.1.16	Mouse	15613679
glutamine---fructose-6-phosphate transaminase (isomerizing)	2.6.1.16	Mouse	17941647
glutamine---fructose-6-phosphate transaminase (isomerizing)	2.6.1.16	Mouse	6184359
glutamine---fructose-6-phosphate transaminase (isomerizing)	2.6.1.16	Mouse	8394312
glutamine---fructose-6-phosphate transaminase (isomerizing)	2.6.1.16	Mouse	9421478
tyrosine transaminase	2.6.1.5	Mouse	1348057
tyrosine transaminase	2.6.1.5	Mouse	1526942
tyrosine transaminase	2.6.1.5	Mouse	240411
tyrosine transaminase	2.6.1.5	Mouse	2870018
tyrosine transaminase	2.6.1.5	Mouse	6123525
tyrosine transaminase	2.6.1.5	Mouse	9089286
tyrosine transaminase	2.6.1.5	Mouse	9228277
hexokinase	2.7.1.1	Mouse	11319725
hexokinase	2.7.1.1	Mouse	11391834
hexokinase	2.7.1.1	Mouse	11828256
hexokinase	2.7.1.1	Mouse	12660493
hexokinase	2.7.1.1	Mouse	1331693
hexokinase	2.7.1.1	Mouse	14672622
hexokinase	2.7.1.1	Mouse	15607940
hexokinase	2.7.1.1	Mouse	6341787
hexokinase	2.7.1.1	Mouse	6363888
hexokinase	2.7.1.1	Mouse	6440018
hexokinase	2.7.1.1	Mouse	6993859
hexokinase	2.7.1.1	Mouse	8027295
hexokinase	2.7.1.1	Mouse	9523722
6-phosphofructokinase	2.7.1.11	Mouse	10323269
6-phosphofructokinase	2.7.1.11	Mouse	10444344
6-phosphofructokinase	2.7.1.11	Mouse	10742704
6-phosphofructokinase	2.7.1.11	Mouse	10909961
6-phosphofructokinase	2.7.1.11	Mouse	10931197

6-phosphofructokinase	2.7.1.11	Mouse	1100622
6-phosphofructokinase	2.7.1.11	Mouse	1100623
6-phosphofructokinase	2.7.1.11	Mouse	11014908
6-phosphofructokinase	2.7.1.11	Mouse	11045948
6-phosphofructokinase	2.7.1.11	Mouse	11058792
6-phosphofructokinase	2.7.1.11	Mouse	11391835
6-phosphofructokinase	2.7.1.11	Mouse	11391836
6-phosphofructokinase	2.7.1.11	Mouse	11391837
6-phosphofructokinase	2.7.1.11	Mouse	11560513
6-phosphofructokinase	2.7.1.11	Mouse	12023862
6-phosphofructokinase	2.7.1.11	Mouse	12051897
6-phosphofructokinase	2.7.1.11	Mouse	12125051
6-phosphofructokinase	2.7.1.11	Mouse	12453221
6-phosphofructokinase	2.7.1.11	Mouse	131232
6-phosphofructokinase	2.7.1.11	Mouse	14585511
6-phosphofructokinase	2.7.1.11	Mouse	147929
6-phosphofructokinase	2.7.1.11	Mouse	149128
6-phosphofructokinase	2.7.1.11	Mouse	15157773
6-phosphofructokinase	2.7.1.11	Mouse	15466668
6-phosphofructokinase	2.7.1.11	Mouse	15504384
6-phosphofructokinase	2.7.1.11	Mouse	156307
6-phosphofructokinase	2.7.1.11	Mouse	15991998
6-phosphofructokinase	2.7.1.11	Mouse	16088331
6-phosphofructokinase	2.7.1.11	Mouse	16103521
6-phosphofructokinase	2.7.1.11	Mouse	16115917
6-phosphofructokinase	2.7.1.11	Mouse	16346876
6-phosphofructokinase	2.7.1.11	Mouse	16377227
6-phosphofructokinase	2.7.1.11	Mouse	1658253
6-phosphofructokinase	2.7.1.11	Mouse	16593209
6-phosphofructokinase	2.7.1.11	Mouse	1825156
6-phosphofructokinase	2.7.1.11	Mouse	1828673
6-phosphofructokinase	2.7.1.11	Mouse	1830744
6-phosphofructokinase	2.7.1.11	Mouse	1833303
6-phosphofructokinase	2.7.1.11	Mouse	191426
6-phosphofructokinase	2.7.1.11	Mouse	2137204
6-phosphofructokinase	2.7.1.11	Mouse	2147292
6-phosphofructokinase	2.7.1.11	Mouse	2149746
6-phosphofructokinase	2.7.1.11	Mouse	2434517
6-phosphofructokinase	2.7.1.11	Mouse	2502581
6-phosphofructokinase	2.7.1.11	Mouse	2522395
6-phosphofructokinase	2.7.1.11	Mouse	2527305
6-phosphofructokinase	2.7.1.11	Mouse	2820531
6-phosphofructokinase	2.7.1.11	Mouse	28629

6-phosphofructokinase	2.7.1.11	Mouse	2933146
6-phosphofructokinase	2.7.1.11	Mouse	2935776
6-phosphofructokinase	2.7.1.11	Mouse	2938549
6-phosphofructokinase	2.7.1.11	Mouse	2956156
6-phosphofructokinase	2.7.1.11	Mouse	2963653
6-phosphofructokinase	2.7.1.11	Mouse	2972577
6-phosphofructokinase	2.7.1.11	Mouse	2981949
6-phosphofructokinase	2.7.1.11	Mouse	3364152
6-phosphofructokinase	2.7.1.11	Mouse	3407760
6-phosphofructokinase	2.7.1.11	Mouse	356174
6-phosphofructokinase	2.7.1.11	Mouse	3768440
6-phosphofructokinase	2.7.1.11	Mouse	3931461
6-phosphofructokinase	2.7.1.11	Mouse	4030556
6-phosphofructokinase	2.7.1.11	Mouse	4243437
6-phosphofructokinase	2.7.1.11	Mouse	4252961
6-phosphofructokinase	2.7.1.11	Mouse	6093562
6-phosphofructokinase	2.7.1.11	Mouse	6133774
6-phosphofructokinase	2.7.1.11	Mouse	6211175
6-phosphofructokinase	2.7.1.11	Mouse	6231923
6-phosphofructokinase	2.7.1.11	Mouse	6232272
6-phosphofructokinase	2.7.1.11	Mouse	6234885
6-phosphofructokinase	2.7.1.11	Mouse	6279392
6-phosphofructokinase	2.7.1.11	Mouse	6325266
6-phosphofructokinase	2.7.1.11	Mouse	6331422
6-phosphofructokinase	2.7.1.11	Mouse	6440018
6-phosphofructokinase	2.7.1.11	Mouse	6444231
6-phosphofructokinase	2.7.1.11	Mouse	6452426
6-phosphofructokinase	2.7.1.11	Mouse	6779470
6-phosphofructokinase	2.7.1.11	Mouse	7233512
6-phosphofructokinase	2.7.1.11	Mouse	7440254
6-phosphofructokinase	2.7.1.11	Mouse	7522206
6-phosphofructokinase	2.7.1.11	Mouse	7589825
6-phosphofructokinase	2.7.1.11	Mouse	7602786
6-phosphofructokinase	2.7.1.11	Mouse	7710770
6-phosphofructokinase	2.7.1.11	Mouse	7875554
6-phosphofructokinase	2.7.1.11	Mouse	8224738
6-phosphofructokinase	2.7.1.11	Mouse	8366430
6-phosphofructokinase	2.7.1.11	Mouse	8514849
6-phosphofructokinase	2.7.1.11	Mouse	8557664
6-phosphofructokinase	2.7.1.11	Mouse	8593533
6-phosphofructokinase	2.7.1.11	Mouse	8643924
6-phosphofructokinase	2.7.1.11	Mouse	8910548
6-phosphofructokinase	2.7.1.11	Mouse	8981075

6-phosphofructokinase	2.7.1.11	Mouse	9267516
6-phosphofructokinase	2.7.1.11	Mouse	9287040
6-phosphofructokinase	2.7.1.11	Mouse	9329694
6-phosphofructokinase	2.7.1.11	Mouse	9371084
6-phosphofructokinase	2.7.1.11	Mouse	9439886
6-phosphofructokinase	2.7.1.11	Mouse	9447322
6-phosphofructokinase	2.7.1.11	Mouse	9555897
6-phosphofructokinase	2.7.1.11	Mouse	9580251
6-phosphofructokinase	2.7.1.11	Mouse	9580875
6-phosphofructokinase	2.7.1.11	Mouse	9608547
6-phosphofructokinase	2.7.1.11	Mouse	9766212
6-phosphofructokinase	2.7.1.11	Mouse	9777012
6-phosphofructokinase	2.7.1.11	Mouse	9851886
6-phosphofructokinase	2.7.1.11	Mouse	9973548
inositol-trisphosphate 3-kinase	2.7.1.127	Mouse	15837423
inositol-trisphosphate 3-kinase	2.7.1.127	Mouse	9211876
inositol-tetrakisphosphate 1-kinase	2.7.1.134	Mouse	11533064
inositol-tetrakisphosphate 1-kinase	2.7.1.134	Mouse	15837423
phosphatidylinositol 3-kinase	2.7.1.137	Mouse	10869418
phosphatidylinositol 3-kinase	2.7.1.137	Mouse	10874027
phosphatidylinositol 3-kinase	2.7.1.137	Mouse	10998146
phosphatidylinositol 3-kinase	2.7.1.137	Mouse	11259761
phosphatidylinositol 3-kinase	2.7.1.137	Mouse	11744698
phosphatidylinositol 3-kinase	2.7.1.137	Mouse	7669049
phosphatidylinositol 3-kinase	2.7.1.137	Mouse	9292730
phosphatidylinositol 3-kinase	2.7.1.137	Mouse	9312149
phosphatidylinositol 3-kinase	2.7.1.137	Mouse	9478990
phosphatidylinositol 3-kinase	2.7.1.137	Mouse	9826526
phosphatidylinositol 3-kinase	2.7.1.137	Mouse	9826674
glucokinase	2.7.1.2	Mouse	10456334
glucokinase	2.7.1.2	Mouse	10494657
glucokinase	2.7.1.2	Mouse	10905475
glucokinase	2.7.1.2	Mouse	11311143
glucokinase	2.7.1.2	Mouse	11947549
glucokinase	2.7.1.2	Mouse	11950391
glucokinase	2.7.1.2	Mouse	12941786
glucokinase	2.7.1.2	Mouse	14979565
glucokinase	2.7.1.2	Mouse	15009676
glucokinase	2.7.1.2	Mouse	15016359
glucokinase	2.7.1.2	Mouse	15134337
glucokinase	2.7.1.2	Mouse	15226592
glucokinase	2.7.1.2	Mouse	15277402
glucokinase	2.7.1.2	Mouse	1545870

glucokinase	2.7.1.2	Mouse	15707679
glucokinase	2.7.1.2	Mouse	15955369
glucokinase	2.7.1.2	Mouse	16186394
glucokinase	2.7.1.2	Mouse	16834571
glucokinase	2.7.1.2	Mouse	16899262
glucokinase	2.7.1.2	Mouse	16916947
glucokinase	2.7.1.2	Mouse	2210070
glucokinase	2.7.1.2	Mouse	2584235
glucokinase	2.7.1.2	Mouse	2682629
glucokinase	2.7.1.2	Mouse	6780351
glucokinase	2.7.1.2	Mouse	6836273
glucokinase	2.7.1.2	Mouse	7010073
glucokinase	2.7.1.2	Mouse	7553875
glucokinase	2.7.1.2	Mouse	7821741
glucokinase	2.7.1.2	Mouse	7983782
glucokinase	2.7.1.2	Mouse	8194664
glucokinase	2.7.1.2	Mouse	8344416
glucokinase	2.7.1.2	Mouse	8433729
glucokinase	2.7.1.2	Mouse	8446591
glucokinase	2.7.1.2	Mouse	8549869
glucokinase	2.7.1.2	Mouse	8550593
glucokinase	2.7.1.2	Mouse	8631975
glucokinase	2.7.1.2	Mouse	8690154
glucokinase	2.7.1.2	Mouse	8692940
glucokinase	2.7.1.2	Mouse	8751724
glucokinase	2.7.1.2	Mouse	9113996
glucokinase	2.7.1.2	Mouse	9460079
adenosine kinase	2.7.1.20	Mouse	10794412
adenosine kinase	2.7.1.20	Mouse	12228764
adenosine kinase	2.7.1.20	Mouse	15632276
adenosine kinase	2.7.1.20	Mouse	2154328
adenosine kinase	2.7.1.20	Mouse	8184939
thymidine kinase	2.7.1.21	Mouse	12750029
thymidine kinase	2.7.1.21	Mouse	12750297
thymidine kinase	2.7.1.21	Mouse	1337357
thymidine kinase	2.7.1.21	Mouse	1455450
thymidine kinase	2.7.1.21	Mouse	14770427
thymidine kinase	2.7.1.21	Mouse	15196542
thymidine kinase	2.7.1.21	Mouse	1525336
thymidine kinase	2.7.1.21	Mouse	1531285
thymidine kinase	2.7.1.21	Mouse	15809747
thymidine kinase	2.7.1.21	Mouse	16336273
thymidine kinase	2.7.1.21	Mouse	16473525

thymidine kinase	2.7.1.21	Mouse	17065087
thymidine kinase	2.7.1.21	Mouse	2025497
thymidine kinase	2.7.1.21	Mouse	348202
thymidine kinase	2.7.1.21	Mouse	3558173
thymidine kinase	2.7.1.21	Mouse	6684556
thymidine kinase	2.7.1.21	Mouse	6840218
thymidine kinase	2.7.1.21	Mouse	8016290
thymidine kinase	2.7.1.21	Mouse	8140585
thymidine kinase	2.7.1.21	Mouse	8432201
thymidine kinase	2.7.1.21	Mouse	8878781
thymidine kinase	2.7.1.21	Mouse	8941385
thymidine kinase	2.7.1.21	Mouse	8955897
thymidine kinase	2.7.1.21	Mouse	9154996
thymidine kinase	2.7.1.21	Mouse	956273
thymidine kinase	2.7.1.21	Mouse	9661884
thymidine kinase	2.7.1.21	Mouse	9816259
riboflavin kinase	2.7.1.26	Mouse	6138398
glycerol kinase	2.7.1.30	Mouse	10.1002/anie.198810401
glycerol kinase	2.7.1.30	Mouse	11388799
glycerol kinase	2.7.1.30	Mouse	11811537
glycerol kinase	2.7.1.30	Mouse	1985967
glycerol kinase	2.7.1.30	Mouse	2547969
glycerol kinase	2.7.1.30	Mouse	4914079
glycerol kinase	2.7.1.30	Mouse	6292169
glycerol kinase	2.7.1.30	Mouse	6440018
glycerol kinase	2.7.1.30	Mouse	9162046
choline kinase	2.7.1.32	Mouse	1336121
choline kinase	2.7.1.32	Mouse	16490392
choline kinase	2.7.1.32	Mouse	2153442
choline kinase	2.7.1.32	Mouse	217369
choline kinase	2.7.1.32	Mouse	3365445
choline kinase	2.7.1.32	Mouse	3447597
choline kinase	2.7.1.32	Mouse	5495730
choline kinase	2.7.1.32	Mouse	6503617
choline kinase	2.7.1.32	Mouse	8182083
choline kinase	2.7.1.32	Mouse	828054
choline kinase	2.7.1.32	Mouse	8414498
pantothenate kinase	2.7.1.33	Mouse	10625688
pantothenate kinase	2.7.1.33	Mouse	11809413
pantothenate kinase	2.7.1.33	Mouse	12697433
pantothenate kinase	2.7.1.33	Mouse	15176870
pantothenate kinase	2.7.1.33	Mouse	15843025
pantothenate kinase	2.7.1.33	Mouse	16701556

pantothenate kinase	2.7.1.33	Mouse	17323930
pantothenate kinase	2.7.1.33	Mouse	17581817
pantothenate kinase	2.7.1.33	Mouse	9890959
pyruvate kinase	2.7.1.40	Mouse	101523
pyruvate kinase	2.7.1.40	Mouse	11181519
pyruvate kinase	2.7.1.40	Mouse	1175605
pyruvate kinase	2.7.1.40	Mouse	1328007
pyruvate kinase	2.7.1.40	Mouse	1406667
pyruvate kinase	2.7.1.40	Mouse	15028426
pyruvate kinase	2.7.1.40	Mouse	15567985
pyruvate kinase	2.7.1.40	Mouse	16046853
pyruvate kinase	2.7.1.40	Mouse	16511150
pyruvate kinase	2.7.1.40	Mouse	16549526
pyruvate kinase	2.7.1.40	Mouse	1959479
pyruvate kinase	2.7.1.40	Mouse	2387024
pyruvate kinase	2.7.1.40	Mouse	2813362
pyruvate kinase	2.7.1.40	Mouse	2820531
pyruvate kinase	2.7.1.40	Mouse	2846196
pyruvate kinase	2.7.1.40	Mouse	291050
pyruvate kinase	2.7.1.40	Mouse	29278
pyruvate kinase	2.7.1.40	Mouse	2935776
pyruvate kinase	2.7.1.40	Mouse	2970638
pyruvate kinase	2.7.1.40	Mouse	3023262
pyruvate kinase	2.7.1.40	Mouse	3032541
pyruvate kinase	2.7.1.40	Mouse	3159473
pyruvate kinase	2.7.1.40	Mouse	3161219
pyruvate kinase	2.7.1.40	Mouse	3350145
pyruvate kinase	2.7.1.40	Mouse	4053567
pyruvate kinase	2.7.1.40	Mouse	6222515
pyruvate kinase	2.7.1.40	Mouse	6241274
pyruvate kinase	2.7.1.40	Mouse	6268138
pyruvate kinase	2.7.1.40	Mouse	6370232
pyruvate kinase	2.7.1.40	Mouse	6588273
pyruvate kinase	2.7.1.40	Mouse	6682991
pyruvate kinase	2.7.1.40	Mouse	6713301
pyruvate kinase	2.7.1.40	Mouse	7357032
pyruvate kinase	2.7.1.40	Mouse	7961441
pyruvate kinase	2.7.1.40	Mouse	8074527
pyruvate kinase	2.7.1.40	Mouse	8144600
pyruvate kinase	2.7.1.40	Mouse	8436141
pyruvate kinase	2.7.1.40	Mouse	8476115
pyruvate kinase	2.7.1.40	Mouse	8765986
pyruvate kinase	2.7.1.40	Mouse	9252361

uridine kinase	2.7.1.48	Mouse	15735337
uridine kinase	2.7.1.48	Mouse	195585
uridine kinase	2.7.1.48	Mouse	9923963
galactokinase	2.7.1.6	Mouse	12694189
galactokinase	2.7.1.6	Mouse	14596685
galactokinase	2.7.1.6	Mouse	14763977
galactokinase	2.7.1.6	Mouse	16452467
galactokinase	2.7.1.6	Mouse	6836273
1-phosphatidylinositol 4-kinase	2.7.1.67	Mouse	12594831
1-phosphatidylinositol 4-kinase	2.7.1.67	Mouse	12620118
1-phosphatidylinositol 4-kinase	2.7.1.67	Mouse	16912074
1-phosphatidylinositol 4-kinase	2.7.1.67	Mouse	17003043
1-phosphatidylinositol 4-kinase	2.7.1.67	Mouse	7961848
1-phosphatidylinositol 4-kinase	2.7.1.67	Mouse	8152413
1-phosphatidylinositol 4-kinase	2.7.1.67	Mouse	8190262
1-phosphatidylinositol 4-kinase	2.7.1.67	Mouse	9654085
1-phosphatidylinositol 4-kinase	2.7.1.67	Mouse	9854149
1-phosphatidylinositol 4-kinase	2.7.1.67	Mouse	9891985
1-phosphatidylinositol-4-phosphate 5-kinase	2.7.1.68	Mouse	11098053
1-phosphatidylinositol-4-phosphate 5-kinase	2.7.1.68	Mouse	12620118
1-phosphatidylinositol-4-phosphate 5-kinase	2.7.1.68	Mouse	15277528
1-phosphatidylinositol-4-phosphate 5-kinase	2.7.1.68	Mouse	15738269
1-phosphatidylinositol-4-phosphate 5-kinase	2.7.1.68	Mouse	17635937
1-phosphatidylinositol-4-phosphate 5-kinase	2.7.1.68	Mouse	2849321
1-phosphatidylinositol-4-phosphate 5-kinase	2.7.1.68	Mouse	8190262
1-phosphatidylinositol-4-phosphate 5-kinase	2.7.1.68	Mouse	9292730
deoxycytidine kinase	2.7.1.74	Mouse	10499616
deoxycytidine kinase	2.7.1.74	Mouse	10848830
deoxycytidine kinase	2.7.1.74	Mouse	11888330
deoxycytidine kinase	2.7.1.74	Mouse	12054682
deoxycytidine kinase	2.7.1.74	Mouse	15561147
deoxycytidine kinase	2.7.1.74	Mouse	15803490
deoxycytidine kinase	2.7.1.74	Mouse	16180016
deoxycytidine kinase	2.7.1.74	Mouse	16421443
deoxycytidine kinase	2.7.1.74	Mouse	16463058
deoxycytidine kinase	2.7.1.74	Mouse	17065079
deoxycytidine kinase	2.7.1.74	Mouse	2436757
deoxycytidine kinase	2.7.1.74	Mouse	3335008
deoxycytidine kinase	2.7.1.74	Mouse	7805176
deoxycytidine kinase	2.7.1.74	Mouse	8616717
deoxycytidine kinase	2.7.1.74	Mouse	8718419
ethanolamine kinase	2.7.1.82	Mouse	11044454
ethanolamine kinase	2.7.1.82	Mouse	1480155

ethanolamine kinase	2.7.1.82	Mouse	475777
ethanolamine kinase	2.7.1.82	Mouse	7142139
sphinganine kinase	2.7.1.91	Mouse	10567432
sphinganine kinase	2.7.1.91	Mouse	14568343
sphinganine kinase	2.7.1.91	Mouse	15451787
sphinganine kinase	2.7.1.91	Mouse	16831409
glutamate 5-kinase	2.7.2.11	Mouse	12602867
glutamate 5-kinase	2.7.2.11	Mouse	15077666
glutamate 5-kinase	2.7.2.11	Mouse	17449694
adenylate kinase	2.7.4.3	Mouse	126626
adenylate kinase	2.7.4.3	Mouse	14656997
adenylate kinase	2.7.4.3	Mouse	15941717
adenylate kinase	2.7.4.3	Mouse	16668787
adenylate kinase	2.7.4.3	Mouse	6440018
adenylate kinase	2.7.4.3	Mouse	7764491
dTMP kinase	2.7.4.9	Mouse	164949
ribose-phosphate diphosphokinase	2.7.6.1	Mouse	15878857
ribose-phosphate diphosphokinase	2.7.6.1	Mouse	217337
nicotinamide-nucleotide adenylyltransferase	2.7.7.1	Mouse	11248244
nicotinamide-nucleotide adenylyltransferase	2.7.7.1	Mouse	11966442
nicotinamide-nucleotide adenylyltransferase	2.7.7.1	Mouse	17360427
ethanolamine-phosphate cytidylyltransferase	2.7.7.14	Mouse	10493918
ethanolamine-phosphate cytidylyltransferase	2.7.7.14	Mouse	10752579
ethanolamine-phosphate cytidylyltransferase	2.7.7.14	Mouse	11097182
ethanolamine-phosphate cytidylyltransferase	2.7.7.14	Mouse	11844611
ethanolamine-phosphate cytidylyltransferase	2.7.7.14	Mouse	14697519
ethanolamine-phosphate cytidylyltransferase	2.7.7.14	Mouse	15147238
ethanolamine-phosphate cytidylyltransferase	2.7.7.14	Mouse	475777
ethanolamine-phosphate cytidylyltransferase	2.7.7.14	Mouse	603639
ethanolamine-phosphate cytidylyltransferase	2.7.7.14	Mouse	6626563
choline-phosphate cytidylyltransferase	2.7.7.15	Mouse	10101264
choline-phosphate cytidylyltransferase	2.7.7.15	Mouse	10208837
choline-phosphate cytidylyltransferase	2.7.7.15	Mouse	10473578
choline-phosphate cytidylyltransferase	2.7.7.15	Mouse	10615073
choline-phosphate cytidylyltransferase	2.7.7.15	Mouse	10908674
choline-phosphate cytidylyltransferase	2.7.7.15	Mouse	10946015
choline-phosphate cytidylyltransferase	2.7.7.15	Mouse	11029581
choline-phosphate cytidylyltransferase	2.7.7.15	Mouse	11097182
choline-phosphate cytidylyltransferase	2.7.7.15	Mouse	11279002
choline-phosphate cytidylyltransferase	2.7.7.15	Mouse	11404252
choline-phosphate cytidylyltransferase	2.7.7.15	Mouse	11404253
choline-phosphate cytidylyltransferase	2.7.7.15	Mouse	11521967
choline-phosphate cytidylyltransferase	2.7.7.15	Mouse	11829742

choline-phosphate cytidylyltransferase	2.7.7.15	Mouse	12034570
choline-phosphate cytidylyltransferase	2.7.7.15	Mouse	12052891
choline-phosphate cytidylyltransferase	2.7.7.15	Mouse	12062780
choline-phosphate cytidylyltransferase	2.7.7.15	Mouse	12221122
choline-phosphate cytidylyltransferase	2.7.7.15	Mouse	12271462
choline-phosphate cytidylyltransferase	2.7.7.15	Mouse	12370080
choline-phosphate cytidylyltransferase	2.7.7.15	Mouse	12620118
choline-phosphate cytidylyltransferase	2.7.7.15	Mouse	12659631
choline-phosphate cytidylyltransferase	2.7.7.15	Mouse	12842190
choline-phosphate cytidylyltransferase	2.7.7.15	Mouse	12928431
choline-phosphate cytidylyltransferase	2.7.7.15	Mouse	14536058
choline-phosphate cytidylyltransferase	2.7.7.15	Mouse	15079868
choline-phosphate cytidylyltransferase	2.7.7.15	Mouse	15139854
choline-phosphate cytidylyltransferase	2.7.7.15	Mouse	15210848
choline-phosphate cytidylyltransferase	2.7.7.15	Mouse	15522825
choline-phosphate cytidylyltransferase	2.7.7.15	Mouse	15574675
choline-phosphate cytidylyltransferase	2.7.7.15	Mouse	15635091
choline-phosphate cytidylyltransferase	2.7.7.15	Mouse	15788406
choline-phosphate cytidylyltransferase	2.7.7.15	Mouse	15982005
choline-phosphate cytidylyltransferase	2.7.7.15	Mouse	16097951
choline-phosphate cytidylyltransferase	2.7.7.15	Mouse	16153613
choline-phosphate cytidylyltransferase	2.7.7.15	Mouse	16236026
choline-phosphate cytidylyltransferase	2.7.7.15	Mouse	16511521
choline-phosphate cytidylyltransferase	2.7.7.15	Mouse	16580250
choline-phosphate cytidylyltransferase	2.7.7.15	Mouse	16580875
choline-phosphate cytidylyltransferase	2.7.7.15	Mouse	2160812
choline-phosphate cytidylyltransferase	2.7.7.15	Mouse	2268410
choline-phosphate cytidylyltransferase	2.7.7.15	Mouse	2665794
choline-phosphate cytidylyltransferase	2.7.7.15	Mouse	2833508
choline-phosphate cytidylyltransferase	2.7.7.15	Mouse	2838058
choline-phosphate cytidylyltransferase	2.7.7.15	Mouse	3004590
choline-phosphate cytidylyltransferase	2.7.7.15	Mouse	3365445
choline-phosphate cytidylyltransferase	2.7.7.15	Mouse	3367156
choline-phosphate cytidylyltransferase	2.7.7.15	Mouse	3447597
choline-phosphate cytidylyltransferase	2.7.7.15	Mouse	603639
choline-phosphate cytidylyltransferase	2.7.7.15	Mouse	6134645
choline-phosphate cytidylyltransferase	2.7.7.15	Mouse	6243289
choline-phosphate cytidylyltransferase	2.7.7.15	Mouse	6477961
choline-phosphate cytidylyltransferase	2.7.7.15	Mouse	7126613
choline-phosphate cytidylyltransferase	2.7.7.15	Mouse	7487944
choline-phosphate cytidylyltransferase	2.7.7.15	Mouse	7588775
choline-phosphate cytidylyltransferase	2.7.7.15	Mouse	7637558
choline-phosphate cytidylyltransferase	2.7.7.15	Mouse	7768909

choline-phosphate cytidylyltransferase	2.7.7.15	Mouse	7782919
choline-phosphate cytidylyltransferase	2.7.7.15	Mouse	7836412
choline-phosphate cytidylyltransferase	2.7.7.15	Mouse	8006517
choline-phosphate cytidylyltransferase	2.7.7.15	Mouse	8182083
choline-phosphate cytidylyltransferase	2.7.7.15	Mouse	8239319
choline-phosphate cytidylyltransferase	2.7.7.15	Mouse	8255685
choline-phosphate cytidylyltransferase	2.7.7.15	Mouse	8387510
choline-phosphate cytidylyltransferase	2.7.7.15	Mouse	8504126
choline-phosphate cytidylyltransferase	2.7.7.15	Mouse	8597584
choline-phosphate cytidylyltransferase	2.7.7.15	Mouse	8626633
choline-phosphate cytidylyltransferase	2.7.7.15	Mouse	8663247
choline-phosphate cytidylyltransferase	2.7.7.15	Mouse	8756587
choline-phosphate cytidylyltransferase	2.7.7.15	Mouse	8761490
choline-phosphate cytidylyltransferase	2.7.7.15	Mouse	8810902
choline-phosphate cytidylyltransferase	2.7.7.15	Mouse	9009253
choline-phosphate cytidylyltransferase	2.7.7.15	Mouse	9046356
choline-phosphate cytidylyltransferase	2.7.7.15	Mouse	9148929
choline-phosphate cytidylyltransferase	2.7.7.15	Mouse	9335949
choline-phosphate cytidylyltransferase	2.7.7.15	Mouse	9370319
choline-phosphate cytidylyltransferase	2.7.7.15	Mouse	9421188
choline-phosphate cytidylyltransferase	2.7.7.15	Mouse	9714757
pantetheine-phosphate adenylyltransferase	2.7.7.3	Mouse	1746161
sulfate adenylyltransferase	2.7.7.4	Mouse	10759525
sulfate adenylyltransferase	2.7.7.4	Mouse	10956658
sulfate adenylyltransferase	2.7.7.4	Mouse	11157739
sulfate adenylyltransferase	2.7.7.4	Mouse	14613928
sulfate adenylyltransferase	2.7.7.4	Mouse	16008502
sulfate adenylyltransferase	2.7.7.4	Mouse	17095009
sulfate adenylyltransferase	2.7.7.4	Mouse	8206850
sulfate adenylyltransferase	2.7.7.4	Mouse	9880353
phosphatidate cytidylyltransferase	2.7.7.41	Mouse	11985865
phosphatidate cytidylyltransferase	2.7.7.41	Mouse	9345289
N-acylneuraminate cytidylyltransferase	2.7.7.43	Mouse	10320348
N-acylneuraminate cytidylyltransferase	2.7.7.43	Mouse	3024643
N-acylneuraminate cytidylyltransferase	2.7.7.43	Mouse	7830552
polyribonucleotide nucleotidyltransferase	2.7.7.8	Mouse	126862
UTP---glucose-1-phosphate uridylyltransferase	2.7.7.9	Mouse	11171080
UTP---glucose-1-phosphate uridylyltransferase	2.7.7.9	Mouse	1149741
UTP---glucose-1-phosphate uridylyltransferase	2.7.7.9	Mouse	12088504

UTP---glucose-1-phosphate uridylyltransferase	2.7.7.9	Mouse	15274139
alcohol sulfotransferase	2.8.2.2	Mouse	7900959
[heparan sulfate]-glucosamine 3-sulfotransferase 1	2.8.2.23	Mouse	12671048
sterol esterase	3.1.1.13	Mouse	10569995
sterol esterase	3.1.1.13	Mouse	15809341
sterol esterase	3.1.1.13	Mouse	8258956
triacylglycerol lipase	3.1.1.3	Mouse	11217140
triacylglycerol lipase	3.1.1.3	Mouse	12689525
triacylglycerol lipase	3.1.1.3	Mouse	3678753
lipoprotein lipase	3.1.1.34	Mouse	10077655
lipoprotein lipase	3.1.1.34	Mouse	10226565
lipoprotein lipase	3.1.1.34	Mouse	10364085
lipoprotein lipase	3.1.1.34	Mouse	10388470
lipoprotein lipase	3.1.1.34	Mouse	10515359
lipoprotein lipase	3.1.1.34	Mouse	10650951
lipoprotein lipase	3.1.1.34	Mouse	10704617
lipoprotein lipase	3.1.1.34	Mouse	10965219
lipoprotein lipase	3.1.1.34	Mouse	11158876
lipoprotein lipase	3.1.1.34	Mouse	11334409
lipoprotein lipase	3.1.1.34	Mouse	11432868
lipoprotein lipase	3.1.1.34	Mouse	11591230
lipoprotein lipase	3.1.1.34	Mouse	12079052
lipoprotein lipase	3.1.1.34	Mouse	12352010
lipoprotein lipase	3.1.1.34	Mouse	12847564
lipoprotein lipase	3.1.1.34	Mouse	12862202
lipoprotein lipase	3.1.1.34	Mouse	12934668
lipoprotein lipase	3.1.1.34	Mouse	1401083
lipoprotein lipase	3.1.1.34	Mouse	14580165
lipoprotein lipase	3.1.1.34	Mouse	14656997
lipoprotein lipase	3.1.1.34	Mouse	14660566
lipoprotein lipase	3.1.1.34	Mouse	15178298
lipoprotein lipase	3.1.1.34	Mouse	15262189
lipoprotein lipase	3.1.1.34	Mouse	15320848
lipoprotein lipase	3.1.1.34	Mouse	15562391
lipoprotein lipase	3.1.1.34	Mouse	15697220
lipoprotein lipase	3.1.1.34	Mouse	15801017
lipoprotein lipase	3.1.1.34	Mouse	16195388
lipoprotein lipase	3.1.1.34	Mouse	16416313
lipoprotein lipase	3.1.1.34	Mouse	16531751
lipoprotein lipase	3.1.1.34	Mouse	16767221
lipoprotein lipase	3.1.1.34	Mouse	1737833

lipoprotein lipase	3.1.1.34	Mouse	1999438
lipoprotein lipase	3.1.1.34	Mouse	2117022
lipoprotein lipase	3.1.1.34	Mouse	2165281
lipoprotein lipase	3.1.1.34	Mouse	2765496
lipoprotein lipase	3.1.1.34	Mouse	3304415
lipoprotein lipase	3.1.1.34	Mouse	3519325
lipoprotein lipase	3.1.1.34	Mouse	3817303
lipoprotein lipase	3.1.1.34	Mouse	3920639
lipoprotein lipase	3.1.1.34	Mouse	3950770
lipoprotein lipase	3.1.1.34	Mouse	4077022
lipoprotein lipase	3.1.1.34	Mouse	6482738
lipoprotein lipase	3.1.1.34	Mouse	7033153
lipoprotein lipase	3.1.1.34	Mouse	7126037
lipoprotein lipase	3.1.1.34	Mouse	7229033
lipoprotein lipase	3.1.1.34	Mouse	7276825
lipoprotein lipase	3.1.1.34	Mouse	7592875
lipoprotein lipase	3.1.1.34	Mouse	7630312
lipoprotein lipase	3.1.1.34	Mouse	7939219
lipoprotein lipase	3.1.1.34	Mouse	7956906
lipoprotein lipase	3.1.1.34	Mouse	8371063
lipoprotein lipase	3.1.1.34	Mouse	8374222
lipoprotein lipase	3.1.1.34	Mouse	8422428
lipoprotein lipase	3.1.1.34	Mouse	8480620
lipoprotein lipase	3.1.1.34	Mouse	8729382
lipoprotein lipase	3.1.1.34	Mouse	8882874
lipoprotein lipase	3.1.1.34	Mouse	8919277
lipoprotein lipase	3.1.1.34	Mouse	9188470
lipoprotein lipase	3.1.1.34	Mouse	9193431
lipoprotein lipase	3.1.1.34	Mouse	9264396
lipoprotein lipase	3.1.1.34	Mouse	9294198
lipoprotein lipase	3.1.1.34	Mouse	9298816
lipoprotein lipase	3.1.1.34	Mouse	9351402
lipoprotein lipase	3.1.1.34	Mouse	9358077
lipoprotein lipase	3.1.1.34	Mouse	9382958
lipoprotein lipase	3.1.1.34	Mouse	9495276
lipoprotein lipase	3.1.1.34	Mouse	9727057
lipoprotein lipase	3.1.1.34	Mouse	9888641
lipoprotein lipase	3.1.1.34	Mouse	9888650
lipoprotein lipase	3.1.1.34	Mouse	9924194
lipoprotein lipase	3.1.1.34	Mouse	9973300
phospholipase A2	3.1.1.4	Mouse	10435206
phospholipase A2	3.1.1.4	Mouse	10482042
phospholipase A2	3.1.1.4	Mouse	10614936

phospholipase A2	3.1.1.4	Mouse	106389
phospholipase A2	3.1.1.4	Mouse	10749741
phospholipase A2	3.1.1.4	Mouse	10793641
phospholipase A2	3.1.1.4	Mouse	10919502
phospholipase A2	3.1.1.4	Mouse	10970711
phospholipase A2	3.1.1.4	Mouse	11080682
phospholipase A2	3.1.1.4	Mouse	11085935
phospholipase A2	3.1.1.4	Mouse	11099485
phospholipase A2	3.1.1.4	Mouse	11115401
phospholipase A2	3.1.1.4	Mouse	11328947
phospholipase A2	3.1.1.4	Mouse	11374398
phospholipase A2	3.1.1.4	Mouse	11964173
phospholipase A2	3.1.1.4	Mouse	12076714
phospholipase A2	3.1.1.4	Mouse	12143044
phospholipase A2	3.1.1.4	Mouse	12189011
phospholipase A2	3.1.1.4	Mouse	124658
phospholipase A2	3.1.1.4	Mouse	12724134
phospholipase A2	3.1.1.4	Mouse	12855693
phospholipase A2	3.1.1.4	Mouse	1322564
phospholipase A2	3.1.1.4	Mouse	1400321
phospholipase A2	3.1.1.4	Mouse	1410519
phospholipase A2	3.1.1.4	Mouse	14561756
phospholipase A2	3.1.1.4	Mouse	15041029
phospholipase A2	3.1.1.4	Mouse	1510970
phospholipase A2	3.1.1.4	Mouse	15211007
phospholipase A2	3.1.1.4	Mouse	15214789
phospholipase A2	3.1.1.4	Mouse	15283760
phospholipase A2	3.1.1.4	Mouse	15377291
phospholipase A2	3.1.1.4	Mouse	15472477
phospholipase A2	3.1.1.4	Mouse	15521009
phospholipase A2	3.1.1.4	Mouse	15743759
phospholipase A2	3.1.1.4	Mouse	15900018
phospholipase A2	3.1.1.4	Mouse	16179540
phospholipase A2	3.1.1.4	Mouse	16221889
phospholipase A2	3.1.1.4	Mouse	16318667
phospholipase A2	3.1.1.4	Mouse	16443193
phospholipase A2	3.1.1.4	Mouse	16716827
phospholipase A2	3.1.1.4	Mouse	16754327
phospholipase A2	3.1.1.4	Mouse	16794537
phospholipase A2	3.1.1.4	Mouse	16968951
phospholipase A2	3.1.1.4	Mouse	17008548
phospholipase A2	3.1.1.4	Mouse	1762050
phospholipase A2	3.1.1.4	Mouse	1836009

phospholipase A2	3.1.1.4	Mouse	1976627
phospholipase A2	3.1.1.4	Mouse	2075195
phospholipase A2	3.1.1.4	Mouse	2217203
phospholipase A2	3.1.1.4	Mouse	2218714
phospholipase A2	3.1.1.4	Mouse	2223919
phospholipase A2	3.1.1.4	Mouse	2250570
phospholipase A2	3.1.1.4	Mouse	2354835
phospholipase A2	3.1.1.4	Mouse	2454329
phospholipase A2	3.1.1.4	Mouse	2646218
phospholipase A2	3.1.1.4	Mouse	2742867
phospholipase A2	3.1.1.4	Mouse	2841909
phospholipase A2	3.1.1.4	Mouse	2848583
phospholipase A2	3.1.1.4	Mouse	3085592
phospholipase A2	3.1.1.4	Mouse	3141409
phospholipase A2	3.1.1.4	Mouse	3164726
phospholipase A2	3.1.1.4	Mouse	3240001
phospholipase A2	3.1.1.4	Mouse	3343241
phospholipase A2	3.1.1.4	Mouse	3745162
phospholipase A2	3.1.1.4	Mouse	386140
phospholipase A2	3.1.1.4	Mouse	6135325
phospholipase A2	3.1.1.4	Mouse	6527548
phospholipase A2	3.1.1.4	Mouse	6955805
phospholipase A2	3.1.1.4	Mouse	7539396
phospholipase A2	3.1.1.4	Mouse	7649158
phospholipase A2	3.1.1.4	Mouse	7681828
phospholipase A2	3.1.1.4	Mouse	7835820
phospholipase A2	3.1.1.4	Mouse	7938094
phospholipase A2	3.1.1.4	Mouse	7945230
phospholipase A2	3.1.1.4	Mouse	7998975
phospholipase A2	3.1.1.4	Mouse	8067978
phospholipase A2	3.1.1.4	Mouse	8148385
phospholipase A2	3.1.1.4	Mouse	8278618
phospholipase A2	3.1.1.4	Mouse	8280164
phospholipase A2	3.1.1.4	Mouse	8292024
phospholipase A2	3.1.1.4	Mouse	8307447
phospholipase A2	3.1.1.4	Mouse	8307472
phospholipase A2	3.1.1.4	Mouse	8347632
phospholipase A2	3.1.1.4	Mouse	8536274
phospholipase A2	3.1.1.4	Mouse	8570769
phospholipase A2	3.1.1.4	Mouse	8648901
phospholipase A2	3.1.1.4	Mouse	8739397
phospholipase A2	3.1.1.4	Mouse	8773214
phospholipase A2	3.1.1.4	Mouse	8808111

phospholipase A2	3.1.1.4	Mouse	8832056
phospholipase A2	3.1.1.4	Mouse	8863185
phospholipase A2	3.1.1.4	Mouse	8865467
phospholipase A2	3.1.1.4	Mouse	8873778
phospholipase A2	3.1.1.4	Mouse	8888134
phospholipase A2	3.1.1.4	Mouse	9172747
phospholipase A2	3.1.1.4	Mouse	9173912
phospholipase A2	3.1.1.4	Mouse	9187306
phospholipase A2	3.1.1.4	Mouse	9219895
phospholipase A2	3.1.1.4	Mouse	9221748
phospholipase A2	3.1.1.4	Mouse	9275050
phospholipase A2	3.1.1.4	Mouse	9314599
phospholipase A2	3.1.1.4	Mouse	9322315
phospholipase A2	3.1.1.4	Mouse	9398170
phospholipase A2	3.1.1.4	Mouse	9410883
phospholipase A2	3.1.1.4	Mouse	9425915
phospholipase A2	3.1.1.4	Mouse	9448723
phospholipase A2	3.1.1.4	Mouse	9463402
phospholipase A2	3.1.1.4	Mouse	9513902
phospholipase A2	3.1.1.4	Mouse	9538252
phospholipase A2	3.1.1.4	Mouse	9562240
phospholipase A2	3.1.1.4	Mouse	9585093
phospholipase A2	3.1.1.4	Mouse	9764845
phospholipase A2	3.1.1.4	Mouse	9879666
acetylcholinesterase	3.1.1.7	Mouse	10766776
acetylcholinesterase	3.1.1.7	Mouse	12415870
acetylcholinesterase	3.1.1.7	Mouse	15936353
acetylcholinesterase	3.1.1.7	Mouse	17562604
acetylcholinesterase	3.1.1.7	Mouse	1820094
acetylcholinesterase	3.1.1.7	Mouse	2587621
acetylcholinesterase	3.1.1.7	Mouse	4795368
acetylcholinesterase	3.1.1.7	Mouse	668
acetylcholinesterase	3.1.1.7	Mouse	9109840
acetylcholinesterase	3.1.1.7	Mouse	9548556
alkaline phosphatase	3.1.3.1	Mouse	11029583
alkaline phosphatase	3.1.3.1	Mouse	11139445
alkaline phosphatase	3.1.3.1	Mouse	12412807
alkaline phosphatase	3.1.3.1	Mouse	1445337
alkaline phosphatase	3.1.3.1	Mouse	14560000
alkaline phosphatase	3.1.3.1	Mouse	3509742
alkaline phosphatase	3.1.3.1	Mouse	7032602
fructose-bisphosphatase	3.1.3.11	Mouse	10327613
fructose-bisphosphatase	3.1.3.11	Mouse	10393302

fructose-bisphosphatase	3.1.3.11	Mouse	10773464
fructose-bisphosphatase	3.1.3.11	Mouse	11536627
fructose-bisphosphatase	3.1.3.11	Mouse	11864619
fructose-bisphosphatase	3.1.3.11	Mouse	12190028
fructose-bisphosphatase	3.1.3.11	Mouse	12686616
fructose-bisphosphatase	3.1.3.11	Mouse	15225753
fructose-bisphosphatase	3.1.3.11	Mouse	15498578
fructose-bisphosphatase	3.1.3.11	Mouse	15631980
fructose-bisphosphatase	3.1.3.11	Mouse	16199065
fructose-bisphosphatase	3.1.3.11	Mouse	16580859
fructose-bisphosphatase	3.1.3.11	Mouse	16593209
fructose-bisphosphatase	3.1.3.11	Mouse	16657971
fructose-bisphosphatase	3.1.3.11	Mouse	16814784
fructose-bisphosphatase	3.1.3.11	Mouse	16857246
fructose-bisphosphatase	3.1.3.11	Mouse	2835013
fructose-bisphosphatase	3.1.3.11	Mouse	2983680
fructose-bisphosphatase	3.1.3.11	Mouse	3068502
fructose-bisphosphatase	3.1.3.11	Mouse	3291467
fructose-bisphosphatase	3.1.3.11	Mouse	4342496
fructose-bisphosphatase	3.1.3.11	Mouse	7532742
fructose-bisphosphatase	3.1.3.11	Mouse	7552262
fructose-bisphosphatase	3.1.3.11	Mouse	7558035
fructose-bisphosphatase	3.1.3.11	Mouse	7579072
fructose-bisphosphatase	3.1.3.11	Mouse	7592860
fructose-bisphosphatase	3.1.3.11	Mouse	8043646
acid phosphatase	3.1.3.2	Mouse	10471332
acid phosphatase	3.1.3.2	Mouse	10646119
acid phosphatase	3.1.3.2	Mouse	1364364
acid phosphatase	3.1.3.2	Mouse	2153810
acid phosphatase	3.1.3.2	Mouse	2545859
acid phosphatase	3.1.3.2	Mouse	2713876
acid phosphatase	3.1.3.2	Mouse	9370316
inositol-phosphate phosphatase	3.1.3.25	Mouse	12479670
inositol-phosphate phosphatase	3.1.3.25	Mouse	15809430
inositol-phosphate phosphatase	3.1.3.25	Mouse	9462881
phosphoserine phosphatase	3.1.3.3	Mouse	8858931
phosphatidate phosphatase	3.1.3.4	Mouse	10695929
phosphatidate phosphatase	3.1.3.4	Mouse	11678440
phosphatidate phosphatase	3.1.3.4	Mouse	12359092
phosphatidate phosphatase	3.1.3.4	Mouse	15527069
phosphatidate phosphatase	3.1.3.4	Mouse	15539300
phosphatidate phosphatase	3.1.3.4	Mouse	15975496
phosphatidate phosphatase	3.1.3.4	Mouse	16684527

phosphatidate phosphatase	3.1.3.4	Mouse	16968695
phosphatidate phosphatase	3.1.3.4	Mouse	2153810
phosphatidate phosphatase	3.1.3.4	Mouse	2174075
phosphatidate phosphatase	3.1.3.4	Mouse	2674663
phosphatidate phosphatase	3.1.3.4	Mouse	2713876
phosphatidate phosphatase	3.1.3.4	Mouse	6277612
phosphatidate phosphatase	3.1.3.4	Mouse	716971
phosphatidate phosphatase	3.1.3.4	Mouse	7273126
phosphatidate phosphatase	3.1.3.4	Mouse	7305894
phosphatidate phosphatase	3.1.3.4	Mouse	7407220
phosphatidate phosphatase	3.1.3.4	Mouse	7748273
phosphatidate phosphatase	3.1.3.4	Mouse	8406356
phosphatidate phosphatase	3.1.3.4	Mouse	8784738
5'-nucleotidase	3.1.3.5	Mouse	10506947
5'-nucleotidase	3.1.3.5	Mouse	10617137
5'-nucleotidase	3.1.3.5	Mouse	10766785
5'-nucleotidase	3.1.3.5	Mouse	10869532
5'-nucleotidase	3.1.3.5	Mouse	11408527
5'-nucleotidase	3.1.3.5	Mouse	12030367
5'-nucleotidase	3.1.3.5	Mouse	12061138
5'-nucleotidase	3.1.3.5	Mouse	12204768
5'-nucleotidase	3.1.3.5	Mouse	12493585
5'-nucleotidase	3.1.3.5	Mouse	12571440
5'-nucleotidase	3.1.3.5	Mouse	12667292
5'-nucleotidase	3.1.3.5	Mouse	15748706
5'-nucleotidase	3.1.3.5	Mouse	15946667
5'-nucleotidase	3.1.3.5	Mouse	6326848
5'-nucleotidase	3.1.3.5	Mouse	7999131
5'-nucleotidase	3.1.3.5	Mouse	9009712
5'-nucleotidase	3.1.3.5	Mouse	9315889
5'-nucleotidase	3.1.3.5	Mouse	9806332
glucose-6-phosphatase	3.1.3.9	Mouse	10625614
glucose-6-phosphatase	3.1.3.9	Mouse	12189168
glucose-6-phosphatase	3.1.3.9	Mouse	12373573
glucose-6-phosphatase	3.1.3.9	Mouse	12507516
glucose-6-phosphatase	3.1.3.9	Mouse	1323600
glucose-6-phosphatase	3.1.3.9	Mouse	15702236
glucose-6-phosphatase	3.1.3.9	Mouse	16012821
glucose-6-phosphatase	3.1.3.9	Mouse	16330542
glucose-6-phosphatase	3.1.3.9	Mouse	17075770
glucose-6-phosphatase	3.1.3.9	Mouse	3038860
glucose-6-phosphatase	3.1.3.9	Mouse	3629725
glucose-6-phosphatase	3.1.3.9	Mouse	6295653

glucose-6-phosphatase	3.1.3.9	Mouse	6317150
glucose-6-phosphatase	3.1.3.9	Mouse	6591771
glucose-6-phosphatase	3.1.3.9	Mouse	8182131
glucose-6-phosphatase	3.1.3.9	Mouse	8211187
glucose-6-phosphatase	3.1.3.9	Mouse	8407995
glucose-6-phosphatase	3.1.3.9	Mouse	8640227
glucose-6-phosphatase	3.1.3.9	Mouse	9369482
glucose-6-phosphatase	3.1.3.9	Mouse	9497333
glucose-6-phosphatase	3.1.3.9	Mouse	9506766
glucose-6-phosphatase	3.1.3.9	Mouse	9839806
cerebroside-sulfatase	3.1.6.8	Mouse	6233132
alpha-glucosidase	3.2.1.20	Mouse	10619707
alpha-glucosidase	3.2.1.20	Mouse	10810293
alpha-glucosidase	3.2.1.20	Mouse	11134937
alpha-glucosidase	3.2.1.20	Mouse	11230125
alpha-glucosidase	3.2.1.20	Mouse	11404235
alpha-glucosidase	3.2.1.20	Mouse	16233203
alpha-glucosidase	3.2.1.20	Mouse	16233375
alpha-glucosidase	3.2.1.20	Mouse	7626594
alpha-glucosidase	3.2.1.20	Mouse	8625892
alpha-mannosidase	3.2.1.24	Mouse	11406577
alpha-mannosidase	3.2.1.24	Mouse	16460512
alpha-mannosidase	3.2.1.24	Mouse	16806128
alpha-mannosidase	3.2.1.24	Mouse	16899540
alpha-mannosidase	3.2.1.24	Mouse	2466460
alpha-mannosidase	3.2.1.24	Mouse	2843530
alpha-mannosidase	3.2.1.24	Mouse	4092864
alpha-mannosidase	3.2.1.24	Mouse	9022667
alpha-mannosidase	3.2.1.24	Mouse	9230311
leukotriene-A4 hydrolase	3.3.2.6	Mouse	10691697
leukotriene-A4 hydrolase	3.3.2.6	Mouse	11805219
leukotriene-A4 hydrolase	3.3.2.6	Mouse	12139459
leukotriene-A4 hydrolase	3.3.2.6	Mouse	12865451
leukotriene-A4 hydrolase	3.3.2.6	Mouse	1311589
leukotriene-A4 hydrolase	3.3.2.6	Mouse	6329309
leukotriene-A4 hydrolase	3.3.2.6	Mouse	7732829
leukotriene-A4 hydrolase	3.3.2.6	Mouse	9395533
leukotriene-A4 hydrolase	3.3.2.6	Mouse	9413890
membrane alanyl aminopeptidase	3.4.11.2	Mouse	11751433
membrane alanyl aminopeptidase	3.4.11.2	Mouse	12075625
membrane alanyl aminopeptidase	3.4.11.2	Mouse	16019130
dihydroorotase	3.5.2.3	Mouse	3345746
dihydroorotase	3.5.2.3	Mouse	4092695

dihydroorotase	3.5.2.3	Mouse	42399
dihydroorotase	3.5.2.3	Mouse	6115855
GTP cyclohydrolase I	3.5.4.16	Mouse	12392559
GTP cyclohydrolase I	3.5.4.16	Mouse	12855421
GTP cyclohydrolase I	3.5.4.16	Mouse	1459137
GTP cyclohydrolase I	3.5.4.16	Mouse	15044686
GTP cyclohydrolase I	3.5.4.16	Mouse	16179591
GTP cyclohydrolase I	3.5.4.16	Mouse	16636057
GTP cyclohydrolase I	3.5.4.16	Mouse	737222
GTP cyclohydrolase I	3.5.4.16	Mouse	7521513
GTP cyclohydrolase I	3.5.4.16	Mouse	9182249
GTP cyclohydrolase I	3.5.4.16	Mouse	9444617
adenosinetriphosphatase	3.6.1.3	Mouse	126449
adenosinetriphosphatase	3.6.1.3	Mouse	160792
adenosinetriphosphatase	3.6.1.3	Mouse	1727783
adenosinetriphosphatase	3.6.1.3	Mouse	3772811
adenosinetriphosphatase	3.6.1.3	Mouse	8667177
H+-transporting two-sector ATPase	3.6.3.14	Mouse	11744700
H+-transporting two-sector ATPase	3.6.3.14	Mouse	11893513
H+-transporting two-sector ATPase	3.6.3.14	Mouse	12587531
H+-transporting two-sector ATPase	3.6.3.14	Mouse	15712234
H+-transporting two-sector ATPase	3.6.3.14	Mouse	16510118
H+-transporting two-sector ATPase	3.6.3.14	Mouse	16730639
H+-transporting two-sector ATPase	3.6.3.14	Mouse	2532597
H+-transporting two-sector ATPase	3.6.3.14	Mouse	2889730
H+-transporting two-sector ATPase	3.6.3.14	Mouse	6456904
H+-transporting two-sector ATPase	3.6.3.14	Mouse	8516333
fumarylacetoacetase	3.7.1.2	Mouse	9734339
glutamate decarboxylase	4.1.1.15	Mouse	10331265
glutamate decarboxylase	4.1.1.15	Mouse	12196588
glutamate decarboxylase	4.1.1.15	Mouse	12746320
glutamate decarboxylase	4.1.1.15	Mouse	15210535
glutamate decarboxylase	4.1.1.15	Mouse	15581395
glutamate decarboxylase	4.1.1.15	Mouse	1697032
glutamate decarboxylase	4.1.1.15	Mouse	17044036
glutamate decarboxylase	4.1.1.15	Mouse	1976015
glutamate decarboxylase	4.1.1.15	Mouse	2180326
glutamate decarboxylase	4.1.1.15	Mouse	2735448
glutamate decarboxylase	4.1.1.15	Mouse	2857768
glutamate decarboxylase	4.1.1.15	Mouse	3896834
glutamate decarboxylase	4.1.1.15	Mouse	6975381
glutamate decarboxylase	4.1.1.15	Mouse	7702443
glutamate decarboxylase	4.1.1.15	Mouse	7885536

glutamate decarboxylase	4.1.1.15	Mouse	8302162
glutamate decarboxylase	4.1.1.15	Mouse	9011754
glutamate decarboxylase	4.1.1.15	Mouse	9053794
ornithine decarboxylase	4.1.1.17	Mouse	10069996
ornithine decarboxylase	4.1.1.17	Mouse	10216947
ornithine decarboxylase	4.1.1.17	Mouse	10319188
ornithine decarboxylase	4.1.1.17	Mouse	10320037
ornithine decarboxylase	4.1.1.17	Mouse	10321508
ornithine decarboxylase	4.1.1.17	Mouse	10430664
ornithine decarboxylase	4.1.1.17	Mouse	10453061
ornithine decarboxylase	4.1.1.17	Mouse	10456943
ornithine decarboxylase	4.1.1.17	Mouse	10473083
ornithine decarboxylase	4.1.1.17	Mouse	10485326
ornithine decarboxylase	4.1.1.17	Mouse	10544213
ornithine decarboxylase	4.1.1.17	Mouse	10550568
ornithine decarboxylase	4.1.1.17	Mouse	10564512
ornithine decarboxylase	4.1.1.17	Mouse	10589756
ornithine decarboxylase	4.1.1.17	Mouse	10593613
ornithine decarboxylase	4.1.1.17	Mouse	10607762
ornithine decarboxylase	4.1.1.17	Mouse	10629084
ornithine decarboxylase	4.1.1.17	Mouse	10712236
ornithine decarboxylase	4.1.1.17	Mouse	10713131
ornithine decarboxylase	4.1.1.17	Mouse	10760944
ornithine decarboxylase	4.1.1.17	Mouse	10772389
ornithine decarboxylase	4.1.1.17	Mouse	10816435
ornithine decarboxylase	4.1.1.17	Mouse	10817834
ornithine decarboxylase	4.1.1.17	Mouse	10882097
ornithine decarboxylase	4.1.1.17	Mouse	10931831
ornithine decarboxylase	4.1.1.17	Mouse	10940513
ornithine decarboxylase	4.1.1.17	Mouse	10965017
ornithine decarboxylase	4.1.1.17	Mouse	11003584
ornithine decarboxylase	4.1.1.17	Mouse	11085920
ornithine decarboxylase	4.1.1.17	Mouse	11095648
ornithine decarboxylase	4.1.1.17	Mouse	11137705
ornithine decarboxylase	4.1.1.17	Mouse	11180396
ornithine decarboxylase	4.1.1.17	Mouse	11235918
ornithine decarboxylase	4.1.1.17	Mouse	11355005
ornithine decarboxylase	4.1.1.17	Mouse	11376395
ornithine decarboxylase	4.1.1.17	Mouse	11408092
ornithine decarboxylase	4.1.1.17	Mouse	11408253
ornithine decarboxylase	4.1.1.17	Mouse	11408542
ornithine decarboxylase	4.1.1.17	Mouse	11413269
ornithine decarboxylase	4.1.1.17	Mouse	11540835

ornithine decarboxylase	4.1.1.17	Mouse	11558274
ornithine decarboxylase	4.1.1.17	Mouse	11736657
ornithine decarboxylase	4.1.1.17	Mouse	11782361
ornithine decarboxylase	4.1.1.17	Mouse	11852055
ornithine decarboxylase	4.1.1.17	Mouse	11883715
ornithine decarboxylase	4.1.1.17	Mouse	11922393
ornithine decarboxylase	4.1.1.17	Mouse	11923270
ornithine decarboxylase	4.1.1.17	Mouse	11964084
ornithine decarboxylase	4.1.1.17	Mouse	11997243
ornithine decarboxylase	4.1.1.17	Mouse	12054570
ornithine decarboxylase	4.1.1.17	Mouse	12105848
ornithine decarboxylase	4.1.1.17	Mouse	12148577
ornithine decarboxylase	4.1.1.17	Mouse	12355213
ornithine decarboxylase	4.1.1.17	Mouse	12452334
ornithine decarboxylase	4.1.1.17	Mouse	12497077
ornithine decarboxylase	4.1.1.17	Mouse	12527115
ornithine decarboxylase	4.1.1.17	Mouse	12663506
ornithine decarboxylase	4.1.1.17	Mouse	12716308
ornithine decarboxylase	4.1.1.17	Mouse	12716758
ornithine decarboxylase	4.1.1.17	Mouse	12766050
ornithine decarboxylase	4.1.1.17	Mouse	12816757
ornithine decarboxylase	4.1.1.17	Mouse	12856719
ornithine decarboxylase	4.1.1.17	Mouse	12882169
ornithine decarboxylase	4.1.1.17	Mouse	1289667
ornithine decarboxylase	4.1.1.17	Mouse	1324153
ornithine decarboxylase	4.1.1.17	Mouse	1360468
ornithine decarboxylase	4.1.1.17	Mouse	1397089
ornithine decarboxylase	4.1.1.17	Mouse	1407701
ornithine decarboxylase	4.1.1.17	Mouse	1409247
ornithine decarboxylase	4.1.1.17	Mouse	1417733
ornithine decarboxylase	4.1.1.17	Mouse	14769544
ornithine decarboxylase	4.1.1.17	Mouse	1495349
ornithine decarboxylase	4.1.1.17	Mouse	15002659
ornithine decarboxylase	4.1.1.17	Mouse	1511780
ornithine decarboxylase	4.1.1.17	Mouse	15120115
ornithine decarboxylase	4.1.1.17	Mouse	15180186
ornithine decarboxylase	4.1.1.17	Mouse	1521915
ornithine decarboxylase	4.1.1.17	Mouse	15223770
ornithine decarboxylase	4.1.1.17	Mouse	15228220
ornithine decarboxylase	4.1.1.17	Mouse	15233741
ornithine decarboxylase	4.1.1.17	Mouse	15247138
ornithine decarboxylase	4.1.1.17	Mouse	15296840
ornithine decarboxylase	4.1.1.17	Mouse	15306645

ornithine decarboxylase	4.1.1.17	Mouse	15355308
ornithine decarboxylase	4.1.1.17	Mouse	15514084
ornithine decarboxylase	4.1.1.17	Mouse	15538383
ornithine decarboxylase	4.1.1.17	Mouse	15539331
ornithine decarboxylase	4.1.1.17	Mouse	1563337
ornithine decarboxylase	4.1.1.17	Mouse	15697240
ornithine decarboxylase	4.1.1.17	Mouse	1569947
ornithine decarboxylase	4.1.1.17	Mouse	15716048
ornithine decarboxylase	4.1.1.17	Mouse	15843384
ornithine decarboxylase	4.1.1.17	Mouse	1584960
ornithine decarboxylase	4.1.1.17	Mouse	1590311
ornithine decarboxylase	4.1.1.17	Mouse	1601800
ornithine decarboxylase	4.1.1.17	Mouse	16091008
ornithine decarboxylase	4.1.1.17	Mouse	16168128
ornithine decarboxylase	4.1.1.17	Mouse	16170669
ornithine decarboxylase	4.1.1.17	Mouse	16181115
ornithine decarboxylase	4.1.1.17	Mouse	16223706
ornithine decarboxylase	4.1.1.17	Mouse	16230862
ornithine decarboxylase	4.1.1.17	Mouse	16290266
ornithine decarboxylase	4.1.1.17	Mouse	16342411
ornithine decarboxylase	4.1.1.17	Mouse	1641775
ornithine decarboxylase	4.1.1.17	Mouse	16445292
ornithine decarboxylase	4.1.1.17	Mouse	1655898
ornithine decarboxylase	4.1.1.17	Mouse	16568078
ornithine decarboxylase	4.1.1.17	Mouse	16630547
ornithine decarboxylase	4.1.1.17	Mouse	16662219
ornithine decarboxylase	4.1.1.17	Mouse	16666570
ornithine decarboxylase	4.1.1.17	Mouse	16678846
ornithine decarboxylase	4.1.1.17	Mouse	16729674
ornithine decarboxylase	4.1.1.17	Mouse	1697882
ornithine decarboxylase	4.1.1.17	Mouse	1703390
ornithine decarboxylase	4.1.1.17	Mouse	1733364
ornithine decarboxylase	4.1.1.17	Mouse	17407445
ornithine decarboxylase	4.1.1.17	Mouse	1745018
ornithine decarboxylase	4.1.1.17	Mouse	1782416
ornithine decarboxylase	4.1.1.17	Mouse	1814556
ornithine decarboxylase	4.1.1.17	Mouse	1814755
ornithine decarboxylase	4.1.1.17	Mouse	1831810
ornithine decarboxylase	4.1.1.17	Mouse	1846091
ornithine decarboxylase	4.1.1.17	Mouse	1878921
ornithine decarboxylase	4.1.1.17	Mouse	1884248
ornithine decarboxylase	4.1.1.17	Mouse	1892753
ornithine decarboxylase	4.1.1.17	Mouse	1900385

ornithine decarboxylase	4.1.1.17	Mouse	1932775
ornithine decarboxylase	4.1.1.17	Mouse	1940203
ornithine decarboxylase	4.1.1.17	Mouse	1962522
ornithine decarboxylase	4.1.1.17	Mouse	196870
ornithine decarboxylase	4.1.1.17	Mouse	1997184
ornithine decarboxylase	4.1.1.17	Mouse	2006469
ornithine decarboxylase	4.1.1.17	Mouse	2009332
ornithine decarboxylase	4.1.1.17	Mouse	2019760
ornithine decarboxylase	4.1.1.17	Mouse	203259
ornithine decarboxylase	4.1.1.17	Mouse	2051775
ornithine decarboxylase	4.1.1.17	Mouse	2088816
ornithine decarboxylase	4.1.1.17	Mouse	2118148
ornithine decarboxylase	4.1.1.17	Mouse	2148056
ornithine decarboxylase	4.1.1.17	Mouse	2160044
ornithine decarboxylase	4.1.1.17	Mouse	2197525
ornithine decarboxylase	4.1.1.17	Mouse	2210666
ornithine decarboxylase	4.1.1.17	Mouse	2243540
ornithine decarboxylase	4.1.1.17	Mouse	2293084
ornithine decarboxylase	4.1.1.17	Mouse	2296762
ornithine decarboxylase	4.1.1.17	Mouse	2298913
ornithine decarboxylase	4.1.1.17	Mouse	2409817
ornithine decarboxylase	4.1.1.17	Mouse	2469492
ornithine decarboxylase	4.1.1.17	Mouse	2472814
ornithine decarboxylase	4.1.1.17	Mouse	2493794
ornithine decarboxylase	4.1.1.17	Mouse	2494779
ornithine decarboxylase	4.1.1.17	Mouse	2497460
ornithine decarboxylase	4.1.1.17	Mouse	2497556
ornithine decarboxylase	4.1.1.17	Mouse	2505399
ornithine decarboxylase	4.1.1.17	Mouse	2505959
ornithine decarboxylase	4.1.1.17	Mouse	2507383
ornithine decarboxylase	4.1.1.17	Mouse	2507471
ornithine decarboxylase	4.1.1.17	Mouse	2525760
ornithine decarboxylase	4.1.1.17	Mouse	2553150
ornithine decarboxylase	4.1.1.17	Mouse	2610929
ornithine decarboxylase	4.1.1.17	Mouse	2651129
ornithine decarboxylase	4.1.1.17	Mouse	2699646
ornithine decarboxylase	4.1.1.17	Mouse	2754510
ornithine decarboxylase	4.1.1.17	Mouse	2775206
ornithine decarboxylase	4.1.1.17	Mouse	2829727
ornithine decarboxylase	4.1.1.17	Mouse	2840461
ornithine decarboxylase	4.1.1.17	Mouse	2915649
ornithine decarboxylase	4.1.1.17	Mouse	2916900
ornithine decarboxylase	4.1.1.17	Mouse	2977154

ornithine decarboxylase	4.1.1.17	Mouse	2979197
ornithine decarboxylase	4.1.1.17	Mouse	2986953
ornithine decarboxylase	4.1.1.17	Mouse	3004707
ornithine decarboxylase	4.1.1.17	Mouse	3009424
ornithine decarboxylase	4.1.1.17	Mouse	3022056
ornithine decarboxylase	4.1.1.17	Mouse	3036091
ornithine decarboxylase	4.1.1.17	Mouse	3037250
ornithine decarboxylase	4.1.1.17	Mouse	3040821
ornithine decarboxylase	4.1.1.17	Mouse	3082276
ornithine decarboxylase	4.1.1.17	Mouse	3084209
ornithine decarboxylase	4.1.1.17	Mouse	3084872
ornithine decarboxylase	4.1.1.17	Mouse	3086160
ornithine decarboxylase	4.1.1.17	Mouse	3092827
ornithine decarboxylase	4.1.1.17	Mouse	3093095
ornithine decarboxylase	4.1.1.17	Mouse	3096557
ornithine decarboxylase	4.1.1.17	Mouse	3100897
ornithine decarboxylase	4.1.1.17	Mouse	3102397
ornithine decarboxylase	4.1.1.17	Mouse	3105968
ornithine decarboxylase	4.1.1.17	Mouse	3106075
ornithine decarboxylase	4.1.1.17	Mouse	3108666
ornithine decarboxylase	4.1.1.17	Mouse	3109979
ornithine decarboxylase	4.1.1.17	Mouse	3109985
ornithine decarboxylase	4.1.1.17	Mouse	3111384
ornithine decarboxylase	4.1.1.17	Mouse	3113732
ornithine decarboxylase	4.1.1.17	Mouse	3117720
ornithine decarboxylase	4.1.1.17	Mouse	3121457
ornithine decarboxylase	4.1.1.17	Mouse	3122042
ornithine decarboxylase	4.1.1.17	Mouse	3129184
ornithine decarboxylase	4.1.1.17	Mouse	3130188
ornithine decarboxylase	4.1.1.17	Mouse	3139441
ornithine decarboxylase	4.1.1.17	Mouse	3141045
ornithine decarboxylase	4.1.1.17	Mouse	3143046
ornithine decarboxylase	4.1.1.17	Mouse	3180091
ornithine decarboxylase	4.1.1.17	Mouse	3279036
ornithine decarboxylase	4.1.1.17	Mouse	3328430
ornithine decarboxylase	4.1.1.17	Mouse	3356404
ornithine decarboxylase	4.1.1.17	Mouse	3403538
ornithine decarboxylase	4.1.1.17	Mouse	3443298
ornithine decarboxylase	4.1.1.17	Mouse	3538740
ornithine decarboxylase	4.1.1.17	Mouse	3548994
ornithine decarboxylase	4.1.1.17	Mouse	3661847
ornithine decarboxylase	4.1.1.17	Mouse	3672608
ornithine decarboxylase	4.1.1.17	Mouse	3688216

ornithine decarboxylase	4.1.1.17	Mouse	3729588
ornithine decarboxylase	4.1.1.17	Mouse	3743773
ornithine decarboxylase	4.1.1.17	Mouse	3753036
ornithine decarboxylase	4.1.1.17	Mouse	3775249
ornithine decarboxylase	4.1.1.17	Mouse	3794781
ornithine decarboxylase	4.1.1.17	Mouse	3857388
ornithine decarboxylase	4.1.1.17	Mouse	3901680
ornithine decarboxylase	4.1.1.17	Mouse	3905315
ornithine decarboxylase	4.1.1.17	Mouse	3921243
ornithine decarboxylase	4.1.1.17	Mouse	3926303
ornithine decarboxylase	4.1.1.17	Mouse	3930649
ornithine decarboxylase	4.1.1.17	Mouse	3931300
ornithine decarboxylase	4.1.1.17	Mouse	3934106
ornithine decarboxylase	4.1.1.17	Mouse	3999751
ornithine decarboxylase	4.1.1.17	Mouse	4029343
ornithine decarboxylase	4.1.1.17	Mouse	4053280
ornithine decarboxylase	4.1.1.17	Mouse	497279
ornithine decarboxylase	4.1.1.17	Mouse	6124275
ornithine decarboxylase	4.1.1.17	Mouse	6178351
ornithine decarboxylase	4.1.1.17	Mouse	6190690
ornithine decarboxylase	4.1.1.17	Mouse	6192925
ornithine decarboxylase	4.1.1.17	Mouse	6223191
ornithine decarboxylase	4.1.1.17	Mouse	6256169
ornithine decarboxylase	4.1.1.17	Mouse	6272311
ornithine decarboxylase	4.1.1.17	Mouse	6307502
ornithine decarboxylase	4.1.1.17	Mouse	6365078
ornithine decarboxylase	4.1.1.17	Mouse	6432312
ornithine decarboxylase	4.1.1.17	Mouse	6432848
ornithine decarboxylase	4.1.1.17	Mouse	6439208
ornithine decarboxylase	4.1.1.17	Mouse	6440787
ornithine decarboxylase	4.1.1.17	Mouse	6445842
ornithine decarboxylase	4.1.1.17	Mouse	6467454
ornithine decarboxylase	4.1.1.17	Mouse	6571411
ornithine decarboxylase	4.1.1.17	Mouse	6591862
ornithine decarboxylase	4.1.1.17	Mouse	6624798
ornithine decarboxylase	4.1.1.17	Mouse	6692409
ornithine decarboxylase	4.1.1.17	Mouse	6696980
ornithine decarboxylase	4.1.1.17	Mouse	6721578
ornithine decarboxylase	4.1.1.17	Mouse	6750139
ornithine decarboxylase	4.1.1.17	Mouse	6812570
ornithine decarboxylase	4.1.1.17	Mouse	6813460
ornithine decarboxylase	4.1.1.17	Mouse	6865777
ornithine decarboxylase	4.1.1.17	Mouse	6891933

ornithine decarboxylase	4.1.1.17	Mouse	7093948
ornithine decarboxylase	4.1.1.17	Mouse	7104206
ornithine decarboxylase	4.1.1.17	Mouse	7159401
ornithine decarboxylase	4.1.1.17	Mouse	7205597
ornithine decarboxylase	4.1.1.17	Mouse	7310281
ornithine decarboxylase	4.1.1.17	Mouse	7381752
ornithine decarboxylase	4.1.1.17	Mouse	7426404
ornithine decarboxylase	4.1.1.17	Mouse	7488168
ornithine decarboxylase	4.1.1.17	Mouse	7498733
ornithine decarboxylase	4.1.1.17	Mouse	7525612
ornithine decarboxylase	4.1.1.17	Mouse	7616440
ornithine decarboxylase	4.1.1.17	Mouse	7628376
ornithine decarboxylase	4.1.1.17	Mouse	7656288
ornithine decarboxylase	4.1.1.17	Mouse	7718766
ornithine decarboxylase	4.1.1.17	Mouse	7813017
ornithine decarboxylase	4.1.1.17	Mouse	7823874
ornithine decarboxylase	4.1.1.17	Mouse	7865470
ornithine decarboxylase	4.1.1.17	Mouse	7872745
ornithine decarboxylase	4.1.1.17	Mouse	7874572
ornithine decarboxylase	4.1.1.17	Mouse	7895420
ornithine decarboxylase	4.1.1.17	Mouse	7929646
ornithine decarboxylase	4.1.1.17	Mouse	7943199
ornithine decarboxylase	4.1.1.17	Mouse	7951165
ornithine decarboxylase	4.1.1.17	Mouse	7965748
ornithine decarboxylase	4.1.1.17	Mouse	7972938
ornithine decarboxylase	4.1.1.17	Mouse	7981636
ornithine decarboxylase	4.1.1.17	Mouse	8010156
ornithine decarboxylase	4.1.1.17	Mouse	8028020
ornithine decarboxylase	4.1.1.17	Mouse	8065308
ornithine decarboxylase	4.1.1.17	Mouse	8095973
ornithine decarboxylase	4.1.1.17	Mouse	8110472
ornithine decarboxylase	4.1.1.17	Mouse	8140036
ornithine decarboxylase	4.1.1.17	Mouse	8141779
ornithine decarboxylase	4.1.1.17	Mouse	8152342
ornithine decarboxylase	4.1.1.17	Mouse	8185631
ornithine decarboxylase	4.1.1.17	Mouse	8190721
ornithine decarboxylase	4.1.1.17	Mouse	8242794
ornithine decarboxylase	4.1.1.17	Mouse	8344985
ornithine decarboxylase	4.1.1.17	Mouse	8368314
ornithine decarboxylase	4.1.1.17	Mouse	8374143
ornithine decarboxylase	4.1.1.17	Mouse	8419528
ornithine decarboxylase	4.1.1.17	Mouse	8447420
ornithine decarboxylase	4.1.1.17	Mouse	8453677

ornithine decarboxylase	4.1.1.17	Mouse	8462726
ornithine decarboxylase	4.1.1.17	Mouse	8465553
ornithine decarboxylase	4.1.1.17	Mouse	8478959
ornithine decarboxylase	4.1.1.17	Mouse	8501729
ornithine decarboxylase	4.1.1.17	Mouse	8538189
ornithine decarboxylase	4.1.1.17	Mouse	8549635
ornithine decarboxylase	4.1.1.17	Mouse	8572176
ornithine decarboxylase	4.1.1.17	Mouse	8660289
ornithine decarboxylase	4.1.1.17	Mouse	8693031
ornithine decarboxylase	4.1.1.17	Mouse	8707896
ornithine decarboxylase	4.1.1.17	Mouse	8727257
ornithine decarboxylase	4.1.1.17	Mouse	8760120
ornithine decarboxylase	4.1.1.17	Mouse	8760129
ornithine decarboxylase	4.1.1.17	Mouse	8768305
ornithine decarboxylase	4.1.1.17	Mouse	8777294
ornithine decarboxylase	4.1.1.17	Mouse	8814137
ornithine decarboxylase	4.1.1.17	Mouse	8848835
ornithine decarboxylase	4.1.1.17	Mouse	8858522
ornithine decarboxylase	4.1.1.17	Mouse	8878500
ornithine decarboxylase	4.1.1.17	Mouse	8882155
ornithine decarboxylase	4.1.1.17	Mouse	8912847
ornithine decarboxylase	4.1.1.17	Mouse	8944705
ornithine decarboxylase	4.1.1.17	Mouse	9009157
ornithine decarboxylase	4.1.1.17	Mouse	9016399
ornithine decarboxylase	4.1.1.17	Mouse	9017896
ornithine decarboxylase	4.1.1.17	Mouse	9022291
ornithine decarboxylase	4.1.1.17	Mouse	9024941
ornithine decarboxylase	4.1.1.17	Mouse	9063811
ornithine decarboxylase	4.1.1.17	Mouse	9073141
ornithine decarboxylase	4.1.1.17	Mouse	9134011
ornithine decarboxylase	4.1.1.17	Mouse	9142900
ornithine decarboxylase	4.1.1.17	Mouse	9191978
ornithine decarboxylase	4.1.1.17	Mouse	9213218
ornithine decarboxylase	4.1.1.17	Mouse	9223345
ornithine decarboxylase	4.1.1.17	Mouse	9224728
ornithine decarboxylase	4.1.1.17	Mouse	9251105
ornithine decarboxylase	4.1.1.17	Mouse	9252524
ornithine decarboxylase	4.1.1.17	Mouse	9294258
ornithine decarboxylase	4.1.1.17	Mouse	9368191
ornithine decarboxylase	4.1.1.17	Mouse	9371082
ornithine decarboxylase	4.1.1.17	Mouse	9454972
ornithine decarboxylase	4.1.1.17	Mouse	9458728
ornithine decarboxylase	4.1.1.17	Mouse	9468098

ornithine decarboxylase	4.1.1.17	Mouse	9495243
ornithine decarboxylase	4.1.1.17	Mouse	9525811
ornithine decarboxylase	4.1.1.17	Mouse	9590135
ornithine decarboxylase	4.1.1.17	Mouse	9609384
ornithine decarboxylase	4.1.1.17	Mouse	9612269
ornithine decarboxylase	4.1.1.17	Mouse	9624108
ornithine decarboxylase	4.1.1.17	Mouse	9648877
ornithine decarboxylase	4.1.1.17	Mouse	9685330
ornithine decarboxylase	4.1.1.17	Mouse	9688665
ornithine decarboxylase	4.1.1.17	Mouse	9769382
ornithine decarboxylase	4.1.1.17	Mouse	9772292
ornithine decarboxylase	4.1.1.17	Mouse	9795249
ornithine decarboxylase	4.1.1.17	Mouse	9806166
ornithine decarboxylase	4.1.1.17	Mouse	9829706
ornithine decarboxylase	4.1.1.17	Mouse	9868187
ornithine decarboxylase	4.1.1.17	Mouse	9884080
ornithine decarboxylase	4.1.1.17	Mouse	9890191
arginine decarboxylase	4.1.1.19	Mouse	10420650
arginine decarboxylase	4.1.1.19	Mouse	11029703
arginine decarboxylase	4.1.1.19	Mouse	11540835
arginine decarboxylase	4.1.1.19	Mouse	11576438
arginine decarboxylase	4.1.1.19	Mouse	11903975
arginine decarboxylase	4.1.1.19	Mouse	12060267
arginine decarboxylase	4.1.1.19	Mouse	15032880
arginine decarboxylase	4.1.1.19	Mouse	15092366
arginine decarboxylase	4.1.1.19	Mouse	15120115
arginine decarboxylase	4.1.1.19	Mouse	15527979
arginine decarboxylase	4.1.1.19	Mouse	15733873
arginine decarboxylase	4.1.1.19	Mouse	16662219
arginine decarboxylase	4.1.1.19	Mouse	16663805
arginine decarboxylase	4.1.1.19	Mouse	16769152
arginine decarboxylase	4.1.1.19	Mouse	3931079
arginine decarboxylase	4.1.1.19	Mouse	9230111
arginine decarboxylase	4.1.1.19	Mouse	9624108
histidine decarboxylase	4.1.1.22	Mouse	10652359
histidine decarboxylase	4.1.1.22	Mouse	10843737
histidine decarboxylase	4.1.1.22	Mouse	11566179
histidine decarboxylase	4.1.1.22	Mouse	11827977
histidine decarboxylase	4.1.1.22	Mouse	12875237
histidine decarboxylase	4.1.1.22	Mouse	12960041
histidine decarboxylase	4.1.1.22	Mouse	15114681
histidine decarboxylase	4.1.1.22	Mouse	16203768
histidine decarboxylase	4.1.1.22	Mouse	16564918

histidine decarboxylase	4.1.1.22	Mouse	2889701
histidine decarboxylase	4.1.1.22	Mouse	3587507
histidine decarboxylase	4.1.1.22	Mouse	7742310
histidine decarboxylase	4.1.1.22	Mouse	9041686
aromatic-L-amino-acid decarboxylase	4.1.1.28	Mouse	10080715
aromatic-L-amino-acid decarboxylase	4.1.1.28	Mouse	10608659
aromatic-L-amino-acid decarboxylase	4.1.1.28	Mouse	12235825
aromatic-L-amino-acid decarboxylase	4.1.1.28	Mouse	15684695
aromatic-L-amino-acid decarboxylase	4.1.1.28	Mouse	15900211
aromatic-L-amino-acid decarboxylase	4.1.1.28	Mouse	15927700
aromatic-L-amino-acid decarboxylase	4.1.1.28	Mouse	16164646
aromatic-L-amino-acid decarboxylase	4.1.1.28	Mouse	17017570
aromatic-L-amino-acid decarboxylase	4.1.1.28	Mouse	1729407
aromatic-L-amino-acid decarboxylase	4.1.1.28	Mouse	6968497
aromatic-L-amino-acid decarboxylase	4.1.1.28	Mouse	7651438
aromatic-L-amino-acid decarboxylase	4.1.1.28	Mouse	9625353
sulfinoalanine decarboxylase	4.1.1.29	Mouse	10993179
phosphoenolpyruvate carboxykinase (GTP)	4.1.1.32	Mouse	11053047
phosphoenolpyruvate carboxykinase (GTP)	4.1.1.32	Mouse	11700062
phosphoenolpyruvate carboxykinase (GTP)	4.1.1.32	Mouse	11728630
phosphoenolpyruvate carboxykinase (GTP)	4.1.1.32	Mouse	11741859
phosphoenolpyruvate carboxykinase (GTP)	4.1.1.32	Mouse	11872659
phosphoenolpyruvate carboxykinase (GTP)	4.1.1.32	Mouse	11959679
phosphoenolpyruvate carboxykinase (GTP)	4.1.1.32	Mouse	12485530
phosphoenolpyruvate carboxykinase (GTP)	4.1.1.32	Mouse	12925798
phosphoenolpyruvate carboxykinase (GTP)	4.1.1.32	Mouse	14739078
phosphoenolpyruvate carboxykinase (GTP)	4.1.1.32	Mouse	15733733
phosphoenolpyruvate carboxykinase (GTP)	4.1.1.32	Mouse	15983413
phosphoenolpyruvate carboxykinase (GTP)	4.1.1.32	Mouse	16132948
phosphoenolpyruvate carboxykinase (GTP)	4.1.1.32	Mouse	16324924
phosphoenolpyruvate carboxykinase (GTP)	4.1.1.32	Mouse	16330542
phosphoenolpyruvate carboxykinase (GTP)	4.1.1.32	Mouse	16375695
phosphoenolpyruvate carboxykinase (GTP)	4.1.1.32	Mouse	16458327
phosphoenolpyruvate carboxykinase (GTP)	4.1.1.32	Mouse	1701430
phosphoenolpyruvate carboxykinase (GTP)	4.1.1.32	Mouse	17097062
phosphoenolpyruvate carboxykinase (GTP)	4.1.1.32	Mouse	1720862
phosphoenolpyruvate carboxykinase (GTP)	4.1.1.32	Mouse	17446233
phosphoenolpyruvate carboxykinase (GTP)	4.1.1.32	Mouse	2919162
phosphoenolpyruvate carboxykinase (GTP)	4.1.1.32	Mouse	3023262
phosphoenolpyruvate carboxykinase (GTP)	4.1.1.32	Mouse	3068502
phosphoenolpyruvate carboxykinase (GTP)	4.1.1.32	Mouse	4053567
phosphoenolpyruvate carboxykinase (GTP)	4.1.1.32	Mouse	6440018
phosphoenolpyruvate carboxykinase (GTP)	4.1.1.32	Mouse	7854322

phosphoenolpyruvate carboxykinase (GTP)	4.1.1.32	Mouse	8902188
phosphoenolpyruvate carboxykinase (GTP)	4.1.1.32	Mouse	9214454
phosphoenolpyruvate carboxykinase (GTP)	4.1.1.32	Mouse	9242918
phosphoenolpyruvate carboxykinase (GTP)	4.1.1.32	Mouse	9762796
adenosylmethionine decarboxylase	4.1.1.50	Mouse	10216947
adenosylmethionine decarboxylase	4.1.1.50	Mouse	10378277
adenosylmethionine decarboxylase	4.1.1.50	Mouse	10413038
adenosylmethionine decarboxylase	4.1.1.50	Mouse	10467042
adenosylmethionine decarboxylase	4.1.1.50	Mouse	10713131
adenosylmethionine decarboxylase	4.1.1.50	Mouse	10949915
adenosylmethionine decarboxylase	4.1.1.50	Mouse	11076965
adenosylmethionine decarboxylase	4.1.1.50	Mouse	11085920
adenosylmethionine decarboxylase	4.1.1.50	Mouse	11348531
adenosylmethionine decarboxylase	4.1.1.50	Mouse	11390378
adenosylmethionine decarboxylase	4.1.1.50	Mouse	11526206
adenosylmethionine decarboxylase	4.1.1.50	Mouse	11583148
adenosylmethionine decarboxylase	4.1.1.50	Mouse	11923270
adenosylmethionine decarboxylase	4.1.1.50	Mouse	12674502
adenosylmethionine decarboxylase	4.1.1.50	Mouse	14618239
adenosylmethionine decarboxylase	4.1.1.50	Mouse	15150268
adenosylmethionine decarboxylase	4.1.1.50	Mouse	15821146
adenosylmethionine decarboxylase	4.1.1.50	Mouse	16372273
adenosylmethionine decarboxylase	4.1.1.50	Mouse	16423999
adenosylmethionine decarboxylase	4.1.1.50	Mouse	16515461
adenosylmethionine decarboxylase	4.1.1.50	Mouse	16642382
adenosylmethionine decarboxylase	4.1.1.50	Mouse	16941339
adenosylmethionine decarboxylase	4.1.1.50	Mouse	2013278
adenosylmethionine decarboxylase	4.1.1.50	Mouse	2209170
adenosylmethionine decarboxylase	4.1.1.50	Mouse	2775206
adenosylmethionine decarboxylase	4.1.1.50	Mouse	3250232
adenosylmethionine decarboxylase	4.1.1.50	Mouse	4062886
adenosylmethionine decarboxylase	4.1.1.50	Mouse	7789170
adenosylmethionine decarboxylase	4.1.1.50	Mouse	7945201
adenosylmethionine decarboxylase	4.1.1.50	Mouse	8142949
adenosylmethionine decarboxylase	4.1.1.50	Mouse	8178573
adenosylmethionine decarboxylase	4.1.1.50	Mouse	8198469
adenosylmethionine decarboxylase	4.1.1.50	Mouse	8353934
adenosylmethionine decarboxylase	4.1.1.50	Mouse	8760129
adenosylmethionine decarboxylase	4.1.1.50	Mouse	8814137
adenosylmethionine decarboxylase	4.1.1.50	Mouse	8973561
adenosylmethionine decarboxylase	4.1.1.50	Mouse	9435790
adenosylmethionine decarboxylase	4.1.1.50	Mouse	9841864
adenosylmethionine decarboxylase	4.1.1.50	Mouse	9879883

fructose-bisphosphate aldolase	4.1.2.13	Mouse	10498814
fructose-bisphosphate aldolase	4.1.2.13	Mouse	12020659
fructose-bisphosphate aldolase	4.1.2.13	Mouse	12876349
fructose-bisphosphate aldolase	4.1.2.13	Mouse	15142555
fructose-bisphosphate aldolase	4.1.2.13	Mouse	15869466
fructose-bisphosphate aldolase	4.1.2.13	Mouse	16502329
fructose-bisphosphate aldolase	4.1.2.13	Mouse	1658253
fructose-bisphosphate aldolase	4.1.2.13	Mouse	1894606
fructose-bisphosphate aldolase	4.1.2.13	Mouse	6440018
fructose-bisphosphate aldolase	4.1.2.13	Mouse	678439
fructose-bisphosphate aldolase	4.1.2.13	Mouse	7388140
fructose-bisphosphate aldolase	4.1.2.13	Mouse	7786768
fructose-bisphosphate aldolase	4.1.2.13	Mouse	8015399
fructose-bisphosphate aldolase	4.1.2.13	Mouse	8913875
fructose-bisphosphate aldolase	4.1.2.13	Mouse	9163906
fructose-bisphosphate aldolase	4.1.2.13	Mouse	9473304
carbonate dehydratase	4.2.1.1	Mouse	16051345
carbonate dehydratase	4.2.1.1	Mouse	16310354
carbonate dehydratase	4.2.1.1	Mouse	1908243
carbonate dehydratase	4.2.1.1	Mouse	2117006
carbonate dehydratase	4.2.1.1	Mouse	3128444
carbonate dehydratase	4.2.1.1	Mouse	6408083
carbonate dehydratase	4.2.1.1	Mouse	6772280
carbonate dehydratase	4.2.1.1	Mouse	6776264
carbonate dehydratase	4.2.1.1	Mouse	6791257
carbonate dehydratase	4.2.1.1	Mouse	7899732
carbonate dehydratase	4.2.1.1	Mouse	9186493
carbonate dehydratase	4.2.1.1	Mouse	9486145
carbonate dehydratase	4.2.1.1	Mouse	9882455
phosphopyruvate hydratase	4.2.1.11	Mouse	15459207
phosphopyruvate hydratase	4.2.1.11	Mouse	17437631
phosphopyruvate hydratase	4.2.1.11	Mouse	8651685
cystathionine beta-synthase	4.2.1.22	Mouse	11051561
cystathionine beta-synthase	4.2.1.22	Mouse	11106665
cystathionine beta-synthase	4.2.1.22	Mouse	12198128
cystathionine beta-synthase	4.2.1.22	Mouse	15581575
cystathionine beta-synthase	4.2.1.22	Mouse	15642325
cystathionine beta-synthase	4.2.1.22	Mouse	16096271
cystathionine beta-synthase	4.2.1.22	Mouse	16769053
cystathionine beta-synthase	4.2.1.22	Mouse	17629356
porphobilinogen synthase	4.2.1.24	Mouse	10634305
porphobilinogen synthase	4.2.1.24	Mouse	10787385
porphobilinogen synthase	4.2.1.24	Mouse	15141099

porphobilinogen synthase	4.2.1.24	Mouse	15259603
porphobilinogen synthase	4.2.1.24	Mouse	1526942
porphobilinogen synthase	4.2.1.24	Mouse	16819823
porphobilinogen synthase	4.2.1.24	Mouse	1959865
porphobilinogen synthase	4.2.1.24	Mouse	2050126
porphobilinogen synthase	4.2.1.24	Mouse	2317819
porphobilinogen synthase	4.2.1.24	Mouse	3009001
porphobilinogen synthase	4.2.1.24	Mouse	3755290
porphobilinogen synthase	4.2.1.24	Mouse	3840094
porphobilinogen synthase	4.2.1.24	Mouse	3966797
porphobilinogen synthase	4.2.1.24	Mouse	4265023
porphobilinogen synthase	4.2.1.24	Mouse	6547609
porphobilinogen synthase	4.2.1.24	Mouse	6688350
porphobilinogen synthase	4.2.1.24	Mouse	6873612
porphobilinogen synthase	4.2.1.24	Mouse	7436670
aconitate hydratase	4.2.1.3	Mouse	11295257
aconitate hydratase	4.2.1.3	Mouse	11329290
aconitate hydratase	4.2.1.3	Mouse	15149735
aconitate hydratase	4.2.1.3	Mouse	15543948
aconitate hydratase	4.2.1.3	Mouse	16094633
aconitate hydratase	4.2.1.3	Mouse	16201454
aconitate hydratase	4.2.1.3	Mouse	7589784
aconitate hydratase	4.2.1.3	Mouse	8115279
6-pyruvoyltetrahydropterin synthase	4.2.3.12	Mouse	11022034
6-pyruvoyltetrahydropterin synthase	4.2.3.12	Mouse	11744995
6-pyruvoyltetrahydropterin synthase	4.2.3.12	Mouse	11778454
6-pyruvoyltetrahydropterin synthase	4.2.3.12	Mouse	7545485
6-pyruvoyltetrahydropterin synthase	4.2.3.12	Mouse	8307017
6-pyruvoyltetrahydropterin synthase	4.2.3.12	Mouse	9788822
cystathionine gamma-lyase	4.4.1.1	Mouse	10960449
cystathionine gamma-lyase	4.4.1.1	Mouse	15038791
cystathionine gamma-lyase	4.4.1.1	Mouse	15347670
cystathionine gamma-lyase	4.4.1.1	Mouse	16786305
cystathionine gamma-lyase	4.4.1.1	Mouse	16946488
cystathionine gamma-lyase	4.4.1.1	Mouse	17095121
cystathionine gamma-lyase	4.4.1.1	Mouse	17525332
cystathionine gamma-lyase	4.4.1.1	Mouse	8973544
leukotriene-C4 synthase	4.4.1.20	Mouse	10222453
leukotriene-C4 synthase	4.4.1.20	Mouse	11964666
leukotriene-C4 synthase	4.4.1.20	Mouse	15730873
leukotriene-C4 synthase	4.4.1.20	Mouse	17397868
leukotriene-C4 synthase	4.4.1.20	Mouse	9431445
UDP-glucose 4-epimerase	5.1.3.2	Mouse	1149741

maleylacetoacetate isomerase	5.2.1.2	Mouse	11327815
maleylacetoacetate isomerase	5.2.1.2	Mouse	9734339
steroid Delta-isomerase	5.3.3.1	Mouse	239964
phosphoglycerate mutase	5.4.2.1	Mouse	15181008
phosphoglycerate mutase	5.4.2.1	Mouse	17204863
phosphoglycerate mutase	5.4.2.1	Mouse	2830218
phosphoglucomutase	5.4.2.2	Mouse	11102370
phosphoglucomutase	5.4.2.2	Mouse	1149741
phosphoglucomutase	5.4.2.2	Mouse	12026175
phosphoglucomutase	5.4.2.2	Mouse	12791685
phosphoglucomutase	5.4.2.2	Mouse	15378030
phosphoglucomutase	5.4.2.2	Mouse	15996095
phosphoglucomutase	5.4.2.2	Mouse	16046289
lanosterol synthase	5.4.99.7	Mouse	11995928
lanosterol synthase	5.4.99.7	Mouse	15763540
lanosterol synthase	5.4.99.7	Mouse	17088293
acetate---CoA ligase	6.2.1.1	Mouse	15236963
acetate---CoA ligase	6.2.1.1	Mouse	15899897
acetate---CoA ligase	6.2.1.1	Mouse	2902801
acetate---CoA ligase	6.2.1.1	Mouse	4149947
acetate---CoA ligase	6.2.1.1	Mouse	8218953
long-chain-fatty-acid---CoA ligase	6.2.1.3	Mouse	11375393
long-chain-fatty-acid---CoA ligase	6.2.1.3	Mouse	1769731
succinate---CoA ligase (ADP-forming)	6.2.1.5	Mouse	16101500
succinate---CoA ligase (ADP-forming)	6.2.1.5	Mouse	1986797
succinate---CoA ligase (ADP-forming)	6.2.1.5	Mouse	3108130
succinate---CoA ligase (ADP-forming)	6.2.1.5	Mouse	3746465
succinate---CoA ligase (ADP-forming)	6.2.1.5	Mouse	6109001
succinate---CoA ligase (ADP-forming)	6.2.1.5	Mouse	7017725
succinate---CoA ligase (ADP-forming)	6.2.1.5	Mouse	7062046
succinate---CoA ligase (ADP-forming)	6.2.1.5	Mouse	7783627
glutamate---ammonia ligase	6.3.1.2	Mouse	10092169
glutamate---ammonia ligase	6.3.1.2	Mouse	10383611
glutamate---ammonia ligase	6.3.1.2	Mouse	10440891
glutamate---ammonia ligase	6.3.1.2	Mouse	10664131
glutamate---ammonia ligase	6.3.1.2	Mouse	11069692
glutamate---ammonia ligase	6.3.1.2	Mouse	11413247
glutamate---ammonia ligase	6.3.1.2	Mouse	11939529
glutamate---ammonia ligase	6.3.1.2	Mouse	12232192
glutamate---ammonia ligase	6.3.1.2	Mouse	12517141
glutamate---ammonia ligase	6.3.1.2	Mouse	12552916
glutamate---ammonia ligase	6.3.1.2	Mouse	14714472
glutamate---ammonia ligase	6.3.1.2	Mouse	14723991

glutamate---ammonia ligase	6.3.1.2	Mouse	15130478
glutamate---ammonia ligase	6.3.1.2	Mouse	15489445
glutamate---ammonia ligase	6.3.1.2	Mouse	15581577
glutamate---ammonia ligase	6.3.1.2	Mouse	1612427
glutamate---ammonia ligase	6.3.1.2	Mouse	16687472
glutamate---ammonia ligase	6.3.1.2	Mouse	16829528
glutamate---ammonia ligase	6.3.1.2	Mouse	16946267
glutamate---ammonia ligase	6.3.1.2	Mouse	2434618
glutamate---ammonia ligase	6.3.1.2	Mouse	26663
glutamate---ammonia ligase	6.3.1.2	Mouse	6118373
glutamate---ammonia ligase	6.3.1.2	Mouse	9624228
glutamate---cysteine ligase	6.3.2.2	Mouse	10215022
glutamate---cysteine ligase	6.3.2.2	Mouse	10218647
glutamate---cysteine ligase	6.3.2.2	Mouse	10349842
glutamate---cysteine ligase	6.3.2.2	Mouse	10385608
glutamate---cysteine ligase	6.3.2.2	Mouse	10385658
glutamate---cysteine ligase	6.3.2.2	Mouse	10399958
glutamate---cysteine ligase	6.3.2.2	Mouse	10439045
glutamate---cysteine ligase	6.3.2.2	Mouse	10441483
glutamate---cysteine ligase	6.3.2.2	Mouse	10486302
glutamate---cysteine ligase	6.3.2.2	Mouse	10515588
glutamate---cysteine ligase	6.3.2.2	Mouse	10518117
glutamate---cysteine ligase	6.3.2.2	Mouse	10544055
glutamate---cysteine ligase	6.3.2.2	Mouse	10544272
glutamate---cysteine ligase	6.3.2.2	Mouse	10590319
glutamate---cysteine ligase	6.3.2.2	Mouse	10593589
glutamate---cysteine ligase	6.3.2.2	Mouse	10594104
glutamate---cysteine ligase	6.3.2.2	Mouse	10600876
glutamate---cysteine ligase	6.3.2.2	Mouse	10623879
glutamate---cysteine ligase	6.3.2.2	Mouse	10644053
glutamate---cysteine ligase	6.3.2.2	Mouse	10674357
glutamate---cysteine ligase	6.3.2.2	Mouse	10677377
glutamate---cysteine ligase	6.3.2.2	Mouse	10702364
glutamate---cysteine ligase	6.3.2.2	Mouse	10719238
glutamate---cysteine ligase	6.3.2.2	Mouse	10733945
glutamate---cysteine ligase	6.3.2.2	Mouse	10748080
glutamate---cysteine ligase	6.3.2.2	Mouse	10773686
glutamate---cysteine ligase	6.3.2.2	Mouse	10777712
glutamate---cysteine ligase	6.3.2.2	Mouse	10802223
glutamate---cysteine ligase	6.3.2.2	Mouse	10928075
glutamate---cysteine ligase	6.3.2.2	Mouse	10960449
glutamate---cysteine ligase	6.3.2.2	Mouse	11007940
glutamate---cysteine ligase	6.3.2.2	Mouse	11025451

glutamate---cysteine ligase	6.3.2.2	Mouse	11028671
glutamate---cysteine ligase	6.3.2.2	Mouse	11032771
glutamate---cysteine ligase	6.3.2.2	Mouse	11097862
glutamate---cysteine ligase	6.3.2.2	Mouse	11133045
glutamate---cysteine ligase	6.3.2.2	Mouse	11157875
glutamate---cysteine ligase	6.3.2.2	Mouse	11163433
glutamate---cysteine ligase	6.3.2.2	Mouse	11181934
glutamate---cysteine ligase	6.3.2.2	Mouse	11233143
glutamate---cysteine ligase	6.3.2.2	Mouse	11306445
glutamate---cysteine ligase	6.3.2.2	Mouse	11339815
glutamate---cysteine ligase	6.3.2.2	Mouse	11352989
glutamate---cysteine ligase	6.3.2.2	Mouse	11353135
glutamate---cysteine ligase	6.3.2.2	Mouse	11500053
glutamate---cysteine ligase	6.3.2.2	Mouse	11560771
glutamate---cysteine ligase	6.3.2.2	Mouse	11565956
glutamate---cysteine ligase	6.3.2.2	Mouse	11687904
glutamate---cysteine ligase	6.3.2.2	Mouse	11705692
glutamate---cysteine ligase	6.3.2.2	Mouse	11780957
glutamate---cysteine ligase	6.3.2.2	Mouse	11781188
glutamate---cysteine ligase	6.3.2.2	Mouse	11790356
glutamate---cysteine ligase	6.3.2.2	Mouse	11812649
glutamate---cysteine ligase	6.3.2.2	Mouse	11820781
glutamate---cysteine ligase	6.3.2.2	Mouse	11841806
glutamate---cysteine ligase	6.3.2.2	Mouse	11849402
glutamate---cysteine ligase	6.3.2.2	Mouse	11876501
glutamate---cysteine ligase	6.3.2.2	Mouse	11970852
glutamate---cysteine ligase	6.3.2.2	Mouse	12079521
glutamate---cysteine ligase	6.3.2.2	Mouse	12111865
glutamate---cysteine ligase	6.3.2.2	Mouse	12147223
glutamate---cysteine ligase	6.3.2.2	Mouse	12196927
glutamate---cysteine ligase	6.3.2.2	Mouse	12200125
glutamate---cysteine ligase	6.3.2.2	Mouse	12204877
glutamate---cysteine ligase	6.3.2.2	Mouse	12433058
glutamate---cysteine ligase	6.3.2.2	Mouse	12448821
glutamate---cysteine ligase	6.3.2.2	Mouse	12452384
glutamate---cysteine ligase	6.3.2.2	Mouse	12535742
glutamate---cysteine ligase	6.3.2.2	Mouse	12594957
glutamate---cysteine ligase	6.3.2.2	Mouse	12601050
glutamate---cysteine ligase	6.3.2.2	Mouse	12607907
glutamate---cysteine ligase	6.3.2.2	Mouse	12628495
glutamate---cysteine ligase	6.3.2.2	Mouse	12637989
glutamate---cysteine ligase	6.3.2.2	Mouse	12814619
glutamate---cysteine ligase	6.3.2.2	Mouse	12882455

glutamate---cysteine ligase	6.3.2.2	Mouse	12913252
glutamate---cysteine ligase	6.3.2.2	Mouse	1350904
glutamate---cysteine ligase	6.3.2.2	Mouse	1351382
glutamate---cysteine ligase	6.3.2.2	Mouse	1353765
glutamate---cysteine ligase	6.3.2.2	Mouse	1355406
glutamate---cysteine ligase	6.3.2.2	Mouse	1362226
glutamate---cysteine ligase	6.3.2.2	Mouse	13679058
glutamate---cysteine ligase	6.3.2.2	Mouse	14500406
glutamate---cysteine ligase	6.3.2.2	Mouse	14514673
glutamate---cysteine ligase	6.3.2.2	Mouse	14744626
glutamate---cysteine ligase	6.3.2.2	Mouse	14962359
glutamate---cysteine ligase	6.3.2.2	Mouse	15020643
glutamate---cysteine ligase	6.3.2.2	Mouse	15050748
glutamate---cysteine ligase	6.3.2.2	Mouse	15257546
glutamate---cysteine ligase	6.3.2.2	Mouse	15314090
glutamate---cysteine ligase	6.3.2.2	Mouse	15374419
glutamate---cysteine ligase	6.3.2.2	Mouse	15451066
glutamate---cysteine ligase	6.3.2.2	Mouse	15477603
glutamate---cysteine ligase	6.3.2.2	Mouse	15509664
glutamate---cysteine ligase	6.3.2.2	Mouse	15878398
glutamate---cysteine ligase	6.3.2.2	Mouse	15946948
glutamate---cysteine ligase	6.3.2.2	Mouse	16032782
glutamate---cysteine ligase	6.3.2.2	Mouse	16081425
glutamate---cysteine ligase	6.3.2.2	Mouse	16162662
glutamate---cysteine ligase	6.3.2.2	Mouse	16183645
glutamate---cysteine ligase	6.3.2.2	Mouse	16549430
glutamate---cysteine ligase	6.3.2.2	Mouse	16566126
glutamate---cysteine ligase	6.3.2.2	Mouse	1678010
glutamate---cysteine ligase	6.3.2.2	Mouse	16781460
glutamate---cysteine ligase	6.3.2.2	Mouse	16949561
glutamate---cysteine ligase	6.3.2.2	Mouse	16960387
glutamate---cysteine ligase	6.3.2.2	Mouse	17144898
glutamate---cysteine ligase	6.3.2.2	Mouse	1784629
glutamate---cysteine ligase	6.3.2.2	Mouse	1970723
glutamate---cysteine ligase	6.3.2.2	Mouse	1997009
glutamate---cysteine ligase	6.3.2.2	Mouse	2572174
glutamate---cysteine ligase	6.3.2.2	Mouse	2574245
glutamate---cysteine ligase	6.3.2.2	Mouse	2879531
glutamate---cysteine ligase	6.3.2.2	Mouse	2897858
glutamate---cysteine ligase	6.3.2.2	Mouse	2901982
glutamate---cysteine ligase	6.3.2.2	Mouse	3621155
glutamate---cysteine ligase	6.3.2.2	Mouse	6897891
glutamate---cysteine ligase	6.3.2.2	Mouse	7351635

glutamate---cysteine ligase	6.3.2.2	Mouse	7503776
glutamate---cysteine ligase	6.3.2.2	Mouse	7568279
glutamate---cysteine ligase	6.3.2.2	Mouse	7570642
glutamate---cysteine ligase	6.3.2.2	Mouse	7585502
glutamate---cysteine ligase	6.3.2.2	Mouse	7622006
glutamate---cysteine ligase	6.3.2.2	Mouse	7651354
glutamate---cysteine ligase	6.3.2.2	Mouse	7768207
glutamate---cysteine ligase	6.3.2.2	Mouse	7901332
glutamate---cysteine ligase	6.3.2.2	Mouse	7908245
glutamate---cysteine ligase	6.3.2.2	Mouse	7910419
glutamate---cysteine ligase	6.3.2.2	Mouse	7929374
glutamate---cysteine ligase	6.3.2.2	Mouse	7955076
glutamate---cysteine ligase	6.3.2.2	Mouse	7969079
glutamate---cysteine ligase	6.3.2.2	Mouse	8001239
glutamate---cysteine ligase	6.3.2.2	Mouse	8065332
glutamate---cysteine ligase	6.3.2.2	Mouse	8101766
glutamate---cysteine ligase	6.3.2.2	Mouse	8106072
glutamate---cysteine ligase	6.3.2.2	Mouse	8120650
glutamate---cysteine ligase	6.3.2.2	Mouse	8538700
glutamate---cysteine ligase	6.3.2.2	Mouse	8582653
glutamate---cysteine ligase	6.3.2.2	Mouse	8648118
glutamate---cysteine ligase	6.3.2.2	Mouse	8661240
glutamate---cysteine ligase	6.3.2.2	Mouse	8751598
glutamate---cysteine ligase	6.3.2.2	Mouse	8781554
glutamate---cysteine ligase	6.3.2.2	Mouse	8792848
glutamate---cysteine ligase	6.3.2.2	Mouse	8806884
glutamate---cysteine ligase	6.3.2.2	Mouse	8843715
glutamate---cysteine ligase	6.3.2.2	Mouse	8917676
glutamate---cysteine ligase	6.3.2.2	Mouse	8930687
glutamate---cysteine ligase	6.3.2.2	Mouse	8947504
glutamate---cysteine ligase	6.3.2.2	Mouse	8973794
glutamate---cysteine ligase	6.3.2.2	Mouse	8981036
glutamate---cysteine ligase	6.3.2.2	Mouse	8995480
glutamate---cysteine ligase	6.3.2.2	Mouse	9029270
glutamate---cysteine ligase	6.3.2.2	Mouse	9054446
glutamate---cysteine ligase	6.3.2.2	Mouse	9063478
glutamate---cysteine ligase	6.3.2.2	Mouse	9093011
glutamate---cysteine ligase	6.3.2.2	Mouse	9119067
glutamate---cysteine ligase	6.3.2.2	Mouse	9157984
glutamate---cysteine ligase	6.3.2.2	Mouse	9163779
glutamate---cysteine ligase	6.3.2.2	Mouse	9185621
glutamate---cysteine ligase	6.3.2.2	Mouse	9214623
glutamate---cysteine ligase	6.3.2.2	Mouse	9259355

glutamate---cysteine ligase	6.3.2.2	Mouse	9268987
glutamate---cysteine ligase	6.3.2.2	Mouse	9288403
glutamate---cysteine ligase	6.3.2.2	Mouse	9311606
glutamate---cysteine ligase	6.3.2.2	Mouse	9374111
glutamate---cysteine ligase	6.3.2.2	Mouse	9389600
glutamate---cysteine ligase	6.3.2.2	Mouse	9393741
glutamate---cysteine ligase	6.3.2.2	Mouse	9425930
glutamate---cysteine ligase	6.3.2.2	Mouse	9582278
glutamate---cysteine ligase	6.3.2.2	Mouse	9614065
glutamate---cysteine ligase	6.3.2.2	Mouse	9626582
glutamate---cysteine ligase	6.3.2.2	Mouse	9647756
glutamate---cysteine ligase	6.3.2.2	Mouse	9679558
glutamate---cysteine ligase	6.3.2.2	Mouse	9703946
glutamate---cysteine ligase	6.3.2.2	Mouse	9729439
glutamate---cysteine ligase	6.3.2.2	Mouse	9750167
glutamate---cysteine ligase	6.3.2.2	Mouse	9756861
glutamate---cysteine ligase	6.3.2.2	Mouse	9762423
glutamate---cysteine ligase	6.3.2.2	Mouse	9875552
glutamate---cysteine ligase	6.3.2.2	Mouse	9895302
glutathione synthase	6.3.2.3	Mouse	10964706
glutathione synthase	6.3.2.3	Mouse	11708780
glutathione synthase	6.3.2.3	Mouse	9880348
phosphoribosylaminoimidazolesuccinocarboxamide synthase	6.3.2.6	Mouse	701284
carbamoyl-phosphate synthase (ammonia)	6.3.4.16	Mouse	10047492
carbamoyl-phosphate synthase (ammonia)	6.3.4.16	Mouse	11872754
carbamoyl-phosphate synthase (ammonia)	6.3.4.16	Mouse	189805
carbamoyl-phosphate synthase (ammonia)	6.3.4.16	Mouse	7459875
carbamoyl-phosphate synthase (ammonia)	6.3.4.16	Mouse	7587391
carbamoyl-phosphate synthase (ammonia)	6.3.4.16	Mouse	8752005
CTP synthase	6.3.4.2	Mouse	12678497
CTP synthase	6.3.4.2	Mouse	16820675
CTP synthase	6.3.4.2	Mouse	17463002
CTP synthase	6.3.4.2	Mouse	17681942
CTP synthase	6.3.4.2	Mouse	2787169
argininosuccinate synthase	6.3.4.5	Mouse	10709858
argininosuccinate synthase	6.3.4.5	Mouse	1122920
argininosuccinate synthase	6.3.4.5	Mouse	11556547
argininosuccinate synthase	6.3.4.5	Mouse	12618329
argininosuccinate synthase	6.3.4.5	Mouse	12672181
argininosuccinate synthase	6.3.4.5	Mouse	1372742
argininosuccinate synthase	6.3.4.5	Mouse	14571701
argininosuccinate synthase	6.3.4.5	Mouse	15588718

argininosuccinate synthase	6.3.4.5	Mouse	16085056
argininosuccinate synthase	6.3.4.5	Mouse	16787144
argininosuccinate synthase	6.3.4.5	Mouse	558104
argininosuccinate synthase	6.3.4.5	Mouse	845694
argininosuccinate synthase	6.3.4.5	Mouse	8616812
argininosuccinate synthase	6.3.4.5	Mouse	8798625
argininosuccinate synthase	6.3.4.5	Mouse	9096605
argininosuccinate synthase	6.3.4.5	Mouse	9176259
argininosuccinate synthase	6.3.4.5	Mouse	9211993
argininosuccinate synthase	6.3.4.5	Mouse	9252090
carbamoyl-phosphate synthase (glutamine-hydrolysing)	6.3.5.5	Mouse	10659854
carbamoyl-phosphate synthase (glutamine-hydrolysing)	6.3.5.5	Mouse	10736367
carbamoyl-phosphate synthase (glutamine-hydrolysing)	6.3.5.5	Mouse	11441057
carbamoyl-phosphate synthase (glutamine-hydrolysing)	6.3.5.5	Mouse	11872754
carbamoyl-phosphate synthase (glutamine-hydrolysing)	6.3.5.5	Mouse	11956684
carbamoyl-phosphate synthase (glutamine-hydrolysing)	6.3.5.5	Mouse	12678497
carbamoyl-phosphate synthase (glutamine-hydrolysing)	6.3.5.5	Mouse	15326225
carbamoyl-phosphate synthase (glutamine-hydrolysing)	6.3.5.5	Mouse	15453495
carbamoyl-phosphate synthase (glutamine-hydrolysing)	6.3.5.5	Mouse	4018077
carbamoyl-phosphate synthase (glutamine-hydrolysing)	6.3.5.5	Mouse	4.0927E+13
carbamoyl-phosphate synthase (glutamine-hydrolysing)	6.3.5.5	Mouse	6115855
carbamoyl-phosphate synthase (glutamine-hydrolysing)	6.3.5.5	Mouse	6408083
carbamoyl-phosphate synthase (glutamine-hydrolysing)	6.3.5.5	Mouse	7053379
carbamoyl-phosphate synthase (glutamine-hydrolysing)	6.3.5.5	Mouse	7209543
carbamoyl-phosphate synthase (glutamine-hydrolysing)	6.3.5.5	Mouse	7608487
carbamoyl-phosphate synthase (glutamine-hydrolysing)	6.3.5.5	Mouse	7916269
pyruvate carboxylase	6.4.1.1	Mouse	10323732

pyruvate carboxylase	6.4.1.1	Mouse	16325442
pyruvate carboxylase	6.4.1.1	Mouse	3182810
pyruvate carboxylase	6.4.1.1	Mouse	6721853
acetyl-CoA carboxylase	6.4.1.2	Mouse	10098661
acetyl-CoA carboxylase	6.4.1.2	Mouse	10215591
acetyl-CoA carboxylase	6.4.1.2	Mouse	10757783
acetyl-CoA carboxylase	6.4.1.2	Mouse	10945143
acetyl-CoA carboxylase	6.4.1.2	Mouse	11078738
acetyl-CoA carboxylase	6.4.1.2	Mouse	11205884
acetyl-CoA carboxylase	6.4.1.2	Mouse	11504381
acetyl-CoA carboxylase	6.4.1.2	Mouse	11515553
acetyl-CoA carboxylase	6.4.1.2	Mouse	11546765
acetyl-CoA carboxylase	6.4.1.2	Mouse	12440972
acetyl-CoA carboxylase	6.4.1.2	Mouse	14627750
acetyl-CoA carboxylase	6.4.1.2	Mouse	15333468
acetyl-CoA carboxylase	6.4.1.2	Mouse	15607423
acetyl-CoA carboxylase	6.4.1.2	Mouse	15607568
acetyl-CoA carboxylase	6.4.1.2	Mouse	16222055
acetyl-CoA carboxylase	6.4.1.2	Mouse	16707454
acetyl-CoA carboxylase	6.4.1.2	Mouse	16968879
acetyl-CoA carboxylase	6.4.1.2	Mouse	17266990
acetyl-CoA carboxylase	6.4.1.2	Mouse	17653193
acetyl-CoA carboxylase	6.4.1.2	Mouse	1978829
acetyl-CoA carboxylase	6.4.1.2	Mouse	2570725
acetyl-CoA carboxylase	6.4.1.2	Mouse	2861941
acetyl-CoA carboxylase	6.4.1.2	Mouse	2894828
acetyl-CoA carboxylase	6.4.1.2	Mouse	7436865
acetyl-CoA carboxylase	6.4.1.2	Mouse	7903266
acetyl-CoA carboxylase	6.4.1.2	Mouse	7915138
acetyl-CoA carboxylase	6.4.1.2	Mouse	8814137
acetyl-CoA carboxylase	6.4.1.2	Mouse	9028876
acetyl-CoA carboxylase	6.4.1.2	Mouse	9082912
acetyl-CoA carboxylase	6.4.1.2	Mouse	9109840
alcohol dehydrogenase	1.1.1.1	Rat	1096557
alcohol dehydrogenase	1.1.1.1	Rat	11303599
alcohol dehydrogenase	1.1.1.1	Rat	1148277
alcohol dehydrogenase	1.1.1.1	Rat	12147722
alcohol dehydrogenase	1.1.1.1	Rat	12489977
alcohol dehydrogenase	1.1.1.1	Rat	16662798
alcohol dehydrogenase	1.1.1.1	Rat	167557
alcohol dehydrogenase	1.1.1.1	Rat	2932116
alcohol dehydrogenase	1.1.1.1	Rat	3067025
alcohol dehydrogenase	1.1.1.1	Rat	3893194

alcohol dehydrogenase	1.1.1.1	Rat	4038269
alcohol dehydrogenase	1.1.1.1	Rat	6340613
alcohol dehydrogenase	1.1.1.1	Rat	6341787
alcohol dehydrogenase	1.1.1.1	Rat	6356161
alcohol dehydrogenase	1.1.1.1	Rat	6363888
alcohol dehydrogenase	1.1.1.1	Rat	6370140
alcohol dehydrogenase	1.1.1.1	Rat	8277258
alcohol dehydrogenase	1.1.1.1	Rat	8692838
alcohol dehydrogenase	1.1.1.1	Rat	8905240
alcohol dehydrogenase	1.1.1.1	Rat	9526508
3beta-hydroxy-Delta5-steroid dehydrogenase	1.1.1.145	Rat	12441193
3beta-hydroxy-Delta5-steroid dehydrogenase	1.1.1.145	Rat	12782399
3beta-hydroxy-Delta5-steroid dehydrogenase	1.1.1.145	Rat	2019257
3beta-hydroxy-Delta5-steroid dehydrogenase	1.1.1.145	Rat	239964
3beta-hydroxy-Delta5-steroid dehydrogenase	1.1.1.145	Rat	7736258
3beta-hydroxy-Delta5-steroid dehydrogenase	1.1.1.145	Rat	8574339
IMP dehydrogenase	1.1.1.205	Rat	10194364
IMP dehydrogenase	1.1.1.205	Rat	10390601
IMP dehydrogenase	1.1.1.205	Rat	10390603
IMP dehydrogenase	1.1.1.205	Rat	10391669
IMP dehydrogenase	1.1.1.205	Rat	10417742
IMP dehydrogenase	1.1.1.205	Rat	10930578
IMP dehydrogenase	1.1.1.205	Rat	10953035
IMP dehydrogenase	1.1.1.205	Rat	10953295
IMP dehydrogenase	1.1.1.205	Rat	10973868
IMP dehydrogenase	1.1.1.205	Rat	11003640
IMP dehydrogenase	1.1.1.205	Rat	1106431
IMP dehydrogenase	1.1.1.205	Rat	11076502
IMP dehydrogenase	1.1.1.205	Rat	11145582
IMP dehydrogenase	1.1.1.205	Rat	11223253
IMP dehydrogenase	1.1.1.205	Rat	11233304
IMP dehydrogenase	1.1.1.205	Rat	11288107
IMP dehydrogenase	1.1.1.205	Rat	11454943
IMP dehydrogenase	1.1.1.205	Rat	11522119
IMP dehydrogenase	1.1.1.205	Rat	11566360
IMP dehydrogenase	1.1.1.205	Rat	11712223
IMP dehydrogenase	1.1.1.205	Rat	11724288

IMP dehydrogenase	1.1.1.205	Rat	11875050
IMP dehydrogenase	1.1.1.205	Rat	11966437
IMP dehydrogenase	1.1.1.205	Rat	11966440
IMP dehydrogenase	1.1.1.205	Rat	11966441
IMP dehydrogenase	1.1.1.205	Rat	12014950
IMP dehydrogenase	1.1.1.205	Rat	12183689
IMP dehydrogenase	1.1.1.205	Rat	12213477
IMP dehydrogenase	1.1.1.205	Rat	12235158
IMP dehydrogenase	1.1.1.205	Rat	12403633
IMP dehydrogenase	1.1.1.205	Rat	12559919
IMP dehydrogenase	1.1.1.205	Rat	12609835
IMP dehydrogenase	1.1.1.205	Rat	12746440
IMP dehydrogenase	1.1.1.205	Rat	12773970
IMP dehydrogenase	1.1.1.205	Rat	12944494
IMP dehydrogenase	1.1.1.205	Rat	1353938
IMP dehydrogenase	1.1.1.205	Rat	1356621
IMP dehydrogenase	1.1.1.205	Rat	14703952
IMP dehydrogenase	1.1.1.205	Rat	14757177
IMP dehydrogenase	1.1.1.205	Rat	14766016
IMP dehydrogenase	1.1.1.205	Rat	14973196
IMP dehydrogenase	1.1.1.205	Rat	14981049
IMP dehydrogenase	1.1.1.205	Rat	15043157
IMP dehydrogenase	1.1.1.205	Rat	15083807
IMP dehydrogenase	1.1.1.205	Rat	15292516
IMP dehydrogenase	1.1.1.205	Rat	15355510
IMP dehydrogenase	1.1.1.205	Rat	15829418
IMP dehydrogenase	1.1.1.205	Rat	15869715
IMP dehydrogenase	1.1.1.205	Rat	15882147
IMP dehydrogenase	1.1.1.205	Rat	15940263
IMP dehydrogenase	1.1.1.205	Rat	16128570
IMP dehydrogenase	1.1.1.205	Rat	16243838
IMP dehydrogenase	1.1.1.205	Rat	16248022
IMP dehydrogenase	1.1.1.205	Rat	16333815
IMP dehydrogenase	1.1.1.205	Rat	16647299
IMP dehydrogenase	1.1.1.205	Rat	16725387
IMP dehydrogenase	1.1.1.205	Rat	1677309
IMP dehydrogenase	1.1.1.205	Rat	16936083
IMP dehydrogenase	1.1.1.205	Rat	17100698
IMP dehydrogenase	1.1.1.205	Rat	1717828
IMP dehydrogenase	1.1.1.205	Rat	1723703
IMP dehydrogenase	1.1.1.205	Rat	1975748
IMP dehydrogenase	1.1.1.205	Rat	197916
IMP dehydrogenase	1.1.1.205	Rat	2902093

IMP dehydrogenase	1.1.1.205	Rat	3314714
IMP dehydrogenase	1.1.1.205	Rat	4868171
IMP dehydrogenase	1.1.1.205	Rat	6120758
IMP dehydrogenase	1.1.1.205	Rat	7476879
IMP dehydrogenase	1.1.1.205	Rat	7476895
IMP dehydrogenase	1.1.1.205	Rat	7520100
IMP dehydrogenase	1.1.1.205	Rat	7874783
IMP dehydrogenase	1.1.1.205	Rat	7903533
IMP dehydrogenase	1.1.1.205	Rat	7914720
IMP dehydrogenase	1.1.1.205	Rat	8103312
IMP dehydrogenase	1.1.1.205	Rat	8555204
IMP dehydrogenase	1.1.1.205	Rat	8560580
IMP dehydrogenase	1.1.1.205	Rat	8830834
IMP dehydrogenase	1.1.1.205	Rat	8869741
IMP dehydrogenase	1.1.1.205	Rat	8910338
IMP dehydrogenase	1.1.1.205	Rat	9042309
IMP dehydrogenase	1.1.1.205	Rat	9108641
IMP dehydrogenase	1.1.1.205	Rat	9268334
IMP dehydrogenase	1.1.1.205	Rat	9278455
IMP dehydrogenase	1.1.1.205	Rat	9339960
IMP dehydrogenase	1.1.1.205	Rat	9399601
IMP dehydrogenase	1.1.1.205	Rat	9413163
IMP dehydrogenase	1.1.1.205	Rat	9436988
IMP dehydrogenase	1.1.1.205	Rat	9752721
IMP dehydrogenase	1.1.1.205	Rat	9766533
IMP dehydrogenase	1.1.1.205	Rat	9881055
aldehyde reductase	1.1.1.21	Rat	10424772
aldehyde reductase	1.1.1.21	Rat	10656235
aldehyde reductase	1.1.1.21	Rat	11095596
aldehyde reductase	1.1.1.21	Rat	11370705
aldehyde reductase	1.1.1.21	Rat	11422753
aldehyde reductase	1.1.1.21	Rat	11440832
aldehyde reductase	1.1.1.21	Rat	11798960
aldehyde reductase	1.1.1.21	Rat	12135102
aldehyde reductase	1.1.1.21	Rat	12363257
aldehyde reductase	1.1.1.21	Rat	12394272
aldehyde reductase	1.1.1.21	Rat	12871133
aldehyde reductase	1.1.1.21	Rat	12871136
aldehyde reductase	1.1.1.21	Rat	12881532
aldehyde reductase	1.1.1.21	Rat	1393828
aldehyde reductase	1.1.1.21	Rat	14577653
aldehyde reductase	1.1.1.21	Rat	1499867
aldehyde reductase	1.1.1.21	Rat	15210146

aldehyde reductase	1.1.1.21	Rat	15569136
aldehyde reductase	1.1.1.21	Rat	15584919
aldehyde reductase	1.1.1.21	Rat	15734861
aldehyde reductase	1.1.1.21	Rat	15736047
aldehyde reductase	1.1.1.21	Rat	16026266
aldehyde reductase	1.1.1.21	Rat	16037296
aldehyde reductase	1.1.1.21	Rat	16048249
aldehyde reductase	1.1.1.21	Rat	16114079
aldehyde reductase	1.1.1.21	Rat	16452468
aldehyde reductase	1.1.1.21	Rat	16806328
aldehyde reductase	1.1.1.21	Rat	16870454
aldehyde reductase	1.1.1.21	Rat	16900332
aldehyde reductase	1.1.1.21	Rat	16911628
aldehyde reductase	1.1.1.21	Rat	2120282
aldehyde reductase	1.1.1.21	Rat	3025043
aldehyde reductase	1.1.1.21	Rat	7641310
aldehyde reductase	1.1.1.21	Rat	8457142
aldehyde reductase	1.1.1.21	Rat	9215310
aldehyde reductase	1.1.1.21	Rat	9454604
aldehyde reductase	1.1.1.21	Rat	9481088
aldehyde reductase	1.1.1.21	Rat	9709964
UDP-glucose 6-dehydrogenase	1.1.1.22	Rat	11044215
UDP-glucose 6-dehydrogenase	1.1.1.22	Rat	15741737
UDP-glucose 6-dehydrogenase	1.1.1.22	Rat	2778766
UDP-glucose 6-dehydrogenase	1.1.1.22	Rat	8471533
hydroxymethylglutaryl-CoA reductase (NADPH)	1.1.1.34	Rat	10084306
hydroxymethylglutaryl-CoA reductase (NADPH)	1.1.1.34	Rat	10204089
hydroxymethylglutaryl-CoA reductase (NADPH)	1.1.1.34	Rat	10377386
hydroxymethylglutaryl-CoA reductase (NADPH)	1.1.1.34	Rat	10892724
hydroxymethylglutaryl-CoA reductase (NADPH)	1.1.1.34	Rat	10964918
hydroxymethylglutaryl-CoA reductase (NADPH)	1.1.1.34	Rat	11043510
hydroxymethylglutaryl-CoA reductase (NADPH)	1.1.1.34	Rat	11516100
hydroxymethylglutaryl-CoA reductase (NADPH)	1.1.1.34	Rat	11881568
hydroxymethylglutaryl-CoA reductase (NADPH)	1.1.1.34	Rat	12405293

hydroxymethylglutaryl-CoA reductase (NADPH)	1.1.1.34	Rat	12467639
hydroxymethylglutaryl-CoA reductase (NADPH)	1.1.1.34	Rat	12736772
hydroxymethylglutaryl-CoA reductase (NADPH)	1.1.1.34	Rat	14523992
hydroxymethylglutaryl-CoA reductase (NADPH)	1.1.1.34	Rat	14720509
hydroxymethylglutaryl-CoA reductase (NADPH)	1.1.1.34	Rat	15034683
hydroxymethylglutaryl-CoA reductase (NADPH)	1.1.1.34	Rat	15248477
hydroxymethylglutaryl-CoA reductase (NADPH)	1.1.1.34	Rat	15605175
hydroxymethylglutaryl-CoA reductase (NADPH)	1.1.1.34	Rat	16101500
hydroxymethylglutaryl-CoA reductase (NADPH)	1.1.1.34	Rat	16168377
hydroxymethylglutaryl-CoA reductase (NADPH)	1.1.1.34	Rat	1629633
hydroxymethylglutaryl-CoA reductase (NADPH)	1.1.1.34	Rat	1652430
hydroxymethylglutaryl-CoA reductase (NADPH)	1.1.1.34	Rat	16611135
hydroxymethylglutaryl-CoA reductase (NADPH)	1.1.1.34	Rat	187533
hydroxymethylglutaryl-CoA reductase (NADPH)	1.1.1.34	Rat	1954650
hydroxymethylglutaryl-CoA reductase (NADPH)	1.1.1.34	Rat	216867
hydroxymethylglutaryl-CoA reductase (NADPH)	1.1.1.34	Rat	2719596
hydroxymethylglutaryl-CoA reductase (NADPH)	1.1.1.34	Rat	278983
hydroxymethylglutaryl-CoA reductase (NADPH)	1.1.1.34	Rat	2921640
hydroxymethylglutaryl-CoA reductase (NADPH)	1.1.1.34	Rat	2995161
hydroxymethylglutaryl-CoA reductase (NADPH)	1.1.1.34	Rat	3055919
hydroxymethylglutaryl-CoA reductase (NADPH)	1.1.1.34	Rat	3131638

hydroxymethylglutaryl-CoA reductase (NADPH)	1.1.1.34	Rat	3308873
hydroxymethylglutaryl-CoA reductase (NADPH)	1.1.1.34	Rat	3314447
hydroxymethylglutaryl-CoA reductase (NADPH)	1.1.1.34	Rat	3689494
hydroxymethylglutaryl-CoA reductase (NADPH)	1.1.1.34	Rat	3968683
hydroxymethylglutaryl-CoA reductase (NADPH)	1.1.1.34	Rat	4075700
hydroxymethylglutaryl-CoA reductase (NADPH)	1.1.1.34	Rat	6088070
hydroxymethylglutaryl-CoA reductase (NADPH)	1.1.1.34	Rat	6256737
hydroxymethylglutaryl-CoA reductase (NADPH)	1.1.1.34	Rat	6274615
hydroxymethylglutaryl-CoA reductase (NADPH)	1.1.1.34	Rat	6286363
hydroxymethylglutaryl-CoA reductase (NADPH)	1.1.1.34	Rat	6347025
hydroxymethylglutaryl-CoA reductase (NADPH)	1.1.1.34	Rat	6396116
hydroxymethylglutaryl-CoA reductase (NADPH)	1.1.1.34	Rat	6594693
hydroxymethylglutaryl-CoA reductase (NADPH)	1.1.1.34	Rat	666819
hydroxymethylglutaryl-CoA reductase (NADPH)	1.1.1.34	Rat	6685129
hydroxymethylglutaryl-CoA reductase (NADPH)	1.1.1.34	Rat	6698994
hydroxymethylglutaryl-CoA reductase (NADPH)	1.1.1.34	Rat	8054400
hydroxymethylglutaryl-CoA reductase (NADPH)	1.1.1.34	Rat	8182149
hydroxymethylglutaryl-CoA reductase (NADPH)	1.1.1.34	Rat	8393338
hydroxymethylglutaryl-CoA reductase (NADPH)	1.1.1.34	Rat	8504036
hydroxymethylglutaryl-CoA reductase (NADPH)	1.1.1.34	Rat	8772195
hydroxymethylglutaryl-CoA reductase (NADPH)	1.1.1.34	Rat	9151797

hydroxymethylglutaryl-CoA reductase (NADPH)	1.1.1.34	Rat	9185766
hydroxymethylglutaryl-CoA reductase (NADPH)	1.1.1.34	Rat	9372476
hydroxymethylglutaryl-CoA reductase (NADPH)	1.1.1.34	Rat	9558731
hydroxymethylglutaryl-CoA reductase (NADPH)	1.1.1.34	Rat	9802623
3-hydroxyacyl-CoA dehydrogenase	1.1.1.35	Rat	15358356
3-hydroxyacyl-CoA dehydrogenase	1.1.1.35	Rat	1637289
3-hydroxyacyl-CoA dehydrogenase	1.1.1.35	Rat	2388659
3-hydroxyacyl-CoA dehydrogenase	1.1.1.35	Rat	6588129
malate dehydrogenase	1.1.1.37	Rat	11583380
malate dehydrogenase	1.1.1.37	Rat	11767008
malate dehydrogenase	1.1.1.37	Rat	11855723
malate dehydrogenase	1.1.1.37	Rat	131232
malate dehydrogenase	1.1.1.37	Rat	16028114
malate dehydrogenase	1.1.1.37	Rat	16212411
malate dehydrogenase	1.1.1.37	Rat	16661455
malate dehydrogenase	1.1.1.37	Rat	3995045
malate dehydrogenase	1.1.1.37	Rat	4053567
malate dehydrogenase	1.1.1.37	Rat	7138874
malate dehydrogenase	1.1.1.37	Rat	8624506
malate dehydrogenase	1.1.1.37	Rat	9348107
isocitrate dehydrogenase (NAD+)	1.1.1.41	Rat	10461937
isocitrate dehydrogenase (NAD+)	1.1.1.41	Rat	12033940
isocitrate dehydrogenase (NAD+)	1.1.1.41	Rat	12619682
isocitrate dehydrogenase (NAD+)	1.1.1.41	Rat	14555658
isocitrate dehydrogenase (NAD+)	1.1.1.41	Rat	15173171
isocitrate dehydrogenase (NAD+)	1.1.1.41	Rat	15314217
isocitrate dehydrogenase (NAD+)	1.1.1.41	Rat	16415587
isocitrate dehydrogenase (NAD+)	1.1.1.41	Rat	6389540
isocitrate dehydrogenase (NAD+)	1.1.1.41	Rat	7710326
isocitrate dehydrogenase (NAD+)	1.1.1.41	Rat	8626605
isocitrate dehydrogenase (NAD+)	1.1.1.41	Rat	9733544
isocitrate dehydrogenase (NAD+)	1.1.1.41	Rat	9881153
glucose-6-phosphate dehydrogenase	1.1.1.49	Rat	10098886
glucose-6-phosphate dehydrogenase	1.1.1.49	Rat	10099785
glucose-6-phosphate dehydrogenase	1.1.1.49	Rat	10329961
glucose-6-phosphate dehydrogenase	1.1.1.49	Rat	10825753
glucose-6-phosphate dehydrogenase	1.1.1.49	Rat	10998184
glucose-6-phosphate dehydrogenase	1.1.1.49	Rat	11023706
glucose-6-phosphate dehydrogenase	1.1.1.49	Rat	11245448

glucose-6-phosphate dehydrogenase	1.1.1.49	Rat	11463792
glucose-6-phosphate dehydrogenase	1.1.1.49	Rat	11520909
glucose-6-phosphate dehydrogenase	1.1.1.49	Rat	12027950
glucose-6-phosphate dehydrogenase	1.1.1.49	Rat	12204336
glucose-6-phosphate dehydrogenase	1.1.1.49	Rat	12393032
glucose-6-phosphate dehydrogenase	1.1.1.49	Rat	12414804
glucose-6-phosphate dehydrogenase	1.1.1.49	Rat	12453665
glucose-6-phosphate dehydrogenase	1.1.1.49	Rat	12472120
glucose-6-phosphate dehydrogenase	1.1.1.49	Rat	12502759
glucose-6-phosphate dehydrogenase	1.1.1.49	Rat	131232
glucose-6-phosphate dehydrogenase	1.1.1.49	Rat	1384463
glucose-6-phosphate dehydrogenase	1.1.1.49	Rat	1417703
glucose-6-phosphate dehydrogenase	1.1.1.49	Rat	147929
glucose-6-phosphate dehydrogenase	1.1.1.49	Rat	15331344
glucose-6-phosphate dehydrogenase	1.1.1.49	Rat	15345489
glucose-6-phosphate dehydrogenase	1.1.1.49	Rat	15527069
glucose-6-phosphate dehydrogenase	1.1.1.49	Rat	15550513
glucose-6-phosphate dehydrogenase	1.1.1.49	Rat	15634201
glucose-6-phosphate dehydrogenase	1.1.1.49	Rat	15739803
glucose-6-phosphate dehydrogenase	1.1.1.49	Rat	15760711
glucose-6-phosphate dehydrogenase	1.1.1.49	Rat	15858258
glucose-6-phosphate dehydrogenase	1.1.1.49	Rat	15975496
glucose-6-phosphate dehydrogenase	1.1.1.49	Rat	16039947
glucose-6-phosphate dehydrogenase	1.1.1.49	Rat	16439706
glucose-6-phosphate dehydrogenase	1.1.1.49	Rat	16849632
glucose-6-phosphate dehydrogenase	1.1.1.49	Rat	17157446
glucose-6-phosphate dehydrogenase	1.1.1.49	Rat	1830744
glucose-6-phosphate dehydrogenase	1.1.1.49	Rat	1922658
glucose-6-phosphate dehydrogenase	1.1.1.49	Rat	1978808
glucose-6-phosphate dehydrogenase	1.1.1.49	Rat	2208076
glucose-6-phosphate dehydrogenase	1.1.1.49	Rat	2296762
glucose-6-phosphate dehydrogenase	1.1.1.49	Rat	2767006
glucose-6-phosphate dehydrogenase	1.1.1.49	Rat	2808772
glucose-6-phosphate dehydrogenase	1.1.1.49	Rat	2846196
glucose-6-phosphate dehydrogenase	1.1.1.49	Rat	2984461
glucose-6-phosphate dehydrogenase	1.1.1.49	Rat	3116361
glucose-6-phosphate dehydrogenase	1.1.1.49	Rat	3161339
glucose-6-phosphate dehydrogenase	1.1.1.49	Rat	3316204
glucose-6-phosphate dehydrogenase	1.1.1.49	Rat	3337882
glucose-6-phosphate dehydrogenase	1.1.1.49	Rat	3365274
glucose-6-phosphate dehydrogenase	1.1.1.49	Rat	3532684
glucose-6-phosphate dehydrogenase	1.1.1.49	Rat	3621197
glucose-6-phosphate dehydrogenase	1.1.1.49	Rat	3717951

glucose-6-phosphate dehydrogenase	1.1.1.49	Rat	3765490
glucose-6-phosphate dehydrogenase	1.1.1.49	Rat	3949801
glucose-6-phosphate dehydrogenase	1.1.1.49	Rat	4288679
glucose-6-phosphate dehydrogenase	1.1.1.49	Rat	4382249
glucose-6-phosphate dehydrogenase	1.1.1.49	Rat	4400642
glucose-6-phosphate dehydrogenase	1.1.1.49	Rat	4941552
glucose-6-phosphate dehydrogenase	1.1.1.49	Rat	6295653
glucose-6-phosphate dehydrogenase	1.1.1.49	Rat	6341787
glucose-6-phosphate dehydrogenase	1.1.1.49	Rat	6363888
glucose-6-phosphate dehydrogenase	1.1.1.49	Rat	6420889
glucose-6-phosphate dehydrogenase	1.1.1.49	Rat	645360
glucose-6-phosphate dehydrogenase	1.1.1.49	Rat	6591771
glucose-6-phosphate dehydrogenase	1.1.1.49	Rat	6696439
glucose-6-phosphate dehydrogenase	1.1.1.49	Rat	7126822
glucose-6-phosphate dehydrogenase	1.1.1.49	Rat	7578910
glucose-6-phosphate dehydrogenase	1.1.1.49	Rat	7681896
glucose-6-phosphate dehydrogenase	1.1.1.49	Rat	7768207
glucose-6-phosphate dehydrogenase	1.1.1.49	Rat	7930940
glucose-6-phosphate dehydrogenase	1.1.1.49	Rat	8316633
glucose-6-phosphate dehydrogenase	1.1.1.49	Rat	864
glucose-6-phosphate dehydrogenase	1.1.1.49	Rat	8760336
glucose-6-phosphate dehydrogenase	1.1.1.49	Rat	8797095
glucose-6-phosphate dehydrogenase	1.1.1.49	Rat	8857518
glucose-6-phosphate dehydrogenase	1.1.1.49	Rat	8910528
glucose-6-phosphate dehydrogenase	1.1.1.49	Rat	8954569
glucose-6-phosphate dehydrogenase	1.1.1.49	Rat	9042391
glucose-6-phosphate dehydrogenase	1.1.1.49	Rat	9553122
glucose-6-phosphate dehydrogenase	1.1.1.49	Rat	9581796
glucose-6-phosphate dehydrogenase	1.1.1.49	Rat	9915806
L-gulonolactone oxidase	1.1.3.8	Rat	15674730
L-gulonolactone oxidase	1.1.3.8	Rat	16177205
L-gulonolactone oxidase	1.1.3.8	Rat	16632110
L-gulonolactone oxidase	1.1.3.8	Rat	1889832
L-gulonolactone oxidase	1.1.3.8	Rat	1962571
L-gulonolactone oxidase	1.1.3.8	Rat	369458
choline dehydrogenase	1.1.99.1	Rat	8868068
ubiquinol---cytochrome-c reductase	1.10.2.2	Rat	16005845
ubiquinol---cytochrome-c reductase	1.10.2.2	Rat	17223530
tryptophan 2,3-dioxygenase	1.13.11.11	Rat	10966936
tryptophan 2,3-dioxygenase	1.13.11.11	Rat	17761498
tryptophan 2,3-dioxygenase	1.13.11.11	Rat	2215078
tryptophan 2,3-dioxygenase	1.13.11.11	Rat	4293961
tryptophan 2,3-dioxygenase	1.13.11.11	Rat	8806758

tryptophan 2,3-dioxygenase	1.13.11.11	Rat	9291104
4-hydroxyphenylpyruvate dioxygenase	1.13.11.27	Rat	10098661
4-hydroxyphenylpyruvate dioxygenase	1.13.11.27	Rat	12014960
4-hydroxyphenylpyruvate dioxygenase	1.13.11.27	Rat	12127941
4-hydroxyphenylpyruvate dioxygenase	1.13.11.27	Rat	240411
4-hydroxyphenylpyruvate dioxygenase	1.13.11.27	Rat	9701587
arachidonate 5-lipoxygenase	1.13.11.34	Rat	10337029
arachidonate 5-lipoxygenase	1.13.11.34	Rat	10378985
arachidonate 5-lipoxygenase	1.13.11.34	Rat	10978197
arachidonate 5-lipoxygenase	1.13.11.34	Rat	10984486
arachidonate 5-lipoxygenase	1.13.11.34	Rat	11369634
arachidonate 5-lipoxygenase	1.13.11.34	Rat	11394934
arachidonate 5-lipoxygenase	1.13.11.34	Rat	11488449
arachidonate 5-lipoxygenase	1.13.11.34	Rat	11542380
arachidonate 5-lipoxygenase	1.13.11.34	Rat	11706027
arachidonate 5-lipoxygenase	1.13.11.34	Rat	11730938
arachidonate 5-lipoxygenase	1.13.11.34	Rat	11844797
arachidonate 5-lipoxygenase	1.13.11.34	Rat	12142344
arachidonate 5-lipoxygenase	1.13.11.34	Rat	12205041
arachidonate 5-lipoxygenase	1.13.11.34	Rat	1311589
arachidonate 5-lipoxygenase	1.13.11.34	Rat	1357659
arachidonate 5-lipoxygenase	1.13.11.34	Rat	15120715
arachidonate 5-lipoxygenase	1.13.11.34	Rat	15265316
arachidonate 5-lipoxygenase	1.13.11.34	Rat	15640495
arachidonate 5-lipoxygenase	1.13.11.34	Rat	15852496
arachidonate 5-lipoxygenase	1.13.11.34	Rat	15947420
arachidonate 5-lipoxygenase	1.13.11.34	Rat	16165096
arachidonate 5-lipoxygenase	1.13.11.34	Rat	16216483
arachidonate 5-lipoxygenase	1.13.11.34	Rat	16223251
arachidonate 5-lipoxygenase	1.13.11.34	Rat	16566819
arachidonate 5-lipoxygenase	1.13.11.34	Rat	16787324
arachidonate 5-lipoxygenase	1.13.11.34	Rat	16821792
arachidonate 5-lipoxygenase	1.13.11.34	Rat	16868922
arachidonate 5-lipoxygenase	1.13.11.34	Rat	17376394
arachidonate 5-lipoxygenase	1.13.11.34	Rat	1903222
arachidonate 5-lipoxygenase	1.13.11.34	Rat	1910248
arachidonate 5-lipoxygenase	1.13.11.34	Rat	2649885
arachidonate 5-lipoxygenase	1.13.11.34	Rat	3417684
arachidonate 5-lipoxygenase	1.13.11.34	Rat	6135325
arachidonate 5-lipoxygenase	1.13.11.34	Rat	6312489
arachidonate 5-lipoxygenase	1.13.11.34	Rat	7768385
arachidonate 5-lipoxygenase	1.13.11.34	Rat	7780156
arachidonate 5-lipoxygenase	1.13.11.34	Rat	8140120

arachidonate 5-lipoxygenase	1.13.11.34	Rat	8245474
arachidonate 5-lipoxygenase	1.13.11.34	Rat	8283055
arachidonate 5-lipoxygenase	1.13.11.34	Rat	8364900
arachidonate 5-lipoxygenase	1.13.11.34	Rat	8567845
arachidonate 5-lipoxygenase	1.13.11.34	Rat	9049593
arachidonate 5-lipoxygenase	1.13.11.34	Rat	9133649
arachidonate 5-lipoxygenase	1.13.11.34	Rat	9411661
arachidonate 5-lipoxygenase	1.13.11.34	Rat	9491904
arachidonate 5-lipoxygenase	1.13.11.34	Rat	9873115
indoleamine 2,3-dioxygenase	1.13.11.52	Rat	10721098
indoleamine 2,3-dioxygenase	1.13.11.52	Rat	10731095
indoleamine 2,3-dioxygenase	1.13.11.52	Rat	10833386
indoleamine 2,3-dioxygenase	1.13.11.52	Rat	10926204
indoleamine 2,3-dioxygenase	1.13.11.52	Rat	10939283
indoleamine 2,3-dioxygenase	1.13.11.52	Rat	10939284
indoleamine 2,3-dioxygenase	1.13.11.52	Rat	10957719
indoleamine 2,3-dioxygenase	1.13.11.52	Rat	11180976
indoleamine 2,3-dioxygenase	1.13.11.52	Rat	11230514
indoleamine 2,3-dioxygenase	1.13.11.52	Rat	11440641
indoleamine 2,3-dioxygenase	1.13.11.52	Rat	11477543
indoleamine 2,3-dioxygenase	1.13.11.52	Rat	11507170
indoleamine 2,3-dioxygenase	1.13.11.52	Rat	12414962
indoleamine 2,3-dioxygenase	1.13.11.52	Rat	12832720
indoleamine 2,3-dioxygenase	1.13.11.52	Rat	12848846
indoleamine 2,3-dioxygenase	1.13.11.52	Rat	15206741
indoleamine 2,3-dioxygenase	1.13.11.52	Rat	15254594
indoleamine 2,3-dioxygenase	1.13.11.52	Rat	15358362
indoleamine 2,3-dioxygenase	1.13.11.52	Rat	15542091
indoleamine 2,3-dioxygenase	1.13.11.52	Rat	15853924
indoleamine 2,3-dioxygenase	1.13.11.52	Rat	15961516
indoleamine 2,3-dioxygenase	1.13.11.52	Rat	16075385
indoleamine 2,3-dioxygenase	1.13.11.52	Rat	16083346
indoleamine 2,3-dioxygenase	1.13.11.52	Rat	16176799
indoleamine 2,3-dioxygenase	1.13.11.52	Rat	16624246
indoleamine 2,3-dioxygenase	1.13.11.52	Rat	17055065
indoleamine 2,3-dioxygenase	1.13.11.52	Rat	8423409
indoleamine 2,3-dioxygenase	1.13.11.52	Rat	8702590
indoleamine 2,3-dioxygenase	1.13.11.52	Rat	9466588
cholestanetriol 26-monooxygenase	1.14.13.15	Rat	11108738
cholestanetriol 26-monooxygenase	1.14.13.15	Rat	11166758
cholestanetriol 26-monooxygenase	1.14.13.15	Rat	11406622
cholestanetriol 26-monooxygenase	1.14.13.15	Rat	15936349
cholestanetriol 26-monooxygenase	1.14.13.15	Rat	2019602

cholestanetriol 26-monooxygenase	1.14.13.15	Rat	7557873
cholestanetriol 26-monooxygenase	1.14.13.15	Rat	8001744
cholesterol 7alpha-monooxygenase	1.14.13.17	Rat	10334992
cholesterol 7alpha-monooxygenase	1.14.13.17	Rat	10405006
cholesterol 7alpha-monooxygenase	1.14.13.17	Rat	10431389
cholesterol 7alpha-monooxygenase	1.14.13.17	Rat	10588945
cholesterol 7alpha-monooxygenase	1.14.13.17	Rat	10599986
cholesterol 7alpha-monooxygenase	1.14.13.17	Rat	10656290
cholesterol 7alpha-monooxygenase	1.14.13.17	Rat	10731667
cholesterol 7alpha-monooxygenase	1.14.13.17	Rat	10744771
cholesterol 7alpha-monooxygenase	1.14.13.17	Rat	10858029
cholesterol 7alpha-monooxygenase	1.14.13.17	Rat	10900260
cholesterol 7alpha-monooxygenase	1.14.13.17	Rat	10936612
cholesterol 7alpha-monooxygenase	1.14.13.17	Rat	11030331
cholesterol 7alpha-monooxygenase	1.14.13.17	Rat	11075809
cholesterol 7alpha-monooxygenase	1.14.13.17	Rat	11108738
cholesterol 7alpha-monooxygenase	1.14.13.17	Rat	11136553
cholesterol 7alpha-monooxygenase	1.14.13.17	Rat	11254888
cholesterol 7alpha-monooxygenase	1.14.13.17	Rat	11264982
cholesterol 7alpha-monooxygenase	1.14.13.17	Rat	11402042
cholesterol 7alpha-monooxygenase	1.14.13.17	Rat	11406622
cholesterol 7alpha-monooxygenase	1.14.13.17	Rat	11427207
cholesterol 7alpha-monooxygenase	1.14.13.17	Rat	11438503
cholesterol 7alpha-monooxygenase	1.14.13.17	Rat	11557507
cholesterol 7alpha-monooxygenase	1.14.13.17	Rat	11604260
cholesterol 7alpha-monooxygenase	1.14.13.17	Rat	11787596
cholesterol 7alpha-monooxygenase	1.14.13.17	Rat	11967026
cholesterol 7alpha-monooxygenase	1.14.13.17	Rat	12042433
cholesterol 7alpha-monooxygenase	1.14.13.17	Rat	12049994
cholesterol 7alpha-monooxygenase	1.14.13.17	Rat	12393855
cholesterol 7alpha-monooxygenase	1.14.13.17	Rat	1245792
cholesterol 7alpha-monooxygenase	1.14.13.17	Rat	12554795
cholesterol 7alpha-monooxygenase	1.14.13.17	Rat	12562858
cholesterol 7alpha-monooxygenase	1.14.13.17	Rat	12675851
cholesterol 7alpha-monooxygenase	1.14.13.17	Rat	12787409
cholesterol 7alpha-monooxygenase	1.14.13.17	Rat	12805410
cholesterol 7alpha-monooxygenase	1.14.13.17	Rat	12865425
cholesterol 7alpha-monooxygenase	1.14.13.17	Rat	12917427
cholesterol 7alpha-monooxygenase	1.14.13.17	Rat	14660582
cholesterol 7alpha-monooxygenase	1.14.13.17	Rat	14698038
cholesterol 7alpha-monooxygenase	1.14.13.17	Rat	14748721
cholesterol 7alpha-monooxygenase	1.14.13.17	Rat	14762172
cholesterol 7alpha-monooxygenase	1.14.13.17	Rat	14960319

cholesterol 7alpha-monooxygenase	1.14.13.17	Rat	15145977
cholesterol 7alpha-monooxygenase	1.14.13.17	Rat	15241483
cholesterol 7alpha-monooxygenase	1.14.13.17	Rat	15333704
cholesterol 7alpha-monooxygenase	1.14.13.17	Rat	15375335
cholesterol 7alpha-monooxygenase	1.14.13.17	Rat	15458444
cholesterol 7alpha-monooxygenase	1.14.13.17	Rat	15574426
cholesterol 7alpha-monooxygenase	1.14.13.17	Rat	15583480
cholesterol 7alpha-monooxygenase	1.14.13.17	Rat	15649292
cholesterol 7alpha-monooxygenase	1.14.13.17	Rat	15707388
cholesterol 7alpha-monooxygenase	1.14.13.17	Rat	15750181
cholesterol 7alpha-monooxygenase	1.14.13.17	Rat	15752749
cholesterol 7alpha-monooxygenase	1.14.13.17	Rat	15795435
cholesterol 7alpha-monooxygenase	1.14.13.17	Rat	15936349
cholesterol 7alpha-monooxygenase	1.14.13.17	Rat	16075052
cholesterol 7alpha-monooxygenase	1.14.13.17	Rat	16213224
cholesterol 7alpha-monooxygenase	1.14.13.17	Rat	16271991
cholesterol 7alpha-monooxygenase	1.14.13.17	Rat	16464476
cholesterol 7alpha-monooxygenase	1.14.13.17	Rat	17054913
cholesterol 7alpha-monooxygenase	1.14.13.17	Rat	223590
cholesterol 7alpha-monooxygenase	1.14.13.17	Rat	2713876
cholesterol 7alpha-monooxygenase	1.14.13.17	Rat	3768033
cholesterol 7alpha-monooxygenase	1.14.13.17	Rat	3981265
cholesterol 7alpha-monooxygenase	1.14.13.17	Rat	8663429
cholesterol 7alpha-monooxygenase	1.14.13.17	Rat	8759369
cholesterol 7alpha-monooxygenase	1.14.13.17	Rat	8858751
cholesterol 7alpha-monooxygenase	1.14.13.17	Rat	8895609
cholesterol 7alpha-monooxygenase	1.14.13.17	Rat	8943286
cholesterol 7alpha-monooxygenase	1.14.13.17	Rat	9013589
cholesterol 7alpha-monooxygenase	1.14.13.17	Rat	9151797
cholesterol 7alpha-monooxygenase	1.14.13.17	Rat	9185766
cholesterol 7alpha-monooxygenase	1.14.13.17	Rat	9462665
cholesterol 7alpha-monooxygenase	1.14.13.17	Rat	9550534
cholesterol 7alpha-monooxygenase	1.14.13.17	Rat	9558731
cholesterol 7alpha-monooxygenase	1.14.13.17	Rat	9625597
cholesterol 7alpha-monooxygenase	1.14.13.17	Rat	9630215
cholesterol 7alpha-monooxygenase	1.14.13.17	Rat	9649606
cholesterol 7alpha-monooxygenase	1.14.13.17	Rat	9721193
cholesterol 7alpha-monooxygenase	1.14.13.17	Rat	9799805
cholesterol 7alpha-monooxygenase	1.14.13.17	Rat	9881645
nitric-oxide synthase	1.14.13.39	Rat	10630682
nitric-oxide synthase	1.14.13.39	Rat	10821631
nitric-oxide synthase	1.14.13.39	Rat	10868974
nitric-oxide synthase	1.14.13.39	Rat	11095648

nitric-oxide synthase	1.14.13.39	Rat	11292821
nitric-oxide synthase	1.14.13.39	Rat	11328947
nitric-oxide synthase	1.14.13.39	Rat	11468403
nitric-oxide synthase	1.14.13.39	Rat	11556547
nitric-oxide synthase	1.14.13.39	Rat	11852055
nitric-oxide synthase	1.14.13.39	Rat	12176955
nitric-oxide synthase	1.14.13.39	Rat	12297263
nitric-oxide synthase	1.14.13.39	Rat	12354096
nitric-oxide synthase	1.14.13.39	Rat	12402580
nitric-oxide synthase	1.14.13.39	Rat	12503100
nitric-oxide synthase	1.14.13.39	Rat	12566086
nitric-oxide synthase	1.14.13.39	Rat	12591128
nitric-oxide synthase	1.14.13.39	Rat	12810358
nitric-oxide synthase	1.14.13.39	Rat	12830073
nitric-oxide synthase	1.14.13.39	Rat	12855421
nitric-oxide synthase	1.14.13.39	Rat	12869534
nitric-oxide synthase	1.14.13.39	Rat	14662726
nitric-oxide synthase	1.14.13.39	Rat	14668561
nitric-oxide synthase	1.14.13.39	Rat	14769821
nitric-oxide synthase	1.14.13.39	Rat	15099358
nitric-oxide synthase	1.14.13.39	Rat	15167268
nitric-oxide synthase	1.14.13.39	Rat	15223360
nitric-oxide synthase	1.14.13.39	Rat	15448133
nitric-oxide synthase	1.14.13.39	Rat	15588718
nitric-oxide synthase	1.14.13.39	Rat	15698596
nitric-oxide synthase	1.14.13.39	Rat	16179540
nitric-oxide synthase	1.14.13.39	Rat	16249336
nitric-oxide synthase	1.14.13.39	Rat	16297560
nitric-oxide synthase	1.14.13.39	Rat	16315601
nitric-oxide synthase	1.14.13.39	Rat	16464859
nitric-oxide synthase	1.14.13.39	Rat	16641207
nitric-oxide synthase	1.14.13.39	Rat	17003331
nitric-oxide synthase	1.14.13.39	Rat	17293453
nitric-oxide synthase	1.14.13.39	Rat	7689840
nitric-oxide synthase	1.14.13.39	Rat	8694803
nitric-oxide synthase	1.14.13.39	Rat	8791097
nitric-oxide synthase	1.14.13.39	Rat	8945918
nitric-oxide synthase	1.14.13.39	Rat	8958563
nitric-oxide synthase	1.14.13.39	Rat	9247967
nitric-oxide synthase	1.14.13.39	Rat	9336394
nitric-oxide synthase	1.14.13.39	Rat	9409304
nitric-oxide synthase	1.14.13.39	Rat	9525991
nitric-oxide synthase	1.14.13.39	Rat	9654136

nitric-oxide synthase	1.14.13.39	Rat	9665318
nitric-oxide synthase	1.14.13.39	Rat	9822514
nitric-oxide synthase	1.14.13.39	Rat	9856817
kynurenine 3-monooxygenase	1.14.13.9	Rat	42292
kynurenine 3-monooxygenase	1.14.13.9	Rat	7131096
kynurenine 3-monooxygenase	1.14.13.9	Rat	7131097
unspecific monooxygenase	1.14.14.1	Rat	10462973
unspecific monooxygenase	1.14.14.1	Rat	10473018
unspecific monooxygenase	1.14.14.1	Rat	10474272
unspecific monooxygenase	1.14.14.1	Rat	10690899
unspecific monooxygenase	1.14.14.1	Rat	10713305
unspecific monooxygenase	1.14.14.1	Rat	10746939
unspecific monooxygenase	1.14.14.1	Rat	10947336
unspecific monooxygenase	1.14.14.1	Rat	11116206
unspecific monooxygenase	1.14.14.1	Rat	11259506
unspecific monooxygenase	1.14.14.1	Rat	11312650
unspecific monooxygenase	1.14.14.1	Rat	11403896
unspecific monooxygenase	1.14.14.1	Rat	11502834
unspecific monooxygenase	1.14.14.1	Rat	11922772
unspecific monooxygenase	1.14.14.1	Rat	11961225
unspecific monooxygenase	1.14.14.1	Rat	12053085
unspecific monooxygenase	1.14.14.1	Rat	12439219
unspecific monooxygenase	1.14.14.1	Rat	12606587
unspecific monooxygenase	1.14.14.1	Rat	12706301
unspecific monooxygenase	1.14.14.1	Rat	12715371
unspecific monooxygenase	1.14.14.1	Rat	12736278
unspecific monooxygenase	1.14.14.1	Rat	12810639
unspecific monooxygenase	1.14.14.1	Rat	12845227
unspecific monooxygenase	1.14.14.1	Rat	12957661
unspecific monooxygenase	1.14.14.1	Rat	12960093
unspecific monooxygenase	1.14.14.1	Rat	1339246
unspecific monooxygenase	1.14.14.1	Rat	14501171
unspecific monooxygenase	1.14.14.1	Rat	14580722
unspecific monooxygenase	1.14.14.1	Rat	14623534
unspecific monooxygenase	1.14.14.1	Rat	14965567
unspecific monooxygenase	1.14.14.1	Rat	15226009
unspecific monooxygenase	1.14.14.1	Rat	15255840
unspecific monooxygenase	1.14.14.1	Rat	15319488
unspecific monooxygenase	1.14.14.1	Rat	15322103
unspecific monooxygenase	1.14.14.1	Rat	15582747
unspecific monooxygenase	1.14.14.1	Rat	15591029
unspecific monooxygenase	1.14.14.1	Rat	15623590
unspecific monooxygenase	1.14.14.1	Rat	15876405

unspecific monooxygenase	1.14.14.1	Rat	15885269
unspecific monooxygenase	1.14.14.1	Rat	15930283
unspecific monooxygenase	1.14.14.1	Rat	16006326
unspecific monooxygenase	1.14.14.1	Rat	16077170
unspecific monooxygenase	1.14.14.1	Rat	16109788
unspecific monooxygenase	1.14.14.1	Rat	16109840
unspecific monooxygenase	1.14.14.1	Rat	16170371
unspecific monooxygenase	1.14.14.1	Rat	16260617
unspecific monooxygenase	1.14.14.1	Rat	16285913
unspecific monooxygenase	1.14.14.1	Rat	16303757
unspecific monooxygenase	1.14.14.1	Rat	16322267
unspecific monooxygenase	1.14.14.1	Rat	16426763
unspecific monooxygenase	1.14.14.1	Rat	16473000
unspecific monooxygenase	1.14.14.1	Rat	16541462
unspecific monooxygenase	1.14.14.1	Rat	16611627
unspecific monooxygenase	1.14.14.1	Rat	16733710
unspecific monooxygenase	1.14.14.1	Rat	16763069
unspecific monooxygenase	1.14.14.1	Rat	16877675
unspecific monooxygenase	1.14.14.1	Rat	1694074
unspecific monooxygenase	1.14.14.1	Rat	17005180
unspecific monooxygenase	1.14.14.1	Rat	17079138
unspecific monooxygenase	1.14.14.1	Rat	17573783
unspecific monooxygenase	1.14.14.1	Rat	1835644
unspecific monooxygenase	1.14.14.1	Rat	2149503
unspecific monooxygenase	1.14.14.1	Rat	3419162
unspecific monooxygenase	1.14.14.1	Rat	3626552
unspecific monooxygenase	1.14.14.1	Rat	6233132
unspecific monooxygenase	1.14.14.1	Rat	6580512
unspecific monooxygenase	1.14.14.1	Rat	6645505
unspecific monooxygenase	1.14.14.1	Rat	7083186
unspecific monooxygenase	1.14.14.1	Rat	7581491
unspecific monooxygenase	1.14.14.1	Rat	7626472
unspecific monooxygenase	1.14.14.1	Rat	7694590
unspecific monooxygenase	1.14.14.1	Rat	7949200
unspecific monooxygenase	1.14.14.1	Rat	7968362
unspecific monooxygenase	1.14.14.1	Rat	8017854
unspecific monooxygenase	1.14.14.1	Rat	8073072
unspecific monooxygenase	1.14.14.1	Rat	8126145
unspecific monooxygenase	1.14.14.1	Rat	8698750
unspecific monooxygenase	1.14.14.1	Rat	8824461
unspecific monooxygenase	1.14.14.1	Rat	8989259
unspecific monooxygenase	1.14.14.1	Rat	9027406
unspecific monooxygenase	1.14.14.1	Rat	9038782

unspecific monooxygenase	1.14.14.1	Rat	9187104
unspecific monooxygenase	1.14.14.1	Rat	9365209
unspecific monooxygenase	1.14.14.1	Rat	9415804
unspecific monooxygenase	1.14.14.1	Rat	9442349
unspecific monooxygenase	1.14.14.1	Rat	9456245
unspecific monooxygenase	1.14.14.1	Rat	9511180
unspecific monooxygenase	1.14.14.1	Rat	9523724
unspecific monooxygenase	1.14.14.1	Rat	9541188
unspecific monooxygenase	1.14.14.1	Rat	9698080
unspecific monooxygenase	1.14.14.1	Rat	9703907
unspecific monooxygenase	1.14.14.1	Rat	9788750
unspecific monooxygenase	1.14.14.1	Rat	9797023
unspecific monooxygenase	1.14.14.1	Rat	9804911
cholesterol monooxygenase (side-chain-cleaving)	1.14.15.6	Rat	10418987
cholesterol monooxygenase (side-chain-cleaving)	1.14.15.6	Rat	10700725
cholesterol monooxygenase (side-chain-cleaving)	1.14.15.6	Rat	10729197
cholesterol monooxygenase (side-chain-cleaving)	1.14.15.6	Rat	10964798
cholesterol monooxygenase (side-chain-cleaving)	1.14.15.6	Rat	11191081
cholesterol monooxygenase (side-chain-cleaving)	1.14.15.6	Rat	11297612
cholesterol monooxygenase (side-chain-cleaving)	1.14.15.6	Rat	12137805
cholesterol monooxygenase (side-chain-cleaving)	1.14.15.6	Rat	12596229
cholesterol monooxygenase (side-chain-cleaving)	1.14.15.6	Rat	1282463
cholesterol monooxygenase (side-chain-cleaving)	1.14.15.6	Rat	1327721
cholesterol monooxygenase (side-chain-cleaving)	1.14.15.6	Rat	1337141
cholesterol monooxygenase (side-chain-cleaving)	1.14.15.6	Rat	15026180
cholesterol monooxygenase (side-chain-cleaving)	1.14.15.6	Rat	15159300
cholesterol monooxygenase (side-chain-cleaving)	1.14.15.6	Rat	15205373
cholesterol monooxygenase (side-chain-cleaving)	1.14.15.6	Rat	15231695

cholesterol monooxygenase (side-chain-cleaving)	1.14.15.6	Rat	15344917
cholesterol monooxygenase (side-chain-cleaving)	1.14.15.6	Rat	15635147
cholesterol monooxygenase (side-chain-cleaving)	1.14.15.6	Rat	15666823
cholesterol monooxygenase (side-chain-cleaving)	1.14.15.6	Rat	15804366
cholesterol monooxygenase (side-chain-cleaving)	1.14.15.6	Rat	16139613
cholesterol monooxygenase (side-chain-cleaving)	1.14.15.6	Rat	16410306
cholesterol monooxygenase (side-chain-cleaving)	1.14.15.6	Rat	16541462
cholesterol monooxygenase (side-chain-cleaving)	1.14.15.6	Rat	16551645
cholesterol monooxygenase (side-chain-cleaving)	1.14.15.6	Rat	16632873
cholesterol monooxygenase (side-chain-cleaving)	1.14.15.6	Rat	16780839
cholesterol monooxygenase (side-chain-cleaving)	1.14.15.6	Rat	16999944
cholesterol monooxygenase (side-chain-cleaving)	1.14.15.6	Rat	1700277
cholesterol monooxygenase (side-chain-cleaving)	1.14.15.6	Rat	1874173
cholesterol monooxygenase (side-chain-cleaving)	1.14.15.6	Rat	2226329
cholesterol monooxygenase (side-chain-cleaving)	1.14.15.6	Rat	2555382
cholesterol monooxygenase (side-chain-cleaving)	1.14.15.6	Rat	3027455
cholesterol monooxygenase (side-chain-cleaving)	1.14.15.6	Rat	3356301
cholesterol monooxygenase (side-chain-cleaving)	1.14.15.6	Rat	3502608
cholesterol monooxygenase (side-chain-cleaving)	1.14.15.6	Rat	6249251
cholesterol monooxygenase (side-chain-cleaving)	1.14.15.6	Rat	6256693
cholesterol monooxygenase (side-chain-cleaving)	1.14.15.6	Rat	6546877

cholesterol monooxygenase (side-chain-cleaving)	1.14.15.6	Rat	7594421
cholesterol monooxygenase (side-chain-cleaving)	1.14.15.6	Rat	7664658
cholesterol monooxygenase (side-chain-cleaving)	1.14.15.6	Rat	7669257
cholesterol monooxygenase (side-chain-cleaving)	1.14.15.6	Rat	7826890
cholesterol monooxygenase (side-chain-cleaving)	1.14.15.6	Rat	7882898
cholesterol monooxygenase (side-chain-cleaving)	1.14.15.6	Rat	7956928
cholesterol monooxygenase (side-chain-cleaving)	1.14.15.6	Rat	8026494
cholesterol monooxygenase (side-chain-cleaving)	1.14.15.6	Rat	8152434
cholesterol monooxygenase (side-chain-cleaving)	1.14.15.6	Rat	8278356
cholesterol monooxygenase (side-chain-cleaving)	1.14.15.6	Rat	8425475
cholesterol monooxygenase (side-chain-cleaving)	1.14.15.6	Rat	8432024
cholesterol monooxygenase (side-chain-cleaving)	1.14.15.6	Rat	8547188
cholesterol monooxygenase (side-chain-cleaving)	1.14.15.6	Rat	8584034
cholesterol monooxygenase (side-chain-cleaving)	1.14.15.6	Rat	9013761
cholesterol monooxygenase (side-chain-cleaving)	1.14.15.6	Rat	9326645
cholesterol monooxygenase (side-chain-cleaving)	1.14.15.6	Rat	9511181
cholesterol monooxygenase (side-chain-cleaving)	1.14.15.6	Rat	9622308
cholesterol monooxygenase (side-chain-cleaving)	1.14.15.6	Rat	9635133
cholesterol monooxygenase (side-chain-cleaving)	1.14.15.6	Rat	9888540
cholesterol monooxygenase (side-chain-cleaving)	1.14.15.6	Rat	9922097
phenylalanine 4-monooxygenase	1.14.16.1	Rat	10444341
phenylalanine 4-monooxygenase	1.14.16.1	Rat	10800950
phenylalanine 4-monooxygenase	1.14.16.1	Rat	10900078

phenylalanine 4-monooxygenase	1.14.16.1	Rat	10984661
phenylalanine 4-monooxygenase	1.14.16.1	Rat	15493924
phenylalanine 4-monooxygenase	1.14.16.1	Rat	16139311
phenylalanine 4-monooxygenase	1.14.16.1	Rat	16402341
phenylalanine 4-monooxygenase	1.14.16.1	Rat	16429477
phenylalanine 4-monooxygenase	1.14.16.1	Rat	3768311
phenylalanine 4-monooxygenase	1.14.16.1	Rat	7635153
phenylalanine 4-monooxygenase	1.14.16.1	Rat	7769265
phenylalanine 4-monooxygenase	1.14.16.1	Rat	8214611
phenylalanine 4-monooxygenase	1.14.16.1	Rat	8246172
phenylalanine 4-monooxygenase	1.14.16.1	Rat	8502995
phenylalanine 4-monooxygenase	1.14.16.1	Rat	8927234
phenylalanine 4-monooxygenase	1.14.16.1	Rat	9168638
tyrosine 3-monooxygenase	1.14.16.2	Rat	10320089
tyrosine 3-monooxygenase	1.14.16.2	Rat	10476677
tyrosine 3-monooxygenase	1.14.16.2	Rat	10725922
tyrosine 3-monooxygenase	1.14.16.2	Rat	10970027
tyrosine 3-monooxygenase	1.14.16.2	Rat	11424954
tyrosine 3-monooxygenase	1.14.16.2	Rat	11517172
tyrosine 3-monooxygenase	1.14.16.2	Rat	11948424
tyrosine 3-monooxygenase	1.14.16.2	Rat	12124430
tyrosine 3-monooxygenase	1.14.16.2	Rat	12640623
tyrosine 3-monooxygenase	1.14.16.2	Rat	12717737
tyrosine 3-monooxygenase	1.14.16.2	Rat	12891655
tyrosine 3-monooxygenase	1.14.16.2	Rat	1383560
tyrosine 3-monooxygenase	1.14.16.2	Rat	14675149
tyrosine 3-monooxygenase	1.14.16.2	Rat	15447670
tyrosine 3-monooxygenase	1.14.16.2	Rat	15649149
tyrosine 3-monooxygenase	1.14.16.2	Rat	15683467
tyrosine 3-monooxygenase	1.14.16.2	Rat	15897221
tyrosine 3-monooxygenase	1.14.16.2	Rat	15935064
tyrosine 3-monooxygenase	1.14.16.2	Rat	16049992
tyrosine 3-monooxygenase	1.14.16.2	Rat	16052322
tyrosine 3-monooxygenase	1.14.16.2	Rat	16080996
tyrosine 3-monooxygenase	1.14.16.2	Rat	16187166
tyrosine 3-monooxygenase	1.14.16.2	Rat	16199893
tyrosine 3-monooxygenase	1.14.16.2	Rat	16650497
tyrosine 3-monooxygenase	1.14.16.2	Rat	1847187
tyrosine 3-monooxygenase	1.14.16.2	Rat	1972039
tyrosine 3-monooxygenase	1.14.16.2	Rat	2573869
tyrosine 3-monooxygenase	1.14.16.2	Rat	2883182
tyrosine 3-monooxygenase	1.14.16.2	Rat	2902543
tyrosine 3-monooxygenase	1.14.16.2	Rat	3267347

tyrosine 3-monooxygenase	1.14.16.2	Rat	6150485
tyrosine 3-monooxygenase	1.14.16.2	Rat	7494448
tyrosine 3-monooxygenase	1.14.16.2	Rat	7576974
tyrosine 3-monooxygenase	1.14.16.2	Rat	7719703
tyrosine 3-monooxygenase	1.14.16.2	Rat	8723206
tyrosine 3-monooxygenase	1.14.16.2	Rat	8984738
tyrosine 3-monooxygenase	1.14.16.2	Rat	9247090
tyrosine 3-monooxygenase	1.14.16.2	Rat	9350044
tyrosine 3-monooxygenase	1.14.16.2	Rat	9719456
tyrosine 3-monooxygenase	1.14.16.2	Rat	9822156
tyrosine 3-monooxygenase	1.14.16.2	Rat	9914719
tryptophan 5-monooxygenase	1.14.16.4	Rat	10327914
tryptophan 5-monooxygenase	1.14.16.4	Rat	10483053
tryptophan 5-monooxygenase	1.14.16.4	Rat	10514446
tryptophan 5-monooxygenase	1.14.16.4	Rat	10581400
tryptophan 5-monooxygenase	1.14.16.4	Rat	1059145
tryptophan 5-monooxygenase	1.14.16.4	Rat	10636468
tryptophan 5-monooxygenase	1.14.16.4	Rat	10676966
tryptophan 5-monooxygenase	1.14.16.4	Rat	10715363
tryptophan 5-monooxygenase	1.14.16.4	Rat	10721034
tryptophan 5-monooxygenase	1.14.16.4	Rat	10800950
tryptophan 5-monooxygenase	1.14.16.4	Rat	10899755
tryptophan 5-monooxygenase	1.14.16.4	Rat	10900076
tryptophan 5-monooxygenase	1.14.16.4	Rat	10900078
tryptophan 5-monooxygenase	1.14.16.4	Rat	10909123
tryptophan 5-monooxygenase	1.14.16.4	Rat	10950846
tryptophan 5-monooxygenase	1.14.16.4	Rat	10953292
tryptophan 5-monooxygenase	1.14.16.4	Rat	10993738
tryptophan 5-monooxygenase	1.14.16.4	Rat	11085312
tryptophan 5-monooxygenase	1.14.16.4	Rat	11113315
tryptophan 5-monooxygenase	1.14.16.4	Rat	11121198
tryptophan 5-monooxygenase	1.14.16.4	Rat	11326294
tryptophan 5-monooxygenase	1.14.16.4	Rat	11386854
tryptophan 5-monooxygenase	1.14.16.4	Rat	11426508
tryptophan 5-monooxygenase	1.14.16.4	Rat	11747434
tryptophan 5-monooxygenase	1.14.16.4	Rat	12015221
tryptophan 5-monooxygenase	1.14.16.4	Rat	12116193
tryptophan 5-monooxygenase	1.14.16.4	Rat	12399958
tryptophan 5-monooxygenase	1.14.16.4	Rat	12507391
tryptophan 5-monooxygenase	1.14.16.4	Rat	12818363
tryptophan 5-monooxygenase	1.14.16.4	Rat	12915291
tryptophan 5-monooxygenase	1.14.16.4	Rat	14563478
tryptophan 5-monooxygenase	1.14.16.4	Rat	14960297

tryptophan 5-monooxygenase	1.14.16.4	Rat	15124006
tryptophan 5-monooxygenase	1.14.16.4	Rat	15163437
tryptophan 5-monooxygenase	1.14.16.4	Rat	15281067
tryptophan 5-monooxygenase	1.14.16.4	Rat	15476687
tryptophan 5-monooxygenase	1.14.16.4	Rat	15663479
tryptophan 5-monooxygenase	1.14.16.4	Rat	15677682
tryptophan 5-monooxygenase	1.14.16.4	Rat	15804496
tryptophan 5-monooxygenase	1.14.16.4	Rat	15940290
tryptophan 5-monooxygenase	1.14.16.4	Rat	15968084
tryptophan 5-monooxygenase	1.14.16.4	Rat	16023677
tryptophan 5-monooxygenase	1.14.16.4	Rat	16109589
tryptophan 5-monooxygenase	1.14.16.4	Rat	16165107
tryptophan 5-monooxygenase	1.14.16.4	Rat	16192985
tryptophan 5-monooxygenase	1.14.16.4	Rat	16198203
tryptophan 5-monooxygenase	1.14.16.4	Rat	16203956
tryptophan 5-monooxygenase	1.14.16.4	Rat	16314762
tryptophan 5-monooxygenase	1.14.16.4	Rat	16381305
tryptophan 5-monooxygenase	1.14.16.4	Rat	16405505
tryptophan 5-monooxygenase	1.14.16.4	Rat	16407550
tryptophan 5-monooxygenase	1.14.16.4	Rat	16436194
tryptophan 5-monooxygenase	1.14.16.4	Rat	16467214
tryptophan 5-monooxygenase	1.14.16.4	Rat	16495936
tryptophan 5-monooxygenase	1.14.16.4	Rat	16498236
tryptophan 5-monooxygenase	1.14.16.4	Rat	16581041
tryptophan 5-monooxygenase	1.14.16.4	Rat	16615082
tryptophan 5-monooxygenase	1.14.16.4	Rat	16806098
tryptophan 5-monooxygenase	1.14.16.4	Rat	16864580
tryptophan 5-monooxygenase	1.14.16.4	Rat	16924415
tryptophan 5-monooxygenase	1.14.16.4	Rat	16979275
tryptophan 5-monooxygenase	1.14.16.4	Rat	17015812
tryptophan 5-monooxygenase	1.14.16.4	Rat	1707735
tryptophan 5-monooxygenase	1.14.16.4	Rat	1997008
tryptophan 5-monooxygenase	1.14.16.4	Rat	2466525
tryptophan 5-monooxygenase	1.14.16.4	Rat	2568404
tryptophan 5-monooxygenase	1.14.16.4	Rat	2737466
tryptophan 5-monooxygenase	1.14.16.4	Rat	3379411
tryptophan 5-monooxygenase	1.14.16.4	Rat	3742199
tryptophan 5-monooxygenase	1.14.16.4	Rat	3996672
tryptophan 5-monooxygenase	1.14.16.4	Rat	7552299
tryptophan 5-monooxygenase	1.14.16.4	Rat	761167
tryptophan 5-monooxygenase	1.14.16.4	Rat	8015380
tryptophan 5-monooxygenase	1.14.16.4	Rat	8255926
tryptophan 5-monooxygenase	1.14.16.4	Rat	8527368

tryptophan 5-monooxygenase	1.14.16.4	Rat	8726569
tryptophan 5-monooxygenase	1.14.16.4	Rat	8752114
tryptophan 5-monooxygenase	1.14.16.4	Rat	9084419
tryptophan 5-monooxygenase	1.14.16.4	Rat	9109552
tryptophan 5-monooxygenase	1.14.16.4	Rat	9244768
tryptophan 5-monooxygenase	1.14.16.4	Rat	9295371
tryptophan 5-monooxygenase	1.14.16.4	Rat	9326303
tryptophan 5-monooxygenase	1.14.16.4	Rat	9392522
tryptophan 5-monooxygenase	1.14.16.4	Rat	9603613
tryptophan 5-monooxygenase	1.14.16.4	Rat	9630672
tryptophan 5-monooxygenase	1.14.16.4	Rat	9672049
tryptophan 5-monooxygenase	1.14.16.4	Rat	9770640
tryptophan 5-monooxygenase	1.14.16.4	Rat	9886051
monophenol monooxygenase	1.14.18.1	Rat	10102625
monophenol monooxygenase	1.14.18.1	Rat	10495428
monophenol monooxygenase	1.14.18.1	Rat	10671066
monophenol monooxygenase	1.14.18.1	Rat	10691963
monophenol monooxygenase	1.14.18.1	Rat	10839460
monophenol monooxygenase	1.14.18.1	Rat	10870514
monophenol monooxygenase	1.14.18.1	Rat	10956040
monophenol monooxygenase	1.14.18.1	Rat	10960773
monophenol monooxygenase	1.14.18.1	Rat	10966567
monophenol monooxygenase	1.14.18.1	Rat	10998146
monophenol monooxygenase	1.14.18.1	Rat	11076759
monophenol monooxygenase	1.14.18.1	Rat	11139343
monophenol monooxygenase	1.14.18.1	Rat	11167218
monophenol monooxygenase	1.14.18.1	Rat	11171088
monophenol monooxygenase	1.14.18.1	Rat	11178974
monophenol monooxygenase	1.14.18.1	Rat	11180971
monophenol monooxygenase	1.14.18.1	Rat	11236829
monophenol monooxygenase	1.14.18.1	Rat	11277411
monophenol monooxygenase	1.14.18.1	Rat	11493672
monophenol monooxygenase	1.14.18.1	Rat	11574907
monophenol monooxygenase	1.14.18.1	Rat	11601654
monophenol monooxygenase	1.14.18.1	Rat	11602344
monophenol monooxygenase	1.14.18.1	Rat	11673476
monophenol monooxygenase	1.14.18.1	Rat	11701975
monophenol monooxygenase	1.14.18.1	Rat	11799132
monophenol monooxygenase	1.14.18.1	Rat	11821691
monophenol monooxygenase	1.14.18.1	Rat	11834083
monophenol monooxygenase	1.14.18.1	Rat	11851885
monophenol monooxygenase	1.14.18.1	Rat	11858948
monophenol monooxygenase	1.14.18.1	Rat	12069489

monophenol monooxygenase	1.14.18.1	Rat	12201672
monophenol monooxygenase	1.14.18.1	Rat	12565907
monophenol monooxygenase	1.14.18.1	Rat	12595535
monophenol monooxygenase	1.14.18.1	Rat	12950726
monophenol monooxygenase	1.14.18.1	Rat	14511124
monophenol monooxygenase	1.14.18.1	Rat	14597395
monophenol monooxygenase	1.14.18.1	Rat	14615486
monophenol monooxygenase	1.14.18.1	Rat	14622170
monophenol monooxygenase	1.14.18.1	Rat	14699163
monophenol monooxygenase	1.14.18.1	Rat	14726002
monophenol monooxygenase	1.14.18.1	Rat	15016305
monophenol monooxygenase	1.14.18.1	Rat	15067002
monophenol monooxygenase	1.14.18.1	Rat	15089040
monophenol monooxygenase	1.14.18.1	Rat	15147727
monophenol monooxygenase	1.14.18.1	Rat	15154289
monophenol monooxygenase	1.14.18.1	Rat	15250942
monophenol monooxygenase	1.14.18.1	Rat	15252133
monophenol monooxygenase	1.14.18.1	Rat	15381243
monophenol monooxygenase	1.14.18.1	Rat	15520878
monophenol monooxygenase	1.14.18.1	Rat	15572362
monophenol monooxygenase	1.14.18.1	Rat	15684492
monophenol monooxygenase	1.14.18.1	Rat	15748887
monophenol monooxygenase	1.14.18.1	Rat	15760340
monophenol monooxygenase	1.14.18.1	Rat	15760341
monophenol monooxygenase	1.14.18.1	Rat	15895362
monophenol monooxygenase	1.14.18.1	Rat	16146766
monophenol monooxygenase	1.14.18.1	Rat	16280010
monophenol monooxygenase	1.14.18.1	Rat	16293777
monophenol monooxygenase	1.14.18.1	Rat	16327152
monophenol monooxygenase	1.14.18.1	Rat	16335789
monophenol monooxygenase	1.14.18.1	Rat	16456529
monophenol monooxygenase	1.14.18.1	Rat	16477373
monophenol monooxygenase	1.14.18.1	Rat	16524430
monophenol monooxygenase	1.14.18.1	Rat	16579986
monophenol monooxygenase	1.14.18.1	Rat	16691509
monophenol monooxygenase	1.14.18.1	Rat	16704452
monophenol monooxygenase	1.14.18.1	Rat	1671405
monophenol monooxygenase	1.14.18.1	Rat	16757562
monophenol monooxygenase	1.14.18.1	Rat	16847266
monophenol monooxygenase	1.14.18.1	Rat	17083330
monophenol monooxygenase	1.14.18.1	Rat	17083484
monophenol monooxygenase	1.14.18.1	Rat	1720016
monophenol monooxygenase	1.14.18.1	Rat	1739407

monophenol monooxygenase	1.14.18.1	Rat	1902569
monophenol monooxygenase	1.14.18.1	Rat	1903356
monophenol monooxygenase	1.14.18.1	Rat	2118105
monophenol monooxygenase	1.14.18.1	Rat	2497447
monophenol monooxygenase	1.14.18.1	Rat	2497448
monophenol monooxygenase	1.14.18.1	Rat	2498458
monophenol monooxygenase	1.14.18.1	Rat	2543086
monophenol monooxygenase	1.14.18.1	Rat	2720797
monophenol monooxygenase	1.14.18.1	Rat	2983883
monophenol monooxygenase	1.14.18.1	Rat	2992767
monophenol monooxygenase	1.14.18.1	Rat	3121644
monophenol monooxygenase	1.14.18.1	Rat	3127037
monophenol monooxygenase	1.14.18.1	Rat	3143738
monophenol monooxygenase	1.14.18.1	Rat	3148919
monophenol monooxygenase	1.14.18.1	Rat	3927896
monophenol monooxygenase	1.14.18.1	Rat	6826662
monophenol monooxygenase	1.14.18.1	Rat	7646526
monophenol monooxygenase	1.14.18.1	Rat	7685020
monophenol monooxygenase	1.14.18.1	Rat	7775602
monophenol monooxygenase	1.14.18.1	Rat	7792254
monophenol monooxygenase	1.14.18.1	Rat	7811291
monophenol monooxygenase	1.14.18.1	Rat	7821799
monophenol monooxygenase	1.14.18.1	Rat	7969144
monophenol monooxygenase	1.14.18.1	Rat	8039502
monophenol monooxygenase	1.14.18.1	Rat	8124097
monophenol monooxygenase	1.14.18.1	Rat	8136316
monophenol monooxygenase	1.14.18.1	Rat	8138743
monophenol monooxygenase	1.14.18.1	Rat	8151127
monophenol monooxygenase	1.14.18.1	Rat	8197131
monophenol monooxygenase	1.14.18.1	Rat	8270948
monophenol monooxygenase	1.14.18.1	Rat	8292889
monophenol monooxygenase	1.14.18.1	Rat	8332936
monophenol monooxygenase	1.14.18.1	Rat	8433000
monophenol monooxygenase	1.14.18.1	Rat	8462726
monophenol monooxygenase	1.14.18.1	Rat	8496620
monophenol monooxygenase	1.14.18.1	Rat	8577719
monophenol monooxygenase	1.14.18.1	Rat	8577860
monophenol monooxygenase	1.14.18.1	Rat	8806453
monophenol monooxygenase	1.14.18.1	Rat	8861753
monophenol monooxygenase	1.14.18.1	Rat	9029814
monophenol monooxygenase	1.14.18.1	Rat	9056640
monophenol monooxygenase	1.14.18.1	Rat	9088570
monophenol monooxygenase	1.14.18.1	Rat	9177196

monophenol monooxygenase	1.14.18.1	Rat	9240530
monophenol monooxygenase	1.14.18.1	Rat	9417870
monophenol monooxygenase	1.14.18.1	Rat	9447965
monophenol monooxygenase	1.14.18.1	Rat	9475178
monophenol monooxygenase	1.14.18.1	Rat	9500554
monophenol monooxygenase	1.14.18.1	Rat	9500997
monophenol monooxygenase	1.14.18.1	Rat	9545341
monophenol monooxygenase	1.14.18.1	Rat	9593634
monophenol monooxygenase	1.14.18.1	Rat	9719456
monophenol monooxygenase	1.14.18.1	Rat	9736634
monophenol monooxygenase	1.14.18.1	Rat	9788743
monophenol monooxygenase	1.14.18.1	Rat	9822646
monophenol monooxygenase	1.14.18.1	Rat	9830058
monophenol monooxygenase	1.14.18.1	Rat	9853616
monophenol monooxygenase	1.14.18.1	Rat	9880801
CMP-N-acetylneuraminate monooxygenase	1.14.18.2	Rat	10320348
CMP-N-acetylneuraminate monooxygenase	1.14.18.2	Rat	11786991
CMP-N-acetylneuraminate monooxygenase	1.14.18.2	Rat	8132639
CMP-N-acetylneuraminate monooxygenase	1.14.18.2	Rat	8195125
stearoyl-CoA 9-desaturase	1.14.19.1	Rat	10484602
stearoyl-CoA 9-desaturase	1.14.19.1	Rat	10581155
stearoyl-CoA 9-desaturase	1.14.19.1	Rat	11352637
stearoyl-CoA 9-desaturase	1.14.19.1	Rat	11396956
stearoyl-CoA 9-desaturase	1.14.19.1	Rat	11415448
stearoyl-CoA 9-desaturase	1.14.19.1	Rat	11533264
stearoyl-CoA 9-desaturase	1.14.19.1	Rat	11677241
stearoyl-CoA 9-desaturase	1.14.19.1	Rat	12364560
stearoyl-CoA 9-desaturase	1.14.19.1	Rat	12815040
stearoyl-CoA 9-desaturase	1.14.19.1	Rat	14610276
stearoyl-CoA 9-desaturase	1.14.19.1	Rat	14654089
stearoyl-CoA 9-desaturase	1.14.19.1	Rat	14734557
stearoyl-CoA 9-desaturase	1.14.19.1	Rat	15096593
stearoyl-CoA 9-desaturase	1.14.19.1	Rat	15180999
stearoyl-CoA 9-desaturase	1.14.19.1	Rat	15556291
stearoyl-CoA 9-desaturase	1.14.19.1	Rat	15726820
stearoyl-CoA 9-desaturase	1.14.19.1	Rat	15761499
stearoyl-CoA 9-desaturase	1.14.19.1	Rat	15829484
stearoyl-CoA 9-desaturase	1.14.19.1	Rat	15836467
stearoyl-CoA 9-desaturase	1.14.19.1	Rat	16245372
stearoyl-CoA 9-desaturase	1.14.19.1	Rat	16767221
stearoyl-CoA 9-desaturase	1.14.19.1	Rat	16804073
stearoyl-CoA 9-desaturase	1.14.19.1	Rat	9272444
prostaglandin-endoperoxide synthase	1.14.99.1	Rat	10024686

prostaglandin-endoperoxide synthase	1.14.99.1	Rat	10091284
prostaglandin-endoperoxide synthase	1.14.99.1	Rat	10226539
prostaglandin-endoperoxide synthase	1.14.99.1	Rat	10228831
prostaglandin-endoperoxide synthase	1.14.99.1	Rat	10365086
prostaglandin-endoperoxide synthase	1.14.99.1	Rat	10395693
prostaglandin-endoperoxide synthase	1.14.99.1	Rat	10399316
prostaglandin-endoperoxide synthase	1.14.99.1	Rat	10425287
prostaglandin-endoperoxide synthase	1.14.99.1	Rat	10426495
prostaglandin-endoperoxide synthase	1.14.99.1	Rat	10560661
prostaglandin-endoperoxide synthase	1.14.99.1	Rat	10652449
prostaglandin-endoperoxide synthase	1.14.99.1	Rat	10667579
prostaglandin-endoperoxide synthase	1.14.99.1	Rat	10754263
prostaglandin-endoperoxide synthase	1.14.99.1	Rat	10807888
prostaglandin-endoperoxide synthase	1.14.99.1	Rat	10859338
prostaglandin-endoperoxide synthase	1.14.99.1	Rat	10935540
prostaglandin-endoperoxide synthase	1.14.99.1	Rat	10991924
prostaglandin-endoperoxide synthase	1.14.99.1	Rat	10999846
prostaglandin-endoperoxide synthase	1.14.99.1	Rat	11153163
prostaglandin-endoperoxide synthase	1.14.99.1	Rat	11192946
prostaglandin-endoperoxide synthase	1.14.99.1	Rat	11220737
prostaglandin-endoperoxide synthase	1.14.99.1	Rat	11239825
prostaglandin-endoperoxide synthase	1.14.99.1	Rat	11303759
prostaglandin-endoperoxide synthase	1.14.99.1	Rat	11338298
prostaglandin-endoperoxide synthase	1.14.99.1	Rat	11338376
prostaglandin-endoperoxide synthase	1.14.99.1	Rat	11407522
prostaglandin-endoperoxide synthase	1.14.99.1	Rat	11427038
prostaglandin-endoperoxide synthase	1.14.99.1	Rat	11451994
prostaglandin-endoperoxide synthase	1.14.99.1	Rat	11455568
prostaglandin-endoperoxide synthase	1.14.99.1	Rat	11501838
prostaglandin-endoperoxide synthase	1.14.99.1	Rat	11506967
prostaglandin-endoperoxide synthase	1.14.99.1	Rat	11519793
prostaglandin-endoperoxide synthase	1.14.99.1	Rat	11520057
prostaglandin-endoperoxide synthase	1.14.99.1	Rat	11588192
prostaglandin-endoperoxide synthase	1.14.99.1	Rat	11673276
prostaglandin-endoperoxide synthase	1.14.99.1	Rat	11746474
prostaglandin-endoperoxide synthase	1.14.99.1	Rat	11758826
prostaglandin-endoperoxide synthase	1.14.99.1	Rat	11781662
prostaglandin-endoperoxide synthase	1.14.99.1	Rat	11857443
prostaglandin-endoperoxide synthase	1.14.99.1	Rat	11870370
prostaglandin-endoperoxide synthase	1.14.99.1	Rat	11945149
prostaglandin-endoperoxide synthase	1.14.99.1	Rat	11966528
prostaglandin-endoperoxide synthase	1.14.99.1	Rat	11991685
prostaglandin-endoperoxide synthase	1.14.99.1	Rat	12021045

prostaglandin-endoperoxide synthase	1.14.99.1	Rat	12034746
prostaglandin-endoperoxide synthase	1.14.99.1	Rat	12047931
prostaglandin-endoperoxide synthase	1.14.99.1	Rat	12211105
prostaglandin-endoperoxide synthase	1.14.99.1	Rat	12242329
prostaglandin-endoperoxide synthase	1.14.99.1	Rat	12349897
prostaglandin-endoperoxide synthase	1.14.99.1	Rat	12352459
prostaglandin-endoperoxide synthase	1.14.99.1	Rat	12377741
prostaglandin-endoperoxide synthase	1.14.99.1	Rat	12397176
prostaglandin-endoperoxide synthase	1.14.99.1	Rat	12414874
prostaglandin-endoperoxide synthase	1.14.99.1	Rat	12432912
prostaglandin-endoperoxide synthase	1.14.99.1	Rat	12467525
prostaglandin-endoperoxide synthase	1.14.99.1	Rat	12468643
prostaglandin-endoperoxide synthase	1.14.99.1	Rat	12491803
prostaglandin-endoperoxide synthase	1.14.99.1	Rat	12502903
prostaglandin-endoperoxide synthase	1.14.99.1	Rat	12576462
prostaglandin-endoperoxide synthase	1.14.99.1	Rat	12604670
prostaglandin-endoperoxide synthase	1.14.99.1	Rat	12614262
prostaglandin-endoperoxide synthase	1.14.99.1	Rat	12669881
prostaglandin-endoperoxide synthase	1.14.99.1	Rat	12683423
prostaglandin-endoperoxide synthase	1.14.99.1	Rat	12735864
prostaglandin-endoperoxide synthase	1.14.99.1	Rat	12845600
prostaglandin-endoperoxide synthase	1.14.99.1	Rat	12846004
prostaglandin-endoperoxide synthase	1.14.99.1	Rat	12885872
prostaglandin-endoperoxide synthase	1.14.99.1	Rat	12969240
prostaglandin-endoperoxide synthase	1.14.99.1	Rat	12970891
prostaglandin-endoperoxide synthase	1.14.99.1	Rat	1305681
prostaglandin-endoperoxide synthase	1.14.99.1	Rat	1316915
prostaglandin-endoperoxide synthase	1.14.99.1	Rat	1400321
prostaglandin-endoperoxide synthase	1.14.99.1	Rat	1410519
prostaglandin-endoperoxide synthase	1.14.99.1	Rat	14518562
prostaglandin-endoperoxide synthase	1.14.99.1	Rat	14566678
prostaglandin-endoperoxide synthase	1.14.99.1	Rat	14634339
prostaglandin-endoperoxide synthase	1.14.99.1	Rat	14704742
prostaglandin-endoperoxide synthase	1.14.99.1	Rat	14707443
prostaglandin-endoperoxide synthase	1.14.99.1	Rat	14743889
prostaglandin-endoperoxide synthase	1.14.99.1	Rat	1480760
prostaglandin-endoperoxide synthase	1.14.99.1	Rat	14965326
prostaglandin-endoperoxide synthase	1.14.99.1	Rat	15037210
prostaglandin-endoperoxide synthase	1.14.99.1	Rat	15106811
prostaglandin-endoperoxide synthase	1.14.99.1	Rat	15109739
prostaglandin-endoperoxide synthase	1.14.99.1	Rat	15148333
prostaglandin-endoperoxide synthase	1.14.99.1	Rat	15205395
prostaglandin-endoperoxide synthase	1.14.99.1	Rat	15299086

prostaglandin-endoperoxide synthase	1.14.99.1	Rat	15351039
prostaglandin-endoperoxide synthase	1.14.99.1	Rat	15486039
prostaglandin-endoperoxide synthase	1.14.99.1	Rat	15496409
prostaglandin-endoperoxide synthase	1.14.99.1	Rat	15507510
prostaglandin-endoperoxide synthase	1.14.99.1	Rat	15596243
prostaglandin-endoperoxide synthase	1.14.99.1	Rat	15604817
prostaglandin-endoperoxide synthase	1.14.99.1	Rat	15639337
prostaglandin-endoperoxide synthase	1.14.99.1	Rat	15653788
prostaglandin-endoperoxide synthase	1.14.99.1	Rat	15668527
prostaglandin-endoperoxide synthase	1.14.99.1	Rat	15753380
prostaglandin-endoperoxide synthase	1.14.99.1	Rat	15809059
prostaglandin-endoperoxide synthase	1.14.99.1	Rat	15821352
prostaglandin-endoperoxide synthase	1.14.99.1	Rat	15885672
prostaglandin-endoperoxide synthase	1.14.99.1	Rat	15889237
prostaglandin-endoperoxide synthase	1.14.99.1	Rat	15900018
prostaglandin-endoperoxide synthase	1.14.99.1	Rat	15992053
prostaglandin-endoperoxide synthase	1.14.99.1	Rat	15993594
prostaglandin-endoperoxide synthase	1.14.99.1	Rat	16046792
prostaglandin-endoperoxide synthase	1.14.99.1	Rat	16137646
prostaglandin-endoperoxide synthase	1.14.99.1	Rat	16169091
prostaglandin-endoperoxide synthase	1.14.99.1	Rat	16169124
prostaglandin-endoperoxide synthase	1.14.99.1	Rat	16253229
prostaglandin-endoperoxide synthase	1.14.99.1	Rat	16284883
prostaglandin-endoperoxide synthase	1.14.99.1	Rat	16445867
prostaglandin-endoperoxide synthase	1.14.99.1	Rat	16458279
prostaglandin-endoperoxide synthase	1.14.99.1	Rat	16493584
prostaglandin-endoperoxide synthase	1.14.99.1	Rat	16516846
prostaglandin-endoperoxide synthase	1.14.99.1	Rat	16529558
prostaglandin-endoperoxide synthase	1.14.99.1	Rat	16709833
prostaglandin-endoperoxide synthase	1.14.99.1	Rat	16716827
prostaglandin-endoperoxide synthase	1.14.99.1	Rat	16740978
prostaglandin-endoperoxide synthase	1.14.99.1	Rat	16753269
prostaglandin-endoperoxide synthase	1.14.99.1	Rat	16827136
prostaglandin-endoperoxide synthase	1.14.99.1	Rat	16840740
prostaglandin-endoperoxide synthase	1.14.99.1	Rat	16842132
prostaglandin-endoperoxide synthase	1.14.99.1	Rat	16842185
prostaglandin-endoperoxide synthase	1.14.99.1	Rat	16847764
prostaglandin-endoperoxide synthase	1.14.99.1	Rat	16870271
prostaglandin-endoperoxide synthase	1.14.99.1	Rat	16871410
prostaglandin-endoperoxide synthase	1.14.99.1	Rat	16872525
prostaglandin-endoperoxide synthase	1.14.99.1	Rat	16885386
prostaglandin-endoperoxide synthase	1.14.99.1	Rat	1694171
prostaglandin-endoperoxide synthase	1.14.99.1	Rat	16966442

prostaglandin-endoperoxide synthase	1.14.99.1	Rat	1706264
prostaglandin-endoperoxide synthase	1.14.99.1	Rat	17066444
prostaglandin-endoperoxide synthase	1.14.99.1	Rat	17082565
prostaglandin-endoperoxide synthase	1.14.99.1	Rat	17140386
prostaglandin-endoperoxide synthase	1.14.99.1	Rat	1721064
prostaglandin-endoperoxide synthase	1.14.99.1	Rat	1730539
prostaglandin-endoperoxide synthase	1.14.99.1	Rat	1731622
prostaglandin-endoperoxide synthase	1.14.99.1	Rat	1744122
prostaglandin-endoperoxide synthase	1.14.99.1	Rat	1836009
prostaglandin-endoperoxide synthase	1.14.99.1	Rat	1903222
prostaglandin-endoperoxide synthase	1.14.99.1	Rat	2125559
prostaglandin-endoperoxide synthase	1.14.99.1	Rat	2150736
prostaglandin-endoperoxide synthase	1.14.99.1	Rat	2497108
prostaglandin-endoperoxide synthase	1.14.99.1	Rat	2519899
prostaglandin-endoperoxide synthase	1.14.99.1	Rat	26389
prostaglandin-endoperoxide synthase	1.14.99.1	Rat	2770427
prostaglandin-endoperoxide synthase	1.14.99.1	Rat	3040660
prostaglandin-endoperoxide synthase	1.14.99.1	Rat	3510842
prostaglandin-endoperoxide synthase	1.14.99.1	Rat	6197028
prostaglandin-endoperoxide synthase	1.14.99.1	Rat	6252692
prostaglandin-endoperoxide synthase	1.14.99.1	Rat	6312489
prostaglandin-endoperoxide synthase	1.14.99.1	Rat	6324240
prostaglandin-endoperoxide synthase	1.14.99.1	Rat	663276
prostaglandin-endoperoxide synthase	1.14.99.1	Rat	7229430
prostaglandin-endoperoxide synthase	1.14.99.1	Rat	7635765
prostaglandin-endoperoxide synthase	1.14.99.1	Rat	7706373
prostaglandin-endoperoxide synthase	1.14.99.1	Rat	7775455
prostaglandin-endoperoxide synthase	1.14.99.1	Rat	7784470
prostaglandin-endoperoxide synthase	1.14.99.1	Rat	7920026
prostaglandin-endoperoxide synthase	1.14.99.1	Rat	7926468
prostaglandin-endoperoxide synthase	1.14.99.1	Rat	7980528
prostaglandin-endoperoxide synthase	1.14.99.1	Rat	8132578
prostaglandin-endoperoxide synthase	1.14.99.1	Rat	8163473
prostaglandin-endoperoxide synthase	1.14.99.1	Rat	8188473
prostaglandin-endoperoxide synthase	1.14.99.1	Rat	8261019
prostaglandin-endoperoxide synthase	1.14.99.1	Rat	8280164
prostaglandin-endoperoxide synthase	1.14.99.1	Rat	8365485
prostaglandin-endoperoxide synthase	1.14.99.1	Rat	8521479
prostaglandin-endoperoxide synthase	1.14.99.1	Rat	8616915
prostaglandin-endoperoxide synthase	1.14.99.1	Rat	8641206
prostaglandin-endoperoxide synthase	1.14.99.1	Rat	8653697
prostaglandin-endoperoxide synthase	1.14.99.1	Rat	8752493
prostaglandin-endoperoxide synthase	1.14.99.1	Rat	8918053

prostaglandin-endoperoxide synthase	1.14.99.1	Rat	9013627
prostaglandin-endoperoxide synthase	1.14.99.1	Rat	9057838
prostaglandin-endoperoxide synthase	1.14.99.1	Rat	9096394
prostaglandin-endoperoxide synthase	1.14.99.1	Rat	9102213
prostaglandin-endoperoxide synthase	1.14.99.1	Rat	9126611
prostaglandin-endoperoxide synthase	1.14.99.1	Rat	9144511
prostaglandin-endoperoxide synthase	1.14.99.1	Rat	9177102
prostaglandin-endoperoxide synthase	1.14.99.1	Rat	9177247
prostaglandin-endoperoxide synthase	1.14.99.1	Rat	9261162
prostaglandin-endoperoxide synthase	1.14.99.1	Rat	9360765
prostaglandin-endoperoxide synthase	1.14.99.1	Rat	9373878
prostaglandin-endoperoxide synthase	1.14.99.1	Rat	9383435
prostaglandin-endoperoxide synthase	1.14.99.1	Rat	9402302
prostaglandin-endoperoxide synthase	1.14.99.1	Rat	9458802
prostaglandin-endoperoxide synthase	1.14.99.1	Rat	9483916
prostaglandin-endoperoxide synthase	1.14.99.1	Rat	9492015
prostaglandin-endoperoxide synthase	1.14.99.1	Rat	9513902
prostaglandin-endoperoxide synthase	1.14.99.1	Rat	9517759
prostaglandin-endoperoxide synthase	1.14.99.1	Rat	9521170
prostaglandin-endoperoxide synthase	1.14.99.1	Rat	9526843
prostaglandin-endoperoxide synthase	1.14.99.1	Rat	9562240
prostaglandin-endoperoxide synthase	1.14.99.1	Rat	9568703
prostaglandin-endoperoxide synthase	1.14.99.1	Rat	9585093
prostaglandin-endoperoxide synthase	1.14.99.1	Rat	9649571
prostaglandin-endoperoxide synthase	1.14.99.1	Rat	97337
prostaglandin-endoperoxide synthase	1.14.99.1	Rat	9740394
prostaglandin-endoperoxide synthase	1.14.99.1	Rat	9758208
prostaglandin-endoperoxide synthase	1.14.99.1	Rat	9764845
prostaglandin-endoperoxide synthase	1.14.99.1	Rat	9790545
prostaglandin-endoperoxide synthase	1.14.99.1	Rat	9811310
prostaglandin-endoperoxide synthase	1.14.99.1	Rat	9823297
prostaglandin-endoperoxide synthase	1.14.99.1	Rat	9870924
prostaglandin-endoperoxide synthase	1.14.99.1	Rat	9879525
heme oxygenase	1.14.99.3	Rat	10349844
heme oxygenase	1.14.99.3	Rat	10353745
heme oxygenase	1.14.99.3	Rat	10630670
heme oxygenase	1.14.99.3	Rat	10634305
heme oxygenase	1.14.99.3	Rat	10644516
heme oxygenase	1.14.99.3	Rat	10692503
heme oxygenase	1.14.99.3	Rat	10727990
heme oxygenase	1.14.99.3	Rat	10793064
heme oxygenase	1.14.99.3	Rat	10814519
heme oxygenase	1.14.99.3	Rat	10961657

heme oxygenase	1.14.99.3	Rat	11007950
heme oxygenase	1.14.99.3	Rat	11135063
heme oxygenase	1.14.99.3	Rat	11258550
heme oxygenase	1.14.99.3	Rat	11498995
heme oxygenase	1.14.99.3	Rat	11591199
heme oxygenase	1.14.99.3	Rat	11592943
heme oxygenase	1.14.99.3	Rat	11698254
heme oxygenase	1.14.99.3	Rat	11705458
heme oxygenase	1.14.99.3	Rat	11758836
heme oxygenase	1.14.99.3	Rat	11801258
heme oxygenase	1.14.99.3	Rat	11803410
heme oxygenase	1.14.99.3	Rat	11868390
heme oxygenase	1.14.99.3	Rat	11978880
heme oxygenase	1.14.99.3	Rat	12042070
heme oxygenase	1.14.99.3	Rat	12057765
heme oxygenase	1.14.99.3	Rat	12117910
heme oxygenase	1.14.99.3	Rat	12136229
heme oxygenase	1.14.99.3	Rat	12230871
heme oxygenase	1.14.99.3	Rat	12236785
heme oxygenase	1.14.99.3	Rat	12372789
heme oxygenase	1.14.99.3	Rat	12372951
heme oxygenase	1.14.99.3	Rat	12397597
heme oxygenase	1.14.99.3	Rat	12489116
heme oxygenase	1.14.99.3	Rat	12508080
heme oxygenase	1.14.99.3	Rat	12581208
heme oxygenase	1.14.99.3	Rat	12646399
heme oxygenase	1.14.99.3	Rat	12668974
heme oxygenase	1.14.99.3	Rat	12709591
heme oxygenase	1.14.99.3	Rat	12736395
heme oxygenase	1.14.99.3	Rat	12757849
heme oxygenase	1.14.99.3	Rat	12783778
heme oxygenase	1.14.99.3	Rat	12874251
heme oxygenase	1.14.99.3	Rat	12892830
heme oxygenase	1.14.99.3	Rat	12963497
heme oxygenase	1.14.99.3	Rat	14523007
heme oxygenase	1.14.99.3	Rat	14649057
heme oxygenase	1.14.99.3	Rat	14683741
heme oxygenase	1.14.99.3	Rat	14761930
heme oxygenase	1.14.99.3	Rat	14766239
heme oxygenase	1.14.99.3	Rat	14985830
heme oxygenase	1.14.99.3	Rat	15018736
heme oxygenase	1.14.99.3	Rat	15276480
heme oxygenase	1.14.99.3	Rat	15298625

heme oxygenase	1.14.99.3	Rat	15345139
heme oxygenase	1.14.99.3	Rat	15345147
heme oxygenase	1.14.99.3	Rat	15345149
heme oxygenase	1.14.99.3	Rat	15499042
heme oxygenase	1.14.99.3	Rat	15499991
heme oxygenase	1.14.99.3	Rat	15565657
heme oxygenase	1.14.99.3	Rat	15588929
heme oxygenase	1.14.99.3	Rat	15599843
heme oxygenase	1.14.99.3	Rat	15649645
heme oxygenase	1.14.99.3	Rat	1572205
heme oxygenase	1.14.99.3	Rat	15833736
heme oxygenase	1.14.99.3	Rat	15869055
heme oxygenase	1.14.99.3	Rat	15896346
heme oxygenase	1.14.99.3	Rat	15897578
heme oxygenase	1.14.99.3	Rat	15933765
heme oxygenase	1.14.99.3	Rat	16043027
heme oxygenase	1.14.99.3	Rat	16048902
heme oxygenase	1.14.99.3	Rat	16123320
heme oxygenase	1.14.99.3	Rat	16181105
heme oxygenase	1.14.99.3	Rat	16181109
heme oxygenase	1.14.99.3	Rat	16214041
heme oxygenase	1.14.99.3	Rat	16276181
heme oxygenase	1.14.99.3	Rat	16309569
heme oxygenase	1.14.99.3	Rat	16319139
heme oxygenase	1.14.99.3	Rat	16374439
heme oxygenase	1.14.99.3	Rat	16476737
heme oxygenase	1.14.99.3	Rat	16598857
heme oxygenase	1.14.99.3	Rat	16678019
heme oxygenase	1.14.99.3	Rat	16775600
heme oxygenase	1.14.99.3	Rat	16828975
heme oxygenase	1.14.99.3	Rat	16858012
heme oxygenase	1.14.99.3	Rat	16948473
heme oxygenase	1.14.99.3	Rat	16964402
heme oxygenase	1.14.99.3	Rat	16966352
heme oxygenase	1.14.99.3	Rat	17002867
heme oxygenase	1.14.99.3	Rat	1700666
heme oxygenase	1.14.99.3	Rat	17042977
heme oxygenase	1.14.99.3	Rat	1737989
heme oxygenase	1.14.99.3	Rat	1884769
heme oxygenase	1.14.99.3	Rat	2759552
heme oxygenase	1.14.99.3	Rat	3107908
heme oxygenase	1.14.99.3	Rat	3113993
heme oxygenase	1.14.99.3	Rat	3177434

heme oxygenase	1.14.99.3	Rat	3965329
heme oxygenase	1.14.99.3	Rat	6548384
heme oxygenase	1.14.99.3	Rat	7525927
heme oxygenase	1.14.99.3	Rat	7576696
heme oxygenase	1.14.99.3	Rat	7626076
heme oxygenase	1.14.99.3	Rat	7768976
heme oxygenase	1.14.99.3	Rat	7793979
heme oxygenase	1.14.99.3	Rat	7889361
heme oxygenase	1.14.99.3	Rat	8016102
heme oxygenase	1.14.99.3	Rat	8216309
heme oxygenase	1.14.99.3	Rat	8548408
heme oxygenase	1.14.99.3	Rat	8564848
heme oxygenase	1.14.99.3	Rat	8590277
heme oxygenase	1.14.99.3	Rat	8694803
heme oxygenase	1.14.99.3	Rat	8774698
heme oxygenase	1.14.99.3	Rat	8816811
heme oxygenase	1.14.99.3	Rat	8827779
heme oxygenase	1.14.99.3	Rat	8852585
heme oxygenase	1.14.99.3	Rat	8872603
heme oxygenase	1.14.99.3	Rat	8897916
heme oxygenase	1.14.99.3	Rat	8913885
heme oxygenase	1.14.99.3	Rat	9125512
heme oxygenase	1.14.99.3	Rat	9225984
heme oxygenase	1.14.99.3	Rat	9236721
heme oxygenase	1.14.99.3	Rat	9259978
heme oxygenase	1.14.99.3	Rat	9276739
heme oxygenase	1.14.99.3	Rat	9495815
heme oxygenase	1.14.99.3	Rat	9530200
heme oxygenase	1.14.99.3	Rat	9608682
heme oxygenase	1.14.99.3	Rat	9927151
squalene monooxygenase	1.14.99.7	Rat	10679280
squalene monooxygenase	1.14.99.7	Rat	10733917
squalene monooxygenase	1.14.99.7	Rat	11199136
squalene monooxygenase	1.14.99.7	Rat	11520216
squalene monooxygenase	1.14.99.7	Rat	12226513
squalene monooxygenase	1.14.99.7	Rat	16466954
squalene monooxygenase	1.14.99.7	Rat	6087072
squalene monooxygenase	1.14.99.7	Rat	6547247
squalene monooxygenase	1.14.99.7	Rat	8771716
squalene monooxygenase	1.14.99.7	Rat	9017503
steroid 17alpha-monooxygenase	1.14.99.9	Rat	10066888
steroid 17alpha-monooxygenase	1.14.99.9	Rat	10352919
steroid 17alpha-monooxygenase	1.14.99.9	Rat	10574247

steroid 17alpha-monooxygenase	1.14.99.9	Rat	11446160
steroid 17alpha-monooxygenase	1.14.99.9	Rat	11817165
steroid 17alpha-monooxygenase	1.14.99.9	Rat	12444467
steroid 17alpha-monooxygenase	1.14.99.9	Rat	15001547
steroid 17alpha-monooxygenase	1.14.99.9	Rat	16022662
steroid 17alpha-monooxygenase	1.14.99.9	Rat	1605399
steroid 17alpha-monooxygenase	1.14.99.9	Rat	1607640
steroid 17alpha-monooxygenase	1.14.99.9	Rat	1958544
steroid 17alpha-monooxygenase	1.14.99.9	Rat	2019257
steroid 17alpha-monooxygenase	1.14.99.9	Rat	3502608
steroid 17alpha-monooxygenase	1.14.99.9	Rat	6593324
steroid 17alpha-monooxygenase	1.14.99.9	Rat	9115645
steroid 17alpha-monooxygenase	1.14.99.9	Rat	9408743
steroid 17alpha-monooxygenase	1.14.99.9	Rat	9892022
xanthine dehydrogenase	1.17.1.4	Rat	11086257
xanthine dehydrogenase	1.17.1.4	Rat	11154741
xanthine dehydrogenase	1.17.1.4	Rat	12502743
xanthine oxidase	1.17.3.2	Rat	11086257
xanthine oxidase	1.17.3.2	Rat	11278616
xanthine oxidase	1.17.3.2	Rat	12423257
xanthine oxidase	1.17.3.2	Rat	12618887
xanthine oxidase	1.17.3.2	Rat	12780970
xanthine oxidase	1.17.3.2	Rat	1353938
xanthine oxidase	1.17.3.2	Rat	16429783
xanthine oxidase	1.17.3.2	Rat	187329
xanthine oxidase	1.17.3.2	Rat	1889832
xanthine oxidase	1.17.3.2	Rat	3245099
xanthine oxidase	1.17.3.2	Rat	8274165
ribonucleoside-diphosphate reductase	1.17.4.1	Rat	10441745
ribonucleoside-diphosphate reductase	1.17.4.1	Rat	10593972
ribonucleoside-diphosphate reductase	1.17.4.1	Rat	10769119
ribonucleoside-diphosphate reductase	1.17.4.1	Rat	10805162
ribonucleoside-diphosphate reductase	1.17.4.1	Rat	10953295
ribonucleoside-diphosphate reductase	1.17.4.1	Rat	10989193
ribonucleoside-diphosphate reductase	1.17.4.1	Rat	11489836
ribonucleoside-diphosphate reductase	1.17.4.1	Rat	11551528
ribonucleoside-diphosphate reductase	1.17.4.1	Rat	11904430
ribonucleoside-diphosphate reductase	1.17.4.1	Rat	12147300
ribonucleoside-diphosphate reductase	1.17.4.1	Rat	12359454
ribonucleoside-diphosphate reductase	1.17.4.1	Rat	12655059
ribonucleoside-diphosphate reductase	1.17.4.1	Rat	12690517
ribonucleoside-diphosphate reductase	1.17.4.1	Rat	12732713
ribonucleoside-diphosphate reductase	1.17.4.1	Rat	12749906

ribonucleoside-diphosphate reductase	1.17.4.1	Rat	12967138
ribonucleoside-diphosphate reductase	1.17.4.1	Rat	1299271
ribonucleoside-diphosphate reductase	1.17.4.1	Rat	1385411
ribonucleoside-diphosphate reductase	1.17.4.1	Rat	1412696
ribonucleoside-diphosphate reductase	1.17.4.1	Rat	14963934
ribonucleoside-diphosphate reductase	1.17.4.1	Rat	14966112
ribonucleoside-diphosphate reductase	1.17.4.1	Rat	1496919
ribonucleoside-diphosphate reductase	1.17.4.1	Rat	15094776
ribonucleoside-diphosphate reductase	1.17.4.1	Rat	15133626
ribonucleoside-diphosphate reductase	1.17.4.1	Rat	1516817
ribonucleoside-diphosphate reductase	1.17.4.1	Rat	15300180
ribonucleoside-diphosphate reductase	1.17.4.1	Rat	15571292
ribonucleoside-diphosphate reductase	1.17.4.1	Rat	15656518
ribonucleoside-diphosphate reductase	1.17.4.1	Rat	15673563
ribonucleoside-diphosphate reductase	1.17.4.1	Rat	15730856
ribonucleoside-diphosphate reductase	1.17.4.1	Rat	15769467
ribonucleoside-diphosphate reductase	1.17.4.1	Rat	15803490
ribonucleoside-diphosphate reductase	1.17.4.1	Rat	15805194
ribonucleoside-diphosphate reductase	1.17.4.1	Rat	15888728
ribonucleoside-diphosphate reductase	1.17.4.1	Rat	160558
ribonucleoside-diphosphate reductase	1.17.4.1	Rat	16399800
ribonucleoside-diphosphate reductase	1.17.4.1	Rat	1643157
ribonucleoside-diphosphate reductase	1.17.4.1	Rat	16489218
ribonucleoside-diphosphate reductase	1.17.4.1	Rat	16530987
ribonucleoside-diphosphate reductase	1.17.4.1	Rat	16834759
ribonucleoside-diphosphate reductase	1.17.4.1	Rat	16925573
ribonucleoside-diphosphate reductase	1.17.4.1	Rat	17065057
ribonucleoside-diphosphate reductase	1.17.4.1	Rat	1717630
ribonucleoside-diphosphate reductase	1.17.4.1	Rat	1748682
ribonucleoside-diphosphate reductase	1.17.4.1	Rat	1793565
ribonucleoside-diphosphate reductase	1.17.4.1	Rat	2085432
ribonucleoside-diphosphate reductase	1.17.4.1	Rat	2178608
ribonucleoside-diphosphate reductase	1.17.4.1	Rat	2199320
ribonucleoside-diphosphate reductase	1.17.4.1	Rat	2257322
ribonucleoside-diphosphate reductase	1.17.4.1	Rat	2642388
ribonucleoside-diphosphate reductase	1.17.4.1	Rat	2673261
ribonucleoside-diphosphate reductase	1.17.4.1	Rat	2775821
ribonucleoside-diphosphate reductase	1.17.4.1	Rat	2827767
ribonucleoside-diphosphate reductase	1.17.4.1	Rat	2832057
ribonucleoside-diphosphate reductase	1.17.4.1	Rat	3044371
ribonucleoside-diphosphate reductase	1.17.4.1	Rat	3044582
ribonucleoside-diphosphate reductase	1.17.4.1	Rat	3061459
ribonucleoside-diphosphate reductase	1.17.4.1	Rat	3300645

ribonucleoside-diphosphate reductase	1.17.4.1	Rat	3511848
ribonucleoside-diphosphate reductase	1.17.4.1	Rat	3536076
ribonucleoside-diphosphate reductase	1.17.4.1	Rat	3907637
ribonucleoside-diphosphate reductase	1.17.4.1	Rat	3914643
ribonucleoside-diphosphate reductase	1.17.4.1	Rat	3915189
ribonucleoside-diphosphate reductase	1.17.4.1	Rat	3986794
ribonucleoside-diphosphate reductase	1.17.4.1	Rat	6353195
ribonucleoside-diphosphate reductase	1.17.4.1	Rat	6375753
ribonucleoside-diphosphate reductase	1.17.4.1	Rat	6752137
ribonucleoside-diphosphate reductase	1.17.4.1	Rat	6757589
ribonucleoside-diphosphate reductase	1.17.4.1	Rat	767333
ribonucleoside-diphosphate reductase	1.17.4.1	Rat	7727399
ribonucleoside-diphosphate reductase	1.17.4.1	Rat	7768988
ribonucleoside-diphosphate reductase	1.17.4.1	Rat	7838172
ribonucleoside-diphosphate reductase	1.17.4.1	Rat	7881162
ribonucleoside-diphosphate reductase	1.17.4.1	Rat	7882331
ribonucleoside-diphosphate reductase	1.17.4.1	Rat	7893463
ribonucleoside-diphosphate reductase	1.17.4.1	Rat	7937896
ribonucleoside-diphosphate reductase	1.17.4.1	Rat	7984431
ribonucleoside-diphosphate reductase	1.17.4.1	Rat	8241321
ribonucleoside-diphosphate reductase	1.17.4.1	Rat	8265664
ribonucleoside-diphosphate reductase	1.17.4.1	Rat	8343143
ribonucleoside-diphosphate reductase	1.17.4.1	Rat	8463252
ribonucleoside-diphosphate reductase	1.17.4.1	Rat	8521087
ribonucleoside-diphosphate reductase	1.17.4.1	Rat	8620054
ribonucleoside-diphosphate reductase	1.17.4.1	Rat	8662944
ribonucleoside-diphosphate reductase	1.17.4.1	Rat	8674535
ribonucleoside-diphosphate reductase	1.17.4.1	Rat	8813126
ribonucleoside-diphosphate reductase	1.17.4.1	Rat	8878781
ribonucleoside-diphosphate reductase	1.17.4.1	Rat	8920917
ribonucleoside-diphosphate reductase	1.17.4.1	Rat	8943056
ribonucleoside-diphosphate reductase	1.17.4.1	Rat	9192674
ribonucleoside-diphosphate reductase	1.17.4.1	Rat	9315670
ribonucleoside-diphosphate reductase	1.17.4.1	Rat	9347313
ribonucleoside-diphosphate reductase	1.17.4.1	Rat	9354452
ribonucleoside-diphosphate reductase	1.17.4.1	Rat	9371820
ribonucleoside-diphosphate reductase	1.17.4.1	Rat	9393942
ribonucleoside-diphosphate reductase	1.17.4.1	Rat	9415718
ribonucleoside-diphosphate reductase	1.17.4.1	Rat	9439883
ribonucleoside-diphosphate reductase	1.17.4.1	Rat	9558318
ribonucleoside-diphosphate reductase	1.17.4.1	Rat	9570515
ribonucleoside-diphosphate reductase	1.17.4.1	Rat	9598136
ribonucleoside-diphosphate reductase	1.17.4.1	Rat	9605773

ribonucleoside-diphosphate reductase	1.17.4.1	Rat	9634002
ribonucleoside-diphosphate reductase	1.17.4.1	Rat	9696008
ribonucleoside-diphosphate reductase	1.17.4.1	Rat	9718080
ribonucleoside-diphosphate reductase	1.17.4.1	Rat	9852067
ribonucleoside-diphosphate reductase	1.17.4.1	Rat	9990288
glyceraldehyde-3-phosphate dehydrogenase (phosphorylating)	1.2.1.12	Rat	10966377
glyceraldehyde-3-phosphate dehydrogenase (phosphorylating)	1.2.1.12	Rat	11018719
glyceraldehyde-3-phosphate dehydrogenase (phosphorylating)	1.2.1.12	Rat	12123463
glyceraldehyde-3-phosphate dehydrogenase (phosphorylating)	1.2.1.12	Rat	12634343
glyceraldehyde-3-phosphate dehydrogenase (phosphorylating)	1.2.1.12	Rat	14502604
glyceraldehyde-3-phosphate dehydrogenase (phosphorylating)	1.2.1.12	Rat	15299328
glyceraldehyde-3-phosphate dehydrogenase (phosphorylating)	1.2.1.12	Rat	15631980
glyceraldehyde-3-phosphate dehydrogenase (phosphorylating)	1.2.1.12	Rat	15680915
glyceraldehyde-3-phosphate dehydrogenase (phosphorylating)	1.2.1.12	Rat	3530169
glyceraldehyde-3-phosphate dehydrogenase (phosphorylating)	1.2.1.12	Rat	6095107
glyceraldehyde-3-phosphate dehydrogenase (phosphorylating)	1.2.1.12	Rat	6440018
glyceraldehyde-3-phosphate dehydrogenase (phosphorylating)	1.2.1.12	Rat	664114
glyceraldehyde-3-phosphate dehydrogenase (phosphorylating)	1.2.1.12	Rat	7165719
glyceraldehyde-3-phosphate dehydrogenase (phosphorylating)	1.2.1.12	Rat	7173737
glyceraldehyde-3-phosphate dehydrogenase (phosphorylating)	1.2.1.12	Rat	8304415
glyceraldehyde-3-phosphate dehydrogenase (phosphorylating)	1.2.1.12	Rat	8454610
glyceraldehyde-3-phosphate dehydrogenase (phosphorylating)	1.2.1.12	Rat	8791005
glyceraldehyde-3-phosphate dehydrogenase (phosphorylating)	1.2.1.12	Rat	8805794
glyceraldehyde-3-phosphate dehydrogenase (phosphorylating)	1.2.1.12	Rat	8905295

glyceraldehyde-3-phosphate dehydrogenase (phosphorylating)	1.2.1.12	Rat	9437188
glyceraldehyde-3-phosphate dehydrogenase (phosphorylating)	1.2.1.12	Rat	9793073
glyceraldehyde-3-phosphate dehydrogenase (phosphorylating)	1.2.1.12	Rat	9922941
aldehyde dehydrogenase (NAD+)	1.2.1.3	Rat	10913633
aldehyde dehydrogenase (NAD+)	1.2.1.3	Rat	11306027
aldehyde dehydrogenase (NAD+)	1.2.1.3	Rat	12223435
aldehyde dehydrogenase (NAD+)	1.2.1.3	Rat	14597338
aldehyde dehydrogenase (NAD+)	1.2.1.3	Rat	1472111
aldehyde dehydrogenase (NAD+)	1.2.1.3	Rat	16126235
aldehyde dehydrogenase (NAD+)	1.2.1.3	Rat	16499490
aldehyde dehydrogenase (NAD+)	1.2.1.3	Rat	16878979
aldehyde dehydrogenase (NAD+)	1.2.1.3	Rat	17607160
aldehyde dehydrogenase (NAD+)	1.2.1.3	Rat	2332351
aldehyde dehydrogenase (NAD+)	1.2.1.3	Rat	3593277
aldehyde dehydrogenase (NAD+)	1.2.1.3	Rat	6603890
aldehyde dehydrogenase (NAD+)	1.2.1.3	Rat	7910607
aldehyde dehydrogenase (NAD+)	1.2.1.3	Rat	8452208
aldehyde dehydrogenase (NAD+)	1.2.1.3	Rat	8823154
aldehyde dehydrogenase (NAD+)	1.2.1.3	Rat	8850269
aldehyde dehydrogenase (NAD+)	1.2.1.3	Rat	9013560
aldehyde dehydrogenase (NAD+)	1.2.1.3	Rat	9228057
aldehyde dehydrogenase (NAD+)	1.2.1.3	Rat	945270
retinal dehydrogenase	1.2.1.36	Rat	11600826
retinal dehydrogenase	1.2.1.36	Rat	12547725
retinal dehydrogenase	1.2.1.36	Rat	16763553
retinal dehydrogenase	1.2.1.36	Rat	17098734
retinal dehydrogenase	1.2.1.36	Rat	2910311
pyruvate dehydrogenase (acetyl-transferring)	1.2.4.1	Rat	10720420
pyruvate dehydrogenase (acetyl-transferring)	1.2.4.1	Rat	10759582
pyruvate dehydrogenase (acetyl-transferring)	1.2.4.1	Rat	10826498
pyruvate dehydrogenase (acetyl-transferring)	1.2.4.1	Rat	11223534
pyruvate dehydrogenase (acetyl-transferring)	1.2.4.1	Rat	11427685
pyruvate dehydrogenase (acetyl-transferring)	1.2.4.1	Rat	11557577
pyruvate dehydrogenase (acetyl-transferring)	1.2.4.1	Rat	11866475
pyruvate dehydrogenase (acetyl-transferring)	1.2.4.1	Rat	11900279
pyruvate dehydrogenase (acetyl-transferring)	1.2.4.1	Rat	12196468
pyruvate dehydrogenase (acetyl-transferring)	1.2.4.1	Rat	12557749
pyruvate dehydrogenase (acetyl-transferring)	1.2.4.1	Rat	12663261
pyruvate dehydrogenase (acetyl-transferring)	1.2.4.1	Rat	14607783
pyruvate dehydrogenase (acetyl-transferring)	1.2.4.1	Rat	14641018

pyruvate dehydrogenase (acetyl-transferring)	1.2.4.1	Rat	15191896
pyruvate dehydrogenase (acetyl-transferring)	1.2.4.1	Rat	15256563
pyruvate dehydrogenase (acetyl-transferring)	1.2.4.1	Rat	15921824
pyruvate dehydrogenase (acetyl-transferring)	1.2.4.1	Rat	1600837
pyruvate dehydrogenase (acetyl-transferring)	1.2.4.1	Rat	17065338
pyruvate dehydrogenase (acetyl-transferring)	1.2.4.1	Rat	2902801
pyruvate dehydrogenase (acetyl-transferring)	1.2.4.1	Rat	3127087
pyruvate dehydrogenase (acetyl-transferring)	1.2.4.1	Rat	3918587
pyruvate dehydrogenase (acetyl-transferring)	1.2.4.1	Rat	4030556
pyruvate dehydrogenase (acetyl-transferring)	1.2.4.1	Rat	6149743
pyruvate dehydrogenase (acetyl-transferring)	1.2.4.1	Rat	6234885
pyruvate dehydrogenase (acetyl-transferring)	1.2.4.1	Rat	7127258
pyruvate dehydrogenase (acetyl-transferring)	1.2.4.1	Rat	7848280
pyruvate dehydrogenase (acetyl-transferring)	1.2.4.1	Rat	7864215
pyruvate dehydrogenase (acetyl-transferring)	1.2.4.1	Rat	8557765
pyruvate dehydrogenase (acetyl-transferring)	1.2.4.1	Rat	9089387
pyruvate dehydrogenase (acetyl-transferring)	1.2.4.1	Rat	9381974
pyruvate dehydrogenase (acetyl-transferring)	1.2.4.1	Rat	9426381
pyruvate dehydrogenase (acetyl-transferring)	1.2.4.1	Rat	9438382
pyruvate dehydrogenase (acetyl-transferring)	1.2.4.1	Rat	9497164
pyruvate dehydrogenase (acetyl-transferring)	1.2.4.1	Rat	9725804
oxoglutarate dehydrogenase (succinyl-transferring)	1.2.4.2	Rat	1352447
oxoglutarate dehydrogenase (succinyl-transferring)	1.2.4.2	Rat	15356188
oxoglutarate dehydrogenase (succinyl-transferring)	1.2.4.2	Rat	15466852
oxoglutarate dehydrogenase (succinyl-transferring)	1.2.4.2	Rat	423807
oxoglutarate dehydrogenase (succinyl-transferring)	1.2.4.2	Rat	6778477
oxoglutarate dehydrogenase (succinyl-transferring)	1.2.4.2	Rat	7995856
3-methyl-2-oxobutanoate dehydrogenase	1.2.4.4	Rat	10562606
3-methyl-2-oxobutanoate dehydrogenase	1.2.4.4	Rat	11385053
3-methyl-2-oxobutanoate dehydrogenase	1.2.4.4	Rat	17656140
3-methyl-2-oxobutanoate dehydrogenase	1.2.4.4	Rat	3343251
3-methyl-2-oxobutanoate dehydrogenase	1.2.4.4	Rat	4054446
3-methyl-2-oxobutanoate dehydrogenase	1.2.4.4	Rat	6510417
3-methyl-2-oxobutanoate dehydrogenase	1.2.4.4	Rat	9460082
enoyl-[acyl-carrier-protein] reductase (NADPH, B-specific)	1.3.1.10	Rat	17095231

enoyl-[acyl-carrier-protein] reductase (NADPH, B-specific)	1.3.1.10	Rat	8075395
dihydropyrimidine dehydrogenase (NADP+)	1.3.1.2	Rat	10348793
dihydropyrimidine dehydrogenase (NADP+)	1.3.1.2	Rat	10473079
dihydropyrimidine dehydrogenase (NADP+)	1.3.1.2	Rat	10848981
dihydropyrimidine dehydrogenase (NADP+)	1.3.1.2	Rat	10853015
dihydropyrimidine dehydrogenase (NADP+)	1.3.1.2	Rat	10887632
dihydropyrimidine dehydrogenase (NADP+)	1.3.1.2	Rat	10897217
dihydropyrimidine dehydrogenase (NADP+)	1.3.1.2	Rat	11060767
dihydropyrimidine dehydrogenase (NADP+)	1.3.1.2	Rat	11098485
dihydropyrimidine dehydrogenase (NADP+)	1.3.1.2	Rat	11219978
dihydropyrimidine dehydrogenase (NADP+)	1.3.1.2	Rat	11267945
dihydropyrimidine dehydrogenase (NADP+)	1.3.1.2	Rat	11334264
dihydropyrimidine dehydrogenase (NADP+)	1.3.1.2	Rat	11376565
dihydropyrimidine dehydrogenase (NADP+)	1.3.1.2	Rat	11445849
dihydropyrimidine dehydrogenase (NADP+)	1.3.1.2	Rat	11555593
dihydropyrimidine dehydrogenase (NADP+)	1.3.1.2	Rat	11555601
dihydropyrimidine dehydrogenase (NADP+)	1.3.1.2	Rat	11697836
dihydropyrimidine dehydrogenase (NADP+)	1.3.1.2	Rat	11862480
dihydropyrimidine dehydrogenase (NADP+)	1.3.1.2	Rat	11865631
dihydropyrimidine dehydrogenase (NADP+)	1.3.1.2	Rat	11936689
dihydropyrimidine dehydrogenase (NADP+)	1.3.1.2	Rat	11962674
dihydropyrimidine dehydrogenase (NADP+)	1.3.1.2	Rat	12084458
dihydropyrimidine dehydrogenase (NADP+)	1.3.1.2	Rat	12187768
dihydropyrimidine dehydrogenase (NADP+)	1.3.1.2	Rat	12469154
dihydropyrimidine dehydrogenase (NADP+)	1.3.1.2	Rat	12527935
dihydropyrimidine dehydrogenase (NADP+)	1.3.1.2	Rat	12616366
dihydropyrimidine dehydrogenase (NADP+)	1.3.1.2	Rat	12820455
dihydropyrimidine dehydrogenase (NADP+)	1.3.1.2	Rat	12885330
dihydropyrimidine dehydrogenase (NADP+)	1.3.1.2	Rat	12967482
dihydropyrimidine dehydrogenase (NADP+)	1.3.1.2	Rat	14689231
dihydropyrimidine dehydrogenase (NADP+)	1.3.1.2	Rat	14744810
dihydropyrimidine dehydrogenase (NADP+)	1.3.1.2	Rat	15025949
dihydropyrimidine dehydrogenase (NADP+)	1.3.1.2	Rat	15045945
dihydropyrimidine dehydrogenase (NADP+)	1.3.1.2	Rat	15069534
dihydropyrimidine dehydrogenase (NADP+)	1.3.1.2	Rat	15134221
dihydropyrimidine dehydrogenase (NADP+)	1.3.1.2	Rat	15222105
dihydropyrimidine dehydrogenase (NADP+)	1.3.1.2	Rat	1581906
dihydropyrimidine dehydrogenase (NADP+)	1.3.1.2	Rat	15944938
dihydropyrimidine dehydrogenase (NADP+)	1.3.1.2	Rat	16163233
dihydropyrimidine dehydrogenase (NADP+)	1.3.1.2	Rat	1629785
dihydropyrimidine dehydrogenase (NADP+)	1.3.1.2	Rat	16556484
dihydropyrimidine dehydrogenase (NADP+)	1.3.1.2	Rat	16761622

dihydropyrimidine dehydrogenase (NADP+)	1.3.1.2	Rat	16786143
dihydropyrimidine dehydrogenase (NADP+)	1.3.1.2	Rat	16912518
dihydropyrimidine dehydrogenase (NADP+)	1.3.1.2	Rat	17046731
dihydropyrimidine dehydrogenase (NADP+)	1.3.1.2	Rat	2060083
dihydropyrimidine dehydrogenase (NADP+)	1.3.1.2	Rat	3202908
dihydropyrimidine dehydrogenase (NADP+)	1.3.1.2	Rat	7602356
dihydropyrimidine dehydrogenase (NADP+)	1.3.1.2	Rat	7825962
dihydropyrimidine dehydrogenase (NADP+)	1.3.1.2	Rat	8093030
dihydropyrimidine dehydrogenase (NADP+)	1.3.1.2	Rat	8097697
dihydropyrimidine dehydrogenase (NADP+)	1.3.1.2	Rat	8504424
dihydropyrimidine dehydrogenase (NADP+)	1.3.1.2	Rat	8878781
dihydropyrimidine dehydrogenase (NADP+)	1.3.1.2	Rat	8895681
dihydropyrimidine dehydrogenase (NADP+)	1.3.1.2	Rat	8950202
dihydropyrimidine dehydrogenase (NADP+)	1.3.1.2	Rat	9029059
dihydropyrimidine dehydrogenase (NADP+)	1.3.1.2	Rat	9056186
dihydropyrimidine dehydrogenase (NADP+)	1.3.1.2	Rat	9152608
dihydropyrimidine dehydrogenase (NADP+)	1.3.1.2	Rat	9182832
dihydropyrimidine dehydrogenase (NADP+)	1.3.1.2	Rat	9264323
dihydropyrimidine dehydrogenase (NADP+)	1.3.1.2	Rat	9280881
dihydropyrimidine dehydrogenase (NADP+)	1.3.1.2	Rat	9414600
dihydropyrimidine dehydrogenase (NADP+)	1.3.1.2	Rat	9464498
dihydropyrimidine dehydrogenase (NADP+)	1.3.1.2	Rat	9597696
dihydropyrimidine dehydrogenase (NADP+)	1.3.1.2	Rat	9808711
dihydropyrimidine dehydrogenase (NADP+)	1.3.1.2	Rat	9819714
dihydropyrimidine dehydrogenase (NADP+)	1.3.1.2	Rat	9914783
dihydroorotate oxidase	1.3.3.1	Rat	10348793
dihydroorotate oxidase	1.3.3.1	Rat	10473079
dihydroorotate oxidase	1.3.3.1	Rat	10848981
dihydroorotate oxidase	1.3.3.1	Rat	10853015
dihydroorotate oxidase	1.3.3.1	Rat	10878295
dihydroorotate oxidase	1.3.3.1	Rat	10887632
dihydroorotate oxidase	1.3.3.1	Rat	11053058
dihydroorotate oxidase	1.3.3.1	Rat	11060767
dihydroorotate oxidase	1.3.3.1	Rat	11098485
dihydroorotate oxidase	1.3.3.1	Rat	11219978
dihydroorotate oxidase	1.3.3.1	Rat	11267945
dihydroorotate oxidase	1.3.3.1	Rat	11334264
dihydroorotate oxidase	1.3.3.1	Rat	11376565
dihydroorotate oxidase	1.3.3.1	Rat	11522581
dihydroorotate oxidase	1.3.3.1	Rat	11555601
dihydroorotate oxidase	1.3.3.1	Rat	11697836
dihydroorotate oxidase	1.3.3.1	Rat	11796730
dihydroorotate oxidase	1.3.3.1	Rat	11862480

dihydroorotate oxidase	1.3.3.1	Rat	11865631
dihydroorotate oxidase	1.3.3.1	Rat	11936689
dihydroorotate oxidase	1.3.3.1	Rat	11962674
dihydroorotate oxidase	1.3.3.1	Rat	12084458
dihydroorotate oxidase	1.3.3.1	Rat	12187768
dihydroorotate oxidase	1.3.3.1	Rat	12469154
dihydroorotate oxidase	1.3.3.1	Rat	12527935
dihydroorotate oxidase	1.3.3.1	Rat	12634225
dihydroorotate oxidase	1.3.3.1	Rat	12820455
dihydroorotate oxidase	1.3.3.1	Rat	12885330
dihydroorotate oxidase	1.3.3.1	Rat	12967482
dihydroorotate oxidase	1.3.3.1	Rat	14689231
dihydroorotate oxidase	1.3.3.1	Rat	14744810
dihydroorotate oxidase	1.3.3.1	Rat	15025949
dihydroorotate oxidase	1.3.3.1	Rat	15045945
dihydroorotate oxidase	1.3.3.1	Rat	15069534
dihydroorotate oxidase	1.3.3.1	Rat	15134221
dihydroorotate oxidase	1.3.3.1	Rat	15222105
dihydroorotate oxidase	1.3.3.1	Rat	15450176
dihydroorotate oxidase	1.3.3.1	Rat	15944938
dihydroorotate oxidase	1.3.3.1	Rat	16163233
dihydroorotate oxidase	1.3.3.1	Rat	1629785
dihydroorotate oxidase	1.3.3.1	Rat	16556484
dihydroorotate oxidase	1.3.3.1	Rat	17046731
dihydroorotate oxidase	1.3.3.1	Rat	2060083
dihydroorotate oxidase	1.3.3.1	Rat	3202908
dihydroorotate oxidase	1.3.3.1	Rat	6761622
dihydroorotate oxidase	1.3.3.1	Rat	7602356
dihydroorotate oxidase	1.3.3.1	Rat	7825962
dihydroorotate oxidase	1.3.3.1	Rat	8093030
dihydroorotate oxidase	1.3.3.1	Rat	8097697
dihydroorotate oxidase	1.3.3.1	Rat	8878781
dihydroorotate oxidase	1.3.3.1	Rat	8895681
dihydroorotate oxidase	1.3.3.1	Rat	8950202
dihydroorotate oxidase	1.3.3.1	Rat	9056186
dihydroorotate oxidase	1.3.3.1	Rat	9182832
dihydroorotate oxidase	1.3.3.1	Rat	9280881
dihydroorotate oxidase	1.3.3.1	Rat	9464498
dihydroorotate oxidase	1.3.3.1	Rat	9597696
dihydroorotate oxidase	1.3.3.1	Rat	9636062
dihydroorotate oxidase	1.3.3.1	Rat	9819714
dihydroorotate oxidase	1.3.3.1	Rat	9860876
dihydroorotate oxidase	1.3.3.1	Rat	9914783

coproporphyrinogen oxidase	1.3.3.3	Rat	10787385
coproporphyrinogen oxidase	1.3.3.3	Rat	11368326
coproporphyrinogen oxidase	1.3.3.3	Rat	16567402
acyl-CoA oxidase	1.3.3.6	Rat	10318668
acyl-CoA oxidase	1.3.3.6	Rat	11156684
acyl-CoA oxidase	1.3.3.6	Rat	11330046
acyl-CoA oxidase	1.3.3.6	Rat	12538078
acyl-CoA oxidase	1.3.3.6	Rat	12758125
acyl-CoA oxidase	1.3.3.6	Rat	14500732
acyl-CoA oxidase	1.3.3.6	Rat	15805059
acyl-CoA oxidase	1.3.3.6	Rat	1989516
acyl-CoA oxidase	1.3.3.6	Rat	2049482
acyl-CoA oxidase	1.3.3.6	Rat	2811611
acyl-CoA oxidase	1.3.3.6	Rat	3367697
acyl-CoA oxidase	1.3.3.6	Rat	3732222
acyl-CoA oxidase	1.3.3.6	Rat	518563
acyl-CoA oxidase	1.3.3.6	Rat	6240978
acyl-CoA oxidase	1.3.3.6	Rat	6540549
acyl-CoA oxidase	1.3.3.6	Rat	6541949
acyl-CoA oxidase	1.3.3.6	Rat	7860752
acyl-CoA oxidase	1.3.3.6	Rat	7867664
acyl-CoA oxidase	1.3.3.6	Rat	8662598
acyl-CoA oxidase	1.3.3.6	Rat	8784738
acyl-CoA oxidase	1.3.3.6	Rat	8798738
acyl-CoA oxidase	1.3.3.6	Rat	8895731
long-chain-acyl-CoA dehydrogenase	1.3.99.13	Rat	12716879
long-chain-acyl-CoA dehydrogenase	1.3.99.13	Rat	15535801
long-chain-acyl-CoA dehydrogenase	1.3.99.13	Rat	15850553
long-chain-acyl-CoA dehydrogenase	1.3.99.13	Rat	1637289
long-chain-acyl-CoA dehydrogenase	1.3.99.13	Rat	1744086
long-chain-acyl-CoA dehydrogenase	1.3.99.13	Rat	2914148
long-chain-acyl-CoA dehydrogenase	1.3.99.13	Rat	3967008
long-chain-acyl-CoA dehydrogenase	1.3.99.13	Rat	7551821
long-chain-acyl-CoA dehydrogenase	1.3.99.13	Rat	8034667
long-chain-acyl-CoA dehydrogenase	1.3.99.13	Rat	8798738
long-chain-acyl-CoA dehydrogenase	1.3.99.13	Rat	8941110
long-chain-acyl-CoA dehydrogenase	1.3.99.13	Rat	9177236
long-chain-acyl-CoA dehydrogenase	1.3.99.13	Rat	9973285
butyryl-CoA dehydrogenase	1.3.99.2	Rat	12892042
butyryl-CoA dehydrogenase	1.3.99.2	Rat	15358373
butyryl-CoA dehydrogenase	1.3.99.2	Rat	15850406
butyryl-CoA dehydrogenase	1.3.99.2	Rat	16297616
butyryl-CoA dehydrogenase	1.3.99.2	Rat	8941110

butyryl-CoA dehydrogenase	1.3.99.2	Rat	9177236
butyryl-CoA dehydrogenase	1.3.99.2	Rat	9271417
acyl-CoA dehydrogenase	1.3.99.3	Rat	10832093
acyl-CoA dehydrogenase	1.3.99.3	Rat	12359260
acyl-CoA dehydrogenase	1.3.99.3	Rat	15358373
acyl-CoA dehydrogenase	1.3.99.3	Rat	15850406
acyl-CoA dehydrogenase	1.3.99.3	Rat	9177236
acyl-CoA dehydrogenase	1.3.99.3	Rat	9484241
3-oxo-5alpha-steroid 4-dehydrogenase	1.3.99.5	Rat	11408363
3-oxo-5alpha-steroid 4-dehydrogenase	1.3.99.5	Rat	12746845
3-oxo-5alpha-steroid 4-dehydrogenase	1.3.99.5	Rat	16174723
3-oxo-5alpha-steroid 4-dehydrogenase	1.3.99.5	Rat	6523531
3-oxo-5alpha-steroid 4-dehydrogenase	1.3.99.5	Rat	7588289
3-oxo-5alpha-steroid 4-dehydrogenase	1.3.99.5	Rat	9078395
glutamate dehydrogenase [NAD(P)+]	1.4.1.3	Rat	17507377
glutamate dehydrogenase [NAD(P)+]	1.4.1.3	Rat	3139028
glutamate dehydrogenase [NAD(P)+]	1.4.1.3	Rat	3449598
glutamate dehydrogenase [NAD(P)+]	1.4.1.3	Rat	7470041
glutamate dehydrogenase [NAD(P)+]	1.4.1.3	Rat	8122033
pyridoxal 5'-phosphate synthase	1.4.3.5	Rat	6822512
pyridoxal 5'-phosphate synthase	1.4.3.5	Rat	9601034
methylenetetrahydrofolate dehydrogenase (NAD+)	1.5.1.15	Rat	718836
6,7-dihydropteridine reductase	1.5.1.34	Rat	2913929
6,7-dihydropteridine reductase	1.5.1.34	Rat	6797415
formyltetrahydrofolate dehydrogenase	1.5.1.6	Rat	9359603
proline dehydrogenase	1.5.99.8	Rat	11788754
proline dehydrogenase	1.5.99.8	Rat	12602867
glutathione-disulfide reductase	1.8.1.7	Rat	11133045
glutathione-disulfide reductase	1.8.1.7	Rat	12204336
glutathione-disulfide reductase	1.8.1.7	Rat	12453665
glutathione-disulfide reductase	1.8.1.7	Rat	1605642
glutathione-disulfide reductase	1.8.1.7	Rat	2848577
glutathione-disulfide reductase	1.8.1.7	Rat	3698652
glutathione-disulfide reductase	1.8.1.7	Rat	3963383
glutathione-disulfide reductase	1.8.1.7	Rat	8843715
glutathione-disulfide reductase	1.8.1.7	Rat	9350472
cytochrome-c oxidase	1.9.3.1	Rat	10490029
cytochrome-c oxidase	1.9.3.1	Rat	11988227
cytochrome-c oxidase	1.9.3.1	Rat	12059041
cytochrome-c oxidase	1.9.3.1	Rat	12145150
cytochrome-c oxidase	1.9.3.1	Rat	12874793
cytochrome-c oxidase	1.9.3.1	Rat	1315683

cytochrome-c oxidase	1.9.3.1	Rat	1450614
cytochrome-c oxidase	1.9.3.1	Rat	15504366
cytochrome-c oxidase	1.9.3.1	Rat	15708625
cytochrome-c oxidase	1.9.3.1	Rat	16704969
cytochrome-c oxidase	1.9.3.1	Rat	167928
cytochrome-c oxidase	1.9.3.1	Rat	174553
cytochrome-c oxidase	1.9.3.1	Rat	194851
cytochrome-c oxidase	1.9.3.1	Rat	206437
cytochrome-c oxidase	1.9.3.1	Rat	2822680
cytochrome-c oxidase	1.9.3.1	Rat	2849368
cytochrome-c oxidase	1.9.3.1	Rat	2854388
cytochrome-c oxidase	1.9.3.1	Rat	3000820
cytochrome-c oxidase	1.9.3.1	Rat	3002436
cytochrome-c oxidase	1.9.3.1	Rat	356174
cytochrome-c oxidase	1.9.3.1	Rat	3569141
cytochrome-c oxidase	1.9.3.1	Rat	3924042
cytochrome-c oxidase	1.9.3.1	Rat	6091751
cytochrome-c oxidase	1.9.3.1	Rat	6254961
cytochrome-c oxidase	1.9.3.1	Rat	6268138
cytochrome-c oxidase	1.9.3.1	Rat	6286610
cytochrome-c oxidase	1.9.3.1	Rat	6320180
cytochrome-c oxidase	1.9.3.1	Rat	7356983
cytochrome-c oxidase	1.9.3.1	Rat	8013452
cytochrome-c oxidase	1.9.3.1	Rat	8386021
phosphatidylethanolamine N-methyltransferase	2.1.1.17	Rat	8207329
phosphatidylethanolamine N-methyltransferase	2.1.1.17	Rat	9380436
thymidylate synthase	2.1.1.45	Rat	11358693
thymidylate synthase	2.1.1.45	Rat	12412165
thymidylate synthase	2.1.1.45	Rat	12457437
thymidylate synthase	2.1.1.45	Rat	12470718
thymidylate synthase	2.1.1.45	Rat	12544347
thymidylate synthase	2.1.1.45	Rat	14578129
thymidylate synthase	2.1.1.45	Rat	14648018
thymidylate synthase	2.1.1.45	Rat	14689231
thymidylate synthase	2.1.1.45	Rat	15025949
thymidylate synthase	2.1.1.45	Rat	15134221
thymidylate synthase	2.1.1.45	Rat	15598787
thymidylate synthase	2.1.1.45	Rat	16077970
thymidylate synthase	2.1.1.45	Rat	16540728
thymidylate synthase	2.1.1.45	Rat	16617381
thymidylate synthase	2.1.1.45	Rat	3709927

thymidylate synthase	2.1.1.45	Rat	7602356
thymidylate synthase	2.1.1.45	Rat	8805515
phosphoribosylaminoimidazolecarboxamide formyltransferase	2.1.2.3	Rat	6335666
ornithine carbamoyltransferase	2.1.3.3	Rat	1505922
ornithine carbamoyltransferase	2.1.3.3	Rat	205703
ornithine carbamoyltransferase	2.1.3.3	Rat	2667139
ornithine carbamoyltransferase	2.1.3.3	Rat	2667140
ornithine carbamoyltransferase	2.1.3.3	Rat	2667141
ornithine carbamoyltransferase	2.1.3.3	Rat	8019156
ornithine carbamoyltransferase	2.1.3.3	Rat	8168544
ornithine carbamoyltransferase	2.1.3.3	Rat	9501170
ornithine carbamoyltransferase	2.1.3.3	Rat	9540805
glycine amidinotransferase	2.1.4.1	Rat	7419715
transketolase	2.2.1.1	Rat	10975072
transketolase	2.2.1.1	Rat	11072071
transketolase	2.2.1.1	Rat	16354724
transketolase	2.2.1.1	Rat	1939098
transketolase	2.2.1.1	Rat	9924800
transaldolase	2.2.1.2	Rat	11390181
transaldolase	2.2.1.2	Rat	12359249
transaldolase	2.2.1.2	Rat	15263091
transaldolase	2.2.1.2	Rat	16092052
transaldolase	2.2.1.2	Rat	17046540
transaldolase	2.2.1.2	Rat	9565623
transaldolase	2.2.1.2	Rat	9973403
dihydrolipoyllysine-residue acetyltransferase	2.3.1.12	Rat	3840997
glycerol-3-phosphate O-acyltransferase	2.3.1.15	Rat	11003606
glycerol-3-phosphate O-acyltransferase	2.3.1.15	Rat	11284717
glycerol-3-phosphate O-acyltransferase	2.3.1.15	Rat	12464581
glycerol-3-phosphate O-acyltransferase	2.3.1.15	Rat	12730219
glycerol-3-phosphate O-acyltransferase	2.3.1.15	Rat	14724270
glycerol-3-phosphate O-acyltransferase	2.3.1.15	Rat	15102885
glycerol-3-phosphate O-acyltransferase	2.3.1.15	Rat	16234267
glycerol-3-phosphate O-acyltransferase	2.3.1.15	Rat	16431156
glycerol-3-phosphate O-acyltransferase	2.3.1.15	Rat	16507761
glycerol-3-phosphate O-acyltransferase	2.3.1.15	Rat	2303421
glycerol-3-phosphate O-acyltransferase	2.3.1.15	Rat	2559137
glycerol-3-phosphate O-acyltransferase	2.3.1.15	Rat	2674663
glycerol-3-phosphate O-acyltransferase	2.3.1.15	Rat	2737209
glycerol-3-phosphate O-acyltransferase	2.3.1.15	Rat	2830105
glycerol-3-phosphate O-acyltransferase	2.3.1.15	Rat	3103689
glycerol-3-phosphate O-acyltransferase	2.3.1.15	Rat	3311005

glycerol-3-phosphate O-acyltransferase	2.3.1.15	Rat	3334861
glycerol-3-phosphate O-acyltransferase	2.3.1.15	Rat	6497849
glycerol-3-phosphate O-acyltransferase	2.3.1.15	Rat	813635
glycerol-3-phosphate O-acyltransferase	2.3.1.15	Rat	8387510
glycerol-3-phosphate O-acyltransferase	2.3.1.15	Rat	9393688
acetyl-CoA C-acyltransferase	2.3.1.16	Rat	3967008
acetyl-CoA C-acyltransferase	2.3.1.16	Rat	7068598
acetyl-CoA C-acyltransferase	2.3.1.16	Rat	7374368
carnitine O-palmitoyltransferase	2.3.1.21	Rat	10417344
carnitine O-palmitoyltransferase	2.3.1.21	Rat	10709666
carnitine O-palmitoyltransferase	2.3.1.21	Rat	10956641
carnitine O-palmitoyltransferase	2.3.1.21	Rat	11356163
carnitine O-palmitoyltransferase	2.3.1.21	Rat	11371554
carnitine O-palmitoyltransferase	2.3.1.21	Rat	11463952
carnitine O-palmitoyltransferase	2.3.1.21	Rat	12015320
carnitine O-palmitoyltransferase	2.3.1.21	Rat	12359092
carnitine O-palmitoyltransferase	2.3.1.21	Rat	12574149
carnitine O-palmitoyltransferase	2.3.1.21	Rat	12619873
carnitine O-palmitoyltransferase	2.3.1.21	Rat	12761301
carnitine O-palmitoyltransferase	2.3.1.21	Rat	14751860
carnitine O-palmitoyltransferase	2.3.1.21	Rat	15044358
carnitine O-palmitoyltransferase	2.3.1.21	Rat	15247243
carnitine O-palmitoyltransferase	2.3.1.21	Rat	15539300
carnitine O-palmitoyltransferase	2.3.1.21	Rat	15590999
carnitine O-palmitoyltransferase	2.3.1.21	Rat	15623825
carnitine O-palmitoyltransferase	2.3.1.21	Rat	1563551
carnitine O-palmitoyltransferase	2.3.1.21	Rat	15878185
carnitine O-palmitoyltransferase	2.3.1.21	Rat	15919095
carnitine O-palmitoyltransferase	2.3.1.21	Rat	16177188
carnitine O-palmitoyltransferase	2.3.1.21	Rat	16225603
carnitine O-palmitoyltransferase	2.3.1.21	Rat	16509570
carnitine O-palmitoyltransferase	2.3.1.21	Rat	16528409
carnitine O-palmitoyltransferase	2.3.1.21	Rat	16545538
carnitine O-palmitoyltransferase	2.3.1.21	Rat	16584169
carnitine O-palmitoyltransferase	2.3.1.21	Rat	16763001
carnitine O-palmitoyltransferase	2.3.1.21	Rat	17021367
carnitine O-palmitoyltransferase	2.3.1.21	Rat	17089095
carnitine O-palmitoyltransferase	2.3.1.21	Rat	1979695
carnitine O-palmitoyltransferase	2.3.1.21	Rat	2166437
carnitine O-palmitoyltransferase	2.3.1.21	Rat	2914148
carnitine O-palmitoyltransferase	2.3.1.21	Rat	3090894
carnitine O-palmitoyltransferase	2.3.1.21	Rat	3709811
carnitine O-palmitoyltransferase	2.3.1.21	Rat	3800962

carnitine O-palmitoyltransferase	2.3.1.21	Rat	3834060
carnitine O-palmitoyltransferase	2.3.1.21	Rat	6105152
carnitine O-palmitoyltransferase	2.3.1.21	Rat	670195
carnitine O-palmitoyltransferase	2.3.1.21	Rat	7334004
carnitine O-palmitoyltransferase	2.3.1.21	Rat	7734439
carnitine O-palmitoyltransferase	2.3.1.21	Rat	8141397
carnitine O-palmitoyltransferase	2.3.1.21	Rat	8335590
carnitine O-palmitoyltransferase	2.3.1.21	Rat	8479178
carnitine O-palmitoyltransferase	2.3.1.21	Rat	8589632
carnitine O-palmitoyltransferase	2.3.1.21	Rat	8857518
carnitine O-palmitoyltransferase	2.3.1.21	Rat	9136891
carnitine O-palmitoyltransferase	2.3.1.21	Rat	9447322
carnitine O-palmitoyltransferase	2.3.1.21	Rat	9545636
carnitine O-palmitoyltransferase	2.3.1.21	Rat	9792707
carnitine O-palmitoyltransferase	2.3.1.21	Rat	9989283
sterol O-acyltransferase	2.3.1.26	Rat	10593897
sterol O-acyltransferase	2.3.1.26	Rat	10656290
sterol O-acyltransferase	2.3.1.26	Rat	11888294
sterol O-acyltransferase	2.3.1.26	Rat	12787409
sterol O-acyltransferase	2.3.1.26	Rat	1450216
sterol O-acyltransferase	2.3.1.26	Rat	2885178
sterol O-acyltransferase	2.3.1.26	Rat	3593752
sterol O-acyltransferase	2.3.1.26	Rat	3719008
sterol O-acyltransferase	2.3.1.26	Rat	3766727
sterol O-acyltransferase	2.3.1.26	Rat	3812206
sterol O-acyltransferase	2.3.1.26	Rat	3926761
sterol O-acyltransferase	2.3.1.26	Rat	4056052
sterol O-acyltransferase	2.3.1.26	Rat	6347025
sterol O-acyltransferase	2.3.1.26	Rat	8258956
sterol O-acyltransferase	2.3.1.26	Rat	8466946
sterol O-acyltransferase	2.3.1.26	Rat	8820097
sterol O-acyltransferase	2.3.1.26	Rat	9857049
5-aminolevulinate synthase	2.3.1.37	Rat	10634305
5-aminolevulinate synthase	2.3.1.37	Rat	10727444
5-aminolevulinate synthase	2.3.1.37	Rat	10729988
5-aminolevulinate synthase	2.3.1.37	Rat	10787385
5-aminolevulinate synthase	2.3.1.37	Rat	10825473
5-aminolevulinate synthase	2.3.1.37	Rat	11202048
5-aminolevulinate synthase	2.3.1.37	Rat	11202050
5-aminolevulinate synthase	2.3.1.37	Rat	11368326
5-aminolevulinate synthase	2.3.1.37	Rat	12121995
5-aminolevulinate synthase	2.3.1.37	Rat	12393745
5-aminolevulinate synthase	2.3.1.37	Rat	12469218

5-aminolevulinate synthase	2.3.1.37	Rat	12627002
5-aminolevulinate synthase	2.3.1.37	Rat	126586
5-aminolevulinate synthase	2.3.1.37	Rat	12881517
5-aminolevulinate synthase	2.3.1.37	Rat	14643887
5-aminolevulinate synthase	2.3.1.37	Rat	1511083
5-aminolevulinate synthase	2.3.1.37	Rat	15178759
5-aminolevulinate synthase	2.3.1.37	Rat	15259603
5-aminolevulinate synthase	2.3.1.37	Rat	1526942
5-aminolevulinate synthase	2.3.1.37	Rat	15547665
5-aminolevulinate synthase	2.3.1.37	Rat	15797241
5-aminolevulinate synthase	2.3.1.37	Rat	15972158
5-aminolevulinate synthase	2.3.1.37	Rat	16122419
5-aminolevulinate synthase	2.3.1.37	Rat	16181105
5-aminolevulinate synthase	2.3.1.37	Rat	16567402
5-aminolevulinate synthase	2.3.1.37	Rat	16846079
5-aminolevulinate synthase	2.3.1.37	Rat	1688552
5-aminolevulinate synthase	2.3.1.37	Rat	16892088
5-aminolevulinate synthase	2.3.1.37	Rat	16904069
5-aminolevulinate synthase	2.3.1.37	Rat	1954232
5-aminolevulinate synthase	2.3.1.37	Rat	1959865
5-aminolevulinate synthase	2.3.1.37	Rat	2050126
5-aminolevulinate synthase	2.3.1.37	Rat	2241158
5-aminolevulinate synthase	2.3.1.37	Rat	2317819
5-aminolevulinate synthase	2.3.1.37	Rat	266710
5-aminolevulinate synthase	2.3.1.37	Rat	3009001
5-aminolevulinate synthase	2.3.1.37	Rat	3094379
5-aminolevulinate synthase	2.3.1.37	Rat	3359971
5-aminolevulinate synthase	2.3.1.37	Rat	3755290
5-aminolevulinate synthase	2.3.1.37	Rat	3840094
5-aminolevulinate synthase	2.3.1.37	Rat	3966797
5-aminolevulinate synthase	2.3.1.37	Rat	6092369
5-aminolevulinate synthase	2.3.1.37	Rat	6213232
5-aminolevulinate synthase	2.3.1.37	Rat	6547609
5-aminolevulinate synthase	2.3.1.37	Rat	6824732
5-aminolevulinate synthase	2.3.1.37	Rat	6873612
5-aminolevulinate synthase	2.3.1.37	Rat	6954546
5-aminolevulinate synthase	2.3.1.37	Rat	7093306
5-aminolevulinate synthase	2.3.1.37	Rat	7592563
5-aminolevulinate synthase	2.3.1.37	Rat	8076930
5-aminolevulinate synthase	2.3.1.37	Rat	818637
5-aminolevulinate synthase	2.3.1.37	Rat	8385933
5-aminolevulinate synthase	2.3.1.37	Rat	8413301
5-aminolevulinate synthase	2.3.1.37	Rat	9169013

5-aminolevulinate synthase	2.3.1.37	Rat	9173682
5-aminolevulinate synthase	2.3.1.37	Rat	9806796
5-aminolevulinate synthase	2.3.1.37	Rat	9879810
arylamine N-acetyltransferase	2.3.1.5	Rat	10100739
arylamine N-acetyltransferase	2.3.1.5	Rat	10467435
arylamine N-acetyltransferase	2.3.1.5	Rat	10471401
arylamine N-acetyltransferase	2.3.1.5	Rat	10721063
arylamine N-acetyltransferase	2.3.1.5	Rat	10843785
arylamine N-acetyltransferase	2.3.1.5	Rat	10931207
arylamine N-acetyltransferase	2.3.1.5	Rat	11122368
arylamine N-acetyltransferase	2.3.1.5	Rat	12465141
arylamine N-acetyltransferase	2.3.1.5	Rat	12734775
arylamine N-acetyltransferase	2.3.1.5	Rat	12736370
arylamine N-acetyltransferase	2.3.1.5	Rat	12736803
arylamine N-acetyltransferase	2.3.1.5	Rat	14550904
arylamine N-acetyltransferase	2.3.1.5	Rat	14578935
arylamine N-acetyltransferase	2.3.1.5	Rat	15219412
arylamine N-acetyltransferase	2.3.1.5	Rat	15228600
arylamine N-acetyltransferase	2.3.1.5	Rat	15782814
arylamine N-acetyltransferase	2.3.1.5	Rat	15978059
arylamine N-acetyltransferase	2.3.1.5	Rat	15978063
arylamine N-acetyltransferase	2.3.1.5	Rat	16095816
arylamine N-acetyltransferase	2.3.1.5	Rat	1715400
arylamine N-acetyltransferase	2.3.1.5	Rat	1806289
arylamine N-acetyltransferase	2.3.1.5	Rat	1829510
arylamine N-acetyltransferase	2.3.1.5	Rat	1974286
arylamine N-acetyltransferase	2.3.1.5	Rat	1976757
arylamine N-acetyltransferase	2.3.1.5	Rat	1977416
arylamine N-acetyltransferase	2.3.1.5	Rat	2054611
arylamine N-acetyltransferase	2.3.1.5	Rat	2133350
arylamine N-acetyltransferase	2.3.1.5	Rat	2167223
arylamine N-acetyltransferase	2.3.1.5	Rat	3179707
arylamine N-acetyltransferase	2.3.1.5	Rat	3574290
arylamine N-acetyltransferase	2.3.1.5	Rat	3702592
arylamine N-acetyltransferase	2.3.1.5	Rat	7552254
arylamine N-acetyltransferase	2.3.1.5	Rat	7686576
arylamine N-acetyltransferase	2.3.1.5	Rat	7741268
arylamine N-acetyltransferase	2.3.1.5	Rat	8098713
arylamine N-acetyltransferase	2.3.1.5	Rat	8106958
arylamine N-acetyltransferase	2.3.1.5	Rat	8176952
arylamine N-acetyltransferase	2.3.1.5	Rat	8420625
arylamine N-acetyltransferase	2.3.1.5	Rat	8524412
arylamine N-acetyltransferase	2.3.1.5	Rat	8550820

arylamine N-acetyltransferase	2.3.1.5	Rat	8599175
arylamine N-acetyltransferase	2.3.1.5	Rat	8888105
arylamine N-acetyltransferase	2.3.1.5	Rat	8930697
arylamine N-acetyltransferase	2.3.1.5	Rat	8943074
arylamine N-acetyltransferase	2.3.1.5	Rat	9181516
arylamine N-acetyltransferase	2.3.1.5	Rat	9238850
arylamine N-acetyltransferase	2.3.1.5	Rat	9437760
arylamine N-acetyltransferase	2.3.1.5	Rat	9566052
arylamine N-acetyltransferase	2.3.1.5	Rat	9597751
arylamine N-acetyltransferase	2.3.1.5	Rat	9669546
arylamine N-acetyltransferase	2.3.1.5	Rat	9811631
arylamine N-acetyltransferase	2.3.1.5	Rat	9949305
fatty-acid synthase	2.3.1.85	Rat	10385596
fatty-acid synthase	2.3.1.85	Rat	11032949
fatty-acid synthase	2.3.1.85	Rat	12131249
fatty-acid synthase	2.3.1.85	Rat	12820377
fatty-acid synthase	2.3.1.85	Rat	14767544
fatty-acid synthase	2.3.1.85	Rat	15302084
fatty-acid synthase	2.3.1.85	Rat	15491158
fatty-acid synthase	2.3.1.85	Rat	15577743
fatty-acid synthase	2.3.1.85	Rat	16007182
fatty-acid synthase	2.3.1.85	Rat	16054091
fatty-acid synthase	2.3.1.85	Rat	16374067
fatty-acid synthase	2.3.1.85	Rat	16582625
fatty-acid synthase	2.3.1.85	Rat	16729974
fatty-acid synthase	2.3.1.85	Rat	7948007
fatty-acid synthase	2.3.1.85	Rat	9510066
fatty-acid synthase	2.3.1.85	Rat	9593836
aralkylamine N-acetyltransferase	2.3.1.87	Rat	10329462
aralkylamine N-acetyltransferase	2.3.1.87	Rat	10451021
aralkylamine N-acetyltransferase	2.3.1.87	Rat	10537047
aralkylamine N-acetyltransferase	2.3.1.87	Rat	10537048
aralkylamine N-acetyltransferase	2.3.1.87	Rat	10537049
aralkylamine N-acetyltransferase	2.3.1.87	Rat	11325593
aralkylamine N-acetyltransferase	2.3.1.87	Rat	11432976
aralkylamine N-acetyltransferase	2.3.1.87	Rat	11506377
aralkylamine N-acetyltransferase	2.3.1.87	Rat	12052171
aralkylamine N-acetyltransferase	2.3.1.87	Rat	12059970
aralkylamine N-acetyltransferase	2.3.1.87	Rat	14759496
aralkylamine N-acetyltransferase	2.3.1.87	Rat	15026119
aralkylamine N-acetyltransferase	2.3.1.87	Rat	15081830
aralkylamine N-acetyltransferase	2.3.1.87	Rat	15519681
aralkylamine N-acetyltransferase	2.3.1.87	Rat	15773915

aralkylamine N-acetyltransferase	2.3.1.87	Rat	15950762
aralkylamine N-acetyltransferase	2.3.1.87	Rat	16024134
aralkylamine N-acetyltransferase	2.3.1.87	Rat	16207298
aralkylamine N-acetyltransferase	2.3.1.87	Rat	16556767
aralkylamine N-acetyltransferase	2.3.1.87	Rat	16604054
aralkylamine N-acetyltransferase	2.3.1.87	Rat	16687310
aralkylamine N-acetyltransferase	2.3.1.87	Rat	16842539
aralkylamine N-acetyltransferase	2.3.1.87	Rat	16842546
aralkylamine N-acetyltransferase	2.3.1.87	Rat	16869299
aralkylamine N-acetyltransferase	2.3.1.87	Rat	16962714
aralkylamine N-acetyltransferase	2.3.1.87	Rat	1705890
aralkylamine N-acetyltransferase	2.3.1.87	Rat	503196
aralkylamine N-acetyltransferase	2.3.1.87	Rat	7498465
aralkylamine N-acetyltransferase	2.3.1.87	Rat	7566441
aralkylamine N-acetyltransferase	2.3.1.87	Rat	8674865
aralkylamine N-acetyltransferase	2.3.1.87	Rat	9605498
aralkylamine N-acetyltransferase	2.3.1.87	Rat	9703021
aralkylamine N-acetyltransferase	2.3.1.87	Rat	9708862
acetyl-CoA C-acetyltransferase	2.3.1.9	Rat	14693556
acetyl-CoA C-acetyltransferase	2.3.1.9	Rat	15135409
acetyl-CoA C-acetyltransferase	2.3.1.9	Rat	15466479
acetyl-CoA C-acetyltransferase	2.3.1.9	Rat	2869784
gamma-glutamyltransferase	2.3.2.2	Rat	11810401
gamma-glutamyltransferase	2.3.2.2	Rat	12030366
gamma-glutamyltransferase	2.3.2.2	Rat	12468440
gamma-glutamyltransferase	2.3.2.2	Rat	12780970
gamma-glutamyltransferase	2.3.2.2	Rat	15006645
gamma-glutamyltransferase	2.3.2.2	Rat	16302185
gamma-glutamyltransferase	2.3.2.2	Rat	2881890
gamma-glutamyltransferase	2.3.2.2	Rat	2903803
gamma-glutamyltransferase	2.3.2.2	Rat	7485380
gamma-glutamyltransferase	2.3.2.2	Rat	8067452
gamma-glutamyltransferase	2.3.2.2	Rat	8564390
gamma-glutamyltransferase	2.3.2.2	Rat	8972486
gamma-glutamyltransferase	2.3.2.2	Rat	9974125
citrate (Si)-synthase	2.3.3.1	Rat	1004246
citrate (Si)-synthase	2.3.3.1	Rat	11842094
citrate (Si)-synthase	2.3.3.1	Rat	11872452
citrate (Si)-synthase	2.3.3.1	Rat	15994367
citrate (Si)-synthase	2.3.3.1	Rat	16269721
citrate (Si)-synthase	2.3.3.1	Rat	3776117
citrate (Si)-synthase	2.3.3.1	Rat	3916224
citrate (Si)-synthase	2.3.3.1	Rat	6799496

citrate (Si)-synthase	2.3.3.1	Rat	8526514
citrate (Si)-synthase	2.3.3.1	Rat	9353808
citrate (Si)-synthase	2.3.3.1	Rat	9554114
hydroxymethylglutaryl-CoA synthase	2.3.3.10	Rat	11160362
hydroxymethylglutaryl-CoA synthase	2.3.3.10	Rat	11485325
hydroxymethylglutaryl-CoA synthase	2.3.3.10	Rat	16101500
hydroxymethylglutaryl-CoA synthase	2.3.3.10	Rat	16864776
hydroxymethylglutaryl-CoA synthase	2.3.3.10	Rat	16962226
hydroxymethylglutaryl-CoA synthase	2.3.3.10	Rat	475
hydroxymethylglutaryl-CoA synthase	2.3.3.10	Rat	7907092
ATP citrate synthase	2.3.3.8	Rat	10759520
ATP citrate synthase	2.3.3.8	Rat	10801800
ATP citrate synthase	2.3.3.8	Rat	12135479
ATP citrate synthase	2.3.3.8	Rat	12957888
ATP citrate synthase	2.3.3.8	Rat	131232
ATP citrate synthase	2.3.3.8	Rat	16226706
ATP citrate synthase	2.3.3.8	Rat	16269773
ATP citrate synthase	2.3.3.8	Rat	16461683
ATP citrate synthase	2.3.3.8	Rat	16988757
ATP citrate synthase	2.3.3.8	Rat	17404227
ATP citrate synthase	2.3.3.8	Rat	2295639
ATP citrate synthase	2.3.3.8	Rat	8832570
ATP citrate synthase	2.3.3.8	Rat	9082912
phosphorylase	2.4.1.1	Rat	10548038
phosphorylase	2.4.1.1	Rat	11391834
phosphorylase	2.4.1.1	Rat	11391835
phosphorylase	2.4.1.1	Rat	11391836
phosphorylase	2.4.1.1	Rat	11391837
phosphorylase	2.4.1.1	Rat	11391838
phosphorylase	2.4.1.1	Rat	11391839
phosphorylase	2.4.1.1	Rat	11391840
phosphorylase	2.4.1.1	Rat	11391841
phosphorylase	2.4.1.1	Rat	11391842
phosphorylase	2.4.1.1	Rat	12769745
phosphorylase	2.4.1.1	Rat	15299833
phosphorylase	2.4.1.1	Rat	15721288
phosphorylase	2.4.1.1	Rat	1691273
phosphorylase	2.4.1.1	Rat	7664039
glycogen(starch) synthase	2.4.1.11	Rat	10067873
glycogen(starch) synthase	2.4.1.11	Rat	10222257
glycogen(starch) synthase	2.4.1.11	Rat	10684630
glycogen(starch) synthase	2.4.1.11	Rat	11181947
glycogen(starch) synthase	2.4.1.11	Rat	11467410

glycogen(starch) synthase	2.4.1.11	Rat	11534633
glycogen(starch) synthase	2.4.1.11	Rat	11834204
glycogen(starch) synthase	2.4.1.11	Rat	11900279
glycogen(starch) synthase	2.4.1.11	Rat	12617691
glycogen(starch) synthase	2.4.1.11	Rat	14570701
glycogen(starch) synthase	2.4.1.11	Rat	15840572
glycogen(starch) synthase	2.4.1.11	Rat	15932409
glycogen(starch) synthase	2.4.1.11	Rat	16101290
glycogen(starch) synthase	2.4.1.11	Rat	1756915
glycogen(starch) synthase	2.4.1.11	Rat	17569761
glycogen(starch) synthase	2.4.1.11	Rat	17698598
glycogen(starch) synthase	2.4.1.11	Rat	1959479
glycogen(starch) synthase	2.4.1.11	Rat	208368
glycogen(starch) synthase	2.4.1.11	Rat	2115296
glycogen(starch) synthase	2.4.1.11	Rat	2154910
glycogen(starch) synthase	2.4.1.11	Rat	219866
glycogen(starch) synthase	2.4.1.11	Rat	227915
glycogen(starch) synthase	2.4.1.11	Rat	2405698
glycogen(starch) synthase	2.4.1.11	Rat	2822414
glycogen(starch) synthase	2.4.1.11	Rat	3032541
glycogen(starch) synthase	2.4.1.11	Rat	3092743
glycogen(starch) synthase	2.4.1.11	Rat	3143265
glycogen(starch) synthase	2.4.1.11	Rat	3930321
glycogen(starch) synthase	2.4.1.11	Rat	6409592
glycogen(starch) synthase	2.4.1.11	Rat	6412593
glycogen(starch) synthase	2.4.1.11	Rat	7010073
glycogen(starch) synthase	2.4.1.11	Rat	7672505
glycogen(starch) synthase	2.4.1.11	Rat	7983805
glycogen(starch) synthase	2.4.1.11	Rat	8226927
glycogen(starch) synthase	2.4.1.11	Rat	8416266
glycogen(starch) synthase	2.4.1.11	Rat	8514767
glycogen(starch) synthase	2.4.1.11	Rat	8514849
glycogen(starch) synthase	2.4.1.11	Rat	8541012
glycogen(starch) synthase	2.4.1.11	Rat	8569754
glycogen(starch) synthase	2.4.1.11	Rat	8591890
glycogen(starch) synthase	2.4.1.11	Rat	8593937
glycogen(starch) synthase	2.4.1.11	Rat	8612539
glycogen(starch) synthase	2.4.1.11	Rat	8645005
glycogen(starch) synthase	2.4.1.11	Rat	8721777
glycogen(starch) synthase	2.4.1.11	Rat	8769349
glycogen(starch) synthase	2.4.1.11	Rat	9126490
glycogen(starch) synthase	2.4.1.11	Rat	9162607
glycogen(starch) synthase	2.4.1.11	Rat	9267990

glycogen(starch) synthase	2.4.1.11	Rat	9389424
glycogen(starch) synthase	2.4.1.11	Rat	9450985
glycogen(starch) synthase	2.4.1.11	Rat	9609122
glycogen(starch) synthase	2.4.1.11	Rat	9712712
alpha-1,6-mannosyl-glycoprotein	2.4.1.143	Rat	11250723
alpha-1,6-mannosyl-glycoprotein	2.4.1.143	Rat	11552947
alpha-1,6-mannosyl-glycoprotein	2.4.1.143	Rat	7841796
alpha-1,6-mannosyl-glycoprotein	2.4.1.143	Rat	9028721
glucuronosyltransferase	2.4.1.17	Rat	10353933
glucuronosyltransferase	2.4.1.17	Rat	10427418
glucuronosyltransferase	2.4.1.17	Rat	15710570
glucuronosyltransferase	2.4.1.17	Rat	16623861
glucuronosyltransferase	2.4.1.17	Rat	4715994
glucuronosyltransferase	2.4.1.17	Rat	7900959
lactosylceramide 1,3-N-acetyl-beta-D-glucosaminyltransferase	2.4.1.206	Rat	2564417
lactose synthase	2.4.1.22	Rat	16664466
lactose synthase	2.4.1.22	Rat	6766957
lactose synthase	2.4.1.22	Rat	7539442
lactose synthase	2.4.1.22	Rat	9387870
amidophosphoribosyltransferase	2.4.2.14	Rat	10675983
amidophosphoribosyltransferase	2.4.2.14	Rat	11158364
amidophosphoribosyltransferase	2.4.2.14	Rat	12930749
amidophosphoribosyltransferase	2.4.2.14	Rat	15266056
amidophosphoribosyltransferase	2.4.2.14	Rat	17434429
amidophosphoribosyltransferase	2.4.2.14	Rat	214373
amidophosphoribosyltransferase	2.4.2.14	Rat	6327016
amidophosphoribosyltransferase	2.4.2.14	Rat	701284
amidophosphoribosyltransferase	2.4.2.14	Rat	7683680
amidophosphoribosyltransferase	2.4.2.14	Rat	8150282
amidophosphoribosyltransferase	2.4.2.14	Rat	8197456
amidophosphoribosyltransferase	2.4.2.14	Rat	8197457
amidophosphoribosyltransferase	2.4.2.14	Rat	8197458
amidophosphoribosyltransferase	2.4.2.14	Rat	8380692
amidophosphoribosyltransferase	2.4.2.14	Rat	8463258
amidophosphoribosyltransferase	2.4.2.14	Rat	8809759
amidophosphoribosyltransferase	2.4.2.14	Rat	8976092
amidophosphoribosyltransferase	2.4.2.14	Rat	9615746
amidophosphoribosyltransferase	2.4.2.14	Rat	9881055
dimethylallyltranstransferase	2.5.1.1	Rat	11442630
dimethylallyltranstransferase	2.5.1.1	Rat	14512521
dimethylallyltranstransferase	2.5.1.1	Rat	15597200
dimethylallyltranstransferase	2.5.1.1	Rat	7697819

dimethylallyltranstransferase	2.5.1.1	Rat	7843406
dimethylallyltranstransferase	2.5.1.1	Rat	8631820
geranyltranstransferase	2.5.1.10	Rat	10484604
geranyltranstransferase	2.5.1.10	Rat	11202437
geranyltranstransferase	2.5.1.10	Rat	15459425
geranyltranstransferase	2.5.1.10	Rat	15605175
geranyltranstransferase	2.5.1.10	Rat	15713990
geranyltranstransferase	2.5.1.10	Rat	15827605
geranyltranstransferase	2.5.1.10	Rat	15827618
geranyltranstransferase	2.5.1.10	Rat	16179378
geranyltranstransferase	2.5.1.10	Rat	16932286
geranyltranstransferase	2.5.1.10	Rat	1779710
geranyltranstransferase	2.5.1.10	Rat	9061016
geranyltranstransferase	2.5.1.10	Rat	9640665
spermidine synthase	2.5.1.16	Rat	16515550
spermidine synthase	2.5.1.16	Rat	2775206
glutathione transferase	2.5.1.18	Rat	12484753
glutathione transferase	2.5.1.18	Rat	15604283
glutathione transferase	2.5.1.18	Rat	15761769
glutathione transferase	2.5.1.18	Rat	16328982
glutathione transferase	2.5.1.18	Rat	17176043
glutathione transferase	2.5.1.18	Rat	17397868
squalene synthase	2.5.1.21	Rat	10649449
squalene synthase	2.5.1.21	Rat	10677224
squalene synthase	2.5.1.21	Rat	12114564
squalene synthase	2.5.1.21	Rat	15356323
squalene synthase	2.5.1.21	Rat	1601846
squalene synthase	2.5.1.21	Rat	17016471
squalene synthase	2.5.1.21	Rat	17531951
squalene synthase	2.5.1.21	Rat	2068081
squalene synthase	2.5.1.21	Rat	7766395
squalene synthase	2.5.1.21	Rat	7843406
squalene synthase	2.5.1.21	Rat	9070296
methionine adenosyltransferase	2.5.1.6	Rat	10415148
methionine adenosyltransferase	2.5.1.6	Rat	1511738
methionine adenosyltransferase	2.5.1.6	Rat	16413417
methionine adenosyltransferase	2.5.1.6	Rat	2764959
methionine adenosyltransferase	2.5.1.6	Rat	7980467
hydroxymethylbilane synthase	2.5.1.61	Rat	10546563
hydroxymethylbilane synthase	2.5.1.61	Rat	10787385
hydroxymethylbilane synthase	2.5.1.61	Rat	11953837
hydroxymethylbilane synthase	2.5.1.61	Rat	14559249
hydroxymethylbilane synthase	2.5.1.61	Rat	1522882

hydroxymethylbilane synthase	2.5.1.61	Rat	16886091
hydroxymethylbilane synthase	2.5.1.61	Rat	4067519
hydroxymethylbilane synthase	2.5.1.61	Rat	7326026
hydroxymethylbilane synthase	2.5.1.61	Rat	7682572
hydroxymethylbilane synthase	2.5.1.61	Rat	8023693
hydroxymethylbilane synthase	2.5.1.61	Rat	9065797
hydroxymethylbilane synthase	2.5.1.61	Rat	9460994
aspartate transaminase	2.6.1.1	Rat	8580353
ornithine aminotransferase	2.6.1.13	Rat	11691635
ornithine aminotransferase	2.6.1.13	Rat	12462748
ornithine aminotransferase	2.6.1.13	Rat	7883744
tyrosine transaminase	2.6.1.5	Rat	1348057
tyrosine transaminase	2.6.1.5	Rat	1526942
tyrosine transaminase	2.6.1.5	Rat	240411
tyrosine transaminase	2.6.1.5	Rat	2870018
tyrosine transaminase	2.6.1.5	Rat	6123525
tyrosine transaminase	2.6.1.5	Rat	9089286
tyrosine transaminase	2.6.1.5	Rat	9228277
hexokinase	2.7.1.1	Rat	11319725
hexokinase	2.7.1.1	Rat	11391834
hexokinase	2.7.1.1	Rat	11828256
hexokinase	2.7.1.1	Rat	12660493
hexokinase	2.7.1.1	Rat	1331693
hexokinase	2.7.1.1	Rat	14672622
hexokinase	2.7.1.1	Rat	15607940
hexokinase	2.7.1.1	Rat	6341787
hexokinase	2.7.1.1	Rat	6363888
hexokinase	2.7.1.1	Rat	6440018
hexokinase	2.7.1.1	Rat	6993859
hexokinase	2.7.1.1	Rat	8027295
hexokinase	2.7.1.1	Rat	9523722
6-phosphofructokinase	2.7.1.11	Rat	10323269
6-phosphofructokinase	2.7.1.11	Rat	10444344
6-phosphofructokinase	2.7.1.11	Rat	10742704
6-phosphofructokinase	2.7.1.11	Rat	10909961
6-phosphofructokinase	2.7.1.11	Rat	10931197
6-phosphofructokinase	2.7.1.11	Rat	1100622
6-phosphofructokinase	2.7.1.11	Rat	1100623
6-phosphofructokinase	2.7.1.11	Rat	11014908
6-phosphofructokinase	2.7.1.11	Rat	11045948
6-phosphofructokinase	2.7.1.11	Rat	11058792
6-phosphofructokinase	2.7.1.11	Rat	11391835
6-phosphofructokinase	2.7.1.11	Rat	11391836

6-phosphofructokinase	2.7.1.11	Rat	11391837
6-phosphofructokinase	2.7.1.11	Rat	11560513
6-phosphofructokinase	2.7.1.11	Rat	12023862
6-phosphofructokinase	2.7.1.11	Rat	12051897
6-phosphofructokinase	2.7.1.11	Rat	12125051
6-phosphofructokinase	2.7.1.11	Rat	12453221
6-phosphofructokinase	2.7.1.11	Rat	131232
6-phosphofructokinase	2.7.1.11	Rat	14585511
6-phosphofructokinase	2.7.1.11	Rat	147929
6-phosphofructokinase	2.7.1.11	Rat	149128
6-phosphofructokinase	2.7.1.11	Rat	15157773
6-phosphofructokinase	2.7.1.11	Rat	15466668
6-phosphofructokinase	2.7.1.11	Rat	15504384
6-phosphofructokinase	2.7.1.11	Rat	156307
6-phosphofructokinase	2.7.1.11	Rat	15991998
6-phosphofructokinase	2.7.1.11	Rat	16088331
6-phosphofructokinase	2.7.1.11	Rat	16103521
6-phosphofructokinase	2.7.1.11	Rat	16115917
6-phosphofructokinase	2.7.1.11	Rat	16346876
6-phosphofructokinase	2.7.1.11	Rat	16377227
6-phosphofructokinase	2.7.1.11	Rat	1658253
6-phosphofructokinase	2.7.1.11	Rat	16593209
6-phosphofructokinase	2.7.1.11	Rat	1825156
6-phosphofructokinase	2.7.1.11	Rat	1828673
6-phosphofructokinase	2.7.1.11	Rat	1830744
6-phosphofructokinase	2.7.1.11	Rat	1833303
6-phosphofructokinase	2.7.1.11	Rat	191426
6-phosphofructokinase	2.7.1.11	Rat	2137204
6-phosphofructokinase	2.7.1.11	Rat	2147292
6-phosphofructokinase	2.7.1.11	Rat	2149746
6-phosphofructokinase	2.7.1.11	Rat	2434517
6-phosphofructokinase	2.7.1.11	Rat	2502581
6-phosphofructokinase	2.7.1.11	Rat	2522395
6-phosphofructokinase	2.7.1.11	Rat	2527305
6-phosphofructokinase	2.7.1.11	Rat	2820531
6-phosphofructokinase	2.7.1.11	Rat	28629
6-phosphofructokinase	2.7.1.11	Rat	2933146
6-phosphofructokinase	2.7.1.11	Rat	2935776
6-phosphofructokinase	2.7.1.11	Rat	2938549
6-phosphofructokinase	2.7.1.11	Rat	2956156
6-phosphofructokinase	2.7.1.11	Rat	2963653
6-phosphofructokinase	2.7.1.11	Rat	2972577
6-phosphofructokinase	2.7.1.11	Rat	2981949

6-phosphofructokinase	2.7.1.11	Rat	3364152
6-phosphofructokinase	2.7.1.11	Rat	3407760
6-phosphofructokinase	2.7.1.11	Rat	356174
6-phosphofructokinase	2.7.1.11	Rat	3768440
6-phosphofructokinase	2.7.1.11	Rat	3931461
6-phosphofructokinase	2.7.1.11	Rat	4030556
6-phosphofructokinase	2.7.1.11	Rat	4243437
6-phosphofructokinase	2.7.1.11	Rat	4252961
6-phosphofructokinase	2.7.1.11	Rat	6093562
6-phosphofructokinase	2.7.1.11	Rat	6133774
6-phosphofructokinase	2.7.1.11	Rat	6211175
6-phosphofructokinase	2.7.1.11	Rat	6231923
6-phosphofructokinase	2.7.1.11	Rat	6232272
6-phosphofructokinase	2.7.1.11	Rat	6234885
6-phosphofructokinase	2.7.1.11	Rat	6279392
6-phosphofructokinase	2.7.1.11	Rat	6325266
6-phosphofructokinase	2.7.1.11	Rat	6331422
6-phosphofructokinase	2.7.1.11	Rat	6440018
6-phosphofructokinase	2.7.1.11	Rat	6444231
6-phosphofructokinase	2.7.1.11	Rat	6452426
6-phosphofructokinase	2.7.1.11	Rat	6779470
6-phosphofructokinase	2.7.1.11	Rat	7233512
6-phosphofructokinase	2.7.1.11	Rat	7440254
6-phosphofructokinase	2.7.1.11	Rat	7522206
6-phosphofructokinase	2.7.1.11	Rat	7589825
6-phosphofructokinase	2.7.1.11	Rat	7602786
6-phosphofructokinase	2.7.1.11	Rat	7710770
6-phosphofructokinase	2.7.1.11	Rat	7875554
6-phosphofructokinase	2.7.1.11	Rat	8224738
6-phosphofructokinase	2.7.1.11	Rat	8366430
6-phosphofructokinase	2.7.1.11	Rat	8514849
6-phosphofructokinase	2.7.1.11	Rat	8557664
6-phosphofructokinase	2.7.1.11	Rat	8593533
6-phosphofructokinase	2.7.1.11	Rat	8643924
6-phosphofructokinase	2.7.1.11	Rat	8910548
6-phosphofructokinase	2.7.1.11	Rat	8981075
6-phosphofructokinase	2.7.1.11	Rat	9267516
6-phosphofructokinase	2.7.1.11	Rat	9287040
6-phosphofructokinase	2.7.1.11	Rat	9329694
6-phosphofructokinase	2.7.1.11	Rat	9371084
6-phosphofructokinase	2.7.1.11	Rat	9439886
6-phosphofructokinase	2.7.1.11	Rat	9447322
6-phosphofructokinase	2.7.1.11	Rat	9555897

6-phosphofructokinase	2.7.1.11	Rat	9580251
6-phosphofructokinase	2.7.1.11	Rat	9580875
6-phosphofructokinase	2.7.1.11	Rat	9608547
6-phosphofructokinase	2.7.1.11	Rat	9766212
6-phosphofructokinase	2.7.1.11	Rat	9777012
6-phosphofructokinase	2.7.1.11	Rat	9851886
6-phosphofructokinase	2.7.1.11	Rat	9973548
inositol-trisphosphate 3-kinase	2.7.1.127	Rat	15837423
inositol-trisphosphate 3-kinase	2.7.1.127	Rat	9211876
phosphatidylinositol 3-kinase	2.7.1.137	Rat	10869418
phosphatidylinositol 3-kinase	2.7.1.137	Rat	10874027
phosphatidylinositol 3-kinase	2.7.1.137	Rat	10998146
phosphatidylinositol 3-kinase	2.7.1.137	Rat	11259761
phosphatidylinositol 3-kinase	2.7.1.137	Rat	11744698
phosphatidylinositol 3-kinase	2.7.1.137	Rat	7669049
phosphatidylinositol 3-kinase	2.7.1.137	Rat	9292730
phosphatidylinositol 3-kinase	2.7.1.137	Rat	9312149
phosphatidylinositol 3-kinase	2.7.1.137	Rat	9478990
phosphatidylinositol 3-kinase	2.7.1.137	Rat	9826526
phosphatidylinositol 3-kinase	2.7.1.137	Rat	9826674
glucokinase	2.7.1.2	Rat	10456334
glucokinase	2.7.1.2	Rat	10494657
glucokinase	2.7.1.2	Rat	10905475
glucokinase	2.7.1.2	Rat	11311143
glucokinase	2.7.1.2	Rat	11947549
glucokinase	2.7.1.2	Rat	11950391
glucokinase	2.7.1.2	Rat	12941786
glucokinase	2.7.1.2	Rat	14979565
glucokinase	2.7.1.2	Rat	15009676
glucokinase	2.7.1.2	Rat	15016359
glucokinase	2.7.1.2	Rat	15134337
glucokinase	2.7.1.2	Rat	15226592
glucokinase	2.7.1.2	Rat	15277402
glucokinase	2.7.1.2	Rat	1545870
glucokinase	2.7.1.2	Rat	15707679
glucokinase	2.7.1.2	Rat	15955369
glucokinase	2.7.1.2	Rat	16186394
glucokinase	2.7.1.2	Rat	16834571
glucokinase	2.7.1.2	Rat	16899262
glucokinase	2.7.1.2	Rat	16916947
glucokinase	2.7.1.2	Rat	2210070
glucokinase	2.7.1.2	Rat	2584235
glucokinase	2.7.1.2	Rat	2682629

glucokinase	2.7.1.2	Rat	6780351
glucokinase	2.7.1.2	Rat	6836273
glucokinase	2.7.1.2	Rat	7010073
glucokinase	2.7.1.2	Rat	7553875
glucokinase	2.7.1.2	Rat	7821741
glucokinase	2.7.1.2	Rat	7983782
glucokinase	2.7.1.2	Rat	8194664
glucokinase	2.7.1.2	Rat	8344416
glucokinase	2.7.1.2	Rat	8433729
glucokinase	2.7.1.2	Rat	8446591
glucokinase	2.7.1.2	Rat	8549869
glucokinase	2.7.1.2	Rat	8550593
glucokinase	2.7.1.2	Rat	8631975
glucokinase	2.7.1.2	Rat	8690154
glucokinase	2.7.1.2	Rat	8692940
glucokinase	2.7.1.2	Rat	8751724
glucokinase	2.7.1.2	Rat	9113996
glucokinase	2.7.1.2	Rat	9460079
adenosine kinase	2.7.1.20	Rat	10794412
adenosine kinase	2.7.1.20	Rat	12228764
adenosine kinase	2.7.1.20	Rat	15632276
adenosine kinase	2.7.1.20	Rat	2154328
adenosine kinase	2.7.1.20	Rat	8184939
glycerol kinase	2.7.1.30	Rat	10.1002/anie.198810401
glycerol kinase	2.7.1.30	Rat	11388799
glycerol kinase	2.7.1.30	Rat	11811537
glycerol kinase	2.7.1.30	Rat	1985967
glycerol kinase	2.7.1.30	Rat	2547969
glycerol kinase	2.7.1.30	Rat	4914079
glycerol kinase	2.7.1.30	Rat	6292169
glycerol kinase	2.7.1.30	Rat	6440018
glycerol kinase	2.7.1.30	Rat	9162046
choline kinase	2.7.1.32	Rat	1336121
choline kinase	2.7.1.32	Rat	16490392
choline kinase	2.7.1.32	Rat	2153442
choline kinase	2.7.1.32	Rat	217369
choline kinase	2.7.1.32	Rat	3365445
choline kinase	2.7.1.32	Rat	3447597
choline kinase	2.7.1.32	Rat	5495730
choline kinase	2.7.1.32	Rat	6503617
choline kinase	2.7.1.32	Rat	8182083
choline kinase	2.7.1.32	Rat	828054
choline kinase	2.7.1.32	Rat	8414498

pantothenate kinase	2.7.1.33	Rat	10625688
pantothenate kinase	2.7.1.33	Rat	11809413
pantothenate kinase	2.7.1.33	Rat	12697433
pantothenate kinase	2.7.1.33	Rat	15176870
pantothenate kinase	2.7.1.33	Rat	15843025
pantothenate kinase	2.7.1.33	Rat	16701556
pantothenate kinase	2.7.1.33	Rat	17323930
pantothenate kinase	2.7.1.33	Rat	17581817
pantothenate kinase	2.7.1.33	Rat	9890959
pyruvate kinase	2.7.1.40	Rat	101523
pyruvate kinase	2.7.1.40	Rat	11181519
pyruvate kinase	2.7.1.40	Rat	1175605
pyruvate kinase	2.7.1.40	Rat	1328007
pyruvate kinase	2.7.1.40	Rat	1406667
pyruvate kinase	2.7.1.40	Rat	15028426
pyruvate kinase	2.7.1.40	Rat	15567985
pyruvate kinase	2.7.1.40	Rat	16046853
pyruvate kinase	2.7.1.40	Rat	16511150
pyruvate kinase	2.7.1.40	Rat	16549526
pyruvate kinase	2.7.1.40	Rat	1959479
pyruvate kinase	2.7.1.40	Rat	2387024
pyruvate kinase	2.7.1.40	Rat	2813362
pyruvate kinase	2.7.1.40	Rat	2820531
pyruvate kinase	2.7.1.40	Rat	2846196
pyruvate kinase	2.7.1.40	Rat	291050
pyruvate kinase	2.7.1.40	Rat	29278
pyruvate kinase	2.7.1.40	Rat	2935776
pyruvate kinase	2.7.1.40	Rat	2970638
pyruvate kinase	2.7.1.40	Rat	3023262
pyruvate kinase	2.7.1.40	Rat	3032541
pyruvate kinase	2.7.1.40	Rat	3159473
pyruvate kinase	2.7.1.40	Rat	3161219
pyruvate kinase	2.7.1.40	Rat	3350145
pyruvate kinase	2.7.1.40	Rat	4053567
pyruvate kinase	2.7.1.40	Rat	6222515
pyruvate kinase	2.7.1.40	Rat	6241274
pyruvate kinase	2.7.1.40	Rat	6268138
pyruvate kinase	2.7.1.40	Rat	6370232
pyruvate kinase	2.7.1.40	Rat	6588273
pyruvate kinase	2.7.1.40	Rat	6682991
pyruvate kinase	2.7.1.40	Rat	6713301
pyruvate kinase	2.7.1.40	Rat	7357032
pyruvate kinase	2.7.1.40	Rat	7961441

pyruvate kinase	2.7.1.40	Rat	8074527
pyruvate kinase	2.7.1.40	Rat	8144600
pyruvate kinase	2.7.1.40	Rat	8436141
pyruvate kinase	2.7.1.40	Rat	8476115
pyruvate kinase	2.7.1.40	Rat	8765986
pyruvate kinase	2.7.1.40	Rat	9252361
N-acylmannosamine kinase	2.7.1.60	Rat	15987957
1-phosphatidylinositol 4-kinase	2.7.1.67	Rat	12594831
1-phosphatidylinositol 4-kinase	2.7.1.67	Rat	12620118
1-phosphatidylinositol 4-kinase	2.7.1.67	Rat	16912074
1-phosphatidylinositol 4-kinase	2.7.1.67	Rat	17003043
1-phosphatidylinositol 4-kinase	2.7.1.67	Rat	7961848
1-phosphatidylinositol 4-kinase	2.7.1.67	Rat	8152413
1-phosphatidylinositol 4-kinase	2.7.1.67	Rat	8190262
1-phosphatidylinositol 4-kinase	2.7.1.67	Rat	9654085
1-phosphatidylinositol 4-kinase	2.7.1.67	Rat	9854149
1-phosphatidylinositol 4-kinase	2.7.1.67	Rat	9891985
1-phosphatidylinositol-4-phosphate 5-kinase	2.7.1.68	Rat	11098053
1-phosphatidylinositol-4-phosphate 5-kinase	2.7.1.68	Rat	12620118
1-phosphatidylinositol-4-phosphate 5-kinase	2.7.1.68	Rat	15277528
1-phosphatidylinositol-4-phosphate 5-kinase	2.7.1.68	Rat	15738269
1-phosphatidylinositol-4-phosphate 5-kinase	2.7.1.68	Rat	17635937
1-phosphatidylinositol-4-phosphate 5-kinase	2.7.1.68	Rat	2849321
1-phosphatidylinositol-4-phosphate 5-kinase	2.7.1.68	Rat	8190262
1-phosphatidylinositol-4-phosphate 5-kinase	2.7.1.68	Rat	9292730
deoxycytidine kinase	2.7.1.74	Rat	10499616
deoxycytidine kinase	2.7.1.74	Rat	10848830
deoxycytidine kinase	2.7.1.74	Rat	11888330
deoxycytidine kinase	2.7.1.74	Rat	12054682
deoxycytidine kinase	2.7.1.74	Rat	15561147
deoxycytidine kinase	2.7.1.74	Rat	15803490
deoxycytidine kinase	2.7.1.74	Rat	16180016
deoxycytidine kinase	2.7.1.74	Rat	16421443
deoxycytidine kinase	2.7.1.74	Rat	16463058
deoxycytidine kinase	2.7.1.74	Rat	17065079
deoxycytidine kinase	2.7.1.74	Rat	2436757
deoxycytidine kinase	2.7.1.74	Rat	3335008
deoxycytidine kinase	2.7.1.74	Rat	7805176
deoxycytidine kinase	2.7.1.74	Rat	8616717
deoxycytidine kinase	2.7.1.74	Rat	8718419
ethanolamine kinase	2.7.1.82	Rat	11044454
ethanolamine kinase	2.7.1.82	Rat	1480155
ethanolamine kinase	2.7.1.82	Rat	475777

ethanolamine kinase	2.7.1.82	Rat	7142139
sphinganine kinase	2.7.1.91	Rat	10567432
sphinganine kinase	2.7.1.91	Rat	14568343
sphinganine kinase	2.7.1.91	Rat	15451787
sphinganine kinase	2.7.1.91	Rat	16831409
adenylate kinase	2.7.4.3	Rat	126626
adenylate kinase	2.7.4.3	Rat	14656997
adenylate kinase	2.7.4.3	Rat	15941717
adenylate kinase	2.7.4.3	Rat	16668787
adenylate kinase	2.7.4.3	Rat	6440018
adenylate kinase	2.7.4.3	Rat	7764491
ribose-phosphate diphosphokinase	2.7.6.1	Rat	15878857
ribose-phosphate diphosphokinase	2.7.6.1	Rat	217337
ethanolamine-phosphate cytidylyltransferase	2.7.7.14	Rat	10493918
ethanolamine-phosphate cytidylyltransferase	2.7.7.14	Rat	10752579
ethanolamine-phosphate cytidylyltransferase	2.7.7.14	Rat	11097182
ethanolamine-phosphate cytidylyltransferase	2.7.7.14	Rat	11844611
ethanolamine-phosphate cytidylyltransferase	2.7.7.14	Rat	14697519
ethanolamine-phosphate cytidylyltransferase	2.7.7.14	Rat	15147238
ethanolamine-phosphate cytidylyltransferase	2.7.7.14	Rat	475777
ethanolamine-phosphate cytidylyltransferase	2.7.7.14	Rat	603639
ethanolamine-phosphate cytidylyltransferase	2.7.7.14	Rat	6626563
choline-phosphate cytidylyltransferase	2.7.7.15	Rat	10101264
choline-phosphate cytidylyltransferase	2.7.7.15	Rat	10208837
choline-phosphate cytidylyltransferase	2.7.7.15	Rat	10473578
choline-phosphate cytidylyltransferase	2.7.7.15	Rat	10615073
choline-phosphate cytidylyltransferase	2.7.7.15	Rat	10908674
choline-phosphate cytidylyltransferase	2.7.7.15	Rat	10946015
choline-phosphate cytidylyltransferase	2.7.7.15	Rat	11029581
choline-phosphate cytidylyltransferase	2.7.7.15	Rat	11097182
choline-phosphate cytidylyltransferase	2.7.7.15	Rat	11279002
choline-phosphate cytidylyltransferase	2.7.7.15	Rat	11404252
choline-phosphate cytidylyltransferase	2.7.7.15	Rat	11404253
choline-phosphate cytidylyltransferase	2.7.7.15	Rat	11521967
choline-phosphate cytidylyltransferase	2.7.7.15	Rat	11829742
choline-phosphate cytidylyltransferase	2.7.7.15	Rat	12034570
choline-phosphate cytidylyltransferase	2.7.7.15	Rat	12052891
choline-phosphate cytidylyltransferase	2.7.7.15	Rat	12062780
choline-phosphate cytidylyltransferase	2.7.7.15	Rat	12221122
choline-phosphate cytidylyltransferase	2.7.7.15	Rat	12271462
choline-phosphate cytidylyltransferase	2.7.7.15	Rat	12370080
choline-phosphate cytidylyltransferase	2.7.7.15	Rat	12620118
choline-phosphate cytidylyltransferase	2.7.7.15	Rat	12659631

choline-phosphate cytidylyltransferase	2.7.7.15	Rat	12842190
choline-phosphate cytidylyltransferase	2.7.7.15	Rat	12928431
choline-phosphate cytidylyltransferase	2.7.7.15	Rat	14536058
choline-phosphate cytidylyltransferase	2.7.7.15	Rat	15079868
choline-phosphate cytidylyltransferase	2.7.7.15	Rat	15139854
choline-phosphate cytidylyltransferase	2.7.7.15	Rat	15210848
choline-phosphate cytidylyltransferase	2.7.7.15	Rat	15522825
choline-phosphate cytidylyltransferase	2.7.7.15	Rat	15574675
choline-phosphate cytidylyltransferase	2.7.7.15	Rat	15635091
choline-phosphate cytidylyltransferase	2.7.7.15	Rat	15788406
choline-phosphate cytidylyltransferase	2.7.7.15	Rat	15982005
choline-phosphate cytidylyltransferase	2.7.7.15	Rat	16097951
choline-phosphate cytidylyltransferase	2.7.7.15	Rat	16153613
choline-phosphate cytidylyltransferase	2.7.7.15	Rat	16236026
choline-phosphate cytidylyltransferase	2.7.7.15	Rat	16511521
choline-phosphate cytidylyltransferase	2.7.7.15	Rat	16580250
choline-phosphate cytidylyltransferase	2.7.7.15	Rat	16580875
choline-phosphate cytidylyltransferase	2.7.7.15	Rat	2160812
choline-phosphate cytidylyltransferase	2.7.7.15	Rat	2268410
choline-phosphate cytidylyltransferase	2.7.7.15	Rat	2665794
choline-phosphate cytidylyltransferase	2.7.7.15	Rat	2833508
choline-phosphate cytidylyltransferase	2.7.7.15	Rat	2838058
choline-phosphate cytidylyltransferase	2.7.7.15	Rat	3004590
choline-phosphate cytidylyltransferase	2.7.7.15	Rat	3365445
choline-phosphate cytidylyltransferase	2.7.7.15	Rat	3367156
choline-phosphate cytidylyltransferase	2.7.7.15	Rat	3447597
choline-phosphate cytidylyltransferase	2.7.7.15	Rat	603639
choline-phosphate cytidylyltransferase	2.7.7.15	Rat	6134645
choline-phosphate cytidylyltransferase	2.7.7.15	Rat	6243289
choline-phosphate cytidylyltransferase	2.7.7.15	Rat	6477961
choline-phosphate cytidylyltransferase	2.7.7.15	Rat	7126613
choline-phosphate cytidylyltransferase	2.7.7.15	Rat	7487944
choline-phosphate cytidylyltransferase	2.7.7.15	Rat	7588775
choline-phosphate cytidylyltransferase	2.7.7.15	Rat	7637558
choline-phosphate cytidylyltransferase	2.7.7.15	Rat	7768909
choline-phosphate cytidylyltransferase	2.7.7.15	Rat	7782919
choline-phosphate cytidylyltransferase	2.7.7.15	Rat	7836412
choline-phosphate cytidylyltransferase	2.7.7.15	Rat	8006517
choline-phosphate cytidylyltransferase	2.7.7.15	Rat	8182083
choline-phosphate cytidylyltransferase	2.7.7.15	Rat	8239319
choline-phosphate cytidylyltransferase	2.7.7.15	Rat	8255685
choline-phosphate cytidylyltransferase	2.7.7.15	Rat	8387510
choline-phosphate cytidylyltransferase	2.7.7.15	Rat	8504126

choline-phosphate cytidylyltransferase	2.7.7.15	Rat	8597584
choline-phosphate cytidylyltransferase	2.7.7.15	Rat	8626633
choline-phosphate cytidylyltransferase	2.7.7.15	Rat	8663247
choline-phosphate cytidylyltransferase	2.7.7.15	Rat	8756587
choline-phosphate cytidylyltransferase	2.7.7.15	Rat	8761490
choline-phosphate cytidylyltransferase	2.7.7.15	Rat	8810902
choline-phosphate cytidylyltransferase	2.7.7.15	Rat	9009253
choline-phosphate cytidylyltransferase	2.7.7.15	Rat	9046356
choline-phosphate cytidylyltransferase	2.7.7.15	Rat	9148929
choline-phosphate cytidylyltransferase	2.7.7.15	Rat	9335949
choline-phosphate cytidylyltransferase	2.7.7.15	Rat	9370319
choline-phosphate cytidylyltransferase	2.7.7.15	Rat	9421188
choline-phosphate cytidylyltransferase	2.7.7.15	Rat	9714757
phosphatidate cytidylyltransferase	2.7.7.41	Rat	11985865
phosphatidate cytidylyltransferase	2.7.7.41	Rat	9345289
N-acylneuraminate cytidylyltransferase	2.7.7.43	Rat	10320348
N-acylneuraminate cytidylyltransferase	2.7.7.43	Rat	3024643
N-acylneuraminate cytidylyltransferase	2.7.7.43	Rat	7830552
polyribonucleotide nucleotidyltransferase	2.7.7.8	Rat	126862
alcohol sulfotransferase	2.8.2.2	Rat	7900959
[heparan sulfate]-glucosamine 3-sulfotransferase 1	2.8.2.23	Rat	12671048
sterol esterase	3.1.1.13	Rat	10569995
sterol esterase	3.1.1.13	Rat	15809341
sterol esterase	3.1.1.13	Rat	8258956
triacylglycerol lipase	3.1.1.3	Rat	11217140
triacylglycerol lipase	3.1.1.3	Rat	12689525
triacylglycerol lipase	3.1.1.3	Rat	3678753
lipoprotein lipase	3.1.1.34	Rat	10077655
lipoprotein lipase	3.1.1.34	Rat	10226565
lipoprotein lipase	3.1.1.34	Rat	10364085
lipoprotein lipase	3.1.1.34	Rat	10388470
lipoprotein lipase	3.1.1.34	Rat	10515359
lipoprotein lipase	3.1.1.34	Rat	10650951
lipoprotein lipase	3.1.1.34	Rat	10704617
lipoprotein lipase	3.1.1.34	Rat	10965219
lipoprotein lipase	3.1.1.34	Rat	11158876
lipoprotein lipase	3.1.1.34	Rat	11334409
lipoprotein lipase	3.1.1.34	Rat	11432868
lipoprotein lipase	3.1.1.34	Rat	11591230
lipoprotein lipase	3.1.1.34	Rat	12079052
lipoprotein lipase	3.1.1.34	Rat	12352010
lipoprotein lipase	3.1.1.34	Rat	12847564

lipoprotein lipase	3.1.1.34	Rat	12862202
lipoprotein lipase	3.1.1.34	Rat	12934668
lipoprotein lipase	3.1.1.34	Rat	1401083
lipoprotein lipase	3.1.1.34	Rat	14580165
lipoprotein lipase	3.1.1.34	Rat	14656997
lipoprotein lipase	3.1.1.34	Rat	14660566
lipoprotein lipase	3.1.1.34	Rat	15178298
lipoprotein lipase	3.1.1.34	Rat	15262189
lipoprotein lipase	3.1.1.34	Rat	15320848
lipoprotein lipase	3.1.1.34	Rat	15562391
lipoprotein lipase	3.1.1.34	Rat	15697220
lipoprotein lipase	3.1.1.34	Rat	15801017
lipoprotein lipase	3.1.1.34	Rat	16195388
lipoprotein lipase	3.1.1.34	Rat	16416313
lipoprotein lipase	3.1.1.34	Rat	16531751
lipoprotein lipase	3.1.1.34	Rat	16767221
lipoprotein lipase	3.1.1.34	Rat	1737833
lipoprotein lipase	3.1.1.34	Rat	1999438
lipoprotein lipase	3.1.1.34	Rat	2117022
lipoprotein lipase	3.1.1.34	Rat	2165281
lipoprotein lipase	3.1.1.34	Rat	2765496
lipoprotein lipase	3.1.1.34	Rat	3304415
lipoprotein lipase	3.1.1.34	Rat	3519325
lipoprotein lipase	3.1.1.34	Rat	3817303
lipoprotein lipase	3.1.1.34	Rat	3920639
lipoprotein lipase	3.1.1.34	Rat	3950770
lipoprotein lipase	3.1.1.34	Rat	4077022
lipoprotein lipase	3.1.1.34	Rat	6482738
lipoprotein lipase	3.1.1.34	Rat	7033153
lipoprotein lipase	3.1.1.34	Rat	7126037
lipoprotein lipase	3.1.1.34	Rat	7229033
lipoprotein lipase	3.1.1.34	Rat	7276825
lipoprotein lipase	3.1.1.34	Rat	7592875
lipoprotein lipase	3.1.1.34	Rat	7630312
lipoprotein lipase	3.1.1.34	Rat	7939219
lipoprotein lipase	3.1.1.34	Rat	7956906
lipoprotein lipase	3.1.1.34	Rat	8371063
lipoprotein lipase	3.1.1.34	Rat	8374222
lipoprotein lipase	3.1.1.34	Rat	8422428
lipoprotein lipase	3.1.1.34	Rat	8480620
lipoprotein lipase	3.1.1.34	Rat	8729382
lipoprotein lipase	3.1.1.34	Rat	8882874
lipoprotein lipase	3.1.1.34	Rat	8919277

lipoprotein lipase	3.1.1.34	Rat	9188470
lipoprotein lipase	3.1.1.34	Rat	9193431
lipoprotein lipase	3.1.1.34	Rat	9264396
lipoprotein lipase	3.1.1.34	Rat	9294198
lipoprotein lipase	3.1.1.34	Rat	9298816
lipoprotein lipase	3.1.1.34	Rat	9351402
lipoprotein lipase	3.1.1.34	Rat	9358077
lipoprotein lipase	3.1.1.34	Rat	9382958
lipoprotein lipase	3.1.1.34	Rat	9495276
lipoprotein lipase	3.1.1.34	Rat	9727057
lipoprotein lipase	3.1.1.34	Rat	9888641
lipoprotein lipase	3.1.1.34	Rat	9888650
lipoprotein lipase	3.1.1.34	Rat	9924194
lipoprotein lipase	3.1.1.34	Rat	9973300
phospholipase A2	3.1.1.4	Rat	10435206
phospholipase A2	3.1.1.4	Rat	10482042
phospholipase A2	3.1.1.4	Rat	10614936
phospholipase A2	3.1.1.4	Rat	106389
phospholipase A2	3.1.1.4	Rat	10749741
phospholipase A2	3.1.1.4	Rat	10793641
phospholipase A2	3.1.1.4	Rat	10919502
phospholipase A2	3.1.1.4	Rat	10970711
phospholipase A2	3.1.1.4	Rat	11080682
phospholipase A2	3.1.1.4	Rat	11085935
phospholipase A2	3.1.1.4	Rat	11099485
phospholipase A2	3.1.1.4	Rat	11115401
phospholipase A2	3.1.1.4	Rat	11328947
phospholipase A2	3.1.1.4	Rat	11374398
phospholipase A2	3.1.1.4	Rat	11964173
phospholipase A2	3.1.1.4	Rat	12076714
phospholipase A2	3.1.1.4	Rat	12143044
phospholipase A2	3.1.1.4	Rat	12189011
phospholipase A2	3.1.1.4	Rat	124658
phospholipase A2	3.1.1.4	Rat	12724134
phospholipase A2	3.1.1.4	Rat	12855693
phospholipase A2	3.1.1.4	Rat	1322564
phospholipase A2	3.1.1.4	Rat	1400321
phospholipase A2	3.1.1.4	Rat	1410519
phospholipase A2	3.1.1.4	Rat	14561756
phospholipase A2	3.1.1.4	Rat	15041029
phospholipase A2	3.1.1.4	Rat	1510970
phospholipase A2	3.1.1.4	Rat	15211007
phospholipase A2	3.1.1.4	Rat	15214789

phospholipase A2	3.1.1.4	Rat	15283760
phospholipase A2	3.1.1.4	Rat	15377291
phospholipase A2	3.1.1.4	Rat	15472477
phospholipase A2	3.1.1.4	Rat	15521009
phospholipase A2	3.1.1.4	Rat	15743759
phospholipase A2	3.1.1.4	Rat	15900018
phospholipase A2	3.1.1.4	Rat	16179540
phospholipase A2	3.1.1.4	Rat	16221889
phospholipase A2	3.1.1.4	Rat	16318667
phospholipase A2	3.1.1.4	Rat	16443193
phospholipase A2	3.1.1.4	Rat	16716827
phospholipase A2	3.1.1.4	Rat	16754327
phospholipase A2	3.1.1.4	Rat	16794537
phospholipase A2	3.1.1.4	Rat	16968951
phospholipase A2	3.1.1.4	Rat	17008548
phospholipase A2	3.1.1.4	Rat	1762050
phospholipase A2	3.1.1.4	Rat	1836009
phospholipase A2	3.1.1.4	Rat	1976627
phospholipase A2	3.1.1.4	Rat	2075195
phospholipase A2	3.1.1.4	Rat	2217203
phospholipase A2	3.1.1.4	Rat	2218714
phospholipase A2	3.1.1.4	Rat	2223919
phospholipase A2	3.1.1.4	Rat	2250570
phospholipase A2	3.1.1.4	Rat	2354835
phospholipase A2	3.1.1.4	Rat	2454329
phospholipase A2	3.1.1.4	Rat	2646218
phospholipase A2	3.1.1.4	Rat	2742867
phospholipase A2	3.1.1.4	Rat	2841909
phospholipase A2	3.1.1.4	Rat	2848583
phospholipase A2	3.1.1.4	Rat	3085592
phospholipase A2	3.1.1.4	Rat	3141409
phospholipase A2	3.1.1.4	Rat	3164726
phospholipase A2	3.1.1.4	Rat	3240001
phospholipase A2	3.1.1.4	Rat	3343241
phospholipase A2	3.1.1.4	Rat	3745162
phospholipase A2	3.1.1.4	Rat	386140
phospholipase A2	3.1.1.4	Rat	6135325
phospholipase A2	3.1.1.4	Rat	6527548
phospholipase A2	3.1.1.4	Rat	6955805
phospholipase A2	3.1.1.4	Rat	7539396
phospholipase A2	3.1.1.4	Rat	7649158
phospholipase A2	3.1.1.4	Rat	7681828
phospholipase A2	3.1.1.4	Rat	7835820

phospholipase A2	3.1.1.4	Rat	7938094
phospholipase A2	3.1.1.4	Rat	7945230
phospholipase A2	3.1.1.4	Rat	7998975
phospholipase A2	3.1.1.4	Rat	8067978
phospholipase A2	3.1.1.4	Rat	8148385
phospholipase A2	3.1.1.4	Rat	8278618
phospholipase A2	3.1.1.4	Rat	8280164
phospholipase A2	3.1.1.4	Rat	8292024
phospholipase A2	3.1.1.4	Rat	8307447
phospholipase A2	3.1.1.4	Rat	8307472
phospholipase A2	3.1.1.4	Rat	8347632
phospholipase A2	3.1.1.4	Rat	8536274
phospholipase A2	3.1.1.4	Rat	8570769
phospholipase A2	3.1.1.4	Rat	8648901
phospholipase A2	3.1.1.4	Rat	8739397
phospholipase A2	3.1.1.4	Rat	8773214
phospholipase A2	3.1.1.4	Rat	8808111
phospholipase A2	3.1.1.4	Rat	8832056
phospholipase A2	3.1.1.4	Rat	8863185
phospholipase A2	3.1.1.4	Rat	8865467
phospholipase A2	3.1.1.4	Rat	8873778
phospholipase A2	3.1.1.4	Rat	8888134
phospholipase A2	3.1.1.4	Rat	9172747
phospholipase A2	3.1.1.4	Rat	9173912
phospholipase A2	3.1.1.4	Rat	9187306
phospholipase A2	3.1.1.4	Rat	9219895
phospholipase A2	3.1.1.4	Rat	9221748
phospholipase A2	3.1.1.4	Rat	9275050
phospholipase A2	3.1.1.4	Rat	9314599
phospholipase A2	3.1.1.4	Rat	9322315
phospholipase A2	3.1.1.4	Rat	9398170
phospholipase A2	3.1.1.4	Rat	9410883
phospholipase A2	3.1.1.4	Rat	9425915
phospholipase A2	3.1.1.4	Rat	9448723
phospholipase A2	3.1.1.4	Rat	9463402
phospholipase A2	3.1.1.4	Rat	9513902
phospholipase A2	3.1.1.4	Rat	9538252
phospholipase A2	3.1.1.4	Rat	9562240
phospholipase A2	3.1.1.4	Rat	9585093
phospholipase A2	3.1.1.4	Rat	9764845
phospholipase A2	3.1.1.4	Rat	9879666
acetylcholinesterase	3.1.1.7	Rat	10766776
acetylcholinesterase	3.1.1.7	Rat	12415870

acetylcholinesterase	3.1.1.7	Rat	15936353
acetylcholinesterase	3.1.1.7	Rat	17562604
acetylcholinesterase	3.1.1.7	Rat	1820094
acetylcholinesterase	3.1.1.7	Rat	2587621
acetylcholinesterase	3.1.1.7	Rat	4795368
acetylcholinesterase	3.1.1.7	Rat	668
acetylcholinesterase	3.1.1.7	Rat	9109840
acetylcholinesterase	3.1.1.7	Rat	9548556
alkaline phosphatase	3.1.3.1	Rat	11029583
alkaline phosphatase	3.1.3.1	Rat	11139445
alkaline phosphatase	3.1.3.1	Rat	12412807
alkaline phosphatase	3.1.3.1	Rat	1445337
alkaline phosphatase	3.1.3.1	Rat	14560000
alkaline phosphatase	3.1.3.1	Rat	3509742
alkaline phosphatase	3.1.3.1	Rat	7032602
fructose-bisphosphatase	3.1.3.11	Rat	10327613
fructose-bisphosphatase	3.1.3.11	Rat	10393302
fructose-bisphosphatase	3.1.3.11	Rat	10773464
fructose-bisphosphatase	3.1.3.11	Rat	11536627
fructose-bisphosphatase	3.1.3.11	Rat	11864619
fructose-bisphosphatase	3.1.3.11	Rat	12190028
fructose-bisphosphatase	3.1.3.11	Rat	12686616
fructose-bisphosphatase	3.1.3.11	Rat	15225753
fructose-bisphosphatase	3.1.3.11	Rat	15498578
fructose-bisphosphatase	3.1.3.11	Rat	15631980
fructose-bisphosphatase	3.1.3.11	Rat	16199065
fructose-bisphosphatase	3.1.3.11	Rat	16580859
fructose-bisphosphatase	3.1.3.11	Rat	16593209
fructose-bisphosphatase	3.1.3.11	Rat	16657971
fructose-bisphosphatase	3.1.3.11	Rat	16814784
fructose-bisphosphatase	3.1.3.11	Rat	16857246
fructose-bisphosphatase	3.1.3.11	Rat	2835013
fructose-bisphosphatase	3.1.3.11	Rat	2983680
fructose-bisphosphatase	3.1.3.11	Rat	3068502
fructose-bisphosphatase	3.1.3.11	Rat	3291467
fructose-bisphosphatase	3.1.3.11	Rat	4342496
fructose-bisphosphatase	3.1.3.11	Rat	7532742
fructose-bisphosphatase	3.1.3.11	Rat	7552262
fructose-bisphosphatase	3.1.3.11	Rat	7558035
fructose-bisphosphatase	3.1.3.11	Rat	7579072
fructose-bisphosphatase	3.1.3.11	Rat	7592860
fructose-bisphosphatase	3.1.3.11	Rat	8043646
acid phosphatase	3.1.3.2	Rat	10471332

acid phosphatase	3.1.3.2	Rat	10646119
acid phosphatase	3.1.3.2	Rat	1364364
acid phosphatase	3.1.3.2	Rat	2153810
acid phosphatase	3.1.3.2	Rat	2545859
acid phosphatase	3.1.3.2	Rat	2713876
acid phosphatase	3.1.3.2	Rat	9370316
inositol-phosphate phosphatase	3.1.3.25	Rat	12479670
inositol-phosphate phosphatase	3.1.3.25	Rat	15809430
inositol-phosphate phosphatase	3.1.3.25	Rat	9462881
phosphoserine phosphatase	3.1.3.3	Rat	8858931
phosphatidate phosphatase	3.1.3.4	Rat	10695929
phosphatidate phosphatase	3.1.3.4	Rat	11678440
phosphatidate phosphatase	3.1.3.4	Rat	12359092
phosphatidate phosphatase	3.1.3.4	Rat	15527069
phosphatidate phosphatase	3.1.3.4	Rat	15539300
phosphatidate phosphatase	3.1.3.4	Rat	15975496
phosphatidate phosphatase	3.1.3.4	Rat	16684527
phosphatidate phosphatase	3.1.3.4	Rat	16968695
phosphatidate phosphatase	3.1.3.4	Rat	2153810
phosphatidate phosphatase	3.1.3.4	Rat	2174075
phosphatidate phosphatase	3.1.3.4	Rat	2674663
phosphatidate phosphatase	3.1.3.4	Rat	2713876
phosphatidate phosphatase	3.1.3.4	Rat	6277612
phosphatidate phosphatase	3.1.3.4	Rat	716971
phosphatidate phosphatase	3.1.3.4	Rat	7273126
phosphatidate phosphatase	3.1.3.4	Rat	7305894
phosphatidate phosphatase	3.1.3.4	Rat	7407220
phosphatidate phosphatase	3.1.3.4	Rat	7748273
phosphatidate phosphatase	3.1.3.4	Rat	8406356
phosphatidate phosphatase	3.1.3.4	Rat	8784738
5'-nucleotidase	3.1.3.5	Rat	10506947
5'-nucleotidase	3.1.3.5	Rat	10617137
5'-nucleotidase	3.1.3.5	Rat	10766785
5'-nucleotidase	3.1.3.5	Rat	10869532
5'-nucleotidase	3.1.3.5	Rat	11408527
5'-nucleotidase	3.1.3.5	Rat	12030367
5'-nucleotidase	3.1.3.5	Rat	12061138
5'-nucleotidase	3.1.3.5	Rat	12204768
5'-nucleotidase	3.1.3.5	Rat	12493585
5'-nucleotidase	3.1.3.5	Rat	12571440
5'-nucleotidase	3.1.3.5	Rat	12667292
5'-nucleotidase	3.1.3.5	Rat	15748706
5'-nucleotidase	3.1.3.5	Rat	15946667

5'-nucleotidase	3.1.3.5	Rat	6326848
5'-nucleotidase	3.1.3.5	Rat	7999131
5'-nucleotidase	3.1.3.5	Rat	9009712
5'-nucleotidase	3.1.3.5	Rat	9315889
5'-nucleotidase	3.1.3.5	Rat	9806332
glucose-6-phosphatase	3.1.3.9	Rat	10625614
glucose-6-phosphatase	3.1.3.9	Rat	12189168
glucose-6-phosphatase	3.1.3.9	Rat	12373573
glucose-6-phosphatase	3.1.3.9	Rat	12507516
glucose-6-phosphatase	3.1.3.9	Rat	1323600
glucose-6-phosphatase	3.1.3.9	Rat	15702236
glucose-6-phosphatase	3.1.3.9	Rat	16012821
glucose-6-phosphatase	3.1.3.9	Rat	16330542
glucose-6-phosphatase	3.1.3.9	Rat	17075770
glucose-6-phosphatase	3.1.3.9	Rat	3038860
glucose-6-phosphatase	3.1.3.9	Rat	3629725
glucose-6-phosphatase	3.1.3.9	Rat	6295653
glucose-6-phosphatase	3.1.3.9	Rat	6317150
glucose-6-phosphatase	3.1.3.9	Rat	6591771
glucose-6-phosphatase	3.1.3.9	Rat	8182131
glucose-6-phosphatase	3.1.3.9	Rat	8211187
glucose-6-phosphatase	3.1.3.9	Rat	8407995
glucose-6-phosphatase	3.1.3.9	Rat	8640227
glucose-6-phosphatase	3.1.3.9	Rat	9369482
glucose-6-phosphatase	3.1.3.9	Rat	9497333
glucose-6-phosphatase	3.1.3.9	Rat	9506766
glucose-6-phosphatase	3.1.3.9	Rat	9839806
alpha-glucosidase	3.2.1.20	Rat	10619707
alpha-glucosidase	3.2.1.20	Rat	10810293
alpha-glucosidase	3.2.1.20	Rat	11134937
alpha-glucosidase	3.2.1.20	Rat	11230125
alpha-glucosidase	3.2.1.20	Rat	11404235
alpha-glucosidase	3.2.1.20	Rat	16233203
alpha-glucosidase	3.2.1.20	Rat	16233375
alpha-glucosidase	3.2.1.20	Rat	7626594
alpha-glucosidase	3.2.1.20	Rat	8625892
alpha-mannosidase	3.2.1.24	Rat	11406577
alpha-mannosidase	3.2.1.24	Rat	16460512
alpha-mannosidase	3.2.1.24	Rat	16806128
alpha-mannosidase	3.2.1.24	Rat	16899540
alpha-mannosidase	3.2.1.24	Rat	2466460
alpha-mannosidase	3.2.1.24	Rat	2843530
alpha-mannosidase	3.2.1.24	Rat	4092864

alpha-mannosidase	3.2.1.24	Rat	9022667
alpha-mannosidase	3.2.1.24	Rat	9230311
leukotriene-A4 hydrolase	3.3.2.6	Rat	10691697
leukotriene-A4 hydrolase	3.3.2.6	Rat	11805219
leukotriene-A4 hydrolase	3.3.2.6	Rat	12139459
leukotriene-A4 hydrolase	3.3.2.6	Rat	12865451
leukotriene-A4 hydrolase	3.3.2.6	Rat	1311589
leukotriene-A4 hydrolase	3.3.2.6	Rat	6329309
leukotriene-A4 hydrolase	3.3.2.6	Rat	7732829
leukotriene-A4 hydrolase	3.3.2.6	Rat	9395533
leukotriene-A4 hydrolase	3.3.2.6	Rat	9413890
membrane alanyl aminopeptidase	3.4.11.2	Rat	11751433
membrane alanyl aminopeptidase	3.4.11.2	Rat	12075625
membrane alanyl aminopeptidase	3.4.11.2	Rat	16019130
GTP cyclohydrolase I	3.5.4.16	Rat	12392559
GTP cyclohydrolase I	3.5.4.16	Rat	12855421
GTP cyclohydrolase I	3.5.4.16	Rat	1459137
GTP cyclohydrolase I	3.5.4.16	Rat	15044686
GTP cyclohydrolase I	3.5.4.16	Rat	16179591
GTP cyclohydrolase I	3.5.4.16	Rat	16636057
GTP cyclohydrolase I	3.5.4.16	Rat	737222
GTP cyclohydrolase I	3.5.4.16	Rat	7521513
GTP cyclohydrolase I	3.5.4.16	Rat	9182249
GTP cyclohydrolase I	3.5.4.16	Rat	9444617
adenosinetriphosphatase	3.6.1.3	Rat	126449
adenosinetriphosphatase	3.6.1.3	Rat	160792
adenosinetriphosphatase	3.6.1.3	Rat	1727783
adenosinetriphosphatase	3.6.1.3	Rat	3772811
adenosinetriphosphatase	3.6.1.3	Rat	8667177
H+-transporting two-sector ATPase	3.6.3.14	Rat	11744700
H+-transporting two-sector ATPase	3.6.3.14	Rat	11893513
H+-transporting two-sector ATPase	3.6.3.14	Rat	12587531
H+-transporting two-sector ATPase	3.6.3.14	Rat	15712234
H+-transporting two-sector ATPase	3.6.3.14	Rat	16510118
H+-transporting two-sector ATPase	3.6.3.14	Rat	16730639
H+-transporting two-sector ATPase	3.6.3.14	Rat	2532597
H+-transporting two-sector ATPase	3.6.3.14	Rat	2889730
H+-transporting two-sector ATPase	3.6.3.14	Rat	6456904
H+-transporting two-sector ATPase	3.6.3.14	Rat	8516333
fumarylacetoacetase	3.7.1.2	Rat	9734339
glutamate decarboxylase	4.1.1.15	Rat	10331265
glutamate decarboxylase	4.1.1.15	Rat	12196588
glutamate decarboxylase	4.1.1.15	Rat	12746320

glutamate decarboxylase	4.1.1.15	Rat	15210535
glutamate decarboxylase	4.1.1.15	Rat	15581395
glutamate decarboxylase	4.1.1.15	Rat	1697032
glutamate decarboxylase	4.1.1.15	Rat	17044036
glutamate decarboxylase	4.1.1.15	Rat	1976015
glutamate decarboxylase	4.1.1.15	Rat	2180326
glutamate decarboxylase	4.1.1.15	Rat	2735448
glutamate decarboxylase	4.1.1.15	Rat	2857768
glutamate decarboxylase	4.1.1.15	Rat	3896834
glutamate decarboxylase	4.1.1.15	Rat	6975381
glutamate decarboxylase	4.1.1.15	Rat	7702443
glutamate decarboxylase	4.1.1.15	Rat	7885536
glutamate decarboxylase	4.1.1.15	Rat	8302162
glutamate decarboxylase	4.1.1.15	Rat	9011754
glutamate decarboxylase	4.1.1.15	Rat	9053794
ornithine decarboxylase	4.1.1.17	Rat	10069996
ornithine decarboxylase	4.1.1.17	Rat	10216947
ornithine decarboxylase	4.1.1.17	Rat	10319188
ornithine decarboxylase	4.1.1.17	Rat	10320037
ornithine decarboxylase	4.1.1.17	Rat	10321508
ornithine decarboxylase	4.1.1.17	Rat	10430664
ornithine decarboxylase	4.1.1.17	Rat	10453061
ornithine decarboxylase	4.1.1.17	Rat	10456943
ornithine decarboxylase	4.1.1.17	Rat	10473083
ornithine decarboxylase	4.1.1.17	Rat	10485326
ornithine decarboxylase	4.1.1.17	Rat	10544213
ornithine decarboxylase	4.1.1.17	Rat	10550568
ornithine decarboxylase	4.1.1.17	Rat	10564512
ornithine decarboxylase	4.1.1.17	Rat	10589756
ornithine decarboxylase	4.1.1.17	Rat	10593613
ornithine decarboxylase	4.1.1.17	Rat	10607762
ornithine decarboxylase	4.1.1.17	Rat	10629084
ornithine decarboxylase	4.1.1.17	Rat	10712236
ornithine decarboxylase	4.1.1.17	Rat	10713131
ornithine decarboxylase	4.1.1.17	Rat	10760944
ornithine decarboxylase	4.1.1.17	Rat	10772389
ornithine decarboxylase	4.1.1.17	Rat	10816435
ornithine decarboxylase	4.1.1.17	Rat	10817834
ornithine decarboxylase	4.1.1.17	Rat	10882097
ornithine decarboxylase	4.1.1.17	Rat	10931831
ornithine decarboxylase	4.1.1.17	Rat	10940513
ornithine decarboxylase	4.1.1.17	Rat	10965017
ornithine decarboxylase	4.1.1.17	Rat	11003584

ornithine decarboxylase	4.1.1.17	Rat	11085920
ornithine decarboxylase	4.1.1.17	Rat	11095648
ornithine decarboxylase	4.1.1.17	Rat	11137705
ornithine decarboxylase	4.1.1.17	Rat	11180396
ornithine decarboxylase	4.1.1.17	Rat	11235918
ornithine decarboxylase	4.1.1.17	Rat	11355005
ornithine decarboxylase	4.1.1.17	Rat	11376395
ornithine decarboxylase	4.1.1.17	Rat	11408092
ornithine decarboxylase	4.1.1.17	Rat	11408253
ornithine decarboxylase	4.1.1.17	Rat	11408542
ornithine decarboxylase	4.1.1.17	Rat	11413269
ornithine decarboxylase	4.1.1.17	Rat	11540835
ornithine decarboxylase	4.1.1.17	Rat	11558274
ornithine decarboxylase	4.1.1.17	Rat	11736657
ornithine decarboxylase	4.1.1.17	Rat	11782361
ornithine decarboxylase	4.1.1.17	Rat	11852055
ornithine decarboxylase	4.1.1.17	Rat	11883715
ornithine decarboxylase	4.1.1.17	Rat	11922393
ornithine decarboxylase	4.1.1.17	Rat	11923270
ornithine decarboxylase	4.1.1.17	Rat	11964084
ornithine decarboxylase	4.1.1.17	Rat	11997243
ornithine decarboxylase	4.1.1.17	Rat	12054570
ornithine decarboxylase	4.1.1.17	Rat	12105848
ornithine decarboxylase	4.1.1.17	Rat	12148577
ornithine decarboxylase	4.1.1.17	Rat	12355213
ornithine decarboxylase	4.1.1.17	Rat	12452334
ornithine decarboxylase	4.1.1.17	Rat	12497077
ornithine decarboxylase	4.1.1.17	Rat	12527115
ornithine decarboxylase	4.1.1.17	Rat	12663506
ornithine decarboxylase	4.1.1.17	Rat	12716308
ornithine decarboxylase	4.1.1.17	Rat	12716758
ornithine decarboxylase	4.1.1.17	Rat	12766050
ornithine decarboxylase	4.1.1.17	Rat	12816757
ornithine decarboxylase	4.1.1.17	Rat	12856719
ornithine decarboxylase	4.1.1.17	Rat	12882169
ornithine decarboxylase	4.1.1.17	Rat	1289667
ornithine decarboxylase	4.1.1.17	Rat	1324153
ornithine decarboxylase	4.1.1.17	Rat	1360468
ornithine decarboxylase	4.1.1.17	Rat	1397089
ornithine decarboxylase	4.1.1.17	Rat	1407701
ornithine decarboxylase	4.1.1.17	Rat	1409247
ornithine decarboxylase	4.1.1.17	Rat	1417733
ornithine decarboxylase	4.1.1.17	Rat	14769544

ornithine decarboxylase	4.1.1.17	Rat	1495349
ornithine decarboxylase	4.1.1.17	Rat	15002659
ornithine decarboxylase	4.1.1.17	Rat	1511780
ornithine decarboxylase	4.1.1.17	Rat	15120115
ornithine decarboxylase	4.1.1.17	Rat	15180186
ornithine decarboxylase	4.1.1.17	Rat	1521915
ornithine decarboxylase	4.1.1.17	Rat	15223770
ornithine decarboxylase	4.1.1.17	Rat	15228220
ornithine decarboxylase	4.1.1.17	Rat	15233741
ornithine decarboxylase	4.1.1.17	Rat	15247138
ornithine decarboxylase	4.1.1.17	Rat	15296840
ornithine decarboxylase	4.1.1.17	Rat	15306645
ornithine decarboxylase	4.1.1.17	Rat	15355308
ornithine decarboxylase	4.1.1.17	Rat	15514084
ornithine decarboxylase	4.1.1.17	Rat	15538383
ornithine decarboxylase	4.1.1.17	Rat	15539331
ornithine decarboxylase	4.1.1.17	Rat	1563337
ornithine decarboxylase	4.1.1.17	Rat	15697240
ornithine decarboxylase	4.1.1.17	Rat	1569947
ornithine decarboxylase	4.1.1.17	Rat	15716048
ornithine decarboxylase	4.1.1.17	Rat	15843384
ornithine decarboxylase	4.1.1.17	Rat	1584960
ornithine decarboxylase	4.1.1.17	Rat	1590311
ornithine decarboxylase	4.1.1.17	Rat	1601800
ornithine decarboxylase	4.1.1.17	Rat	16091008
ornithine decarboxylase	4.1.1.17	Rat	16168128
ornithine decarboxylase	4.1.1.17	Rat	16170669
ornithine decarboxylase	4.1.1.17	Rat	16181115
ornithine decarboxylase	4.1.1.17	Rat	16223706
ornithine decarboxylase	4.1.1.17	Rat	16230862
ornithine decarboxylase	4.1.1.17	Rat	16290266
ornithine decarboxylase	4.1.1.17	Rat	16342411
ornithine decarboxylase	4.1.1.17	Rat	1641775
ornithine decarboxylase	4.1.1.17	Rat	16445292
ornithine decarboxylase	4.1.1.17	Rat	1655898
ornithine decarboxylase	4.1.1.17	Rat	16568078
ornithine decarboxylase	4.1.1.17	Rat	16630547
ornithine decarboxylase	4.1.1.17	Rat	16662219
ornithine decarboxylase	4.1.1.17	Rat	16666570
ornithine decarboxylase	4.1.1.17	Rat	16678846
ornithine decarboxylase	4.1.1.17	Rat	16729674
ornithine decarboxylase	4.1.1.17	Rat	1697882
ornithine decarboxylase	4.1.1.17	Rat	1703390

ornithine decarboxylase	4.1.1.17	Rat	1733364
ornithine decarboxylase	4.1.1.17	Rat	17407445
ornithine decarboxylase	4.1.1.17	Rat	1745018
ornithine decarboxylase	4.1.1.17	Rat	1782416
ornithine decarboxylase	4.1.1.17	Rat	1814556
ornithine decarboxylase	4.1.1.17	Rat	1814755
ornithine decarboxylase	4.1.1.17	Rat	1831810
ornithine decarboxylase	4.1.1.17	Rat	1846091
ornithine decarboxylase	4.1.1.17	Rat	1878921
ornithine decarboxylase	4.1.1.17	Rat	1884248
ornithine decarboxylase	4.1.1.17	Rat	1892753
ornithine decarboxylase	4.1.1.17	Rat	1900385
ornithine decarboxylase	4.1.1.17	Rat	1932775
ornithine decarboxylase	4.1.1.17	Rat	1940203
ornithine decarboxylase	4.1.1.17	Rat	1962522
ornithine decarboxylase	4.1.1.17	Rat	196870
ornithine decarboxylase	4.1.1.17	Rat	1997184
ornithine decarboxylase	4.1.1.17	Rat	2006469
ornithine decarboxylase	4.1.1.17	Rat	2009332
ornithine decarboxylase	4.1.1.17	Rat	2019760
ornithine decarboxylase	4.1.1.17	Rat	203259
ornithine decarboxylase	4.1.1.17	Rat	2051775
ornithine decarboxylase	4.1.1.17	Rat	2088816
ornithine decarboxylase	4.1.1.17	Rat	2118148
ornithine decarboxylase	4.1.1.17	Rat	2148056
ornithine decarboxylase	4.1.1.17	Rat	2160044
ornithine decarboxylase	4.1.1.17	Rat	2197525
ornithine decarboxylase	4.1.1.17	Rat	2210666
ornithine decarboxylase	4.1.1.17	Rat	2243540
ornithine decarboxylase	4.1.1.17	Rat	2293084
ornithine decarboxylase	4.1.1.17	Rat	2296762
ornithine decarboxylase	4.1.1.17	Rat	2298913
ornithine decarboxylase	4.1.1.17	Rat	2409817
ornithine decarboxylase	4.1.1.17	Rat	2469492
ornithine decarboxylase	4.1.1.17	Rat	2472814
ornithine decarboxylase	4.1.1.17	Rat	2493794
ornithine decarboxylase	4.1.1.17	Rat	2494779
ornithine decarboxylase	4.1.1.17	Rat	2497460
ornithine decarboxylase	4.1.1.17	Rat	2497556
ornithine decarboxylase	4.1.1.17	Rat	2505399
ornithine decarboxylase	4.1.1.17	Rat	2505959
ornithine decarboxylase	4.1.1.17	Rat	2507383
ornithine decarboxylase	4.1.1.17	Rat	2507471

ornithine decarboxylase	4.1.1.17	Rat	2525760
ornithine decarboxylase	4.1.1.17	Rat	2553150
ornithine decarboxylase	4.1.1.17	Rat	2610929
ornithine decarboxylase	4.1.1.17	Rat	2651129
ornithine decarboxylase	4.1.1.17	Rat	2699646
ornithine decarboxylase	4.1.1.17	Rat	2754510
ornithine decarboxylase	4.1.1.17	Rat	2775206
ornithine decarboxylase	4.1.1.17	Rat	2829727
ornithine decarboxylase	4.1.1.17	Rat	2840461
ornithine decarboxylase	4.1.1.17	Rat	2915649
ornithine decarboxylase	4.1.1.17	Rat	2916900
ornithine decarboxylase	4.1.1.17	Rat	2977154
ornithine decarboxylase	4.1.1.17	Rat	2979197
ornithine decarboxylase	4.1.1.17	Rat	2986953
ornithine decarboxylase	4.1.1.17	Rat	3004707
ornithine decarboxylase	4.1.1.17	Rat	3009424
ornithine decarboxylase	4.1.1.17	Rat	3022056
ornithine decarboxylase	4.1.1.17	Rat	3036091
ornithine decarboxylase	4.1.1.17	Rat	3037250
ornithine decarboxylase	4.1.1.17	Rat	3040821
ornithine decarboxylase	4.1.1.17	Rat	3082276
ornithine decarboxylase	4.1.1.17	Rat	3084209
ornithine decarboxylase	4.1.1.17	Rat	3084872
ornithine decarboxylase	4.1.1.17	Rat	3086160
ornithine decarboxylase	4.1.1.17	Rat	3092827
ornithine decarboxylase	4.1.1.17	Rat	3093095
ornithine decarboxylase	4.1.1.17	Rat	3096557
ornithine decarboxylase	4.1.1.17	Rat	3100897
ornithine decarboxylase	4.1.1.17	Rat	3102397
ornithine decarboxylase	4.1.1.17	Rat	3105968
ornithine decarboxylase	4.1.1.17	Rat	3106075
ornithine decarboxylase	4.1.1.17	Rat	3108666
ornithine decarboxylase	4.1.1.17	Rat	3109979
ornithine decarboxylase	4.1.1.17	Rat	3109985
ornithine decarboxylase	4.1.1.17	Rat	3111384
ornithine decarboxylase	4.1.1.17	Rat	3113732
ornithine decarboxylase	4.1.1.17	Rat	3117720
ornithine decarboxylase	4.1.1.17	Rat	3121457
ornithine decarboxylase	4.1.1.17	Rat	3122042
ornithine decarboxylase	4.1.1.17	Rat	3129184
ornithine decarboxylase	4.1.1.17	Rat	3130188
ornithine decarboxylase	4.1.1.17	Rat	3139441
ornithine decarboxylase	4.1.1.17	Rat	3141045

ornithine decarboxylase	4.1.1.17	Rat	3143046
ornithine decarboxylase	4.1.1.17	Rat	3180091
ornithine decarboxylase	4.1.1.17	Rat	3279036
ornithine decarboxylase	4.1.1.17	Rat	3328430
ornithine decarboxylase	4.1.1.17	Rat	3356404
ornithine decarboxylase	4.1.1.17	Rat	3403538
ornithine decarboxylase	4.1.1.17	Rat	3443298
ornithine decarboxylase	4.1.1.17	Rat	3538740
ornithine decarboxylase	4.1.1.17	Rat	3548994
ornithine decarboxylase	4.1.1.17	Rat	3661847
ornithine decarboxylase	4.1.1.17	Rat	3672608
ornithine decarboxylase	4.1.1.17	Rat	3688216
ornithine decarboxylase	4.1.1.17	Rat	3729588
ornithine decarboxylase	4.1.1.17	Rat	3743773
ornithine decarboxylase	4.1.1.17	Rat	3753036
ornithine decarboxylase	4.1.1.17	Rat	3775249
ornithine decarboxylase	4.1.1.17	Rat	3794781
ornithine decarboxylase	4.1.1.17	Rat	3857388
ornithine decarboxylase	4.1.1.17	Rat	3901680
ornithine decarboxylase	4.1.1.17	Rat	3905315
ornithine decarboxylase	4.1.1.17	Rat	3921243
ornithine decarboxylase	4.1.1.17	Rat	3926303
ornithine decarboxylase	4.1.1.17	Rat	3930649
ornithine decarboxylase	4.1.1.17	Rat	3931300
ornithine decarboxylase	4.1.1.17	Rat	3934106
ornithine decarboxylase	4.1.1.17	Rat	3999751
ornithine decarboxylase	4.1.1.17	Rat	4029343
ornithine decarboxylase	4.1.1.17	Rat	4053280
ornithine decarboxylase	4.1.1.17	Rat	497279
ornithine decarboxylase	4.1.1.17	Rat	6124275
ornithine decarboxylase	4.1.1.17	Rat	6178351
ornithine decarboxylase	4.1.1.17	Rat	6190690
ornithine decarboxylase	4.1.1.17	Rat	6192925
ornithine decarboxylase	4.1.1.17	Rat	6223191
ornithine decarboxylase	4.1.1.17	Rat	6256169
ornithine decarboxylase	4.1.1.17	Rat	6272311
ornithine decarboxylase	4.1.1.17	Rat	6307502
ornithine decarboxylase	4.1.1.17	Rat	6365078
ornithine decarboxylase	4.1.1.17	Rat	6432312
ornithine decarboxylase	4.1.1.17	Rat	6432848
ornithine decarboxylase	4.1.1.17	Rat	6439208
ornithine decarboxylase	4.1.1.17	Rat	6440787
ornithine decarboxylase	4.1.1.17	Rat	6445842

ornithine decarboxylase	4.1.1.17	Rat	6467454
ornithine decarboxylase	4.1.1.17	Rat	6571411
ornithine decarboxylase	4.1.1.17	Rat	6591862
ornithine decarboxylase	4.1.1.17	Rat	6624798
ornithine decarboxylase	4.1.1.17	Rat	6692409
ornithine decarboxylase	4.1.1.17	Rat	6696980
ornithine decarboxylase	4.1.1.17	Rat	6721578
ornithine decarboxylase	4.1.1.17	Rat	6750139
ornithine decarboxylase	4.1.1.17	Rat	6812570
ornithine decarboxylase	4.1.1.17	Rat	6813460
ornithine decarboxylase	4.1.1.17	Rat	6865777
ornithine decarboxylase	4.1.1.17	Rat	6891933
ornithine decarboxylase	4.1.1.17	Rat	7093948
ornithine decarboxylase	4.1.1.17	Rat	7104206
ornithine decarboxylase	4.1.1.17	Rat	7159401
ornithine decarboxylase	4.1.1.17	Rat	7205597
ornithine decarboxylase	4.1.1.17	Rat	7310281
ornithine decarboxylase	4.1.1.17	Rat	7381752
ornithine decarboxylase	4.1.1.17	Rat	7426404
ornithine decarboxylase	4.1.1.17	Rat	7488168
ornithine decarboxylase	4.1.1.17	Rat	7498733
ornithine decarboxylase	4.1.1.17	Rat	7525612
ornithine decarboxylase	4.1.1.17	Rat	7616440
ornithine decarboxylase	4.1.1.17	Rat	7628376
ornithine decarboxylase	4.1.1.17	Rat	7656288
ornithine decarboxylase	4.1.1.17	Rat	7718766
ornithine decarboxylase	4.1.1.17	Rat	7813017
ornithine decarboxylase	4.1.1.17	Rat	7823874
ornithine decarboxylase	4.1.1.17	Rat	7865470
ornithine decarboxylase	4.1.1.17	Rat	7872745
ornithine decarboxylase	4.1.1.17	Rat	7874572
ornithine decarboxylase	4.1.1.17	Rat	7895420
ornithine decarboxylase	4.1.1.17	Rat	7929646
ornithine decarboxylase	4.1.1.17	Rat	7943199
ornithine decarboxylase	4.1.1.17	Rat	7951165
ornithine decarboxylase	4.1.1.17	Rat	7965748
ornithine decarboxylase	4.1.1.17	Rat	7972938
ornithine decarboxylase	4.1.1.17	Rat	7981636
ornithine decarboxylase	4.1.1.17	Rat	8010156
ornithine decarboxylase	4.1.1.17	Rat	8028020
ornithine decarboxylase	4.1.1.17	Rat	8065308
ornithine decarboxylase	4.1.1.17	Rat	8095973
ornithine decarboxylase	4.1.1.17	Rat	8110472

ornithine decarboxylase	4.1.1.17	Rat	8140036
ornithine decarboxylase	4.1.1.17	Rat	8141779
ornithine decarboxylase	4.1.1.17	Rat	8152342
ornithine decarboxylase	4.1.1.17	Rat	8185631
ornithine decarboxylase	4.1.1.17	Rat	8190721
ornithine decarboxylase	4.1.1.17	Rat	8242794
ornithine decarboxylase	4.1.1.17	Rat	8344985
ornithine decarboxylase	4.1.1.17	Rat	8368314
ornithine decarboxylase	4.1.1.17	Rat	8374143
ornithine decarboxylase	4.1.1.17	Rat	8419528
ornithine decarboxylase	4.1.1.17	Rat	8447420
ornithine decarboxylase	4.1.1.17	Rat	8453677
ornithine decarboxylase	4.1.1.17	Rat	8462726
ornithine decarboxylase	4.1.1.17	Rat	8465553
ornithine decarboxylase	4.1.1.17	Rat	8478959
ornithine decarboxylase	4.1.1.17	Rat	8501729
ornithine decarboxylase	4.1.1.17	Rat	8538189
ornithine decarboxylase	4.1.1.17	Rat	8549635
ornithine decarboxylase	4.1.1.17	Rat	8572176
ornithine decarboxylase	4.1.1.17	Rat	8660289
ornithine decarboxylase	4.1.1.17	Rat	8693031
ornithine decarboxylase	4.1.1.17	Rat	8707896
ornithine decarboxylase	4.1.1.17	Rat	8727257
ornithine decarboxylase	4.1.1.17	Rat	8760120
ornithine decarboxylase	4.1.1.17	Rat	8760129
ornithine decarboxylase	4.1.1.17	Rat	8768305
ornithine decarboxylase	4.1.1.17	Rat	8777294
ornithine decarboxylase	4.1.1.17	Rat	8814137
ornithine decarboxylase	4.1.1.17	Rat	8848835
ornithine decarboxylase	4.1.1.17	Rat	8858522
ornithine decarboxylase	4.1.1.17	Rat	8878500
ornithine decarboxylase	4.1.1.17	Rat	8882155
ornithine decarboxylase	4.1.1.17	Rat	8912847
ornithine decarboxylase	4.1.1.17	Rat	8944705
ornithine decarboxylase	4.1.1.17	Rat	9009157
ornithine decarboxylase	4.1.1.17	Rat	9016399
ornithine decarboxylase	4.1.1.17	Rat	9017896
ornithine decarboxylase	4.1.1.17	Rat	9022291
ornithine decarboxylase	4.1.1.17	Rat	9024941
ornithine decarboxylase	4.1.1.17	Rat	9063811
ornithine decarboxylase	4.1.1.17	Rat	9073141
ornithine decarboxylase	4.1.1.17	Rat	9134011
ornithine decarboxylase	4.1.1.17	Rat	9142900

ornithine decarboxylase	4.1.1.17	Rat	9191978
ornithine decarboxylase	4.1.1.17	Rat	9213218
ornithine decarboxylase	4.1.1.17	Rat	9223345
ornithine decarboxylase	4.1.1.17	Rat	9224728
ornithine decarboxylase	4.1.1.17	Rat	9251105
ornithine decarboxylase	4.1.1.17	Rat	9252524
ornithine decarboxylase	4.1.1.17	Rat	9294258
ornithine decarboxylase	4.1.1.17	Rat	9368191
ornithine decarboxylase	4.1.1.17	Rat	9371082
ornithine decarboxylase	4.1.1.17	Rat	9454972
ornithine decarboxylase	4.1.1.17	Rat	9458728
ornithine decarboxylase	4.1.1.17	Rat	9468098
ornithine decarboxylase	4.1.1.17	Rat	9495243
ornithine decarboxylase	4.1.1.17	Rat	9525811
ornithine decarboxylase	4.1.1.17	Rat	9590135
ornithine decarboxylase	4.1.1.17	Rat	9609384
ornithine decarboxylase	4.1.1.17	Rat	9612269
ornithine decarboxylase	4.1.1.17	Rat	9624108
ornithine decarboxylase	4.1.1.17	Rat	9648877
ornithine decarboxylase	4.1.1.17	Rat	9685330
ornithine decarboxylase	4.1.1.17	Rat	9688665
ornithine decarboxylase	4.1.1.17	Rat	9769382
ornithine decarboxylase	4.1.1.17	Rat	9772292
ornithine decarboxylase	4.1.1.17	Rat	9795249
ornithine decarboxylase	4.1.1.17	Rat	9806166
ornithine decarboxylase	4.1.1.17	Rat	9829706
ornithine decarboxylase	4.1.1.17	Rat	9868187
ornithine decarboxylase	4.1.1.17	Rat	9884080
ornithine decarboxylase	4.1.1.17	Rat	9890191
histidine decarboxylase	4.1.1.22	Rat	10652359
histidine decarboxylase	4.1.1.22	Rat	10843737
histidine decarboxylase	4.1.1.22	Rat	11566179
histidine decarboxylase	4.1.1.22	Rat	11827977
histidine decarboxylase	4.1.1.22	Rat	12875237
histidine decarboxylase	4.1.1.22	Rat	12960041
histidine decarboxylase	4.1.1.22	Rat	15114681
histidine decarboxylase	4.1.1.22	Rat	16203768
histidine decarboxylase	4.1.1.22	Rat	16564918
histidine decarboxylase	4.1.1.22	Rat	2889701
histidine decarboxylase	4.1.1.22	Rat	3587507
histidine decarboxylase	4.1.1.22	Rat	7742310
histidine decarboxylase	4.1.1.22	Rat	9041686
aromatic-L-amino-acid decarboxylase	4.1.1.28	Rat	10080715

aromatic-L-amino-acid decarboxylase	4.1.1.28	Rat	10608659
aromatic-L-amino-acid decarboxylase	4.1.1.28	Rat	12235825
aromatic-L-amino-acid decarboxylase	4.1.1.28	Rat	15684695
aromatic-L-amino-acid decarboxylase	4.1.1.28	Rat	15900211
aromatic-L-amino-acid decarboxylase	4.1.1.28	Rat	15927700
aromatic-L-amino-acid decarboxylase	4.1.1.28	Rat	16164646
aromatic-L-amino-acid decarboxylase	4.1.1.28	Rat	17017570
aromatic-L-amino-acid decarboxylase	4.1.1.28	Rat	1729407
aromatic-L-amino-acid decarboxylase	4.1.1.28	Rat	6968497
aromatic-L-amino-acid decarboxylase	4.1.1.28	Rat	7651438
aromatic-L-amino-acid decarboxylase	4.1.1.28	Rat	9625353
sulfinoalanine decarboxylase	4.1.1.29	Rat	10993179
phosphoenolpyruvate carboxykinase (GTP)	4.1.1.32	Rat	11053047
phosphoenolpyruvate carboxykinase (GTP)	4.1.1.32	Rat	11700062
phosphoenolpyruvate carboxykinase (GTP)	4.1.1.32	Rat	11728630
phosphoenolpyruvate carboxykinase (GTP)	4.1.1.32	Rat	11741859
phosphoenolpyruvate carboxykinase (GTP)	4.1.1.32	Rat	11872659
phosphoenolpyruvate carboxykinase (GTP)	4.1.1.32	Rat	11959679
phosphoenolpyruvate carboxykinase (GTP)	4.1.1.32	Rat	12485530
phosphoenolpyruvate carboxykinase (GTP)	4.1.1.32	Rat	12925798
phosphoenolpyruvate carboxykinase (GTP)	4.1.1.32	Rat	14739078
phosphoenolpyruvate carboxykinase (GTP)	4.1.1.32	Rat	15733733
phosphoenolpyruvate carboxykinase (GTP)	4.1.1.32	Rat	15983413
phosphoenolpyruvate carboxykinase (GTP)	4.1.1.32	Rat	16132948
phosphoenolpyruvate carboxykinase (GTP)	4.1.1.32	Rat	16324924
phosphoenolpyruvate carboxykinase (GTP)	4.1.1.32	Rat	16330542
phosphoenolpyruvate carboxykinase (GTP)	4.1.1.32	Rat	16375695
phosphoenolpyruvate carboxykinase (GTP)	4.1.1.32	Rat	16458327
phosphoenolpyruvate carboxykinase (GTP)	4.1.1.32	Rat	1701430
phosphoenolpyruvate carboxykinase (GTP)	4.1.1.32	Rat	17097062
phosphoenolpyruvate carboxykinase (GTP)	4.1.1.32	Rat	1720862
phosphoenolpyruvate carboxykinase (GTP)	4.1.1.32	Rat	17446233
phosphoenolpyruvate carboxykinase (GTP)	4.1.1.32	Rat	2919162
phosphoenolpyruvate carboxykinase (GTP)	4.1.1.32	Rat	3023262
phosphoenolpyruvate carboxykinase (GTP)	4.1.1.32	Rat	3068502
phosphoenolpyruvate carboxykinase (GTP)	4.1.1.32	Rat	4053567
phosphoenolpyruvate carboxykinase (GTP)	4.1.1.32	Rat	6440018
phosphoenolpyruvate carboxykinase (GTP)	4.1.1.32	Rat	7854322
phosphoenolpyruvate carboxykinase (GTP)	4.1.1.32	Rat	8902188
phosphoenolpyruvate carboxykinase (GTP)	4.1.1.32	Rat	9214454
phosphoenolpyruvate carboxykinase (GTP)	4.1.1.32	Rat	9242918
phosphoenolpyruvate carboxykinase (GTP)	4.1.1.32	Rat	9762796
adenosylmethionine decarboxylase	4.1.1.50	Rat	10216947

adenosylmethionine decarboxylase	4.1.1.50	Rat	10378277
adenosylmethionine decarboxylase	4.1.1.50	Rat	10413038
adenosylmethionine decarboxylase	4.1.1.50	Rat	10467042
adenosylmethionine decarboxylase	4.1.1.50	Rat	10713131
adenosylmethionine decarboxylase	4.1.1.50	Rat	10949915
adenosylmethionine decarboxylase	4.1.1.50	Rat	11076965
adenosylmethionine decarboxylase	4.1.1.50	Rat	11085920
adenosylmethionine decarboxylase	4.1.1.50	Rat	11348531
adenosylmethionine decarboxylase	4.1.1.50	Rat	11390378
adenosylmethionine decarboxylase	4.1.1.50	Rat	11526206
adenosylmethionine decarboxylase	4.1.1.50	Rat	11583148
adenosylmethionine decarboxylase	4.1.1.50	Rat	11923270
adenosylmethionine decarboxylase	4.1.1.50	Rat	12674502
adenosylmethionine decarboxylase	4.1.1.50	Rat	14618239
adenosylmethionine decarboxylase	4.1.1.50	Rat	15150268
adenosylmethionine decarboxylase	4.1.1.50	Rat	15821146
adenosylmethionine decarboxylase	4.1.1.50	Rat	16372273
adenosylmethionine decarboxylase	4.1.1.50	Rat	16423999
adenosylmethionine decarboxylase	4.1.1.50	Rat	16515461
adenosylmethionine decarboxylase	4.1.1.50	Rat	16642382
adenosylmethionine decarboxylase	4.1.1.50	Rat	16941339
adenosylmethionine decarboxylase	4.1.1.50	Rat	2013278
adenosylmethionine decarboxylase	4.1.1.50	Rat	2209170
adenosylmethionine decarboxylase	4.1.1.50	Rat	2775206
adenosylmethionine decarboxylase	4.1.1.50	Rat	3250232
adenosylmethionine decarboxylase	4.1.1.50	Rat	4062886
adenosylmethionine decarboxylase	4.1.1.50	Rat	7789170
adenosylmethionine decarboxylase	4.1.1.50	Rat	7945201
adenosylmethionine decarboxylase	4.1.1.50	Rat	8142949
adenosylmethionine decarboxylase	4.1.1.50	Rat	8178573
adenosylmethionine decarboxylase	4.1.1.50	Rat	8198469
adenosylmethionine decarboxylase	4.1.1.50	Rat	8353934
adenosylmethionine decarboxylase	4.1.1.50	Rat	8760129
adenosylmethionine decarboxylase	4.1.1.50	Rat	8814137
adenosylmethionine decarboxylase	4.1.1.50	Rat	8973561
adenosylmethionine decarboxylase	4.1.1.50	Rat	9435790
adenosylmethionine decarboxylase	4.1.1.50	Rat	9841864
adenosylmethionine decarboxylase	4.1.1.50	Rat	9879883
fructose-bisphosphate aldolase	4.1.2.13	Rat	10498814
fructose-bisphosphate aldolase	4.1.2.13	Rat	12020659
fructose-bisphosphate aldolase	4.1.2.13	Rat	12876349
fructose-bisphosphate aldolase	4.1.2.13	Rat	15142555
fructose-bisphosphate aldolase	4.1.2.13	Rat	15869466

fructose-bisphosphate aldolase	4.1.2.13	Rat	16502329
fructose-bisphosphate aldolase	4.1.2.13	Rat	1658253
fructose-bisphosphate aldolase	4.1.2.13	Rat	1894606
fructose-bisphosphate aldolase	4.1.2.13	Rat	6440018
fructose-bisphosphate aldolase	4.1.2.13	Rat	678439
fructose-bisphosphate aldolase	4.1.2.13	Rat	7388140
fructose-bisphosphate aldolase	4.1.2.13	Rat	7786768
fructose-bisphosphate aldolase	4.1.2.13	Rat	8015399
fructose-bisphosphate aldolase	4.1.2.13	Rat	8913875
fructose-bisphosphate aldolase	4.1.2.13	Rat	9163906
fructose-bisphosphate aldolase	4.1.2.13	Rat	9473304
carbonate dehydratase	4.2.1.1	Rat	16051345
carbonate dehydratase	4.2.1.1	Rat	16310354
carbonate dehydratase	4.2.1.1	Rat	1908243
carbonate dehydratase	4.2.1.1	Rat	2117006
carbonate dehydratase	4.2.1.1	Rat	3128444
carbonate dehydratase	4.2.1.1	Rat	6408083
carbonate dehydratase	4.2.1.1	Rat	6772280
carbonate dehydratase	4.2.1.1	Rat	6776264
carbonate dehydratase	4.2.1.1	Rat	6791257
carbonate dehydratase	4.2.1.1	Rat	7899732
carbonate dehydratase	4.2.1.1	Rat	9186493
carbonate dehydratase	4.2.1.1	Rat	9486145
carbonate dehydratase	4.2.1.1	Rat	9882455
phosphopyruvate hydratase	4.2.1.11	Rat	15459207
phosphopyruvate hydratase	4.2.1.11	Rat	17437631
phosphopyruvate hydratase	4.2.1.11	Rat	8651685
cystathionine beta-synthase	4.2.1.22	Rat	11051561
cystathionine beta-synthase	4.2.1.22	Rat	11106665
cystathionine beta-synthase	4.2.1.22	Rat	12198128
cystathionine beta-synthase	4.2.1.22	Rat	15581575
cystathionine beta-synthase	4.2.1.22	Rat	15642325
cystathionine beta-synthase	4.2.1.22	Rat	16096271
cystathionine beta-synthase	4.2.1.22	Rat	16769053
cystathionine beta-synthase	4.2.1.22	Rat	17629356
porphobilinogen synthase	4.2.1.24	Rat	10634305
porphobilinogen synthase	4.2.1.24	Rat	10787385
porphobilinogen synthase	4.2.1.24	Rat	15141099
porphobilinogen synthase	4.2.1.24	Rat	15259603
porphobilinogen synthase	4.2.1.24	Rat	1526942
porphobilinogen synthase	4.2.1.24	Rat	16819823
porphobilinogen synthase	4.2.1.24	Rat	1959865
porphobilinogen synthase	4.2.1.24	Rat	2050126

porphobilinogen synthase	4.2.1.24	Rat	2317819
porphobilinogen synthase	4.2.1.24	Rat	3009001
porphobilinogen synthase	4.2.1.24	Rat	3755290
porphobilinogen synthase	4.2.1.24	Rat	3840094
porphobilinogen synthase	4.2.1.24	Rat	3966797
porphobilinogen synthase	4.2.1.24	Rat	4265023
porphobilinogen synthase	4.2.1.24	Rat	6547609
porphobilinogen synthase	4.2.1.24	Rat	6688350
porphobilinogen synthase	4.2.1.24	Rat	6873612
porphobilinogen synthase	4.2.1.24	Rat	7436670
aconitate hydratase	4.2.1.3	Rat	11295257
aconitate hydratase	4.2.1.3	Rat	11329290
aconitate hydratase	4.2.1.3	Rat	15149735
aconitate hydratase	4.2.1.3	Rat	15543948
aconitate hydratase	4.2.1.3	Rat	16094633
aconitate hydratase	4.2.1.3	Rat	16201454
aconitate hydratase	4.2.1.3	Rat	7589784
aconitate hydratase	4.2.1.3	Rat	8115279
6-pyruvoyltetrahydropterin synthase	4.2.3.12	Rat	11022034
6-pyruvoyltetrahydropterin synthase	4.2.3.12	Rat	11744995
6-pyruvoyltetrahydropterin synthase	4.2.3.12	Rat	11778454
6-pyruvoyltetrahydropterin synthase	4.2.3.12	Rat	7545485
6-pyruvoyltetrahydropterin synthase	4.2.3.12	Rat	8307017
6-pyruvoyltetrahydropterin synthase	4.2.3.12	Rat	9788822
cystathionine gamma-lyase	4.4.1.1	Rat	10960449
cystathionine gamma-lyase	4.4.1.1	Rat	15038791
cystathionine gamma-lyase	4.4.1.1	Rat	15347670
cystathionine gamma-lyase	4.4.1.1	Rat	16786305
cystathionine gamma-lyase	4.4.1.1	Rat	16946488
cystathionine gamma-lyase	4.4.1.1	Rat	17095121
cystathionine gamma-lyase	4.4.1.1	Rat	17525332
cystathionine gamma-lyase	4.4.1.1	Rat	8973544
leukotriene-C4 synthase	4.4.1.20	Rat	10222453
leukotriene-C4 synthase	4.4.1.20	Rat	11964666
leukotriene-C4 synthase	4.4.1.20	Rat	15730873
leukotriene-C4 synthase	4.4.1.20	Rat	17397868
leukotriene-C4 synthase	4.4.1.20	Rat	9431445
UDP-N-acetylglucosamine 2-epimerase	5.1.3.14	Rat	10334995
UDP-N-acetylglucosamine 2-epimerase	5.1.3.14	Rat	11326336
UDP-N-acetylglucosamine 2-epimerase	5.1.3.14	Rat	11956597
UDP-N-acetylglucosamine 2-epimerase	5.1.3.14	Rat	12927803
UDP-N-acetylglucosamine 2-epimerase	5.1.3.14	Rat	15135418
UDP-N-acetylglucosamine 2-epimerase	5.1.3.14	Rat	17565386

UDP-N-acetylglucosamine 2-epimerase	5.1.3.14	Rat	3780977
UDP-N-acetylglucosamine 2-epimerase	5.1.3.14	Rat	8439453
UDP-N-acetylglucosamine 2-epimerase	5.1.3.14	Rat	9305888
UDP-glucose 4-epimerase	5.1.3.2	Rat	1149741
steroid Delta-isomerase	5.3.3.1	Rat	239964
phosphoglycerate mutase	5.4.2.1	Rat	15181008
phosphoglycerate mutase	5.4.2.1	Rat	17204863
phosphoglycerate mutase	5.4.2.1	Rat	2830218
phosphoglucomutase	5.4.2.2	Rat	11102370
phosphoglucomutase	5.4.2.2	Rat	1149741
phosphoglucomutase	5.4.2.2	Rat	12026175
phosphoglucomutase	5.4.2.2	Rat	12791685
phosphoglucomutase	5.4.2.2	Rat	15378030
phosphoglucomutase	5.4.2.2	Rat	15996095
phosphoglucomutase	5.4.2.2	Rat	16046289
lanosterol synthase	5.4.99.7	Rat	11995928
lanosterol synthase	5.4.99.7	Rat	15763540
lanosterol synthase	5.4.99.7	Rat	17088293
long-chain-fatty-acid---CoA ligase	6.2.1.3	Rat	11375393
long-chain-fatty-acid---CoA ligase	6.2.1.3	Rat	1769731
glutamate---ammonia ligase	6.3.1.2	Rat	10092169
glutamate---ammonia ligase	6.3.1.2	Rat	10383611
glutamate---ammonia ligase	6.3.1.2	Rat	10440891
glutamate---ammonia ligase	6.3.1.2	Rat	10664131
glutamate---ammonia ligase	6.3.1.2	Rat	11069692
glutamate---ammonia ligase	6.3.1.2	Rat	11413247
glutamate---ammonia ligase	6.3.1.2	Rat	11939529
glutamate---ammonia ligase	6.3.1.2	Rat	12232192
glutamate---ammonia ligase	6.3.1.2	Rat	12517141
glutamate---ammonia ligase	6.3.1.2	Rat	12552916
glutamate---ammonia ligase	6.3.1.2	Rat	14714472
glutamate---ammonia ligase	6.3.1.2	Rat	14723991
glutamate---ammonia ligase	6.3.1.2	Rat	15130478
glutamate---ammonia ligase	6.3.1.2	Rat	15489445
glutamate---ammonia ligase	6.3.1.2	Rat	15581577
glutamate---ammonia ligase	6.3.1.2	Rat	1612427
glutamate---ammonia ligase	6.3.1.2	Rat	16687472
glutamate---ammonia ligase	6.3.1.2	Rat	16829528
glutamate---ammonia ligase	6.3.1.2	Rat	16946267
glutamate---ammonia ligase	6.3.1.2	Rat	2434618
glutamate---ammonia ligase	6.3.1.2	Rat	26663
glutamate---ammonia ligase	6.3.1.2	Rat	6118373
glutamate---ammonia ligase	6.3.1.2	Rat	9624228

glutamate---cysteine ligase	6.3.2.2	Rat	10215022
glutamate---cysteine ligase	6.3.2.2	Rat	10218647
glutamate---cysteine ligase	6.3.2.2	Rat	10349842
glutamate---cysteine ligase	6.3.2.2	Rat	10385608
glutamate---cysteine ligase	6.3.2.2	Rat	10385658
glutamate---cysteine ligase	6.3.2.2	Rat	10399958
glutamate---cysteine ligase	6.3.2.2	Rat	10439045
glutamate---cysteine ligase	6.3.2.2	Rat	10441483
glutamate---cysteine ligase	6.3.2.2	Rat	10486302
glutamate---cysteine ligase	6.3.2.2	Rat	10515588
glutamate---cysteine ligase	6.3.2.2	Rat	10518117
glutamate---cysteine ligase	6.3.2.2	Rat	10544055
glutamate---cysteine ligase	6.3.2.2	Rat	10544272
glutamate---cysteine ligase	6.3.2.2	Rat	10590319
glutamate---cysteine ligase	6.3.2.2	Rat	10593589
glutamate---cysteine ligase	6.3.2.2	Rat	10594104
glutamate---cysteine ligase	6.3.2.2	Rat	10600876
glutamate---cysteine ligase	6.3.2.2	Rat	10623879
glutamate---cysteine ligase	6.3.2.2	Rat	10644053
glutamate---cysteine ligase	6.3.2.2	Rat	10674357
glutamate---cysteine ligase	6.3.2.2	Rat	10677377
glutamate---cysteine ligase	6.3.2.2	Rat	10702364
glutamate---cysteine ligase	6.3.2.2	Rat	10719238
glutamate---cysteine ligase	6.3.2.2	Rat	10733945
glutamate---cysteine ligase	6.3.2.2	Rat	10748080
glutamate---cysteine ligase	6.3.2.2	Rat	10773686
glutamate---cysteine ligase	6.3.2.2	Rat	10777712
glutamate---cysteine ligase	6.3.2.2	Rat	10802223
glutamate---cysteine ligase	6.3.2.2	Rat	10928075
glutamate---cysteine ligase	6.3.2.2	Rat	10960449
glutamate---cysteine ligase	6.3.2.2	Rat	11007940
glutamate---cysteine ligase	6.3.2.2	Rat	11025451
glutamate---cysteine ligase	6.3.2.2	Rat	11028671
glutamate---cysteine ligase	6.3.2.2	Rat	11032771
glutamate---cysteine ligase	6.3.2.2	Rat	11097862
glutamate---cysteine ligase	6.3.2.2	Rat	11133045
glutamate---cysteine ligase	6.3.2.2	Rat	11157875
glutamate---cysteine ligase	6.3.2.2	Rat	11163433
glutamate---cysteine ligase	6.3.2.2	Rat	11181934
glutamate---cysteine ligase	6.3.2.2	Rat	11233143
glutamate---cysteine ligase	6.3.2.2	Rat	11306445
glutamate---cysteine ligase	6.3.2.2	Rat	11339815
glutamate---cysteine ligase	6.3.2.2	Rat	11352989

glutamate---cysteine ligase	6.3.2.2	Rat	11353135
glutamate---cysteine ligase	6.3.2.2	Rat	11500053
glutamate---cysteine ligase	6.3.2.2	Rat	11560771
glutamate---cysteine ligase	6.3.2.2	Rat	11565956
glutamate---cysteine ligase	6.3.2.2	Rat	11687904
glutamate---cysteine ligase	6.3.2.2	Rat	11705692
glutamate---cysteine ligase	6.3.2.2	Rat	11780957
glutamate---cysteine ligase	6.3.2.2	Rat	11781188
glutamate---cysteine ligase	6.3.2.2	Rat	11790356
glutamate---cysteine ligase	6.3.2.2	Rat	11812649
glutamate---cysteine ligase	6.3.2.2	Rat	11820781
glutamate---cysteine ligase	6.3.2.2	Rat	11841806
glutamate---cysteine ligase	6.3.2.2	Rat	11849402
glutamate---cysteine ligase	6.3.2.2	Rat	11876501
glutamate---cysteine ligase	6.3.2.2	Rat	11970852
glutamate---cysteine ligase	6.3.2.2	Rat	12079521
glutamate---cysteine ligase	6.3.2.2	Rat	12111865
glutamate---cysteine ligase	6.3.2.2	Rat	12147223
glutamate---cysteine ligase	6.3.2.2	Rat	12196927
glutamate---cysteine ligase	6.3.2.2	Rat	12200125
glutamate---cysteine ligase	6.3.2.2	Rat	12204877
glutamate---cysteine ligase	6.3.2.2	Rat	12433058
glutamate---cysteine ligase	6.3.2.2	Rat	12448821
glutamate---cysteine ligase	6.3.2.2	Rat	12452384
glutamate---cysteine ligase	6.3.2.2	Rat	12535742
glutamate---cysteine ligase	6.3.2.2	Rat	12594957
glutamate---cysteine ligase	6.3.2.2	Rat	12601050
glutamate---cysteine ligase	6.3.2.2	Rat	12607907
glutamate---cysteine ligase	6.3.2.2	Rat	12628495
glutamate---cysteine ligase	6.3.2.2	Rat	12637989
glutamate---cysteine ligase	6.3.2.2	Rat	12814619
glutamate---cysteine ligase	6.3.2.2	Rat	12882455
glutamate---cysteine ligase	6.3.2.2	Rat	12913252
glutamate---cysteine ligase	6.3.2.2	Rat	1350904
glutamate---cysteine ligase	6.3.2.2	Rat	1351382
glutamate---cysteine ligase	6.3.2.2	Rat	1353765
glutamate---cysteine ligase	6.3.2.2	Rat	1355406
glutamate---cysteine ligase	6.3.2.2	Rat	1362226
glutamate---cysteine ligase	6.3.2.2	Rat	13679058
glutamate---cysteine ligase	6.3.2.2	Rat	14500406
glutamate---cysteine ligase	6.3.2.2	Rat	14514673
glutamate---cysteine ligase	6.3.2.2	Rat	14744626
glutamate---cysteine ligase	6.3.2.2	Rat	14962359

glutamate---cysteine ligase	6.3.2.2	Rat	15020643
glutamate---cysteine ligase	6.3.2.2	Rat	15050748
glutamate---cysteine ligase	6.3.2.2	Rat	15257546
glutamate---cysteine ligase	6.3.2.2	Rat	15314090
glutamate---cysteine ligase	6.3.2.2	Rat	15374419
glutamate---cysteine ligase	6.3.2.2	Rat	15451066
glutamate---cysteine ligase	6.3.2.2	Rat	15477603
glutamate---cysteine ligase	6.3.2.2	Rat	15509664
glutamate---cysteine ligase	6.3.2.2	Rat	15878398
glutamate---cysteine ligase	6.3.2.2	Rat	15946948
glutamate---cysteine ligase	6.3.2.2	Rat	16032782
glutamate---cysteine ligase	6.3.2.2	Rat	16081425
glutamate---cysteine ligase	6.3.2.2	Rat	16162662
glutamate---cysteine ligase	6.3.2.2	Rat	16183645
glutamate---cysteine ligase	6.3.2.2	Rat	16549430
glutamate---cysteine ligase	6.3.2.2	Rat	16566126
glutamate---cysteine ligase	6.3.2.2	Rat	1678010
glutamate---cysteine ligase	6.3.2.2	Rat	16781460
glutamate---cysteine ligase	6.3.2.2	Rat	16949561
glutamate---cysteine ligase	6.3.2.2	Rat	16960387
glutamate---cysteine ligase	6.3.2.2	Rat	17144898
glutamate---cysteine ligase	6.3.2.2	Rat	1784629
glutamate---cysteine ligase	6.3.2.2	Rat	1970723
glutamate---cysteine ligase	6.3.2.2	Rat	1997009
glutamate---cysteine ligase	6.3.2.2	Rat	2572174
glutamate---cysteine ligase	6.3.2.2	Rat	2574245
glutamate---cysteine ligase	6.3.2.2	Rat	2879531
glutamate---cysteine ligase	6.3.2.2	Rat	2897858
glutamate---cysteine ligase	6.3.2.2	Rat	2901982
glutamate---cysteine ligase	6.3.2.2	Rat	3621155
glutamate---cysteine ligase	6.3.2.2	Rat	6897891
glutamate---cysteine ligase	6.3.2.2	Rat	7351635
glutamate---cysteine ligase	6.3.2.2	Rat	7503776
glutamate---cysteine ligase	6.3.2.2	Rat	7568279
glutamate---cysteine ligase	6.3.2.2	Rat	7570642
glutamate---cysteine ligase	6.3.2.2	Rat	7585502
glutamate---cysteine ligase	6.3.2.2	Rat	7622006
glutamate---cysteine ligase	6.3.2.2	Rat	7651354
glutamate---cysteine ligase	6.3.2.2	Rat	7768207
glutamate---cysteine ligase	6.3.2.2	Rat	7901332
glutamate---cysteine ligase	6.3.2.2	Rat	7908245
glutamate---cysteine ligase	6.3.2.2	Rat	7910419
glutamate---cysteine ligase	6.3.2.2	Rat	7929374

glutamate---cysteine ligase	6.3.2.2	Rat	7955076
glutamate---cysteine ligase	6.3.2.2	Rat	7969079
glutamate---cysteine ligase	6.3.2.2	Rat	8001239
glutamate---cysteine ligase	6.3.2.2	Rat	8065332
glutamate---cysteine ligase	6.3.2.2	Rat	8101766
glutamate---cysteine ligase	6.3.2.2	Rat	8106072
glutamate---cysteine ligase	6.3.2.2	Rat	8120650
glutamate---cysteine ligase	6.3.2.2	Rat	8538700
glutamate---cysteine ligase	6.3.2.2	Rat	8582653
glutamate---cysteine ligase	6.3.2.2	Rat	8648118
glutamate---cysteine ligase	6.3.2.2	Rat	8661240
glutamate---cysteine ligase	6.3.2.2	Rat	8751598
glutamate---cysteine ligase	6.3.2.2	Rat	8781554
glutamate---cysteine ligase	6.3.2.2	Rat	8792848
glutamate---cysteine ligase	6.3.2.2	Rat	8806884
glutamate---cysteine ligase	6.3.2.2	Rat	8843715
glutamate---cysteine ligase	6.3.2.2	Rat	8917676
glutamate---cysteine ligase	6.3.2.2	Rat	8930687
glutamate---cysteine ligase	6.3.2.2	Rat	8947504
glutamate---cysteine ligase	6.3.2.2	Rat	8973794
glutamate---cysteine ligase	6.3.2.2	Rat	8981036
glutamate---cysteine ligase	6.3.2.2	Rat	8995480
glutamate---cysteine ligase	6.3.2.2	Rat	9029270
glutamate---cysteine ligase	6.3.2.2	Rat	9054446
glutamate---cysteine ligase	6.3.2.2	Rat	9063478
glutamate---cysteine ligase	6.3.2.2	Rat	9093011
glutamate---cysteine ligase	6.3.2.2	Rat	9119067
glutamate---cysteine ligase	6.3.2.2	Rat	9157984
glutamate---cysteine ligase	6.3.2.2	Rat	9163779
glutamate---cysteine ligase	6.3.2.2	Rat	9185621
glutamate---cysteine ligase	6.3.2.2	Rat	9214623
glutamate---cysteine ligase	6.3.2.2	Rat	9259355
glutamate---cysteine ligase	6.3.2.2	Rat	9268987
glutamate---cysteine ligase	6.3.2.2	Rat	9288403
glutamate---cysteine ligase	6.3.2.2	Rat	9311606
glutamate---cysteine ligase	6.3.2.2	Rat	9374111
glutamate---cysteine ligase	6.3.2.2	Rat	9389600
glutamate---cysteine ligase	6.3.2.2	Rat	9393741
glutamate---cysteine ligase	6.3.2.2	Rat	9425930
glutamate---cysteine ligase	6.3.2.2	Rat	9582278
glutamate---cysteine ligase	6.3.2.2	Rat	9614065
glutamate---cysteine ligase	6.3.2.2	Rat	9626582
glutamate---cysteine ligase	6.3.2.2	Rat	9647756

glutamate---cysteine ligase	6.3.2.2	Rat	9679558
glutamate---cysteine ligase	6.3.2.2	Rat	9703946
glutamate---cysteine ligase	6.3.2.2	Rat	9729439
glutamate---cysteine ligase	6.3.2.2	Rat	9750167
glutamate---cysteine ligase	6.3.2.2	Rat	9756861
glutamate---cysteine ligase	6.3.2.2	Rat	9762423
glutamate---cysteine ligase	6.3.2.2	Rat	9875552
glutamate---cysteine ligase	6.3.2.2	Rat	9895302
glutathione synthase	6.3.2.3	Rat	10964706
glutathione synthase	6.3.2.3	Rat	11708780
glutathione synthase	6.3.2.3	Rat	9880348
phosphoribosylaminoimidazolesuccinocarboxamide synthase	6.3.2.6	Rat	701284
carbamoyl-phosphate synthase (ammonia)	6.3.4.16	Rat	10047492
carbamoyl-phosphate synthase (ammonia)	6.3.4.16	Rat	11872754
carbamoyl-phosphate synthase (ammonia)	6.3.4.16	Rat	189805
carbamoyl-phosphate synthase (ammonia)	6.3.4.16	Rat	7459875
carbamoyl-phosphate synthase (ammonia)	6.3.4.16	Rat	7587391
carbamoyl-phosphate synthase (ammonia)	6.3.4.16	Rat	8752005
CTP synthase	6.3.4.2	Rat	12678497
CTP synthase	6.3.4.2	Rat	16820675
CTP synthase	6.3.4.2	Rat	17463002
CTP synthase	6.3.4.2	Rat	17681942
CTP synthase	6.3.4.2	Rat	2787169
argininosuccinate synthase	6.3.4.5	Rat	10709858
argininosuccinate synthase	6.3.4.5	Rat	1122920
argininosuccinate synthase	6.3.4.5	Rat	11556547
argininosuccinate synthase	6.3.4.5	Rat	12618329
argininosuccinate synthase	6.3.4.5	Rat	12672181
argininosuccinate synthase	6.3.4.5	Rat	1372742
argininosuccinate synthase	6.3.4.5	Rat	14571701
argininosuccinate synthase	6.3.4.5	Rat	15588718
argininosuccinate synthase	6.3.4.5	Rat	16085056
argininosuccinate synthase	6.3.4.5	Rat	16787144
argininosuccinate synthase	6.3.4.5	Rat	558104
argininosuccinate synthase	6.3.4.5	Rat	845694
argininosuccinate synthase	6.3.4.5	Rat	8616812
argininosuccinate synthase	6.3.4.5	Rat	8798625
argininosuccinate synthase	6.3.4.5	Rat	9096605
argininosuccinate synthase	6.3.4.5	Rat	9176259
argininosuccinate synthase	6.3.4.5	Rat	9211993
argininosuccinate synthase	6.3.4.5	Rat	9252090
pyruvate carboxylase	6.4.1.1	Rat	10323732

pyruvate carboxylase	6.4.1.1	Rat	16325442
pyruvate carboxylase	6.4.1.1	Rat	3182810
pyruvate carboxylase	6.4.1.1	Rat	6721853
acetyl-CoA carboxylase	6.4.1.2	Rat	10098661
acetyl-CoA carboxylase	6.4.1.2	Rat	10215591
acetyl-CoA carboxylase	6.4.1.2	Rat	10757783
acetyl-CoA carboxylase	6.4.1.2	Rat	10945143
acetyl-CoA carboxylase	6.4.1.2	Rat	11078738
acetyl-CoA carboxylase	6.4.1.2	Rat	11205884
acetyl-CoA carboxylase	6.4.1.2	Rat	11504381
acetyl-CoA carboxylase	6.4.1.2	Rat	11515553
acetyl-CoA carboxylase	6.4.1.2	Rat	11546765
acetyl-CoA carboxylase	6.4.1.2	Rat	12440972
acetyl-CoA carboxylase	6.4.1.2	Rat	14627750
acetyl-CoA carboxylase	6.4.1.2	Rat	15333468
acetyl-CoA carboxylase	6.4.1.2	Rat	15607423
acetyl-CoA carboxylase	6.4.1.2	Rat	15607568
acetyl-CoA carboxylase	6.4.1.2	Rat	16222055
acetyl-CoA carboxylase	6.4.1.2	Rat	16707454
acetyl-CoA carboxylase	6.4.1.2	Rat	16968879
acetyl-CoA carboxylase	6.4.1.2	Rat	17266990
acetyl-CoA carboxylase	6.4.1.2	Rat	17653193
acetyl-CoA carboxylase	6.4.1.2	Rat	1978829
acetyl-CoA carboxylase	6.4.1.2	Rat	2570725
acetyl-CoA carboxylase	6.4.1.2	Rat	2861941
acetyl-CoA carboxylase	6.4.1.2	Rat	2894828
acetyl-CoA carboxylase	6.4.1.2	Rat	7436865
acetyl-CoA carboxylase	6.4.1.2	Rat	7903266
acetyl-CoA carboxylase	6.4.1.2	Rat	7915138
acetyl-CoA carboxylase	6.4.1.2	Rat	8814137
acetyl-CoA carboxylase	6.4.1.2	Rat	9028876
acetyl-CoA carboxylase	6.4.1.2	Rat	9082912
acetyl-CoA carboxylase	6.4.1.2	Rat	9109840
alcohol dehydrogenase	1.1.1.1	Yeast	1096557
alcohol dehydrogenase	1.1.1.1	Yeast	11303599
alcohol dehydrogenase	1.1.1.1	Yeast	1148277
alcohol dehydrogenase	1.1.1.1	Yeast	12147722
alcohol dehydrogenase	1.1.1.1	Yeast	12489977
alcohol dehydrogenase	1.1.1.1	Yeast	16662798
alcohol dehydrogenase	1.1.1.1	Yeast	167557
alcohol dehydrogenase	1.1.1.1	Yeast	2932116
alcohol dehydrogenase	1.1.1.1	Yeast	3067025
alcohol dehydrogenase	1.1.1.1	Yeast	3893194

alcohol dehydrogenase	1.1.1.1	Yeast	4038269
alcohol dehydrogenase	1.1.1.1	Yeast	6340613
alcohol dehydrogenase	1.1.1.1	Yeast	6341787
alcohol dehydrogenase	1.1.1.1	Yeast	6356161
alcohol dehydrogenase	1.1.1.1	Yeast	6363888
alcohol dehydrogenase	1.1.1.1	Yeast	6370140
alcohol dehydrogenase	1.1.1.1	Yeast	8277258
alcohol dehydrogenase	1.1.1.1	Yeast	8692838
alcohol dehydrogenase	1.1.1.1	Yeast	8905240
alcohol dehydrogenase	1.1.1.1	Yeast	9526508
IMP dehydrogenase	1.1.1.205	Yeast	10194364
IMP dehydrogenase	1.1.1.205	Yeast	10390601
IMP dehydrogenase	1.1.1.205	Yeast	10390603
IMP dehydrogenase	1.1.1.205	Yeast	10391669
IMP dehydrogenase	1.1.1.205	Yeast	10417742
IMP dehydrogenase	1.1.1.205	Yeast	10930578
IMP dehydrogenase	1.1.1.205	Yeast	10953035
IMP dehydrogenase	1.1.1.205	Yeast	10953295
IMP dehydrogenase	1.1.1.205	Yeast	10973868
IMP dehydrogenase	1.1.1.205	Yeast	11003640
IMP dehydrogenase	1.1.1.205	Yeast	1106431
IMP dehydrogenase	1.1.1.205	Yeast	11076502
IMP dehydrogenase	1.1.1.205	Yeast	11145582
IMP dehydrogenase	1.1.1.205	Yeast	11223253
IMP dehydrogenase	1.1.1.205	Yeast	11233304
IMP dehydrogenase	1.1.1.205	Yeast	11288107
IMP dehydrogenase	1.1.1.205	Yeast	11454943
IMP dehydrogenase	1.1.1.205	Yeast	11522119
IMP dehydrogenase	1.1.1.205	Yeast	11566360
IMP dehydrogenase	1.1.1.205	Yeast	11712223
IMP dehydrogenase	1.1.1.205	Yeast	11724288
IMP dehydrogenase	1.1.1.205	Yeast	11875050
IMP dehydrogenase	1.1.1.205	Yeast	11966437
IMP dehydrogenase	1.1.1.205	Yeast	11966440
IMP dehydrogenase	1.1.1.205	Yeast	11966441
IMP dehydrogenase	1.1.1.205	Yeast	12014950
IMP dehydrogenase	1.1.1.205	Yeast	12183689
IMP dehydrogenase	1.1.1.205	Yeast	12213477
IMP dehydrogenase	1.1.1.205	Yeast	12235158
IMP dehydrogenase	1.1.1.205	Yeast	12403633
IMP dehydrogenase	1.1.1.205	Yeast	12559919
IMP dehydrogenase	1.1.1.205	Yeast	12609835
IMP dehydrogenase	1.1.1.205	Yeast	12746440

IMP dehydrogenase	1.1.1.205	Yeast	12773970
IMP dehydrogenase	1.1.1.205	Yeast	12944494
IMP dehydrogenase	1.1.1.205	Yeast	1353938
IMP dehydrogenase	1.1.1.205	Yeast	1356621
IMP dehydrogenase	1.1.1.205	Yeast	14703952
IMP dehydrogenase	1.1.1.205	Yeast	14757177
IMP dehydrogenase	1.1.1.205	Yeast	14766016
IMP dehydrogenase	1.1.1.205	Yeast	14973196
IMP dehydrogenase	1.1.1.205	Yeast	14981049
IMP dehydrogenase	1.1.1.205	Yeast	15043157
IMP dehydrogenase	1.1.1.205	Yeast	15083807
IMP dehydrogenase	1.1.1.205	Yeast	15292516
IMP dehydrogenase	1.1.1.205	Yeast	15355510
IMP dehydrogenase	1.1.1.205	Yeast	15829418
IMP dehydrogenase	1.1.1.205	Yeast	15869715
IMP dehydrogenase	1.1.1.205	Yeast	15882147
IMP dehydrogenase	1.1.1.205	Yeast	15940263
IMP dehydrogenase	1.1.1.205	Yeast	16128570
IMP dehydrogenase	1.1.1.205	Yeast	16243838
IMP dehydrogenase	1.1.1.205	Yeast	16248022
IMP dehydrogenase	1.1.1.205	Yeast	16333815
IMP dehydrogenase	1.1.1.205	Yeast	16647299
IMP dehydrogenase	1.1.1.205	Yeast	16725387
IMP dehydrogenase	1.1.1.205	Yeast	1677309
IMP dehydrogenase	1.1.1.205	Yeast	16936083
IMP dehydrogenase	1.1.1.205	Yeast	17100698
IMP dehydrogenase	1.1.1.205	Yeast	1717828
IMP dehydrogenase	1.1.1.205	Yeast	1723703
IMP dehydrogenase	1.1.1.205	Yeast	1975748
IMP dehydrogenase	1.1.1.205	Yeast	197916
IMP dehydrogenase	1.1.1.205	Yeast	2902093
IMP dehydrogenase	1.1.1.205	Yeast	3314714
IMP dehydrogenase	1.1.1.205	Yeast	4868171
IMP dehydrogenase	1.1.1.205	Yeast	6120758
IMP dehydrogenase	1.1.1.205	Yeast	7476879
IMP dehydrogenase	1.1.1.205	Yeast	7476895
IMP dehydrogenase	1.1.1.205	Yeast	7520100
IMP dehydrogenase	1.1.1.205	Yeast	7874783
IMP dehydrogenase	1.1.1.205	Yeast	7903533
IMP dehydrogenase	1.1.1.205	Yeast	7914720
IMP dehydrogenase	1.1.1.205	Yeast	8103312
IMP dehydrogenase	1.1.1.205	Yeast	8555204
IMP dehydrogenase	1.1.1.205	Yeast	8560580

IMP dehydrogenase	1.1.1.205	Yeast	8830834
IMP dehydrogenase	1.1.1.205	Yeast	8869741
IMP dehydrogenase	1.1.1.205	Yeast	8910338
IMP dehydrogenase	1.1.1.205	Yeast	9042309
IMP dehydrogenase	1.1.1.205	Yeast	9108641
IMP dehydrogenase	1.1.1.205	Yeast	9268334
IMP dehydrogenase	1.1.1.205	Yeast	9278455
IMP dehydrogenase	1.1.1.205	Yeast	9339960
IMP dehydrogenase	1.1.1.205	Yeast	9399601
IMP dehydrogenase	1.1.1.205	Yeast	9413163
IMP dehydrogenase	1.1.1.205	Yeast	9436988
IMP dehydrogenase	1.1.1.205	Yeast	9752721
IMP dehydrogenase	1.1.1.205	Yeast	9766533
IMP dehydrogenase	1.1.1.205	Yeast	9881055
aldehyde reductase	1.1.1.21	Yeast	10424772
aldehyde reductase	1.1.1.21	Yeast	10656235
aldehyde reductase	1.1.1.21	Yeast	11095596
aldehyde reductase	1.1.1.21	Yeast	11370705
aldehyde reductase	1.1.1.21	Yeast	11422753
aldehyde reductase	1.1.1.21	Yeast	11440832
aldehyde reductase	1.1.1.21	Yeast	11798960
aldehyde reductase	1.1.1.21	Yeast	12135102
aldehyde reductase	1.1.1.21	Yeast	12363257
aldehyde reductase	1.1.1.21	Yeast	12394272
aldehyde reductase	1.1.1.21	Yeast	12871133
aldehyde reductase	1.1.1.21	Yeast	12871136
aldehyde reductase	1.1.1.21	Yeast	12881532
aldehyde reductase	1.1.1.21	Yeast	1393828
aldehyde reductase	1.1.1.21	Yeast	14577653
aldehyde reductase	1.1.1.21	Yeast	1499867
aldehyde reductase	1.1.1.21	Yeast	15210146
aldehyde reductase	1.1.1.21	Yeast	15569136
aldehyde reductase	1.1.1.21	Yeast	15584919
aldehyde reductase	1.1.1.21	Yeast	15734861
aldehyde reductase	1.1.1.21	Yeast	15736047
aldehyde reductase	1.1.1.21	Yeast	16026266
aldehyde reductase	1.1.1.21	Yeast	16037296
aldehyde reductase	1.1.1.21	Yeast	16048249
aldehyde reductase	1.1.1.21	Yeast	16114079
aldehyde reductase	1.1.1.21	Yeast	16452468
aldehyde reductase	1.1.1.21	Yeast	16806328
aldehyde reductase	1.1.1.21	Yeast	16870454
aldehyde reductase	1.1.1.21	Yeast	16900332

aldehyde reductase	1.1.1.21	Yeast	16911628
aldehyde reductase	1.1.1.21	Yeast	2120282
aldehyde reductase	1.1.1.21	Yeast	3025043
aldehyde reductase	1.1.1.21	Yeast	7641310
aldehyde reductase	1.1.1.21	Yeast	8457142
aldehyde reductase	1.1.1.21	Yeast	9215310
aldehyde reductase	1.1.1.21	Yeast	9454604
aldehyde reductase	1.1.1.21	Yeast	9481088
aldehyde reductase	1.1.1.21	Yeast	9709964
hydroxymethylglutaryl-CoA reductase (NADPH)	1.1.1.34	Yeast	10084306
hydroxymethylglutaryl-CoA reductase (NADPH)	1.1.1.34	Yeast	10204089
hydroxymethylglutaryl-CoA reductase (NADPH)	1.1.1.34	Yeast	10377386
hydroxymethylglutaryl-CoA reductase (NADPH)	1.1.1.34	Yeast	10892724
hydroxymethylglutaryl-CoA reductase (NADPH)	1.1.1.34	Yeast	10964918
hydroxymethylglutaryl-CoA reductase (NADPH)	1.1.1.34	Yeast	11043510
hydroxymethylglutaryl-CoA reductase (NADPH)	1.1.1.34	Yeast	11516100
hydroxymethylglutaryl-CoA reductase (NADPH)	1.1.1.34	Yeast	11881568
hydroxymethylglutaryl-CoA reductase (NADPH)	1.1.1.34	Yeast	12405293
hydroxymethylglutaryl-CoA reductase (NADPH)	1.1.1.34	Yeast	12467639
hydroxymethylglutaryl-CoA reductase (NADPH)	1.1.1.34	Yeast	12736772
hydroxymethylglutaryl-CoA reductase (NADPH)	1.1.1.34	Yeast	14523992
hydroxymethylglutaryl-CoA reductase (NADPH)	1.1.1.34	Yeast	14720509
hydroxymethylglutaryl-CoA reductase (NADPH)	1.1.1.34	Yeast	15034683
hydroxymethylglutaryl-CoA reductase (NADPH)	1.1.1.34	Yeast	15248477
hydroxymethylglutaryl-CoA reductase (NADPH)	1.1.1.34	Yeast	15605175
hydroxymethylglutaryl-CoA reductase (NADPH)	1.1.1.34	Yeast	16101500

hydroxymethylglutaryl-CoA reductase (NADPH)	1.1.1.34	Yeast	16168377
hydroxymethylglutaryl-CoA reductase (NADPH)	1.1.1.34	Yeast	1629633
hydroxymethylglutaryl-CoA reductase (NADPH)	1.1.1.34	Yeast	1652430
hydroxymethylglutaryl-CoA reductase (NADPH)	1.1.1.34	Yeast	16611135
hydroxymethylglutaryl-CoA reductase (NADPH)	1.1.1.34	Yeast	187533
hydroxymethylglutaryl-CoA reductase (NADPH)	1.1.1.34	Yeast	1954650
hydroxymethylglutaryl-CoA reductase (NADPH)	1.1.1.34	Yeast	216867
hydroxymethylglutaryl-CoA reductase (NADPH)	1.1.1.34	Yeast	2719596
hydroxymethylglutaryl-CoA reductase (NADPH)	1.1.1.34	Yeast	278983
hydroxymethylglutaryl-CoA reductase (NADPH)	1.1.1.34	Yeast	2921640
hydroxymethylglutaryl-CoA reductase (NADPH)	1.1.1.34	Yeast	2995161
hydroxymethylglutaryl-CoA reductase (NADPH)	1.1.1.34	Yeast	3055919
hydroxymethylglutaryl-CoA reductase (NADPH)	1.1.1.34	Yeast	3131638
hydroxymethylglutaryl-CoA reductase (NADPH)	1.1.1.34	Yeast	3308873
hydroxymethylglutaryl-CoA reductase (NADPH)	1.1.1.34	Yeast	3314447
hydroxymethylglutaryl-CoA reductase (NADPH)	1.1.1.34	Yeast	3689494
hydroxymethylglutaryl-CoA reductase (NADPH)	1.1.1.34	Yeast	3968683
hydroxymethylglutaryl-CoA reductase (NADPH)	1.1.1.34	Yeast	4075700
hydroxymethylglutaryl-CoA reductase (NADPH)	1.1.1.34	Yeast	6088070
hydroxymethylglutaryl-CoA reductase (NADPH)	1.1.1.34	Yeast	6256737
hydroxymethylglutaryl-CoA reductase (NADPH)	1.1.1.34	Yeast	6274615

hydroxymethylglutaryl-CoA reductase (NADPH)	1.1.1.34	Yeast	6286363
hydroxymethylglutaryl-CoA reductase (NADPH)	1.1.1.34	Yeast	6347025
hydroxymethylglutaryl-CoA reductase (NADPH)	1.1.1.34	Yeast	6396116
hydroxymethylglutaryl-CoA reductase (NADPH)	1.1.1.34	Yeast	6594693
hydroxymethylglutaryl-CoA reductase (NADPH)	1.1.1.34	Yeast	666819
hydroxymethylglutaryl-CoA reductase (NADPH)	1.1.1.34	Yeast	6685129
hydroxymethylglutaryl-CoA reductase (NADPH)	1.1.1.34	Yeast	6698994
hydroxymethylglutaryl-CoA reductase (NADPH)	1.1.1.34	Yeast	8054400
hydroxymethylglutaryl-CoA reductase (NADPH)	1.1.1.34	Yeast	8182149
hydroxymethylglutaryl-CoA reductase (NADPH)	1.1.1.34	Yeast	8393338
hydroxymethylglutaryl-CoA reductase (NADPH)	1.1.1.34	Yeast	8504036
hydroxymethylglutaryl-CoA reductase (NADPH)	1.1.1.34	Yeast	8772195
hydroxymethylglutaryl-CoA reductase (NADPH)	1.1.1.34	Yeast	9151797
hydroxymethylglutaryl-CoA reductase (NADPH)	1.1.1.34	Yeast	9185766
hydroxymethylglutaryl-CoA reductase (NADPH)	1.1.1.34	Yeast	9372476
hydroxymethylglutaryl-CoA reductase (NADPH)	1.1.1.34	Yeast	9558731
hydroxymethylglutaryl-CoA reductase (NADPH)	1.1.1.34	Yeast	9802623
malate dehydrogenase	1.1.1.37	Yeast	11583380
malate dehydrogenase	1.1.1.37	Yeast	11767008
malate dehydrogenase	1.1.1.37	Yeast	11855723
malate dehydrogenase	1.1.1.37	Yeast	131232
malate dehydrogenase	1.1.1.37	Yeast	16028114
malate dehydrogenase	1.1.1.37	Yeast	16212411
malate dehydrogenase	1.1.1.37	Yeast	16661455
malate dehydrogenase	1.1.1.37	Yeast	3995045
malate dehydrogenase	1.1.1.37	Yeast	4053567

malate dehydrogenase	1.1.1.37	Yeast	7138874
malate dehydrogenase	1.1.1.37	Yeast	8624506
malate dehydrogenase	1.1.1.37	Yeast	9348107
isocitrate dehydrogenase (NAD+)	1.1.1.41	Yeast	10461937
isocitrate dehydrogenase (NAD+)	1.1.1.41	Yeast	12033940
isocitrate dehydrogenase (NAD+)	1.1.1.41	Yeast	12619682
isocitrate dehydrogenase (NAD+)	1.1.1.41	Yeast	14555658
isocitrate dehydrogenase (NAD+)	1.1.1.41	Yeast	15173171
isocitrate dehydrogenase (NAD+)	1.1.1.41	Yeast	15314217
isocitrate dehydrogenase (NAD+)	1.1.1.41	Yeast	16415587
isocitrate dehydrogenase (NAD+)	1.1.1.41	Yeast	6389540
isocitrate dehydrogenase (NAD+)	1.1.1.41	Yeast	7710326
isocitrate dehydrogenase (NAD+)	1.1.1.41	Yeast	8626605
isocitrate dehydrogenase (NAD+)	1.1.1.41	Yeast	9733544
isocitrate dehydrogenase (NAD+)	1.1.1.41	Yeast	9881153
glucose-6-phosphate dehydrogenase	1.1.1.49	Yeast	10098886
glucose-6-phosphate dehydrogenase	1.1.1.49	Yeast	10099785
glucose-6-phosphate dehydrogenase	1.1.1.49	Yeast	10329961
glucose-6-phosphate dehydrogenase	1.1.1.49	Yeast	10825753
glucose-6-phosphate dehydrogenase	1.1.1.49	Yeast	10998184
glucose-6-phosphate dehydrogenase	1.1.1.49	Yeast	11023706
glucose-6-phosphate dehydrogenase	1.1.1.49	Yeast	11245448
glucose-6-phosphate dehydrogenase	1.1.1.49	Yeast	11463792
glucose-6-phosphate dehydrogenase	1.1.1.49	Yeast	11520909
glucose-6-phosphate dehydrogenase	1.1.1.49	Yeast	12027950
glucose-6-phosphate dehydrogenase	1.1.1.49	Yeast	12204336
glucose-6-phosphate dehydrogenase	1.1.1.49	Yeast	12393032
glucose-6-phosphate dehydrogenase	1.1.1.49	Yeast	12414804
glucose-6-phosphate dehydrogenase	1.1.1.49	Yeast	12453665
glucose-6-phosphate dehydrogenase	1.1.1.49	Yeast	12472120
glucose-6-phosphate dehydrogenase	1.1.1.49	Yeast	12502759
glucose-6-phosphate dehydrogenase	1.1.1.49	Yeast	131232
glucose-6-phosphate dehydrogenase	1.1.1.49	Yeast	1384463
glucose-6-phosphate dehydrogenase	1.1.1.49	Yeast	1417703
glucose-6-phosphate dehydrogenase	1.1.1.49	Yeast	147929
glucose-6-phosphate dehydrogenase	1.1.1.49	Yeast	15331344
glucose-6-phosphate dehydrogenase	1.1.1.49	Yeast	15345489
glucose-6-phosphate dehydrogenase	1.1.1.49	Yeast	15527069
glucose-6-phosphate dehydrogenase	1.1.1.49	Yeast	15550513
glucose-6-phosphate dehydrogenase	1.1.1.49	Yeast	15634201
glucose-6-phosphate dehydrogenase	1.1.1.49	Yeast	15739803
glucose-6-phosphate dehydrogenase	1.1.1.49	Yeast	15760711
glucose-6-phosphate dehydrogenase	1.1.1.49	Yeast	15858258

glucose-6-phosphate dehydrogenase	1.1.1.49	Yeast	15975496
glucose-6-phosphate dehydrogenase	1.1.1.49	Yeast	16039947
glucose-6-phosphate dehydrogenase	1.1.1.49	Yeast	16439706
glucose-6-phosphate dehydrogenase	1.1.1.49	Yeast	16849632
glucose-6-phosphate dehydrogenase	1.1.1.49	Yeast	17157446
glucose-6-phosphate dehydrogenase	1.1.1.49	Yeast	1830744
glucose-6-phosphate dehydrogenase	1.1.1.49	Yeast	1922658
glucose-6-phosphate dehydrogenase	1.1.1.49	Yeast	1978808
glucose-6-phosphate dehydrogenase	1.1.1.49	Yeast	2208076
glucose-6-phosphate dehydrogenase	1.1.1.49	Yeast	2296762
glucose-6-phosphate dehydrogenase	1.1.1.49	Yeast	2767006
glucose-6-phosphate dehydrogenase	1.1.1.49	Yeast	2808772
glucose-6-phosphate dehydrogenase	1.1.1.49	Yeast	2846196
glucose-6-phosphate dehydrogenase	1.1.1.49	Yeast	2984461
glucose-6-phosphate dehydrogenase	1.1.1.49	Yeast	3116361
glucose-6-phosphate dehydrogenase	1.1.1.49	Yeast	3161339
glucose-6-phosphate dehydrogenase	1.1.1.49	Yeast	3316204
glucose-6-phosphate dehydrogenase	1.1.1.49	Yeast	3337882
glucose-6-phosphate dehydrogenase	1.1.1.49	Yeast	3365274
glucose-6-phosphate dehydrogenase	1.1.1.49	Yeast	3532684
glucose-6-phosphate dehydrogenase	1.1.1.49	Yeast	3621197
glucose-6-phosphate dehydrogenase	1.1.1.49	Yeast	3717951
glucose-6-phosphate dehydrogenase	1.1.1.49	Yeast	3765490
glucose-6-phosphate dehydrogenase	1.1.1.49	Yeast	3949801
glucose-6-phosphate dehydrogenase	1.1.1.49	Yeast	4288679
glucose-6-phosphate dehydrogenase	1.1.1.49	Yeast	4382249
glucose-6-phosphate dehydrogenase	1.1.1.49	Yeast	4400642
glucose-6-phosphate dehydrogenase	1.1.1.49	Yeast	4941552
glucose-6-phosphate dehydrogenase	1.1.1.49	Yeast	6295653
glucose-6-phosphate dehydrogenase	1.1.1.49	Yeast	6341787
glucose-6-phosphate dehydrogenase	1.1.1.49	Yeast	6363888
glucose-6-phosphate dehydrogenase	1.1.1.49	Yeast	6420889
glucose-6-phosphate dehydrogenase	1.1.1.49	Yeast	645360
glucose-6-phosphate dehydrogenase	1.1.1.49	Yeast	6591771
glucose-6-phosphate dehydrogenase	1.1.1.49	Yeast	6696439
glucose-6-phosphate dehydrogenase	1.1.1.49	Yeast	7126822
glucose-6-phosphate dehydrogenase	1.1.1.49	Yeast	7578910
glucose-6-phosphate dehydrogenase	1.1.1.49	Yeast	7681896
glucose-6-phosphate dehydrogenase	1.1.1.49	Yeast	7768207
glucose-6-phosphate dehydrogenase	1.1.1.49	Yeast	7930940
glucose-6-phosphate dehydrogenase	1.1.1.49	Yeast	8316633
glucose-6-phosphate dehydrogenase	1.1.1.49	Yeast	864
glucose-6-phosphate dehydrogenase	1.1.1.49	Yeast	8760336

glucose-6-phosphate dehydrogenase	1.1.1.49	Yeast	8797095
glucose-6-phosphate dehydrogenase	1.1.1.49	Yeast	8857518
glucose-6-phosphate dehydrogenase	1.1.1.49	Yeast	8910528
glucose-6-phosphate dehydrogenase	1.1.1.49	Yeast	8954569
glucose-6-phosphate dehydrogenase	1.1.1.49	Yeast	9042391
glucose-6-phosphate dehydrogenase	1.1.1.49	Yeast	9553122
glucose-6-phosphate dehydrogenase	1.1.1.49	Yeast	9581796
glucose-6-phosphate dehydrogenase	1.1.1.49	Yeast	9915806
ubiquinol---cytochrome-c reductase	1.10.2.2	Yeast	16005845
ubiquinol---cytochrome-c reductase	1.10.2.2	Yeast	17223530
indoleamine 2,3-dioxygenase	1.13.11.52	Yeast	10721098
indoleamine 2,3-dioxygenase	1.13.11.52	Yeast	10731095
indoleamine 2,3-dioxygenase	1.13.11.52	Yeast	10833386
indoleamine 2,3-dioxygenase	1.13.11.52	Yeast	10926204
indoleamine 2,3-dioxygenase	1.13.11.52	Yeast	10939283
indoleamine 2,3-dioxygenase	1.13.11.52	Yeast	10939284
indoleamine 2,3-dioxygenase	1.13.11.52	Yeast	10957719
indoleamine 2,3-dioxygenase	1.13.11.52	Yeast	11180976
indoleamine 2,3-dioxygenase	1.13.11.52	Yeast	11230514
indoleamine 2,3-dioxygenase	1.13.11.52	Yeast	11440641
indoleamine 2,3-dioxygenase	1.13.11.52	Yeast	11477543
indoleamine 2,3-dioxygenase	1.13.11.52	Yeast	11507170
indoleamine 2,3-dioxygenase	1.13.11.52	Yeast	12414962
indoleamine 2,3-dioxygenase	1.13.11.52	Yeast	12832720
indoleamine 2,3-dioxygenase	1.13.11.52	Yeast	12848846
indoleamine 2,3-dioxygenase	1.13.11.52	Yeast	15206741
indoleamine 2,3-dioxygenase	1.13.11.52	Yeast	15254594
indoleamine 2,3-dioxygenase	1.13.11.52	Yeast	15358362
indoleamine 2,3-dioxygenase	1.13.11.52	Yeast	15542091
indoleamine 2,3-dioxygenase	1.13.11.52	Yeast	15853924
indoleamine 2,3-dioxygenase	1.13.11.52	Yeast	15961516
indoleamine 2,3-dioxygenase	1.13.11.52	Yeast	16075385
indoleamine 2,3-dioxygenase	1.13.11.52	Yeast	16083346
indoleamine 2,3-dioxygenase	1.13.11.52	Yeast	16176799
indoleamine 2,3-dioxygenase	1.13.11.52	Yeast	16624246
indoleamine 2,3-dioxygenase	1.13.11.52	Yeast	17055065
indoleamine 2,3-dioxygenase	1.13.11.52	Yeast	8423409
indoleamine 2,3-dioxygenase	1.13.11.52	Yeast	8702590
indoleamine 2,3-dioxygenase	1.13.11.52	Yeast	9466588
unspecific monooxygenase	1.14.14.1	Yeast	10462973
unspecific monooxygenase	1.14.14.1	Yeast	10473018
unspecific monooxygenase	1.14.14.1	Yeast	10474272
unspecific monooxygenase	1.14.14.1	Yeast	10690899

unspecific monooxygenase	1.14.14.1	Yeast	10713305
unspecific monooxygenase	1.14.14.1	Yeast	10746939
unspecific monooxygenase	1.14.14.1	Yeast	10947336
unspecific monooxygenase	1.14.14.1	Yeast	11116206
unspecific monooxygenase	1.14.14.1	Yeast	11259506
unspecific monooxygenase	1.14.14.1	Yeast	11312650
unspecific monooxygenase	1.14.14.1	Yeast	11403896
unspecific monooxygenase	1.14.14.1	Yeast	11502834
unspecific monooxygenase	1.14.14.1	Yeast	11922772
unspecific monooxygenase	1.14.14.1	Yeast	11961225
unspecific monooxygenase	1.14.14.1	Yeast	12053085
unspecific monooxygenase	1.14.14.1	Yeast	12439219
unspecific monooxygenase	1.14.14.1	Yeast	12606587
unspecific monooxygenase	1.14.14.1	Yeast	12706301
unspecific monooxygenase	1.14.14.1	Yeast	12715371
unspecific monooxygenase	1.14.14.1	Yeast	12736278
unspecific monooxygenase	1.14.14.1	Yeast	12810639
unspecific monooxygenase	1.14.14.1	Yeast	12845227
unspecific monooxygenase	1.14.14.1	Yeast	12957661
unspecific monooxygenase	1.14.14.1	Yeast	12960093
unspecific monooxygenase	1.14.14.1	Yeast	1339246
unspecific monooxygenase	1.14.14.1	Yeast	14501171
unspecific monooxygenase	1.14.14.1	Yeast	14580722
unspecific monooxygenase	1.14.14.1	Yeast	14623534
unspecific monooxygenase	1.14.14.1	Yeast	14965567
unspecific monooxygenase	1.14.14.1	Yeast	15226009
unspecific monooxygenase	1.14.14.1	Yeast	15255840
unspecific monooxygenase	1.14.14.1	Yeast	15319488
unspecific monooxygenase	1.14.14.1	Yeast	15322103
unspecific monooxygenase	1.14.14.1	Yeast	15582747
unspecific monooxygenase	1.14.14.1	Yeast	15591029
unspecific monooxygenase	1.14.14.1	Yeast	15623590
unspecific monooxygenase	1.14.14.1	Yeast	15876405
unspecific monooxygenase	1.14.14.1	Yeast	15885269
unspecific monooxygenase	1.14.14.1	Yeast	15930283
unspecific monooxygenase	1.14.14.1	Yeast	16006326
unspecific monooxygenase	1.14.14.1	Yeast	16077170
unspecific monooxygenase	1.14.14.1	Yeast	16109788
unspecific monooxygenase	1.14.14.1	Yeast	16109840
unspecific monooxygenase	1.14.14.1	Yeast	16170371
unspecific monooxygenase	1.14.14.1	Yeast	16260617
unspecific monooxygenase	1.14.14.1	Yeast	16285913
unspecific monooxygenase	1.14.14.1	Yeast	16303757

unspecific monooxygenase	1.14.14.1	Yeast	16322267
unspecific monooxygenase	1.14.14.1	Yeast	16426763
unspecific monooxygenase	1.14.14.1	Yeast	16473000
unspecific monooxygenase	1.14.14.1	Yeast	16541462
unspecific monooxygenase	1.14.14.1	Yeast	16611627
unspecific monooxygenase	1.14.14.1	Yeast	16733710
unspecific monooxygenase	1.14.14.1	Yeast	16763069
unspecific monooxygenase	1.14.14.1	Yeast	16877675
unspecific monooxygenase	1.14.14.1	Yeast	1694074
unspecific monooxygenase	1.14.14.1	Yeast	17005180
unspecific monooxygenase	1.14.14.1	Yeast	17079138
unspecific monooxygenase	1.14.14.1	Yeast	17573783
unspecific monooxygenase	1.14.14.1	Yeast	1835644
unspecific monooxygenase	1.14.14.1	Yeast	2149503
unspecific monooxygenase	1.14.14.1	Yeast	3419162
unspecific monooxygenase	1.14.14.1	Yeast	3626552
unspecific monooxygenase	1.14.14.1	Yeast	6233132
unspecific monooxygenase	1.14.14.1	Yeast	6580512
unspecific monooxygenase	1.14.14.1	Yeast	6645505
unspecific monooxygenase	1.14.14.1	Yeast	7083186
unspecific monooxygenase	1.14.14.1	Yeast	7581491
unspecific monooxygenase	1.14.14.1	Yeast	7626472
unspecific monooxygenase	1.14.14.1	Yeast	7694590
unspecific monooxygenase	1.14.14.1	Yeast	7949200
unspecific monooxygenase	1.14.14.1	Yeast	7968362
unspecific monooxygenase	1.14.14.1	Yeast	8017854
unspecific monooxygenase	1.14.14.1	Yeast	8073072
unspecific monooxygenase	1.14.14.1	Yeast	8126145
unspecific monooxygenase	1.14.14.1	Yeast	8698750
unspecific monooxygenase	1.14.14.1	Yeast	8824461
unspecific monooxygenase	1.14.14.1	Yeast	8989259
unspecific monooxygenase	1.14.14.1	Yeast	9027406
unspecific monooxygenase	1.14.14.1	Yeast	9038782
unspecific monooxygenase	1.14.14.1	Yeast	9187104
unspecific monooxygenase	1.14.14.1	Yeast	9365209
unspecific monooxygenase	1.14.14.1	Yeast	9415804
unspecific monooxygenase	1.14.14.1	Yeast	9442349
unspecific monooxygenase	1.14.14.1	Yeast	9456245
unspecific monooxygenase	1.14.14.1	Yeast	9511180
unspecific monooxygenase	1.14.14.1	Yeast	9523724
unspecific monooxygenase	1.14.14.1	Yeast	9541188
unspecific monooxygenase	1.14.14.1	Yeast	9698080
unspecific monooxygenase	1.14.14.1	Yeast	9703907

unspecific monooxygenase	1.14.14.1	Yeast	9788750
unspecific monooxygenase	1.14.14.1	Yeast	9797023
unspecific monooxygenase	1.14.14.1	Yeast	9804911
stearoyl-CoA 9-desaturase	1.14.19.1	Yeast	10484602
stearoyl-CoA 9-desaturase	1.14.19.1	Yeast	10581155
stearoyl-CoA 9-desaturase	1.14.19.1	Yeast	11352637
stearoyl-CoA 9-desaturase	1.14.19.1	Yeast	11396956
stearoyl-CoA 9-desaturase	1.14.19.1	Yeast	11415448
stearoyl-CoA 9-desaturase	1.14.19.1	Yeast	11533264
stearoyl-CoA 9-desaturase	1.14.19.1	Yeast	11677241
stearoyl-CoA 9-desaturase	1.14.19.1	Yeast	12364560
stearoyl-CoA 9-desaturase	1.14.19.1	Yeast	12815040
stearoyl-CoA 9-desaturase	1.14.19.1	Yeast	14610276
stearoyl-CoA 9-desaturase	1.14.19.1	Yeast	14654089
stearoyl-CoA 9-desaturase	1.14.19.1	Yeast	14734557
stearoyl-CoA 9-desaturase	1.14.19.1	Yeast	15096593
stearoyl-CoA 9-desaturase	1.14.19.1	Yeast	15180999
stearoyl-CoA 9-desaturase	1.14.19.1	Yeast	15556291
stearoyl-CoA 9-desaturase	1.14.19.1	Yeast	15726820
stearoyl-CoA 9-desaturase	1.14.19.1	Yeast	15761499
stearoyl-CoA 9-desaturase	1.14.19.1	Yeast	15829484
stearoyl-CoA 9-desaturase	1.14.19.1	Yeast	15836467
stearoyl-CoA 9-desaturase	1.14.19.1	Yeast	16245372
stearoyl-CoA 9-desaturase	1.14.19.1	Yeast	16767221
stearoyl-CoA 9-desaturase	1.14.19.1	Yeast	16804073
stearoyl-CoA 9-desaturase	1.14.19.1	Yeast	9272444
squalene monooxygenase	1.14.99.7	Yeast	10679280
squalene monooxygenase	1.14.99.7	Yeast	10733917
squalene monooxygenase	1.14.99.7	Yeast	11199136
squalene monooxygenase	1.14.99.7	Yeast	11520216
squalene monooxygenase	1.14.99.7	Yeast	12226513
squalene monooxygenase	1.14.99.7	Yeast	16466954
squalene monooxygenase	1.14.99.7	Yeast	6087072
squalene monooxygenase	1.14.99.7	Yeast	6547247
squalene monooxygenase	1.14.99.7	Yeast	8771716
squalene monooxygenase	1.14.99.7	Yeast	9017503
ribonucleoside-diphosphate reductase	1.17.4.1	Yeast	10441745
ribonucleoside-diphosphate reductase	1.17.4.1	Yeast	10593972
ribonucleoside-diphosphate reductase	1.17.4.1	Yeast	10769119
ribonucleoside-diphosphate reductase	1.17.4.1	Yeast	10805162
ribonucleoside-diphosphate reductase	1.17.4.1	Yeast	10953295
ribonucleoside-diphosphate reductase	1.17.4.1	Yeast	10989193
ribonucleoside-diphosphate reductase	1.17.4.1	Yeast	11489836

ribonucleoside-diphosphate reductase	1.17.4.1	Yeast	11551528
ribonucleoside-diphosphate reductase	1.17.4.1	Yeast	11904430
ribonucleoside-diphosphate reductase	1.17.4.1	Yeast	12147300
ribonucleoside-diphosphate reductase	1.17.4.1	Yeast	12359454
ribonucleoside-diphosphate reductase	1.17.4.1	Yeast	12655059
ribonucleoside-diphosphate reductase	1.17.4.1	Yeast	12690517
ribonucleoside-diphosphate reductase	1.17.4.1	Yeast	12732713
ribonucleoside-diphosphate reductase	1.17.4.1	Yeast	12749906
ribonucleoside-diphosphate reductase	1.17.4.1	Yeast	12967138
ribonucleoside-diphosphate reductase	1.17.4.1	Yeast	1299271
ribonucleoside-diphosphate reductase	1.17.4.1	Yeast	1385411
ribonucleoside-diphosphate reductase	1.17.4.1	Yeast	1412696
ribonucleoside-diphosphate reductase	1.17.4.1	Yeast	14963934
ribonucleoside-diphosphate reductase	1.17.4.1	Yeast	14966112
ribonucleoside-diphosphate reductase	1.17.4.1	Yeast	1496919
ribonucleoside-diphosphate reductase	1.17.4.1	Yeast	15094776
ribonucleoside-diphosphate reductase	1.17.4.1	Yeast	15133626
ribonucleoside-diphosphate reductase	1.17.4.1	Yeast	1516817
ribonucleoside-diphosphate reductase	1.17.4.1	Yeast	15300180
ribonucleoside-diphosphate reductase	1.17.4.1	Yeast	15571292
ribonucleoside-diphosphate reductase	1.17.4.1	Yeast	15656518
ribonucleoside-diphosphate reductase	1.17.4.1	Yeast	15673563
ribonucleoside-diphosphate reductase	1.17.4.1	Yeast	15730856
ribonucleoside-diphosphate reductase	1.17.4.1	Yeast	15769467
ribonucleoside-diphosphate reductase	1.17.4.1	Yeast	15803490
ribonucleoside-diphosphate reductase	1.17.4.1	Yeast	15805194
ribonucleoside-diphosphate reductase	1.17.4.1	Yeast	15888728
ribonucleoside-diphosphate reductase	1.17.4.1	Yeast	160558
ribonucleoside-diphosphate reductase	1.17.4.1	Yeast	16399800
ribonucleoside-diphosphate reductase	1.17.4.1	Yeast	1643157
ribonucleoside-diphosphate reductase	1.17.4.1	Yeast	16489218
ribonucleoside-diphosphate reductase	1.17.4.1	Yeast	16530987
ribonucleoside-diphosphate reductase	1.17.4.1	Yeast	16834759
ribonucleoside-diphosphate reductase	1.17.4.1	Yeast	16925573
ribonucleoside-diphosphate reductase	1.17.4.1	Yeast	17065057
ribonucleoside-diphosphate reductase	1.17.4.1	Yeast	1717630
ribonucleoside-diphosphate reductase	1.17.4.1	Yeast	1748682
ribonucleoside-diphosphate reductase	1.17.4.1	Yeast	1793565
ribonucleoside-diphosphate reductase	1.17.4.1	Yeast	2085432
ribonucleoside-diphosphate reductase	1.17.4.1	Yeast	2178608
ribonucleoside-diphosphate reductase	1.17.4.1	Yeast	2199320
ribonucleoside-diphosphate reductase	1.17.4.1	Yeast	2257322
ribonucleoside-diphosphate reductase	1.17.4.1	Yeast	2642388

ribonucleoside-diphosphate reductase	1.17.4.1	Yeast	2673261
ribonucleoside-diphosphate reductase	1.17.4.1	Yeast	2775821
ribonucleoside-diphosphate reductase	1.17.4.1	Yeast	2827767
ribonucleoside-diphosphate reductase	1.17.4.1	Yeast	2832057
ribonucleoside-diphosphate reductase	1.17.4.1	Yeast	3044371
ribonucleoside-diphosphate reductase	1.17.4.1	Yeast	3044582
ribonucleoside-diphosphate reductase	1.17.4.1	Yeast	3061459
ribonucleoside-diphosphate reductase	1.17.4.1	Yeast	3300645
ribonucleoside-diphosphate reductase	1.17.4.1	Yeast	3511848
ribonucleoside-diphosphate reductase	1.17.4.1	Yeast	3536076
ribonucleoside-diphosphate reductase	1.17.4.1	Yeast	3907637
ribonucleoside-diphosphate reductase	1.17.4.1	Yeast	3914643
ribonucleoside-diphosphate reductase	1.17.4.1	Yeast	3915189
ribonucleoside-diphosphate reductase	1.17.4.1	Yeast	3986794
ribonucleoside-diphosphate reductase	1.17.4.1	Yeast	6353195
ribonucleoside-diphosphate reductase	1.17.4.1	Yeast	6375753
ribonucleoside-diphosphate reductase	1.17.4.1	Yeast	6752137
ribonucleoside-diphosphate reductase	1.17.4.1	Yeast	6757589
ribonucleoside-diphosphate reductase	1.17.4.1	Yeast	767333
ribonucleoside-diphosphate reductase	1.17.4.1	Yeast	7727399
ribonucleoside-diphosphate reductase	1.17.4.1	Yeast	7768988
ribonucleoside-diphosphate reductase	1.17.4.1	Yeast	7838172
ribonucleoside-diphosphate reductase	1.17.4.1	Yeast	7881162
ribonucleoside-diphosphate reductase	1.17.4.1	Yeast	7882331
ribonucleoside-diphosphate reductase	1.17.4.1	Yeast	7893463
ribonucleoside-diphosphate reductase	1.17.4.1	Yeast	7937896
ribonucleoside-diphosphate reductase	1.17.4.1	Yeast	7984431
ribonucleoside-diphosphate reductase	1.17.4.1	Yeast	8241321
ribonucleoside-diphosphate reductase	1.17.4.1	Yeast	8265664
ribonucleoside-diphosphate reductase	1.17.4.1	Yeast	8343143
ribonucleoside-diphosphate reductase	1.17.4.1	Yeast	8463252
ribonucleoside-diphosphate reductase	1.17.4.1	Yeast	8521087
ribonucleoside-diphosphate reductase	1.17.4.1	Yeast	8620054
ribonucleoside-diphosphate reductase	1.17.4.1	Yeast	8662944
ribonucleoside-diphosphate reductase	1.17.4.1	Yeast	8674535
ribonucleoside-diphosphate reductase	1.17.4.1	Yeast	8813126
ribonucleoside-diphosphate reductase	1.17.4.1	Yeast	8878781
ribonucleoside-diphosphate reductase	1.17.4.1	Yeast	8920917
ribonucleoside-diphosphate reductase	1.17.4.1	Yeast	8943056
ribonucleoside-diphosphate reductase	1.17.4.1	Yeast	9192674
ribonucleoside-diphosphate reductase	1.17.4.1	Yeast	9315670
ribonucleoside-diphosphate reductase	1.17.4.1	Yeast	9347313
ribonucleoside-diphosphate reductase	1.17.4.1	Yeast	9354452

ribonucleoside-diphosphate reductase	1.17.4.1	Yeast	9371820
ribonucleoside-diphosphate reductase	1.17.4.1	Yeast	9393942
ribonucleoside-diphosphate reductase	1.17.4.1	Yeast	9415718
ribonucleoside-diphosphate reductase	1.17.4.1	Yeast	9439883
ribonucleoside-diphosphate reductase	1.17.4.1	Yeast	9558318
ribonucleoside-diphosphate reductase	1.17.4.1	Yeast	9570515
ribonucleoside-diphosphate reductase	1.17.4.1	Yeast	9598136
ribonucleoside-diphosphate reductase	1.17.4.1	Yeast	9605773
ribonucleoside-diphosphate reductase	1.17.4.1	Yeast	9634002
ribonucleoside-diphosphate reductase	1.17.4.1	Yeast	9696008
ribonucleoside-diphosphate reductase	1.17.4.1	Yeast	9718080
ribonucleoside-diphosphate reductase	1.17.4.1	Yeast	9852067
ribonucleoside-diphosphate reductase	1.17.4.1	Yeast	9990288
glyceraldehyde-3-phosphate dehydrogenase (phosphorylating)	1.2.1.12	Yeast	10966377
glyceraldehyde-3-phosphate dehydrogenase (phosphorylating)	1.2.1.12	Yeast	11018719
glyceraldehyde-3-phosphate dehydrogenase (phosphorylating)	1.2.1.12	Yeast	12123463
glyceraldehyde-3-phosphate dehydrogenase (phosphorylating)	1.2.1.12	Yeast	12634343
glyceraldehyde-3-phosphate dehydrogenase (phosphorylating)	1.2.1.12	Yeast	14502604
glyceraldehyde-3-phosphate dehydrogenase (phosphorylating)	1.2.1.12	Yeast	15299328
glyceraldehyde-3-phosphate dehydrogenase (phosphorylating)	1.2.1.12	Yeast	15631980
glyceraldehyde-3-phosphate dehydrogenase (phosphorylating)	1.2.1.12	Yeast	15680915
glyceraldehyde-3-phosphate dehydrogenase (phosphorylating)	1.2.1.12	Yeast	3530169
glyceraldehyde-3-phosphate dehydrogenase (phosphorylating)	1.2.1.12	Yeast	6095107
glyceraldehyde-3-phosphate dehydrogenase (phosphorylating)	1.2.1.12	Yeast	6440018
glyceraldehyde-3-phosphate dehydrogenase (phosphorylating)	1.2.1.12	Yeast	664114
glyceraldehyde-3-phosphate dehydrogenase (phosphorylating)	1.2.1.12	Yeast	7165719
glyceraldehyde-3-phosphate dehydrogenase (phosphorylating)	1.2.1.12	Yeast	7173737
glyceraldehyde-3-phosphate dehydrogenase (phosphorylating)	1.2.1.12	Yeast	8304415

glyceraldehyde-3-phosphate dehydrogenase (phosphorylating)	1.2.1.12	Yeast	8454610
glyceraldehyde-3-phosphate dehydrogenase (phosphorylating)	1.2.1.12	Yeast	8791005
glyceraldehyde-3-phosphate dehydrogenase (phosphorylating)	1.2.1.12	Yeast	8805794
glyceraldehyde-3-phosphate dehydrogenase (phosphorylating)	1.2.1.12	Yeast	8905295
glyceraldehyde-3-phosphate dehydrogenase (phosphorylating)	1.2.1.12	Yeast	9437188
glyceraldehyde-3-phosphate dehydrogenase (phosphorylating)	1.2.1.12	Yeast	9793073
glyceraldehyde-3-phosphate dehydrogenase (phosphorylating)	1.2.1.12	Yeast	9922941
aldehyde dehydrogenase (NAD+)	1.2.1.3	Yeast	10913633
aldehyde dehydrogenase (NAD+)	1.2.1.3	Yeast	11306027
aldehyde dehydrogenase (NAD+)	1.2.1.3	Yeast	12223435
aldehyde dehydrogenase (NAD+)	1.2.1.3	Yeast	14597338
aldehyde dehydrogenase (NAD+)	1.2.1.3	Yeast	1472111
aldehyde dehydrogenase (NAD+)	1.2.1.3	Yeast	16126235
aldehyde dehydrogenase (NAD+)	1.2.1.3	Yeast	16499490
aldehyde dehydrogenase (NAD+)	1.2.1.3	Yeast	16878979
aldehyde dehydrogenase (NAD+)	1.2.1.3	Yeast	17607160
aldehyde dehydrogenase (NAD+)	1.2.1.3	Yeast	2332351
aldehyde dehydrogenase (NAD+)	1.2.1.3	Yeast	3593277
aldehyde dehydrogenase (NAD+)	1.2.1.3	Yeast	6603890
aldehyde dehydrogenase (NAD+)	1.2.1.3	Yeast	7910607
aldehyde dehydrogenase (NAD+)	1.2.1.3	Yeast	8452208
aldehyde dehydrogenase (NAD+)	1.2.1.3	Yeast	8823154
aldehyde dehydrogenase (NAD+)	1.2.1.3	Yeast	8850269
aldehyde dehydrogenase (NAD+)	1.2.1.3	Yeast	9013560
aldehyde dehydrogenase (NAD+)	1.2.1.3	Yeast	9228057
aldehyde dehydrogenase (NAD+)	1.2.1.3	Yeast	945270
N-acetyl-gamma-glutamyl-phosphate reductase	1.2.1.38	Yeast	14602584
pyruvate dehydrogenase (acetyl-transferring)	1.2.4.1	Yeast	10720420
pyruvate dehydrogenase (acetyl-transferring)	1.2.4.1	Yeast	10759582
pyruvate dehydrogenase (acetyl-transferring)	1.2.4.1	Yeast	10826498
pyruvate dehydrogenase (acetyl-transferring)	1.2.4.1	Yeast	11223534
pyruvate dehydrogenase (acetyl-transferring)	1.2.4.1	Yeast	11427685
pyruvate dehydrogenase (acetyl-transferring)	1.2.4.1	Yeast	11557577
pyruvate dehydrogenase (acetyl-transferring)	1.2.4.1	Yeast	11866475
pyruvate dehydrogenase (acetyl-transferring)	1.2.4.1	Yeast	11900279

pyruvate dehydrogenase (acetyl-transferring)	1.2.4.1	Yeast	12196468
pyruvate dehydrogenase (acetyl-transferring)	1.2.4.1	Yeast	12557749
pyruvate dehydrogenase (acetyl-transferring)	1.2.4.1	Yeast	12663261
pyruvate dehydrogenase (acetyl-transferring)	1.2.4.1	Yeast	14607783
pyruvate dehydrogenase (acetyl-transferring)	1.2.4.1	Yeast	14641018
pyruvate dehydrogenase (acetyl-transferring)	1.2.4.1	Yeast	15191896
pyruvate dehydrogenase (acetyl-transferring)	1.2.4.1	Yeast	15256563
pyruvate dehydrogenase (acetyl-transferring)	1.2.4.1	Yeast	15921824
pyruvate dehydrogenase (acetyl-transferring)	1.2.4.1	Yeast	1600837
pyruvate dehydrogenase (acetyl-transferring)	1.2.4.1	Yeast	17065338
pyruvate dehydrogenase (acetyl-transferring)	1.2.4.1	Yeast	2902801
pyruvate dehydrogenase (acetyl-transferring)	1.2.4.1	Yeast	3127087
pyruvate dehydrogenase (acetyl-transferring)	1.2.4.1	Yeast	3918587
pyruvate dehydrogenase (acetyl-transferring)	1.2.4.1	Yeast	4030556
pyruvate dehydrogenase (acetyl-transferring)	1.2.4.1	Yeast	6149743
pyruvate dehydrogenase (acetyl-transferring)	1.2.4.1	Yeast	6234885
pyruvate dehydrogenase (acetyl-transferring)	1.2.4.1	Yeast	7127258
pyruvate dehydrogenase (acetyl-transferring)	1.2.4.1	Yeast	7848280
pyruvate dehydrogenase (acetyl-transferring)	1.2.4.1	Yeast	7864215
pyruvate dehydrogenase (acetyl-transferring)	1.2.4.1	Yeast	8557765
pyruvate dehydrogenase (acetyl-transferring)	1.2.4.1	Yeast	9089387
pyruvate dehydrogenase (acetyl-transferring)	1.2.4.1	Yeast	9381974
pyruvate dehydrogenase (acetyl-transferring)	1.2.4.1	Yeast	9426381
pyruvate dehydrogenase (acetyl-transferring)	1.2.4.1	Yeast	9438382
pyruvate dehydrogenase (acetyl-transferring)	1.2.4.1	Yeast	9497164
pyruvate dehydrogenase (acetyl-transferring)	1.2.4.1	Yeast	9725804
oxoglutarate dehydrogenase (succinyl-transferring)	1.2.4.2	Yeast	1352447
oxoglutarate dehydrogenase (succinyl-transferring)	1.2.4.2	Yeast	15356188
oxoglutarate dehydrogenase (succinyl-transferring)	1.2.4.2	Yeast	15466852
oxoglutarate dehydrogenase (succinyl-transferring)	1.2.4.2	Yeast	423807
oxoglutarate dehydrogenase (succinyl-transferring)	1.2.4.2	Yeast	6778477
oxoglutarate dehydrogenase (succinyl-transferring)	1.2.4.2	Yeast	7995856
enoyl-[acyl-carrier-protein] reductase (NADPH, B-specific)	1.3.1.10	Yeast	17095231
enoyl-[acyl-carrier-protein] reductase (NADPH, B-specific)	1.3.1.10	Yeast	8075395

enoyl-[acyl-carrier-protein] reductase (NADH)	1.3.1.9	Yeast	17095231
enoyl-[acyl-carrier-protein] reductase (NADH)	1.3.1.9	Yeast	8075395
dihydroorotate oxidase	1.3.3.1	Yeast	10348793
dihydroorotate oxidase	1.3.3.1	Yeast	10473079
dihydroorotate oxidase	1.3.3.1	Yeast	10848981
dihydroorotate oxidase	1.3.3.1	Yeast	10853015
dihydroorotate oxidase	1.3.3.1	Yeast	10878295
dihydroorotate oxidase	1.3.3.1	Yeast	10887632
dihydroorotate oxidase	1.3.3.1	Yeast	11053058
dihydroorotate oxidase	1.3.3.1	Yeast	11060767
dihydroorotate oxidase	1.3.3.1	Yeast	11098485
dihydroorotate oxidase	1.3.3.1	Yeast	11219978
dihydroorotate oxidase	1.3.3.1	Yeast	11267945
dihydroorotate oxidase	1.3.3.1	Yeast	11334264
dihydroorotate oxidase	1.3.3.1	Yeast	11376565
dihydroorotate oxidase	1.3.3.1	Yeast	11522581
dihydroorotate oxidase	1.3.3.1	Yeast	11555601
dihydroorotate oxidase	1.3.3.1	Yeast	11697836
dihydroorotate oxidase	1.3.3.1	Yeast	11796730
dihydroorotate oxidase	1.3.3.1	Yeast	11862480
dihydroorotate oxidase	1.3.3.1	Yeast	11865631
dihydroorotate oxidase	1.3.3.1	Yeast	11936689
dihydroorotate oxidase	1.3.3.1	Yeast	11962674
dihydroorotate oxidase	1.3.3.1	Yeast	12084458
dihydroorotate oxidase	1.3.3.1	Yeast	12187768
dihydroorotate oxidase	1.3.3.1	Yeast	12469154
dihydroorotate oxidase	1.3.3.1	Yeast	12527935
dihydroorotate oxidase	1.3.3.1	Yeast	12634225
dihydroorotate oxidase	1.3.3.1	Yeast	12820455
dihydroorotate oxidase	1.3.3.1	Yeast	12885330
dihydroorotate oxidase	1.3.3.1	Yeast	12967482
dihydroorotate oxidase	1.3.3.1	Yeast	14689231
dihydroorotate oxidase	1.3.3.1	Yeast	14744810
dihydroorotate oxidase	1.3.3.1	Yeast	15025949
dihydroorotate oxidase	1.3.3.1	Yeast	15045945
dihydroorotate oxidase	1.3.3.1	Yeast	15069534
dihydroorotate oxidase	1.3.3.1	Yeast	15134221
dihydroorotate oxidase	1.3.3.1	Yeast	15222105
dihydroorotate oxidase	1.3.3.1	Yeast	15450176
dihydroorotate oxidase	1.3.3.1	Yeast	15944938
dihydroorotate oxidase	1.3.3.1	Yeast	16163233

dihydroorotate oxidase	1.3.3.1	Yeast	1629785
dihydroorotate oxidase	1.3.3.1	Yeast	16556484
dihydroorotate oxidase	1.3.3.1	Yeast	17046731
dihydroorotate oxidase	1.3.3.1	Yeast	2060083
dihydroorotate oxidase	1.3.3.1	Yeast	3202908
dihydroorotate oxidase	1.3.3.1	Yeast	6761622
dihydroorotate oxidase	1.3.3.1	Yeast	7602356
dihydroorotate oxidase	1.3.3.1	Yeast	7825962
dihydroorotate oxidase	1.3.3.1	Yeast	8093030
dihydroorotate oxidase	1.3.3.1	Yeast	8097697
dihydroorotate oxidase	1.3.3.1	Yeast	8878781
dihydroorotate oxidase	1.3.3.1	Yeast	8895681
dihydroorotate oxidase	1.3.3.1	Yeast	8950202
dihydroorotate oxidase	1.3.3.1	Yeast	9056186
dihydroorotate oxidase	1.3.3.1	Yeast	9182832
dihydroorotate oxidase	1.3.3.1	Yeast	9280881
dihydroorotate oxidase	1.3.3.1	Yeast	9464498
dihydroorotate oxidase	1.3.3.1	Yeast	9597696
dihydroorotate oxidase	1.3.3.1	Yeast	9636062
dihydroorotate oxidase	1.3.3.1	Yeast	9819714
dihydroorotate oxidase	1.3.3.1	Yeast	9860876
dihydroorotate oxidase	1.3.3.1	Yeast	9914783
coproporphyrinogen oxidase	1.3.3.3	Yeast	10787385
coproporphyrinogen oxidase	1.3.3.3	Yeast	11368326
coproporphyrinogen oxidase	1.3.3.3	Yeast	16567402
acyl-CoA oxidase	1.3.3.6	Yeast	10318668
acyl-CoA oxidase	1.3.3.6	Yeast	11156684
acyl-CoA oxidase	1.3.3.6	Yeast	11330046
acyl-CoA oxidase	1.3.3.6	Yeast	12538078
acyl-CoA oxidase	1.3.3.6	Yeast	12758125
acyl-CoA oxidase	1.3.3.6	Yeast	14500732
acyl-CoA oxidase	1.3.3.6	Yeast	15805059
acyl-CoA oxidase	1.3.3.6	Yeast	1989516
acyl-CoA oxidase	1.3.3.6	Yeast	2049482
acyl-CoA oxidase	1.3.3.6	Yeast	2811611
acyl-CoA oxidase	1.3.3.6	Yeast	3367697
acyl-CoA oxidase	1.3.3.6	Yeast	3732222
acyl-CoA oxidase	1.3.3.6	Yeast	518563
acyl-CoA oxidase	1.3.3.6	Yeast	6240978
acyl-CoA oxidase	1.3.3.6	Yeast	6540549
acyl-CoA oxidase	1.3.3.6	Yeast	6541949
acyl-CoA oxidase	1.3.3.6	Yeast	7860752
acyl-CoA oxidase	1.3.3.6	Yeast	7867664

acyl-CoA oxidase	1.3.3.6	Yeast	8662598
acyl-CoA oxidase	1.3.3.6	Yeast	8784738
acyl-CoA oxidase	1.3.3.6	Yeast	8798738
acyl-CoA oxidase	1.3.3.6	Yeast	8895731
glutamate dehydrogenase	1.4.1.2	Yeast	3139028
glutamate dehydrogenase	1.4.1.2	Yeast	7075604
glutamate dehydrogenase	1.4.1.2	Yeast	7075605
glutamate dehydrogenase	1.4.1.2	Yeast	7075606
glutamate dehydrogenase	1.4.1.2	Yeast	7075607
glutamate dehydrogenase	1.4.1.2	Yeast	7470041
glutamate dehydrogenase	1.4.1.2	Yeast	7918368
glutamate dehydrogenase	1.4.1.2	Yeast	8122033
glutamate dehydrogenase (NADP+)	1.4.1.4	Yeast	10323225
glutamate dehydrogenase (NADP+)	1.4.1.4	Yeast	1576153
glutamate dehydrogenase (NADP+)	1.4.1.4	Yeast	3139028
glutamate dehydrogenase (NADP+)	1.4.1.4	Yeast	6116360
glutamate dehydrogenase (NADP+)	1.4.1.4	Yeast	7470041
glutamate dehydrogenase (NADP+)	1.4.1.4	Yeast	7765894
glutamate dehydrogenase (NADP+)	1.4.1.4	Yeast	8122033
glutamate dehydrogenase (NADP+)	1.4.1.4	Yeast	9918511
pyridoxal 5'-phosphate synthase	1.4.3.5	Yeast	6822512
pyridoxal 5'-phosphate synthase	1.4.3.5	Yeast	9601034
1-pyrroline-5-carboxylate dehydrogenase	1.5.1.12	Yeast	10363370
1-pyrroline-5-carboxylate dehydrogenase	1.5.1.12	Yeast	10398729
1-pyrroline-5-carboxylate dehydrogenase	1.5.1.12	Yeast	10441499
1-pyrroline-5-carboxylate dehydrogenase	1.5.1.12	Yeast	10759508
1-pyrroline-5-carboxylate dehydrogenase	1.5.1.12	Yeast	10945345
1-pyrroline-5-carboxylate dehydrogenase	1.5.1.12	Yeast	11726714
1-pyrroline-5-carboxylate dehydrogenase	1.5.1.12	Yeast	12602867
1-pyrroline-5-carboxylate dehydrogenase	1.5.1.12	Yeast	14602584
1-pyrroline-5-carboxylate dehydrogenase	1.5.1.12	Yeast	15077666
1-pyrroline-5-carboxylate dehydrogenase	1.5.1.12	Yeast	2860838
1-pyrroline-5-carboxylate dehydrogenase	1.5.1.12	Yeast	9351242
methylenetetrahydrofolate dehydrogenase (NAD+)	1.5.1.15	Yeast	718836
methylenetetrahydrofolate reductase [NAD(P)H]	1.5.1.20	Yeast	10462593
methylenetetrahydrofolate reductase [NAD(P)H]	1.5.1.20	Yeast	10948708
methylenetetrahydrofolate reductase [NAD(P)H]	1.5.1.20	Yeast	11302003
methylenetetrahydrofolate reductase [NAD(P)H]	1.5.1.20	Yeast	11343335

methylenetetrahydrofolate reductase [NAD(P)H]	1.5.1.20	Yeast	11398138
methylenetetrahydrofolate reductase [NAD(P)H]	1.5.1.20	Yeast	11680544
methylenetetrahydrofolate reductase [NAD(P)H]	1.5.1.20	Yeast	11712321
methylenetetrahydrofolate reductase [NAD(P)H]	1.5.1.20	Yeast	11927833
methylenetetrahydrofolate reductase [NAD(P)H]	1.5.1.20	Yeast	12028998
methylenetetrahydrofolate reductase [NAD(P)H]	1.5.1.20	Yeast	12038037
methylenetetrahydrofolate reductase [NAD(P)H]	1.5.1.20	Yeast	12600862
methylenetetrahydrofolate reductase [NAD(P)H]	1.5.1.20	Yeast	12897091
methylenetetrahydrofolate reductase [NAD(P)H]	1.5.1.20	Yeast	12914571
methylenetetrahydrofolate reductase [NAD(P)H]	1.5.1.20	Yeast	14608052
methylenetetrahydrofolate reductase [NAD(P)H]	1.5.1.20	Yeast	14728017
methylenetetrahydrofolate reductase [NAD(P)H]	1.5.1.20	Yeast	14973104
methylenetetrahydrofolate reductase [NAD(P)H]	1.5.1.20	Yeast	15033905
methylenetetrahydrofolate reductase [NAD(P)H]	1.5.1.20	Yeast	15207432
methylenetetrahydrofolate reductase [NAD(P)H]	1.5.1.20	Yeast	15449187
methylenetetrahydrofolate reductase [NAD(P)H]	1.5.1.20	Yeast	15546509
methylenetetrahydrofolate reductase [NAD(P)H]	1.5.1.20	Yeast	15581487
methylenetetrahydrofolate reductase [NAD(P)H]	1.5.1.20	Yeast	15598763
methylenetetrahydrofolate reductase [NAD(P)H]	1.5.1.20	Yeast	15688606
methylenetetrahydrofolate reductase [NAD(P)H]	1.5.1.20	Yeast	15773669
methylenetetrahydrofolate reductase [NAD(P)H]	1.5.1.20	Yeast	15894672

methylenetetrahydrofolate reductase [NAD(P)H]	1.5.1.20	Yeast	15935452
methylenetetrahydrofolate reductase [NAD(P)H]	1.5.1.20	Yeast	16055944
methylenetetrahydrofolate reductase [NAD(P)H]	1.5.1.20	Yeast	16108833
methylenetetrahydrofolate reductase [NAD(P)H]	1.5.1.20	Yeast	16128738
methylenetetrahydrofolate reductase [NAD(P)H]	1.5.1.20	Yeast	16274753
methylenetetrahydrofolate reductase [NAD(P)H]	1.5.1.20	Yeast	16538645
methylenetetrahydrofolate reductase [NAD(P)H]	1.5.1.20	Yeast	16706930
methylenetetrahydrofolate reductase [NAD(P)H]	1.5.1.20	Yeast	17105984
methylenetetrahydrofolate reductase [NAD(P)H]	1.5.1.20	Yeast	3143307
methylenetetrahydrofolate reductase [NAD(P)H]	1.5.1.20	Yeast	6352699
methylenetetrahydrofolate reductase [NAD(P)H]	1.5.1.20	Yeast	6391540
methylenetetrahydrofolate reductase [NAD(P)H]	1.5.1.20	Yeast	9789068
proline dehydrogenase	1.5.99.8	Yeast	11788754
proline dehydrogenase	1.5.99.8	Yeast	12602867
dihydrolipoyl dehydrogenase	1.8.1.4	Yeast	10885793
dihydrolipoyl dehydrogenase	1.8.1.4	Yeast	11641455
dihydrolipoyl dehydrogenase	1.8.1.4	Yeast	12812918
dihydrolipoyl dehydrogenase	1.8.1.4	Yeast	15173434
dihydrolipoyl dehydrogenase	1.8.1.4	Yeast	15915669
dihydrolipoyl dehydrogenase	1.8.1.4	Yeast	16581023
dihydrolipoyl dehydrogenase	1.8.1.4	Yeast	16616211
dihydrolipoyl dehydrogenase	1.8.1.4	Yeast	16875466
dihydrolipoyl dehydrogenase	1.8.1.4	Yeast	2381301
dihydrolipoyl dehydrogenase	1.8.1.4	Yeast	2404025
dihydrolipoyl dehydrogenase	1.8.1.4	Yeast	3103467
dihydrolipoyl dehydrogenase	1.8.1.4	Yeast	3425711
dihydrolipoyl dehydrogenase	1.8.1.4	Yeast	7782942
glutathione-disulfide reductase	1.8.1.7	Yeast	11133045
glutathione-disulfide reductase	1.8.1.7	Yeast	12204336
glutathione-disulfide reductase	1.8.1.7	Yeast	12453665
glutathione-disulfide reductase	1.8.1.7	Yeast	1605642

glutathione-disulfide reductase	1.8.1.7	Yeast	2848577
glutathione-disulfide reductase	1.8.1.7	Yeast	3698652
glutathione-disulfide reductase	1.8.1.7	Yeast	3963383
glutathione-disulfide reductase	1.8.1.7	Yeast	8843715
glutathione-disulfide reductase	1.8.1.7	Yeast	9350472
cytochrome-c oxidase	1.9.3.1	Yeast	10490029
cytochrome-c oxidase	1.9.3.1	Yeast	11988227
cytochrome-c oxidase	1.9.3.1	Yeast	12059041
cytochrome-c oxidase	1.9.3.1	Yeast	12145150
cytochrome-c oxidase	1.9.3.1	Yeast	12874793
cytochrome-c oxidase	1.9.3.1	Yeast	1315683
cytochrome-c oxidase	1.9.3.1	Yeast	1450614
cytochrome-c oxidase	1.9.3.1	Yeast	15504366
cytochrome-c oxidase	1.9.3.1	Yeast	15708625
cytochrome-c oxidase	1.9.3.1	Yeast	16704969
cytochrome-c oxidase	1.9.3.1	Yeast	167928
cytochrome-c oxidase	1.9.3.1	Yeast	174553
cytochrome-c oxidase	1.9.3.1	Yeast	194851
cytochrome-c oxidase	1.9.3.1	Yeast	206437
cytochrome-c oxidase	1.9.3.1	Yeast	2822680
cytochrome-c oxidase	1.9.3.1	Yeast	2849368
cytochrome-c oxidase	1.9.3.1	Yeast	2854388
cytochrome-c oxidase	1.9.3.1	Yeast	3000820
cytochrome-c oxidase	1.9.3.1	Yeast	3002436
cytochrome-c oxidase	1.9.3.1	Yeast	356174
cytochrome-c oxidase	1.9.3.1	Yeast	3569141
cytochrome-c oxidase	1.9.3.1	Yeast	3924042
cytochrome-c oxidase	1.9.3.1	Yeast	6091751
cytochrome-c oxidase	1.9.3.1	Yeast	6254961
cytochrome-c oxidase	1.9.3.1	Yeast	6268138
cytochrome-c oxidase	1.9.3.1	Yeast	6286610
cytochrome-c oxidase	1.9.3.1	Yeast	6320180
cytochrome-c oxidase	1.9.3.1	Yeast	7356983
cytochrome-c oxidase	1.9.3.1	Yeast	8013452
cytochrome-c oxidase	1.9.3.1	Yeast	8386021
phosphatidylethanolamine N-methyltransferase	2.1.1.17	Yeast	8207329
phosphatidylethanolamine N-methyltransferase	2.1.1.17	Yeast	9380436
sterol 24-C-methyltransferase	2.1.1.41	Yeast	8621604
thymidylate synthase	2.1.1.45	Yeast	11358693
thymidylate synthase	2.1.1.45	Yeast	12412165
thymidylate synthase	2.1.1.45	Yeast	12457437

thymidylate synthase	2.1.1.45	Yeast	12470718
thymidylate synthase	2.1.1.45	Yeast	12544347
thymidylate synthase	2.1.1.45	Yeast	14578129
thymidylate synthase	2.1.1.45	Yeast	14648018
thymidylate synthase	2.1.1.45	Yeast	14689231
thymidylate synthase	2.1.1.45	Yeast	15025949
thymidylate synthase	2.1.1.45	Yeast	15134221
thymidylate synthase	2.1.1.45	Yeast	15598787
thymidylate synthase	2.1.1.45	Yeast	16077970
thymidylate synthase	2.1.1.45	Yeast	16540728
thymidylate synthase	2.1.1.45	Yeast	16617381
thymidylate synthase	2.1.1.45	Yeast	3709927
thymidylate synthase	2.1.1.45	Yeast	7602356
thymidylate synthase	2.1.1.45	Yeast	8805515
phosphoribosylaminoimidazolecarboxamide formyltransferase	2.1.2.3	Yeast	6335666
aspartate carbamoyltransferase	2.1.3.2	Yeast	10336386
aspartate carbamoyltransferase	2.1.3.2	Yeast	10593256
aspartate carbamoyltransferase	2.1.3.2	Yeast	11397099
aspartate carbamoyltransferase	2.1.3.2	Yeast	15165857
aspartate carbamoyltransferase	2.1.3.2	Yeast	15529744
aspartate carbamoyltransferase	2.1.3.2	Yeast	16120448
aspartate carbamoyltransferase	2.1.3.2	Yeast	17008138
aspartate carbamoyltransferase	2.1.3.2	Yeast	3047117
aspartate carbamoyltransferase	2.1.3.2	Yeast	3542019
aspartate carbamoyltransferase	2.1.3.2	Yeast	3907993
aspartate carbamoyltransferase	2.1.3.2	Yeast	4092695
aspartate carbamoyltransferase	2.1.3.2	Yeast	6115855
aspartate carbamoyltransferase	2.1.3.2	Yeast	6298785
aspartate carbamoyltransferase	2.1.3.2	Yeast	7051000
aspartate carbamoyltransferase	2.1.3.2	Yeast	9611817
aspartate carbamoyltransferase	2.1.3.2	Yeast	9626698
aspartate carbamoyltransferase	2.1.3.2	Yeast	9659392
ornithine carbamoyltransferase	2.1.3.3	Yeast	1505922
ornithine carbamoyltransferase	2.1.3.3	Yeast	205703
ornithine carbamoyltransferase	2.1.3.3	Yeast	2667139
ornithine carbamoyltransferase	2.1.3.3	Yeast	2667140
ornithine carbamoyltransferase	2.1.3.3	Yeast	2667141
ornithine carbamoyltransferase	2.1.3.3	Yeast	8019156
ornithine carbamoyltransferase	2.1.3.3	Yeast	8168544
ornithine carbamoyltransferase	2.1.3.3	Yeast	9501170
ornithine carbamoyltransferase	2.1.3.3	Yeast	9540805
transketolase	2.2.1.1	Yeast	10975072

transketolase	2.2.1.1	Yeast	11072071
transketolase	2.2.1.1	Yeast	16354724
transketolase	2.2.1.1	Yeast	1939098
transketolase	2.2.1.1	Yeast	9924800
transaldolase	2.2.1.2	Yeast	11390181
transaldolase	2.2.1.2	Yeast	12359249
transaldolase	2.2.1.2	Yeast	15263091
transaldolase	2.2.1.2	Yeast	16092052
transaldolase	2.2.1.2	Yeast	17046540
transaldolase	2.2.1.2	Yeast	9565623
transaldolase	2.2.1.2	Yeast	9973403
dihydrolipoyllysine-residue acetyltransferase	2.3.1.12	Yeast	3840997
acetyl-CoA C-acyltransferase	2.3.1.16	Yeast	3967008
acetyl-CoA C-acyltransferase	2.3.1.16	Yeast	7068598
acetyl-CoA C-acyltransferase	2.3.1.16	Yeast	7374368
sphingosine N-acyltransferase	2.3.1.24	Yeast	10575354
sphingosine N-acyltransferase	2.3.1.24	Yeast	10822508
sphingosine N-acyltransferase	2.3.1.24	Yeast	10862608
sphingosine N-acyltransferase	2.3.1.24	Yeast	10930700
sphingosine N-acyltransferase	2.3.1.24	Yeast	11958538
sphingosine N-acyltransferase	2.3.1.24	Yeast	12383489
sphingosine N-acyltransferase	2.3.1.24	Yeast	12676494
sphingosine N-acyltransferase	2.3.1.24	Yeast	8078996
sphingosine N-acyltransferase	2.3.1.24	Yeast	8090377
sphingosine N-acyltransferase	2.3.1.24	Yeast	8658522
sterol O-acyltransferase	2.3.1.26	Yeast	10593897
sterol O-acyltransferase	2.3.1.26	Yeast	10656290
sterol O-acyltransferase	2.3.1.26	Yeast	11888294
sterol O-acyltransferase	2.3.1.26	Yeast	12787409
sterol O-acyltransferase	2.3.1.26	Yeast	1450216
sterol O-acyltransferase	2.3.1.26	Yeast	2885178
sterol O-acyltransferase	2.3.1.26	Yeast	3593752
sterol O-acyltransferase	2.3.1.26	Yeast	3719008
sterol O-acyltransferase	2.3.1.26	Yeast	3766727
sterol O-acyltransferase	2.3.1.26	Yeast	3812206
sterol O-acyltransferase	2.3.1.26	Yeast	3926761
sterol O-acyltransferase	2.3.1.26	Yeast	4056052
sterol O-acyltransferase	2.3.1.26	Yeast	6347025
sterol O-acyltransferase	2.3.1.26	Yeast	8258956
sterol O-acyltransferase	2.3.1.26	Yeast	8466946
sterol O-acyltransferase	2.3.1.26	Yeast	8820097
sterol O-acyltransferase	2.3.1.26	Yeast	9857049
glutamate N-acetyltransferase	2.3.1.35	Yeast	10931207

glutamate N-acetyltransferase	2.3.1.35	Yeast	16664327
glutamate N-acetyltransferase	2.3.1.35	Yeast	17347518
glutamate N-acetyltransferase	2.3.1.35	Yeast	17651682
glutamate N-acetyltransferase	2.3.1.35	Yeast	361732
5-aminolevulinate synthase	2.3.1.37	Yeast	10634305
5-aminolevulinate synthase	2.3.1.37	Yeast	10727444
5-aminolevulinate synthase	2.3.1.37	Yeast	10729988
5-aminolevulinate synthase	2.3.1.37	Yeast	10787385
5-aminolevulinate synthase	2.3.1.37	Yeast	10825473
5-aminolevulinate synthase	2.3.1.37	Yeast	11202048
5-aminolevulinate synthase	2.3.1.37	Yeast	11202050
5-aminolevulinate synthase	2.3.1.37	Yeast	11368326
5-aminolevulinate synthase	2.3.1.37	Yeast	12121995
5-aminolevulinate synthase	2.3.1.37	Yeast	12393745
5-aminolevulinate synthase	2.3.1.37	Yeast	12469218
5-aminolevulinate synthase	2.3.1.37	Yeast	12627002
5-aminolevulinate synthase	2.3.1.37	Yeast	126586
5-aminolevulinate synthase	2.3.1.37	Yeast	12881517
5-aminolevulinate synthase	2.3.1.37	Yeast	14643887
5-aminolevulinate synthase	2.3.1.37	Yeast	1511083
5-aminolevulinate synthase	2.3.1.37	Yeast	15178759
5-aminolevulinate synthase	2.3.1.37	Yeast	15259603
5-aminolevulinate synthase	2.3.1.37	Yeast	1526942
5-aminolevulinate synthase	2.3.1.37	Yeast	15547665
5-aminolevulinate synthase	2.3.1.37	Yeast	15797241
5-aminolevulinate synthase	2.3.1.37	Yeast	15972158
5-aminolevulinate synthase	2.3.1.37	Yeast	16122419
5-aminolevulinate synthase	2.3.1.37	Yeast	16181105
5-aminolevulinate synthase	2.3.1.37	Yeast	16567402
5-aminolevulinate synthase	2.3.1.37	Yeast	16846079
5-aminolevulinate synthase	2.3.1.37	Yeast	1688552
5-aminolevulinate synthase	2.3.1.37	Yeast	16892088
5-aminolevulinate synthase	2.3.1.37	Yeast	16904069
5-aminolevulinate synthase	2.3.1.37	Yeast	1954232
5-aminolevulinate synthase	2.3.1.37	Yeast	1959865
5-aminolevulinate synthase	2.3.1.37	Yeast	2050126
5-aminolevulinate synthase	2.3.1.37	Yeast	2241158
5-aminolevulinate synthase	2.3.1.37	Yeast	2317819
5-aminolevulinate synthase	2.3.1.37	Yeast	266710
5-aminolevulinate synthase	2.3.1.37	Yeast	3009001
5-aminolevulinate synthase	2.3.1.37	Yeast	3094379
5-aminolevulinate synthase	2.3.1.37	Yeast	3359971
5-aminolevulinate synthase	2.3.1.37	Yeast	3755290

5-aminolevulinate synthase	2.3.1.37	Yeast	3840094
5-aminolevulinate synthase	2.3.1.37	Yeast	3966797
5-aminolevulinate synthase	2.3.1.37	Yeast	6092369
5-aminolevulinate synthase	2.3.1.37	Yeast	6213232
5-aminolevulinate synthase	2.3.1.37	Yeast	6547609
5-aminolevulinate synthase	2.3.1.37	Yeast	6824732
5-aminolevulinate synthase	2.3.1.37	Yeast	6873612
5-aminolevulinate synthase	2.3.1.37	Yeast	6954546
5-aminolevulinate synthase	2.3.1.37	Yeast	7093306
5-aminolevulinate synthase	2.3.1.37	Yeast	7592563
5-aminolevulinate synthase	2.3.1.37	Yeast	8076930
5-aminolevulinate synthase	2.3.1.37	Yeast	818637
5-aminolevulinate synthase	2.3.1.37	Yeast	8385933
5-aminolevulinate synthase	2.3.1.37	Yeast	8413301
5-aminolevulinate synthase	2.3.1.37	Yeast	9169013
5-aminolevulinate synthase	2.3.1.37	Yeast	9173682
5-aminolevulinate synthase	2.3.1.37	Yeast	9806796
5-aminolevulinate synthase	2.3.1.37	Yeast	9879810
serine C-palmitoyltransferase	2.3.1.50	Yeast	10722759
serine C-palmitoyltransferase	2.3.1.50	Yeast	10736421
serine C-palmitoyltransferase	2.3.1.50	Yeast	10764732
serine C-palmitoyltransferase	2.3.1.50	Yeast	10818445
serine C-palmitoyltransferase	2.3.1.50	Yeast	10862608
serine C-palmitoyltransferase	2.3.1.50	Yeast	10971324
serine C-palmitoyltransferase	2.3.1.50	Yeast	11279212
serine C-palmitoyltransferase	2.3.1.50	Yeast	11903061
serine C-palmitoyltransferase	2.3.1.50	Yeast	12445191
serine C-palmitoyltransferase	2.3.1.50	Yeast	12531548
serine C-palmitoyltransferase	2.3.1.50	Yeast	12570999
serine C-palmitoyltransferase	2.3.1.50	Yeast	12612207
serine C-palmitoyltransferase	2.3.1.50	Yeast	12686119
serine C-palmitoyltransferase	2.3.1.50	Yeast	12704216
serine C-palmitoyltransferase	2.3.1.50	Yeast	12736045
serine C-palmitoyltransferase	2.3.1.50	Yeast	12782147
serine C-palmitoyltransferase	2.3.1.50	Yeast	14563682
serine C-palmitoyltransferase	2.3.1.50	Yeast	14744154
serine C-palmitoyltransferase	2.3.1.50	Yeast	15066023
serine C-palmitoyltransferase	2.3.1.50	Yeast	15180163
serine C-palmitoyltransferase	2.3.1.50	Yeast	15297021
serine C-palmitoyltransferase	2.3.1.50	Yeast	15545514
serine C-palmitoyltransferase	2.3.1.50	Yeast	15560753
serine C-palmitoyltransferase	2.3.1.50	Yeast	15562249
serine C-palmitoyltransferase	2.3.1.50	Yeast	16117797

serine C-palmitoyltransferase	2.3.1.50	Yeast	16157870
serine C-palmitoyltransferase	2.3.1.50	Yeast	16210380
serine C-palmitoyltransferase	2.3.1.50	Yeast	16216550
serine C-palmitoyltransferase	2.3.1.50	Yeast	17090526
serine C-palmitoyltransferase	2.3.1.50	Yeast	9186561
serine C-palmitoyltransferase	2.3.1.50	Yeast	9363775
serine C-palmitoyltransferase	2.3.1.50	Yeast	9405408
serine C-palmitoyltransferase	2.3.1.50	Yeast	9714132
serine C-palmitoyltransferase	2.3.1.50	Yeast	9788249
acetyl-CoA C-acetyltransferase	2.3.1.9	Yeast	14693556
acetyl-CoA C-acetyltransferase	2.3.1.9	Yeast	15135409
acetyl-CoA C-acetyltransferase	2.3.1.9	Yeast	15466479
acetyl-CoA C-acetyltransferase	2.3.1.9	Yeast	2869784
gamma-glutamyltransferase	2.3.2.2	Yeast	11810401
gamma-glutamyltransferase	2.3.2.2	Yeast	12030366
gamma-glutamyltransferase	2.3.2.2	Yeast	12468440
gamma-glutamyltransferase	2.3.2.2	Yeast	12780970
gamma-glutamyltransferase	2.3.2.2	Yeast	15006645
gamma-glutamyltransferase	2.3.2.2	Yeast	16302185
gamma-glutamyltransferase	2.3.2.2	Yeast	2881890
gamma-glutamyltransferase	2.3.2.2	Yeast	2903803
gamma-glutamyltransferase	2.3.2.2	Yeast	7485380
gamma-glutamyltransferase	2.3.2.2	Yeast	8067452
gamma-glutamyltransferase	2.3.2.2	Yeast	8564390
gamma-glutamyltransferase	2.3.2.2	Yeast	8972486
gamma-glutamyltransferase	2.3.2.2	Yeast	9974125
citrate (Si)-synthase	2.3.3.1	Yeast	1004246
citrate (Si)-synthase	2.3.3.1	Yeast	11842094
citrate (Si)-synthase	2.3.3.1	Yeast	11872452
citrate (Si)-synthase	2.3.3.1	Yeast	15994367
citrate (Si)-synthase	2.3.3.1	Yeast	16269721
citrate (Si)-synthase	2.3.3.1	Yeast	3776117
citrate (Si)-synthase	2.3.3.1	Yeast	3916224
citrate (Si)-synthase	2.3.3.1	Yeast	6799496
citrate (Si)-synthase	2.3.3.1	Yeast	8526514
citrate (Si)-synthase	2.3.3.1	Yeast	9353808
citrate (Si)-synthase	2.3.3.1	Yeast	9554114
hydroxymethylglutaryl-CoA synthase	2.3.3.10	Yeast	11160362
hydroxymethylglutaryl-CoA synthase	2.3.3.10	Yeast	11485325
hydroxymethylglutaryl-CoA synthase	2.3.3.10	Yeast	16101500
hydroxymethylglutaryl-CoA synthase	2.3.3.10	Yeast	16864776
hydroxymethylglutaryl-CoA synthase	2.3.3.10	Yeast	16962226
hydroxymethylglutaryl-CoA synthase	2.3.3.10	Yeast	475

hydroxymethylglutaryl-CoA synthase	2.3.3.10	Yeast	7907092
phosphorylase	2.4.1.1	Yeast	10548038
phosphorylase	2.4.1.1	Yeast	11391834
phosphorylase	2.4.1.1	Yeast	11391835
phosphorylase	2.4.1.1	Yeast	11391836
phosphorylase	2.4.1.1	Yeast	11391837
phosphorylase	2.4.1.1	Yeast	11391838
phosphorylase	2.4.1.1	Yeast	11391839
phosphorylase	2.4.1.1	Yeast	11391840
phosphorylase	2.4.1.1	Yeast	11391841
phosphorylase	2.4.1.1	Yeast	11391842
phosphorylase	2.4.1.1	Yeast	12769745
phosphorylase	2.4.1.1	Yeast	15299833
phosphorylase	2.4.1.1	Yeast	15721288
phosphorylase	2.4.1.1	Yeast	1691273
phosphorylase	2.4.1.1	Yeast	7664039
glycogen(starch) synthase	2.4.1.11	Yeast	10067873
glycogen(starch) synthase	2.4.1.11	Yeast	10222257
glycogen(starch) synthase	2.4.1.11	Yeast	10684630
glycogen(starch) synthase	2.4.1.11	Yeast	11181947
glycogen(starch) synthase	2.4.1.11	Yeast	11467410
glycogen(starch) synthase	2.4.1.11	Yeast	11534633
glycogen(starch) synthase	2.4.1.11	Yeast	11834204
glycogen(starch) synthase	2.4.1.11	Yeast	11900279
glycogen(starch) synthase	2.4.1.11	Yeast	12617691
glycogen(starch) synthase	2.4.1.11	Yeast	14570701
glycogen(starch) synthase	2.4.1.11	Yeast	15840572
glycogen(starch) synthase	2.4.1.11	Yeast	15932409
glycogen(starch) synthase	2.4.1.11	Yeast	16101290
glycogen(starch) synthase	2.4.1.11	Yeast	1756915
glycogen(starch) synthase	2.4.1.11	Yeast	17569761
glycogen(starch) synthase	2.4.1.11	Yeast	17698598
glycogen(starch) synthase	2.4.1.11	Yeast	1959479
glycogen(starch) synthase	2.4.1.11	Yeast	208368
glycogen(starch) synthase	2.4.1.11	Yeast	2115296
glycogen(starch) synthase	2.4.1.11	Yeast	2154910
glycogen(starch) synthase	2.4.1.11	Yeast	219866
glycogen(starch) synthase	2.4.1.11	Yeast	227915
glycogen(starch) synthase	2.4.1.11	Yeast	2405698
glycogen(starch) synthase	2.4.1.11	Yeast	2822414
glycogen(starch) synthase	2.4.1.11	Yeast	3032541
glycogen(starch) synthase	2.4.1.11	Yeast	3092743
glycogen(starch) synthase	2.4.1.11	Yeast	3143265

glycogen(starch) synthase	2.4.1.11	Yeast	3930321
glycogen(starch) synthase	2.4.1.11	Yeast	6409592
glycogen(starch) synthase	2.4.1.11	Yeast	6412593
glycogen(starch) synthase	2.4.1.11	Yeast	7010073
glycogen(starch) synthase	2.4.1.11	Yeast	7672505
glycogen(starch) synthase	2.4.1.11	Yeast	7983805
glycogen(starch) synthase	2.4.1.11	Yeast	8226927
glycogen(starch) synthase	2.4.1.11	Yeast	8416266
glycogen(starch) synthase	2.4.1.11	Yeast	8514767
glycogen(starch) synthase	2.4.1.11	Yeast	8514849
glycogen(starch) synthase	2.4.1.11	Yeast	8541012
glycogen(starch) synthase	2.4.1.11	Yeast	8569754
glycogen(starch) synthase	2.4.1.11	Yeast	8591890
glycogen(starch) synthase	2.4.1.11	Yeast	8593937
glycogen(starch) synthase	2.4.1.11	Yeast	8612539
glycogen(starch) synthase	2.4.1.11	Yeast	8645005
glycogen(starch) synthase	2.4.1.11	Yeast	8721777
glycogen(starch) synthase	2.4.1.11	Yeast	8769349
glycogen(starch) synthase	2.4.1.11	Yeast	9126490
glycogen(starch) synthase	2.4.1.11	Yeast	9162607
glycogen(starch) synthase	2.4.1.11	Yeast	9267990
glycogen(starch) synthase	2.4.1.11	Yeast	9389424
glycogen(starch) synthase	2.4.1.11	Yeast	9450985
glycogen(starch) synthase	2.4.1.11	Yeast	9609122
glycogen(starch) synthase	2.4.1.11	Yeast	9712712
nicotinate phosphoribosyltransferase	2.4.2.11	Yeast	10825532
amidophosphoribosyltransferase	2.4.2.14	Yeast	10675983
amidophosphoribosyltransferase	2.4.2.14	Yeast	11158364
amidophosphoribosyltransferase	2.4.2.14	Yeast	12930749
amidophosphoribosyltransferase	2.4.2.14	Yeast	15266056
amidophosphoribosyltransferase	2.4.2.14	Yeast	17434429
amidophosphoribosyltransferase	2.4.2.14	Yeast	214373
amidophosphoribosyltransferase	2.4.2.14	Yeast	6327016
amidophosphoribosyltransferase	2.4.2.14	Yeast	701284
amidophosphoribosyltransferase	2.4.2.14	Yeast	7683680
amidophosphoribosyltransferase	2.4.2.14	Yeast	8150282
amidophosphoribosyltransferase	2.4.2.14	Yeast	8197456
amidophosphoribosyltransferase	2.4.2.14	Yeast	8197457
amidophosphoribosyltransferase	2.4.2.14	Yeast	8197458
amidophosphoribosyltransferase	2.4.2.14	Yeast	8380692
amidophosphoribosyltransferase	2.4.2.14	Yeast	8463258
amidophosphoribosyltransferase	2.4.2.14	Yeast	8809759
amidophosphoribosyltransferase	2.4.2.14	Yeast	8976092

amidophosphoribosyltransferase	2.4.2.14	Yeast	9615746
amidophosphoribosyltransferase	2.4.2.14	Yeast	9881055
adenine phosphoribosyltransferase	2.4.2.7	Yeast	2154328
adenine phosphoribosyltransferase	2.4.2.7	Yeast	6327016
dimethylallyltranstransferase	2.5.1.1	Yeast	11442630
dimethylallyltranstransferase	2.5.1.1	Yeast	14512521
dimethylallyltranstransferase	2.5.1.1	Yeast	15597200
dimethylallyltranstransferase	2.5.1.1	Yeast	7697819
dimethylallyltranstransferase	2.5.1.1	Yeast	7843406
dimethylallyltranstransferase	2.5.1.1	Yeast	8631820
geranyltranstransferase	2.5.1.10	Yeast	10484604
geranyltranstransferase	2.5.1.10	Yeast	11202437
geranyltranstransferase	2.5.1.10	Yeast	15459425
geranyltranstransferase	2.5.1.10	Yeast	15605175
geranyltranstransferase	2.5.1.10	Yeast	15713990
geranyltranstransferase	2.5.1.10	Yeast	15827605
geranyltranstransferase	2.5.1.10	Yeast	15827618
geranyltranstransferase	2.5.1.10	Yeast	16179378
geranyltranstransferase	2.5.1.10	Yeast	16932286
geranyltranstransferase	2.5.1.10	Yeast	1779710
geranyltranstransferase	2.5.1.10	Yeast	9061016
geranyltranstransferase	2.5.1.10	Yeast	9640665
spermidine synthase	2.5.1.16	Yeast	16515550
spermidine synthase	2.5.1.16	Yeast	2775206
squalene synthase	2.5.1.21	Yeast	10649449
squalene synthase	2.5.1.21	Yeast	10677224
squalene synthase	2.5.1.21	Yeast	12114564
squalene synthase	2.5.1.21	Yeast	15356323
squalene synthase	2.5.1.21	Yeast	1601846
squalene synthase	2.5.1.21	Yeast	17016471
squalene synthase	2.5.1.21	Yeast	17531951
squalene synthase	2.5.1.21	Yeast	2068081
squalene synthase	2.5.1.21	Yeast	7766395
squalene synthase	2.5.1.21	Yeast	7843406
squalene synthase	2.5.1.21	Yeast	9070296
cystathionine gamma-synthase	2.5.1.48	Yeast	10558994
cystathionine gamma-synthase	2.5.1.48	Yeast	11842149
cystathionine gamma-synthase	2.5.1.48	Yeast	12692344
cystathionine gamma-synthase	2.5.1.48	Yeast	14618405
cystathionine gamma-synthase	2.5.1.48	Yeast	14622248
cystathionine gamma-synthase	2.5.1.48	Yeast	15270685
cystathionine gamma-synthase	2.5.1.48	Yeast	15581575
methionine adenosyltransferase	2.5.1.6	Yeast	10415148

methionine adenosyltransferase	2.5.1.6	Yeast	1511738
methionine adenosyltransferase	2.5.1.6	Yeast	16413417
methionine adenosyltransferase	2.5.1.6	Yeast	2764959
methionine adenosyltransferase	2.5.1.6	Yeast	7980467
hydroxymethylbilane synthase	2.5.1.61	Yeast	10546563
hydroxymethylbilane synthase	2.5.1.61	Yeast	10787385
hydroxymethylbilane synthase	2.5.1.61	Yeast	11953837
hydroxymethylbilane synthase	2.5.1.61	Yeast	14559249
hydroxymethylbilane synthase	2.5.1.61	Yeast	1522882
hydroxymethylbilane synthase	2.5.1.61	Yeast	16886091
hydroxymethylbilane synthase	2.5.1.61	Yeast	4067519
hydroxymethylbilane synthase	2.5.1.61	Yeast	7326026
hydroxymethylbilane synthase	2.5.1.61	Yeast	7682572
hydroxymethylbilane synthase	2.5.1.61	Yeast	8023693
hydroxymethylbilane synthase	2.5.1.61	Yeast	9065797
hydroxymethylbilane synthase	2.5.1.61	Yeast	9460994
aspartate transaminase	2.6.1.1	Yeast	8580353
ornithine aminotransferase	2.6.1.13	Yeast	11691635
ornithine aminotransferase	2.6.1.13	Yeast	12462748
ornithine aminotransferase	2.6.1.13	Yeast	7883744
glutamine---fructose-6-phosphate transaminase (isomerizing)	2.6.1.16	Yeast	10329452
glutamine---fructose-6-phosphate transaminase (isomerizing)	2.6.1.16	Yeast	10865863
glutamine---fructose-6-phosphate transaminase (isomerizing)	2.6.1.16	Yeast	11270676
glutamine---fructose-6-phosphate transaminase (isomerizing)	2.6.1.16	Yeast	11895440
glutamine---fructose-6-phosphate transaminase (isomerizing)	2.6.1.16	Yeast	15158264
glutamine---fructose-6-phosphate transaminase (isomerizing)	2.6.1.16	Yeast	15308130
glutamine---fructose-6-phosphate transaminase (isomerizing)	2.6.1.16	Yeast	15613679
glutamine---fructose-6-phosphate transaminase (isomerizing)	2.6.1.16	Yeast	17941647
glutamine---fructose-6-phosphate transaminase (isomerizing)	2.6.1.16	Yeast	6184359
glutamine---fructose-6-phosphate transaminase (isomerizing)	2.6.1.16	Yeast	8394312
glutamine---fructose-6-phosphate transaminase (isomerizing)	2.6.1.16	Yeast	9421478
hexokinase	2.7.1.1	Yeast	11319725

hexokinase	2.7.1.1	Yeast	11391834
hexokinase	2.7.1.1	Yeast	11828256
hexokinase	2.7.1.1	Yeast	12660493
hexokinase	2.7.1.1	Yeast	1331693
hexokinase	2.7.1.1	Yeast	14672622
hexokinase	2.7.1.1	Yeast	15607940
hexokinase	2.7.1.1	Yeast	6341787
hexokinase	2.7.1.1	Yeast	6363888
hexokinase	2.7.1.1	Yeast	6440018
hexokinase	2.7.1.1	Yeast	6993859
hexokinase	2.7.1.1	Yeast	8027295
hexokinase	2.7.1.1	Yeast	9523722
dolichol kinase	2.7.1.108	Yeast	3023132
6-phosphofructokinase	2.7.1.11	Yeast	10323269
6-phosphofructokinase	2.7.1.11	Yeast	10444344
6-phosphofructokinase	2.7.1.11	Yeast	10742704
6-phosphofructokinase	2.7.1.11	Yeast	10909961
6-phosphofructokinase	2.7.1.11	Yeast	10931197
6-phosphofructokinase	2.7.1.11	Yeast	1100622
6-phosphofructokinase	2.7.1.11	Yeast	1100623
6-phosphofructokinase	2.7.1.11	Yeast	11014908
6-phosphofructokinase	2.7.1.11	Yeast	11045948
6-phosphofructokinase	2.7.1.11	Yeast	11058792
6-phosphofructokinase	2.7.1.11	Yeast	11391835
6-phosphofructokinase	2.7.1.11	Yeast	11391836
6-phosphofructokinase	2.7.1.11	Yeast	11391837
6-phosphofructokinase	2.7.1.11	Yeast	11560513
6-phosphofructokinase	2.7.1.11	Yeast	12023862
6-phosphofructokinase	2.7.1.11	Yeast	12051897
6-phosphofructokinase	2.7.1.11	Yeast	12125051
6-phosphofructokinase	2.7.1.11	Yeast	12453221
6-phosphofructokinase	2.7.1.11	Yeast	131232
6-phosphofructokinase	2.7.1.11	Yeast	14585511
6-phosphofructokinase	2.7.1.11	Yeast	147929
6-phosphofructokinase	2.7.1.11	Yeast	149128
6-phosphofructokinase	2.7.1.11	Yeast	15157773
6-phosphofructokinase	2.7.1.11	Yeast	15466668
6-phosphofructokinase	2.7.1.11	Yeast	15504384
6-phosphofructokinase	2.7.1.11	Yeast	156307
6-phosphofructokinase	2.7.1.11	Yeast	15991998
6-phosphofructokinase	2.7.1.11	Yeast	16088331
6-phosphofructokinase	2.7.1.11	Yeast	16103521
6-phosphofructokinase	2.7.1.11	Yeast	16115917

6-phosphofructokinase	2.7.1.11	Yeast	16346876
6-phosphofructokinase	2.7.1.11	Yeast	16377227
6-phosphofructokinase	2.7.1.11	Yeast	1658253
6-phosphofructokinase	2.7.1.11	Yeast	16593209
6-phosphofructokinase	2.7.1.11	Yeast	1825156
6-phosphofructokinase	2.7.1.11	Yeast	1828673
6-phosphofructokinase	2.7.1.11	Yeast	1830744
6-phosphofructokinase	2.7.1.11	Yeast	1833303
6-phosphofructokinase	2.7.1.11	Yeast	191426
6-phosphofructokinase	2.7.1.11	Yeast	2137204
6-phosphofructokinase	2.7.1.11	Yeast	2147292
6-phosphofructokinase	2.7.1.11	Yeast	2149746
6-phosphofructokinase	2.7.1.11	Yeast	2434517
6-phosphofructokinase	2.7.1.11	Yeast	2502581
6-phosphofructokinase	2.7.1.11	Yeast	2522395
6-phosphofructokinase	2.7.1.11	Yeast	2527305
6-phosphofructokinase	2.7.1.11	Yeast	2820531
6-phosphofructokinase	2.7.1.11	Yeast	28629
6-phosphofructokinase	2.7.1.11	Yeast	2933146
6-phosphofructokinase	2.7.1.11	Yeast	2935776
6-phosphofructokinase	2.7.1.11	Yeast	2938549
6-phosphofructokinase	2.7.1.11	Yeast	2956156
6-phosphofructokinase	2.7.1.11	Yeast	2963653
6-phosphofructokinase	2.7.1.11	Yeast	2972577
6-phosphofructokinase	2.7.1.11	Yeast	2981949
6-phosphofructokinase	2.7.1.11	Yeast	3364152
6-phosphofructokinase	2.7.1.11	Yeast	3407760
6-phosphofructokinase	2.7.1.11	Yeast	356174
6-phosphofructokinase	2.7.1.11	Yeast	3768440
6-phosphofructokinase	2.7.1.11	Yeast	3931461
6-phosphofructokinase	2.7.1.11	Yeast	4030556
6-phosphofructokinase	2.7.1.11	Yeast	4243437
6-phosphofructokinase	2.7.1.11	Yeast	4252961
6-phosphofructokinase	2.7.1.11	Yeast	6093562
6-phosphofructokinase	2.7.1.11	Yeast	6133774
6-phosphofructokinase	2.7.1.11	Yeast	6211175
6-phosphofructokinase	2.7.1.11	Yeast	6231923
6-phosphofructokinase	2.7.1.11	Yeast	6232272
6-phosphofructokinase	2.7.1.11	Yeast	6234885
6-phosphofructokinase	2.7.1.11	Yeast	6279392
6-phosphofructokinase	2.7.1.11	Yeast	6325266
6-phosphofructokinase	2.7.1.11	Yeast	6331422
6-phosphofructokinase	2.7.1.11	Yeast	6440018

6-phosphofructokinase	2.7.1.11	Yeast	6444231
6-phosphofructokinase	2.7.1.11	Yeast	6452426
6-phosphofructokinase	2.7.1.11	Yeast	6779470
6-phosphofructokinase	2.7.1.11	Yeast	7233512
6-phosphofructokinase	2.7.1.11	Yeast	7440254
6-phosphofructokinase	2.7.1.11	Yeast	7522206
6-phosphofructokinase	2.7.1.11	Yeast	7589825
6-phosphofructokinase	2.7.1.11	Yeast	7602786
6-phosphofructokinase	2.7.1.11	Yeast	7710770
6-phosphofructokinase	2.7.1.11	Yeast	7875554
6-phosphofructokinase	2.7.1.11	Yeast	8224738
6-phosphofructokinase	2.7.1.11	Yeast	8366430
6-phosphofructokinase	2.7.1.11	Yeast	8514849
6-phosphofructokinase	2.7.1.11	Yeast	8557664
6-phosphofructokinase	2.7.1.11	Yeast	8593533
6-phosphofructokinase	2.7.1.11	Yeast	8643924
6-phosphofructokinase	2.7.1.11	Yeast	8910548
6-phosphofructokinase	2.7.1.11	Yeast	8981075
6-phosphofructokinase	2.7.1.11	Yeast	9267516
6-phosphofructokinase	2.7.1.11	Yeast	9287040
6-phosphofructokinase	2.7.1.11	Yeast	9329694
6-phosphofructokinase	2.7.1.11	Yeast	9371084
6-phosphofructokinase	2.7.1.11	Yeast	9439886
6-phosphofructokinase	2.7.1.11	Yeast	9447322
6-phosphofructokinase	2.7.1.11	Yeast	9555897
6-phosphofructokinase	2.7.1.11	Yeast	9580251
6-phosphofructokinase	2.7.1.11	Yeast	9580875
6-phosphofructokinase	2.7.1.11	Yeast	9608547
6-phosphofructokinase	2.7.1.11	Yeast	9766212
6-phosphofructokinase	2.7.1.11	Yeast	9777012
6-phosphofructokinase	2.7.1.11	Yeast	9851886
6-phosphofructokinase	2.7.1.11	Yeast	9973548
phosphatidylinositol 3-kinase	2.7.1.137	Yeast	10869418
phosphatidylinositol 3-kinase	2.7.1.137	Yeast	10874027
phosphatidylinositol 3-kinase	2.7.1.137	Yeast	10998146
phosphatidylinositol 3-kinase	2.7.1.137	Yeast	11259761
phosphatidylinositol 3-kinase	2.7.1.137	Yeast	11744698
phosphatidylinositol 3-kinase	2.7.1.137	Yeast	7669049
phosphatidylinositol 3-kinase	2.7.1.137	Yeast	9292730
phosphatidylinositol 3-kinase	2.7.1.137	Yeast	9312149
phosphatidylinositol 3-kinase	2.7.1.137	Yeast	9478990
phosphatidylinositol 3-kinase	2.7.1.137	Yeast	9826526
phosphatidylinositol 3-kinase	2.7.1.137	Yeast	9826674

xylulokinase	2.7.1.17	Yeast	10981687
xylulokinase	2.7.1.17	Yeast	15727825
glucokinase	2.7.1.2	Yeast	10456334
glucokinase	2.7.1.2	Yeast	10494657
glucokinase	2.7.1.2	Yeast	10905475
glucokinase	2.7.1.2	Yeast	11311143
glucokinase	2.7.1.2	Yeast	11947549
glucokinase	2.7.1.2	Yeast	11950391
glucokinase	2.7.1.2	Yeast	12941786
glucokinase	2.7.1.2	Yeast	14979565
glucokinase	2.7.1.2	Yeast	15009676
glucokinase	2.7.1.2	Yeast	15016359
glucokinase	2.7.1.2	Yeast	15134337
glucokinase	2.7.1.2	Yeast	15226592
glucokinase	2.7.1.2	Yeast	15277402
glucokinase	2.7.1.2	Yeast	1545870
glucokinase	2.7.1.2	Yeast	15707679
glucokinase	2.7.1.2	Yeast	15955369
glucokinase	2.7.1.2	Yeast	16186394
glucokinase	2.7.1.2	Yeast	16834571
glucokinase	2.7.1.2	Yeast	16899262
glucokinase	2.7.1.2	Yeast	16916947
glucokinase	2.7.1.2	Yeast	2210070
glucokinase	2.7.1.2	Yeast	2584235
glucokinase	2.7.1.2	Yeast	2682629
glucokinase	2.7.1.2	Yeast	6780351
glucokinase	2.7.1.2	Yeast	6836273
glucokinase	2.7.1.2	Yeast	7010073
glucokinase	2.7.1.2	Yeast	7553875
glucokinase	2.7.1.2	Yeast	7821741
glucokinase	2.7.1.2	Yeast	7983782
glucokinase	2.7.1.2	Yeast	8194664
glucokinase	2.7.1.2	Yeast	8344416
glucokinase	2.7.1.2	Yeast	8433729
glucokinase	2.7.1.2	Yeast	8446591
glucokinase	2.7.1.2	Yeast	8549869
glucokinase	2.7.1.2	Yeast	8550593
glucokinase	2.7.1.2	Yeast	8631975
glucokinase	2.7.1.2	Yeast	8690154
glucokinase	2.7.1.2	Yeast	8692940
glucokinase	2.7.1.2	Yeast	8751724
glucokinase	2.7.1.2	Yeast	9113996
glucokinase	2.7.1.2	Yeast	9460079

adenosine kinase	2.7.1.20	Yeast	10794412
adenosine kinase	2.7.1.20	Yeast	12228764
adenosine kinase	2.7.1.20	Yeast	15632276
adenosine kinase	2.7.1.20	Yeast	2154328
adenosine kinase	2.7.1.20	Yeast	8184939
riboflavin kinase	2.7.1.26	Yeast	6138398
glycerol kinase	2.7.1.30	Yeast	10.1002/anie.198810401
glycerol kinase	2.7.1.30	Yeast	11388799
glycerol kinase	2.7.1.30	Yeast	11811537
glycerol kinase	2.7.1.30	Yeast	1985967
glycerol kinase	2.7.1.30	Yeast	2547969
glycerol kinase	2.7.1.30	Yeast	4914079
glycerol kinase	2.7.1.30	Yeast	6292169
glycerol kinase	2.7.1.30	Yeast	6440018
glycerol kinase	2.7.1.30	Yeast	9162046
choline kinase	2.7.1.32	Yeast	1336121
choline kinase	2.7.1.32	Yeast	16490392
choline kinase	2.7.1.32	Yeast	2153442
choline kinase	2.7.1.32	Yeast	217369
choline kinase	2.7.1.32	Yeast	3365445
choline kinase	2.7.1.32	Yeast	3447597
choline kinase	2.7.1.32	Yeast	5495730
choline kinase	2.7.1.32	Yeast	6503617
choline kinase	2.7.1.32	Yeast	8182083
choline kinase	2.7.1.32	Yeast	828054
choline kinase	2.7.1.32	Yeast	8414498
pantothenate kinase	2.7.1.33	Yeast	10625688
pantothenate kinase	2.7.1.33	Yeast	11809413
pantothenate kinase	2.7.1.33	Yeast	12697433
pantothenate kinase	2.7.1.33	Yeast	15176870
pantothenate kinase	2.7.1.33	Yeast	15843025
pantothenate kinase	2.7.1.33	Yeast	16701556
pantothenate kinase	2.7.1.33	Yeast	17323930
pantothenate kinase	2.7.1.33	Yeast	17581817
pantothenate kinase	2.7.1.33	Yeast	9890959
pyruvate kinase	2.7.1.40	Yeast	101523
pyruvate kinase	2.7.1.40	Yeast	11181519
pyruvate kinase	2.7.1.40	Yeast	1175605
pyruvate kinase	2.7.1.40	Yeast	1328007
pyruvate kinase	2.7.1.40	Yeast	1406667
pyruvate kinase	2.7.1.40	Yeast	15028426
pyruvate kinase	2.7.1.40	Yeast	15567985
pyruvate kinase	2.7.1.40	Yeast	16046853

pyruvate kinase	2.7.1.40	Yeast	16511150
pyruvate kinase	2.7.1.40	Yeast	16549526
pyruvate kinase	2.7.1.40	Yeast	1959479
pyruvate kinase	2.7.1.40	Yeast	2387024
pyruvate kinase	2.7.1.40	Yeast	2813362
pyruvate kinase	2.7.1.40	Yeast	2820531
pyruvate kinase	2.7.1.40	Yeast	2846196
pyruvate kinase	2.7.1.40	Yeast	291050
pyruvate kinase	2.7.1.40	Yeast	29278
pyruvate kinase	2.7.1.40	Yeast	2935776
pyruvate kinase	2.7.1.40	Yeast	2970638
pyruvate kinase	2.7.1.40	Yeast	3023262
pyruvate kinase	2.7.1.40	Yeast	3032541
pyruvate kinase	2.7.1.40	Yeast	3159473
pyruvate kinase	2.7.1.40	Yeast	3161219
pyruvate kinase	2.7.1.40	Yeast	3350145
pyruvate kinase	2.7.1.40	Yeast	4053567
pyruvate kinase	2.7.1.40	Yeast	6222515
pyruvate kinase	2.7.1.40	Yeast	6241274
pyruvate kinase	2.7.1.40	Yeast	6268138
pyruvate kinase	2.7.1.40	Yeast	6370232
pyruvate kinase	2.7.1.40	Yeast	6588273
pyruvate kinase	2.7.1.40	Yeast	6682991
pyruvate kinase	2.7.1.40	Yeast	6713301
pyruvate kinase	2.7.1.40	Yeast	7357032
pyruvate kinase	2.7.1.40	Yeast	7961441
pyruvate kinase	2.7.1.40	Yeast	8074527
pyruvate kinase	2.7.1.40	Yeast	8144600
pyruvate kinase	2.7.1.40	Yeast	8436141
pyruvate kinase	2.7.1.40	Yeast	8476115
pyruvate kinase	2.7.1.40	Yeast	8765986
pyruvate kinase	2.7.1.40	Yeast	9252361
uridine kinase	2.7.1.48	Yeast	15735337
uridine kinase	2.7.1.48	Yeast	195585
uridine kinase	2.7.1.48	Yeast	9923963
galactokinase	2.7.1.6	Yeast	12694189
galactokinase	2.7.1.6	Yeast	14596685
galactokinase	2.7.1.6	Yeast	14763977
galactokinase	2.7.1.6	Yeast	16452467
galactokinase	2.7.1.6	Yeast	6836273
1-phosphatidylinositol 4-kinase	2.7.1.67	Yeast	12594831
1-phosphatidylinositol 4-kinase	2.7.1.67	Yeast	12620118
1-phosphatidylinositol 4-kinase	2.7.1.67	Yeast	16912074

1-phosphatidylinositol 4-kinase	2.7.1.67	Yeast	17003043
1-phosphatidylinositol 4-kinase	2.7.1.67	Yeast	7961848
1-phosphatidylinositol 4-kinase	2.7.1.67	Yeast	8152413
1-phosphatidylinositol 4-kinase	2.7.1.67	Yeast	8190262
1-phosphatidylinositol 4-kinase	2.7.1.67	Yeast	9654085
1-phosphatidylinositol 4-kinase	2.7.1.67	Yeast	9854149
1-phosphatidylinositol 4-kinase	2.7.1.67	Yeast	9891985
1-phosphatidylinositol-4-phosphate 5-kinase	2.7.1.68	Yeast	11098053
1-phosphatidylinositol-4-phosphate 5-kinase	2.7.1.68	Yeast	12620118
1-phosphatidylinositol-4-phosphate 5-kinase	2.7.1.68	Yeast	15277528
1-phosphatidylinositol-4-phosphate 5-kinase	2.7.1.68	Yeast	15738269
1-phosphatidylinositol-4-phosphate 5-kinase	2.7.1.68	Yeast	17635937
1-phosphatidylinositol-4-phosphate 5-kinase	2.7.1.68	Yeast	2849321
1-phosphatidylinositol-4-phosphate 5-kinase	2.7.1.68	Yeast	8190262
1-phosphatidylinositol-4-phosphate 5-kinase	2.7.1.68	Yeast	9292730
ethanolamine kinase	2.7.1.82	Yeast	11044454
ethanolamine kinase	2.7.1.82	Yeast	1480155
ethanolamine kinase	2.7.1.82	Yeast	475777
ethanolamine kinase	2.7.1.82	Yeast	7142139
sphinganine kinase	2.7.1.91	Yeast	10567432
sphinganine kinase	2.7.1.91	Yeast	14568343
sphinganine kinase	2.7.1.91	Yeast	15451787
sphinganine kinase	2.7.1.91	Yeast	16831409
glutamate 5-kinase	2.7.2.11	Yeast	12602867
glutamate 5-kinase	2.7.2.11	Yeast	15077666
glutamate 5-kinase	2.7.2.11	Yeast	17449694
aspartate kinase	2.7.2.4	Yeast	16232634
aspartate kinase	2.7.2.4	Yeast	16232840
aspartate kinase	2.7.2.4	Yeast	16658323
aspartate kinase	2.7.2.4	Yeast	16666968
aspartate kinase	2.7.2.4	Yeast	17309616
aspartate kinase	2.7.2.4	Yeast	17350037
aspartate kinase	2.7.2.4	Yeast	17611754
aspartate kinase	2.7.2.4	Yeast	3081884
aspartate kinase	2.7.2.4	Yeast	8299165
adenylate kinase	2.7.4.3	Yeast	126626
adenylate kinase	2.7.4.3	Yeast	14656997
adenylate kinase	2.7.4.3	Yeast	15941717
adenylate kinase	2.7.4.3	Yeast	16668787
adenylate kinase	2.7.4.3	Yeast	6440018
adenylate kinase	2.7.4.3	Yeast	7764491
dTMP kinase	2.7.4.9	Yeast	164949
ribose-phosphate diphosphokinase	2.7.6.1	Yeast	15878857

ribose-phosphate diphosphokinase	2.7.6.1	Yeast	217337
nicotinamide-nucleotide adenylyltransferase	2.7.7.1	Yeast	11248244
nicotinamide-nucleotide adenylyltransferase	2.7.7.1	Yeast	11966442
nicotinamide-nucleotide adenylyltransferase	2.7.7.1	Yeast	17360427
ethanolamine-phosphate cytidylyltransferase	2.7.7.14	Yeast	10493918
ethanolamine-phosphate cytidylyltransferase	2.7.7.14	Yeast	10752579
ethanolamine-phosphate cytidylyltransferase	2.7.7.14	Yeast	11097182
ethanolamine-phosphate cytidylyltransferase	2.7.7.14	Yeast	11844611
ethanolamine-phosphate cytidylyltransferase	2.7.7.14	Yeast	14697519
ethanolamine-phosphate cytidylyltransferase	2.7.7.14	Yeast	15147238
ethanolamine-phosphate cytidylyltransferase	2.7.7.14	Yeast	475777
ethanolamine-phosphate cytidylyltransferase	2.7.7.14	Yeast	603639
ethanolamine-phosphate cytidylyltransferase	2.7.7.14	Yeast	6626563
choline-phosphate cytidylyltransferase	2.7.7.15	Yeast	10101264
choline-phosphate cytidylyltransferase	2.7.7.15	Yeast	10208837
choline-phosphate cytidylyltransferase	2.7.7.15	Yeast	10473578
choline-phosphate cytidylyltransferase	2.7.7.15	Yeast	10615073
choline-phosphate cytidylyltransferase	2.7.7.15	Yeast	10908674
choline-phosphate cytidylyltransferase	2.7.7.15	Yeast	10946015
choline-phosphate cytidylyltransferase	2.7.7.15	Yeast	11029581
choline-phosphate cytidylyltransferase	2.7.7.15	Yeast	11097182
choline-phosphate cytidylyltransferase	2.7.7.15	Yeast	11279002
choline-phosphate cytidylyltransferase	2.7.7.15	Yeast	11404252
choline-phosphate cytidylyltransferase	2.7.7.15	Yeast	11404253
choline-phosphate cytidylyltransferase	2.7.7.15	Yeast	11521967
choline-phosphate cytidylyltransferase	2.7.7.15	Yeast	11829742
choline-phosphate cytidylyltransferase	2.7.7.15	Yeast	12034570
choline-phosphate cytidylyltransferase	2.7.7.15	Yeast	12052891
choline-phosphate cytidylyltransferase	2.7.7.15	Yeast	12062780
choline-phosphate cytidylyltransferase	2.7.7.15	Yeast	12221122
choline-phosphate cytidylyltransferase	2.7.7.15	Yeast	12271462
choline-phosphate cytidylyltransferase	2.7.7.15	Yeast	12370080
choline-phosphate cytidylyltransferase	2.7.7.15	Yeast	12620118
choline-phosphate cytidylyltransferase	2.7.7.15	Yeast	12659631
choline-phosphate cytidylyltransferase	2.7.7.15	Yeast	12842190
choline-phosphate cytidylyltransferase	2.7.7.15	Yeast	12928431
choline-phosphate cytidylyltransferase	2.7.7.15	Yeast	14536058
choline-phosphate cytidylyltransferase	2.7.7.15	Yeast	15079868
choline-phosphate cytidylyltransferase	2.7.7.15	Yeast	15139854
choline-phosphate cytidylyltransferase	2.7.7.15	Yeast	15210848
choline-phosphate cytidylyltransferase	2.7.7.15	Yeast	15522825
choline-phosphate cytidylyltransferase	2.7.7.15	Yeast	15574675
choline-phosphate cytidylyltransferase	2.7.7.15	Yeast	15635091

choline-phosphate cytidylyltransferase	2.7.7.15	Yeast	15788406
choline-phosphate cytidylyltransferase	2.7.7.15	Yeast	15982005
choline-phosphate cytidylyltransferase	2.7.7.15	Yeast	16097951
choline-phosphate cytidylyltransferase	2.7.7.15	Yeast	16153613
choline-phosphate cytidylyltransferase	2.7.7.15	Yeast	16236026
choline-phosphate cytidylyltransferase	2.7.7.15	Yeast	16511521
choline-phosphate cytidylyltransferase	2.7.7.15	Yeast	16580250
choline-phosphate cytidylyltransferase	2.7.7.15	Yeast	16580875
choline-phosphate cytidylyltransferase	2.7.7.15	Yeast	2160812
choline-phosphate cytidylyltransferase	2.7.7.15	Yeast	2268410
choline-phosphate cytidylyltransferase	2.7.7.15	Yeast	2665794
choline-phosphate cytidylyltransferase	2.7.7.15	Yeast	2833508
choline-phosphate cytidylyltransferase	2.7.7.15	Yeast	2838058
choline-phosphate cytidylyltransferase	2.7.7.15	Yeast	3004590
choline-phosphate cytidylyltransferase	2.7.7.15	Yeast	3365445
choline-phosphate cytidylyltransferase	2.7.7.15	Yeast	3367156
choline-phosphate cytidylyltransferase	2.7.7.15	Yeast	3447597
choline-phosphate cytidylyltransferase	2.7.7.15	Yeast	603639
choline-phosphate cytidylyltransferase	2.7.7.15	Yeast	6134645
choline-phosphate cytidylyltransferase	2.7.7.15	Yeast	6243289
choline-phosphate cytidylyltransferase	2.7.7.15	Yeast	6477961
choline-phosphate cytidylyltransferase	2.7.7.15	Yeast	7126613
choline-phosphate cytidylyltransferase	2.7.7.15	Yeast	7487944
choline-phosphate cytidylyltransferase	2.7.7.15	Yeast	7588775
choline-phosphate cytidylyltransferase	2.7.7.15	Yeast	7637558
choline-phosphate cytidylyltransferase	2.7.7.15	Yeast	7768909
choline-phosphate cytidylyltransferase	2.7.7.15	Yeast	7782919
choline-phosphate cytidylyltransferase	2.7.7.15	Yeast	7836412
choline-phosphate cytidylyltransferase	2.7.7.15	Yeast	8006517
choline-phosphate cytidylyltransferase	2.7.7.15	Yeast	8182083
choline-phosphate cytidylyltransferase	2.7.7.15	Yeast	8239319
choline-phosphate cytidylyltransferase	2.7.7.15	Yeast	8255685
choline-phosphate cytidylyltransferase	2.7.7.15	Yeast	8387510
choline-phosphate cytidylyltransferase	2.7.7.15	Yeast	8504126
choline-phosphate cytidylyltransferase	2.7.7.15	Yeast	8597584
choline-phosphate cytidylyltransferase	2.7.7.15	Yeast	8626633
choline-phosphate cytidylyltransferase	2.7.7.15	Yeast	8663247
choline-phosphate cytidylyltransferase	2.7.7.15	Yeast	8756587
choline-phosphate cytidylyltransferase	2.7.7.15	Yeast	8761490
choline-phosphate cytidylyltransferase	2.7.7.15	Yeast	8810902
choline-phosphate cytidylyltransferase	2.7.7.15	Yeast	9009253
choline-phosphate cytidylyltransferase	2.7.7.15	Yeast	9046356
choline-phosphate cytidylyltransferase	2.7.7.15	Yeast	9148929

choline-phosphate cytidylyltransferase	2.7.7.15	Yeast	9335949
choline-phosphate cytidylyltransferase	2.7.7.15	Yeast	9370319
choline-phosphate cytidylyltransferase	2.7.7.15	Yeast	9421188
choline-phosphate cytidylyltransferase	2.7.7.15	Yeast	9714757
sulfate adenylyltransferase	2.7.7.4	Yeast	10759525
sulfate adenylyltransferase	2.7.7.4	Yeast	10956658
sulfate adenylyltransferase	2.7.7.4	Yeast	11157739
sulfate adenylyltransferase	2.7.7.4	Yeast	14613928
sulfate adenylyltransferase	2.7.7.4	Yeast	16008502
sulfate adenylyltransferase	2.7.7.4	Yeast	17095009
sulfate adenylyltransferase	2.7.7.4	Yeast	8206850
sulfate adenylyltransferase	2.7.7.4	Yeast	9880353
phosphatidate cytidylyltransferase	2.7.7.41	Yeast	11985865
phosphatidate cytidylyltransferase	2.7.7.41	Yeast	9345289
UTP---glucose-1-phosphate uridylyltransferase	2.7.7.9	Yeast	11171080
UTP---glucose-1-phosphate uridylyltransferase	2.7.7.9	Yeast	1149741
UTP---glucose-1-phosphate uridylyltransferase	2.7.7.9	Yeast	12088504
UTP---glucose-1-phosphate uridylyltransferase	2.7.7.9	Yeast	15274139
biotin synthase	2.8.1.6	Yeast	16195795
triacylglycerol lipase	3.1.1.3	Yeast	11217140
triacylglycerol lipase	3.1.1.3	Yeast	12689525
triacylglycerol lipase	3.1.1.3	Yeast	3678753
alkaline phosphatase	3.1.3.1	Yeast	11029583
alkaline phosphatase	3.1.3.1	Yeast	11139445
alkaline phosphatase	3.1.3.1	Yeast	12412807
alkaline phosphatase	3.1.3.1	Yeast	1445337
alkaline phosphatase	3.1.3.1	Yeast	14560000
alkaline phosphatase	3.1.3.1	Yeast	3509742
alkaline phosphatase	3.1.3.1	Yeast	7032602
fructose-bisphosphatase	3.1.3.11	Yeast	10327613
fructose-bisphosphatase	3.1.3.11	Yeast	10393302
fructose-bisphosphatase	3.1.3.11	Yeast	10773464
fructose-bisphosphatase	3.1.3.11	Yeast	11536627
fructose-bisphosphatase	3.1.3.11	Yeast	11864619
fructose-bisphosphatase	3.1.3.11	Yeast	12190028
fructose-bisphosphatase	3.1.3.11	Yeast	12686616
fructose-bisphosphatase	3.1.3.11	Yeast	15225753
fructose-bisphosphatase	3.1.3.11	Yeast	15498578
fructose-bisphosphatase	3.1.3.11	Yeast	15631980

fructose-bisphosphatase	3.1.3.11	Yeast	16199065
fructose-bisphosphatase	3.1.3.11	Yeast	16580859
fructose-bisphosphatase	3.1.3.11	Yeast	16593209
fructose-bisphosphatase	3.1.3.11	Yeast	16657971
fructose-bisphosphatase	3.1.3.11	Yeast	16814784
fructose-bisphosphatase	3.1.3.11	Yeast	16857246
fructose-bisphosphatase	3.1.3.11	Yeast	2835013
fructose-bisphosphatase	3.1.3.11	Yeast	2983680
fructose-bisphosphatase	3.1.3.11	Yeast	3068502
fructose-bisphosphatase	3.1.3.11	Yeast	3291467
fructose-bisphosphatase	3.1.3.11	Yeast	4342496
fructose-bisphosphatase	3.1.3.11	Yeast	7532742
fructose-bisphosphatase	3.1.3.11	Yeast	7552262
fructose-bisphosphatase	3.1.3.11	Yeast	7558035
fructose-bisphosphatase	3.1.3.11	Yeast	7579072
fructose-bisphosphatase	3.1.3.11	Yeast	7592860
fructose-bisphosphatase	3.1.3.11	Yeast	8043646
acid phosphatase	3.1.3.2	Yeast	10471332
acid phosphatase	3.1.3.2	Yeast	10646119
acid phosphatase	3.1.3.2	Yeast	1364364
acid phosphatase	3.1.3.2	Yeast	2153810
acid phosphatase	3.1.3.2	Yeast	2545859
acid phosphatase	3.1.3.2	Yeast	2713876
acid phosphatase	3.1.3.2	Yeast	9370316
phosphoserine phosphatase	3.1.3.3	Yeast	8858931
alpha-glucosidase	3.2.1.20	Yeast	10619707
alpha-glucosidase	3.2.1.20	Yeast	10810293
alpha-glucosidase	3.2.1.20	Yeast	11134937
alpha-glucosidase	3.2.1.20	Yeast	11230125
alpha-glucosidase	3.2.1.20	Yeast	11404235
alpha-glucosidase	3.2.1.20	Yeast	16233203
alpha-glucosidase	3.2.1.20	Yeast	16233375
alpha-glucosidase	3.2.1.20	Yeast	7626594
alpha-glucosidase	3.2.1.20	Yeast	8625892
alpha-mannosidase	3.2.1.24	Yeast	11406577
alpha-mannosidase	3.2.1.24	Yeast	16460512
alpha-mannosidase	3.2.1.24	Yeast	16806128
alpha-mannosidase	3.2.1.24	Yeast	16899540
alpha-mannosidase	3.2.1.24	Yeast	2466460
alpha-mannosidase	3.2.1.24	Yeast	2843530
alpha-mannosidase	3.2.1.24	Yeast	4092864
alpha-mannosidase	3.2.1.24	Yeast	9022667
alpha-mannosidase	3.2.1.24	Yeast	9230311

leukotriene-A4 hydrolase	3.3.2.6	Yeast	10691697
leukotriene-A4 hydrolase	3.3.2.6	Yeast	11805219
leukotriene-A4 hydrolase	3.3.2.6	Yeast	12139459
leukotriene-A4 hydrolase	3.3.2.6	Yeast	12865451
leukotriene-A4 hydrolase	3.3.2.6	Yeast	1311589
leukotriene-A4 hydrolase	3.3.2.6	Yeast	6329309
leukotriene-A4 hydrolase	3.3.2.6	Yeast	7732829
leukotriene-A4 hydrolase	3.3.2.6	Yeast	9395533
leukotriene-A4 hydrolase	3.3.2.6	Yeast	9413890
dihydroorotase	3.5.2.3	Yeast	3345746
dihydroorotase	3.5.2.3	Yeast	4092695
dihydroorotase	3.5.2.3	Yeast	42399
dihydroorotase	3.5.2.3	Yeast	6115855
GTP cyclohydrolase I	3.5.4.16	Yeast	12392559
GTP cyclohydrolase I	3.5.4.16	Yeast	12855421
GTP cyclohydrolase I	3.5.4.16	Yeast	1459137
GTP cyclohydrolase I	3.5.4.16	Yeast	15044686
GTP cyclohydrolase I	3.5.4.16	Yeast	16179591
GTP cyclohydrolase I	3.5.4.16	Yeast	16636057
GTP cyclohydrolase I	3.5.4.16	Yeast	737222
GTP cyclohydrolase I	3.5.4.16	Yeast	7521513
GTP cyclohydrolase I	3.5.4.16	Yeast	9182249
GTP cyclohydrolase I	3.5.4.16	Yeast	9444617
H+-transporting two-sector ATPase	3.6.3.14	Yeast	11744700
H+-transporting two-sector ATPase	3.6.3.14	Yeast	11893513
H+-transporting two-sector ATPase	3.6.3.14	Yeast	12587531
H+-transporting two-sector ATPase	3.6.3.14	Yeast	15712234
H+-transporting two-sector ATPase	3.6.3.14	Yeast	16510118
H+-transporting two-sector ATPase	3.6.3.14	Yeast	16730639
H+-transporting two-sector ATPase	3.6.3.14	Yeast	2532597
H+-transporting two-sector ATPase	3.6.3.14	Yeast	2889730
H+-transporting two-sector ATPase	3.6.3.14	Yeast	6456904
H+-transporting two-sector ATPase	3.6.3.14	Yeast	8516333
glutamate decarboxylase	4.1.1.15	Yeast	10331265
glutamate decarboxylase	4.1.1.15	Yeast	12196588
glutamate decarboxylase	4.1.1.15	Yeast	12746320
glutamate decarboxylase	4.1.1.15	Yeast	15210535
glutamate decarboxylase	4.1.1.15	Yeast	15581395
glutamate decarboxylase	4.1.1.15	Yeast	1697032
glutamate decarboxylase	4.1.1.15	Yeast	17044036
glutamate decarboxylase	4.1.1.15	Yeast	1976015
glutamate decarboxylase	4.1.1.15	Yeast	2180326
glutamate decarboxylase	4.1.1.15	Yeast	2735448

glutamate decarboxylase	4.1.1.15	Yeast	2857768
glutamate decarboxylase	4.1.1.15	Yeast	3896834
glutamate decarboxylase	4.1.1.15	Yeast	6975381
glutamate decarboxylase	4.1.1.15	Yeast	7702443
glutamate decarboxylase	4.1.1.15	Yeast	7885536
glutamate decarboxylase	4.1.1.15	Yeast	8302162
glutamate decarboxylase	4.1.1.15	Yeast	9011754
glutamate decarboxylase	4.1.1.15	Yeast	9053794
ornithine decarboxylase	4.1.1.17	Yeast	10069996
ornithine decarboxylase	4.1.1.17	Yeast	10216947
ornithine decarboxylase	4.1.1.17	Yeast	10319188
ornithine decarboxylase	4.1.1.17	Yeast	10320037
ornithine decarboxylase	4.1.1.17	Yeast	10321508
ornithine decarboxylase	4.1.1.17	Yeast	10430664
ornithine decarboxylase	4.1.1.17	Yeast	10453061
ornithine decarboxylase	4.1.1.17	Yeast	10456943
ornithine decarboxylase	4.1.1.17	Yeast	10473083
ornithine decarboxylase	4.1.1.17	Yeast	10485326
ornithine decarboxylase	4.1.1.17	Yeast	10544213
ornithine decarboxylase	4.1.1.17	Yeast	10550568
ornithine decarboxylase	4.1.1.17	Yeast	10564512
ornithine decarboxylase	4.1.1.17	Yeast	10589756
ornithine decarboxylase	4.1.1.17	Yeast	10593613
ornithine decarboxylase	4.1.1.17	Yeast	10607762
ornithine decarboxylase	4.1.1.17	Yeast	10629084
ornithine decarboxylase	4.1.1.17	Yeast	10712236
ornithine decarboxylase	4.1.1.17	Yeast	10713131
ornithine decarboxylase	4.1.1.17	Yeast	10760944
ornithine decarboxylase	4.1.1.17	Yeast	10772389
ornithine decarboxylase	4.1.1.17	Yeast	10816435
ornithine decarboxylase	4.1.1.17	Yeast	10817834
ornithine decarboxylase	4.1.1.17	Yeast	10882097
ornithine decarboxylase	4.1.1.17	Yeast	10931831
ornithine decarboxylase	4.1.1.17	Yeast	10940513
ornithine decarboxylase	4.1.1.17	Yeast	10965017
ornithine decarboxylase	4.1.1.17	Yeast	11003584
ornithine decarboxylase	4.1.1.17	Yeast	11085920
ornithine decarboxylase	4.1.1.17	Yeast	11095648
ornithine decarboxylase	4.1.1.17	Yeast	11137705
ornithine decarboxylase	4.1.1.17	Yeast	11180396
ornithine decarboxylase	4.1.1.17	Yeast	11235918
ornithine decarboxylase	4.1.1.17	Yeast	11355005
ornithine decarboxylase	4.1.1.17	Yeast	11376395

ornithine decarboxylase	4.1.1.17	Yeast	11408092
ornithine decarboxylase	4.1.1.17	Yeast	11408253
ornithine decarboxylase	4.1.1.17	Yeast	11408542
ornithine decarboxylase	4.1.1.17	Yeast	11413269
ornithine decarboxylase	4.1.1.17	Yeast	11540835
ornithine decarboxylase	4.1.1.17	Yeast	11558274
ornithine decarboxylase	4.1.1.17	Yeast	11736657
ornithine decarboxylase	4.1.1.17	Yeast	11782361
ornithine decarboxylase	4.1.1.17	Yeast	11852055
ornithine decarboxylase	4.1.1.17	Yeast	11883715
ornithine decarboxylase	4.1.1.17	Yeast	11922393
ornithine decarboxylase	4.1.1.17	Yeast	11923270
ornithine decarboxylase	4.1.1.17	Yeast	11964084
ornithine decarboxylase	4.1.1.17	Yeast	11997243
ornithine decarboxylase	4.1.1.17	Yeast	12054570
ornithine decarboxylase	4.1.1.17	Yeast	12105848
ornithine decarboxylase	4.1.1.17	Yeast	12148577
ornithine decarboxylase	4.1.1.17	Yeast	12355213
ornithine decarboxylase	4.1.1.17	Yeast	12452334
ornithine decarboxylase	4.1.1.17	Yeast	12497077
ornithine decarboxylase	4.1.1.17	Yeast	12527115
ornithine decarboxylase	4.1.1.17	Yeast	12663506
ornithine decarboxylase	4.1.1.17	Yeast	12716308
ornithine decarboxylase	4.1.1.17	Yeast	12716758
ornithine decarboxylase	4.1.1.17	Yeast	12766050
ornithine decarboxylase	4.1.1.17	Yeast	12816757
ornithine decarboxylase	4.1.1.17	Yeast	12856719
ornithine decarboxylase	4.1.1.17	Yeast	12882169
ornithine decarboxylase	4.1.1.17	Yeast	1289667
ornithine decarboxylase	4.1.1.17	Yeast	1324153
ornithine decarboxylase	4.1.1.17	Yeast	1360468
ornithine decarboxylase	4.1.1.17	Yeast	1397089
ornithine decarboxylase	4.1.1.17	Yeast	1407701
ornithine decarboxylase	4.1.1.17	Yeast	1409247
ornithine decarboxylase	4.1.1.17	Yeast	1417733
ornithine decarboxylase	4.1.1.17	Yeast	14769544
ornithine decarboxylase	4.1.1.17	Yeast	1495349
ornithine decarboxylase	4.1.1.17	Yeast	15002659
ornithine decarboxylase	4.1.1.17	Yeast	1511780
ornithine decarboxylase	4.1.1.17	Yeast	15120115
ornithine decarboxylase	4.1.1.17	Yeast	15180186
ornithine decarboxylase	4.1.1.17	Yeast	1521915
ornithine decarboxylase	4.1.1.17	Yeast	15223770

ornithine decarboxylase	4.1.1.17	Yeast	15228220
ornithine decarboxylase	4.1.1.17	Yeast	15233741
ornithine decarboxylase	4.1.1.17	Yeast	15247138
ornithine decarboxylase	4.1.1.17	Yeast	15296840
ornithine decarboxylase	4.1.1.17	Yeast	15306645
ornithine decarboxylase	4.1.1.17	Yeast	15355308
ornithine decarboxylase	4.1.1.17	Yeast	15514084
ornithine decarboxylase	4.1.1.17	Yeast	15538383
ornithine decarboxylase	4.1.1.17	Yeast	15539331
ornithine decarboxylase	4.1.1.17	Yeast	1563337
ornithine decarboxylase	4.1.1.17	Yeast	15697240
ornithine decarboxylase	4.1.1.17	Yeast	1569947
ornithine decarboxylase	4.1.1.17	Yeast	15716048
ornithine decarboxylase	4.1.1.17	Yeast	15843384
ornithine decarboxylase	4.1.1.17	Yeast	1584960
ornithine decarboxylase	4.1.1.17	Yeast	1590311
ornithine decarboxylase	4.1.1.17	Yeast	1601800
ornithine decarboxylase	4.1.1.17	Yeast	16091008
ornithine decarboxylase	4.1.1.17	Yeast	16168128
ornithine decarboxylase	4.1.1.17	Yeast	16170669
ornithine decarboxylase	4.1.1.17	Yeast	16181115
ornithine decarboxylase	4.1.1.17	Yeast	16223706
ornithine decarboxylase	4.1.1.17	Yeast	16230862
ornithine decarboxylase	4.1.1.17	Yeast	16290266
ornithine decarboxylase	4.1.1.17	Yeast	16342411
ornithine decarboxylase	4.1.1.17	Yeast	1641775
ornithine decarboxylase	4.1.1.17	Yeast	16445292
ornithine decarboxylase	4.1.1.17	Yeast	1655898
ornithine decarboxylase	4.1.1.17	Yeast	16568078
ornithine decarboxylase	4.1.1.17	Yeast	16630547
ornithine decarboxylase	4.1.1.17	Yeast	16662219
ornithine decarboxylase	4.1.1.17	Yeast	16666570
ornithine decarboxylase	4.1.1.17	Yeast	16678846
ornithine decarboxylase	4.1.1.17	Yeast	16729674
ornithine decarboxylase	4.1.1.17	Yeast	1697882
ornithine decarboxylase	4.1.1.17	Yeast	1703390
ornithine decarboxylase	4.1.1.17	Yeast	1733364
ornithine decarboxylase	4.1.1.17	Yeast	17407445
ornithine decarboxylase	4.1.1.17	Yeast	1745018
ornithine decarboxylase	4.1.1.17	Yeast	1782416
ornithine decarboxylase	4.1.1.17	Yeast	1814556
ornithine decarboxylase	4.1.1.17	Yeast	1814755
ornithine decarboxylase	4.1.1.17	Yeast	1831810

ornithine decarboxylase	4.1.1.17	Yeast	1846091
ornithine decarboxylase	4.1.1.17	Yeast	1878921
ornithine decarboxylase	4.1.1.17	Yeast	1884248
ornithine decarboxylase	4.1.1.17	Yeast	1892753
ornithine decarboxylase	4.1.1.17	Yeast	1900385
ornithine decarboxylase	4.1.1.17	Yeast	1932775
ornithine decarboxylase	4.1.1.17	Yeast	1940203
ornithine decarboxylase	4.1.1.17	Yeast	1962522
ornithine decarboxylase	4.1.1.17	Yeast	196870
ornithine decarboxylase	4.1.1.17	Yeast	1997184
ornithine decarboxylase	4.1.1.17	Yeast	2006469
ornithine decarboxylase	4.1.1.17	Yeast	2009332
ornithine decarboxylase	4.1.1.17	Yeast	2019760
ornithine decarboxylase	4.1.1.17	Yeast	203259
ornithine decarboxylase	4.1.1.17	Yeast	2051775
ornithine decarboxylase	4.1.1.17	Yeast	2088816
ornithine decarboxylase	4.1.1.17	Yeast	2118148
ornithine decarboxylase	4.1.1.17	Yeast	2148056
ornithine decarboxylase	4.1.1.17	Yeast	2160044
ornithine decarboxylase	4.1.1.17	Yeast	2197525
ornithine decarboxylase	4.1.1.17	Yeast	2210666
ornithine decarboxylase	4.1.1.17	Yeast	2243540
ornithine decarboxylase	4.1.1.17	Yeast	2293084
ornithine decarboxylase	4.1.1.17	Yeast	2296762
ornithine decarboxylase	4.1.1.17	Yeast	2298913
ornithine decarboxylase	4.1.1.17	Yeast	2409817
ornithine decarboxylase	4.1.1.17	Yeast	2469492
ornithine decarboxylase	4.1.1.17	Yeast	2472814
ornithine decarboxylase	4.1.1.17	Yeast	2493794
ornithine decarboxylase	4.1.1.17	Yeast	2494779
ornithine decarboxylase	4.1.1.17	Yeast	2497460
ornithine decarboxylase	4.1.1.17	Yeast	2497556
ornithine decarboxylase	4.1.1.17	Yeast	2505399
ornithine decarboxylase	4.1.1.17	Yeast	2505959
ornithine decarboxylase	4.1.1.17	Yeast	2507383
ornithine decarboxylase	4.1.1.17	Yeast	2507471
ornithine decarboxylase	4.1.1.17	Yeast	2525760
ornithine decarboxylase	4.1.1.17	Yeast	2553150
ornithine decarboxylase	4.1.1.17	Yeast	2610929
ornithine decarboxylase	4.1.1.17	Yeast	2651129
ornithine decarboxylase	4.1.1.17	Yeast	2699646
ornithine decarboxylase	4.1.1.17	Yeast	2754510
ornithine decarboxylase	4.1.1.17	Yeast	2775206

ornithine decarboxylase	4.1.1.17	Yeast	2829727
ornithine decarboxylase	4.1.1.17	Yeast	2840461
ornithine decarboxylase	4.1.1.17	Yeast	2915649
ornithine decarboxylase	4.1.1.17	Yeast	2916900
ornithine decarboxylase	4.1.1.17	Yeast	2977154
ornithine decarboxylase	4.1.1.17	Yeast	2979197
ornithine decarboxylase	4.1.1.17	Yeast	2986953
ornithine decarboxylase	4.1.1.17	Yeast	3004707
ornithine decarboxylase	4.1.1.17	Yeast	3009424
ornithine decarboxylase	4.1.1.17	Yeast	3022056
ornithine decarboxylase	4.1.1.17	Yeast	3036091
ornithine decarboxylase	4.1.1.17	Yeast	3037250
ornithine decarboxylase	4.1.1.17	Yeast	3040821
ornithine decarboxylase	4.1.1.17	Yeast	3082276
ornithine decarboxylase	4.1.1.17	Yeast	3084209
ornithine decarboxylase	4.1.1.17	Yeast	3084872
ornithine decarboxylase	4.1.1.17	Yeast	3086160
ornithine decarboxylase	4.1.1.17	Yeast	3092827
ornithine decarboxylase	4.1.1.17	Yeast	3093095
ornithine decarboxylase	4.1.1.17	Yeast	3096557
ornithine decarboxylase	4.1.1.17	Yeast	3100897
ornithine decarboxylase	4.1.1.17	Yeast	3102397
ornithine decarboxylase	4.1.1.17	Yeast	3105968
ornithine decarboxylase	4.1.1.17	Yeast	3106075
ornithine decarboxylase	4.1.1.17	Yeast	3108666
ornithine decarboxylase	4.1.1.17	Yeast	3109979
ornithine decarboxylase	4.1.1.17	Yeast	3109985
ornithine decarboxylase	4.1.1.17	Yeast	3111384
ornithine decarboxylase	4.1.1.17	Yeast	3113732
ornithine decarboxylase	4.1.1.17	Yeast	3117720
ornithine decarboxylase	4.1.1.17	Yeast	3121457
ornithine decarboxylase	4.1.1.17	Yeast	3122042
ornithine decarboxylase	4.1.1.17	Yeast	3129184
ornithine decarboxylase	4.1.1.17	Yeast	3130188
ornithine decarboxylase	4.1.1.17	Yeast	3139441
ornithine decarboxylase	4.1.1.17	Yeast	3141045
ornithine decarboxylase	4.1.1.17	Yeast	3143046
ornithine decarboxylase	4.1.1.17	Yeast	3180091
ornithine decarboxylase	4.1.1.17	Yeast	3279036
ornithine decarboxylase	4.1.1.17	Yeast	3328430
ornithine decarboxylase	4.1.1.17	Yeast	3356404
ornithine decarboxylase	4.1.1.17	Yeast	3403538
ornithine decarboxylase	4.1.1.17	Yeast	3443298

ornithine decarboxylase	4.1.1.17	Yeast	3538740
ornithine decarboxylase	4.1.1.17	Yeast	3548994
ornithine decarboxylase	4.1.1.17	Yeast	3661847
ornithine decarboxylase	4.1.1.17	Yeast	3672608
ornithine decarboxylase	4.1.1.17	Yeast	3688216
ornithine decarboxylase	4.1.1.17	Yeast	3729588
ornithine decarboxylase	4.1.1.17	Yeast	3743773
ornithine decarboxylase	4.1.1.17	Yeast	3753036
ornithine decarboxylase	4.1.1.17	Yeast	3775249
ornithine decarboxylase	4.1.1.17	Yeast	3794781
ornithine decarboxylase	4.1.1.17	Yeast	3857388
ornithine decarboxylase	4.1.1.17	Yeast	3901680
ornithine decarboxylase	4.1.1.17	Yeast	3905315
ornithine decarboxylase	4.1.1.17	Yeast	3921243
ornithine decarboxylase	4.1.1.17	Yeast	3926303
ornithine decarboxylase	4.1.1.17	Yeast	3930649
ornithine decarboxylase	4.1.1.17	Yeast	3931300
ornithine decarboxylase	4.1.1.17	Yeast	3934106
ornithine decarboxylase	4.1.1.17	Yeast	3999751
ornithine decarboxylase	4.1.1.17	Yeast	4029343
ornithine decarboxylase	4.1.1.17	Yeast	4053280
ornithine decarboxylase	4.1.1.17	Yeast	497279
ornithine decarboxylase	4.1.1.17	Yeast	6124275
ornithine decarboxylase	4.1.1.17	Yeast	6178351
ornithine decarboxylase	4.1.1.17	Yeast	6190690
ornithine decarboxylase	4.1.1.17	Yeast	6192925
ornithine decarboxylase	4.1.1.17	Yeast	6223191
ornithine decarboxylase	4.1.1.17	Yeast	6256169
ornithine decarboxylase	4.1.1.17	Yeast	6272311
ornithine decarboxylase	4.1.1.17	Yeast	6307502
ornithine decarboxylase	4.1.1.17	Yeast	6365078
ornithine decarboxylase	4.1.1.17	Yeast	6432312
ornithine decarboxylase	4.1.1.17	Yeast	6432848
ornithine decarboxylase	4.1.1.17	Yeast	6439208
ornithine decarboxylase	4.1.1.17	Yeast	6440787
ornithine decarboxylase	4.1.1.17	Yeast	6445842
ornithine decarboxylase	4.1.1.17	Yeast	6467454
ornithine decarboxylase	4.1.1.17	Yeast	6571411
ornithine decarboxylase	4.1.1.17	Yeast	6591862
ornithine decarboxylase	4.1.1.17	Yeast	6624798
ornithine decarboxylase	4.1.1.17	Yeast	6692409
ornithine decarboxylase	4.1.1.17	Yeast	6696980
ornithine decarboxylase	4.1.1.17	Yeast	6721578

ornithine decarboxylase	4.1.1.17	Yeast	6750139
ornithine decarboxylase	4.1.1.17	Yeast	6812570
ornithine decarboxylase	4.1.1.17	Yeast	6813460
ornithine decarboxylase	4.1.1.17	Yeast	6865777
ornithine decarboxylase	4.1.1.17	Yeast	6891933
ornithine decarboxylase	4.1.1.17	Yeast	7093948
ornithine decarboxylase	4.1.1.17	Yeast	7104206
ornithine decarboxylase	4.1.1.17	Yeast	7159401
ornithine decarboxylase	4.1.1.17	Yeast	7205597
ornithine decarboxylase	4.1.1.17	Yeast	7310281
ornithine decarboxylase	4.1.1.17	Yeast	7381752
ornithine decarboxylase	4.1.1.17	Yeast	7426404
ornithine decarboxylase	4.1.1.17	Yeast	7488168
ornithine decarboxylase	4.1.1.17	Yeast	7498733
ornithine decarboxylase	4.1.1.17	Yeast	7525612
ornithine decarboxylase	4.1.1.17	Yeast	7616440
ornithine decarboxylase	4.1.1.17	Yeast	7628376
ornithine decarboxylase	4.1.1.17	Yeast	7656288
ornithine decarboxylase	4.1.1.17	Yeast	7718766
ornithine decarboxylase	4.1.1.17	Yeast	7813017
ornithine decarboxylase	4.1.1.17	Yeast	7823874
ornithine decarboxylase	4.1.1.17	Yeast	7865470
ornithine decarboxylase	4.1.1.17	Yeast	7872745
ornithine decarboxylase	4.1.1.17	Yeast	7874572
ornithine decarboxylase	4.1.1.17	Yeast	7895420
ornithine decarboxylase	4.1.1.17	Yeast	7929646
ornithine decarboxylase	4.1.1.17	Yeast	7943199
ornithine decarboxylase	4.1.1.17	Yeast	7951165
ornithine decarboxylase	4.1.1.17	Yeast	7965748
ornithine decarboxylase	4.1.1.17	Yeast	7972938
ornithine decarboxylase	4.1.1.17	Yeast	7981636
ornithine decarboxylase	4.1.1.17	Yeast	8010156
ornithine decarboxylase	4.1.1.17	Yeast	8028020
ornithine decarboxylase	4.1.1.17	Yeast	8065308
ornithine decarboxylase	4.1.1.17	Yeast	8095973
ornithine decarboxylase	4.1.1.17	Yeast	8110472
ornithine decarboxylase	4.1.1.17	Yeast	8140036
ornithine decarboxylase	4.1.1.17	Yeast	8141779
ornithine decarboxylase	4.1.1.17	Yeast	8152342
ornithine decarboxylase	4.1.1.17	Yeast	8185631
ornithine decarboxylase	4.1.1.17	Yeast	8190721
ornithine decarboxylase	4.1.1.17	Yeast	8242794
ornithine decarboxylase	4.1.1.17	Yeast	8344985

ornithine decarboxylase	4.1.1.17	Yeast	8368314
ornithine decarboxylase	4.1.1.17	Yeast	8374143
ornithine decarboxylase	4.1.1.17	Yeast	8419528
ornithine decarboxylase	4.1.1.17	Yeast	8447420
ornithine decarboxylase	4.1.1.17	Yeast	8453677
ornithine decarboxylase	4.1.1.17	Yeast	8462726
ornithine decarboxylase	4.1.1.17	Yeast	8465553
ornithine decarboxylase	4.1.1.17	Yeast	8478959
ornithine decarboxylase	4.1.1.17	Yeast	8501729
ornithine decarboxylase	4.1.1.17	Yeast	8538189
ornithine decarboxylase	4.1.1.17	Yeast	8549635
ornithine decarboxylase	4.1.1.17	Yeast	8572176
ornithine decarboxylase	4.1.1.17	Yeast	8660289
ornithine decarboxylase	4.1.1.17	Yeast	8693031
ornithine decarboxylase	4.1.1.17	Yeast	8707896
ornithine decarboxylase	4.1.1.17	Yeast	8727257
ornithine decarboxylase	4.1.1.17	Yeast	8760120
ornithine decarboxylase	4.1.1.17	Yeast	8760129
ornithine decarboxylase	4.1.1.17	Yeast	8768305
ornithine decarboxylase	4.1.1.17	Yeast	8777294
ornithine decarboxylase	4.1.1.17	Yeast	8814137
ornithine decarboxylase	4.1.1.17	Yeast	8848835
ornithine decarboxylase	4.1.1.17	Yeast	8858522
ornithine decarboxylase	4.1.1.17	Yeast	8878500
ornithine decarboxylase	4.1.1.17	Yeast	8882155
ornithine decarboxylase	4.1.1.17	Yeast	8912847
ornithine decarboxylase	4.1.1.17	Yeast	8944705
ornithine decarboxylase	4.1.1.17	Yeast	9009157
ornithine decarboxylase	4.1.1.17	Yeast	9016399
ornithine decarboxylase	4.1.1.17	Yeast	9017896
ornithine decarboxylase	4.1.1.17	Yeast	9022291
ornithine decarboxylase	4.1.1.17	Yeast	9024941
ornithine decarboxylase	4.1.1.17	Yeast	9063811
ornithine decarboxylase	4.1.1.17	Yeast	9073141
ornithine decarboxylase	4.1.1.17	Yeast	9134011
ornithine decarboxylase	4.1.1.17	Yeast	9142900
ornithine decarboxylase	4.1.1.17	Yeast	9191978
ornithine decarboxylase	4.1.1.17	Yeast	9213218
ornithine decarboxylase	4.1.1.17	Yeast	9223345
ornithine decarboxylase	4.1.1.17	Yeast	9224728
ornithine decarboxylase	4.1.1.17	Yeast	9251105
ornithine decarboxylase	4.1.1.17	Yeast	9252524
ornithine decarboxylase	4.1.1.17	Yeast	9294258

ornithine decarboxylase	4.1.1.17	Yeast	9368191
ornithine decarboxylase	4.1.1.17	Yeast	9371082
ornithine decarboxylase	4.1.1.17	Yeast	9454972
ornithine decarboxylase	4.1.1.17	Yeast	9458728
ornithine decarboxylase	4.1.1.17	Yeast	9468098
ornithine decarboxylase	4.1.1.17	Yeast	9495243
ornithine decarboxylase	4.1.1.17	Yeast	9525811
ornithine decarboxylase	4.1.1.17	Yeast	9590135
ornithine decarboxylase	4.1.1.17	Yeast	9609384
ornithine decarboxylase	4.1.1.17	Yeast	9612269
ornithine decarboxylase	4.1.1.17	Yeast	9624108
ornithine decarboxylase	4.1.1.17	Yeast	9648877
ornithine decarboxylase	4.1.1.17	Yeast	9685330
ornithine decarboxylase	4.1.1.17	Yeast	9688665
ornithine decarboxylase	4.1.1.17	Yeast	9769382
ornithine decarboxylase	4.1.1.17	Yeast	9772292
ornithine decarboxylase	4.1.1.17	Yeast	9795249
ornithine decarboxylase	4.1.1.17	Yeast	9806166
ornithine decarboxylase	4.1.1.17	Yeast	9829706
ornithine decarboxylase	4.1.1.17	Yeast	9868187
ornithine decarboxylase	4.1.1.17	Yeast	9884080
ornithine decarboxylase	4.1.1.17	Yeast	9890191
phosphoenolpyruvate carboxykinase (ATP)	4.1.1.49	Yeast	10230645
phosphoenolpyruvate carboxykinase (ATP)	4.1.1.49	Yeast	10409689
phosphoenolpyruvate carboxykinase (ATP)	4.1.1.49	Yeast	10416947
phosphoenolpyruvate carboxykinase (ATP)	4.1.1.49	Yeast	10508111
phosphoenolpyruvate carboxykinase (ATP)	4.1.1.49	Yeast	10805165
phosphoenolpyruvate carboxykinase (ATP)	4.1.1.49	Yeast	10909974
phosphoenolpyruvate carboxykinase (ATP)	4.1.1.49	Yeast	11038056
phosphoenolpyruvate carboxykinase (ATP)	4.1.1.49	Yeast	11553511
phosphoenolpyruvate carboxykinase (ATP)	4.1.1.49	Yeast	11557984
phosphoenolpyruvate carboxykinase (ATP)	4.1.1.49	Yeast	11677594
phosphoenolpyruvate carboxykinase (ATP)	4.1.1.49	Yeast	11679417
phosphoenolpyruvate carboxykinase (ATP)	4.1.1.49	Yeast	11700062
phosphoenolpyruvate carboxykinase (ATP)	4.1.1.49	Yeast	11728630
phosphoenolpyruvate carboxykinase (ATP)	4.1.1.49	Yeast	11741859
phosphoenolpyruvate carboxykinase (ATP)	4.1.1.49	Yeast	12020659
phosphoenolpyruvate carboxykinase (ATP)	4.1.1.49	Yeast	12089355
phosphoenolpyruvate carboxykinase (ATP)	4.1.1.49	Yeast	12455594
phosphoenolpyruvate carboxykinase (ATP)	4.1.1.49	Yeast	12485530
phosphoenolpyruvate carboxykinase (ATP)	4.1.1.49	Yeast	12644461
phosphoenolpyruvate carboxykinase (ATP)	4.1.1.49	Yeast	12646246
phosphoenolpyruvate carboxykinase (ATP)	4.1.1.49	Yeast	12757760

phosphoenolpyruvate carboxykinase (ATP)	4.1.1.49	Yeast	12865425
phosphoenolpyruvate carboxykinase (ATP)	4.1.1.49	Yeast	1315012
phosphoenolpyruvate carboxykinase (ATP)	4.1.1.49	Yeast	1398916
phosphoenolpyruvate carboxykinase (ATP)	4.1.1.49	Yeast	14739071
phosphoenolpyruvate carboxykinase (ATP)	4.1.1.49	Yeast	14739078
phosphoenolpyruvate carboxykinase (ATP)	4.1.1.49	Yeast	14739255
phosphoenolpyruvate carboxykinase (ATP)	4.1.1.49	Yeast	1510582
phosphoenolpyruvate carboxykinase (ATP)	4.1.1.49	Yeast	15265774
phosphoenolpyruvate carboxykinase (ATP)	4.1.1.49	Yeast	15733733
phosphoenolpyruvate carboxykinase (ATP)	4.1.1.49	Yeast	15907483
phosphoenolpyruvate carboxykinase (ATP)	4.1.1.49	Yeast	15983413
phosphoenolpyruvate carboxykinase (ATP)	4.1.1.49	Yeast	16236252
phosphoenolpyruvate carboxykinase (ATP)	4.1.1.49	Yeast	16249187
phosphoenolpyruvate carboxykinase (ATP)	4.1.1.49	Yeast	16324924
phosphoenolpyruvate carboxykinase (ATP)	4.1.1.49	Yeast	16330542
phosphoenolpyruvate carboxykinase (ATP)	4.1.1.49	Yeast	16458327
phosphoenolpyruvate carboxykinase (ATP)	4.1.1.49	Yeast	16503364
phosphoenolpyruvate carboxykinase (ATP)	4.1.1.49	Yeast	1653277
phosphoenolpyruvate carboxykinase (ATP)	4.1.1.49	Yeast	16819824
phosphoenolpyruvate carboxykinase (ATP)	4.1.1.49	Yeast	16941667
phosphoenolpyruvate carboxykinase (ATP)	4.1.1.49	Yeast	17097062
phosphoenolpyruvate carboxykinase (ATP)	4.1.1.49	Yeast	1720862
phosphoenolpyruvate carboxykinase (ATP)	4.1.1.49	Yeast	1733721
phosphoenolpyruvate carboxykinase (ATP)	4.1.1.49	Yeast	176867
phosphoenolpyruvate carboxykinase (ATP)	4.1.1.49	Yeast	1820332
phosphoenolpyruvate carboxykinase (ATP)	4.1.1.49	Yeast	1848696
phosphoenolpyruvate carboxykinase (ATP)	4.1.1.49	Yeast	2265217
phosphoenolpyruvate carboxykinase (ATP)	4.1.1.49	Yeast	25836
phosphoenolpyruvate carboxykinase (ATP)	4.1.1.49	Yeast	2919162
phosphoenolpyruvate carboxykinase (ATP)	4.1.1.49	Yeast	3023262
phosphoenolpyruvate carboxykinase (ATP)	4.1.1.49	Yeast	3059968
phosphoenolpyruvate carboxykinase (ATP)	4.1.1.49	Yeast	3068502
phosphoenolpyruvate carboxykinase (ATP)	4.1.1.49	Yeast	3203688
phosphoenolpyruvate carboxykinase (ATP)	4.1.1.49	Yeast	329666
phosphoenolpyruvate carboxykinase (ATP)	4.1.1.49	Yeast	3542066
phosphoenolpyruvate carboxykinase (ATP)	4.1.1.49	Yeast	3947691
phosphoenolpyruvate carboxykinase (ATP)	4.1.1.49	Yeast	3957187
phosphoenolpyruvate carboxykinase (ATP)	4.1.1.49	Yeast	422559
phosphoenolpyruvate carboxykinase (ATP)	4.1.1.49	Yeast	582464
phosphoenolpyruvate carboxykinase (ATP)	4.1.1.49	Yeast	6440018
phosphoenolpyruvate carboxykinase (ATP)	4.1.1.49	Yeast	6583707
phosphoenolpyruvate carboxykinase (ATP)	4.1.1.49	Yeast	6917781
phosphoenolpyruvate carboxykinase (ATP)	4.1.1.49	Yeast	7587654

phosphoenolpyruvate carboxykinase (ATP)	4.1.1.49	Yeast	7854322
phosphoenolpyruvate carboxykinase (ATP)	4.1.1.49	Yeast	7980440
phosphoenolpyruvate carboxykinase (ATP)	4.1.1.49	Yeast	8074220
phosphoenolpyruvate carboxykinase (ATP)	4.1.1.49	Yeast	8375031
phosphoenolpyruvate carboxykinase (ATP)	4.1.1.49	Yeast	8384841
phosphoenolpyruvate carboxykinase (ATP)	4.1.1.49	Yeast	8432541
phosphoenolpyruvate carboxykinase (ATP)	4.1.1.49	Yeast	8449898
phosphoenolpyruvate carboxykinase (ATP)	4.1.1.49	Yeast	8557765
phosphoenolpyruvate carboxykinase (ATP)	4.1.1.49	Yeast	8567635
phosphoenolpyruvate carboxykinase (ATP)	4.1.1.49	Yeast	8636258
phosphoenolpyruvate carboxykinase (ATP)	4.1.1.49	Yeast	9214454
phosphoenolpyruvate carboxykinase (ATP)	4.1.1.49	Yeast	9242918
phosphoenolpyruvate carboxykinase (ATP)	4.1.1.49	Yeast	9452431
phosphoenolpyruvate carboxykinase (ATP)	4.1.1.49	Yeast	9473304
phosphoenolpyruvate carboxykinase (ATP)	4.1.1.49	Yeast	9530152
phosphoenolpyruvate carboxykinase (ATP)	4.1.1.49	Yeast	9762796
phosphoenolpyruvate carboxykinase (ATP)	4.1.1.49	Yeast	9854182
adenosylmethionine decarboxylase	4.1.1.50	Yeast	10216947
adenosylmethionine decarboxylase	4.1.1.50	Yeast	10378277
adenosylmethionine decarboxylase	4.1.1.50	Yeast	10413038
adenosylmethionine decarboxylase	4.1.1.50	Yeast	10467042
adenosylmethionine decarboxylase	4.1.1.50	Yeast	10713131
adenosylmethionine decarboxylase	4.1.1.50	Yeast	10949915
adenosylmethionine decarboxylase	4.1.1.50	Yeast	11076965
adenosylmethionine decarboxylase	4.1.1.50	Yeast	11085920
adenosylmethionine decarboxylase	4.1.1.50	Yeast	11348531
adenosylmethionine decarboxylase	4.1.1.50	Yeast	11390378
adenosylmethionine decarboxylase	4.1.1.50	Yeast	11526206
adenosylmethionine decarboxylase	4.1.1.50	Yeast	11583148
adenosylmethionine decarboxylase	4.1.1.50	Yeast	11923270
adenosylmethionine decarboxylase	4.1.1.50	Yeast	12674502
adenosylmethionine decarboxylase	4.1.1.50	Yeast	14618239
adenosylmethionine decarboxylase	4.1.1.50	Yeast	15150268
adenosylmethionine decarboxylase	4.1.1.50	Yeast	15821146
adenosylmethionine decarboxylase	4.1.1.50	Yeast	16372273
adenosylmethionine decarboxylase	4.1.1.50	Yeast	16423999
adenosylmethionine decarboxylase	4.1.1.50	Yeast	16515461
adenosylmethionine decarboxylase	4.1.1.50	Yeast	16642382
adenosylmethionine decarboxylase	4.1.1.50	Yeast	16941339
adenosylmethionine decarboxylase	4.1.1.50	Yeast	2013278
adenosylmethionine decarboxylase	4.1.1.50	Yeast	2209170
adenosylmethionine decarboxylase	4.1.1.50	Yeast	2775206
adenosylmethionine decarboxylase	4.1.1.50	Yeast	3250232

adenosylmethionine decarboxylase	4.1.1.50	Yeast	4062886
adenosylmethionine decarboxylase	4.1.1.50	Yeast	7789170
adenosylmethionine decarboxylase	4.1.1.50	Yeast	7945201
adenosylmethionine decarboxylase	4.1.1.50	Yeast	8142949
adenosylmethionine decarboxylase	4.1.1.50	Yeast	8178573
adenosylmethionine decarboxylase	4.1.1.50	Yeast	8198469
adenosylmethionine decarboxylase	4.1.1.50	Yeast	8353934
adenosylmethionine decarboxylase	4.1.1.50	Yeast	8760129
adenosylmethionine decarboxylase	4.1.1.50	Yeast	8814137
adenosylmethionine decarboxylase	4.1.1.50	Yeast	8973561
adenosylmethionine decarboxylase	4.1.1.50	Yeast	9435790
adenosylmethionine decarboxylase	4.1.1.50	Yeast	9841864
adenosylmethionine decarboxylase	4.1.1.50	Yeast	9879883
fructose-bisphosphate aldolase	4.1.2.13	Yeast	10498814
fructose-bisphosphate aldolase	4.1.2.13	Yeast	12020659
fructose-bisphosphate aldolase	4.1.2.13	Yeast	12876349
fructose-bisphosphate aldolase	4.1.2.13	Yeast	15142555
fructose-bisphosphate aldolase	4.1.2.13	Yeast	15869466
fructose-bisphosphate aldolase	4.1.2.13	Yeast	16502329
fructose-bisphosphate aldolase	4.1.2.13	Yeast	1658253
fructose-bisphosphate aldolase	4.1.2.13	Yeast	1894606
fructose-bisphosphate aldolase	4.1.2.13	Yeast	6440018
fructose-bisphosphate aldolase	4.1.2.13	Yeast	678439
fructose-bisphosphate aldolase	4.1.2.13	Yeast	7388140
fructose-bisphosphate aldolase	4.1.2.13	Yeast	7786768
fructose-bisphosphate aldolase	4.1.2.13	Yeast	8015399
fructose-bisphosphate aldolase	4.1.2.13	Yeast	8913875
fructose-bisphosphate aldolase	4.1.2.13	Yeast	9163906
fructose-bisphosphate aldolase	4.1.2.13	Yeast	9473304
isocitrate lyase	4.1.3.1	Yeast	10439413
isocitrate lyase	4.1.3.1	Yeast	10805817
isocitrate lyase	4.1.3.1	Yeast	11038056
isocitrate lyase	4.1.3.1	Yeast	11526312
isocitrate lyase	4.1.3.1	Yeast	11888209
isocitrate lyase	4.1.3.1	Yeast	14558475
isocitrate lyase	4.1.3.1	Yeast	15299923
isocitrate lyase	4.1.3.1	Yeast	16603391
isocitrate lyase	4.1.3.1	Yeast	16856937
isocitrate lyase	4.1.3.1	Yeast	2209599
isocitrate lyase	4.1.3.1	Yeast	2361956
isocitrate lyase	4.1.3.1	Yeast	238950
isocitrate lyase	4.1.3.1	Yeast	6389540
isocitrate lyase	4.1.3.1	Yeast	9738442

anthranilate synthase	4.1.3.27	Yeast	10978548
anthranilate synthase	4.1.3.27	Yeast	15563620
anthranilate synthase	4.1.3.27	Yeast	15645305
anthranilate synthase	4.1.3.27	Yeast	15980261
anthranilate synthase	4.1.3.27	Yeast	16040654
anthranilate synthase	4.1.3.27	Yeast	3311153
anthranilate synthase	4.1.3.27	Yeast	378978
anthranilate synthase	4.1.3.27	Yeast	9299222
phosphopyruvate hydratase	4.2.1.11	Yeast	15459207
phosphopyruvate hydratase	4.2.1.11	Yeast	17437631
phosphopyruvate hydratase	4.2.1.11	Yeast	8651685
tryptophan synthase	4.2.1.20	Yeast	10433729
tryptophan synthase	4.2.1.20	Yeast	10769125
tryptophan synthase	4.2.1.20	Yeast	11756454
tryptophan synthase	4.2.1.20	Yeast	15542062
tryptophan synthase	4.2.1.20	Yeast	2183877
tryptophan synthase	4.2.1.20	Yeast	2185841
tryptophan synthase	4.2.1.20	Yeast	3297161
tryptophan synthase	4.2.1.20	Yeast	8193152
cystathionine beta-synthase	4.2.1.22	Yeast	11051561
cystathionine beta-synthase	4.2.1.22	Yeast	11106665
cystathionine beta-synthase	4.2.1.22	Yeast	12198128
cystathionine beta-synthase	4.2.1.22	Yeast	15581575
cystathionine beta-synthase	4.2.1.22	Yeast	15642325
cystathionine beta-synthase	4.2.1.22	Yeast	16096271
cystathionine beta-synthase	4.2.1.22	Yeast	16769053
cystathionine beta-synthase	4.2.1.22	Yeast	17629356
porphobilinogen synthase	4.2.1.24	Yeast	10634305
porphobilinogen synthase	4.2.1.24	Yeast	10787385
porphobilinogen synthase	4.2.1.24	Yeast	15141099
porphobilinogen synthase	4.2.1.24	Yeast	15259603
porphobilinogen synthase	4.2.1.24	Yeast	1526942
porphobilinogen synthase	4.2.1.24	Yeast	16819823
porphobilinogen synthase	4.2.1.24	Yeast	1959865
porphobilinogen synthase	4.2.1.24	Yeast	2050126
porphobilinogen synthase	4.2.1.24	Yeast	2317819
porphobilinogen synthase	4.2.1.24	Yeast	3009001
porphobilinogen synthase	4.2.1.24	Yeast	3755290
porphobilinogen synthase	4.2.1.24	Yeast	3840094
porphobilinogen synthase	4.2.1.24	Yeast	3966797
porphobilinogen synthase	4.2.1.24	Yeast	4265023
porphobilinogen synthase	4.2.1.24	Yeast	6547609
porphobilinogen synthase	4.2.1.24	Yeast	6688350

porphobilinogen synthase	4.2.1.24	Yeast	6873612
porphobilinogen synthase	4.2.1.24	Yeast	7436670
aconitate hydratase	4.2.1.3	Yeast	11295257
aconitate hydratase	4.2.1.3	Yeast	11329290
aconitate hydratase	4.2.1.3	Yeast	15149735
aconitate hydratase	4.2.1.3	Yeast	15543948
aconitate hydratase	4.2.1.3	Yeast	16094633
aconitate hydratase	4.2.1.3	Yeast	16201454
aconitate hydratase	4.2.1.3	Yeast	7589784
aconitate hydratase	4.2.1.3	Yeast	8115279
prephenate dehydratase	4.2.1.51	Yeast	11326337
prephenate dehydratase	4.2.1.51	Yeast	14749915
prephenate dehydratase	4.2.1.51	Yeast	14749916
prephenate dehydratase	4.2.1.51	Yeast	14749917
prephenate dehydratase	4.2.1.51	Yeast	14749918
prephenate dehydratase	4.2.1.51	Yeast	15753077
prephenate dehydratase	4.2.1.51	Yeast	6150022
cystathionine gamma-lyase	4.4.1.1	Yeast	10960449
cystathionine gamma-lyase	4.4.1.1	Yeast	15038791
cystathionine gamma-lyase	4.4.1.1	Yeast	15347670
cystathionine gamma-lyase	4.4.1.1	Yeast	16786305
cystathionine gamma-lyase	4.4.1.1	Yeast	16946488
cystathionine gamma-lyase	4.4.1.1	Yeast	17095121
cystathionine gamma-lyase	4.4.1.1	Yeast	17525332
cystathionine gamma-lyase	4.4.1.1	Yeast	8973544
UDP-glucose 4-epimerase	5.1.3.2	Yeast	1149741
phosphoglycerate mutase	5.4.2.1	Yeast	15181008
phosphoglycerate mutase	5.4.2.1	Yeast	17204863
phosphoglycerate mutase	5.4.2.1	Yeast	2830218
phosphoglucomutase	5.4.2.2	Yeast	11102370
phosphoglucomutase	5.4.2.2	Yeast	1149741
phosphoglucomutase	5.4.2.2	Yeast	12026175
phosphoglucomutase	5.4.2.2	Yeast	12791685
phosphoglucomutase	5.4.2.2	Yeast	15378030
phosphoglucomutase	5.4.2.2	Yeast	15996095
phosphoglucomutase	5.4.2.2	Yeast	16046289
lanosterol synthase	5.4.99.7	Yeast	11995928
lanosterol synthase	5.4.99.7	Yeast	15763540
lanosterol synthase	5.4.99.7	Yeast	17088293
inositol-3-phosphate synthase	5.5.1.4	Yeast	10713523
inositol-3-phosphate synthase	5.5.1.4	Yeast	11779862
inositol-3-phosphate synthase	5.5.1.4	Yeast	12005437
inositol-3-phosphate synthase	5.5.1.4	Yeast	12941308

inositol-3-phosphate synthase	5.5.1.4	Yeast	14550537
inositol-3-phosphate synthase	5.5.1.4	Yeast	14684747
inositol-3-phosphate synthase	5.5.1.4	Yeast	14730448
inositol-3-phosphate synthase	5.5.1.4	Yeast	1587797
inositol-3-phosphate synthase	5.5.1.4	Yeast	15965017
inositol-3-phosphate synthase	5.5.1.4	Yeast	16453101
inositol-3-phosphate synthase	5.5.1.4	Yeast	6687929
inositol-3-phosphate synthase	5.5.1.4	Yeast	9106505
acetate---CoA ligase	6.2.1.1	Yeast	15236963
acetate---CoA ligase	6.2.1.1	Yeast	15899897
acetate---CoA ligase	6.2.1.1	Yeast	2902801
acetate---CoA ligase	6.2.1.1	Yeast	4149947
acetate---CoA ligase	6.2.1.1	Yeast	8218953
long-chain-fatty-acid---CoA ligase	6.2.1.3	Yeast	11375393
long-chain-fatty-acid---CoA ligase	6.2.1.3	Yeast	1769731
succinate---CoA ligase (ADP-forming)	6.2.1.5	Yeast	16101500
succinate---CoA ligase (ADP-forming)	6.2.1.5	Yeast	1986797
succinate---CoA ligase (ADP-forming)	6.2.1.5	Yeast	3108130
succinate---CoA ligase (ADP-forming)	6.2.1.5	Yeast	3746465
succinate---CoA ligase (ADP-forming)	6.2.1.5	Yeast	6109001
succinate---CoA ligase (ADP-forming)	6.2.1.5	Yeast	7017725
succinate---CoA ligase (ADP-forming)	6.2.1.5	Yeast	7062046
succinate---CoA ligase (ADP-forming)	6.2.1.5	Yeast	7783627
glutamate---ammonia ligase	6.3.1.2	Yeast	10092169
glutamate---ammonia ligase	6.3.1.2	Yeast	10383611
glutamate---ammonia ligase	6.3.1.2	Yeast	10440891
glutamate---ammonia ligase	6.3.1.2	Yeast	10664131
glutamate---ammonia ligase	6.3.1.2	Yeast	11069692
glutamate---ammonia ligase	6.3.1.2	Yeast	11413247
glutamate---ammonia ligase	6.3.1.2	Yeast	11939529
glutamate---ammonia ligase	6.3.1.2	Yeast	12232192
glutamate---ammonia ligase	6.3.1.2	Yeast	12517141
glutamate---ammonia ligase	6.3.1.2	Yeast	12552916
glutamate---ammonia ligase	6.3.1.2	Yeast	14714472
glutamate---ammonia ligase	6.3.1.2	Yeast	14723991
glutamate---ammonia ligase	6.3.1.2	Yeast	15130478
glutamate---ammonia ligase	6.3.1.2	Yeast	15489445
glutamate---ammonia ligase	6.3.1.2	Yeast	15581577
glutamate---ammonia ligase	6.3.1.2	Yeast	1612427
glutamate---ammonia ligase	6.3.1.2	Yeast	16687472
glutamate---ammonia ligase	6.3.1.2	Yeast	16829528
glutamate---ammonia ligase	6.3.1.2	Yeast	16946267
glutamate---ammonia ligase	6.3.1.2	Yeast	2434618

glutamate---ammonia ligase	6.3.1.2	Yeast	26663
glutamate---ammonia ligase	6.3.1.2	Yeast	6118373
glutamate---ammonia ligase	6.3.1.2	Yeast	9624228
glutamate---cysteine ligase	6.3.2.2	Yeast	10215022
glutamate---cysteine ligase	6.3.2.2	Yeast	10218647
glutamate---cysteine ligase	6.3.2.2	Yeast	10349842
glutamate---cysteine ligase	6.3.2.2	Yeast	10385608
glutamate---cysteine ligase	6.3.2.2	Yeast	10385658
glutamate---cysteine ligase	6.3.2.2	Yeast	10399958
glutamate---cysteine ligase	6.3.2.2	Yeast	10439045
glutamate---cysteine ligase	6.3.2.2	Yeast	10441483
glutamate---cysteine ligase	6.3.2.2	Yeast	10486302
glutamate---cysteine ligase	6.3.2.2	Yeast	10515588
glutamate---cysteine ligase	6.3.2.2	Yeast	10518117
glutamate---cysteine ligase	6.3.2.2	Yeast	10544055
glutamate---cysteine ligase	6.3.2.2	Yeast	10544272
glutamate---cysteine ligase	6.3.2.2	Yeast	10590319
glutamate---cysteine ligase	6.3.2.2	Yeast	10593589
glutamate---cysteine ligase	6.3.2.2	Yeast	10594104
glutamate---cysteine ligase	6.3.2.2	Yeast	10600876
glutamate---cysteine ligase	6.3.2.2	Yeast	10623879
glutamate---cysteine ligase	6.3.2.2	Yeast	10644053
glutamate---cysteine ligase	6.3.2.2	Yeast	10674357
glutamate---cysteine ligase	6.3.2.2	Yeast	10677377
glutamate---cysteine ligase	6.3.2.2	Yeast	10702364
glutamate---cysteine ligase	6.3.2.2	Yeast	10719238
glutamate---cysteine ligase	6.3.2.2	Yeast	10733945
glutamate---cysteine ligase	6.3.2.2	Yeast	10748080
glutamate---cysteine ligase	6.3.2.2	Yeast	10773686
glutamate---cysteine ligase	6.3.2.2	Yeast	10777712
glutamate---cysteine ligase	6.3.2.2	Yeast	10802223
glutamate---cysteine ligase	6.3.2.2	Yeast	10928075
glutamate---cysteine ligase	6.3.2.2	Yeast	10960449
glutamate---cysteine ligase	6.3.2.2	Yeast	11007940
glutamate---cysteine ligase	6.3.2.2	Yeast	11025451
glutamate---cysteine ligase	6.3.2.2	Yeast	11028671
glutamate---cysteine ligase	6.3.2.2	Yeast	11032771
glutamate---cysteine ligase	6.3.2.2	Yeast	11097862
glutamate---cysteine ligase	6.3.2.2	Yeast	11133045
glutamate---cysteine ligase	6.3.2.2	Yeast	11157875
glutamate---cysteine ligase	6.3.2.2	Yeast	11163433
glutamate---cysteine ligase	6.3.2.2	Yeast	11181934
glutamate---cysteine ligase	6.3.2.2	Yeast	11233143

glutamate---cysteine ligase	6.3.2.2	Yeast	11306445
glutamate---cysteine ligase	6.3.2.2	Yeast	11339815
glutamate---cysteine ligase	6.3.2.2	Yeast	11352989
glutamate---cysteine ligase	6.3.2.2	Yeast	11353135
glutamate---cysteine ligase	6.3.2.2	Yeast	11500053
glutamate---cysteine ligase	6.3.2.2	Yeast	11560771
glutamate---cysteine ligase	6.3.2.2	Yeast	11565956
glutamate---cysteine ligase	6.3.2.2	Yeast	11687904
glutamate---cysteine ligase	6.3.2.2	Yeast	11705692
glutamate---cysteine ligase	6.3.2.2	Yeast	11780957
glutamate---cysteine ligase	6.3.2.2	Yeast	11781188
glutamate---cysteine ligase	6.3.2.2	Yeast	11790356
glutamate---cysteine ligase	6.3.2.2	Yeast	11812649
glutamate---cysteine ligase	6.3.2.2	Yeast	11820781
glutamate---cysteine ligase	6.3.2.2	Yeast	11841806
glutamate---cysteine ligase	6.3.2.2	Yeast	11849402
glutamate---cysteine ligase	6.3.2.2	Yeast	11876501
glutamate---cysteine ligase	6.3.2.2	Yeast	11970852
glutamate---cysteine ligase	6.3.2.2	Yeast	12079521
glutamate---cysteine ligase	6.3.2.2	Yeast	12111865
glutamate---cysteine ligase	6.3.2.2	Yeast	12147223
glutamate---cysteine ligase	6.3.2.2	Yeast	12196927
glutamate---cysteine ligase	6.3.2.2	Yeast	12200125
glutamate---cysteine ligase	6.3.2.2	Yeast	12204877
glutamate---cysteine ligase	6.3.2.2	Yeast	12433058
glutamate---cysteine ligase	6.3.2.2	Yeast	12448821
glutamate---cysteine ligase	6.3.2.2	Yeast	12452384
glutamate---cysteine ligase	6.3.2.2	Yeast	12535742
glutamate---cysteine ligase	6.3.2.2	Yeast	12594957
glutamate---cysteine ligase	6.3.2.2	Yeast	12601050
glutamate---cysteine ligase	6.3.2.2	Yeast	12607907
glutamate---cysteine ligase	6.3.2.2	Yeast	12628495
glutamate---cysteine ligase	6.3.2.2	Yeast	12637989
glutamate---cysteine ligase	6.3.2.2	Yeast	12814619
glutamate---cysteine ligase	6.3.2.2	Yeast	12882455
glutamate---cysteine ligase	6.3.2.2	Yeast	12913252
glutamate---cysteine ligase	6.3.2.2	Yeast	1350904
glutamate---cysteine ligase	6.3.2.2	Yeast	1351382
glutamate---cysteine ligase	6.3.2.2	Yeast	1353765
glutamate---cysteine ligase	6.3.2.2	Yeast	1355406
glutamate---cysteine ligase	6.3.2.2	Yeast	1362226
glutamate---cysteine ligase	6.3.2.2	Yeast	13679058
glutamate---cysteine ligase	6.3.2.2	Yeast	14500406

glutamate---cysteine ligase	6.3.2.2	Yeast	14514673
glutamate---cysteine ligase	6.3.2.2	Yeast	14744626
glutamate---cysteine ligase	6.3.2.2	Yeast	14962359
glutamate---cysteine ligase	6.3.2.2	Yeast	15020643
glutamate---cysteine ligase	6.3.2.2	Yeast	15050748
glutamate---cysteine ligase	6.3.2.2	Yeast	15257546
glutamate---cysteine ligase	6.3.2.2	Yeast	15314090
glutamate---cysteine ligase	6.3.2.2	Yeast	15374419
glutamate---cysteine ligase	6.3.2.2	Yeast	15451066
glutamate---cysteine ligase	6.3.2.2	Yeast	15477603
glutamate---cysteine ligase	6.3.2.2	Yeast	15509664
glutamate---cysteine ligase	6.3.2.2	Yeast	15878398
glutamate---cysteine ligase	6.3.2.2	Yeast	15946948
glutamate---cysteine ligase	6.3.2.2	Yeast	16032782
glutamate---cysteine ligase	6.3.2.2	Yeast	16081425
glutamate---cysteine ligase	6.3.2.2	Yeast	16162662
glutamate---cysteine ligase	6.3.2.2	Yeast	16183645
glutamate---cysteine ligase	6.3.2.2	Yeast	16549430
glutamate---cysteine ligase	6.3.2.2	Yeast	16566126
glutamate---cysteine ligase	6.3.2.2	Yeast	1678010
glutamate---cysteine ligase	6.3.2.2	Yeast	16781460
glutamate---cysteine ligase	6.3.2.2	Yeast	16949561
glutamate---cysteine ligase	6.3.2.2	Yeast	16960387
glutamate---cysteine ligase	6.3.2.2	Yeast	17144898
glutamate---cysteine ligase	6.3.2.2	Yeast	1784629
glutamate---cysteine ligase	6.3.2.2	Yeast	1970723
glutamate---cysteine ligase	6.3.2.2	Yeast	1997009
glutamate---cysteine ligase	6.3.2.2	Yeast	2572174
glutamate---cysteine ligase	6.3.2.2	Yeast	2574245
glutamate---cysteine ligase	6.3.2.2	Yeast	2879531
glutamate---cysteine ligase	6.3.2.2	Yeast	2897858
glutamate---cysteine ligase	6.3.2.2	Yeast	2901982
glutamate---cysteine ligase	6.3.2.2	Yeast	3621155
glutamate---cysteine ligase	6.3.2.2	Yeast	6897891
glutamate---cysteine ligase	6.3.2.2	Yeast	7351635
glutamate---cysteine ligase	6.3.2.2	Yeast	7503776
glutamate---cysteine ligase	6.3.2.2	Yeast	7568279
glutamate---cysteine ligase	6.3.2.2	Yeast	7570642
glutamate---cysteine ligase	6.3.2.2	Yeast	7585502
glutamate---cysteine ligase	6.3.2.2	Yeast	7622006
glutamate---cysteine ligase	6.3.2.2	Yeast	7651354
glutamate---cysteine ligase	6.3.2.2	Yeast	7768207
glutamate---cysteine ligase	6.3.2.2	Yeast	7901332

glutamate---cysteine ligase	6.3.2.2	Yeast	7908245
glutamate---cysteine ligase	6.3.2.2	Yeast	7910419
glutamate---cysteine ligase	6.3.2.2	Yeast	7929374
glutamate---cysteine ligase	6.3.2.2	Yeast	7955076
glutamate---cysteine ligase	6.3.2.2	Yeast	7969079
glutamate---cysteine ligase	6.3.2.2	Yeast	8001239
glutamate---cysteine ligase	6.3.2.2	Yeast	8065332
glutamate---cysteine ligase	6.3.2.2	Yeast	8101766
glutamate---cysteine ligase	6.3.2.2	Yeast	8106072
glutamate---cysteine ligase	6.3.2.2	Yeast	8120650
glutamate---cysteine ligase	6.3.2.2	Yeast	8538700
glutamate---cysteine ligase	6.3.2.2	Yeast	8582653
glutamate---cysteine ligase	6.3.2.2	Yeast	8648118
glutamate---cysteine ligase	6.3.2.2	Yeast	8661240
glutamate---cysteine ligase	6.3.2.2	Yeast	8751598
glutamate---cysteine ligase	6.3.2.2	Yeast	8781554
glutamate---cysteine ligase	6.3.2.2	Yeast	8792848
glutamate---cysteine ligase	6.3.2.2	Yeast	8806884
glutamate---cysteine ligase	6.3.2.2	Yeast	8843715
glutamate---cysteine ligase	6.3.2.2	Yeast	8917676
glutamate---cysteine ligase	6.3.2.2	Yeast	8930687
glutamate---cysteine ligase	6.3.2.2	Yeast	8947504
glutamate---cysteine ligase	6.3.2.2	Yeast	8973794
glutamate---cysteine ligase	6.3.2.2	Yeast	8981036
glutamate---cysteine ligase	6.3.2.2	Yeast	8995480
glutamate---cysteine ligase	6.3.2.2	Yeast	9029270
glutamate---cysteine ligase	6.3.2.2	Yeast	9054446
glutamate---cysteine ligase	6.3.2.2	Yeast	9063478
glutamate---cysteine ligase	6.3.2.2	Yeast	9093011
glutamate---cysteine ligase	6.3.2.2	Yeast	9119067
glutamate---cysteine ligase	6.3.2.2	Yeast	9157984
glutamate---cysteine ligase	6.3.2.2	Yeast	9163779
glutamate---cysteine ligase	6.3.2.2	Yeast	9185621
glutamate---cysteine ligase	6.3.2.2	Yeast	9214623
glutamate---cysteine ligase	6.3.2.2	Yeast	9259355
glutamate---cysteine ligase	6.3.2.2	Yeast	9268987
glutamate---cysteine ligase	6.3.2.2	Yeast	9288403
glutamate---cysteine ligase	6.3.2.2	Yeast	9311606
glutamate---cysteine ligase	6.3.2.2	Yeast	9374111
glutamate---cysteine ligase	6.3.2.2	Yeast	9389600
glutamate---cysteine ligase	6.3.2.2	Yeast	9393741
glutamate---cysteine ligase	6.3.2.2	Yeast	9425930
glutamate---cysteine ligase	6.3.2.2	Yeast	9582278

glutamate---cysteine ligase	6.3.2.2	Yeast	9614065
glutamate---cysteine ligase	6.3.2.2	Yeast	9626582
glutamate---cysteine ligase	6.3.2.2	Yeast	9647756
glutamate---cysteine ligase	6.3.2.2	Yeast	9679558
glutamate---cysteine ligase	6.3.2.2	Yeast	9703946
glutamate---cysteine ligase	6.3.2.2	Yeast	9729439
glutamate---cysteine ligase	6.3.2.2	Yeast	9750167
glutamate---cysteine ligase	6.3.2.2	Yeast	9756861
glutamate---cysteine ligase	6.3.2.2	Yeast	9762423
glutamate---cysteine ligase	6.3.2.2	Yeast	9875552
glutamate---cysteine ligase	6.3.2.2	Yeast	9895302
glutathione synthase	6.3.2.3	Yeast	10964706
glutathione synthase	6.3.2.3	Yeast	11708780
glutathione synthase	6.3.2.3	Yeast	9880348
phosphoribosylaminoimidazolesuccinocarboxamide synthase	6.3.2.6	Yeast	701284
CTP synthase	6.3.4.2	Yeast	12678497
CTP synthase	6.3.4.2	Yeast	16820675
CTP synthase	6.3.4.2	Yeast	17463002
CTP synthase	6.3.4.2	Yeast	17681942
CTP synthase	6.3.4.2	Yeast	2787169
argininosuccinate synthase	6.3.4.5	Yeast	10709858
argininosuccinate synthase	6.3.4.5	Yeast	1122920
argininosuccinate synthase	6.3.4.5	Yeast	11556547
argininosuccinate synthase	6.3.4.5	Yeast	12618329
argininosuccinate synthase	6.3.4.5	Yeast	12672181
argininosuccinate synthase	6.3.4.5	Yeast	1372742
argininosuccinate synthase	6.3.4.5	Yeast	14571701
argininosuccinate synthase	6.3.4.5	Yeast	15588718
argininosuccinate synthase	6.3.4.5	Yeast	16085056
argininosuccinate synthase	6.3.4.5	Yeast	16787144
argininosuccinate synthase	6.3.4.5	Yeast	558104
argininosuccinate synthase	6.3.4.5	Yeast	845694
argininosuccinate synthase	6.3.4.5	Yeast	8616812
argininosuccinate synthase	6.3.4.5	Yeast	8798625
argininosuccinate synthase	6.3.4.5	Yeast	9096605
argininosuccinate synthase	6.3.4.5	Yeast	9176259
argininosuccinate synthase	6.3.4.5	Yeast	9211993
argininosuccinate synthase	6.3.4.5	Yeast	9252090
carbamoyl-phosphate synthase (glutamine-hydrolysing)	6.3.5.5	Yeast	10659854
carbamoyl-phosphate synthase (glutamine-hydrolysing)	6.3.5.5	Yeast	10736367

carbamoyl-phosphate synthase (glutamine-hydrolysing)	6.3.5.5	Yeast	11441057
carbamoyl-phosphate synthase (glutamine-hydrolysing)	6.3.5.5	Yeast	11872754
carbamoyl-phosphate synthase (glutamine-hydrolysing)	6.3.5.5	Yeast	11956684
carbamoyl-phosphate synthase (glutamine-hydrolysing)	6.3.5.5	Yeast	12678497
carbamoyl-phosphate synthase (glutamine-hydrolysing)	6.3.5.5	Yeast	15326225
carbamoyl-phosphate synthase (glutamine-hydrolysing)	6.3.5.5	Yeast	15453495
carbamoyl-phosphate synthase (glutamine-hydrolysing)	6.3.5.5	Yeast	4018077
carbamoyl-phosphate synthase (glutamine-hydrolysing)	6.3.5.5	Yeast	4.0927E+13
carbamoyl-phosphate synthase (glutamine-hydrolysing)	6.3.5.5	Yeast	6115855
carbamoyl-phosphate synthase (glutamine-hydrolysing)	6.3.5.5	Yeast	6408083
carbamoyl-phosphate synthase (glutamine-hydrolysing)	6.3.5.5	Yeast	7053379
carbamoyl-phosphate synthase (glutamine-hydrolysing)	6.3.5.5	Yeast	7209543
carbamoyl-phosphate synthase (glutamine-hydrolysing)	6.3.5.5	Yeast	7608487
carbamoyl-phosphate synthase (glutamine-hydrolysing)	6.3.5.5	Yeast	7916269
pyruvate carboxylase	6.4.1.1	Yeast	10323732
pyruvate carboxylase	6.4.1.1	Yeast	16325442
pyruvate carboxylase	6.4.1.1	Yeast	3182810
pyruvate carboxylase	6.4.1.1	Yeast	6721853
acetyl-CoA carboxylase	6.4.1.2	Yeast	10098661
acetyl-CoA carboxylase	6.4.1.2	Yeast	10215591
acetyl-CoA carboxylase	6.4.1.2	Yeast	10757783
acetyl-CoA carboxylase	6.4.1.2	Yeast	10945143
acetyl-CoA carboxylase	6.4.1.2	Yeast	11078738
acetyl-CoA carboxylase	6.4.1.2	Yeast	11205884
acetyl-CoA carboxylase	6.4.1.2	Yeast	11504381
acetyl-CoA carboxylase	6.4.1.2	Yeast	11515553
acetyl-CoA carboxylase	6.4.1.2	Yeast	11546765
acetyl-CoA carboxylase	6.4.1.2	Yeast	12440972
acetyl-CoA carboxylase	6.4.1.2	Yeast	14627750

acetyl-CoA carboxylase	6.4.1.2	Yeast	15333468
acetyl-CoA carboxylase	6.4.1.2	Yeast	15607423
acetyl-CoA carboxylase	6.4.1.2	Yeast	15607568
acetyl-CoA carboxylase	6.4.1.2	Yeast	16222055
acetyl-CoA carboxylase	6.4.1.2	Yeast	16707454
acetyl-CoA carboxylase	6.4.1.2	Yeast	16968879
acetyl-CoA carboxylase	6.4.1.2	Yeast	17266990
acetyl-CoA carboxylase	6.4.1.2	Yeast	17653193
acetyl-CoA carboxylase	6.4.1.2	Yeast	1978829
acetyl-CoA carboxylase	6.4.1.2	Yeast	2570725
acetyl-CoA carboxylase	6.4.1.2	Yeast	2861941
acetyl-CoA carboxylase	6.4.1.2	Yeast	2894828
acetyl-CoA carboxylase	6.4.1.2	Yeast	7436865
acetyl-CoA carboxylase	6.4.1.2	Yeast	7903266
acetyl-CoA carboxylase	6.4.1.2	Yeast	7915138
acetyl-CoA carboxylase	6.4.1.2	Yeast	8814137
acetyl-CoA carboxylase	6.4.1.2	Yeast	9028876
acetyl-CoA carboxylase	6.4.1.2	Yeast	9082912
acetyl-CoA carboxylase	6.4.1.2	Yeast	9109840
