# Supplementary material for: High similarity of phylogenetic profiles of rate-limiting enzymes with inhibitory relation in Human, Mouse, Rat, budding Yeast and E. coli
Source: BMC Genomics. 2011 Nov 30;12(Suppl 3):S10. doi: 10.1186/1471-2164-12-S3-S10 (PMC3333169; doi:10.1186/1471-2164-12-S3-S10)
Supplement: Additional file 2 — The conservative inhibitory relations initiated by ADP and AMP produced by RLEs in Human, Mouse, Rat, budding Yeast and E. coli The conservative inhibitory relations initiated by ADP (171 pairs) and AMP (32 pairs), products of RLEs, are listed in Additional file 2. [file 1471-2164-12-S3-S10-S2.pdf]

Additional 2: The conservative inhibitory relations initiated by ADP and AMP produced by RLEs in Human, Mouse, Rat, budding Yeast and *E . coli*

The conservative inhibitory relations initiated by ADP (171 pairs) and AMP (32 pairs), products of RLEs, are listed in Additional file 2.

Inhibitor provider(rate-limiting enzyme)	Inhibited target	Species	Inhibitor
1.17.4.1	1.2.1.12	E. coli	ADP
1.17.4.1	1.2.1.12	Human	ADP
1.17.4.1	1.2.1.12	Rat	ADP
1.17.4.1	2.7.1.20	Human	ADP
1.17.4.1	2.7.1.20	Mouse	ADP
1.17.4.1	2.7.1.20	Rat	ADP
1.17.4.1	2.7.1.67	Human	ADP
1.17.4.1	2.7.1.67	Rat	ADP
1.17.4.1	2.7.1.67	Yeast	ADP
1.17.4.1	2.7.4.9	Human	ADP
1.17.4.1	2.7.4.9	Mouse	ADP
1.17.4.1	2.7.4.9	Yeast	ADP
1.17.4.1	2.7.6.1	E. coli	ADP
1.17.4.1	2.7.6.1	Human	ADP
1.17.4.1	2.7.6.1	Mouse	ADP
1.17.4.1	2.7.6.1	Rat	ADP
1.17.4.1	3.1.3.5	E. coli	ADP
1.17.4.1	3.1.3.5	Human	ADP
1.17.4.1	3.1.3.5	Mouse	ADP
1.17.4.1	3.1.3.5	Rat	ADP
2.3.3.8	2.7.1.20	Human	ADP
2.3.3.8	2.7.1.20	Mouse	ADP
2.3.3.8	2.7.1.20	Rat	ADP
2.3.3.8	2.7.6.1	Human	ADP
2.3.3.8	2.7.6.1	Mouse	ADP
2.3.3.8	2.7.6.1	Rat	ADP
2.3.3.8	3.1.3.5	Human	ADP
2.3.3.8	3.1.3.5	Mouse	ADP
2.3.3.8	3.1.3.5	Rat	ADP
2.7.1.1	2.7.1.20	Human	ADP
2.7.1.1	2.7.1.20	Mouse	ADP
2.7.1.1	2.7.1.20	Rat	ADP
2.7.1.1	2.7.1.67	Human	ADP
2.7.1.1	2.7.1.67	Rat	ADP
2.7.1.1	2.7.1.67	Yeast	ADP
2.7.1.1	2.7.4.9	Human	ADP

2.7.1.1	2.7.4.9	Mouse	ADP
2.7.1.1	2.7.4.9	Yeast	ADP
2.7.1.1	2.7.6.1	Human	ADP
2.7.1.1	2.7.6.1	Mouse	ADP
2.7.1.1	2.7.6.1	Rat	ADP
2.7.1.1	3.1.3.5	Human	ADP
2.7.1.1	3.1.3.5	Mouse	ADP
2.7.1.1	3.1.3.5	Rat	ADP
2.7.1.11	1.2.1.12	E. coli	ADP
2.7.1.11	1.2.1.12	Human	ADP
2.7.1.11	1.2.1.12	Rat	ADP
2.7.1.11	2.7.1.20	Human	ADP
2.7.1.11	2.7.1.20	Mouse	ADP
2.7.1.11	2.7.1.20	Rat	ADP
2.7.1.11	2.7.1.67	Human	ADP
2.7.1.11	2.7.1.67	Rat	ADP
2.7.1.11	2.7.1.67	Yeast	ADP
2.7.1.11	2.7.4.9	Human	ADP
2.7.1.11	2.7.4.9	Mouse	ADP
2.7.1.11	2.7.4.9	Yeast	ADP
2.7.1.11	2.7.6.1	E. coli	ADP
2.7.1.11	2.7.6.1	Human	ADP
2.7.1.11	2.7.6.1	Mouse	ADP
2.7.1.11	2.7.6.1	Rat	ADP
2.7.1.11	3.1.3.5	E. coli	ADP
2.7.1.11	3.1.3.5	Human	ADP
2.7.1.11	3.1.3.5	Mouse	ADP
2.7.1.11	3.1.3.5	Rat	ADP
2.7.1.127	2.7.1.20	Human	ADP
2.7.1.127	2.7.1.20	Mouse	ADP
2.7.1.127	2.7.1.20	Rat	ADP
2.7.1.127	2.7.6.1	Human	ADP
2.7.1.127	2.7.6.1	Mouse	ADP
2.7.1.127	2.7.6.1	Rat	ADP
2.7.1.127	3.1.3.5	Human	ADP
2.7.1.127	3.1.3.5	Mouse	ADP
2.7.1.127	3.1.3.5	Rat	ADP
2.7.1.137	2.7.1.20	Human	ADP
2.7.1.137	2.7.1.20	Mouse	ADP
2.7.1.137	2.7.1.20	Rat	ADP
2.7.1.137	2.7.1.67	Human	ADP
2.7.1.137	2.7.1.67	Rat	ADP
2.7.1.137	2.7.1.67	Yeast	ADP

2.7.1.137	2.7.4.9	Human	ADP
2.7.1.137	2.7.4.9	Mouse	ADP
2.7.1.137	2.7.4.9	Yeast	ADP
2.7.1.137	2.7.6.1	Human	ADP
2.7.1.137	2.7.6.1	Mouse	ADP
2.7.1.137	2.7.6.1	Rat	ADP
2.7.1.137	3.1.3.5	Human	ADP
2.7.1.137	3.1.3.5	Mouse	ADP
2.7.1.137	3.1.3.5	Rat	ADP
2.7.1.2	1.2.1.12	E. coli	ADP
2.7.1.2	1.2.1.12	Human	ADP
2.7.1.2	1.2.1.12	Rat	ADP
2.7.1.2	2.7.1.20	Human	ADP
2.7.1.2	2.7.1.20	Mouse	ADP
2.7.1.2	2.7.1.20	Rat	ADP
2.7.1.2	2.7.1.67	Human	ADP
2.7.1.2	2.7.1.67	Rat	ADP
2.7.1.2	2.7.1.67	Yeast	ADP
2.7.1.2	2.7.4.9	Human	ADP
2.7.1.2	2.7.4.9	Mouse	ADP
2.7.1.2	2.7.4.9	Yeast	ADP
2.7.1.2	2.7.6.1	E. coli	ADP
2.7.1.2	2.7.6.1	Human	ADP
2.7.1.2	2.7.6.1	Mouse	ADP
2.7.1.2	2.7.6.1	Rat	ADP
2.7.1.2	3.1.3.5	E. coli	ADP
2.7.1.2	3.1.3.5	Human	ADP
2.7.1.2	3.1.3.5	Mouse	ADP
2.7.1.2	3.1.3.5	Rat	ADP
2.7.1.20	1.1.1.205	Human	AMP
2.7.1.20	1.1.1.205	Mouse	AMP
2.7.1.20	1.1.1.205	Rat	AMP
2.7.1.20	2.4.2.14	Human	AMP
2.7.1.20	2.4.2.14	Mouse	AMP
2.7.1.20	2.4.2.14	Rat	AMP
2.7.1.20	2.7.1.67	Human	ADP
2.7.1.20	2.7.1.67	Rat	ADP
2.7.1.20	2.7.1.67	Yeast	ADP
2.7.1.20	2.7.2.3	Human	AMP
2.7.1.20	2.7.2.3	Mouse	AMP
2.7.1.20	2.7.2.3	Yeast	AMP
2.7.1.20	2.7.4.9	Human	ADP
2.7.1.20	2.7.4.9	Mouse	ADP

2.7.1.20	2.7.4.9	Yeast	ADP
2.7.1.20	2.7.6.1	Human	ADP
2.7.1.20	2.7.6.1	Mouse	ADP
2.7.1.20	2.7.6.1	Rat	ADP
2.7.1.20	3.1.3.11	Human	AMP
2.7.1.20	3.1.3.11	Mouse	AMP
2.7.1.20	3.1.3.11	Rat	AMP
2.7.1.20	3.1.3.11	Yeast	AMP
2.7.1.20	3.1.3.5	Human	ADP
2.7.1.20	3.1.3.5	Mouse	ADP
2.7.1.20	3.1.3.5	Rat	ADP
2.7.1.20	6.3.4.4	Human	AMP
2.7.1.20	6.3.4.4	Mouse	AMP
2.7.1.20	6.3.4.4	Yeast	AMP
2.7.1.21	2.7.6.1	E. coli	ADP
2.7.1.21	2.7.6.1	Human	ADP
2.7.1.21	2.7.6.1	Mouse	ADP
2.7.1.21	3.1.3.5	E. coli	ADP
2.7.1.21	3.1.3.5	Human	ADP
2.7.1.21	3.1.3.5	Mouse	ADP
2.7.1.26	2.7.4.9	Human	ADP
2.7.1.26	2.7.4.9	Mouse	ADP
2.7.1.26	2.7.4.9	Yeast	ADP
2.7.1.26	2.7.6.1	E. coli	ADP
2.7.1.26	2.7.6.1	Human	ADP
2.7.1.26	2.7.6.1	Mouse	ADP
2.7.1.26	3.1.3.5	E. coli	ADP
2.7.1.26	3.1.3.5	Human	ADP
2.7.1.26	3.1.3.5	Mouse	ADP
2.7.1.30	1.2.1.12	E. coli	ADP
2.7.1.30	1.2.1.12	Human	ADP
2.7.1.30	1.2.1.12	Rat	ADP
2.7.1.30	2.7.1.20	Human	ADP
2.7.1.30	2.7.1.20	Mouse	ADP
2.7.1.30	2.7.1.20	Rat	ADP
2.7.1.30	2.7.1.67	Human	ADP
2.7.1.30	2.7.1.67	Rat	ADP
2.7.1.30	2.7.1.67	Yeast	ADP
2.7.1.30	2.7.4.9	Human	ADP
2.7.1.30	2.7.4.9	Mouse	ADP
2.7.1.30	2.7.4.9	Yeast	ADP
2.7.1.30	2.7.6.1	E. coli	ADP
2.7.1.30	2.7.6.1	Human	ADP

2.7.1.30	2.7.6.1	Mouse	ADP
2.7.1.30	2.7.6.1	Rat	ADP
2.7.1.30	3.1.3.5	E. coli	ADP
2.7.1.30	3.1.3.5	Human	ADP
2.7.1.30	3.1.3.5	Mouse	ADP
2.7.1.30	3.1.3.5	Rat	ADP
2.7.1.32	2.7.1.20	Human	ADP
2.7.1.32	2.7.1.20	Mouse	ADP
2.7.1.32	2.7.1.20	Rat	ADP
2.7.1.32	2.7.1.67	Human	ADP
2.7.1.32	2.7.1.67	Rat	ADP
2.7.1.32	2.7.1.67	Yeast	ADP
2.7.1.32	2.7.4.9	Human	ADP
2.7.1.32	2.7.4.9	Mouse	ADP
2.7.1.32	2.7.4.9	Yeast	ADP
2.7.1.32	2.7.6.1	Human	ADP
2.7.1.32	2.7.6.1	Mouse	ADP
2.7.1.32	2.7.6.1	Rat	ADP
2.7.1.32	3.1.3.5	Human	ADP
2.7.1.32	3.1.3.5	Mouse	ADP
2.7.1.32	3.1.3.5	Rat	ADP
2.7.1.33	1.2.1.12	E. coli	ADP
2.7.1.33	1.2.1.12	Human	ADP
2.7.1.33	1.2.1.12	Rat	ADP
2.7.1.33	2.7.1.20	Human	ADP
2.7.1.33	2.7.1.20	Mouse	ADP
2.7.1.33	2.7.1.20	Rat	ADP
2.7.1.33	2.7.1.67	Human	ADP
2.7.1.33	2.7.1.67	Rat	ADP
2.7.1.33	2.7.1.67	Yeast	ADP
2.7.1.33	2.7.4.9	Human	ADP
2.7.1.33	2.7.4.9	Mouse	ADP
2.7.1.33	2.7.4.9	Yeast	ADP
2.7.1.33	2.7.6.1	E. coli	ADP
2.7.1.33	2.7.6.1	Human	ADP
2.7.1.33	2.7.6.1	Mouse	ADP
2.7.1.33	2.7.6.1	Rat	ADP
2.7.1.33	3.1.3.5	E. coli	ADP
2.7.1.33	3.1.3.5	Human	ADP
2.7.1.33	3.1.3.5	Mouse	ADP
2.7.1.33	3.1.3.5	Rat	ADP
2.7.1.40	1.2.1.12	E. coli	ADP
2.7.1.40	1.2.1.12	Human	ADP

2.7.1.40	1.2.1.12	Rat	ADP
2.7.1.40	2.7.1.20	Human	ADP
2.7.1.40	2.7.1.20	Mouse	ADP
2.7.1.40	2.7.1.20	Rat	ADP
2.7.1.40	2.7.1.67	Human	ADP
2.7.1.40	2.7.1.67	Rat	ADP
2.7.1.40	2.7.1.67	Yeast	ADP
2.7.1.40	2.7.4.9	Human	ADP
2.7.1.40	2.7.4.9	Mouse	ADP
2.7.1.40	2.7.4.9	Yeast	ADP
2.7.1.40	2.7.6.1	E. coli	ADP
2.7.1.40	2.7.6.1	Human	ADP
2.7.1.40	2.7.6.1	Mouse	ADP
2.7.1.40	2.7.6.1	Rat	ADP
2.7.1.40	3.1.3.5	E. coli	ADP
2.7.1.40	3.1.3.5	Human	ADP
2.7.1.40	3.1.3.5	Mouse	ADP
2.7.1.40	3.1.3.5	Rat	ADP
2.7.1.48	2.7.4.9	Human	ADP
2.7.1.48	2.7.4.9	Mouse	ADP
2.7.1.48	2.7.4.9	Yeast	ADP
2.7.1.48	2.7.6.1	E. coli	ADP
2.7.1.48	2.7.6.1	Human	ADP
2.7.1.48	2.7.6.1	Mouse	ADP
2.7.1.48	3.1.3.5	E. coli	ADP
2.7.1.48	3.1.3.5	Human	ADP
2.7.1.48	3.1.3.5	Mouse	ADP
2.7.1.6	2.7.4.9	Human	ADP
2.7.1.6	2.7.4.9	Mouse	ADP
2.7.1.6	2.7.4.9	Yeast	ADP
2.7.1.6	2.7.6.1	E. coli	ADP
2.7.1.6	2.7.6.1	Human	ADP
2.7.1.6	2.7.6.1	Mouse	ADP
2.7.1.6	3.1.3.5	E. coli	ADP
2.7.1.6	3.1.3.5	Human	ADP
2.7.1.6	3.1.3.5	Mouse	ADP
2.7.1.60	1.2.1.12	E. coli	ADP
2.7.1.60	1.2.1.12	Human	ADP
2.7.1.60	1.2.1.12	Rat	ADP
2.7.1.60	2.7.6.1	E. coli	ADP
2.7.1.60	2.7.6.1	Human	ADP
2.7.1.60	2.7.6.1	Rat	ADP
2.7.1.60	3.1.3.5	E. coli	ADP

2.7.1.60	3.1.3.5	Human	ADP
2.7.1.60	3.1.3.5	Rat	ADP
2.7.1.67	2.7.1.20	Human	ADP
2.7.1.67	2.7.1.20	Mouse	ADP
2.7.1.67	2.7.1.20	Rat	ADP
2.7.1.67	2.7.4.9	Human	ADP
2.7.1.67	2.7.4.9	Mouse	ADP
2.7.1.67	2.7.4.9	Yeast	ADP
2.7.1.67	2.7.6.1	Human	ADP
2.7.1.67	2.7.6.1	Mouse	ADP
2.7.1.67	2.7.6.1	Rat	ADP
2.7.1.67	3.1.3.5	Human	ADP
2.7.1.67	3.1.3.5	Mouse	ADP
2.7.1.67	3.1.3.5	Rat	ADP
2.7.1.68	2.7.1.20	Human	ADP
2.7.1.68	2.7.1.20	Mouse	ADP
2.7.1.68	2.7.1.20	Rat	ADP
2.7.1.68	2.7.1.67	Human	ADP
2.7.1.68	2.7.1.67	Rat	ADP
2.7.1.68	2.7.1.67	Yeast	ADP
2.7.1.68	2.7.4.9	Human	ADP
2.7.1.68	2.7.4.9	Mouse	ADP
2.7.1.68	2.7.4.9	Yeast	ADP
2.7.1.68	2.7.6.1	Human	ADP
2.7.1.68	2.7.6.1	Mouse	ADP
2.7.1.68	2.7.6.1	Rat	ADP
2.7.1.68	3.1.3.5	Human	ADP
2.7.1.68	3.1.3.5	Mouse	ADP
2.7.1.68	3.1.3.5	Rat	ADP
2.7.1.74	1.1.1.205	Human	AMP
2.7.1.74	1.1.1.205	Mouse	AMP
2.7.1.74	1.1.1.205	Rat	AMP
2.7.1.74	2.4.2.14	Human	AMP
2.7.1.74	2.4.2.14	Mouse	AMP
2.7.1.74	2.4.2.14	Rat	AMP
2.7.1.74	2.7.1.20	Human	ADP
2.7.1.74	2.7.1.20	Mouse	ADP
2.7.1.74	2.7.1.20	Rat	ADP
2.7.1.74	2.7.6.1	Human	ADP
2.7.1.74	2.7.6.1	Mouse	ADP
2.7.1.74	2.7.6.1	Rat	ADP
2.7.1.74	3.1.3.11	Human	AMP
2.7.1.74	3.1.3.11	Mouse	AMP

2.7.1.74	3.1.3.11	Rat	AMP
2.7.1.74	3.1.3.5	Human	ADP
2.7.1.74	3.1.3.5	Mouse	ADP
2.7.1.74	3.1.3.5	Rat	ADP
2.7.1.82	2.7.1.20	Human	ADP
2.7.1.82	2.7.1.20	Mouse	ADP
2.7.1.82	2.7.1.20	Rat	ADP
2.7.1.82	2.7.1.67	Human	ADP
2.7.1.82	2.7.1.67	Rat	ADP
2.7.1.82	2.7.1.67	Yeast	ADP
2.7.1.82	2.7.4.9	Human	ADP
2.7.1.82	2.7.4.9	Mouse	ADP
2.7.1.82	2.7.4.9	Yeast	ADP
2.7.1.82	2.7.6.1	Human	ADP
2.7.1.82	2.7.6.1	Mouse	ADP
2.7.1.82	2.7.6.1	Rat	ADP
2.7.1.82	3.1.3.5	Human	ADP
2.7.1.82	3.1.3.5	Mouse	ADP
2.7.1.82	3.1.3.5	Rat	ADP
2.7.1.91	2.7.1.20	Human	ADP
2.7.1.91	2.7.1.20	Mouse	ADP
2.7.1.91	2.7.1.20	Rat	ADP
2.7.1.91	2.7.1.67	Human	ADP
2.7.1.91	2.7.1.67	Rat	ADP
2.7.1.91	2.7.1.67	Yeast	ADP
2.7.1.91	2.7.4.9	Human	ADP
2.7.1.91	2.7.4.9	Mouse	ADP
2.7.1.91	2.7.4.9	Yeast	ADP
2.7.1.91	2.7.6.1	Human	ADP
2.7.1.91	2.7.6.1	Mouse	ADP
2.7.1.91	2.7.6.1	Rat	ADP
2.7.1.91	3.1.3.5	Human	ADP
2.7.1.91	3.1.3.5	Mouse	ADP
2.7.1.91	3.1.3.5	Rat	ADP
2.7.2.11	2.7.4.9	Human	ADP
2.7.2.11	2.7.4.9	Mouse	ADP
2.7.2.11	2.7.4.9	Yeast	ADP
2.7.2.11	2.7.6.1	E. coli	ADP
2.7.2.11	2.7.6.1	Human	ADP
2.7.2.11	2.7.6.1	Mouse	ADP
2.7.2.11	3.1.3.5	E. coli	ADP
2.7.2.11	3.1.3.5	Human	ADP
2.7.2.11	3.1.3.5	Mouse	ADP

2.7.4.3	1.2.1.12	E. coli	ADP
2.7.4.3	1.2.1.12	Human	ADP
2.7.4.3	1.2.1.12	Rat	ADP
2.7.4.3	2.7.1.20	Human	ADP
2.7.4.3	2.7.1.20	Mouse	ADP
2.7.4.3	2.7.1.20	Rat	ADP
2.7.4.3	2.7.1.67	Human	ADP
2.7.4.3	2.7.1.67	Rat	ADP
2.7.4.3	2.7.1.67	Yeast	ADP
2.7.4.3	2.7.4.9	Human	ADP
2.7.4.3	2.7.4.9	Mouse	ADP
2.7.4.3	2.7.4.9	Yeast	ADP
2.7.4.3	2.7.6.1	E. coli	ADP
2.7.4.3	2.7.6.1	Human	ADP
2.7.4.3	2.7.6.1	Mouse	ADP
2.7.4.3	2.7.6.1	Rat	ADP
2.7.4.3	3.1.3.5	E. coli	ADP
2.7.4.3	3.1.3.5	Human	ADP
2.7.4.3	3.1.3.5	Mouse	ADP
2.7.4.3	3.1.3.5	Rat	ADP
2.7.4.9	2.7.6.1	E. coli	ADP
2.7.4.9	2.7.6.1	Human	ADP
2.7.4.9	2.7.6.1	Mouse	ADP
2.7.4.9	3.1.3.5	E. coli	ADP
2.7.4.9	3.1.3.5	Human	ADP
2.7.4.9	3.1.3.5	Mouse	ADP
2.7.6.1	1.1.1.205	E. coli	AMP
2.7.6.1	1.1.1.205	Human	AMP
2.7.6.1	1.1.1.205	Mouse	AMP
2.7.6.1	1.1.1.205	Rat	AMP
2.7.6.1	2.4.2.14	E. coli	AMP
2.7.6.1	2.4.2.14	Human	AMP
2.7.6.1	2.4.2.14	Mouse	AMP
2.7.6.1	2.4.2.14	Rat	AMP
2.7.6.1	2.4.2.7	E. coli	AMP
2.7.6.1	2.4.2.7	Human	AMP
2.7.6.1	2.4.2.7	Mouse	AMP
2.7.6.1	2.7.2.3	Human	AMP
2.7.6.1	2.7.2.3	Mouse	AMP
2.7.6.1	2.7.2.3	Yeast	AMP
2.7.6.1	3.1.3.11	E. coli	AMP
2.7.6.1	3.1.3.11	Human	AMP
2.7.6.1	3.1.3.11	Mouse	AMP

2.7.6.1	3.1.3.11	Rat	AMP
2.7.6.1	3.1.3.11	Yeast	AMP
2.7.6.1	6.3.4.4	Human	AMP
2.7.6.1	6.3.4.4	Mouse	AMP
2.7.6.1	6.3.4.4	Yeast	AMP
2.7.7.8	1.2.1.12	E. coli	ADP
2.7.7.8	1.2.1.12	Human	ADP
2.7.7.8	1.2.1.12	Rat	ADP
2.7.7.8	2.7.1.20	Human	ADP
2.7.7.8	2.7.1.20	Mouse	ADP
2.7.7.8	2.7.1.20	Rat	ADP
2.7.7.8	2.7.6.1	E. coli	ADP
2.7.7.8	2.7.6.1	Human	ADP
2.7.7.8	2.7.6.1	Mouse	ADP
2.7.7.8	2.7.6.1	Rat	ADP
2.7.7.8	3.1.3.5	E. coli	ADP
2.7.7.8	3.1.3.5	Human	ADP
2.7.7.8	3.1.3.5	Mouse	ADP
2.7.7.8	3.1.3.5	Rat	ADP
3.6.1.3	2.7.1.20	Human	ADP
3.6.1.3	2.7.1.20	Mouse	ADP
3.6.1.3	2.7.1.20	Rat	ADP
3.6.1.3	2.7.6.1	Human	ADP
3.6.1.3	2.7.6.1	Mouse	ADP
3.6.1.3	2.7.6.1	Rat	ADP
3.6.1.3	3.1.3.5	Human	ADP
3.6.1.3	3.1.3.5	Mouse	ADP
3.6.1.3	3.1.3.5	Rat	ADP
3.6.3.14	1.2.1.12	E. coli	ADP
3.6.3.14	1.2.1.12	Human	ADP
3.6.3.14	1.2.1.12	Rat	ADP
3.6.3.14	2.7.1.20	Human	ADP
3.6.3.14	2.7.1.20	Mouse	ADP
3.6.3.14	2.7.1.20	Rat	ADP
3.6.3.14	2.7.1.67	Human	ADP
3.6.3.14	2.7.1.67	Rat	ADP
3.6.3.14	2.7.1.67	Yeast	ADP
3.6.3.14	2.7.4.9	Human	ADP
3.6.3.14	2.7.4.9	Mouse	ADP
3.6.3.14	2.7.4.9	Yeast	ADP
3.6.3.14	2.7.6.1	E. coli	ADP
3.6.3.14	2.7.6.1	Human	ADP
3.6.3.14	2.7.6.1	Mouse	ADP

3.6.3.14	2.7.6.1	Rat	ADP
3.6.3.14	3.1.3.5	E. coli	ADP
3.6.3.14	3.1.3.5	Human	ADP
3.6.3.14	3.1.3.5	Mouse	ADP
3.6.3.14	3.1.3.5	Rat	ADP
6.2.1.1	1.1.1.205	E. coli	AMP
6.2.1.1	1.1.1.205	Human	AMP
6.2.1.1	1.1.1.205	Mouse	AMP
6.2.1.1	2.4.2.14	E. coli	AMP
6.2.1.1	2.4.2.14	Human	AMP
6.2.1.1	2.4.2.14	Mouse	AMP
6.2.1.1	2.4.2.7	E. coli	AMP
6.2.1.1	2.4.2.7	Human	AMP
6.2.1.1	2.4.2.7	Mouse	AMP
6.2.1.1	2.7.2.3	Human	AMP
6.2.1.1	2.7.2.3	Mouse	AMP
6.2.1.1	2.7.2.3	Yeast	AMP
6.2.1.1	3.1.3.11	E. coli	AMP
6.2.1.1	3.1.3.11	Human	AMP
6.2.1.1	3.1.3.11	Mouse	AMP
6.2.1.1	3.1.3.11	Yeast	AMP
6.2.1.1	6.3.4.4	Human	AMP
6.2.1.1	6.3.4.4	Mouse	AMP
6.2.1.1	6.3.4.4	Yeast	AMP
6.2.1.3	1.1.1.205	E. coli	AMP
6.2.1.3	1.1.1.205	Human	AMP
6.2.1.3	1.1.1.205	Mouse	AMP
6.2.1.3	1.1.1.205	Rat	AMP
6.2.1.3	2.4.2.14	E. coli	AMP
6.2.1.3	2.4.2.14	Human	AMP
6.2.1.3	2.4.2.14	Mouse	AMP
6.2.1.3	2.4.2.14	Rat	AMP
6.2.1.3	2.4.2.7	E. coli	AMP
6.2.1.3	2.4.2.7	Human	AMP
6.2.1.3	2.4.2.7	Mouse	AMP
6.2.1.3	2.7.2.3	Human	AMP
6.2.1.3	2.7.2.3	Mouse	AMP
6.2.1.3	2.7.2.3	Yeast	AMP
6.2.1.3	3.1.3.11	E. coli	AMP
6.2.1.3	3.1.3.11	Human	AMP
6.2.1.3	3.1.3.11	Mouse	AMP
6.2.1.3	3.1.3.11	Rat	AMP
6.2.1.3	3.1.3.11	Yeast	AMP

6.2.1.3	6.3.4.4	Human	AMP
6.2.1.3	6.3.4.4	Mouse	AMP
6.2.1.3	6.3.4.4	Yeast	AMP
6.2.1.5	2.7.4.9	Human	ADP
6.2.1.5	2.7.4.9	Mouse	ADP
6.2.1.5	2.7.4.9	Yeast	ADP
6.2.1.5	2.7.6.1	E. coli	ADP
6.2.1.5	2.7.6.1	Human	ADP
6.2.1.5	2.7.6.1	Mouse	ADP
6.2.1.5	3.1.3.5	E. coli	ADP
6.2.1.5	3.1.3.5	Human	ADP
6.2.1.5	3.1.3.5	Mouse	ADP
6.3.1.2	1.2.1.12	E. coli	ADP
6.3.1.2	1.2.1.12	Human	ADP
6.3.1.2	1.2.1.12	Rat	ADP
6.3.1.2	2.7.1.20	Human	ADP
6.3.1.2	2.7.1.20	Mouse	ADP
6.3.1.2	2.7.1.20	Rat	ADP
6.3.1.2	2.7.1.67	Human	ADP
6.3.1.2	2.7.1.67	Rat	ADP
6.3.1.2	2.7.1.67	Yeast	ADP
6.3.1.2	2.7.4.9	Human	ADP
6.3.1.2	2.7.4.9	Mouse	ADP
6.3.1.2	2.7.4.9	Yeast	ADP
6.3.1.2	2.7.6.1	E. coli	ADP
6.3.1.2	2.7.6.1	Human	ADP
6.3.1.2	2.7.6.1	Mouse	ADP
6.3.1.2	2.7.6.1	Rat	ADP
6.3.1.2	3.1.3.5	E. coli	ADP
6.3.1.2	3.1.3.5	Human	ADP
6.3.1.2	3.1.3.5	Mouse	ADP
6.3.1.2	3.1.3.5	Rat	ADP
6.3.2.2	1.2.1.12	E. coli	ADP
6.3.2.2	1.2.1.12	Human	ADP
6.3.2.2	1.2.1.12	Rat	ADP
6.3.2.2	2.7.1.20	Human	ADP
6.3.2.2	2.7.1.20	Mouse	ADP
6.3.2.2	2.7.1.20	Rat	ADP
6.3.2.2	2.7.1.67	Human	ADP
6.3.2.2	2.7.1.67	Rat	ADP
6.3.2.2	2.7.1.67	Yeast	ADP
6.3.2.2	2.7.4.9	Human	ADP
6.3.2.2	2.7.4.9	Mouse	ADP

6.3.2.2	2.7.4.9	Yeast	ADP
6.3.2.2	2.7.6.1	E. coli	ADP
6.3.2.2	2.7.6.1	Human	ADP
6.3.2.2	2.7.6.1	Mouse	ADP
6.3.2.2	2.7.6.1	Rat	ADP
6.3.2.2	3.1.3.5	E. coli	ADP
6.3.2.2	3.1.3.5	Human	ADP
6.3.2.2	3.1.3.5	Mouse	ADP
6.3.2.2	3.1.3.5	Rat	ADP
6.3.2.3	1.2.1.12	E. coli	ADP
6.3.2.3	1.2.1.12	Human	ADP
6.3.2.3	1.2.1.12	Rat	ADP
6.3.2.3	2.7.1.20	Human	ADP
6.3.2.3	2.7.1.20	Mouse	ADP
6.3.2.3	2.7.1.20	Rat	ADP
6.3.2.3	2.7.1.67	Human	ADP
6.3.2.3	2.7.1.67	Rat	ADP
6.3.2.3	2.7.1.67	Yeast	ADP
6.3.2.3	2.7.4.9	Human	ADP
6.3.2.3	2.7.4.9	Mouse	ADP
6.3.2.3	2.7.4.9	Yeast	ADP
6.3.2.3	2.7.6.1	E. coli	ADP
6.3.2.3	2.7.6.1	Human	ADP
6.3.2.3	2.7.6.1	Mouse	ADP
6.3.2.3	2.7.6.1	Rat	ADP
6.3.2.3	3.1.3.5	E. coli	ADP
6.3.2.3	3.1.3.5	Human	ADP
6.3.2.3	3.1.3.5	Mouse	ADP
6.3.2.3	3.1.3.5	Rat	ADP
6.3.2.6	1.2.1.12	E. coli	ADP
6.3.2.6	1.2.1.12	Human	ADP
6.3.2.6	1.2.1.12	Rat	ADP
6.3.2.6	2.7.1.20	Human	ADP
6.3.2.6	2.7.1.20	Mouse	ADP
6.3.2.6	2.7.1.20	Rat	ADP
6.3.2.6	2.7.1.67	Human	ADP
6.3.2.6	2.7.1.67	Rat	ADP
6.3.2.6	2.7.1.67	Yeast	ADP
6.3.2.6	2.7.4.9	Human	ADP
6.3.2.6	2.7.4.9	Mouse	ADP
6.3.2.6	2.7.4.9	Yeast	ADP
6.3.2.6	2.7.6.1	E. coli	ADP
6.3.2.6	2.7.6.1	Human	ADP

6.3.2.6	2.7.6.1	Mouse	ADP
6.3.2.6	2.7.6.1	Rat	ADP
6.3.2.6	3.1.3.5	E. coli	ADP
6.3.2.6	3.1.3.5	Human	ADP
6.3.2.6	3.1.3.5	Mouse	ADP
6.3.2.6	3.1.3.5	Rat	ADP
6.3.4.16	2.7.1.20	Human	ADP
6.3.4.16	2.7.1.20	Mouse	ADP
6.3.4.16	2.7.1.20	Rat	ADP
6.3.4.16	2.7.6.1	Human	ADP
6.3.4.16	2.7.6.1	Mouse	ADP
6.3.4.16	2.7.6.1	Rat	ADP
6.3.4.16	3.1.3.5	Human	ADP
6.3.4.16	3.1.3.5	Mouse	ADP
6.3.4.16	3.1.3.5	Rat	ADP
6.3.4.2	1.2.1.12	E. coli	ADP
6.3.4.2	1.2.1.12	Human	ADP
6.3.4.2	1.2.1.12	Rat	ADP
6.3.4.2	2.7.1.20	Human	ADP
6.3.4.2	2.7.1.20	Mouse	ADP
6.3.4.2	2.7.1.20	Rat	ADP
6.3.4.2	2.7.1.67	Human	ADP
6.3.4.2	2.7.1.67	Rat	ADP
6.3.4.2	2.7.1.67	Yeast	ADP
6.3.4.2	2.7.4.9	Human	ADP
6.3.4.2	2.7.4.9	Mouse	ADP
6.3.4.2	2.7.4.9	Yeast	ADP
6.3.4.2	2.7.6.1	E. coli	ADP
6.3.4.2	2.7.6.1	Human	ADP
6.3.4.2	2.7.6.1	Mouse	ADP
6.3.4.2	2.7.6.1	Rat	ADP
6.3.4.2	3.1.3.5	E. coli	ADP
6.3.4.2	3.1.3.5	Human	ADP
6.3.4.2	3.1.3.5	Mouse	ADP
6.3.4.2	3.1.3.5	Rat	ADP
6.3.4.5	1.1.1.205	E. coli	AMP
6.3.4.5	1.1.1.205	Human	AMP
6.3.4.5	1.1.1.205	Mouse	AMP
6.3.4.5	1.1.1.205	Rat	AMP
6.3.4.5	2.4.2.14	E. coli	AMP
6.3.4.5	2.4.2.14	Human	AMP
6.3.4.5	2.4.2.14	Mouse	AMP
6.3.4.5	2.4.2.14	Rat	AMP

6.3.4.5	2.4.2.7	E. coli	AMP
6.3.4.5	2.4.2.7	Human	AMP
6.3.4.5	2.4.2.7	Mouse	AMP
6.3.4.5	2.7.2.3	Human	AMP
6.3.4.5	2.7.2.3	Mouse	AMP
6.3.4.5	2.7.2.3	Yeast	AMP
6.3.4.5	3.1.3.11	E. coli	AMP
6.3.4.5	3.1.3.11	Human	AMP
6.3.4.5	3.1.3.11	Mouse	AMP
6.3.4.5	3.1.3.11	Rat	AMP
6.3.4.5	3.1.3.11	Yeast	AMP
6.3.4.5	6.3.4.4	Human	AMP
6.3.4.5	6.3.4.4	Mouse	AMP
6.3.4.5	6.3.4.4	Yeast	AMP
6.3.5.5	2.7.4.9	Human	ADP
6.3.5.5	2.7.4.9	Mouse	ADP
6.3.5.5	2.7.4.9	Yeast	ADP
6.3.5.5	2.7.6.1	E. coli	ADP
6.3.5.5	2.7.6.1	Human	ADP
6.3.5.5	2.7.6.1	Mouse	ADP
6.3.5.5	3.1.3.5	E. coli	ADP
6.3.5.5	3.1.3.5	Human	ADP
6.3.5.5	3.1.3.5	Mouse	ADP
6.4.1.1	2.7.1.20	Human	ADP
6.4.1.1	2.7.1.20	Mouse	ADP
6.4.1.1	2.7.1.20	Rat	ADP
6.4.1.1	2.7.1.67	Human	ADP
6.4.1.1	2.7.1.67	Rat	ADP
6.4.1.1	2.7.1.67	Yeast	ADP
6.4.1.1	2.7.4.9	Human	ADP
6.4.1.1	2.7.4.9	Mouse	ADP
6.4.1.1	2.7.4.9	Yeast	ADP
6.4.1.1	2.7.6.1	Human	ADP
6.4.1.1	2.7.6.1	Mouse	ADP
6.4.1.1	2.7.6.1	Rat	ADP
6.4.1.1	3.1.3.5	Human	ADP
6.4.1.1	3.1.3.5	Mouse	ADP
6.4.1.1	3.1.3.5	Rat	ADP
6.4.1.2	1.2.1.12	E. coli	ADP
6.4.1.2	1.2.1.12	Human	ADP
6.4.1.2	1.2.1.12	Rat	ADP
6.4.1.2	2.7.1.20	Human	ADP
6.4.1.2	2.7.1.20	Mouse	ADP

6.4.1.2	2.7.1.20	Rat	ADP
6.4.1.2	2.7.1.67	Human	ADP
6.4.1.2	2.7.1.67	Rat	ADP
6.4.1.2	2.7.1.67	Yeast	ADP
6.4.1.2	2.7.4.9	Human	ADP
6.4.1.2	2.7.4.9	Mouse	ADP
6.4.1.2	2.7.4.9	Yeast	ADP
6.4.1.2	2.7.6.1	E. coli	ADP
6.4.1.2	2.7.6.1	Human	ADP
6.4.1.2	2.7.6.1	Mouse	ADP
6.4.1.2	2.7.6.1	Rat	ADP
6.4.1.2	3.1.3.5	E. coli	ADP
6.4.1.2	3.1.3.5	Human	ADP
6.4.1.2	3.1.3.5	Mouse	ADP
6.4.1.2	3.1.3.5	Rat	ADP
